# Supplementary material for: Clinical safety and pharmacokinetics of a novel oral niclosamide formulation compared with marketed niclosamide chewing tablets in healthy volunteers: A three-part randomized, double-blind, placebo-controlled trial
Source: PLoS One. 2025 Feb 25;20(2):e0303924. doi: 10.1371/journal.pone.0303924 (PMC11856320; doi:10.1371/journal.pone.0303924)
Supplement: S1 Table — (DOCX) [file pone.0303924.s001.docx]

**S1 Table** Summary of individual niclosamide PK parameters and Adverse Events.

| **RND** | **Dose Group**  **mg** | **AUClast**  **µg*h/mL [AUC/D]** | **Cmax**  **µg/mL [Cmax/D]** | **AEs by preferred term** | **Number of AEs** |
| --- | --- | --- | --- | --- | --- |
| 111 | 200/fasted | 1.13 | 0.18 [0.90] | No AE | 0 |
| 112 | Placebo/fasted | N/A | N/A | No AE | 0 |
| 113 | 200/fasted | 1.11 | 0.38 [1.90] | No AE | 0 |
| 114 | 200/fasted | 1.29 | 0.98 [4.90] | No AE | 0 |
| 121 | 600/fasted | 2.22 | 0.53 [0.88] | Throat clearing; Dry throat; Erythema | 3 |
| 122 | 600/fasted | 6.37 | 0.91 [1.52] | Nausea; Vomiting; Diarrhoea; Abdominal pain | 4 |
| 123 | 600/fasted | 4.48 | 1.21 [2.02] | No AE | 0 |
| 124 | Placebo/fasted | N/A | N/A | Dysmenorrhoea; Oral disorder | 2 |
| 131 | 1600/fasted | 10.6 [6.63] | 2.66 [1.66] | Oral disorder; Nausea | 2 |
| 132 | 1600/fasted | 1.19 [0.74] | 0.21 [0.13] | Faeces soft; Nausea; Dyspepsia | 3 |
| 133 | Placebo/fasted | N/A | N/A | Faeces soft | 1 |
| 134 | 1600/fasted | 9.07 [5.67] | 2.24 [1.40] | Diarrhoea; Back pain | 2 |
| 131 | 1600/fed | 19.15 [11.97] | 2.27 [1.42] | Oral disorder; Nausea | 2 |
| 132 | 1600/fed | 6.81 [4.26] | 0.98 [0.61] | Diarrhoea | 1 |
| 133 | Placebo/fed | N/A | N/A | No AE | 0 |
| 134 | 1600/fed | 7.43 [4.64] | 0.94 [0.59] | No AE | 0 |
| 211 | 1600/fed | 1.46 [0.91] | 0.62 [0.39] | No AE | 0 |
| 212 | 1600/fed | 13.6 [8.50] | 2.77 [1.73] | No AE | 0 |
| 213 | 1600/fed | 5.22 [3.26] | 1.10 [ 0.69] | Nausea | 1 |
| 214 | 1600/fed | 1.40 [0.88] | 0.20 [0.13] | Diarrhoea; Vomiting | 2 |
| 211 | 2000/fed | 4.77 [2.39] | 1.23 [0.62] | No AE | 0 |
| 212 | 2000/fed | 6.83 [3.41] | 0.73 [0.36] | No AE | 0 |
| 213 | 2000/fed | 5.02 [2.51] | 1.24 [0.62] | No AE | 0 |
| 214 | 2000/fed | 4.34 [2.17] | 0.60 [0.30] | No AE | 0 |
| 311 | 1600/fed | D1: 7.38 [4.61] D7: 4.37 [2.73] | D1: 0.86 [0.54] D7: 0.94 [0.59] | Rhinitis; Headache; Diarrhoea; Nausea; Headache | 5 |
| 312 | 1200/fed | D1: 4.19 [3.49] D7: 5.18 [4.32] | D1: 0.83 [0.69] D7: 0.81 [0.68] | Nausea; Cough; Headache; Diarrhoea; Flatulence; Puncture site pain | 6 |
| 313 | 1600/fed | D1: 6.07 [3.79] D7: 4.22 [2.64] | D1: 1.19 [0.74] D7: 0.75 [0.47] | Vomiting; Nausea; Diarrhoea; Diarrhoea | 4 |
| 314 | 1200/fed | D1: 0.40 [0.33] D7: 1.98 [1.65] | D1: 0.68 [0.57] D7: 0.61 [0.51] | Diarrhoea; Dry mouth; Rectal haemorrhage | 3 |
| 315 | Placebo/fed | N/A | N/A | Diarrhoea; Headache; Flatulence | 3 |
| 316 | Placebo/fed | N/A | N/A | Flatulence; Faeces soft | 2 |
| 317 | Placebo/fed | N/A | N/A | Diarrhoea; Fatigue | 2 |
| 318 | Placebo/fed | N/A | N/A | Nausea; Diarrhoea | 2 |
| 319 | 1200/fed | D1: 3.79 [3.16] D7: 4.95 [4.13] | D1: 0.37 [0.31] D7: 0.50 [0.42] | Diarrhoea; Headache | 2 |
| 320 | 1600/fed | D1: 14.23 [8.89] D7: 2.94 [1.84] | D1: 2.66 [1.66] D7: 0.79 [0.49] | Vomiting; Nausea; Diarrhoea | 3 |
| 321 | 1600/fed | D1: 7.03 [4.39] D7: 6.54 [4.09] | D1: 1.36 [0.85] D7: 1.16 [0.73] | Nausea; Diarrhoea | 2 |
| 322 | 1200/fed | D1: 5.89 [4.91] D7: 4.87 [4.06] | D1: 1.05 [0.88] D7: 0.82 [0.68] | Nausea; Diarrhoea | 2 |

2000 mg were administered as a chewing tablet
