# Supplementary material for: Clinical safety and pharmacokinetics of a novel oral niclosamide formulation compared with marketed niclosamide chewing tablets in healthy volunteers: A three-part randomized, double-blind, placebo-controlled trial
Source: PLoS One. 2025 Feb 25;20(2):e0303924. doi: 10.1371/journal.pone.0303924 (PMC11856320; doi:10.1371/journal.pone.0303924)
Supplement: S1 Protocol — (PDF) [file pone.0303924.s003.pdf]

[redacted]

Clinical Study Protocol

Title:	A 3-part study to investigate the safety and pharmacokinetics of a novel niclosamide solution as a treatment option for COVID-19 in combination with camostat
Compounds:	Niclosamide, Bayer AG, oral solution, 40 mg/mL<br>Active incredient: Niclosamide<br><br>Niclosamide (Yomesan® Bayer Vital GmbH) 500 mg chewing tablets<br>Active ingredient: Niclosamide
Sponsor:	Charité Research Organisation GmbH<br>Charitéplatz 1<br>10117 Berlin, Germany
Clinical Phase:	I
Protocol Code	NIC-002
EudraCT:	2020-003451-15
Investigator:	Dr. med. Maximilian Posch<br>Charité Research Organisation GmbH<br>Charitéplatz 1

Version & Effective Date:	Version 1.0, 20 AUG 2020<br>Version 2.0, 06 OCT 2020, AMD 1<br>Version 3.0, 16 FEB 2021, AMD 2
Authors:	R Schultz-Heienbrok, R Hertrampf, M Posch, Niklas Walther

[redacted]

SPONSOR INFORMATION

A 3-part study to investigate the safety and pharmacokinetics of a novel niclosamide solution as a treatment option for COVID-19 in combination with camostat

SPONSOR SIGNATORY

______________________________ ____________

Signature Date

Prof. Dr. med. Frank Wagner

CEO and CSO

[redacted]

SITE INFORMATION

A 3-part study to investigate the safety and pharmacokinetics of a novel niclosamide solution as a treatment option for COVID-19 in combination with camostat

Investigator:	Maximilian Posch, Dr. med.

INVESTIGATOR PROTOCOL AGREEMENT PAGE

I confirm I have read and understood the protocol and agree to conduct this study in accordance with it:

Dr. med. M. Posch   Date

[redacted]

TABLE OF CONTENTS

ABBREVIATIONS 7

AMENDMENTS 9

AMENDMENT 2 9

AMENDMENT 1 11

PROTOCOL SYNOPSIS 13

SCHEDULE OF ASSESSMENTS 18

SCHEDULE OF ASSESSMENTS 19

1. INTRODUCTION 21

1.1. BACKGROUND 21

1.2. RATIONALE FOR THE STUDY 22

1.3. RATIONALE FOR DOSE SELECTION 23

1.4. RISK ASSESSMENT 23

1.5. RISK ASSESSMENT ON COVID-19 PANDEMIC AND RISK MITIGATION MEASURES 24

2. STUDY OBJECTIVES AND ENDPOINTS 24

2.1. OBJECTIVES 24

2.1.1. PRIMARY OBJECTIVES 24

2.1.2. SECONDARY OBJECTIVES 25

2.2. ENDPOINTS 25

2.2.1. PRIMARY ENDPOINTS 25

2.2.2. SECONDARY ENDPOINTS 25

3. INVESTIGATIONAL PLAN 25

3.1. STUDY DESIGN 25

3.1.1. REPLACEMENTS 28

3.2. RATIONALE FOR STUDY DESIGN 28

3.3. STOPPING RULES FOR DOSE ESCALATION (PART A) 29

3.4. STOPPING RULES FOR THE 1600 MG DOSE GROUP (PART C) 30

3.5. PREMATURE TERMINATION OF THE STUDY 30

3.6. SAFETY MONITORING COMMITTEE (SMC) 30

3.7. STUDY COMPLETION 31

4. STUDY POPULATION 31

4.1. NUMBER OF SUBJECTS 31

4.2. INCLUSION CRITERIA 31

4.3. EXCLUSION CRITERIA 32

4.4. INDIVIDUAL WITHDRAWAL CRITERIA 33
4.5. STUDY POPULATION 33
4.6. PROHIBITED CONCOMITANT TREATMENT 33
4.7. DIETARY RESTRICTION AND SMOKING 33
4.8. OTHER RESTRICTIONS 34
5. METHODS AND ASSESSMENTS 34
5.1. WRITTEN INFORMED CONSENT 34
5.2. INCLUSION/EXCLUSION CRITERIA 35
5.3. DEMOGRAPHICS AND MEDICAL HISTORY 35
5.4. PHYSICAL EXAMINATION 35
5.5. HEIGHT AND WEIGHT 35
5.6. URINE PREGNANCY TEST 35
5.7. VITAL SIGNS 35
5.8. BODY TEMPERATURE 35
5.9. ECG 35
5.10. ADVERSE EVENTS 36
5.11. CONCOMITANT MEDICATION 36
5.12. HEMATOLOGY, CHEMISTRY 36
5.12.1. HEMATOLOGY, SEROLOGY AND URINALYSIS 36
5.12.2. CLINICAL CHEMISTRY 36
5.12.3. COAGULATION 36
5.13. URINE DRUG SCREEN 36
5.14. TEST FOR SARS-COV-2 INFECTION 37
5.15. PLASMA LEVEL DETERMINATION OF NICLOSAMIDE 37
6. STUDY TREATMENT 37
6.1. INVESTIGATIONAL PRODUCT 37
6.1.1. DOSE AND TIME OF ADMINISTRATION 37
6.2. METHOD OF ASSIGNING SUBJECTS TO TREATMENT GROUPS 39
6.3. BLINDING METHODS 39
6.4. EMERGENCY UNBLINDING BY THE INVESTIGATOR 39
6.5. STORAGE AND STABILITY 39
7. SAFETY MONITORING 39
7.1. DEFINITIONS 39
7.1.1. ADVERSE EVENTS (AES) 39
7.1.2. SERIOUS ADVERSE EVENTS (SAES) 40
7.1.3. SUSPECTED UNEXPECTED SERIOUS ADVERSE REACTIONS (SUSARS) 40

[redacted]

7.2. ASSESSMENT CRITERIA **40**
7.2.1. ASSESSMENT OF SEVERITY 40
7.2.2. RELATIONSHIP AND OUTCOME OF AES 41
7.3. REPORTING OF AES, SAES AND SUSARS **42**
7.3.1. TIME PERIOD AND FREQUENCY FOR DETECTING AES, SAES AND SUSARS 42
7.3.2. TIMEFRAME FOR REPORTING SAES TO SPONSOR 42
7.3.3. DOCUMENTATION OF AES AND SAES AND FOLLOW-UP PROCEDURE 42
7.4. LABORATORY ABNORMALITIES **42**
7.5. CONTRACEPTION **43**

8. STATISTICAL ANALYSIS **43**
8.1. SAMPLE SIZE ESTIMATION **44**
8.2. ANALYSIS SETS **44**
8.3. PROTOCOL DEVIATIONS AND HANDLING OF MISSING VALUES **44**

9. GENERAL STUDY CONDUCT CONSIDERATIONS **44**
9.1. REGULATORY AND ETHICAL CONSIDERATIONS **44**
9.2. INFORMED CONSENT **45**
9.3. PROTOCOL AMENDMENTS **45**
9.4. MONITORING **45**
9.5. QUALITY ASSURANCE **45**
9.6. RECORD RETENTION AND ARCHIVING **46**
9.7. DATA MANAGEMENT **46**
9.7.1. SOURCE DOCUMENTS 46
9.7.2. CASE REPORT FORMS 47
9.8. PUBLICATION **47**

10. REFERENCES **48**

LIST OF FIGURES

Figure 1 Study Design SAD Part 26

LIST OF TABLES

Table 1 Schedule of Assessments, Part A, SAD, Cohorts A1, A2, A3[1] 18
Table 2 Schedule of Assessments, Part B 19
Table 3 Schedule of Assessments, Part C, Multiple Dose 20
Table 4 Assessment of severity of an AE according to CTCAE Version 5.0 41
Table 5 Relationship and outcome of AEs....................41

Abbreviations

Abbreviation	Term
AE	Adverse Event
AMG	German Drug Law; Arzneimittelgesetz
ALT	Alanine Aminotranferase
AP	Alkaline Phosphatase
aPTT	Partial thromboplastin time
AST	Aspartate Aminotransferase
AUC	Area under the curve
CA	Competent Authority
CDASH	Clinical Data Acquisition Standards Harmonization
C_{max}	Maximum plasma concentration
CDISC SDTM	Clinical Data Interchange Standards Consortium Study Data Tabulation Model
CL/F	Total apparent systemic clearance of drug after extravascular administration, calculated as Dose/AUC_{inf}
COVID	Corona Virus Disease
CRO	Charité Research Organisation GmbH
CTCAE	Common terminology criteria for adverse events
EC	Ethics Committee
ECG	Electrocardiogram
eCRF	electronic Case Report Form
EDC	Electronic Data Capture
GGT	Gammaglutamyltransferase
EOS	End of study
ICH-GCP	International Council for Harmonisation - Good Clinical Practice
ICF	Informed Consent Form
IEC	Independent Ethics Committee
INR	International normalized ratio
IMP	Investigational Medicinal Product
IRB	Institutional Review Board
MedDRA	Medical Dictionary for Drug Regulatory Activities
MERS	Middle East Respiratory Syndrome
PEG-400	Polyethylenglycol 400 g/mol
PK	Pharmacokinetics
PI	Principal Investigator

Abbreviation	Term
PT	Preferred Term
SAE	Serious Adverse Events
SAP	Statistical Analysis Plan
SARS	Severe Acute Respiratory Syndrome
SMC	Safety Monitoring Committee
SmPC	Summary of Product Characteristics
SOC	System Organ Class
SOP	Standard Operating Procedures
SUSAR	Suspected Unexpected Serious Adverse Reactions
$T_{1/2}$	Half-life
TID	Ter in die, three times a day
t_{max}	Time of maximum analyte concentration
ULN	Upper Limit Normal

[redacted]

Amendments

Amendment 2

Rationale

Based on the evaluation of the study data from Part A of the study and considering the currently ongoing study of the niclosamide/camostat combination treatment in COVID-19 infected patients (sponsor number 201741), the following major changes of the protocol have been implemented in this Amendment 2.

1) Part B: Cohort B1 will be conducted under fed conditions
   Part A of the study shows that a single dose of niclosamide solution in a dose range between 200 mg and 1600 mg is safe and well tolerated. The highest dose of 1600 mg was repeated in the same subject under fed conditions. On average fed conditions were better tolerated without loss in bioavailability. Conducting B1 under the same conditions as cohort A3 will increase the safety and PK data base for this promising treatment approach. The aim of this study part, assessment of relative bioavailability compared to the chewing tablets, is not affected by this change.

2) Part B: Deletion of cohort B2
   Part B2 will be deleted completely. Part B2 has been designed with a view on a possible TID treatment in combination with camostat. Camostat treatment has been changed in the patient study (sponsor number 201741) to a QID dosing schedule based on new data from Ono Pharmaceuticals. Moreover, the 500 mg dosage of the oral solution would result in sub-therapeutic plasma levels. The QD high dosage administration is regarded the more promising treatment option.

3) Part C: Deletion of Camostat
   Camostat has been deleted in Part C because a study with the combination of camostat and niclosamide has already been started in COVID-19 infected patients. Thus, the combination therapy was replaced by a multiple dose design with the new oral niclosamide solution.

4) Part C: redesigned with 3 groups: medium dose niclosamide solution, high dose niclosamide solution, and placebo; all groups under fed conditions
   Currently, Part C is designed as a single arm MD safety and tolerability study for the Niclosamide/Camostat combination treatment. The combination is no longer needed to be tested (see above). Instead, it is proposed to have a 3 group MD study on niclosamide solution. Evaluation of Part A of the study has revealed that drug administration might result in liquid stool or even diarrhea. Even though clinically mild, these symptoms might have a severe impact on the therapy if correlated with reduced absorption of the active substance. The symptoms might be either due to the active substance or, more likely, to the excipient. PEG 400 is known to cause diarrhea and PEG formulations are indeed clinically used as laxatives. PEG is not absorbed but resides in the gastro-intestinal tract. It might therefore accumulate in subjects with a stool frequency less than 24 hours (administration frequency). Therefore, even though the 1600 mg dose is well-tolerated, and the active substance is unlikely to accumulate (half-life 4-5 hours), the excipient might accumulate, and the treatment might need to be discontinued. Therefore, a second dose arm of 1200 mg, containing 25% less PEG, will be tested in parallel to compare bioavailability over the course of the treatment (7 days). Based on the PK data from study Part A, 1200 mg niclosamide solution is estimated to still reach therapeutic plasma levels. The dose group of 1600 mg will be stopped if intolerable GI side effects occur. If the SMC and Investigator agree (after

reviewing the safety data), subsequent subjects in the 1600 mg dosing group will be dosed with 1200 mg niclosamide solution.

5) <u>Part C will run in parallel with Part B</u>
The current protocol foreseen submission of a substantial amendment after Part A and after Part B in order to start Part B and C, respectively.
It is now proposed to run Part B and C in parallel. Since Part B is only an extension to Part A3 plus (1600 mg under fed conditions) with the additional goal to investigate the relative bioavailability of the new niclosamide solution with the approved chewing tablets, there is no additional safety data to be expected from Part B for Part C. Therefore, both parts can now be run in parallel.

The following changes to the protocol have been implemented:

**Change**	**Sections changed**
Cohort B2 in Part B of the study has been deleted because a TID dosing regimen might lead to subtherapeutic niclosamide plasma levels (based on *in-vitro* data) and is not line with the chosen camostat regimen in a new PK study performed by Ono Pharmaceuticals.	Synopsis<br>Flow Chart, Table 2<br>3.1 Study Design<br>6.1.1 Dose and Time of Administration
Camostat has been deleted in Part C because a study with the combination of camostat and niclosamide has already been started in COVID-19 infected patients.	Synopsis<br>Flow Chart, Table 3<br>Section 1.1 Background<br>Section 1.2 Rationale for the Study<br>Section 1.4 Risk Assessment<br>Section 2.1.2 Secondary Objectives<br>Section 3.2 Rationale for the Study Design<br>Section 5.15 Plasma Level Determination of Niclosamide<br>Section 6.1.1 Dose and Time of Administration
Camostat was deleted as an IMP	Synopsis<br>Section 6.1 Investigational Product
The dosing regimen in Part C changed from a TID dose regiment to a QD dose regimen to avoid subtherapeutic niclosamide plasma levels (based on *in-vitro* data)	Synopsis<br>Section 1.3 Rationale for Dose Selection<br>Section 3.1 Study Design<br>Section 6.1.1 Dose and Time of Administration
Time points for PK blood samples were adopted in Part C	Flow chart, Table 3
The rationale for the chosen number of subjects has been adapted in Part C	Section 8.1 Sample Size Estimation
A medium dose (1200 mg) of niclosamide solution has been added in Part C to have the possibility to stop the 1600 mg dose group and continue dosing with the 1200 mg in case of intolerable GI side effects caused by the IMP or by the excipient PEG-400.<br>Stopping rules have been changed accordingly	Synopsis<br>Section 1.3 Rationale for Dose Selection<br>Section 3.1 Study Design<br>Section 3.4 Stopping Rules for Cohort 1 (1600 mg) (Part C)<br>Section 6.1.1 Dose and Time of Administration

Change	Sections changed
It has been added that Part B and Part C will be performed under fed conditions	Synopsis<br>Section 1.3 Rationale for Dose Selection<br>Section 2.1 Objectives<br>Section 3.1 Study Design<br>Section 4.7 Dietary Restriction and Smoking<br>Section 6.1.1 Dose and Time of Administration
It has been added that Part B and Part C will run in parallel, since Part B will only repeat the dose which was safe and well tolerated in Part A. There is no additional risk to start with Part C in parallel to Part B	Synopsis<br>3.1 Study Design
Because of the change in design, the maximum number of subjects changed from 32 to 28 subjects	Synopsis<br>3.1 Study Design<br>4.1 Number of Subjects<br>4.5 Study Population
Duration of subject's participation changed in Part C since the test dose on Day 1 was deleted. Part C will be performed without Camostat and with a niclosamide dose which was safe and well tolerated in Part A.	Synopsis<br>Flow Chart, Table 3<br>Section 1.4 Risk Assessment<br>Section 3.1 Study Design
The SMC Charter has been revised in line with the changes in design of Part B and C.	Section 3.6 Safety Monitoring Committee (SMC)
The processing of blood for the PK determination has been revised	Section 5.15 Plasma Level Determination of Niclosamide
The reference list has been revised accordingly	Section 10 Reference List

Amendment 1

The following changes to the protocol in response to questions from the ethics committee (EC) and competent authority (CA) during the clinical trial application have been implemented:

Change	Sections changed
Safety and PK data of Part A have to be reviewed and approved by ethics committee and authority before start of Part B	Protocol Synopsis<br>Section 3.1 Study Design has been adapted accordingly
Urine drug screen was added at Screening and Day -1	Schedule of Assessments Table 1-3<br>Section 5.13
Subjects with a positive urine drug screen are to be excluded from study	Section 4.3 Exclusion Criterion no. 12

Change	Sections changed
Part A3: The washout period was specified for clarify that at least 5 half-lives are necessary before initiating the fed cohort	Schedule of Assessments Table 1, Footnote<br>Section 3.1 Study Design
Part B: The washout period was modified for clarify that at least 5 half-lives are necessary before initiating the 2nd period	Schedule of Assessments Table 2, Footnote<br>Section 3.1 Study Design<br>Section 3.2 Rationale for Study Design
For all parts, drop-outs will be replaced in order to obtain 4 completed subjects at each dose level in Part A and 4 completed subjects in Part B	New Section 3.1.1 Replacement<br>Section 4.5 Study Population
Clarified that subjects who are not able to consume the entire contents of the meal provided, the approximate percentage of the consumed food has to be documented	Section 4.7 Dietary Restriction and Smoking<br>Section 6.1.1 Dose and time of administration
An inclusion criterion for heart rate has been added	Section 4.2 Inclusion Criterion no. 5
Lower limit of platelets should be <150000/ml instead of <100000	Section 1.4 Risk Assessment<br>Section 4.3 Exclusion Criterion no. 8
Exclusion criterion no. 5 has been amended to include an upper limit of allowed increase of bilirubin of 20% above ULN (except in case of Gilbert's disease)	Section 4.3 Exclusion criterion no. 5
The sentence mentioning that Part B may start before completion of Part A, and Part C may start before completion of Part B has been deleted	Section 3.1 Study Design
A new section including procedures for emergency unblinding has been added	Section 6.4 Emergency Unblinding by the Investigator
Defined the time points of PK and safety data to be reviewed by the SMC	Section 3.5 Safety Monitoring committee (SMC)

Furthermore, minor editorial changes, grammar, inconsistencies and typos have been corrected

Protocol Synopsis

Title:	A 3-part study to investigate the safety and pharmacokinetics of a novel niclosamide solution as a treatment option for COVID-19 in combination with camostat
Investigational Medicinal Products:	Niclosamide, Bayer AG, oral solution, 40 mg/mL, active ingredient: Niclosamide<br>Niclosamide (Yomesan® Bayer Vital GmbH) 500 mg chewing tablets, active ingredient: Niclosamide
Clinical Phase:	I
Rationale:	Niclosamide is a well-established active substance that is a promising candidate for a repurposing approach to treat COVID-19. Niclosamide is currently marketed as a chewing tablet for the treatment of intestinal worm infections. The marketed formulation is optimized for minimal drug substance absorption. Moreover, absorption levels seem to be highly variable with peaks ranging from 0.76 – 18.3 µM when administering the chewing tablets 2 g QD.<br>A niclosamide solution has been developed that is expected to release the drug substance more readily and more reproducibly.<br>In subsequent studies, niclosamide solution will be administered together with Camostat in COVID-19 infected patients. Camostat is approved for oral treatment of chronic pancreatitis and reflux oesophagitis in Japan. Camostat has been shown to effectively block viral replication in a SARS-CoV-2 animal model.<br>Since the mechanisms of actions are different, it was hypothesized that a combination of both substances might have an additive or even synergistic effect in the treatment of COVID-19 patients.<br>This 3-part study is designed to investigate (1) safety and pharmacokinetics of single ascending doses of the new niclosamide solution after fasted and fed conditions, (2) the relative bioavailability of the niclosamide solution compared to the chewing tablet, and (3) safety and pharmacokinetics of the new niclosamide solution after multiple doses in healthy volunteers.
Objectives:	Primary Objectives:<br>– To assess the safety and tolerability of single ascending doses of the niclosamide solution in healthy volunteers (Part A)<br>– To assess PK parameters of single ascending doses of the niclosamide solution in healthy volunteers (Part A)

	Secondary Objectives:<br>– To assess the PK of a single dose of the niclosamide solution under fed and fasted conditions in healthy volunteers (Part A)<br>– To assess saturation and/or dumping effects of niclosamide solution by dose escalation (Part A)<br>– To assess the relative bioavailability of the niclosamide solution compared to the already marketed chewing tablet administered as single dose under fed conditions in healthy volunteers (Part B)<br>– To assess the safety and PK of the niclosamide solution after multiple oral doses under fed conditions in healthy volunteers (Part C)
Design, Dosage and Administration	This study consists of three parts:<br>Part A is a randomized, double-blinded, placebo-controlled, single ascending dose (SAD) study with 3 planned cohorts.<br>In total 4 subjects will be enrolled in each cohort (ratio 3:1, verum : placebo).<br>Planned single oral doses are:<br>*Cohort A1*: 200 mg (fasted conditions)<br>*Cohort A2*: 600 mg (fasted conditions)<br>*Cohort A3*: 1600 mg (fasted and fed conditions)<br>Dose escalation will only be performed with the new oral solution if the previous dose was safe and the plasma concentration of 5µM (approx. 1.6 µg/ml) after 8 hours has not been reached in both subjects. If the 1600 mg is not tolerated there is an option to decrease the dose to 1200 mg niclosamide solution.<br>Cohort A3 will be repeated in the same subjects under fed conditions if the administration under fasted conditions is safe and well tolerated.<br>Part B* consists of one randomized, open-label, two-sequence, two-period crossover cohorts comparing the new niclosamide solution with the marketed chewing tablets.<br>Cohort B compares a single dose of the highest dose that was tested to be safe and tolerable in part A with the marketed chewing tablet in the approved dose of 2000 mg.<br>Approximately 4 subjects will be enrolled in this part<br>*Cohort B*

Sequence	Period 1	Period 2
1 (n=2)	Solution 1600mg under fed conditions	Chewing tablet 2000 mg under fed conditions
2 (n=2)	Chewing tablet 2000 mg under fed conditions	Solution 1600 mg under fed conditions

	*Part B will only be initiated after approval from Ethics Committee and Competent Authority via a substantial amendment, along with the relevant PK, and safety data from the niclosamide solution from Part A.<br><br><u>Part C</u> is a randomized, double-blinded, placebo-controlled multiple dose study investigating the safety and PK of the niclosamide solution over a treatment period of 7 days. This part consists of three treatment groups, which will start in parallel: Group 1 1200 mg (n=4), Group 2 1600 mg (n=4) and group 3 placebo (n=4). The 1600 mg niclosamide dose will be stopped and continued with the 1200 mg dose if the 1600 mg dose causes intolerable side effect, most likely GI side effects due to the amount of PEG-400 in the new formulation.<br><br>In total, 12 subjects will be enrolled in this part of the study.<br><br>Part B and Part C will start in parallel, since Part B will only be performed to get information about the relative bioavailability compared to the approved chewing tablets. The chosen dose of the niclosamide solution in Part B is identical with the highest dose given in Part A of the study which was safe and well tolerated. Thus, there is no additional risk to start with Part C in parallel to Part B.<br><br>A Safety Monitoring Committee (SMC) will continuously monitor subject safety and take decision on dose escalation in Part A and on initiation of Part B and C.
Number of Subjects:	The maximum number of subjects is 28
Number of Study Sites:	This is a single center trial of Charité Research Organisation GmbH, Berlin
Duration of Subject Participation:	<u>Part A</u>:<br><br>For each subject under fasted condition, the study will last max. 7 days (not including the screening period): 1 baseline assessment (Day -1), 1 treatment day (Day 1) and an EOS visit 3-5 days (Day 4 – 6) after last treatment.<br><br>For subjects under fed and fasted conditions, the study will last max. 9 days (not including the screening period).<br><br><u>Part B</u>:<br><br>For each subject, the study will last max. 9 days (not included the screening period): 1 baseline assessment (Day -1), 2 treatment periods, 1 washout phase of at least 48 hrs and an EOS visit 3-5 days after last treatment.<br><br><u>Part C</u>:<br><br>For each subject, the study will last max. 13 days (not including the screening period). 1 baseline assessment (Day -1), 1 treatment period of 7 days (days 1-7; discharge on Day 8) and an EOS visit 3-5 days after the last treatment.
Main Inclusion Criteria:	– Healthy male or female subjects

	– in good health as determined by past medical history, physical examination, vital signs and safety lab at screening<br>– between 18 to 45 years of age
Main Exclusion Criteria:	– Significant illness<br>– pregnant or lactating women
Evaluation:	The primary endpoints of the study are safety and PK.<br>Safety will be evaluated according to the following parameters:<br>– Adverse event monitoring - AEs and SAEs<br>– Physical examination<br>– Vital signs (blood pressure, pulse rate, temperature, respiratory rate)<br>– Safety lab<br>– ECG<br><br>The following PK parameters will be determined:<br>C_{max}, C_{8h}, C_{12h}, C_{24h}, T_{max}, AUC_{last}, AUC_{inf}, $T_{1/2}$, CL/F
Statistical Analysis:	All medical terms reported as adverse events (AE) are coded according to the Medical Dictionary for Regulatory Activities (MedDRA) for safety analysis. At least the primary System Organ Class (SOC) as well as the Preferred Term (PT) will be available for the statistical analysis. Incidences of AEs will be summarized by intensity and relationship to the study drug.<br>For each treatment, descriptive statistics will be calculated for plasma concentration of niclosamide at each applicable time point specified, and for the derived plasma PK parameters.<br>The primary PK parameters are C_{max}, AUC_{last} and AUC_{inf}, C_{8h}, C_{12h}, C_{24h}, $T_{1/2}$.<br>The food effect evaluation in Part A and the calculation of the relative bioavailability compared to the chewing tablets in Part B will be performed using a linear fixed effect model containing fixed effects for sequence, treatment, period and subjects within sequence for log-transformed C_{max}, AUC_{last} and AUC_{inf}, respectively. Only subjects with evaluable data for both periods will be evaluated. The point estimate of the ratio of geometric means (fed/fasted; oral solution/chewing tablets) will be provided together with a 90% confidence interval.
Justifications and Considerations	*Rationale*<br>In cell culture experiments, niclosamide has been shown to be a promising candidate for cellular SARS-CoV-2 viral clearance through stabilization of cellular autophagy. Based on EC_{50} data from these experiments, a plasma level of 3-5 µM could be an effective COVID-19 treatment. Niclosamide is currently marketed as chewing tablets only. The tablets are used to treat intestinal worm infections and therefore formulated to minimize systemic absorption. An oral solution has been developed with the aim to achieve higher and more reproducible plasma levels. This study

	is designed to obtain safety and PK data for the oral solution as compared to the chewing tablets.<br><br>*Dosing*<br><br>From the marketed product, it is known that 2 g niclosamide per day is well-tolerated.<br><br>The bioavailability is on average 10% (although there is a high variability), i.e. 200 mg. The oral solution contains 40 mg/mL. Administering 5 mL as a first dose would thus equal 200 mg. Even assuming that this is absorbed to 100%, only an equivalent of 10 % of the marketed formulation would enter systemic circulation, i.e. the average bioavailability. 100% absorption is highly unlikely. It is known that niclosamide is already metabolized in the intestine.<br><br>The highest dose of the oral solution corresponds to 1600 mg niclosamide which is still below the 2 g administered with the chewing tablets.<br><br>Part C of this study has the goal to get preliminary safety and PK data from the oral niclosamide solution after multiple dose administration in healthy volunteers.
Ethical Considerations:	The study will be conducted in compliance with Good Clinical Practices (ICH-GCP) and the Declaration of Helsinki, and in accordance with applicable legal and regulatory requirements, including archiving of essential documents.

[redacted]

Schedule of Assessments

Table 1 Schedule of Assessments, Part A, SAD, Cohorts A1, A2, A3[1]

	SCR	Baseline Check-in	Treatment phase		EOS
**Study day**	**-21 to -2**	**-1**	**1**	**2**	**4 (+2 days)**
**Domiciling**		**X**	**X**		
**Discharge**				**X**	
Informed consent	X				
Randomization		X			
Medical history	X				
Check Inclusion/exclusion criteria	X	X			
Demographics and medical history	X				
Physical examination	X	X			X
Body height	X				
Body weight	X				X
ECG	X		X[6]		X
Vital signs (BP, HR, respiratory rate)	X	X	X[4]		X
Body temperature	X	X	X[6]		X
Pregnancy test	X	X			X
Serology[3]	X				
Urine drug screen	X	X			
SARS-CoV2-test[5]	X				
Safety lab (haematology, clinical chemistry including coagulation, urinalysis)	X	X		X	X
Administration of niclosamide solution			X		
Pharmakokinetic sample			X[2]	X	
SAE / AE ConMed reporting	X	X	X	X	X

[1] In cohort 3, the administration of the oral solution in fasted state will be repeated in the same subject in fed state with the same assessments as in fasted conditions on Day 1 and Day 2. The subjects will be dosed under fed conditions as soon as the PK is available and safety data for 48 hrs, however not shorter than 5 half-lives based on the PK results of cohort A3 fasted conditions
[2] PK: pre-dose and 0.5, 1.0, 1.5, 2, 4, 6, 8, 12, 24 h post dose
[3] Serology: Anti HCV Ab, HBs Ag, Anti, HIV1 and HIV2
[4] vital signs: pre-dose, 1.0, 2.0, 4.0, 8.0 and 12 h post dose
[5] SARS CoV2-test will be done before domiciling as close to the baseline check-in as possible
[6] ECG, body temperature: pre-dose and 3 h post-dose

Schedule of Assessments

Table 2 Schedule of Assessments, Part B

	SCR	Baseline Check-in	Treatment Periods 1/2 Solution/Chewing Tablets				EOS
**Study day**	**-21 to -2**	**-1**	**1**	**2**	**3**	**4**	**6 (+2 days)**
**Domiciling**		**X**	**X**	**X**	**X**		
**Discharge**						**X**	
Informed consent	X						
Randomization		X					
Medical history	X						
Check Inclusion/exclusion criteria	X	X					
Demographics and medical history	X						
Physical examination	X	X					X
Body height	X						
Body weight	X						X
ECG	X		X[7]		X[7]		X
Vital signs (BP, HR, respiratory rate)	X	X	X[4]		X[4]		X
Body temperature	X	X	X[7]		X[7]		X
Pregnancy test	X	X					X
SARS-CoV2-test[6]	X						
Urine drug screen	X	X					
Serology[2]	X						
Safety lab (haematology, clinical chemistry incl. coagulation, urinalysis)	X	X		X		X	X
Administration of niclosamide as solution and chewing tablets			X		X		
Washout period				X[5]			
Pharmakokinetic sample			X[1]	X	X[1]	X	
SAE / AE ConMed reporting	X	X	X	X	X	X	X

[1] PK: pre-dose and 30 min, 60 min, 90 min, 2, 4, 6, 8, 12, 24 h
[2] Serology: Anti HCV Ab, HBs Ag, Anti, HIV1 and HIV2
[4] vital signs: pre-dose, 1.0, 2.0, 4.0, 8.0 and 12 h
[5] washout period of at least 48 hrs and not shorter than 5 half-lives based on the PK results of Part A
[6] SARS CoV2-test will be done before domiciling as close to the baseline check-in as possible
[7] ECG, body temperature: pre-dose and 3 h post-dose

Table 3 Schedule of Assessments, Part C, Multiple Dose

	SCR	Baseline Check-in	Treatment Period								EOS
**Study day**	**-21 to -2**	**-1**	**1**	**2**	**3**	**4**	**5**	**6**	**7**	**8**	**10 (+2 days)**
**Domiciling**		**X**	**X**	**X**	**X**	**X**	**X**	**X**	**X**		
**Discharge**										**X**	
Informed consent	X										
Randomization		X									
Medical history	x										
Check Inclusion/exclusion criteria	X	X									
Demographics and medical history	X										
Physical examination	X	X			X					X	X
Body height	X										
Body weight	X										X
ECG	X				X					X	X
Vital signs (BP, HR, respiratory rate)	X	X	X[3]		X					X	X
Body temperature	X	X	X		X						X
Pregnancy test	X	X									X
Urine drug screen	X	X									
Serology[1]	X										
SARS-CoV2-test[4]	X										
Safety lab (haematology, clinical chemistry incl.coagulation, urinalysis)	X	X			X					X	X
Administration of niclosamide solution (TID)			X	X	X	X	X	X	X		
Pharmakokinetic samples, niclosamide solution			X[2]	X[6]	X[6]	X[6]	X[6]	X[6]	X[2]		
SAE / AE ConMed reporting	X	X	X	X	X	X	X	X	X	X	X

[1]Serology: Anti HCV Ab, HBs Ag, Anti, HIV1 and HIV2
[2] PK: predose, 30 min, 60 min, 90 min, 2h, 4h, 6h, 8h, 12h, 24h post dose
[3] vital signs: pre-dose, 1.0, 2.0, 4.0, 8.0 and 12 h
[4] SARS CoV2-test will be done before domiciling as close to the baseline check-in as possible
[5] ECG, body temperature: pre-dose and 3 h post-dose
[6] PK at trough level

[redacted]

1. Introduction

1.1. Background

At the beginning of the year 2020, an epidemic caused by the new coronavirus SARS-CoV-2 causing COVID-19 began in China (Zhu et al. 2020). A total of 215 countries have reported infections. The World Health Organisation (WHO) declared the coronavirus epidemic a pandemic on 11 March 2020. This is the first pandemic known to be caused by the emergence of a new coronavirus. Since 31 December 2019 and as of 15 July 2020, 13299163 cases of COVID-19 have been reported, including 578319 deaths (Up to date, most cases have emerged in Europe (more than 2 million confirmed cases) and in the US (more than 3 million cases) (https://www.ecdc.europa.eu/en/geographical-distribution-2019-ncov-cases).

The SARS-CoV-2 virus is a beta-coronavirus, like MERS-CoV and SARS-CoV. All three of these viruses have their origins in bats.

Several trials have been set up in the last weeks to test various antiviral substances like remdesivir (a substance originally developed against Ebola virus) that showed activity against SARS-CoV-2 in vitro and against SARS and MERS-CoV both in vitro and in animal studies (Sheahan et al. 2017, Wang et al. 2020). That drug received a conditional marketing authorization in Europe which was based on rolling review of supporting data that began in April 2020.

Other drugs are under investigation. However, to date no proven pharmacological regime exist that could improve the natural course of the disease.

People with COVID-19 have had a wide range of symptoms reported – ranging from mild symptoms to severe illness. Symptoms may appear 2-14 days after exposure to the virus.

This study will primarily investigate the safety and pharmacokinetics of a new oral solution of niclosamide, a potential antiviral substance. The new formulation is being developed to be used in subsequent studies in combination with camostat for the treatment of COVID-19. Niclosamide is currently used in antihelminthic therapy and was shown to be effective in vitro against SARS-CoV-2 infections by activating cellular autophagy processes. In Part C of the study, niclosamide will be administered in a multiple dose regimen in healthy volunteers.

Niclosamide (Yomesan®):

Niclosamide is an approved drug for the treatment of tapeworm infection for more than 50 years. The highest approved dose of niclosamide is 2000 mg on Day 1, followed by 1000 mg/d up to six days. In addition to its current use as a treatment of tapeworm infection, niclosamide has also found a novel role in cancer therapy and as an antiviral agent (Xu et al. 2020).

Drug-induced modulations of cellular pathways like autophagy were shown to broadly affect virus growth (Lundi et al. 2014; Pfefferle et al. 2011). Researchers from the Institute of Virology at Charité – Universitätsmedizin Berlin recently found that highly pathogenic MERS-CoV limits the cellular recycling process (autophagy) via Beclin-1 degradation and that stabilization of the autophagy-initiating Beclin-1 protein effectively reduced propagation of MERS-CoV (Gassen et al. 2014). Niclosamide was the most promising Beclin-1-stabilizing drug reducing MERS-CoV growth *in vitro* up to 28,000-fold. In ensuing experiments, it was shown that SARS-CoV-2, similar to MERS-CoV, limits autophagy. Furthermore, niclosamide reduced SARS-CoV-2 propagation up to 16,000-fold in cell cultures. The IC_{50} of niclosamide was 0.17 µM (R^2= 0.63) at 48 hours post SARS-CoV-2 infection, being well below the observed niclosamide serum levels of 0.76-18.35 µM upon oral uptake of 2000 mg. These observations were recently confirmed and pre-published by a Korean research team (Jeon et al. 2020). In a recent study performed by the team of C. Drosten (Dept. of Virology, Charité – Universitätsmedizin Berlin) it was confirmed that niclosamide also potently inhibits SARS-CoV-2 (Hoffmann et al. 2020).

Based on EC_{50} data from these experiments, a plasma level of 3-5 µM could be an effective COVID-19 treatment. Niclosamide is currently marketed as chewing tablets only. The tablets are used to treat intestinal worm infections and therefore formulated to minimize systemic absorption.

An oral solution has been developed with the aim to achieve higher and more reproducible plasma levels.

Safety Profile of Niclosamide:

The safety profile of niclosamide is well known from the treatment of tapeworm infection and from treatment in other not approved indications.

According to the SmPC of niclosamide, the following adverse drug reactions are known from spontaneous reporting: Hypersensitivity reactions (e.g. erythema, pruritus, and exanthema), dizziness, cyanosis, gastrointestinal symptoms such as such as nausea, retching, abdominal pain and diarrhea.

In a prostate cancer study, a dose of 3000 mg in combination with enzalutamide led to prolonged grade 3 nausea, vomiting, diarrhea and colitis.

Overall, doses up to 2000 mg niclosamide were safe and well tolerated.

Niclosamide solution

A niclosamide solution has been developed with the aim to achieve higher and more reproducible plasma levels compared to the already marketed niclosamide chewing tablet. In contrast to the chewing tablet, which is formulated to minimize systemic absorption, the new oral solution is being developed to reach systemic exposure which is sufficient to be effective in the treatment of COVID-19 in combination with camostat.

The solution consists of polyethyleneglycol 400 as the main ingredient and solvent for niclosamide. It also contains plysorbate 20 as a surfactant and levomenthol as flavor. All excipients are well known and widely used in drug development. All excipients are described in the European Pharmacopoeia. Polyethylene glycol 400 is known to be a safe and has been used for many drug substances since the 1950ies (Smyth et al. 1950 and 1955, Tusing et al. 1954). It is described in the “Handbook of Pharmaceutical Excipients”. The WHO has set an acceptable daily intake of polyethylene glycols at up to 10 mg/kg body weight (World Health Organ Tech Rep Ser 1980, No 648) which is far in excess of the maximum proposed doses here. Polyethylene glycols are known drug substances themselves, used to treat obstipation. Diarrhea would therefore be the only adverse event to be expected.

This study is primarily designed to investigate the safety and pharmacokinetics of a novel niclosamide solution after single and multiple dose administration. The new formulation is being developed to be used in subsequent studies in combination with camostat for the treatment of COVID-19.

1.2. Rationale for the Study

Niclosamide is a well-established active substance that is a promising candidate for a repurposing approach to treat COVID-19. Niclosamide is currently marketed as a chewing tablet for the treatment of intestinal worm infections. The marketed formulation is optimized for minimal drug substance absorption Moreover, absorption levels seem to be highly variable with peaks ranging from 0.76 – 18.3 µM when administering the chewing tablets 2 g QD.

A niclosamide solution has been developed that is expected to release the drug substance more readily and more reproducibly.

This study is primarily designed to investigate pharmacokinetics and safety of single ascending doses of the new oral niclosamide solution. Comparison will be made with the already marketed chewing tablet. A multiple dose part with the niclosamide solution is included to

assess the safety and PK after multiple doses of niclosamide solution. The new formulation is being developed to be used in subsequent studies in combination with camostat for the treatment of COVID-19.

1.3. Rationale for Dose Selection

The niclosamide treatment regimen against tape worms comprises a 2 g dose at day 1 followed by 6 days of 1 g for individuals aged >6 years. Intestinal resorption is low, and the bioavailability is on average 10% (although there is a high variability).

Part A of the study consists of single ascending doses of the new oral solution with three planned cohorts (A1, A2, A3). The initial dose of 200 mg, in case of assuming 100% absorption, corresponds to the systemic exposure of the chewing tablet of 2000 mg assuming a bioavailability of 10%. However, complete (100%) absorption of the oral solution is highly unlikely since niclosamide is likely to be metabolized already in the intestine. The starting dose of 200 mg is therefore considered safe. The next higher dose of 600 mg is a threefold increase from the starting dose. The final dose of 1600 mg is limited by the solution excipients which are known to give raise to diarrhea at higher volumes. Both the 600 mg and 1600 mg cohorts will only be started if the previous dose is safe and the pre-defined plasma level of above 5 µM (approx. 1.6 µg/ml) after 8 hours has not been reached. If the 1600 mg dose is not tolerated, there is an option to repeat the cohort with a lower dose of 1200 mg. The last cohort will be repeated under fed conditions to investigate a possible food effect.

The first subject in each cohort will be the sentinel subject receiving an open label niclosamide solution administration.

Overall, the proposed SAD design for this study mimics conventional FIH studies, and thus, seems to be a suitably conservative approach for testing the niclosamide solution formulation.

Part B of the study compares the highest acceptable dose in Part A (1600 mg oral solution) with the highest approved dose of the chewing tablet (2000 mg) given as a QD dosing regimen under fed conditions to determine the relative bioavailability of the new oral solution formulation.

The chosen dose will not exceed the maximum tolerable dose in Part A.

Part C investigate safety and PK of the niclosamide solution over a period of 7 days.

This part consists of three groups: Group 1, 1200 mg niclosamide solution administered once daily, Group 2, 1600 mg niclosmide solution administered once daily and Group 3, placebo.

The medium dose of niclosamide has been added to have the possibility to stop dosing in the 1600 mg group and continue with 1200 mg dosing in case of intolerable side effects, which may occur by accumulation of the excipient PEG-400 in the gut.

Accumulation of niclosamide is not expected after multiple dosing considering the plasma concentration-time profiles observed in Part A of the study.

Niclosamide solution will be administered once daily under fed conditions.

1.4. Risk Assessment

The risks of the treatment is limited in the sense that investigational substance, niclosamide, has been marketed and used for decades and have thus an established safety profile. The risks in the study derive from using niclosamide as solution which most probably lead to an increase of the plasma concentration of niclosamide.

Niclosamide chewing tablets has been approved for the treatment of tapeworm infection for more than 50 years. The highest approved dose of niclosamide is 2000 mg on day 1, followed by 1000 mg/d up to seven days.

According to the SmPC of niclosamide chewing tablets (SmPC, Yomesan®), the following adverse drug reactions are known from spontaneous reporting: Hypersensitivity reactions (e.g. erythema, pruritus, and exanthema), dizziness, cyanosis, gastrointestinal symptoms such as such as nausea, retching, abdominal pain and diarrhea. The healthy volunteers will be monitored closed about these expected adverse events.

Overall, doses up to 2000 mg niclosamide chewing tablets were safe and well tolerated.

The potential risk of the niclosamide solution will be minimized by a SAD design in Part A of the study, which is comparable with a FIH study. The initial dose is low and will only be increased to the next higher dose after reviewing the safety and PK data by a SMC.

The risk to subjects in this 3-part study will be minimized by adherence to the inclusion/exclusion criteria and close clinical monitoring as well as attention to adverse effects throughout the trial. Safety will be monitored by safety lab, vital signs, physical examination, ECG and recording of adverse events.

For a detailed benefit/risk assessment please see Investigator's Brochures of niclosamide

1.5. Risk Assessment on COVID-19 Pandemic and Risk Mitigation Measures

As for the general population, there is a potential risk of a severe acute respiratory syndrome coronavirus type 2 (SARS-CoV-2) infection for study participants as long as the COVID-19 pandemic continues. In order to avoid the inclusion of SARS-CoV-2 infected subjects and to minimize the potential infection risk during study participation, only proven SARS-CoV-2 negative participants without any symptoms of a respiratory disease will be included into the study (see Section 4.3 Exclusion Criteria). During the entire study, all recommendations issued by authorities and local guidelines with respect to minimizing the risk of disease spreading, e.g. social distancing, disinfection, strict hygiene measures, and wearing of mouth-nose masks shall be followed. Details are described in a separate document provided to the EC and BfArM. All participants will be closely monitored for signs and symptoms of COVID-19 during the entire study duration. Study treatment will be immediately terminated for study participants with a SARS-CoV-2 infection or with contact to SARS-CoV-2 positive subjects. All respective subjects will be clinically followed-up according to the recommendations issued by local regulations. During the pandemic situation, further measures according to recommendations and requirements from local health authorities may become necessary and will be followed within the context of this study as far as applicable in order to ensure full implementation of the principles of GCP with priority on subject safety in this study also during the COVID-19 pandemic situation.

2. Study Objectives and Endpoints

2.1. Objectives

2.1.1. Primary Objectives

- To assess the safety and tolerability of single ascending doses of the niclosamide solution in healthy volunteers (Part A)
- To assess PK parameters of single ascending doses of the niclosamide solution in healthy volunteers (Part A)

[redacted]

2.1.2. Secondary Objectives

- To assess the PK of a single dose of the niclosamide solution under fed and fasted conditions in healthy volunteers (Part A)
- To assess saturation and/or dumping effects of niclosamide solution by dose escalation (Part A)
- To assess the relative bioavailability of the niclosamide solution compared to the already marketed chewing tablet administered as single dose under fed conditions in healthy volunteers (Part B)
- To assess the safety and PK of the niclosamide solution after multiple oral doses under fed conditions in healthy volunteers (Part C)

2.2. Endpoints

2.2.1. Primary Endpoints

Safety:

- Adverse event monitoring - AEs and SAEs
- Physical examination
- Vital sign monitoring (blood pressure, pulse rate, respiratory rate)
- ECG
- Safety lab

Single dose PK Parameters:

- C_{max}, C_{12h}, C_{24h}, T_{max}, AUC_{last}, AUC_{inf}, $T_{1/2}$, CL/F

2.2.2. Secondary Endpoints

Food effect and saturation and/or dumping effects: C_{max}, T_{max}, AUC_{last}, and others if deemed appropriate.

Multiple dose PK Parameters:

C_{maxss}, T_{maxss}, $AUC_{tau, ss}$, CL/F_{ss}, $T_{1/2}$

Others may be added if deemed necessary.

3. Investigational Plan

3.1. Study Design

The study consists of three parts:

- Part A is a randomized, double-blinded, placebo-controlled, single ascending dose (SAD) study with 3 cohorts (N= approximately 12 subjects)
- Part B is a randomized, open-label, two time two-sequence, two-period crossover study to investigate the relative bioavailability of the new niclosamide solution compared to the marketed chewing tablets as single dose under fed conditions (N= approximately 4 subjects in a crossover design)
- Part C is a randomized, double-blinded, placebo-controlled multiple dose study investigating safety and PK of the niclosamide solution (N= approximately 12 subjects).

For all three parts, each subject will participate in a screening period during which a full physical examination, medical history, vital signs, ECG evaluation and safety laboratory tests evaluation will be performed.

Part A: Single ascending dose (SAD)

Four subjects per cohort will be randomized to receive a single oral dose of the niclosamide solution or matching placebo in a 3:1 ratio.

The SAD cohorts are planned as follows:

Cohort A1: 200 mg (fasted conditions)

Cohort A2: 600 mg (fasted conditions)

Cohort A3: 1600 mg (fasted and fed conditions)

Dose escalation will only be performed with the niclosamide solution if the previous dose was safe and the plasma concentration of above 5µM after 8 hours has not been reached in all subjects in a cohort. If the 1600 mg is not tolerated there is an option to decrease the dose to 1200 mg niclosamide solution.

The last dose cohort (A3) will be repeated in the same subjects under fed conditions. The subjects will be dosed under fed conditions if the cohort A3 under fasted condition is safe and well toleration (based on 48 hrs safety data) and PK data is available.

Figure 1 Study Design SAD Part

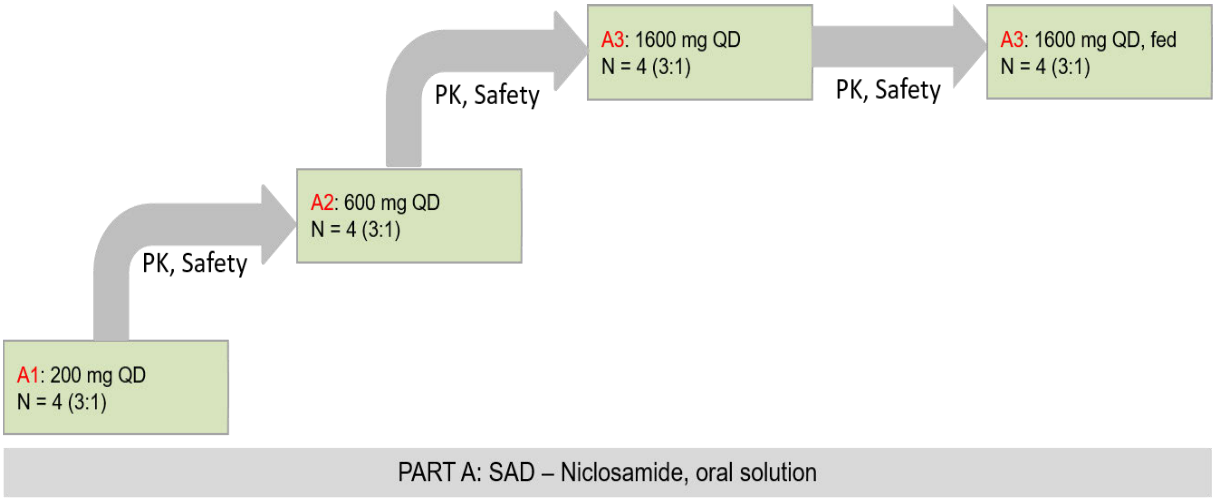

Criteria for dose escalation in SAD

A safety monitoring committee (SMC) will continuously monitor subject safety and take decision on dose escalation.

Sentinel dosing in SAD

In each cohort, the first subject will be the sentinel subject receiving an open-label niclosamide solution administration. Safety and tolerability data for this subject up to 24 hrs after dosing will be evaluated by the Investigator and sponsor and if there are no clinically relevant safety or tolerability findings, the remaining 3 subjects within the same cohort will be dosed.

SAD Part visits design:

Part A of this study is comprised of a screening visit (between Day -21 and -2), a baseline visit (Day -1), a treatment visit with a single dose of niclosamide solution or placebo (Day 1), and an End of Study (EOS) visit (between Day 4 and 6).

Subjects will remain domiciled in the study site from Day -1 until the Day 2 (24h post dose) and until assessments have been completed. Subjects will be allowed to leave the study center on Day 2 (safety signals, if any, are expected to occur within 24h from the drug administration, due to the expected short half-life of niclosamide solution) at the discretion of the Investigator, if there are no prohibitive safety findings. The subjects will be asked to return to the study site for the EOS assessments on Day 4 (+2 days). The EOS evaluation has also to be performed if a subject prematurely terminates the study for any reason.

All baseline safety evaluations results must be available prior to dosing. Subjects will fast ≥9 hours overnight between Day -1 and Day 1 pre-dose and remain fasted 4 hours post-dose on Day 1. Subjects will be randomized to receive either the niclosamide solution or matching placebo, depending on the randomization assignment.

Cohort A3 will be repeated in the same subjects under fed conditions (high fat breakfast). The subjects will be dosed under fed conditions as soon as the PK is available and safety data for 48 hrs, however not shorter than 5 half-lives based on the PK results of cohort A3 fasted conditions.

Part B relative bioavailability evaluation

Part B will only be initiated after approval from Ethics Committee and Competent Authority via a substantial amendment, along with the relevant PK, and safety data from the niclosamide solution from Part A.

Part B focus on the bioavailability of the new niclosamide solution compared to the marketed chewing tablets. Based on the PK-results of Part A, Part B will be performed under fed conditions.

This approach compared a single dose of the oral solution of the highest dose that was tested to be safe and tolerable in Part A with the marketed chewing tablet in the approved dose of 2000 mg under fed conditions.

Approximately 4 subjects will be enrolled in Part B.

Cohort B1:

**Sequence**	**Period 1**	**Period 2**
1 (n=2)	Solution 1600mg[1] QD under fed conditons	Chewing tablet 2000 mg QD under fed conditions
2 (n=2)	Chewing tablet 2000 mg QD under fed conditions	Solution 1600[1]QD under fed conditions

[1] highest dose that was tested to be safe and tolerable in Part A.

Each subject will participate in a 21-day screening period, one treatment period with a wash out period of 1 day and an EOS visit (between Day 6 and 8). The wash-out phase might be increased to 2 days depending on $T_{1/2}$ in early cohorts. The washout phase will be at least 5 times $T_{1/2}$ based on the PK results of Part A.

Subjects will remain domiciled in the study site from Day -1 until the Day 4 (24h post dose) until the safety assessments have been completed. Subjects will be allowed to leave the study center on Day 4 (safety signals, if any, are expected to occur within 24h from the drug administration, due to the expected short half-life of niclosamide solution) at the discretion of the Investigator, if there are no prohibitive safety findings. The subjects will be asked to return

to the study site for the EOS assessments on Day 6 (+2 days). The EOS evaluation has also to be performed if a subject prematurely terminates the study for any reason.

All baseline safety evaluations results must be available prior to dosing.

Part C: Multiple dose part with the niclosamide solution

The multiple dose part (Part C) will start in parallel to Part B since Part B will only be performed to get information about the relative bioavailability of the niclosamide solution compared to the approved niclosamide chewing tablets. The chosen dose of the niclosamide solution in Part B is identical with the highest dose given under fed conditions in Part A of this study, which was safe and well tolerated. Thus, there is no additional risk to start with Part C in parallel to Part B.

Part C is comprised of a screening visit (between Day -21 and Day -2), a baseline evaluation (Day -1), a multiple dose treatment period (Day 1 through Day 7, discharge on Day 8) with OD dosing of niclosamide solution and one EOS visit between Day 10 and Day 12.

Part C consists of three parallel groups:

Group 1: 1200 mg niclosamide solution (n=4)

Group 2: 1600 mg niclosamide solution (n=4)

Group 3: Placebo (n=4)

The high dose of niclosamide solution can be stopped and continue with the medium dose in the subsequent subjects, if moderate to severe GI side effects occur most likely due to an accumulation of the excipient PEG-400 in the gut.

Approximately 12 subjects (4/group) will be enrolled in this part of the study, who receive the niclosamide solution (1200 mg and 1600 mg QD) or placebo over a treatment period of 7 days. The dose of the niclosamide solution depends on the safety and PK results of Part A and will not exceed 1600 mg QD.

Subjects will remain domiciled in the study site from Day -1 until the Day 8 (24h post dose) and until all assessments have been completed. Subjects will be allowed to leave the study center on Day 8 at the discretion of the Investigator, if there are no prohibitive safety findings. The subjects will be asked to return to the study site for the EOS assessments on Day 10 (+2 days). The EOS evaluation has also to be performed if a subject prematurely terminates the study for any reason.

All baseline safety evaluations results must be available prior to dosing.

3.1.1. Replacements

Drop-outs will be replaced to obtain 4 completed subjects at the end of dose levels in Part A and to complete the planned subjects in Part B and Part C. Replacements will receive the same treatments as the drop outs.

3.2. Rationale for Study Design

A niclosamide solution has been developed that is expected to release the drug substance more readily and more reproducibly.

This 3-part study is designed to investigate (1) safety and pharmacokinetics of single ascending doses of the new niclosamide solution formulation after fasted and fed conditions, (2) the relative bioavailability of the new solution compared to the chewing tablet, and (3) safety and pharmacokinetics of multiple doses of the niclosamide solution in healthy volunteers.

Healthy volunteers: Healthy volunteers have been selected for this 3-part study to reduce the variability in the pharmacokinetics of niclosamide due to interfering factors such as drug-drug interactions with concomitant medications and underlying diseases. The findings in this population are expected to adequately predict findings in the target patient population for which the niclosamide solution is intended, i.e. COVID-19 infected patients.

SAD design (Part A): Sequentially increasing the oral solution dose from one cohort to the next allows the SMC and the investigator to get key data from the preceding cohorts prior to making a decision whether the next cohort is safe to be exposed to a higher oral solution dose.

Sentinel dosing (Part A): By initially dosing one subject of niclosamide solution in each cohort prior to dosing of the remaining subjects controls the risk of potentially high-frequency serious or severe adverse events which are acute and monitorable, such as gastrointestinal disorders. Due to the expected short half-life of niclosamide, safety signals are expected to occur within 24 h after the drug administration. Sentinel dosing will be implemented in the SAD cohorts to minimize the risk for the remainder of the cohorts. The treatment stopping rules, cohort stopping rules and study stopping rules will guide decisions on administering next doses to subjects.

Crossover (Part B): The two-sequence, two-period crossover design is considered appropriate to minimize variability of PK parameters. A washout-period of only 2 days reflect the short half-life of 2 to 5 hours of the niclosamide chewing tablet, but will be extended, if the half-life of the niclosamide solution formulation (determined in Part A) turns out to be longer. The washout-period will cover at least 5 half-lives based on the PK results in Part A.

Placebo-control: A placebo control is included in cohorts A and C that may allow to differentiate between drug-related AEs and AEs which might occur in the absence of active treatment.

Food effect: The last cohort in Part A will be repeated under fed conditions. It is expected that food influences the systemic exposure of niclosamide solution formulation.

MD design of Part C: A MD design was chosen to obtain safety and PK data over a treatment period which is comparable with the treatment period in COVID-2 infected patients.

3.3. Stopping Rules for Dose Escalation (Part A)

Safety stopping rules

Safety data of all subjects in a given cohort until Day 2 must be reviewed. If one or more of the following criteria is met, dosing will be paused at any time during the study and the SMC will be consulted.

- Occurrence of a serious adverse event (SAE) assessed as related to the IMP in at least one subject.
- Occurrence of severe (Grade ≥ 3 based on common terminology criteria for adverse events [CTCAE]) adverse events (AE) assessed as related to IMP in two subjects in the same cohort, independent of within or not within the same system-organ-class.
- Occurrence of relevant (as judged by the investigator) and confirmed safety lab deviations in two or more subjects.

The study may continue after comprehensive review by the SMC if the safety signals obtained are considered not to preclude further dosing within the cohort. Dose escalation of the niclosamide solution will only be performed if the SMC and the Investigator confirm and agree that the current emerging safety and tolerability data support dose escalation.

Stopping Rules for dose escalation based on PK data

Dose escalation to the next higher cohort will be stopped if the plasma levels of the niclosamide solution is above 5 µM (approx. 1.6 µg/ml) at 8h post-dosing in all subjects treated with niclosamide. It is noted that this limit is not informed by safety concerns but by efficacy considerations. 5 µM trough is estimated to be in a sufficiently therapeutic range.

3.4. Stopping Rules for the 1600 mg Dose Group (Part C)

The dosing of the high dose group (1600 mg) may be discontinued if moderate to severe GI side effects occur which are most likely due to accumulation of the excipient PEG-400 in the gut.

In that case, the SMC will review the safety data. Subsequent subjects in the high dose group will receive the medium dose of 1200 mg if the SMC and the Investigator confirm and agree that the safety data support this decision.

3.5. Premature Termination of the Study

The study may be terminated at any time for any reason by the sponsor.

The progress of the clinical trial with regard to safety-relevant events will be monitored and, if necessary, the premature termination of the entire clinical trial will be initiated. The entire trial must be terminated prematurely for the following reasons:

- The sponsor or the investigator consider that the number and/or severity of adverse events justify discontinuation of the study cohort
- New information is received regarding product safety or other issues arise related to the trial that preclude its completion
- Intervention of the SMC, Competent Authority (CA) or involved Ethics Committee.
- In the event of a negative change in the risk/benefit ratio, a necessary adjustment of the maximum amount of insurance cover is not possible

The reason for such a decision will be documented in writing. The respective IEC and the applicable CA will be informed in writing as appropriate.

3.6. Safety Monitoring Committee (SMC)

A SMC will monitor study progress and safety clinical data. Details of the members and procedures are described in the SMC charter.

The SMC will meet:

- Before dose escalation in Part A to decide, under consideration of the occurrence of safety issues and PK, whether treatment of the next higher dose cohort of the oral solution can be initiated. The meeting can be held if all subjects in each cohort completed their EOS visit.
- After Part A of the study to decide about the further dosing in Part B and Part C. The meeting can be held if all subjects completed their EOS visit.
- at any time if safety issues arise during the study

The SMC will have access to all safety, lab data and PK data during the study. It will decide under consideration of all safety issues and with respect to dose escalation and stopping rules as described in sections 3.3. and 3.4.

Initiation of the next higher dose in Part A and proceed with Part B and Part C can only occur after review of the following data by the SMC:

- Safety data of all subjects in a given cohort until 48 h postdose
- PK data of all subjects until 24 h postdose

3.7. Study Completion

Study completion is defined as the date when the last subject finishes the EOS visit and any assessments have been documented and followed up by the investigator. In case of an early study termination, it will be the date of the decision to terminate the study.

4. Study Population

4.1. Number of Subjects

This study will be performed in approx. maximum 28 healthy subjects.

4.2. Inclusion Criteria

Each subject must satisfy all of the following criteria to be enrolled in the study:

1. Healthy male or female subjects
2. Age 18-45 years (inclusive) at the time of informed consent signature
3. Body Mass Index (BMI) between 18.0 and 30 kg/m^2, inclusive at screening.
4. Healthy based on physical examination, medical history, vital signs, clinical laboratory tests, and 12-lead ECG performed at screening. If any of the results are abnormal, the subject may be included only if the investigator judges that the abnormalities or deviations from normal are not clinically significant.
5. A resting heart rate between 50 and 90 beats per minute
6. Men must agree to use condoms (including men who have had vasectomies) even if their partner is pregnant (this is to ensure that the fetus is not exposed to the study drug through vaginal absorption) and to not donate sperm and to not plan to father a child during the study and for 3 months after receiving the last dose of study drug. Male subjects must be counselled by the investigator that they should encourage their female partner to use a highly effective method of contraception consistent with local regulations regarding the use of contraceptive methods for subjects participating in clinical studies (e.g. prescription oral contraceptives, contraceptive injections, intrauterine device [IUD]) in addition to the condom used by the male study subject.
7. A female subject of childbearing potential must either commit to true abstinence from heterosexual contact or agree to use, and be able to comply with effective contraceptive methods with a failure rate < 1 % e.g. use of oral (estrogen and progesterone), injected or implanted hormonal methods of contraception by woman with correct and consistent use or placement of an IUD or intrauterine system (IUS) or other forms of hormonal contraception that have comparable efficacy from the time of signing the ICF and for 3 months after receiving the last dose of study drug. In addition to hormonal contraception method by woman, the male sexual partner should use a barrier method (e.g. condom).
8. Woman are considered post-menopausal and not of childbearing potential if they have had:
   - Female sterilization (have had surgical bilateral oophorectomy with or without hysterectomy), total hysterectomy or tubal ligation at least six weeks before taking

study treatment. In case of oophorectomy alone, only when the reproductive status of the woman has been confirmed by follow up hormone level assessment.

- 12 months of natural (spontaneous) amenorrhea with an appropriate clinical profile (e.g., age appropriate, history of vasomotor symptoms), confirmed by hormone level assessment.

9. Subject must have signed the informed consent form prior to the first study-related procedure indicating they understand the purpose of and procedures required for the study and are willing to participate in it.

4.3. Exclusion Criteria

1 Known or suspected hypersensitivity to trial product or related products

2 Female who is pregnant, breast-feeding or intends to become pregnant or is of child-bearing potential and not using a highly effective contraceptive method.

3 Any disorder which in the investigator's opinion might jeopardize subject's safety, evaluation of results, or compliance with the protocol.

4 Aspartate aminotransferase (AST) and alanine aminotransferase (ALT) exceeding the upper limit of normal (ULN) of the clinical laboratory's reference range at screening.

5 Total bilirubin 20% above ULN at screening (except in case of Gilbert's disease).

6 Serum creatinine exceeding the ULN and/or creatinine clearance ≤80 mL/min according to MDRD equation at screening.

7 Serum potassium > 5.5 mmol/L,

8 Platelets < 150.000/ml at screening.

9 Subject has a hepatitis B and/or C infection or human immunodeficiency virus (HIV) type-1 or HIV-2 antibodies or infection at screening.

10 Positive SARS-CoV-2 test

11 Subject has evidence of SARS-CoV-2 infection and/or subject is deemed at risk for the coronavirus disease (COVID-19) in the opinion of the treating physician or the subject has participated in another clinical study involving treatment(s), which may increase such risk.

12 Positive drug screen

13 Use of other investigational drugs at the time of enrollment, or within 5 half-lives of enrollment, or within 30 days, whichever is longer; or longer if required by local regulations.

14 Donated blood or blood products (approximately 450 mL) or lost a significant amount of blood within 2 months before the first administration of study drug.

15 Inability or unwillingness to comply with study procedures, including study prohibitions and restrictions.

16 Any condition for which, in the opinion of the investigator, participation would not be in the best interest of the subject (e.g. compromise the well-being) or that could prevent, limit, or confound the protocol-specified assessments

17 Staff member or relative of a staff member, or a subordinate relationship with the Investigator.

18 Vulnerable subject who lives in an institution on court or authority order.

4.4. Individual Withdrawal Criteria

Subjects must be withdrawn from the study if any of the following occurs:

- At their own request. At any time during the study and without giving reasons, a subject may decline to participate further. The subject will not suffer any disadvantage as a result
- If, in the investigator's opinion, continuation of the study would be harmful to the subject's well-being
- At the specific request of the sponsor and in liaison with the investigator (e.g. obvious non-compliance, safety concerns)
- Pregnancy
- Use of prohibited medication
- Any SAE reported
- CTCAE Grade 3 or higher adverse event
- Any laboratory abnormalities that in the judgment of the investigator, taking into consideration the subject’s overall status, prevents the subject from continuing participation in the study.
- Any other medically important AE in the judgement of the investigator

4.5. Study Population

The study population will be comprised of adult male and female healthy volunteers. A maximum of 28 subjects will be enrolled in the 3-part study. All subjects are expected to complete the study. Drop-outs will be replaced.

The investigator must ensure that all subjects being considered for the study meet the eligibility criteria. No additional criteria should be applied by the investigator, in order that the study population will be representative of all eligible subjects.

Subject selection is to be established by checking through all eligibility criteria at screening and baseline. A relevant record (e.g. checklist) of the eligibility criteria must be stored with the source documentation at the study site.

Deviation from **any** entry criterion excludes a subject from enrollment into the study.

No additional exclusions may be applied by the investigator to ensure that the study population will be representative of all eligible subjects.

4.6. Prohibited Concomitant Treatment

Except for medication which may be required to treat adverse events, no medication, or herbal remedies other than study drugs will be allowed from the first dosing until all of the Study Completion evaluations have been conducted.

4.7. Dietary Restriction and Smoking

- No alcohol for at least 48 hours before dosing until after Study Completion evaluation.

- No cigarettes/use of nicotine products for 48 hours before dosing until after Study Completion evaluation.
- Intake of xanthine (e.g. caffeine) containing food or beverages must be discontinued 48 hours before dosing. Consumption of such foods and beverages (i.e., coffee, tea, soda, chocolate) is not permitted at any time while the subjects are domiciled. If a deviation occurs, it must be noted in the eCRF. During the out-patient phase of the study caffeinated beverages will be restricted to no more than 4 cups/day.
- Meals should be provided when all other study procedures scheduled for that time have been completed.

Subjects in Cohort 3 of Part A will receive the oral niclosamide solution under fasted and under fed conditions.

- Under fasted condition, subjects will fast for at least 9 hours prior to administration of study medication and will continue to fast for at least 4 hours thereafter. No breakfast will be provided. No fluid intake apart from the fluid given at the time of drug intake is allowed from 2 hours before until 2 hours after dosing.
- Under fed condition, subjects will receive the standard high-fat breakfast according to the U.S. Food and Drug Administration (*Guidance for Industry: Food-Effect Bioavailability and Fed Bioequivalence Studies*.; 2002), following an overnight fast of at least 9 hours. Breakfast should be consumed within 30 minutes and the study drug should be administered within 10 minutes after completing breakfast but no more than 30 minutes from the start of the high fat breakfast. Subjects should consume the entire contents of the meal provided. If this is not possible, the approximate percentage of the consumed food should be documented.

Subjects in Part B and C will receive the oral niclosamide solution under fed conditions.

- Under fed condition, subjects will receive the standard high-fat breakfast according to the U.S. Food and Drug Administration (*Guidance for Industry: Food-Effect Bioavailability and Fed Bioequivalence Studies*.; 2002), following an overnight fast of at least 9 hours. Breakfast should be consumed within 30 minutes and the study drug should be administered within 10 minutes after completing breakfast but no more than 30 minutes from the start of the high fat breakfast. Subjects should consume the entire contents of the meal provided. If this is not possible, the approximate percentage of the consumed food should be documented.

4.8. Other Restrictions

No strenuous physical exercise (e.g. weight training, aerobics, football) for 7 days prior to dosing until after study completion evaluation.

5. Methods and Assessments

Time points of assessments are listed in the Schedule of Assessments (Tables 1-3).

5.1. Written Informed Consent

Prior to any study-specific procedure or assessment, written informed consent must be obtained from the study subject.

[redacted]

5.2. Inclusion/Exclusion criteria

Each subject must satisfy all the criteria mentioned in sections in- and exclusion criteria to be enrolled in the study.

5.3. Demographics and Medical History

Demographic data include year of birth, age and sex.

Relevant medical history data (including smoking history) includes data until screening visit. If possible, diagnoses and not symptoms will be recorded.

5.4. Physical Examination

A standard physical examination will be done at screening visit. All other examinations during the course of the study can be done by orientation on the baseline findings and actual symptoms.

Information for all physical examinations must be included in the source documentation at the study site, only medically relevant findings are recorded in the eCRF. For examinations at the Screening visit, medically relevant findings are also captured as medical history in the eCRF.

5.5. Height and Weight

Height and weight will be measured to calculate BMI. Weight will be also measured and reported before discharge or relocation.

5.6. Urine Pregnancy Test

Female subjects will provide a urine sample for pregnancy testing. If the urine test is positive, subjects will be excluded from study.

5.7. Vital Signs

Vital signs include heart rate, systolic and diastolic blood pressure, and respiratory rate Vital signs may be recorded at any time, if medically imperative for clarification of clinical signs and symptoms.

5.8. Body Temperature

Body temperature will be measured in the ear.

5.9. ECG

Only pathological and clinically relevant findings in 12-lead ECG determined on predefined study days will be documented on appropriate eCRF-pages. No records of numerical values, such as heart rate, particular times and intervals will be collected.

12-lead ECG may be recorded at any time at discretion of the responsible investigator, if medically imperative for clarification of clinical signs and symptoms. Pathological and clinically relevant findings will be documented as adverse events/serious adverse events.

5.10. Adverse Events

Adverse events will be interrogated for at each contact between the responsible investigator and the study subject. Furthermore, all pathological and clinically significant findings in physical examinations, vital signs, 12-lead ECGs, clinical chemistry including coagulation parameters, and hematology will be documented as adverse events.

Wherever possible, adverse events will be reported based on CTCAE v5.0.

Adverse events will be reported with subject-ID, start and end date, description, grading, seriousness, relationship, action taken and outcome.

5.11. Concomitant Medication

Prior and concomitant medications are recorded at the screening visit and reviewed throughout the entire study until end-of-study visit.

5.12. Hematology, Chemistry

5.12.1. Hematology, Serology and Urinalysis

Following parameters will be determined on the predefined study days: leukocytes, granulocytes, neutrophils, eosinophils, basophiles, lymphocytes, monocytes, erythrocytes, thrombocytes, hematocrit, hemoglobin.

Serum samples will be taken during screening and are analyzed for hepatitis B surface antigen (HBsAg) or a positive HBV core antigen, as well as Hepatitis C virus antibodies, and HIV antibodies ½ (Screening visit only). A polymerase chain reaction (PCR) test for HCV ribonucleic acid (RNA) will be performed in case the subject has a positive HCV antibody test result. In case of seropositivity for HIV, a second analysis by Western Blot will be performed. Results will be available as source data and will not be recorded within the eCRF. Urine will be analyzed using a dipstick only on screening. In case of positivity in a dipstick test, further appropriate analyses will be performed.

5.12.2. Clinical Chemistry

Following parameters will be determined on the predefined study days: sodium, potassium, calcium, magnesium, bicarbonate, glucose, creatinine, urea, bilirubin, AST, ALT, GGT, AP.

5.12.3. Coagulation

Following parameters will be determined on the predefined study days: aPTT and INR

5.13. Urine Drug Screen

Urine drug screen includes tests for illicit substances such as amphetamines, barbiturates, methadone, benzodiazepines, cannabinoids, cocaine and opiates, and is performed at screening visit and at Day -1. Results will be available as source data and will not be recorded within the eCRF.

5.14. Test for SARS-CoV-2 infection

A test for SARS-CoV-2 infection will be conducted during screening (as close as possible to start of confinement). This test may be repeated if clinically indicated and in accordance with local health care regulations (e.g., if the individuals' infection risk changes and/or if signs and symptoms of an infection are reported).

5.15. Plasma Level Determination of Niclosamide

Niclosamide plasma levels will be determined according to the schedules of assessments (Tables 1-3).

Niclosamide will be measured by HPLC method. Blood will be processed in the lab for generation of plasma aliquots which will be stored frozen at -80±16°C. A detailed description of the analysis can be found in Burock et al. (2018). Samples will be shipped to

Experimental and Clinical Research Center,
Charité – Universitätsmedizin Berlin, and Max-Delbrück-Center for Molecular Medicine
Robert-Rössle-Straße 10
13125 Berlin, Germany

6. Study Treatment

Study drug must be received by a designated person at the study site, handled, and stored safely and properly, and kept in a secured location to which only the investigator and designated assistants have access. Upon receipt, all study drugs should be stored according to the instructions specified on the drug labels. Clinical supplies are to be dispensed only in accordance with the protocol.

Medication labels will be in the local language and comply with the legal requirements in Germany. They will include storage conditions for the drug, but no information about the subject, except for the medication number.

6.1. Investigational Product

There are two investigational products, niclosamide in two different formulations. Furthermore, placebo will be administered in Part A and C.

Niclosamide is approved for the treatment of tapeworm infection. Niclosamide is available as 500 mg chewing tablets (Yomesan® from Bayer Vital GmbH).

The new oral solution of Bayer AG is available as a 40 mg/mL solution.

6.1.1. Dose and Time of Administration

Niclosamide will be supplied as oral solution 40 mg/mL and 500 mg chewing tablets.

The chewing tablets must be thoroughly chewed to a fine paste before swallowing with only a small amount of water. However, the tablets can also be ingested after dissolving them in water. The oral solution will be taken with approx. 240 ml of non-carbonated water.

Part A (SAD):

The new oral solution will be administered in ascending doses in cohorts A1 to A3 under fasted condition and compared to placebo. The last cohort will be repeated in the same subject under fed conditions.

Planned doses:

Cohort A1:

- Single dose of 200 mg oral niclosamide solution
- Single dose of matching placebo

Cohort A2:

- Single dose of 600 mg oral niclosamide solution
- Single dose of matching placebo

Cohort A3:

- Single dose of 1600 mg oral niclosamide solution
- Single dose of matching placebo

Part B (relative bioavailability compared to chewing tablets)

Planned doses:

- Single dose of 1600 mg oral niclosamide solution (highest dose that was tested to be safe and tolerable in Part A) under fed conditions
- Single dose of 2000 mg niclosamide chewing tablets as multiples of 500 mg chewing tablets under fed conditions

Part C (multiple doses of the niclosamide solution)

- Multiple doses of 1200 mg oral niclosamide solution under fed conditions over 7 days
- Multiple doses of 1600 mg oral niclosamide solution under fed conditions over 7 days
- Placebo under fed conditions over 7 days

In all cohorts, study drug will be administered at the study site.

In Part A cohorts, subjects must have fasted overnight for at least 9 hours before study drug intake. Intake of water is not allowed from approximately 2 hours before until approximately 2 hours after study drug intake (except for the non-carbonated water required for study drug intake). Standard lunch will be served within 15 to 30 minutes after the 4-hour PK sampling on Day 1 and in Part B also on Day 3. The lunch should be of the same composition for all subjects on Day 1. The standard lunches should be ingested entirely within 30 minutes. If this is not possible, the approximate percentage of the consumed food should be documented.

A standard snack and a standard dinner will be served approximately 7 and 10 hours postdose, respectively. This snack and dinner should be of the same composition for all subjects.

In the fed dose cohort in Part A (cohort 3) and in Part B and Part C, following overnight fast of at least 9 hours, subjects should start a high fat meal 30 minutes prior to the study drug administration at the study site between 7.30 and 10.00 AM. This meal should contain approximately 800 to 1000 calories and should derive approximately 150, 250, and 500 to 600 calories from protein, carbohydrate, and fat, respectively (according to the FDA guidance for industry). Subjects should consume the entire meal within 30 minutes. If this is not possible, the approximate percentage of the consumed food should be documented. Within 10 minutes after completing breakfast, but no more than 30 minutes from the start of their high fat meal, study drug will be administered with approximately 240 mL of non-carbonated water. Thereafter, subjects should receive standardized meals scheduled at the same time in each cohort of the study.

Times of intake of study drug, start and stop times of standard lunch and start and stop times of standard snack and dinner will be recorded on the eCRF.

6.2. Method of Assigning Subjects to Treatment Groups

After written informed consent, subjects meeting all in-/exclusion criteria will be randomized at baseline (Day -1) to one of the different treatment arms:

Randomization numbers will be assigned in ascending, sequential order to eligible patients. The investigator will document the randomization number in the source data sheet and the eCRF.

The randomization list will be kept in safe and confidential custody at the CRO. Only personnel not involved in the study will have access to the list. If one of the single verum cohorts will be stopped for safety reasons, the study will nevertheless be continued with the other study drug. The use of the randomization list will then be adapted in a way that only the randomization numbers of the remaining drug will be used.

6.3. Blinding Methods

Subjects will be randomized to receive either niclosamide or placebo in Part A and C. The treatment is supposed to be double-blinded compared to placebo. Cohort B will be conducted in an open-label design.

6.4. Emergency Unblinding by the Investigator

At any time, the investigator will have access to sealed emergency code break envelopes. At his/her discretion (e.g. in case of an emergency, or any finding that requires unblinding), the investigator may open the envelope decoding the subject’s treatment.), This will allow breaking the blinding code for an individual subject without impairing the study as a whole. At the end of the study, all code break information will be removed from the study site.

6.5. Storage and Stability

Drug supplies will be kept in their original packaging and in a secure limited access storage area according to the recommended storage conditions on the medication label. The oral solution will be protected from light.

Details can be found in the pharmacy manual.

7. Safety Monitoring

7.1. Definitions

7.1.1. Adverse Events (AEs)

An Adverse Event (AE) is defined as any untoward medical occurrence in a patient or clinical investigation subject administered a pharmaceutical product and which does not necessarily have a causal relationship with this treatment.

Thus, an AE can be any unfavorable and unintended sign (including abnormal laboratory findings), symptom, or disease temporally associated with the use of an investigational medicinal product, whether or not considered related to it.

Pre-existing diseases or symptoms or abnormal laboratory values present upon enrollment are not considered as an AE even when observed during the further course of the study. However, every worsening of a pre-existing condition is to be considered as an AE.

7.1.2. Serious Adverse Events (SAEs)

An SAE is any untoward medical occurrence that at any dose:

- Results in death.
- Is life-threatening.

  NOTE: The term "life-threatening" in this definition refers to an event in which the subject is at risk of death at the time of the event; it does not refer to an event that hypothetically might cause death if it were more severe.
- Requires or prolongs hospital stay - note that hospitalizations for the following reason should not be reported as SAEs:
  - Treatment on an emergency outpatient basis for an event not fulfilling any of the definitions of a SAE given above and not resulting in hospital admission
- Results in persistent or significant disability/incapacity.
- Is a congenital anomaly/birth defect.
- Is otherwise considered as medically important, e.g. requiring medical or surgical intervention to prevent one of the other outcomes listed in the above definition.

In case the AE fulfills one or several of these criteria, the investigator completes an SAE report form.

7.1.3. Suspected Unexpected Serious Adverse Reactions (SUSARs)

Suspected unexpected serious adverse reactions (SUSARs) are defined as any adverse reaction that is classified in nature as serious and which is not consistent with the information about the investigational product set out in the IB. Any unexpected SAE that is considered related to the Drug Product will be reported as a SUSAR.

7.2. Assessment Criteria

7.2.1. Assessment of Severity

The terms "serious" and "severe" are not synonymous. The term 'severe' should be used to describe the intensity (severity) of a specific event; the event itself, however, may be of relatively minor significance (such as severe headache). This is not the same as "serious", which is based on the existence of one of the above (section 7.1.2) mentioned seriousness criteria.

The severity of an AE is graded according to National Cancer Institute (NCI) Common Terminology Criteria for Adverse Events (CTCAE). In general, this refers to the definitions described in Table 4.

Table 4 Assessment of severity of an AE according to CTCAE Version 5.0

Grade	Clinical description of severity
1	**Mild**; asymptomatic or mild symptoms; clinical or diagnostic observations only; intervention not indicated.
2	**Moderate**; minimal, local or noninvasive intervention indicated; limiting age-appropriate instrumental ADL*.
3	**Severe or medically significant but not immediately life-threatening**; hospitalization or prolongation of hospitalization indicated; disabling; limiting self care ADL**.
4	**Life-threatening consequences;** urgent intervention indicated.
5	**Death** related to AE.

ADL: Activities of Daily Living
*Instrumental ADL refer to preparing meals, shopping for groceries or clothes, using the telephone, managing money, etc.
**Self-care ADL refer to bathing, dressing and undressing, feeding self, using the toilet, taking medications, and not bedridden

7.2.2. Relationship and Outcome of AEs

The investigator will evaluate each AE that occurred after administration of the IMP regarding the **relationship** with the administration of the IMP:

Table 5 Relationship and outcome of AEs

Related:	• There is a reasonable possibility that the event may have been caused by the IMP. A certain event has a **strong temporal relationship** and an alternative cause is unlikely.
	• An AE that has a reasonable possibility that the event is likely to have been caused by the IMP. The AE has a **timely relationship** and **follows a known pattern of response**, but a potential alternative cause may be present.
	• An AE that has a reasonable possibility that the event may have been caused by the IMP. The AE has a **timely relationship** to the IMP; **however, the pattern of response is untypical**, and an alternative cause seems more likely, or there is significant uncertainty about the cause of the event.
Non- related:	• Only a remote connection exists between the IMP and the reported adverse event. Other conditions including concurrent illness, progression or expression of the disease state or reaction of the concomitant medication appear to explain the reported adverse event.
	• An AE that does not follow a reasonable temporal sequence related to the IMP and is likely to have been produced by the subject's clinical state, other modes of therapy or other known etiology.
	• There is insufficient or incomplete evidence to make a clinical judgement of the causal relationship.

AE relationship to the study medication will be assessed at the site by the investigator and recorded on the AE page of the eCRF.

When the final causality assessment is unknown and it is uncertain whether or not the IMP caused the AE, then the AE should be handled as related to the IMP for reporting purposes.

As far as possible, each AE should be further evaluated to determine its:

- **Duration** (start and end date, time)
- **Action taken** with respect to IMP (dose interrupted, drug withdrawn, not applicable, unknown) **or** whether other action taken (to be specified, concomitant medication changed, not applicable, unknown)
- **Outcome** (not recovered/not resolved, recovered/resolved, recovering/resolving, recovered/resolved with sequelae, fatal, unknown)

7.3. Reporting of AEs, SAEs and SUSARs

Any AE that meets any criterion for an SAE defined in section 7.1.2 requires the completion of an SAE Report Form in addition to being recorded on the AE page of the eCRF, regardless of causal relationship to the study drug. SAEs occurring after EoS will only be reported in case the investigator suspects a relation to study drug.

Where possible, a diagnosis rather than a list of symptoms should be given. The investigator is responsible for assessing seriousness, severity, and causality of the SAE. The SAE form should be completed with as much information as possible. The investigator should not wait for full details before making the initial report.

According to national and international legislation all SAEs, which have been identified as SUSAR, will be reported to the respective competent authorities (CA) and ethics committee (EC).

7.3.1. Time Period and Frequency for Detecting AEs, SAEs and SUSARs

For this study, collection of all AEs will begin after providing written informed consent for participation in the study until the EOS visit.

7.3.2. Timeframe for Reporting SAEs to Sponsor

Immediate reporting should allow the sponsor to take the appropriate measures to address potential new risks in the clinical trial. Therefore, the immediate report after knowledge of a SAE should be made by the investigator within a very short period of time and under no circumstances should this exceed 24 hours following knowledge of the serious adverse event. The investigator must report the SAEs within 24 hours of knowledge by fax or other appropriate methods (e.g. via e-mail) on the SAE form to the sponsor. Personal data has to be replaced by the trial subject number before forwarding any information to the sponsor.

7.3.3. Documentation of AEs and SAEs and Follow-Up Procedure

AEs and SAEs will be documented in the eCRF with frequency, seriousness and severity, as well as the actions taken in relation to the AE (medication or other treatments). All SAEs should be followed up until clinical recovery is complete, stable condition is reached or the subject is lost to follow-up.

7.4. Laboratory Abnormalities

A clinical laboratory abnormality should be documented as an AE if any of the following conditions is met:

- judged to be of significant clinical importance
- results in discontinuation of the study
- abnormality is of a degree that requires active management.

Laboratory abnormalities that meet the criteria for AE should be followed until they have returned to normal or an adequate explanation of the abnormality is found. A Grade 3 or 4 event (severe) as per CTCAE does not automatically indicate a SAE unless it meets the definition of serious as defined above.

7.5. Contraception

Reproductive toxicity and teratogenicity data are not sufficiently reliable for the investigational drug. As this study enrolls women as well who are considered to be of childbearing potential, all suitable measures must be taken to avoid falling pregnant. Male participants and female participants of childbearing potential must maintain highly effective contraception methods starting from signing of the informed consent form and for at least 3 months after the last dose of study treatment.

Highly effective methods of birth control for female participants and female partners of male participant who are able to become pregnant include the methods below (according to the CTFG Recommendations related to contraception and pregnancy testing in clinical methods with a failure rate of less than 1% per year):

- Total abstinence from heterosexual intercourse (when this is in line with the preferred and usual lifestyle of the subject). Periodic abstinence (e.g. calendar, ovulation, symptothermal, post-ovulation methods) and withdrawal are not acceptable methods of contraception.
- Female sterilization (surgical bilateral oophorectomy with or without hysterectomy), total hysterectomy or tubal ligation at least six weeks before taking investigational drug. In case of oophorectomy alone, only when the reproductive status of the woman has been confirmed by follow up hormone level assessment.
- Male sterilization (at least 6 months prior to drug intake). For female subjects on the study the vasectomized male partner must be the sole partner for that woman.
- Use of oral (estrogen and progesterone), injected or implanted hormonal methods of contraception by woman with correct and consistent use or placement of an IUD or intrauterine system (IUS) or other forms of hormonal contraception that have comparable efficacy (failure rate <1 %), for example hormone vaginal ring or transdermal hormone contraception. In case of use of oral contraception, women should be stable on the same product for a minimum of 3 months before taking investigational drug. In addition to hormonal contraception method by woman, the male sexual partner should use a barrier method (e.g. condom).

Men must avoid sperm donation for up to 90 days after treatment

8. Statistical Analysis

All medical terms reported as adverse events (AE) are coded according to the Medical Dictionary for Regulatory Activities (MedDRA) for safety analysis. At least the primary System Organ Class (SOC) as well as the Preferred Term (PT) will be available for the statistical analysis. Incidences of AEs will be summarized by intensity and relationship to the study drug.

Descriptive statistics will be calculated for plasma concentration of niclosamide at each applicable time point specified, and for the derived plasma PK parameters.

The primary PK parameters are C_{max}, AUC_{last} and AUC_{inf}, C_{8h}, C_{12h}, $C_{24h,}$ $T_{1/2}$.

The food effect evaluation in Part A and the relative bioavailability compared to the chewing tablets in Part B will be performed using a linear fixed effect model containing fixed effects for sequence, treatment, period and subjects within sequence for log-transformed C_{max}, AUC_{last}

and AUC_{inf}, respectively. Only subjects with evaluable data for both periods. The point estimate of the ratio of geometric means (fed/fasted; oral solution/chewing tablets) will be provided together with a 90% confidence interval

8.1. Sample Size Estimation

No formal statistical hypothesis testing is planned for this study due to the exploratory and descriptive nature of the study. All subject data will be listed. All analyses will be descriptive, providing summary statistics (number of observations, arithmetic mean, standard deviation, minimum, median and maximum) for quantitative data, and frequency tables (absolute and relative frequencies) for qualitative/ordinal data.

For Part A, the sample size of 4 subjects with a randomization ratio of 3:1 is chosen to balance the need to maximize information on the niclosamide solution and the need to have a placebo subject to blind the study. Placebo subjects in Part A will be pooled, if appropriate.

Four subjects (in a crossover design) in Part B seems large enough to compare the two different niclosamide formulations and to get preliminary safety data of and PK data of the new oral solution.

Eight subjects on verum (together in the low and high dose group) seems large enough to have an adequate safety and PK data set in a multiple dose setting (Part C) considering the preliminary PK and safety data from Part A of this study.

A more technical and detailed elaboration of the statistical analysis will be included in a separate Statistical Analysis Plan (SAP).

8.2. Analysis Sets

For all analysis sets, subjects will be analyzed according to the study treatment received.

- The safety analysis set will include all subjects that received any study drug.
- The PK analysis set will include all subjects with at least one available valid PK concentration measurement, who received any study drug and with no protocol deviations that impact on PK data.
- Per protocol analysis set: Defined as all subjects completing the study without major protocol deviations

8.3. Protocol Deviations and Handling of Missing Values

Data from subjects who prematurely terminate the study will be used up to the maximum extent possible.

All protocol non-compliances will be listed and the reasons for exclusion of subjects from any of the analysis sets will be listed. Where relevant, the data from these subjects will be described separately.

9. General Study Conduct Considerations

9.1. Regulatory and Ethical Considerations

The trial will be carried out in compliance with the protocol, the ethical principles laid down in the Declaration of Helsinki, in accordance with the ICH Harmonized Tripartite Guideline for Good Clinical Practice (GCP), and other relevant regulations.

[redacted]

9.2. Informed Consent

Prior to performing any other study-related procedure, each potential subject must provide signed acknowledgement of their freely given informed consent. Either the investigator or a designated person will explain the aims, methods, anticipated benefits and potential hazards of this protocol and any discomfort it may entail. A corresponding written explanation will also be provided (informed consent form; ICF) and the subject allowed sufficient time to consider the information.

Prior to signing the consent form, the subject will be given an opportunity to discuss any issues concerning this protocol with a physician who has suitable knowledge of the procedures involved and to have all questions answered openly and honestly.

If the subject is willing to participate, the ICF will be signed and personally dated by the subject and the physician taking the consent. The subject will receive a signed copy.

9.3. Protocol Amendments

Any significant change in the study requirements, design or scheduled activities requires a protocol amendment to be issued. The investigator must not make any changes to the study or deviate from this protocol without competent regulatory authority(s), ethical committee and sponsor approval, except when necessary to avoid immediate danger to subjects. A change in procedures specified in this protocol that is intended to remove an immediate danger to subjects may be implemented immediately. The change must be documented and reported to the ethical committee and the appropriate regulatory authority(s). An appropriate amendment to the protocol is to be implemented.

All protocol amendments must be reviewed and approved in the same manner as the protocol.

9.4. Monitoring

Monitoring will be conducted by regular on-site monitoring visits and inhouse data quality review. The frequency of site monitoring will be determined by assessing all characteristics of the study, including its nature, objective and methodology.

Monitors will review every subject’s medical records and other source data to ensure consistency and/or identify omissions compared to the eCRF. Clinical monitoring will be performed by the Charité Research Organisation GmbH according to local SOP.

The investigator/institution will allow study-related site monitoring, audits, IRB/IEC review and regulatory inspections. Direct access must be provided to the eCRF and all source documents/data, including progress notes, copies of laboratory and medical test results, which must be available at all times for review by the CRA, auditor and regulatory inspector. They may review all eCRFs and informed consents. The accuracy of the data will be verified by direct comparison with the source documents. The sponsor will also monitor compliance with the protocol and eGCP.

9.5. Quality Assurance

High scientific quality of the study will be assured through several measures. Pre-study initiation visits will be performed checking all prerequisites and established procedures and issuing reports on possible shortcomings which will have to be overcome by the study site. At the same time, extensive on-site training, especially regarding the therapeutic interventions and procedures, will be given. On regular basis, completeness and validity of data and correctness of procedures will be verified and sources of missing data will be identified by monitoring visits. Source data verification will be conducted for 100 % of subjects.

Additional quality assurance measures comprise: Plausibility checks during data entry and daily comprehensive plausibility checks on the database with active query management.

A quality assurance audit/inspection of this study may be conducted by the sponsor, sponsor's designees, or by IRB/IEC or by regulatory authorities. The quality assurance auditor will have access to all medical records, the investigator's trial-related files and correspondence, and the informed consent documentation of this clinical study.

9.6. Record Retention and Archiving

The investigator is to retain and archive one copy of all data generated in the course of the study. This shall include, but not be limited to, those items defined by GCP as essential.

Records shall be retained securely and under appropriate conditions for their preservation for a minimum period of 15 years.

All study documents and drugs will be stored at the investigational site in accordance with GCP and GMP requirements. Records and documents, including signed informed consent forms, pertaining to the conduct of this trial will be retained by the investigator for 15 years after end of trial. All clinical data will be recorded, handled, and stored in a way that allows its accurate reporting. Data associated with biological samples will be transferred to laboratories in accordance with the Laboratory Manual.

9.7. Data Management

For each subject screened, an electronic eCRF will be completed within the Electronic Data Capture (EDC) system and approved by the investigator.

All data entered in the eCRF will be verifiable in source documentation other than the eCRF. Source documents are filed at the trial site according to local standard operating procedure (SOP). Trained study site staff will be responsible for entering subject source data into the validated EDC system. Data captured electronically will be immediately saved to the applicable database and changes tracked to provide an audit trail. Data validation procedures will be applied by Data Management to each stage of data handling to ensure that all data are reliable and have been processed correctly.

CDASH (Clinical Data Acquisition Standards Harmonization) format will be applied to EDC setup to ensure adaptation to CDISC SDTM (Clinical Data Interchange Standards Consortium Study Data Tabulation Model).

Data Management SOPs are in place for EDC Setup, EDC User Management, Data Validation and Database Lock. The EDC database will be locked once all expected data captured in EDC, all discrepancies resolved, all external data reconciled, all medical/surgical terms and medication classified by latest versions of Medical and Drug dictionaries, all SAE reconciled with the safety database.

9.7.1. Source Documents

Source documents are defined as original documents, data, and records. These may include hospital records, clinical and office charts, laboratory data/information or evaluation checklists, pharmacy dispensing and other records, recorded data from automated instruments etc. Source document data may be transcribed onto case report forms (CRFs) as required. Data collected during this study must be recorded on the appropriate source document.

The investigator/institution will permit study-related monitoring, audits, IEC/IRB review, and regulatory inspection(s), providing direct access to source data documents.

9.7.2. **Case Report Forms**

For each subject screened, an eCRF will be completed and approved by the investigator or an authorized sub-investigator. It is the responsibility of the investigator to ensure that the eCRFs are totally completed, and that the data therein are supported by source documentation.

The eCRF must be kept in order and up-to-date so that they always reflect the latest observations on the subject. Monitors will perform source data verification (SDV) as defined for the study.

Discrepancies and queries can only be corrected by the investigator(s) or other authorized site personnel. The study monitor should provide guidance to investigator(s) and the investigator(s)' designated representatives on making such corrections. All eCRFs will be approved and signed by the Investigator prior to database lock.

9.8. Publication

Upon study completion and finalization of the study report the results of this trial may be submitted for publication (e.g. peer-reviewed journal).

10. References

Burock S, Daum S, Keilholz U, Neumann K, Walther W, Stein U. Phase II trial to investigate the safety and efficacy of orally applied niclosamide in patients with metachronous or sychronous metastases of a colorectal cancer progressing after therapy: the NIKOLO trial. BMC Cancer. 2018;18(1):297.

Gassen NC, Niemeyer D, Muth D, Corman VM, Martinelli S, Gassen A, et al. SKP2 attenuates autophagy through Beclin1-ubiquitination and its inhibition reduces MERS-Coronavirus infection. Nat Commun. 2019;10(1):5770.

Hirano T, Murakami M. COVID-19: A New Virus, but a Familiar Receptor and Cytokine Release Syndrome. Immunity. 2020.

Hoffmann M, Schroeder S, Kleine-Weber H, Muller MA, Drosten C, Pohlmann S. Nafamostat mesylate blocks activation of SARS-CoV-2: New treatment option for COVID-19. Antimicrob Agents Chemother. 2020.

Hoffmann M, Kleine-Weber H, Schroeder S, Kruger N, Herrler T, Erichsen S, et al. SARS-CoV-2 Cell Entry Depends on ACE2 and TMPRSS2 and Is Blocked by a Clinically Proven Protease Inhibitor. Cell. 2020.

Jeon S, Ko M, Lee J, Choi I, Byun SY, Park S, et al. Identification of antiviral drug candidates against SARS-CoV-2 from FDA-approved drugs. bioRxiv. 2020:2020.03.20.999730.

Lundin A, Dijkman R, Bergstrom T, Kann N, Adamiak B, Hannoun C, et al. Targeting membrane-bound viral RNA synthesis reveals potent inhibition of diverse coronaviruses including the middle East respiratory syndrome virus. PLoS Pathog. 2014;10(5):e1004166.

Ohkoshi M. Oka T. Clincial experience with a protease inhibitor methanesulfate for prevention of recurrence of carcinoma of the mouth in treatment of terminal caricnom. J. Maxillofax Sug. 1984, 12: 148-152

Pfefferle S, Schopf J, Kogl M, Friedel CC, Muller MA, Carbajo-Lozoya J, et al. The SARS-coronavirus-host interactome: identification of cyclophilins as target for pan-coronavirus inhibitors. PLoS Pathog. 2011;7(10):e1002331.

Sheahan TP, Sims AC, Graham RL, Menachery VD, Gralinski LE, Case JB, et al. Broad-spectrum antiviral GS-5734 inhibits both epidemic and zoonotic coronaviruses. Sci Transl Med. 2017;9(396).

Smyth HFJr et al. The toxicology of the polyethylene glycols. J Am Pharm Assoc (Sci) 1950, 39: 349-354

Smyth HF et al. The chronic oral toxicology of the polyethylene glycols. J Am pharm Assoc 1955, 44: 27-30

Tusing TW et al. The chronic dermal toxicity of a series of polyethylne glycols. J Am Pharm Assoc (Sci) 1954, 43: 489-490

U.S. Food and Drug Administration. *Guidance for Industry: Food-Effect Bioavailability and Fed Bioequivalence Studies*.; 2002.

Wang M, Cao R, Zhang L, Yang X, Liu J, Xu M, et al. Remdesivir and chloroquine effectively inhibit the recently emerged novel coronavirus (2019-nCoV) in vitro. Cell Res. 2020;30(3):269-71.

Xu J, Shi PY, Li H, Zhou J. Broad Spectrum Antiviral Agent Niclosamide and Its Therapeutic Potential. ACS Infect Dis. 2020.

Zhu N, Zhang D, Wang W, Li X, Yang B, Song J, et al. A Novel Coronavirus from Patients with Pneumonia in China, 2019. N Engl J Med. 2020;382(8):727-33.
