# Supplementary material for: Clinical safety and pharmacokinetics of a novel oral niclosamide formulation compared with marketed niclosamide chewing tablets in healthy volunteers: A three-part randomized, double-blind, placebo-controlled trial
Source: PLoS One. 2025 Feb 25;20(2):e0303924. doi: 10.1371/journal.pone.0303924 (PMC11856320; doi:10.1371/journal.pone.0303924)
Supplement: S1 Listings — (PDF) [file pone.0303924.s004.pdf]

Statistical Output

Listings

Version 0.2 (03DEC2021)

Study: NIC-002

A 3-part study to investigate the safety and pharmacokinetics of a novel niclosamide solution as a treatment option for COVID-19 in combination with camostat

Sponsor: Charité Research GmbH

Author: GCP-Service Int. Ltd. & Co.KG
Matthes Metz
Anne-Conway-Straße 2
28359 Bremen, Germany

Table of contents

Listing 16.2.1.1: Subject enrolment 3
Listing 16.2.1.2: Subject Randomization 6
Listing 16.2.1.3: Subject Discontinuations.......... 9
Listing 16.2.2: Protocol deviations.......... 12
Listing 16.2.4: Subject Demographics 13
Listing 16.2.5: Subject Compliance 16
Listing 16.2.7: Adverse events listing 19
Listing 16.2.8: Laboratory values.......... 30
Listing 16.2.9: Vital signs 214
Listing 16.2.10: Other data.......... 228

Listing 16.2.1.1: Study subject data
Subject enrolment

Part A

					Fasting conditions				Fed conditions			
Cohort	Subj. ID	IC Date	Screening	IE	Base-line	Day 1	Day 2	Follow-up	Base-line	Day 1	Day 2	Follow-up
Cohort A1	2	05NOV2020	05NOV2020	Yes	16NOV2020	17NOV2020	18NOV2020	20NOV2020				
	5	05NOV2020	05NOV2020	Yes	18NOV2020	19NOV2020	20NOV2020	22NOV2020				
	7	05NOV2020	05NOV2020	Yes	18NOV2020	19NOV2020	20NOV2020	22NOV2020				
	8	06NOV2020	06NOV2020	Yes	18NOV2020	19NOV2020	20NOV2020	22NOV2020				
Cohort A2	13	11NOV2020	11NOV2020	Yes	30NOV2020	01DEC2020	02DEC2020	04DEC2020				
	19	18NOV2020	18NOV2020	Yes	02DEC2020	03DEC2020	04DEC2020	08DEC2020				
	21	23NOV2020	23NOV2020	Yes	02DEC2020	03DEC2020	04DEC2020	06DEC2020				
	22	25NOV2020	25NOV2020	Yes	02DEC2020	03DEC2020	04DEC2020	06DEC2020				
Cohort A3	24	21DEC2020	21DEC2020	Yes	05JAN2021	06JAN2021	07JAN2021	09JAN2021	19JAN2021	20JAN2021	21JAN2021	25JAN2021
	25	21DEC2020	21DEC2020	Yes	07JAN2021	08JAN2021	09JAN2021	11JAN2021	21JAN2021	22JAN2021	23JAN2021	25JAN2021
	27	21DEC2020	21DEC2020	Yes	07JAN2021	08JAN2021	09JAN2021	11JAN2021	21JAN2021	22JAN2021	23JAN2021	25JAN2021
	28	21DEC2020	21DEC2020	Yes	07JAN2021	08JAN2021	09JAN2021	11JAN2021	21JAN2021	22JAN2021	23JAN2021	25JAN2021

Subj.: Subject; IE: Subject fulfilled the inclusion and exclusion criteria; IC: Informed consent form;

Output generated by program 'NIC002_L16_2_1_1_Enrolment_V01_0_0'

Listing 16.2.1.1: Study subject data
Subject enrolment

Part B

Cohort	Subj. ID	IC Date	Screening	IE	Base-line	Day 1	Day 2	Day 3	Day 4	Follow-up
Seq. 1	48	23MAR2021	23MAR2021	Yes	28MAR2021	29MAR2021	30MAR2021	31MAR2021	01APR2021	03APR2021
	51	23MAR2021	23MAR2021	Yes	28MAR2021	29MAR2021	30MAR2021	31MAR2021	01APR2021	03APR2021
Seq. 2	49	23MAR2021	23MAR2021	Yes	28MAR2021	29MAR2021	30MAR2021	31MAR2021	01APR2021	03APR2021
	76	15APR2021	15APR2021	Yes	27APR2021	28APR2021	29APR2021	30APR2021	01MAY2021	03MAY2021

Subj.: Subject; Seq.: Sequence; IE: Subject fulfilled the inclusion and exclusion criteria; IC: Informed consent form;

Output generated by program 'NIC002_L16_2_1_1_Enrolment_V01_0_0'

Listing 16.2.1.1: Study subject data
Subject enrolment

Part C

Cohort	Subj. ID	IC Date	Screening	IE	Base-line	Day 1	Day 2	Day 3	Day 4	Day 5	Day 6	Day 7	Day 8	Follow-up
Group 1	54	01APR2021	01APR2021	Yes	12APR2021	13APR2021	14APR2021	15APR2021	16APR2021	17APR2021	18APR2021	19APR2021	20APR2021	22APR2021
	63	06APR2021	06APR2021	Yes	12APR2021	13APR2021	14APR2021	15APR2021	16APR2021	17APR2021	18APR2021	19APR2021	20APR2021	22APR2021
	67	08APR2021	08APR2021	Yes	22APR2021	23APR2021	24APR2021	25APR2021	26APR2021	27APR2021	28APR2021	29APR2021	30APR2021	03MAY2021
	71	14APR2021	14APR2021	Yes	22APR2021	23APR2021	24APR2021	25APR2021	26APR2021	27APR2021	28APR2021	29APR2021	30APR2021	03MAY2021
Group 2	57	01APR2021	01APR2021	Yes	12APR2021	13APR2021	14APR2021	15APR2021	16APR2021	17APR2021	18APR2021	19APR2021	20APR2021	22APR2021
	58	01APR2021	01APR2021	Yes	12APR2021	13APR2021	14APR2021	15APR2021	16APR2021	17APR2021	18APR2021	19APR2021	20APR2021	22APR2021
	68	14APR2021	14APR2021	Yes	22APR2021	23APR2021	24APR2021	25APR2021	26APR2021	27APR2021	28APR2021	29APR2021	30APR2021	03MAY2021
	69	14APR2021	14APR2021	Yes	22APR2021	23APR2021	24APR2021	25APR2021	26APR2021	27APR2021	28APR2021	29APR2021	30APR2021	03MAY2021
Group 3	55	01APR2021	01APR2021	Yes	12APR2021	13APR2021	14APR2021	15APR2021	16APR2021	17APR2021	18APR2021	19APR2021	20APR2021	22APR2021
	62	06APR2021	06APR2021	Yes	12APR2021	13APR2021	14APR2021	15APR2021	16APR2021	17APR2021	18APR2021	19APR2021	20APR2021	22APR2021
	65	08APR2021	08APR2021	Yes	22APR2021	23APR2021	24APR2021	25APR2021	26APR2021	27APR2021	28APR2021	29APR2021	30APR2021	03MAY2021
	66	08APR2021	08APR2021	Yes	22APR2021	23APR2021	24APR2021	25APR2021	26APR2021	27APR2021	28APR2021	29APR2021	30APR2021	03MAY2021

Subj.: Subject; IE: Subject fulfilled the inclusion and exclusion criteria; IC: Informed consent form;

Output generated by program 'NIC002_L16_2_1_1_Enrolment_V01_0_0'

Listing 16.2.1.2: Study subject data
Subject Randomization

Part A

Cohort	Subject ID	Subject randomized	Randomization date	Randomization number	Treatment allocation
Cohort A1	2	Yes	16NOV2020	111	200mg oral dose niclosamide
	5	Yes	18NOV2020	113	200mg oral dose niclosamide
	7	Yes	18NOV2020	112	Placebo
	8	Yes	18NOV2020	114	200mg oral dose niclosamide
Cohort A2	13	Yes	30NOV2020	121	600mg oral dose niclosamide
	19	Yes	02DEC2020	122	600mg oral dose niclosamide
	21	Yes	02DEC2020	124	Placebo
	22	Yes	02DEC2020	123	600mg oral dose niclosamide
Cohort A3	24	Yes	05JAN2021	131	1600mg oral dose niclosamide
	25	Yes	07JAN2021	132	1600mg oral dose niclosamide
	27	Yes	07JAN2021	133	Placebo
	28	Yes	07JAN2021	134	1600mg oral dose niclosamide

Output generated by program 'NIC002_L16_2_1_2_Randomization_V01_0_0'

Listing 16.2.1.2: Study subject data
Subject Randomization

Part B

Sequence	Subject ID	Subject randomized	Randomization date	Randomization number	Treatment allocation
Sequence 1	48	Yes	28MAR2021	211	Solution 1600 mg - Chewing tablet 2000 mg
	51	Yes	28MAR2021	212	Solution 1600 mg - Chewing tablet 2000 mg
Sequence 2	49	Yes	28MAR2021	213	Chewing tablet 2000 mg - Solution 1600 mg
	76	Yes	27APR2021	214	Chewing tablet 2000 mg - Solution 1600 mg

Output generated by program 'NIC002_L16_2_1_2_Randomization_V01_0_0'

Listing 16.2.1.2: Study subject data
Subject Randomization

Part C

Group	Subject ID	Subject randomized	Randomization date	Randomization number	Treatment allocation
Group 1	54	Yes	12APR2021	314	1200 mg niclosamide solution
	63	Yes	12APR2021	312	1200 mg niclosamide solution
	67	Yes	22APR2021	319	1200 mg niclosamide solution
	71	Yes	22APR2021	322	1200 mg niclosamide solution
Group 2	57	Yes	12APR2021	311	1600 mg niclosamide solution
	58	Yes	12APR2021	313	1600 mg niclosamide solution
	68	Yes	22APR2021	321	1600 mg niclosamide solution
	69	Yes	22APR2021	320	1600 mg niclosamide solution
Group 3	55	Yes	12APR2021	316	Placebo
	62	Yes	12APR2021	315	Placebo
	65	Yes	22APR2021	317	Placebo
	66	Yes	22APR2021	318	Placebo

Output generated by program 'NIC002_L16_2_1_2_Randomization_V01_0_0'

Listing 16.2.1.3: Study subject data
Subject Discontinuations

Part A

Cohort	Subject ID	Screening Date	Subject randomized	First treatment	Last visit	Reason for discontinuation
No subjects discontinued their study participation prematurely.						

Output generated by program 'NIC002_L16_2_1_3_Discontinuations_V01_0_0'

Listing 16.2.1.3: Study subject data
Subject Discontinuations

Part B

Sequence	Subject ID	Screening Date	Subject randomized	First treatment	Last visit	Reason for discontinuation
No subjects discontinued their study participation prematurely.						

Output generated by program 'NIC002_L16_2_1_3_Discontinuations_V01_0_0'

Listing 16.2.1.3: Study subject data
Subject Discontinuations

Part C

Group	Subject ID	Screening Date	Subject randomized	First treatment	Last visit	Reason for discontinuation
No subjects discontinued their study participation prematurely.						

Output generated by program 'NIC002_L16_2_1_3_Discontinuations_V01_0_0'

Listing 16.2.2: Subject data
Listing of Protocol deviations by Subject

Part	Cohort	Subject ID	Protocol deviation classification	Protocol deviation description	Category for protocol deviations
A	Cohort A2	13	minor	Deviation of PK sampling from the planned schedule (4hrs post dose value) Difference: 18min late	Study procedure criteria
	Cohort A3 Fed	28	minor	Deviation of PK sampling from the planned schedule (8hrs post dose value) Difference: 24 min late	Study procedure criteria
			minor	Deviation of PK sampling from the planned schedule (15hrs post dose value)Difference: 16 min late	Study procedure criteria

Output generated by program 'NIC002_L16_2_2_PDs_V01_0_0'

Listing 16.2.4: Study subject data
Subject Demographics

Part A

Cohort	Subject ID	Sex	Age [yrs]	Race	Ethnicity	Smoking history	Weight [kg]	Height [cm]	BMI [kg/m^2]
Cohort A1	2	Female	39	White/caucasian	Not hispanic or latino	Former	59	171	20
	5	Female	22	White/caucasian	Not hispanic or latino	Never	57	165	21
	8	Female	25	White/caucasian	Not hispanic or latino	Former	58	169	20
Cohort A1/Placebo	7	Female	22	White/caucasian	Not hispanic or latino	Never	59	164	22
Cohort A2	13	Female	28	White/caucasian	Not hispanic or latino	Never	56	172	19
	19	Female	30	White/caucasian	Not hispanic or latino	Never	60	171	21
	22	Female	26	White/caucasian	Not hispanic or latino	Never	81	178	26
Cohort A2/Placebo	21	Female	35	White/caucasian	Not hispanic or latino	Never	54	155	22
Cohort A3	24	Female	26	White/caucasian	Not hispanic or latino	Never	59	170	20
	25	Female	24	White/caucasian	Not hispanic or latino	Never	63	169	22
	28	Female	21	White/caucasian	Not hispanic or latino	Never	61	173	20
Cohort A3/Placebo	27	Female	36	White/caucasian	Not hispanic or latino	Former	81	178	26

Output generated by program 'NIC002_L16_2_4_Demographics_V01_0_0'

Listing 16.2.4: Study subject data
Subject Demographics

Part B

Sequence	Subject ID	Sex	Age [yrs]	Race	Ethnicity	Smoking history	Weight [kg]	Height [cm]	BMI [kg/m^2]
Sequence 1	48	Female	31	White/caucasian	Not hispanic or latino	Current	92	179	29
	51	Female	31	White/caucasian	Not hispanic or latino	Current	55	164	20
Sequence 2	49	Female	28	White/caucasian	Not hispanic or latino	Never	63	166	23
	76	Female	25	White/caucasian	Not hispanic or latino	Current	73	169	26

Output generated by program 'NIC002_L16_2_4_Demographics_V01_0_0'

Listing 16.2.4: Study subject data
Subject Demographics

Part C

Group	Subject ID	Sex	Age [yrs]	Race	Ethnicity	Smoking history	Weight [kg]	Height [cm]	BMI [kg/m^2]
Group 1	54	Female	35	White/caucasian	Not hispanic or latino	Current	83	171	28
	63	Female	31	White/caucasian	Not hispanic or latino	Former	49	159	20
	67	Female	33	White/caucasian	Not hispanic or latino	Never	50	166	18
	71	Female	22	White/caucasian	Not hispanic or latino	Never	72	171	25
Group 2	57	Female	31	White/caucasian	Not hispanic or latino	Former	79	169	28
	58	Female	42	White/caucasian	Not hispanic or latino	Never	64	160	25
	68	Female	27	White/caucasian	Not hispanic or latino	Former	75	168	26
	69	Female	35	White/caucasian	Not hispanic or latino	Former	66	162	25
Group 3	55	Female	34	White/caucasian	Not hispanic or latino	Current	57	171	20
	62	Female	39	White/caucasian	Not hispanic or latino	Never	72	163	27
	65	Female	34	White/caucasian	Not hispanic or latino	Former	65	166	24
	66	Female	24	White/caucasian	Not hispanic or latino	Never	56	159	22

Output generated by program 'NIC002_L16_2_4_Demographics_V01_0_0'

Listing 16.2.5: Study subject data
Subject Compliance

Part: A

Cohort	Subject ID	Fasting conditions Treated	Date	Dose [mg]	Fed conditions Treated	Date	Dose [mg]
Cohort A1	2	Yes	17NOV2020	200			
	5	Yes	19NOV2020	200			
	7	Yes	19NOV2020	200			
	8	Yes	19NOV2020	200			
Cohort A2	13	Yes	01DEC2020	600			
	19	Yes	03DEC2020	600			
	21	Yes	03DEC2020	600			
	22	Yes	03DEC2020	600			
Cohort A3	24	Yes	06JAN2021	1600	Yes	20JAN2021	1600
	25	Yes	08JAN2021	1600	Yes	22JAN2021	1600
	27	Yes	08JAN2021	1600	Yes	22JAN2021	1600
	28	Yes	08JAN2021	1600	Yes	22JAN2021	1600

Output generated by program 'NIC002_L16_2_5_Compliance_V01_0_0'

Listing 16.2.5: Study subject data
Subject Compliance

Part: B

Sequence	Subject ID	Day 1				Day 3			
		Treated	Date	Dose [mg]	Form	Treated	Date	Dose [mg]	Form
Sequence 1	48	Yes	29MAR2021	1600	Oral solution	Yes	31MAR2021	2000	Chewing tablet
	51	Yes	29MAR2021	1600	Oral solution	Yes	31MAR2021	2000	Chewing tablet
Sequence 2	49	Yes	29MAR2021	2000	Chewing tablet	Yes	31MAR2021	1600	Oral solution
	76	Yes	28APR2021	2000	Chewing tablet	Yes	30APR2021	1600	Oral solution

Output generated by program 'NIC002_L16_2_5_Compliance_V01_0_0'

Listing 16.2.5: Study subject data
Subject Compliance

Part: C

Group	Subj.	Day 1		Day 2		Day 3		Day 4		Day 5		Day 6		Day 7	
	ID	Trt	Date	Trt	Date	Trt	Date	Trt	Date	Trt	Date	Trt	Date	Trt	Date
Group 1	54	Yes	13APR2021	Yes	14APR2021	Yes	15APR2021	Yes	16APR2021	Yes	17APR2021	Yes	18APR2021	Yes	19APR2021
	63	Yes	13APR2021	Yes	14APR2021	Yes	15APR2021	Yes	16APR2021	Yes	17APR2021	Yes	18APR2021	Yes	19APR2021
	67	Yes	23APR2021	Yes	24APR2021	Yes	25APR2021	Yes	26APR2021	Yes	27APR2021	Yes	28APR2021	Yes	29APR2021
	71	Yes	23APR2021	Yes	23APR2021	Yes	25APR2021	Yes	26APR2021	Yes	27APR2021	Yes	28APR2021	Yes	29APR2021
Group 2	57	Yes	13APR2021	Yes	14APR2021	Yes	15APR2021	Yes	16APR2021	Yes	17APR2021	Yes	18APR2021	Yes	19APR2021
	58	Yes	13APR2021	Yes	14APR2021	Yes	15APR2021	Yes	16APR2021	Yes	17APR2021	Yes	18APR2021	Yes	19APR2021
	68	Yes	23APR2021	Yes	24APR2021	Yes	25APR2021	Yes	26APR2021	Yes	27APR2021	Yes	28APR2021	Yes	29APR2021
	69	Yes	23APR2021	Yes	24APR2021	Yes	25APR2021	Yes	26APR2021	Yes	27APR2021	Yes	28APR2021	Yes	29APR2021
Group 3	55	Yes	13APR2021	Yes	14APR2021	Yes	15APR2021	Yes	16APR2021	Yes	17APR2021	Yes	18APR2021	Yes	19APR2021
	62	Yes	13APR2021	Yes	14APR2021	Yes	15APR2021	Yes	16APR2021	Yes	17APR2021	Yes	18APR2021	Yes	19APR2021
	65	Yes	23APR2021	Yes	24APR2021	Yes	25APR2021	Yes	26APR2021	Yes	27APR2021	Yes	28APR2021	Yes	29APR2021
	66	Yes	23APR2021	Yes	24APR2021	Yes	25APR2021	Yes	26APR2021	Yes	27APR2021	Yes	28APR2021	Yes	29APR2021

Subj.: Subject; Trt: Treated;

Output generated by program 'NIC002_L16_2_5_Compliance_V01_0_0'

Listing 16.2.7: Safety data
Listing of Adverse Events by Subject

Part A

Subject ID<br>Cohort<br>Treatment	AE<br>no.	MedDRA system organ class<br>MedDRA Preferred Term<br>Investigator term	First treatment<br>Start date<br>End date	Time to<br>onset<br>[days] #<br>Duration [days] #	Severity<br>Rel. to<br>treatment	Action taken with treatment/<br>Other actions<br>Outcome	SAE/<br>Category
13<br>Cohort A2<br>600mg oral dose<br>niclosamide	1	RESPIRATORY, THORACIC AND<br>MEDIASTINAL DISORDERS<br>THROAT CLEARING<br>feeling the need to clear throat	01DEC2020<br>01DEC2020<br>01DEC2020	0<br>1	Mild<br>Related	Not applicable<br>Not applicable<br>Recovered/resolved	No
	2	RESPIRATORY, THORACIC AND<br>MEDIASTINAL DISORDERS<br>DRY THROAT<br>dry throat	01DEC2020<br>01DEC2020<br>01DEC2020	0<br>1	Mild<br>Related	Not applicable<br>Not applicable<br>Recovered/resolved	No
	3	SKIN AND SUBCUTANEOUS<br>TISSUE DISORDERS<br>ERYTHEMA<br>spotted erythema of the face	01DEC2020<br>02DEC2020<br>04DEC2020	1<br>3	Mild<br>Related	Not applicable<br>Not applicable<br>Recovered/resolved	No
19<br>Cohort A2<br>600mg oral dose<br>niclosamide	1	GASTROINTESTINAL DISORDERS<br>NAUSEA<br>nausea	03DEC2020<br>05DEC2020<br>05DEC2020	2<br>1	Mild<br>Related	Not applicable<br>Not applicable<br>Recovered/resolved	No
	2	GASTROINTESTINAL DISORDERS<br>VOMITING<br>vomiting	03DEC2020<br>05DEC2020<br>05DEC2020	2<br>1	Mild<br>Related	Not applicable<br>Not applicable<br>Recovered/resolved	No

AE: Adverse Event; no.: Number; #: Derived data; Rel.: Relationship; N/A: Not applicable; Coding based on MedDRA version 24.0; Duration = stop date – start date + 1; Time to onset = start date of AE – date of randomization;
Cohort A3: Treatment was applied under fasting and fed conditions in the same subjects;
SAE categories are defined as follows: 1: Results in death; 2: Results in serious injury; 3: A malfunction that might cause or contribute to a serious injury or death if it were to recur; 4: Requires in-patient hospitalization or prolongation of existing hospitalization; 5: Leads to fetal distress, fetal death, a congenital abnormality or birth defect; 6: Other;

Output generated by program 'NIC002_L16_2_7_AES_V02_0_0'

Listing 16.2.7: Safety data
Listing of Adverse Events by Subject

Part A

Subject ID<br>Cohort<br>Treatment	AE<br>no.	MedDRA system organ class<br>MedDRA Preferred Term<br>Investigator term	First treatment<br>Start date<br>End date	Time to onset [days] #<br>Duration [days] #	Severity<br>Rel. to treatment	Action taken with treatment/<br>Other actions<br>Outcome	SAE/<br>Category
19<br>Cohort A2<br>600mg oral dose niclosamide	3	GASTROINTESTINAL DISORDERS<br>DIARRHOEA<br>diarrhoea	03DEC2020<br>05DEC2020	2<br>Ongoing	Mild<br>Related	Not applicable<br>Not applicable<br>Recovering/resolving	No
	4	GASTROINTESTINAL DISORDERS<br>ABDOMINAL PAIN<br>intermittent abdominal cramps	03DEC2020<br>05DEC2020	2<br>Ongoing	Moderate<br>Related	Not applicable<br>Concomitant medication changed<br>Recovering/resolving	No
21<br>Cohort A2<br>Placebo	1	REPRODUCTIVE SYSTEM AND BREAST DISORDERS<br>DYSMENORRHOEA<br>lower abdominal pain (due to menstruation)	03DEC2020<br>02DEC2020<br>03DEC2020	N/A<br>2	Mild<br>Non-related	Not applicable<br>Not applicable<br>Recovered/resolved	No
	2	GASTROINTESTINAL DISORDERS<br>ORAL DISORDER<br>burning sensation in throat	03DEC2020<br>03DEC2020<br>03DEC2020	0<br>1	Mild<br>Related	Not applicable<br>Not applicable<br>Recovered/resolved	No
24<br>Cohort A3 Fast<br>1600mg oral dose niclosamide	1	GASTROINTESTINAL DISORDERS<br>ORAL DISORDER<br>feeling of irritated throat	06JAN2021<br>06JAN2021<br>06JAN2021	0<br>1	Mild<br>Related	Not applicable<br>Not applicable<br>Recovered/resolved	No

AE: Adverse Event; no.: Number; #: Derived data; Rel.: Relationship; N/A: Not applicable; Coding based on MedDRA version 24.0; Duration = stop date – start date + 1; Time to onset = start date of AE – date of randomization; Cohort A3: Treatment was applied under fasting and fed conditions in the same subjects;
SAE categories are defined as follows: 1: Results in death; 2: Results in serious injury; 3: A malfunction that might cause or contribute to a serious injury or death if it were to recur; 4: Requires in-patient hospitalization or prolongation of existing hospitalization; 5: Leads to fetal distress, fetal death, a congenital abnormality or birth defect; 6: Other;

Output generated by program 'NIC002_L16_2_7_AES_V02_0_0'

Listing 16.2.7: Safety data
Listing of Adverse Events by Subject

Part A

Subject ID<br>Cohort<br>Treatment	AE<br>no.	MedDRA system organ class<br>MedDRA Preferred Term<br>Investigator term	First treatment<br>Start date<br>End date	Time to<br>onset<br>[days] #<br>Duration [days] #	Severity<br>Rel. to<br>treatment	Action taken with treatment/<br>Other actions<br>Outcome	SAE/<br>Category
24<br>Cohort A3 Fast<br>1600mg oral dose<br>niclosamide	2	GASTROINTESTINAL DISORDERS<br>NAUSEA<br>nausea	06JAN2021<br>06JAN2021<br>06JAN2021	0<br>1	Mild<br>Related	Not applicable<br>Not applicable<br>Recovered/resolved	No
24<br>Cohort A3 Fed<br>1600mg oral dose<br>niclosamide	3	GASTROINTESTINAL DISORDERS<br>ORAL DISORDER<br>feeling of irritated throat	06JAN2021<br>20JAN2021<br>20JAN2021	14<br>1	Mild<br>Related	Not applicable<br>Not applicable<br>Recovered/resolved	No
	4	GASTROINTESTINAL DISORDERS<br>NAUSEA<br>mild nausea	06JAN2021<br>20JAN2021<br>20JAN2021	14<br>1	Mild<br>Related	Not applicable<br>Not applicable<br>Recovered/resolved	No
25<br>Cohort A3 Fast<br>1600mg oral dose<br>niclosamide	1	GASTROINTESTINAL DISORDERS<br>FAECES SOFT<br>soft stool (normal frequency)	08JAN2021<br>08JAN2021<br>09JAN2021	0<br>2	Mild<br>Related	Not applicable<br>Not applicable<br>Recovered/resolved	No
	2	GASTROINTESTINAL DISORDERS<br>NAUSEA<br>nausea	08JAN2021<br>08JAN2021<br>08JAN2021	0<br>1	Mild<br>Related	Not applicable<br>Not applicable<br>Recovered/resolved	No
	3	GASTROINTESTINAL DISORDERS<br>DYSPEPSIA<br>heartburn	08JAN2021<br>08JAN2021<br>08JAN2021	0<br>1	Mild<br>Related	Not applicable<br>Not applicable<br>Recovered/resolved	No

AE: Adverse Event; no.: Number; #: Derived data; Rel.: Relationship; N/A: Not applicable; Coding based on MedDRA version 24.0; Duration = stop date – start date + 1; Time to onset = start date of AE – date of randomization; Cohort A3: Treatment was applied under fasting and fed conditions in the same subjects;
SAE categories are defined as follows: 1: Results in death; 2: Results in serious injury; 3: A malfunction that might cause or contribute to a serious injury or death if it were to recur; 4: Requires in-patient hospitalization or prolongation of existing hospitalization; 5: Leads to fetal distress, fetal death, a congenital abnormality or birth defect; 6: Other;

Output generated by program 'NIC002_L16_2_7_AES_V02_0_0'

Listing 16.2.7: Safety data
Listing of Adverse Events by Subject

Part A

Subject ID<br>Cohort<br>Treatment	AE<br>no.	MedDRA system organ class<br>MedDRA Preferred Term<br>Investigator term	First treatment<br>Start date<br>End date	Time to<br>onset<br>[days] #<br>Duration [days] #	Severity<br>Rel. to<br>treatment	Action taken with treatment/<br>Other actions<br>Outcome	SAE/<br>Category
25<br>Cohort A3 Fed<br>1600mg oral dose<br>niclosamide	4	GASTROINTESTINAL DISORDERS<br>DIARRHOEA<br>loose stools	08JAN2021<br>22JAN2021<br>23JAN2021	14<br>2	Mild<br>Related	Not applicable<br>Not applicable<br>Recovered/resolved	No
27<br>Cohort A3 Fast<br>Placebo	1	GASTROINTESTINAL DISORDERS<br>FAECES SOFT<br>soft stool (once)	08JAN2021<br>09JAN2021<br>09JAN2021	1<br>1	Mild<br>Related	Not applicable<br>Not applicable<br>Recovered/resolved	No
28<br>Cohort A3 Fast<br>1600mg oral dose<br>niclosamide	1	GASTROINTESTINAL DISORDERS<br>DIARRHOEA<br>diarrhea	08JAN2021<br>08JAN2021<br>10JAN2021	0<br>3	Mild<br>Related	Not applicable<br>Not applicable<br>Recovered/resolved	No
	2	MUSCULOSKELETAL AND<br>CONNECTIVE TISSUE<br>DISORDERS<br>BACK PAIN<br>lower backpain (due to bed conditions)	08JAN2021<br>08JAN2021<br>08JAN2021	0<br>1	Mild<br>Non-related	Not applicable<br>Not applicable<br>Recovered/resolved	No

AE: Adverse Event; no.: Number; #: Derived data; Rel.: Relationship; N/A: Not applicable; Coding based on MedDRA version 24.0; Duration = stop date – start date + 1; Time to onset = start date of AE – date of randomization;
Cohort A3: Treatment was applied under fasting and fed conditions in the same subjects;
SAE categories are defined as follows: 1: Results in death; 2: Results in serious injury; 3: A malfunction that might cause or contribute to a serious injury or death if it were to recur; 4: Requires in-patient hospitalization or prolongation of existing hospitalization; 5: Leads to fetal distress, fetal death, a congenital abnormality or birth defect; 6: Other;

Output generated by program 'NIC002_L16_2_7_AES_V02_0_0'

Listing 16.2.7: Safety data
Listing of Adverse Events by Subject

Part: B

Subject ID<br>Treatment	AE<br>no.	MedDRA system organ class<br>MedDRA Preferred Term<br>Investigator term	Start date<br>End date	Time to<br>onset<br>[days] #<br>Duration [days] #	Severity<br>Rel. to<br>treatment	Action taken with treatment/<br>Other actions<br>Outcome	SAE/<br>Category
49<br>Solution 1600 mg	1	GASTROINTESTINAL DISORDERS<br>NAUSEA<br>nausea	29MAR2021<br>31MAR2021<br>31MAR2021	2<br>1	Mild<br>Related	Not applicable<br>Not applicable<br>Recovered/resolved	No
76<br>Solution 1600 mg	1	GASTROINTESTINAL DISORDERS<br>DIARRHOEA<br>loose stool	28APR2021<br>30APR2021<br>30APR2021	2<br>1	Mild<br>Related	Not applicable<br>Not applicable<br>Recovered/resolved	No
	2	GASTROINTESTINAL DISORDERS<br>VOMITING<br>vomitting	28APR2021<br>30APR2021<br>30APR2021	2<br>1	Mild<br>Related	Not applicable<br>Not applicable<br>Recovered/resolved	No

AE: Adverse Event; no.: Number; #: Derived data; Rel.: Relationship; N/A: Not applicable; Coding based on MedDRA version 24.0; Duration = stop date – start date + 1; Time to onset = start date of AE – date of randomization; Part B used a cross-over design;
SAE categories are defined as follows: 1: Results in death; 2: Results in serious injury; 3: A malfunction that might cause or contribute to a serious injury or death if it were to recur; 4: Requires in-patient hospitalization or prolongation of existing hospitalization; 5: Leads to fetal distress, fetal death, a congenital abnormality or birth defect; 6: Other;

Output generated by program 'NIC002_L16_2_7_AES_V02_0_0'

Listing 16.2.7: Safety data
Listing of Adverse Events by Subject

Part: C

Subject ID<br>Group<br>Treatment	AE<br>no.	MedDRA system organ class<br>MedDRA Preferred Term<br>Investigator term	Start date<br>End date	Time to<br>onset<br>[days] #<br>Duration [days] #	Severity<br>Rel. to<br>treatment	Action taken with treatment/<br>Other actions<br>Outcome	SAE/<br>Category
54<br>Group 1<br>1200 mg niclosamide<br>solution	3	GASTROINTESTINAL DISORDERS<br>DIARRHOEA<br>intermittent diarrhea	13APR2021<br>13APR2021<br>22APR2021	0<br>10	Mild<br>Related	dose not changed<br>Not applicable<br>Recovered/resolved	No
	4	GASTROINTESTINAL DISORDERS<br>DRY MOUTH<br>intermittent dry mouth	13APR2021<br>13APR2021<br>19APR2021	0<br>7	Mild<br>Related	dose not changed<br>Not applicable<br>Recovered/resolved	No
	5	GASTROINTESTINAL DISORDERS<br>RECTAL HAEMORRHAGE<br>rectal bleeding after stool	13APR2021<br>18APR2021<br>19APR2021	5<br>2	Mild<br>Related	dose not changed<br>Not applicable<br>Recovered/resolved	No
55<br>Group 3<br>Placebo	1	GASTROINTESTINAL DISORDERS<br>FLATULENCE<br>flatulence	13APR2021<br>13APR2021<br>21APR2021	0<br>9	Mild<br>Related	dose not changed<br>Not applicable<br>Recovered/resolved	No
	2	GASTROINTESTINAL DISORDERS<br>FAECES SOFT<br>soft stool	13APR2021<br>15APR2021	2<br>Ongoing	Mild<br>Related	dose not changed<br>Not applicable<br>Not recovered/not resolved	No
57<br>Group 2<br>1600 mg niclosamide<br>solution	2	INFECTIONS AND INFESTATIONS<br>RHINITIS<br>rhinitis	13APR2021<br>14APR2021<br>15APR2021	1<br>2	Mild<br>Non-related	dose not changed<br>Not applicable<br>Recovered/resolved	No

AE: Adverse Event; no.: Number; #: Derived data; Rel.: Relationship; N/A: Not applicable; Coding based on MedDRA version 24.0; Duration = stop date – start date + 1; Time to onset = start date of AE – date of randomization
SAE categories are defined as follows: 1: Results in death; 2: Results in serious injury; 3: A malfunction that might cause or contribute to a serious injury or death if it were to recur; 4: Requires in-patient hospitalization or prolongation of existing hospitalization; 5: Leads to fetal distress, fetal death, a congenital abnormality or birth defect; 6: Other;

Output generated by program 'NIC002_L16_2_7_AES_V02_0_0'

Listing 16.2.7: Safety data
Listing of Adverse Events by Subject

Part: C

Subject ID<br>Group<br>Treatment	AE<br>no.	MedDRA system organ class<br>MedDRA Preferred Term<br>Investigator term	Start date<br>End date	Time to<br>onset<br>[days] #<br>Duration [days] #	Severity<br>Rel. to<br>treatment	Action taken with treatment/<br>Other actions<br>Outcome	SAE/<br>Category
57<br>Group 2<br>1600 mg niclosamide<br>solution	3	NERVOUS SYSTEM DISORDERS<br>HEADACHE<br>headache	13APR2021<br>13APR2021<br>13APR2021	0<br>1	Mild<br>Related	dose not changed<br>Not applicable<br>Recovered/resolved	No
	4	GASTROINTESTINAL DISORDERS<br>DIARRHOEA<br>diarrhea	13APR2021<br>14APR2021<br>19APR2021	1<br>6	Mild<br>Related	dose not changed<br>Not applicable<br>Recovered/resolved	No
	5	GASTROINTESTINAL DISORDERS<br>NAUSEA<br>nausea	13APR2021<br>18APR2021<br>18APR2021	5<br>1	Mild<br>Related	dose not changed<br>Not applicable<br>Recovered/resolved	No
	6	NERVOUS SYSTEM DISORDERS<br>HEADACHE<br>headache	13APR2021<br>21APR2021<br>21APR2021	8<br>1	Mild<br>Non-related	Not applicable<br>Not applicable<br>Recovered/resolved	No
58<br>Group 2<br>1600 mg niclosamide<br>solution	1	GASTROINTESTINAL DISORDERS<br>VOMITING<br>vomiting (once) (due to according to subject too much food)	13APR2021<br>14APR2021<br>14APR2021	1<br>1	Mild<br>Related	dose not changed<br>Not applicable<br>Recovered/resolved	No
	2	GASTROINTESTINAL DISORDERS<br>NAUSEA<br>nausea (according to subject due to food)	13APR2021<br>14APR2021<br>14APR2021	1<br>1	Moderate<br>Related	dose not changed<br>Not applicable<br>Recovered/resolved	No
	3	GASTROINTESTINAL DISORDERS<br>DIARRHOEA<br>loose stool (3 times)	13APR2021<br>13APR2021<br>13APR2021	0<br>1	Mild<br>Related	dose not changed<br>Not applicable<br>Recovered/resolved	No

AE: Adverse Event; no.: Number; #: Derived data; Rel.: Relationship; N/A: Not applicable; Coding based on MedDRA version 24.0; Duration = stop date – start date + 1; Time to onset = start date of AE – date of randomization
SAE categories are defined as follows: 1: Results in death; 2: Results in serious injury; 3: A malfunction that might cause or contribute to a serious injury or death if it were to recur; 4: Requires in-patient hospitalization or prolongation of existing hospitalization; 5: Leads to fetal distress, fetal death, a congenital abnormality or birth defect; 6: Other;

Output generated by program 'NIC002_L16_2_7_AES_V02_0_0'

Listing 16.2.7: Safety data
Listing of Adverse Events by Subject

Part: C

Subject ID<br>Group<br>Treatment	AE<br>no.	MedDRA system organ class<br>MedDRA Preferred Term<br>Investigator term	Start date<br>End date	Time to onset [days] #<br>Duration [days] #	Severity<br>Rel. to treatment	Action taken with treatment/<br>Other actions<br>Outcome	SAE/<br>Category
58<br>Group 2<br>1600 mg niclosamide solution	4	GASTROINTESTINAL DISORDERS<br>DIARRHOEA<br>loose stool	13APR2021<br>17APR2021<br>19APR2021	4<br>3	Mild<br>Related	dose not changed<br>Not applicable<br>Recovered/resolved	No
62<br>Group 3<br>Placebo	1	GASTROINTESTINAL DISORDERS<br>DIARRHOEA<br>intermittent loose stool	13APR2021<br>15APR2021<br>21APR2021	2<br>7	Mild<br>Related	dose not changed<br>Not applicable<br>Recovered/resolved	No
	2	NERVOUS SYSTEM DISORDERS<br>HEADACHE<br>headache	13APR2021<br>13APR2021<br>13APR2021	0<br>1	Mild<br>Non-related	dose not changed<br>Not applicable<br>Recovered/resolved	No
	3	GASTROINTESTINAL DISORDERS<br>FLATULENCE<br>flatulence	13APR2021<br>15APR2021<br>22APR2021	2<br>8	Mild<br>Related	dose not changed<br>Not applicable<br>Recovered/resolved	No
63<br>Group 1<br>1200 mg niclosamide solution	1	GASTROINTESTINAL DISORDERS<br>NAUSEA<br>intermittent nausea (according to subject due to breakfast)	13APR2021<br>14APR2021<br>19APR2021	1<br>6	Mild<br>Related	dose not changed<br>Not applicable<br>Recovered/resolved	No
	2	RESPIRATORY, THORACIC AND MEDIASTINAL DISORDERS<br>COUGH<br>coughing	13APR2021<br>14APR2021<br>15APR2021	1<br>2	Mild<br>Related	dose not changed<br>Not applicable<br>Recovered/resolved	No
	3	NERVOUS SYSTEM DISORDERS<br>HEADACHE<br>headache	13APR2021<br>13APR2021<br>13APR2021	0<br>1	Mild<br>Related	dose not changed<br>Not applicable<br>Recovered/resolved	No

AE: Adverse Event; no.: Number; #: Derived data; Rel.: Relationship; N/A: Not applicable; Coding based on MedDRA version 24.0; Duration = stop date – start date + 1; Time to onset = start date of AE – date of randomization
SAE categories are defined as follows: 1: Results in death; 2: Results in serious injury; 3: A malfunction that might cause or contribute to a serious injury or death if it were to recur; 4: Requires in-patient hospitalization or prolongation of existing hospitalization; 5: Leads to fetal distress, fetal death, a congenital abnormality or birth defect; 6: Other;

Output generated by program 'NIC002_L16_2_7_AES_V02_0_0'

Listing 16.2.7: Safety data
Listing of Adverse Events by Subject

Part: C

Subject ID<br>Group<br>Treatment	AE<br>no.	MedDRA system organ class<br>MedDRA Preferred Term<br>Investigator term	Start date<br>End date	Time to<br>onset<br>[days] #<br>Duration [days] #	Severity<br>Rel. to<br>treatment	Action taken with treatment/<br>Other actions<br>Outcome	SAE/<br>Category
63<br>Group 1<br>1200 mg niclosamide<br>solution	4	GASTROINTESTINAL DISORDERS<br>DIARRHOEA<br>diarrhea	13APR2021<br>15APR2021<br>21APR2021	2<br>7	Mild<br>Related	dose not changed<br>Not applicable<br>Recovered/resolved	No
	5	GASTROINTESTINAL DISORDERS<br>FLATULENCE<br>flatulences	13APR2021<br>16APR2021<br>18APR2021	3<br>3	Mild<br>Related	dose not changed<br>Not applicable<br>Recovered/resolved	No
	6	GENERAL DISORDERS AND<br>ADMINISTRATION SITE<br>CONDITIONS<br>PUNCTURE SITE PAIN<br>pain right arm after blood draw	13APR2021<br>19APR2021	6<br>Ongoing	Mild<br>Non-related	dose not changed<br>Not applicable<br>Recovering/resolving	No
65<br>Group 3<br>Placebo	2	GASTROINTESTINAL DISORDERS<br>DIARRHOEA<br>intermittent diarrhea	23APR2021<br>24APR2021<br>29APR2021	1<br>6	Moderate<br>Related	dose not changed<br>Not applicable<br>Recovered/resolved	No
	3	GENERAL DISORDERS AND<br>ADMINISTRATION SITE<br>CONDITIONS<br>FATIGUE<br>tiredness	23APR2021<br>28APR2021<br>29APR2021	5<br>2	Mild<br>Related	dose not changed<br>Not applicable<br>Recovered/resolved	No
66<br>Group 3<br>Placebo	1	GASTROINTESTINAL DISORDERS<br>NAUSEA<br>nausea	23APR2021<br>23APR2021<br>23APR2021	0<br>1	Mild<br>Non-related	dose not changed<br>Not applicable<br>Recovered/resolved	No
	2	GASTROINTESTINAL DISORDERS<br>DIARRHOEA<br>intermittent loose stool	23APR2021<br>24APR2021<br>02MAY2021	1<br>9	Mild<br>Related	dose not changed<br>Not applicable<br>Recovered/resolved	No

AE: Adverse Event; no.: Number; #: Derived data; Rel.: Relationship; N/A: Not applicable; Coding based on MedDRA version 24.0; Duration = stop date – start date + 1; Time to onset = start date of AE – date of randomization
SAE categories are defined as follows: 1: Results in death; 2: Results in serious injury; 3: A malfunction that might cause or contribute to a serious injury or death if it were to recur; 4: Requires in-patient hospitalization or prolongation of existing hospitalization; 5: Leads to fetal distress, fetal death, a congenital abnormality or birth defect; 6: Other;

Output generated by program 'NIC002_L16_2_7_AES_V02_0_0'

Listing 16.2.7: Safety data
Listing of Adverse Events by Subject

Part: C

Subject ID<br>Group<br>Treatment	AE<br>no.	MedDRA system organ class<br>MedDRA Preferred Term<br>Investigator term	Start date<br>End date	Time to<br>onset<br>[days] #<br>Duration [days] #	Severity<br>Rel. to<br>treatment	Action taken with treatment/<br>Other actions<br>Outcome	SAE/<br>Category
67<br>Group 1<br>1200 mg niclosamide<br>solution	1	GASTROINTESTINAL DISORDERS<br>DIARRHOEA<br>intermittent loose stool (once - twice per day)	23APR2021<br>25APR2021<br>30APR2021	2<br>6	Mild<br>Related	dose not changed<br>Not applicable<br>Recovered/resolved	No
	2	NERVOUS SYSTEM DISORDERS<br>HEADACHE<br>intermittent headache after dosing	23APR2021<br>26APR2021<br>29APR2021	3<br>4	Mild<br>Related	dose not changed<br>Not applicable<br>Recovered/resolved	No
68<br>Group 2<br>1600 mg niclosamide<br>solution	1	GASTROINTESTINAL DISORDERS<br>NAUSEA<br>nausea	23APR2021<br>25APR2021<br>27APR2021	2<br>3	Mild<br>Related	dose not changed<br>Not applicable<br>Recovered/resolved	No
	2	GASTROINTESTINAL DISORDERS<br>DIARRHOEA<br>intermittent loose stool	23APR2021<br>24APR2021<br>29APR2021	1<br>6	Mild<br>Related	dose not changed<br>Not applicable<br>Recovered/resolved	No
69<br>Group 2<br>1600 mg niclosamide<br>solution	3	GASTROINTESTINAL DISORDERS<br>VOMITING<br>episode of vomiting	23APR2021<br>24APR2021<br>24APR2021	1<br>1	Mild<br>Non-related	dose not changed<br>Not applicable<br>Recovered/resolved	No
	4	GASTROINTESTINAL DISORDERS<br>NAUSEA<br>intermittent nausea after breakfast	23APR2021<br>24APR2021<br>29APR2021	1<br>6	Mild<br>Non-related	dose not changed<br>Not applicable<br>Recovered/resolved	No

AE: Adverse Event; no.: Number; #: Derived data; Rel.: Relationship; N/A: Not applicable; Coding based on MedDRA version 24.0; Duration = stop date – start date + 1; Time to onset = start date of AE – date of randomization
SAE categories are defined as follows: 1: Results in death; 2: Results in serious injury; 3: A malfunction that might cause or contribute to a serious injury or death if it were to recur; 4: Requires in-patient hospitalization or prolongation of existing hospitalization; 5: Leads to fetal distress, fetal death, a congenital abnormality or birth defect; 6: Other;

Output generated by program 'NIC002_L16_2_7_AES_V02_0_0'

Listing 16.2.7: Safety data
Listing of Adverse Events by Subject

Part: C

Subject ID<br>Group<br>Treatment	AE<br>no.	MedDRA system organ class<br>MedDRA Preferred Term<br>Investigator term	Start date<br>End date	Time to onset [days] #<br>Duration [days] #	Severity<br>Rel. to treatment	Action taken with treatment/<br>Other actions<br>Outcome	SAE/<br>Category
69<br>Group 2<br>1600 mg niclosamide solution	5	GASTROINTESTINAL DISORDERS<br>DIARRHOEA<br>intermittent loose stool	23APR2021<br>23APR2021<br>29APR2021	0<br>7	Mild<br>Related	dose not changed<br>Not applicable<br>Recovered/resolved	No
71<br>Group 1<br>1200 mg niclosamide solution	1	GASTROINTESTINAL DISORDERS<br>NAUSEA<br>nausea after breakfast	23APR2021<br>27APR2021<br>27APR2021	4<br>1	Mild<br>Non-related	dose not changed<br>Not applicable<br>Recovered/resolved	No
	3	GASTROINTESTINAL DISORDERS<br>DIARRHOEA<br>intermittent diarrhea (6 times)	23APR2021<br>27APR2021<br>28APR2021	4<br>2	Moderate<br>Related	dose not changed<br>Not applicable<br>Recovered/resolved	No

AE: Adverse Event; no.: Number; #: Derived data; Rel.: Relationship; N/A: Not applicable; Coding based on MedDRA version 24.0; Duration = stop date – start date + 1; Time to onset = start date of AE – date of randomization
SAE categories are defined as follows: 1: Results in death; 2: Results in serious injury; 3: A malfunction that might cause or contribute to a serious injury or death if it were to recur; 4: Requires in-patient hospitalization or prolongation of existing hospitalization; 5: Leads to fetal distress, fetal death, a congenital abnormality or birth defect; 6: Other;

Output generated by program 'NIC002_L16_2_7_AES_V02_0_0'

Listing 16.2.8: Study subject data
Laboratory values

Part A

Cohort	Subject ID	Visit	Type	Measurement	Result		Flagged	Sign.
Cohort A1	2	SC	Clinical Chemistry	ALT, 37 °C	7.0	U/L		
				Alkaline Phosphatase, 37 °C	45.9	U/L		
				AST, 37 °C	15.5	U/L		
				Bicarbonate	25.7	mmol/L		
				Bilirubin, total	8.4	umol/L		
				Urea/BUN	3.69	mmol/L		
				Calcium	2.12	mmol/L	L	No
				Creatinine	55.0	umol/L		
				Glucose, serum	5.14	mmol/L		
				Gamma-GT, 37 °C	7.0	U/L		
				Potassium	3.78	mmol/L		
				Creatinine Clearance MDRD	107	ml/min/1.73m		
				Magnesium	0.81	mmol/L		
				Sodium	137.6	mmol/L		
			Drugs	Amphetamines, Urine	negative			
				Barbiturates, Urine	negative			
				Benzodiazepines, Urine	negative			
				Cannabin., Urine	negative			
				Cocaine, Urine	negative			
				Methadone, Urine	negative			
				Opiates, Urine	negative			
			Haematology	Basophils, abs.	0.06	10^9/L		
				Basophils, %	1.0	%		
				Eosinophils, abs.	0.30	10^9/L		
				Eosinophils, %	5.0	%		
				Haemoglobin	118.0	g/L		
				Haematocrit	0.35	L/L		
				Lymphocytes, abs.	1.88	10^9/L		
				Lymphocytes, %	31.4	%		
				Monocytes, abs.	0.53	10^9/L		

Sign.: Significant finding; L: Result considered low; H: Result considered high; SC: Screening; BL: Baseline; D: Day; FUP: Follow-up; Cohort A3: Treatment was applied under fasting and fed conditions in the same subjects;

Output generated by program 'NIC002_L16_2_8_Laboratory_V02_0_0'

Listing 16.2.8: Study subject data
Laboratory values

Part A

Cohort	Subject ID	Visit	Type	Measurement	Result		Flagged	Sign.
Cohort A1	2	SC	Haematology	Monocytes, %	8.9	%		
				Neutrophils, abs.	3.21	10^9/L		
				Neutrophils, %	53.7	%		
				Platelets	178	10^9/L		
				Erythrocytes	3.66	10^{12}/L	L	No
				Leucocytes	5.98	10^9/L		
			Haemostasis	APTT	27.7	s		
				Prothrombin Time (INR)	1.03	N/A		
				Prothrombin Time (PT)	96.3	%		
			Infectious Diseases	HBs-Ag (Hep. B Surf. Ag)	negative	N/A		
				Anti-HCV (Hep. C-AB)	non-reactive	N/A		
				HIV 1+2, AG/AB	negative	N/A		
			Urine	Bilirubin, urine (Stix)	negative			
				Blood (Ery/Hb), urine (Stix)	3+		H	No
				Glucose, urine (Stix)	negative			
				Beta-HCG, urine	negative			
				Ketone, urine (Stix)	negative			
				Leucocytes, urine (Stix)	negative			
				Nitrite, urine (Stix)	negative			
				pH, urine (Stix)	6.0	neg.log[H+]		
				Protein, total, urine (Stix)	negative			
				Bacteria, Sediment	positive		H	No
				Carbonate, Sediment	negative			
				Epithelial Cells, Sediment	4	per field	L	No
				Erythrocytes, Sediment	3	per field	H	No
				Casts granul., Sediment	0	per field		
				Casts hyaline, Sediment	0	per field		
				Leucocytes, Sediment	2	per field		
				Oxalate, Sediment	negative			
				Specific Gravity	1.020			
				Mucus, Sediment	positive		H	No

Sign.: Significant finding; L: Result considered low; H: Result considered high; SC: Screening; BL: Baseline; D: Day; FUP: Follow-up; Cohort A3: Treatment was applied under fasting and fed conditions in the same subjects;

Output generated by program 'NIC002_L16_2_8_Laboratory_V02_0_0'

Listing 16.2.8: Study subject data
Laboratory values

Part A

Cohort	Subject ID	Visit	Type	Measurement	Result		Flagged	Sign.
Cohort A1	2	SC	Urine	Triple Phosphate, Sediment	negative			
				Urates, Sediment	negative			
				Urobilinogen, urine (Stix)	0.2	mg/dl		
		BL	Clinical Chemistry	ALT, 37 °C	8.9	U/L		
				Alkaline Phosphatase, 37 °C	47.6	U/L		
				AST, 37 °C	15.9	U/L		
				Bicarbonate	27.1	mmol/L		
				Bilirubin, total	10.8	umol/L		
				Urea/BUN	2.58	mmol/L	L	No
				Calcium	2.21	mmol/L		
				Creatinine	49.5	umol/L		
				Glucose, serum	5.10	mmol/L		
				Gamma-GT, 37 °C	7.3	U/L		
				Potassium	3.84	mmol/L		
				Magnesium	0.80	mmol/L		
				Sodium	138.6	mmol/L		
			Drugs	Amphetamines, Urine	negative			
				Barbiturates, Urine	negative			
				Benzodiazepines, Urine	negative			
				Cannabin., Urine	negative			
				Cocaine, Urine	negative			
				Methadone, Urine	negative			
				Opiates, Urine	negative			
			Haematology	Basophils, abs.	0.05	10^9/L		
				Basophils, %	1.1	%		
				Eosinophils, abs.	0.27	10^9/L		
				Eosinophils, %	6.1	%		
				Haemoglobin	117.0	g/L		
				Haematocrit	0.34	L/L	L	No
				Lymphocytes, abs.	1.73	10^9/L		

Sign.: Significant finding; L: Result considered low; H: Result considered high; SC: Screening; BL: Baseline; D: Day; FUP: Follow-up; Cohort A3: Treatment was applied under fasting and fed conditions in the same subjects;

Output generated by program 'NIC002_L16_2_8_Laboratory_V02_0_0'

Listing 16.2.8: Study subject data
Laboratory values

Part A

Cohort	Subject ID	Visit	Type	Measurement	Result		Flagged	Sign.
		BL	Haematology	Lymphocytes, %	38.9	%		
				Monocytes, abs.	0.39	10^9/L		
				Monocytes, %	8.8	%		
				Neutrophils, abs.	2.01	10^9/L		
				Neutrophils, %	45.1	%		
				Platelets	184	10^9/L		
				Erythrocytes	3.60	10^{12}/L	L	No
				Leucocytes	4.45	10^9/L		
			Haemostasis	APTT	27.6	s		
				Prothrombin Time (INR)	1.05	N/A		
				Prothrombin Time (PT)	91.5	%		
			Urine	Beta-HCG, urine	negative			
		D02	Clinical Chemistry	ALT, 37 °C	11.1	U/L		
				Alkaline Phosphatase, 37 °C	45.8	U/L		
				AST, 37 °C	21.2	U/L		
				Bicarbonate	24.3	mmol/L		
				Bilirubin, total	8.0	umol/L		
				Urea/BUN	3.97	mmol/L		
				Calcium	2.18	mmol/L	L	No
				Creatinine	53.7	umol/L		
				Glucose, serum	4.88	mmol/L		
				Gamma-GT, 37 °C	6.2	U/L		
				Potassium	3.72	mmol/L		
				Magnesium	0.77	mmol/L		
				Sodium	138.1	mmol/L		
			Haematology	Basophils, abs.	0.07	10^9/L		
				Basophils, %	1.3	%		
				Eosinophils, abs.	0.15	10^9/L		
				Eosinophils, %	2.9	%		
				Haemoglobin	115.0	g/L		

Sign.: Significant finding; L: Result considered low; H: Result considered high; SC: Screening; BL: Baseline; D: Day; FUP: Follow-up; Cohort A3: Treatment was applied under fasting and fed conditions in the same subjects;

Output generated by program 'NIC002_L16_2_8_Laboratory_V02_0_0'

Listing 16.2.8: Study subject data
Laboratory values

Part A

Cohort	Subject ID	Visit	Type	Measurement	Result		Flagged	Sign.
		D02	Haematology	Haematocrit	0.33	L/L	L	No
				Lymphocytes, abs.	1.84	10^9/L		
				Lymphocytes, %	35.1	%		
				Monocytes, abs.	0.38	10^9/L		
				Monocytes, %	7.3	%		
				Neutrophils, abs.	2.80	10^9/L		
				Neutrophils, %	53.4	%		
				Platelets	183	10^9/L		
				Erythrocytes	3.53	10^{12}/L	L	No
				Leucocytes	5.24	10^9/L		
			Haemostasis	APTT	27.7	s		
				Prothrombin Time (INR)	1.05	N/A		
				Prothrombin Time (PT)	91.5	%		
		FUP	Clinical Chemistry	ALT, 37 °C	11.9	U/L		
				Alkaline Phosphatase, 37 °C	49.3	U/L		
				AST, 37 °C	18.7	U/L		
				Bicarbonate	26.9	mmol/L		
				Bilirubin, total	7.7	umol/L		
				Urea/BUN	3.32	mmol/L		
				Calcium	2.19	mmol/L	L	No
				Creatinine	50.2	umol/L		
				Glucose, serum	4.66	mmol/L		
				Gamma-GT, 37 °C	7.3	U/L		
				Potassium	4.35	mmol/L		
				Magnesium	0.79	mmol/L		
				Sodium	137.5	mmol/L		
			Haematology	Basophils, abs.	0.04	10^9/L		
				Basophils, %	1.0	%		
				Eosinophils, abs.	0.17	10^9/L		
				Eosinophils, %	4.1	%		

Sign.: Significant finding; L: Result considered low; H: Result considered high; SC: Screening; BL: Baseline; D: Day; FUP: Follow-up; Cohort A3: Treatment was applied under fasting and fed conditions in the same subjects;

Output generated by program 'NIC002_L16_2_8_Laboratory_V02_0_0'

Listing 16.2.8: Study subject data
Laboratory values

Part A

Cohort	Subject ID	Visit	Type	Measurement	Result		Flagged	Sign.
		FUP	Haematology	Haemoglobin	111.0	g/L		
				Haematocrit	0.33	L/L	L	No
				Lymphocytes, abs.	1.32	10^9/L		
				Lymphocytes, %	32.1	%		
				Monocytes, abs.	0.29	10^9/L		
				Monocytes, %	7.1	%		
				Neutrophils, abs.	2.29	10^9/L		
				Neutrophils, %	55.7	%		
				Platelets	177	10^9/L		
				Erythrocytes	3.43	10^{12}/L	L	No
				Leucocytes	4.11	10^9/L		
			Haemostasis	APTT	27.3	s		
				Prothrombin Time (INR)	1.04	N/A		
				Prothrombin Time (PT)	93.9	%		
			Urine	Beta-HCG, urine	negative			
	5	SC	Clinical Chemistry	ALT, 37 °C	11.2	U/L		
				Alkaline Phosphatase, 37 °C	32.0	U/L		
				AST, 37 °C	24.0	U/L		
				Bicarbonate	24.2	mmol/L		
				Bilirubin, total	6.7	umol/L		
				Urea/BUN	3.74	mmol/L		
				Calcium	2.36	mmol/L		
				Creatinine	54.4	umol/L		
				Glucose, serum	4.57	mmol/L		
				Gamma-GT, 37 °C	13.8	U/L		
				Potassium	3.69	mmol/L		
				Creatinine Clearance MDRD	120	ml/min/1.73m		
				Magnesium	0.83	mmol/L		
				Sodium	137.8	mmol/L		
			Drugs	Amphetamines, Urine	negative			

Sign.: Significant finding; L: Result considered low; H: Result considered high; SC: Screening; BL: Baseline; D: Day; FUP: Follow-up; Cohort A3: Treatment was applied under fasting and fed conditions in the same subjects;

Output generated by program 'NIC002_L16_2_8_Laboratory_V02_0_0'

Listing 16.2.8: Study subject data
Laboratory values

Part A

Cohort	Subject ID	Visit	Type	Measurement	Result		Flagged	Sign.
	5	SC	Drugs	Barbiturates, Urine	negative			
				Benzodiazepines, Urine	negative			
				Cannabin., Urine	negative			
				Cocaine, Urine	negative			
				Methadone, Urine	negative			
				Opiates, Urine	negative			
			Haematology	Basophils, abs.	0.06	10^9/L		
				Basophils, %	1.3	%		
				Eosinophils, abs.	0.12	10^9/L		
				Eosinophils, %	2.5	%		
				Haemoglobin	116.0	g/L		
				Haematocrit	0.35	L/L		
				Lymphocytes, abs.	1.77	10^9/L		
				Lymphocytes, %	37.4	%		
				Monocytes, abs.	0.39	10^9/L		
				Monocytes, %	8.2	%		
				Neutrophils, abs.	2.39	10^9/L		
				Neutrophils, %	50.6	%		
				Platelets	263	10^9/L		
				Erythrocytes	3.85	10^{12}/L	L	No
				Leucocytes	4.73	10^9/L		
			Haemostasis	APTT	25.7	s		
				Prothrombin Time (INR)	1.14	N/A		
				Prothrombin Time (PT)	73.5	%		
			Infectious Diseases	HBs-Ag (Hep. B Surf. Ag)	negative	N/A		
				Anti-HCV (Hep. C-AB)	non-reactive	N/A		
				HIV 1+2, AG/AB	negative	N/A		
			Urine	Bilirubin, urine (Stix)	negative			
				Blood (Ery/Hb), urine (Stix)	negative			
				Glucose, urine (Stix)	negative			
				Beta-HCG, urine	negative			

Sign.: Significant finding; L: Result considered low; H: Result considered high; SC: Screening; BL: Baseline; D: Day; FUP: Follow-up; Cohort A3: Treatment was applied under fasting and fed conditions in the same subjects;

Output generated by program 'NIC002_L16_2_8_Laboratory_V02_0_0'

Listing 16.2.8: Study subject data
Laboratory values

Part A

Cohort	Subject ID	Visit	Type	Measurement	Result		Flagged	Sign.
	5	SC	Urine	Ketone, urine (Stix)	negative			
				Leucocytes, urine (Stix)	negative			
				Nitrite, urine (Stix)	negative			
				pH, urine (Stix)	7.0	neg.log[H+]		
				Protein, total, urine (Stix)	negative			
				Bacteria, Sediment	negative			
				Carbonate, Sediment	negative			
				Epithelial Cells, Sediment	3	per field	L	No
				Erythrocytes, Sediment	0	per field		
				Casts granul., Sediment	0	per field		
				Casts hyaline, Sediment	0	per field		
				Leucocytes, Sediment	1	per field		
				Oxalate, Sediment	negative			
				Specific Gravity	<=1.005			
				Mucus, Sediment	negative			
				Triple Phosphate, Sediment	negative			
				Urates, Sediment	negative			
				Urobilinogen, urine (Stix)	0.2	mg/dl		
		BL	Clinical Chemistry	ALT, 37 °C	13.7	U/L		
				Alkaline Phosphatase, 37 °C	33.3	U/L		
				AST, 37 °C	22.8	U/L		
				Bicarbonate	25.1	mmol/L		
				Bilirubin, total	7.0	umol/L		
				Urea/BUN	3.68	mmol/L		
				Calcium	2.39	mmol/L		
				Creatinine	50.0	umol/L		
				Glucose, serum	5.03	mmol/L		
				Gamma-GT, 37 °C	13.2	U/L		
				Potassium	3.87	mmol/L		
				Magnesium	0.80	mmol/L		

Sign.: Significant finding; L: Result considered low; H: Result considered high; SC: Screening; BL: Baseline; D: Day; FUP: Follow-up; Cohort A3: Treatment was applied under fasting and fed conditions in the same subjects;

Output generated by program 'NIC002_L16_2_8_Laboratory_V02_0_0'

Listing 16.2.8: Study subject data
Laboratory values

Part A

Cohort	Subject ID	Visit	Type	Measurement	Result		Flagged	Sign.
		BL	Clinical Chemistry	Sodium	140.5	mmol/L		
			Drugs	Amphetamines, Urine	negative			
				Barbiturates, Urine	negative			
				Benzodiazepines, Urine	negative			
				Cannabin., Urine	negative			
				Cocaine, Urine	negative			
				Methadone, Urine	negative			
				Opiates, Urine	negative			
			Haematology	Basophils, abs.	0.05	10^9/L		
				Basophils, %	1.1	%		
				Eosinophils, abs.	0.10	10^9/L		
				Eosinophils, %	2.3	%		
				Haemoglobin	113.0	g/L		
				Haematocrit	0.34	L/L	L	No
				Lymphocytes, abs.	1.74	10^9/L		
				Lymphocytes, %	39.9	%		
				Monocytes, abs.	0.31	10^9/L		
				Monocytes, %	7.1	%		
				Neutrophils, abs.	2.16	10^9/L		
				Neutrophils, %	49.6	%		
				Platelets	265	10^9/L		
				Erythrocytes	3.74	10^{12}/L	L	No
				Leucocytes	4.36	10^9/L		
			Haemostasis	APTT	26.1	s		
				Prothrombin Time (INR)	1.20	N/A	H	No
				Prothrombin Time (PT)	66.0	%	L	No
			Urine	Beta-HCG, urine	negative			
		D02	Clinical Chemistry	ALT, 37 °C	12.3	U/L		
				Alkaline Phosphatase, 37 °C	34.3	U/L		
				AST, 37 °C	21.0	U/L		

Sign.: Significant finding; L: Result considered low; H: Result considered high; SC: Screening; BL: Baseline; D: Day; FUP: Follow-up; Cohort A3: Treatment was applied under fasting and fed conditions in the same subjects;

Output generated by program 'NIC002_L16_2_8_Laboratory_V02_0_0'

Listing 16.2.8: Study subject data
Laboratory values

Part A

Cohort	Subject ID	Visit	Type	Measurement	Result		Flagged	Sign.
		D02	Clinical Chemistry	Bicarbonate	25.2	mmol/L		
				Bilirubin, total	11.1	umol/L		
				Urea/BUN	3.92	mmol/L		
				Calcium	2.38	mmol/L		
				Creatinine	54.0	umol/L		
				Glucose, serum	4.61	mmol/L		
				Gamma-GT, 37 °C	12.5	U/L		
				Potassium	3.93	mmol/L		
				Magnesium	0.82	mmol/L		
				Sodium	137.6	mmol/L		
			Haematology	Basophils, abs.	0.05	10^9/L		
				Basophils, %	1.0	%		
				Eosinophils, abs.	0.11	10^9/L		
				Eosinophils, %	2.3	%		
				Haemoglobin	117.0	g/L		
				Haematocrit	0.35	L/L		
				Lymphocytes, abs.	1.83	10^9/L		
				Lymphocytes, %	38.4	%		
				Monocytes, abs.	0.33	10^9/L		
				Monocytes, %	6.9	%		
				Neutrophils, abs.	2.45	10^9/L		
				Neutrophils, %	51.4	%		
				Platelets	280	10^9/L		
				Erythrocytes	3.87	10^{12}/L	L	No
				Leucocytes	4.77	10^9/L		
			Haemostasis	APTT	26.7	s		
				Prothrombin Time (INR)	1.21	N/A	H	No
				Prothrombin Time (PT)	65.0	%	L	No
		FUP	Clinical Chemistry	ALT, 37 °C	13.4	U/L		
				Alkaline Phosphatase, 37 °C	35.4	U/L		

Sign.: Significant finding; L: Result considered low; H: Result considered high; SC: Screening; BL: Baseline; D: Day; FUP: Follow-up; Cohort A3: Treatment was applied under fasting and fed conditions in the same subjects;

Output generated by program 'NIC002_L16_2_8_Laboratory_V02_0_0'

Listing 16.2.8: Study subject data
Laboratory values

Part A

Cohort	Subject ID	Visit	Type	Measurement	Result		Flagged	Sign.
		FUP	Clinical Chemistry	AST, 37 °C	22.7	U/L		
				Bicarbonate	25.0	mmol/L		
				Bilirubin, total	8.5	umol/L		
				Urea/BUN	3.77	mmol/L		
				Calcium	2.42	mmol/L		
				Creatinine	47.7	umol/L		
				Glucose, serum	4.22	mmol/L		
				Gamma-GT, 37 °C	13.1	U/L		
				Potassium	3.86	mmol/L		
				Magnesium	0.80	mmol/L		
				Sodium	139.2	mmol/L		
			Haematology	Basophils, abs.	0.05	10^9/L		
				Basophils, %	1.1	%		
				Eosinophils, abs.	0.10	10^9/L		
				Eosinophils, %	2.3	%		
				Haemoglobin	115.0	g/L		
				Haematocrit	0.34	L/L	L	No
				Lymphocytes, abs.	1.66	10^9/L		
				Lymphocytes, %	38.0	%		
				Monocytes, abs.	0.31	10^9/L		
				Monocytes, %	7.1	%		
				Neutrophils, abs.	2.25	10^9/L		
				Neutrophils, %	51.5	%		
				Platelets	281	10^9/L		
				Erythrocytes	3.82	10^{12}/L	L	No
				Leucocytes	4.37	10^9/L		
			Haemostasis	APTT	26.6	s		
				Prothrombin Time (INR)	1.15	N/A		
				Prothrombin Time (PT)	71.8	%		
			Urine	Beta-HCG, urine	negative			

Sign.: Significant finding; L: Result considered low; H: Result considered high; SC: Screening; BL: Baseline; D: Day; FUP: Follow-up; Cohort A3: Treatment was applied under fasting and fed conditions in the same subjects;

Output generated by program 'NIC002_L16_2_8_Laboratory_V02_0_0'

Listing 16.2.8: Study subject data
Laboratory values

Part A

Cohort	Subject ID	Visit	Type	Measurement	Result		Flagged	Sign.
	7	SC	Clinical Chemistry	ALT, 37 °C	11.9	U/L		
				Alkaline Phosphatase, 37 °C	72.7	U/L		
				AST, 37 °C	25.3	U/L		
				Bicarbonate	27.5	mmol/L		
				Bilirubin, total	10.3	umol/L		
				Urea/BUN	3.61	mmol/L		
				Calcium	2.40	mmol/L		
				Creatinine	62.4	umol/L		
				Glucose, serum	4.81	mmol/L		
				Gamma-GT, 37 °C	12.2	U/L		
				Potassium	4.36	mmol/L		
				Creatinine Clearance MDRD	104	ml/min/1.73m		
				Magnesium	0.81	mmol/L		
				Sodium	138.3	mmol/L		
			Drugs	Amphetamines, Urine	negative			
				Barbiturates, Urine	negative			
				Benzodiazepines, Urine	negative			
				Cannabin., Urine	negative			
				Cocaine, Urine	negative			
				Methadone, Urine	negative			
				Opiates, Urine	negative			
			Haematology	Basophils, abs.	0.03	10^9/L		
				Basophils, %	0.8	%		
				Eosinophils, abs.	0.07	10^9/L		
				Eosinophils, %	1.9	%		
				Haemoglobin	126.0	g/L		
				Haematocrit	0.36	L/L		
				Lymphocytes, abs.	1.32	10^9/L		
				Lymphocytes, %	36.6	%		
				Monocytes, abs.	0.42	10^9/L		
				Monocytes, %	11.6	%		

Sign.: Significant finding; L: Result considered low; H: Result considered high; SC: Screening; BL: Baseline; D: Day; FUP: Follow-up; Cohort A3: Treatment was applied under fasting and fed conditions in the same subjects;

Output generated by program 'NIC002_L16_2_8_Laboratory_V02_0_0'

Listing 16.2.8: Study subject data
Laboratory values

Part A

Cohort	Subject ID	Visit	Type	Measurement	Result		Flagged	Sign.
	7	SC	Haematology	Neutrophils, abs.	1.77	10^9/L		
				Neutrophils, %	49.1	%		
				Platelets	332	10^9/L		
				Erythrocytes	3.91	10^{12}/L	L	No
				Leucocytes	3.61	10^9/L	L	No
			Haemostasis	APTT	27.7	s		
				Prothrombin Time (INR)	1.06	N/A		
				Prothrombin Time (PT)	89.2	%		
			Infectious Diseases	HBs-Ag (Hep. B Surf. Ag)	negative	N/A		
				Anti-HCV (Hep. C-AB)	non-reactive	N/A		
				HIV 1+2, AG/AB	negative	N/A		
			Urine	Bilirubin, urine (Stix)	negative			
				Blood (Ery/Hb), urine (Stix)	negative			
				Glucose, urine (Stix)	negative			
				Beta-HCG, urine	negative			
				Ketone, urine (Stix)	negative			
				Leucocytes, urine (Stix)	negative			
				Nitrite, urine (Stix)	negative			
				pH, urine (Stix)	7.5	neg.log[H+]		
				Protein, total, urine (Stix)	negative			
				Bacteria, Sediment	negative			
				Carbonate, Sediment	negative			
				Epithelial Cells, Sediment	6	per field		
				Erythrocytes, Sediment	0	per field		
				Casts granul., Sediment	0	per field		
				Casts hyaline, Sediment	0	per field		
				Leucocytes, Sediment	2	per field		
				Oxalate, Sediment	negative			
				Specific Gravity	<=1.005			
				Mucus, Sediment	negative			
				Triple Phosphate, Sediment	negative			

Sign.: Significant finding; L: Result considered low; H: Result considered high; SC: Screening; BL: Baseline; D: Day; FUP: Follow-up; Cohort A3: Treatment was applied under fasting and fed conditions in the same subjects;

Output generated by program 'NIC002_L16_2_8_Laboratory_V02_0_0'

Listing 16.2.8: Study subject data
Laboratory values

Part A

Cohort	Subject ID	Visit	Type	Measurement	Result		Flagged	Sign.
	7	SC	Urine	Urates, Sediment	negative			
				Urobilinogen, urine (Stix)	0.2	mg/dl		
		BL	Clinical Chemistry	ALT, 37 °C	18.6	U/L		
				Alkaline Phosphatase, 37 °C	72.9	U/L		
				AST, 37 °C	24.9	U/L		
				Bicarbonate	27.5	mmol/L		
				Bilirubin, total	7.4	umol/L		
				Urea/BUN	4.71	mmol/L		
				Calcium	2.39	mmol/L		
				Creatinine	62.1	umol/L		
				Glucose, serum	5.05	mmol/L		
				Gamma-GT, 37 °C	13.8	U/L		
				Potassium	4.40	mmol/L		
				Magnesium	0.81	mmol/L		
				Sodium	140.0	mmol/L		
			Drugs	Amphetamines, Urine	negative			
				Barbiturates, Urine	negative			
				Benzodiazepines, Urine	negative			
				Cannabin., Urine	negative			
				Cocaine, Urine	negative			
				Methadone, Urine	negative			
				Opiates, Urine	negative			
			Haematology	Basophils, abs.	0.03	10^9/L		
				Basophils, %	0.7	%		
				Eosinophils, abs.	0.13	10^9/L		
				Eosinophils, %	3.2	%		
				Haemoglobin	127.0	g/L		
				Haematocrit	0.36	L/L		
				Lymphocytes, abs.	1.32	10^9/L		
				Lymphocytes, %	32.8	%		

Sign.: Significant finding; L: Result considered low; H: Result considered high; SC: Screening; BL: Baseline; D: Day; FUP: Follow-up; Cohort A3: Treatment was applied under fasting and fed conditions in the same subjects;

Output generated by program 'NIC002_L16_2_8_Laboratory_V02_0_0'

Listing 16.2.8: Study subject data
Laboratory values

Part A

Cohort	Subject ID	Visit	Type	Measurement	Result		Flagged	Sign.
		BL	Haematology	Monocytes, abs.	0.41	10^9/L		
				Monocytes, %	10.2	%		
				Neutrophils, abs.	2.13	10^9/L		
				Neutrophils, %	53.1	%		
				Platelets	306	10^9/L		
				Erythrocytes	3.94	10^{12}/L	L	No
				Leucocytes	4.02	10^9/L		
			Haemostasis	APTT	26.7	s		
				Prothrombin Time (INR)	0.98	N/A		
				Prothrombin Time (PT)	107.0	%		
			Urine	Beta-HCG, urine	negative			
		D02	Clinical Chemistry	ALT, 37 °C	17.7	U/L		
				Alkaline Phosphatase, 37 °C	69.7	U/L		
				AST, 37 °C	24.1	U/L		
				Bicarbonate	27.9	mmol/L		
				Bilirubin, total	8.7	umol/L		
				Urea/BUN	4.40	mmol/L		
				Calcium	2.34	mmol/L		
				Creatinine	66.0	umol/L		
				Glucose, serum	4.58	mmol/L		
				Gamma-GT, 37 °C	14.9	U/L		
				Potassium	4.05	mmol/L		
				Magnesium	0.83	mmol/L		
				Sodium	138.6	mmol/L		
			Haematology	Basophils, abs.	0.05	10^9/L		
				Basophils, %	1.3	%		
				Eosinophils, abs.	0.14	10^9/L		
				Eosinophils, %	3.6	%		
				Haemoglobin	126.0	g/L		
				Haematocrit	0.36	L/L		

Sign.: Significant finding; L: Result considered low; H: Result considered high; SC: Screening; BL: Baseline; D: Day; FUP: Follow-up; Cohort A3: Treatment was applied under fasting and fed conditions in the same subjects;

Output generated by program 'NIC002_L16_2_8_Laboratory_V02_0_0'

Listing 16.2.8: Study subject data
Laboratory values

Part A

Cohort	Subject ID	Visit	Type	Measurement	Result		Flagged	Sign.
		D02	Haematology	Lymphocytes, abs.	1.61	10^9/L		
				Lymphocytes, %	41.4	%		
				Monocytes, abs.	0.43	10^9/L		
				Monocytes, %	11.1	%		
				Neutrophils, abs.	1.66	10^9/L		
				Neutrophils, %	42.6	%		
				Platelets	307	10^9/L		
				Erythrocytes	3.94	10^{12}/L	L	No
				Leucocytes	3.89	10^9/L		
			Haemostasis	APTT	27.2	s		
				Prothrombin Time (INR)	1.04	N/A		
				Prothrombin Time (PT)	93.9	%		
		FUP	Clinical Chemistry	ALT, 37 °C	21.9	U/L		
				Alkaline Phosphatase, 37 °C	75.7	U/L		
				AST, 37 °C	33.1	U/L		
				Bicarbonate	27.4	mmol/L		
				Bilirubin, total	10.5	umol/L		
				Urea/BUN	4.93	mmol/L		
				Calcium	2.40	mmol/L		
				Creatinine	68.4	umol/L		
				Glucose, serum	4.45	mmol/L		
				Gamma-GT, 37 °C	15.6	U/L		
				Potassium	3.95	mmol/L		
				Magnesium	0.86	mmol/L		
				Sodium	139.0	mmol/L		
			Haematology	Basophils, abs.	0.03	10^9/L		
				Basophils, %	1.1	%		
				Eosinophils, abs.	0.11	10^9/L		
				Eosinophils, %	3.9	%		
				Haemoglobin	120.0	g/L		

Sign.: Significant finding; L: Result considered low; H: Result considered high; SC: Screening; BL: Baseline; D: Day; FUP: Follow-up; Cohort A3: Treatment was applied under fasting and fed conditions in the same subjects;

Output generated by program 'NIC002_L16_2_8_Laboratory_V02_0_0'

Listing 16.2.8: Study subject data
Laboratory values

Part A

Cohort	Subject ID	Visit	Type	Measurement	Result		Flagged	Sign.
		FUP	Haematology	Haematocrit	0.34	L/L	L	No
				Lymphocytes, abs.	1.22	10^9/L		
				Lymphocytes, %	43.4	%		
				Monocytes, abs.	0.40	10^9/L		
				Monocytes, %	14.2	%		
				Neutrophils, abs.	1.05	10^9/L	L	No
				Neutrophils, %	37.4	%	L	No
				Platelets	304	10^9/L		
				Erythrocytes	3.70	10^{12}/L	L	No
				Leucocytes	2.81	10^9/L	L	No
			Haemostasis	APTT	27.1	s		
				Prothrombin Time (INR)	1.00	N/A		
				Prothrombin Time (PT)	101.5	%		
			Urine	Beta-HCG, urine	negative			
	8	SC	Clinical Chemistry	ALT, 37 °C	18.8	U/L		
				Alkaline Phosphatase, 37 °C	52.3	U/L		
				AST, 37 °C	24.5	U/L		
				Bicarbonate	28.0	mmol/L		
				Bilirubin, total	12.6	umol/L		
				Urea/BUN	2.77	mmol/L	L	No
				Calcium	2.41	mmol/L		
				Creatinine	65.0	umol/L		
				Glucose, serum	5.20	mmol/L		
				Gamma-GT, 37 °C	11.1	U/L		
				Potassium	4.23	mmol/L		
				Crea Clearance MDRD (female, afr.)	117	ml/min/1.73m		
				Creatinine Clearance MDRD	96	ml/min/1.73m		
				Magnesium	0.81	mmol/L		
				Sodium	139.3	mmol/L		

Sign.: Significant finding; L: Result considered low; H: Result considered high; SC: Screening; BL: Baseline; D: Day; FUP: Follow-up; Cohort A3: Treatment was applied under fasting and fed conditions in the same subjects;

Output generated by program 'NIC002_L16_2_8_Laboratory_V02_0_0'

Listing 16.2.8: Study subject data
Laboratory values

Part A

Cohort	Subject ID	Visit	Type	Measurement	Result		Flagged	Sign.
	8	SC	Drugs	Amphetamines, Urine	negative			
				Barbiturates, Urine	negative			
				Benzodiazepines, Urine	negative			
				Cannabin., Urine	negative			
				Cocaine, Urine	negative			
				Methadone, Urine	negative			
				Opiates, Urine	negative			
			Haematology	Basophils, abs.	0.03	10^9/L		
				Basophils, %	0.5	%		
				Eosinophils, abs.	0.11	10^9/L		
				Eosinophils, %	1.9	%		
				Haemoglobin	121.0	g/L		
				Haematocrit	0.35	L/L		
				Lymphocytes, abs.	1.71	10^9/L		
				Lymphocytes, %	29.5	%		
				Monocytes, abs.	0.38	10^9/L		
				Monocytes, %	6.6	%		
				Neutrophils, abs.	3.57	10^9/L		
				Neutrophils, %	61.5	%		
				Platelets	245	10^9/L		
				Erythrocytes	3.93	10^{12}/L	L	No
				Leucocytes	5.80	10^9/L		
			Haemostasis	APTT	25.1	s		
				Prothrombin Time (INR)	1.05	N/A		
				Prothrombin Time (PT)	91.5	%		
			Infectious Diseases	HBs-Ag (Hep. B Surf. Ag)	negative	N/A		
				Anti-HCV (Hep. C-AB)	non-reactive	N/A		
				HIV 1+2, AG/AB	negative	N/A		
			Urine	Bilirubin, urine (Stix)	negative			
				Blood (Ery/Hb), urine (Stix)	trace-intact		H	No
				Glucose, urine (Stix)	negative			

Sign.: Significant finding; L: Result considered low; H: Result considered high; SC: Screening; BL: Baseline; D: Day; FUP: Follow-up; Cohort A3: Treatment was applied under fasting and fed conditions in the same subjects;

Output generated by program 'NIC002_L16_2_8_Laboratory_V02_0_0'

Listing 16.2.8: Study subject data
Laboratory values

Part A

Cohort	Subject ID	Visit	Type	Measurement	Result		Flagged	Sign.
	8	SC	Urine	Beta-HCG, urine	negative			
				Ketone, urine (Stix)	negative			
				Leucocytes, urine (Stix)	negative			
				Nitrite, urine (Stix)	negative			
				pH, urine (Stix)	6.5	neg.log[H+]		
				Protein, total, urine (Stix)	negative			
				Bacteria, Sediment	negative			
				Carbonate, Sediment	negative			
				Epithelial Cells, Sediment	5	per field		
				Erythrocytes, Sediment	0	per field		
				Casts granul., Sediment	0	per field		
				Casts hyaline, Sediment	0	per field		
				Leucocytes, Sediment	1	per field		
				Oxalate, Sediment	negative			
				Specific Gravity	<=1.005			
				Mucus, Sediment	negative			
				Triple Phosphate, Sediment	negative			
				Urates, Sediment	negative			
				Urobilinogen, urine (Stix)	0.2	mg/dl		
		BL	Clinical Chemistry	ALT, 37 °C	17.0	U/L		
				Alkaline Phosphatase, 37 °C	52.4	U/L		
				AST, 37 °C	24.5	U/L		
				Bicarbonate	29.6	mmol/L		
				Bilirubin, total	13.4	umol/L		
				Urea/BUN	4.26	mmol/L		
				Calcium	2.38	mmol/L		
				Creatinine	66.4	umol/L		
				Glucose, serum	4.80	mmol/L		
				Gamma-GT, 37 °C	11.1	U/L		
				Potassium	3.88	mmol/L		

Sign.: Significant finding; L: Result considered low; H: Result considered high; SC: Screening; BL: Baseline; D: Day; FUP: Follow-up; Cohort A3: Treatment was applied under fasting and fed conditions in the same subjects;

Output generated by program 'NIC002_L16_2_8_Laboratory_V02_0_0'

Listing 16.2.8: Study subject data
Laboratory values

Part A

Cohort	Subject ID	Visit	Type	Measurement	Result		Flagged	Sign.
		BL	Clinical Chemistry	Magnesium	0.84	mmol/L		
				Sodium	139.4	mmol/L		
			Drugs	Amphetamines, Urine	negative			
				Barbiturates, Urine	negative			
				Benzodiazepines, Urine	negative			
				Cannabin., Urine	negative			
				Cocaine, Urine	negative			
				Methadone, Urine	negative			
				Opiates, Urine	negative			
			Haematology	Basophils, abs.	0.04	10^9/L		
				Basophils, %	0.8	%		
				Eosinophils, abs.	0.11	10^9/L		
				Eosinophils, %	2.1	%		
				Haemoglobin	120.0	g/L		
				Haematocrit	0.35	L/L		
				Lymphocytes, abs.	1.39	10^9/L		
				Lymphocytes, %	26.9	%		
				Monocytes, abs.	0.31	10^9/L		
				Monocytes, %	6.0	%		
				Neutrophils, abs.	3.31	10^9/L		
				Neutrophils, %	64.2	%		
				Platelets	212	10^9/L		
				Erythrocytes	3.91	10^{12}/L	L	No
				Leucocytes	5.16	10^9/L		
			Haemostasis	APTT	22.6	s		
				Prothrombin Time (INR)	1.05	N/A		
				Prothrombin Time (PT)	91.5	%		
			Urine	Beta-HCG, urine	negative			
		D02	Clinical Chemistry	ALT, 37 °C	19.8	U/L		
				Alkaline Phosphatase, 37 °C	53.9	U/L		

Sign.: Significant finding; L: Result considered low; H: Result considered high; SC: Screening; BL: Baseline; D: Day; FUP: Follow-up; Cohort A3: Treatment was applied under fasting and fed conditions in the same subjects;

Output generated by program 'NIC002_L16_2_8_Laboratory_V02_0_0'

Listing 16.2.8: Study subject data
Laboratory values

Part A

Cohort	Subject ID	Visit	Type	Measurement	Result		Flagged	Sign.
		D02	Clinical Chemistry	AST, 37 °C	26.1	U/L		
				Bicarbonate	28.7	mmol/L		
				Bilirubin, total	10.2	umol/L		
				Urea/BUN	4.46	mmol/L		
				Calcium	2.41	mmol/L		
				Creatinine	66.7	umol/L		
				Glucose, serum	4.76	mmol/L		
				Gamma-GT, 37 °C	11.6	U/L		
				Potassium	4.10	mmol/L		
				Magnesium	0.82	mmol/L		
				Sodium	139.0	mmol/L		
			Haematology	Basophils, abs.	0.04	10^9/L		
				Basophils, %	0.8	%		
				Eosinophils, abs.	0.07	10^9/L		
				Eosinophils, %	1.4	%		
				Haemoglobin	127.0	g/L		
				Haematocrit	0.36	L/L		
				Lymphocytes, abs.	1.42	10^9/L		
				Lymphocytes, %	29.4	%		
				Monocytes, abs.	0.25	10^9/L	L	No
				Monocytes, %	5.2	%	L	No
				Neutrophils, abs.	3.05	10^9/L		
				Neutrophils, %	63.2	%		
				Platelets	245	10^9/L		
				Erythrocytes	4.08	10^{12}/L		
				Leucocytes	4.83	10^9/L		
			Haemostasis	APTT	23.9	s		
				Prothrombin Time (INR)	1.05	N/A		
				Prothrombin Time (PT)	91.5	%		
		FUP	Clinical Chemistry	ALT, 37 °C	17.8	U/L		

Sign.: Significant finding; L: Result considered low; H: Result considered high; SC: Screening; BL: Baseline; D: Day; FUP: Follow-up; Cohort A3: Treatment was applied under fasting and fed conditions in the same subjects;

Output generated by program 'NIC002_L16_2_8_Laboratory_V02_0_0'

Listing 16.2.8: Study subject data
Laboratory values

Part A

Cohort	Subject ID	Visit	Type	Measurement	Result		Flagged	Sign.
		FUP	Clinical Chemistry	Alkaline Phosphatase, 37 °C	53.3	U/L		
				AST, 37 °C	23.9	U/L		
				Bicarbonate	29.8	mmol/L		
				Bilirubin, total	11.2	umol/L		
				Urea/BUN	2.89	mmol/L		
				Calcium	2.40	mmol/L		
				Creatinine	63.7	umol/L		
				Glucose, serum	4.40	mmol/L		
				Gamma-GT, 37 °C	12.2	U/L		
				Potassium	3.88	mmol/L		
				Magnesium	0.87	mmol/L		
				Sodium	138.6	mmol/L		
			Haematology	Basophils, abs.	0.05	10^9/L		
				Basophils, %	1.0	%		
				Eosinophils, abs.	0.16	10^9/L		
				Eosinophils, %	3.0	%		
				Haemoglobin	120.0	g/L		
				Haematocrit	0.35	L/L		
				Lymphocytes, abs.	1.83	10^9/L		
				Lymphocytes, %	34.8	%		
				Monocytes, abs.	0.35	10^9/L		
				Monocytes, %	6.7	%		
				Neutrophils, abs.	2.87	10^9/L		
				Neutrophils, %	54.5	%		
				Platelets	246	10^9/L		
				Erythrocytes	3.90	10^{12}/L	L	No
				Leucocytes	5.26	10^9/L		
			Haemostasis	APTT	24.4	s		
				Prothrombin Time (INR)	1.05	N/A		
				Prothrombin Time (PT)	91.5	%		
			Urine	Beta-HCG, urine	negative			

Sign.: Significant finding; L: Result considered low; H: Result considered high; SC: Screening; BL: Baseline; D: Day; FUP: Follow-up; Cohort A3: Treatment was applied under fasting and fed conditions in the same subjects;

Output generated by program 'NIC002_L16_2_8_Laboratory_V02_0_0'

Listing 16.2.8: Study subject data
Laboratory values

Part A

Cohort	Subject ID	Visit	Type	Measurement	Result		Flagged	Sign.
Cohort A2	13	SC	Clinical Chemistry	ALT, 37 °C	13.5	U/L		
				Alkaline Phosphatase, 37 °C	65.8	U/L		
				AST, 37 °C	20.0	U/L		
				Bicarbonate	23.5	mmol/L		
				Bilirubin, total	15.2	umol/L		
				Urea/BUN	3.24	mmol/L		
				Calcium	2.37	mmol/L		
				Creatinine	53.6	umol/L		
				Glucose, serum	4.84	mmol/L		
				Gamma-GT, 37 °C	11.5	U/L		
				Potassium	4.42	mmol/L		
				Creatinine Clearance MDRD	118	ml/min/1.73m		
				Magnesium	0.84	mmol/L		
				Sodium	138.5	mmol/L		
			Drugs	Amphetamines, Urine	negative			
				Barbiturates, Urine	negative			
				Benzodiazepines, Urine	negative			
				Cannabin., Urine	negative			
				Cocaine, Urine	negative			
				Methadone, Urine	negative			
				Opiates, Urine	negative			
			Haematology	Basophils, abs.	0.02	10^9/L		
				Basophils, %	0.5	%		
				Eosinophils, abs.	0.12	10^9/L		
				Eosinophils, %	3.0	%		
				Haemoglobin	126.0	g/L		
				Haematocrit	0.36	L/L		
				Lymphocytes, abs.	1.26	10^9/L		
				Lymphocytes, %	31.7	%		
				Monocytes, abs.	0.32	10^9/L		

Sign.: Significant finding; L: Result considered low; H: Result considered high; SC: Screening; BL: Baseline; D: Day; FUP: Follow-up; Cohort A3: Treatment was applied under fasting and fed conditions in the same subjects;

Output generated by program 'NIC002_L16_2_8_Laboratory_V02_0_0'

Listing 16.2.8: Study subject data
Laboratory values

Part A

Cohort	Subject ID	Visit	Type	Measurement	Result		Flagged	Sign.
Cohort A2	13	SC	Haematology	Monocytes, %	8.1	%		
				Neutrophils, abs.	2.25	10^9/L		
				Neutrophils, %	56.7	%		
				Platelets	267	10^9/L		
				Erythrocytes	3.89	10^{12}/L	L	No
				Leucocytes	3.97	10^9/L		
			Haemostasis	APTT	24.1	s		
				Prothrombin Time (INR)	1.04	N/A		
				Prothrombin Time (PT)	93.9	%		
			Infectious Diseases	HBs-Ag (Hep. B Surf. Ag)	negative	N/A		
				Anti-HCV (Hep. C-AB)	non-reactive	N/A		
				HIV 1+2, AG/AB	negative	N/A		
			Urine	Bilirubin, urine (Stix)	negative			
				Blood (Ery/Hb), urine (Stix)	trace-intact		H	No
				Glucose, urine (Stix)	negative			
				Beta-HCG, urine	negative			
				Ketone, urine (Stix)	negative			
				Leucocytes, urine (Stix)	negative			
				Nitrite, urine (Stix)	negative			
				pH, urine (Stix)	6.0	neg.log[H+]		
				Protein, total, urine (Stix)	negative			
				Bacteria, Sediment	positive		H	No
				Carbonate, Sediment	negative			
				Epithelial Cells, Sediment	10	per field		
				Erythrocytes, Sediment	1	per field		
				Casts granul., Sediment	0	per field		
				Casts hyaline, Sediment	0	per field		
				Leucocytes, Sediment	2	per field		
				Oxalate, Sediment	negative			
				Specific Gravity	<=1.005			
				Mucus, Sediment	positive		H	No

Sign.: Significant finding; L: Result considered low; H: Result considered high; SC: Screening; BL: Baseline; D: Day; FUP: Follow-up; Cohort A3: Treatment was applied under fasting and fed conditions in the same subjects;

Output generated by program 'NIC002_L16_2_8_Laboratory_V02_0_0'

Listing 16.2.8: Study subject data
Laboratory values

Part A

Cohort	Subject ID	Visit	Type	Measurement	Result		Flagged	Sign.
Cohort A2	13	SC	Urine	Triple Phosphate, Sediment	negative			
				Urates, Sediment	negative			
				Urobilinogen, urine (Stix)	0.2	mg/dl		
		BL	Clinical Chemistry	ALT, 37 °C	8.3	U/L		
				Alkaline Phosphatase, 37 °C	58.7	U/L		
				AST, 37 °C	17.3	U/L		
				Bicarbonate	25.5	mmol/L		
				Bilirubin, total	18.0	umol/L		
				Urea/BUN	3.10	mmol/L		
				Calcium	2.33	mmol/L		
				Creatinine	50.7	umol/L		
				Glucose, serum	5.18	mmol/L		
				Gamma-GT, 37 °C	11.8	U/L		
				Potassium	4.38	mmol/L		
				Magnesium	0.85	mmol/L		
				Sodium	137.8	mmol/L		
			Drugs	Amphetamines, Urine	negative			
				Barbiturates, Urine	negative			
				Benzodiazepines, Urine	negative			
				Cannabin., Urine	negative			
				Cocaine, Urine	negative			
				Methadone, Urine	negative			
				Opiates, Urine	negative			
			Haematology	Basophils, abs.	0.04	10^9/L		
				Basophils, %	0.9	%		
				Eosinophils, abs.	0.15	10^9/L		
				Eosinophils, %	3.3	%		
				Haemoglobin	127.0	g/L		
				Haematocrit	0.36	L/L		
				Lymphocytes, abs.	1.38	10^9/L		

Sign.: Significant finding; L: Result considered low; H: Result considered high; SC: Screening; BL: Baseline; D: Day; FUP: Follow-up; Cohort A3: Treatment was applied under fasting and fed conditions in the same subjects;

Output generated by program 'NIC002_L16_2_8_Laboratory_V02_0_0'

Listing 16.2.8: Study subject data
Laboratory values

Part A

Cohort	Subject ID	Visit	Type	Measurement	Result		Flagged	Sign.
		BL	Haematology	Lymphocytes, %	30.4	%		
				Monocytes, abs.	0.52	10^9/L		
				Monocytes, %	11.5	%		
				Neutrophils, abs.	2.45	10^9/L		
				Neutrophils, %	53.9	%		
				Platelets	245	10^9/L		
				Erythrocytes	3.93	10^{12}/L	L	No
				Leucocytes	4.54	10^9/L		
			Haemostasis	APTT	24.5	s		
				Prothrombin Time (INR)	1.05	N/A		
				Prothrombin Time (PT)	91.5	%		
			Urine	Beta-HCG, urine	negative			
		D02	Clinical Chemistry	ALT, 37 °C	7.7	U/L		
				Alkaline Phosphatase, 37 °C	59.1	U/L		
				AST, 37 °C	17.6	U/L		
				Bicarbonate	22.6	mmol/L		
				Bilirubin, total	11.6	umol/L		
				Urea/BUN	3.17	mmol/L		
				Calcium	2.33	mmol/L		
				Creatinine	49.6	umol/L		
				Glucose, serum	4.65	mmol/L		
				Gamma-GT, 37 °C	11.3	U/L		
				Potassium	3.97	mmol/L		
				Magnesium	0.77	mmol/L		
				Sodium	136.0	mmol/L		
			Haematology	Basophils, abs.	0.02	10^9/L		
				Basophils, %	0.4	%		
				Eosinophils, abs.	0.08	10^9/L		
				Eosinophils, %	1.6	%		
				Haemoglobin	121.0	g/L		

Sign.: Significant finding; L: Result considered low; H: Result considered high; SC: Screening; BL: Baseline; D: Day; FUP: Follow-up; Cohort A3: Treatment was applied under fasting and fed conditions in the same subjects;

Output generated by program 'NIC002_L16_2_8_Laboratory_V02_0_0'

Listing 16.2.8: Study subject data
Laboratory values

Part A

Cohort	Subject ID	Visit	Type	Measurement	Result		Flagged	Sign.
		D02	Haematology	Haematocrit	0.34	L/L	L	No
				Lymphocytes, abs.	1.44	10^9/L		
				Lymphocytes, %	29.3	%		
				Monocytes, abs.	0.38	10^9/L		
				Monocytes, %	7.7	%		
				Neutrophils, abs.	2.99	10^9/L		
				Neutrophils, %	61.0	%		
				Platelets	260	10^9/L		
				Erythrocytes	3.71	10^{12}/L	L	No
				Leucocytes	4.91	10^9/L		
			Haemostasis	APTT	25.9	s		
				Prothrombin Time (INR)	1.10	N/A		
				Prothrombin Time (PT)	80.8	%		
		FUP	Clinical Chemistry	ALT, 37 °C	9.4	U/L		
				Alkaline Phosphatase, 37 °C	57.1	U/L		
				AST, 37 °C	19.3	U/L		
				Bicarbonate	23.9	mmol/L		
				Bilirubin, total	12.7	umol/L		
				Urea/BUN	3.11	mmol/L		
				Calcium	2.26	mmol/L		
				Creatinine	48.2	umol/L		
				Glucose, serum	4.42	mmol/L		
				Gamma-GT, 37 °C	9.7	U/L		
				Potassium	4.22	mmol/L		
				Magnesium	0.85	mmol/L		
				Sodium	137.5	mmol/L		
			Haematology	Basophils, abs.	0.02	10^9/L		
				Basophils, %	0.5	%		
				Eosinophils, abs.	0.11	10^9/L		
				Eosinophils, %	3.0	%		

Sign.: Significant finding; L: Result considered low; H: Result considered high; SC: Screening; BL: Baseline; D: Day; FUP: Follow-up; Cohort A3: Treatment was applied under fasting and fed conditions in the same subjects;

Output generated by program 'NIC002_L16_2_8_Laboratory_V02_0_0'

Listing 16.2.8: Study subject data
Laboratory values

Part A

Cohort	Subject ID	Visit	Type	Measurement	Result		Flagged	Sign.
		FUP	Haematology	Haemoglobin	119.0	g/L		
				Haematocrit	0.34	L/L	L	No
				Lymphocytes, abs.	1.22	10^9/L		
				Lymphocytes, %	33.1	%		
				Monocytes, abs.	0.41	10^9/L		
				Monocytes, %	11.1	%		
				Neutrophils, abs.	1.93	10^9/L		
				Neutrophils, %	52.3	%		
				Platelets	254	10^9/L		
				Erythrocytes	3.69	10^{12}/L	L	No
				Leucocytes	3.69	10^9/L		
			Haemostasis	APTT	25.5	s		
				Prothrombin Time (INR)	1.05	N/A		
				Prothrombin Time (PT)	91.5	%		
			Urine	Beta-HCG, urine	negative			
	19	SC	Clinical Chemistry	ALT, 37 °C	16.2	U/L		
				Alkaline Phosphatase, 37 °C	45.2	U/L		
				AST, 37 °C	22.3	U/L		
				Bicarbonate	24.3	mmol/L		
				Bilirubin, total	11.1	umol/L		
				Urea/BUN	2.85	mmol/L		
				Calcium	2.39	mmol/L		
				Creatinine	67.0	umol/L		
				Glucose, serum	4.55	mmol/L		
				Gamma-GT, 37 °C	14.6	U/L		
				Potassium	4.66	mmol/L		
				Creatinine Clearance MDRD	90	ml/min/1.73m		
				Magnesium	0.80	mmol/L		
				Sodium	136.6	mmol/L		
			Drugs	Amphetamines, Urine	negative			

Sign.: Significant finding; L: Result considered low; H: Result considered high; SC: Screening; BL: Baseline; D: Day; FUP: Follow-up; Cohort A3: Treatment was applied under fasting and fed conditions in the same subjects;

Output generated by program 'NIC002_L16_2_8_Laboratory_V02_0_0'

Listing 16.2.8: Study subject data
Laboratory values

Part A

Cohort	Subject ID	Visit	Type	Measurement	Result		Flagged	Sign.
	19	SC	Drugs	Barbiturates, Urine	negative			
				Benzodiazepines, Urine	negative			
				Cannabin., Urine	negative			
				Cocaine, Urine	negative			
				Methadone, Urine	negative			
				Opiates, Urine	negative			
			Haematology	Basophils, abs.	0.03	10^9/L		
				Basophils, %	0.4	%		
				Eosinophils, abs.	0.17	10^9/L		
				Eosinophils, %	2.5	%		
				Haemoglobin	119.0	g/L		
				Haematocrit	0.35	L/L		
				Lymphocytes, abs.	1.95	10^9/L		
				Lymphocytes, %	28.3	%		
				Monocytes, abs.	0.48	10^9/L		
				Monocytes, %	7.0	%		
				Neutrophils, abs.	4.26	10^9/L		
				Neutrophils, %	61.8	%		
				Platelets	266	10^9/L		
				Erythrocytes	3.90	10^{12}/L	L	No
				Leucocytes	6.89	10^9/L		
			Haemostasis	APTT	26.3	s		
				Prothrombin Time (INR)	1.05	N/A		
				Prothrombin Time (PT)	91.5	%		
			Infectious Diseases	HBs-Ag (Hep. B Surf. Ag)	negative	N/A		
				Anti-HCV (Hep. C-AB)	non-reactive	N/A		
				HIV 1+2, AG/AB	negative	N/A		
			Urine	Bilirubin, urine (Stix)	negative			
				Blood (Ery/Hb), urine (Stix)	1+		H	No
				Glucose, urine (Stix)	negative			
				Beta-HCG, urine	negative			

Sign.: Significant finding; L: Result considered low; H: Result considered high; SC: Screening; BL: Baseline; D: Day; FUP: Follow-up; Cohort A3: Treatment was applied under fasting and fed conditions in the same subjects;

Output generated by program 'NIC002_L16_2_8_Laboratory_V02_0_0'

Listing 16.2.8: Study subject data
Laboratory values

Part A

Cohort	Subject ID	Visit	Type	Measurement	Result		Flagged	Sign.
	19	SC	Urine	Ketone, urine (Stix)	1+		H	No
				Leucocytes, urine (Stix)	1+		H	No
				Nitrite, urine (Stix)	negative			
				pH, urine (Stix)	6.0	neg.log[H+]		
				Protein, total, urine (Stix)	negative			
				Bacteria, Sediment	positive		H	No
				Carbonate, Sediment	negative			
				Epithelial Cells, Sediment	5	per field		
				Erythrocytes, Sediment	6	per field	H	No
				Casts granul., Sediment	0	per field		
				Casts hyaline, Sediment	0	per field		
				Leucocytes, Sediment	10	per field	H	No
				Oxalate, Sediment	negative			
				Specific Gravity	1.015			
				Mucus, Sediment	positive		H	No
				Triple Phosphate, Sediment	negative			
				Urates, Sediment	negative			
				Urobilinogen, urine (Stix)	0.2	mg/dl		
		BL	Clinical Chemistry	ALT, 37 °C	17.4	U/L		
				Alkaline Phosphatase, 37 °C	50.3	U/L		
				AST, 37 °C	20.3	U/L		
				Bicarbonate	25.7	mmol/L		
				Bilirubin, total	6.1	umol/L		
				Urea/BUN	4.90	mmol/L		
				Calcium	2.45	mmol/L		
				Creatinine	65.7	umol/L		
				Glucose, serum	4.43	mmol/L		
				Gamma-GT, 37 °C	15.5	U/L		
				Potassium	3.87	mmol/L		
				Magnesium	0.78	mmol/L		

Sign.: Significant finding; L: Result considered low; H: Result considered high; SC: Screening; BL: Baseline; D: Day; FUP: Follow-up; Cohort A3: Treatment was applied under fasting and fed conditions in the same subjects;

Output generated by program 'NIC002_L16_2_8_Laboratory_V02_0_0'

Listing 16.2.8: Study subject data
Laboratory values

Part A

Cohort	Subject ID	Visit	Type	Measurement	Result		Flagged	Sign.
		BL	Clinical Chemistry	Sodium	139.7	mmol/L		
			Drugs	Amphetamines, Urine	negative			
				Barbiturates, Urine	negative			
				Benzodiazepines, Urine	negative			
				Cannabin., Urine	negative			
				Cocaine, Urine	negative			
				Methadone, Urine	negative			
				Opiates, Urine	negative			
			Haematology	Basophils, abs.	0.03	10^9/L		
				Basophils, %	0.4	%		
				Eosinophils, abs.	0.22	10^9/L		
				Eosinophils, %	2.8	%		
				Haemoglobin	123.0	g/L		
				Haematocrit	0.36	L/L		
				Lymphocytes, abs.	1.82	10^9/L		
				Lymphocytes, %	23.5	%		
				Monocytes, abs.	0.45	10^9/L		
				Monocytes, %	5.8	%		
				Neutrophils, abs.	5.24	10^9/L		
				Neutrophils, %	67.5	%		
				Platelets	294	10^9/L		
				Erythrocytes	4.03	10^{12}/L		
				Leucocytes	7.76	10^9/L		
			Haemostasis	APTT	25.9	s		
				Prothrombin Time (INR)	1.00	N/A		
				Prothrombin Time (PT)	101.5	%		
			Urine	Beta-HCG, urine	negative			
		D02	Clinical Chemistry	ALT, 37 °C	14.7	U/L		
				Alkaline Phosphatase, 37 °C	50.0	U/L		
				AST, 37 °C	21.7	U/L		

Sign.: Significant finding; L: Result considered low; H: Result considered high; SC: Screening; BL: Baseline; D: Day; FUP: Follow-up; Cohort A3: Treatment was applied under fasting and fed conditions in the same subjects;

Output generated by program 'NIC002_L16_2_8_Laboratory_V02_0_0'

Listing 16.2.8: Study subject data
Laboratory values

Part A

Cohort	Subject ID	Visit	Type	Measurement	Result		Flagged	Sign.
		D02	Clinical Chemistry	Bicarbonate	23.8	mmol/L		
				Bilirubin, total	9.2	umol/L		
				Urea/BUN	3.11	mmol/L		
				Calcium	2.40	mmol/L		
				Creatinine	62.4	umol/L		
				Glucose, serum	4.23	mmol/L		
				Gamma-GT, 37 °C	15.4	U/L		
				Potassium	4.18	mmol/L		
				Magnesium	0.79	mmol/L		
				Sodium	137.8	mmol/L		
			Haematology	Basophils, abs.	0.04	10^9/L		
				Basophils, %	0.6	%		
				Eosinophils, abs.	0.14	10^9/L		
				Eosinophils, %	2.0	%		
				Haemoglobin	130.0	g/L		
				Haematocrit	0.38	L/L		
				Lymphocytes, abs.	1.17	10^9/L		
				Lymphocytes, %	16.6	%	L	No
				Monocytes, abs.	0.50	10^9/L		
				Monocytes, %	7.1	%		
				Neutrophils, abs.	5.20	10^9/L		
				Neutrophils, %	73.7	%	H	No
				Platelets	309	10^9/L		
				Erythrocytes	4.30	10^{12}/L		
				Leucocytes	7.05	10^9/L		
			Haemostasis	APTT	25.3	s		
				Prothrombin Time (INR)	1.05	N/A		
				Prothrombin Time (PT)	91.5	%		
		FUP	Clinical Chemistry	ALT, 37 °C	16.0	U/L		
				Alkaline Phosphatase, 37 °C	56.0	U/L		

Sign.: Significant finding; L: Result considered low; H: Result considered high; SC: Screening; BL: Baseline; D: Day; FUP: Follow-up; Cohort A3: Treatment was applied under fasting and fed conditions in the same subjects;

Output generated by program 'NIC002_L16_2_8_Laboratory_V02_0_0'

Listing 16.2.8: Study subject data
Laboratory values

Part A

Cohort	Subject ID	Visit	Type	Measurement	Result		Flagged	Sign.
		FUP	Clinical Chemistry	AST, 37 °C	24.3	U/L		
				Bicarbonate	25.3	mmol/L		
				Bilirubin, total	5.4	umol/L		
				Urea/BUN	2.95	mmol/L		
				Calcium	2.39	mmol/L		
				Creatinine	76.7	umol/L		
				Glucose, serum	4.75	mmol/L		
				Gamma-GT, 37 °C	15.5	U/L		
				Potassium	4.18	mmol/L		
				Magnesium	0.79	mmol/L		
				Sodium	138.2	mmol/L		
			Haematology	Basophils, abs.	0.03	10^9/L		
				Basophils, %	0.4	%		
				Eosinophils, abs.	0.13	10^9/L		
				Eosinophils, %	1.9	%		
				Haemoglobin	121.0	g/L		
				Haematocrit	0.35	L/L		
				Lymphocytes, abs.	1.26	10^9/L		
				Lymphocytes, %	18.0	%		
				Monocytes, abs.	0.64	10^9/L		
				Monocytes, %	9.1	%		
				Neutrophils, abs.	4.94	10^9/L		
				Neutrophils, %	70.6	%	H	No
				Platelets	280	10^9/L		
				Erythrocytes	3.97	10^{12}/L	L	No
				Leucocytes	7.00	10^9/L		
			Haemostasis	APTT	23.6	s		
				Prothrombin Time (INR)	0.97	N/A		
				Prothrombin Time (PT)	109.9	%		
			Urine	Beta-HCG, urine	negative			

Sign.: Significant finding; L: Result considered low; H: Result considered high; SC: Screening; BL: Baseline; D: Day; FUP: Follow-up; Cohort A3: Treatment was applied under fasting and fed conditions in the same subjects;

Output generated by program 'NIC002_L16_2_8_Laboratory_V02_0_0'

Listing 16.2.8: Study subject data
Laboratory values

Part A

Cohort	Subject ID	Visit	Type	Measurement	Result		Flagged	Sign.
	21	SC	Clinical Chemistry	ALT, 37 °C	13.3	U/L		
				Alkaline Phosphatase, 37 °C	60.5	U/L		
				AST, 37 °C	19.8	U/L		
				Bicarbonate	24.0	mmol/L		
				Bilirubin, total	8.5	umol/L		
				Urea/BUN	5.15	mmol/L		
				Calcium	2.21	mmol/L		
				Creatinine	56.0	umol/L		
				Glucose, serum	4.20	mmol/L		
				Gamma-GT, 37 °C	9.2	U/L		
				Potassium	3.94	mmol/L		
				Creatinine Clearance MDRD	106	ml/min/1.73m		
				Magnesium	0.82	mmol/L		
				Sodium	136.0	mmol/L		
			Drugs	Amphetamines, Urine	negative			
				Barbiturates, Urine	negative			
				Benzodiazepines, Urine	negative			
				Cannabin., Urine	negative			
				Cocaine, Urine	negative			
				Methadone, Urine	negative			
				Opiates, Urine	negative			
			Haematology	Basophils, abs.	0.01	10^9/L		
				Basophils, %	0.2	%		
				Eosinophils, abs.	0.11	10^9/L		
				Eosinophils, %	2.7	%		
				Haemoglobin	129.0	g/L		
				Haematocrit	0.38	L/L		
				Lymphocytes, abs.	1.41	10^9/L		
				Lymphocytes, %	34.6	%		
				Monocytes, abs.	0.47	10^9/L		
				Monocytes, %	11.5	%		

Sign.: Significant finding; L: Result considered low; H: Result considered high; SC: Screening; BL: Baseline; D: Day; FUP: Follow-up; Cohort A3: Treatment was applied under fasting and fed conditions in the same subjects;

Output generated by program 'NIC002_L16_2_8_Laboratory_V02_0_0'

Listing 16.2.8: Study subject data
Laboratory values

Part A

Cohort	Subject ID	Visit	Type	Measurement	Result		Flagged	Sign.
	21	SC	Haematology	Neutrophils, abs.	2.07	10^9/L		
				Neutrophils, %	51.0	%		
				Platelets	167	10^9/L	L	No
				Erythrocytes	4.30	10^{12}/L		
				Leucocytes	4.07	10^9/L		
			Haemostasis	APTT	26.5	s		
				Prothrombin Time (INR)	1.05	N/A		
				Prothrombin Time (PT)	91.5	%		
			Infectious Diseases	HBs-Ag (Hep. B Surf. Ag)	negative	N/A		
				Anti-HCV (Hep. C-AB)	non-reactive	N/A		
				HIV 1+2, AG/AB	negative	N/A		
			Urine	Bilirubin, urine (Stix)	negative			
				Blood (Ery/Hb), urine (Stix)	negative			
				Glucose, urine (Stix)	negative			
				Beta-HCG, urine	negative			
				Ketone, urine (Stix)	negative			
				Leucocytes, urine (Stix)	negative			
				Nitrite, urine (Stix)	negative			
				pH, urine (Stix)	6.0	neg.log[H+]		
				Protein, total, urine (Stix)	negative			
				Specific Gravity	1.020			
				Urobilinogen, urine (Stix)	0.2	mg/dl		
		BL	Clinical Chemistry	ALT, 37 °C	12.2	U/L		
				Alkaline Phosphatase, 37 °C	68.7	U/L		
				AST, 37 °C	17.8	U/L		
				Bicarbonate	24.0	mmol/L		
				Bilirubin, total	9.1	umol/L		
				Urea/BUN	5.34	mmol/L		
				Calcium	2.22	mmol/L		
				Creatinine	62.7	umol/L		

Sign.: Significant finding; L: Result considered low; H: Result considered high; SC: Screening; BL: Baseline; D: Day; FUP: Follow-up; Cohort A3: Treatment was applied under fasting and fed conditions in the same subjects;

Output generated by program 'NIC002_L16_2_8_Laboratory_V02_0_0'

Listing 16.2.8: Study subject data
Laboratory values

Part A

Cohort	Subject ID	Visit	Type	Measurement	Result		Flagged	Sign.
		BL	Clinical Chemistry	Glucose, serum	4.85	mmol/L		
				Gamma-GT, 37 °C	9.0	U/L		
				Potassium	4.14	mmol/L		
				Magnesium	0.79	mmol/L		
				Sodium	137.4	mmol/L		
			Drugs	Amphetamines, Urine	negative			
				Barbiturates, Urine	negative			
				Benzodiazepines, Urine	negative			
				Cannabin., Urine	negative			
				Cocaine, Urine	negative			
				Methadone, Urine	negative			
				Opiates, Urine	negative			
			Haematology	Basophils, abs.	0.01	10^9/L		
				Basophils, %	0.2	%		
				Eosinophils, abs.	0.11	10^9/L		
				Eosinophils, %	2.2	%		
				Haemoglobin	129.0	g/L		
				Haematocrit	0.38	L/L		
				Lymphocytes, abs.	1.38	10^9/L		
				Lymphocytes, %	27.5	%		
				Monocytes, abs.	0.45	10^9/L		
				Monocytes, %	9.0	%		
				Neutrophils, abs.	3.06	10^9/L		
				Neutrophils, %	61.1	%		
				Platelets	176	10^9/L		
				Erythrocytes	4.29	10^{12}/L		
				Leucocytes	5.01	10^9/L		
			Haemostasis	APTT	26.1	s		
				Prothrombin Time (INR)	1.05	N/A		
				Prothrombin Time (PT)	91.5	%		
			Urine	Beta-HCG, urine	negative			

Sign.: Significant finding; L: Result considered low; H: Result considered high; SC: Screening; BL: Baseline; D: Day; FUP: Follow-up; Cohort A3: Treatment was applied under fasting and fed conditions in the same subjects;

Output generated by program 'NIC002_L16_2_8_Laboratory_V02_0_0'

Listing 16.2.8: Study subject data
Laboratory values

Part A

Cohort	Subject ID	Visit	Type	Measurement	Result		Flagged	Sign.
		D02	Clinical Chemistry	ALT, 37 °C	13.4	U/L		
				Alkaline Phosphatase, 37 °C	70.0	U/L		
				AST, 37 °C	17.0	U/L		
				Bicarbonate	26.0	mmol/L		
				Bilirubin, total	7.9	umol/L		
				Urea/BUN	4.97	mmol/L		
				Calcium	2.26	mmol/L		
				Creatinine	55.6	umol/L		
				Glucose, serum	4.47	mmol/L		
				Gamma-GT, 37 °C	9.2	U/L		
				Potassium	4.56	mmol/L		
				Magnesium	0.88	mmol/L		
				Sodium	136.5	mmol/L		
			Haematology	Basophils, abs.	0.01	10^9/L		
				Basophils, %	0.2	%		
				Eosinophils, abs.	0.11	10^9/L		
				Eosinophils, %	2.7	%		
				Haemoglobin	129.0	g/L		
				Haematocrit	0.38	L/L		
				Lymphocytes, abs.	1.58	10^9/L		
				Lymphocytes, %	38.3	%		
				Monocytes, abs.	0.39	10^9/L		
				Monocytes, %	9.4	%		
				Neutrophils, abs.	2.04	10^9/L		
				Neutrophils, %	49.4	%		
				Platelets	196	10^9/L		
				Erythrocytes	4.29	10^{12}/L		
				Leucocytes	4.13	10^9/L		
			Haemostasis	APTT	25.9	s		
				Prothrombin Time (INR)	1.05	N/A		

Sign.: Significant finding; L: Result considered low; H: Result considered high; SC: Screening; BL: Baseline; D: Day; FUP: Follow-up; Cohort A3: Treatment was applied under fasting and fed conditions in the same subjects;

Output generated by program 'NIC002_L16_2_8_Laboratory_V02_0_0'

Listing 16.2.8: Study subject data
Laboratory values

Part A

Cohort	Subject ID	Visit	Type	Measurement	Result		Flagged	Sign.
		D02	Haemostasis	Prothrombin Time (PT)	91.5	%		
		FUP	Clinical Chemistry	ALT, 37 °C	13.1	U/L		
				Alkaline Phosphatase, 37 °C	65.7	U/L		
				AST, 37 °C	19.5	U/L		
				Bicarbonate	24.4	mmol/L		
				Bilirubin, total	7.3	umol/L		
				Urea/BUN	4.63	mmol/L		
				Calcium	2.20	mmol/L		
				Creatinine	57.1	umol/L		
				Glucose, serum	4.55	mmol/L		
				Gamma-GT, 37 °C	8.8	U/L		
				Potassium	4.50	mmol/L		
				Magnesium	0.87	mmol/L		
				Sodium	138.9	mmol/L		
			Haematology	Basophils, abs.	0.02	10^9/L		
				Basophils, %	0.5	%		
				Eosinophils, abs.	0.15	10^9/L		
				Eosinophils, %	4.0	%		
				Haemoglobin	125.0	g/L		
				Haematocrit	0.36	L/L		
				Lymphocytes, abs.	1.53	10^9/L		
				Lymphocytes, %	40.9	%		
				Monocytes, abs.	0.34	10^9/L		
				Monocytes, %	9.1	%		
				Neutrophils, abs.	1.70	10^9/L		
				Neutrophils, %	45.5	%		
				Platelets	179	10^9/L		
				Erythrocytes	4.09	10^{12}/L		
				Leucocytes	3.74	10^9/L		
			Haemostasis	APTT	26.4	s		

Sign.: Significant finding; L: Result considered low; H: Result considered high; SC: Screening; BL: Baseline; D: Day; FUP: Follow-up; Cohort A3: Treatment was applied under fasting and fed conditions in the same subjects;

Output generated by program 'NIC002_L16_2_8_Laboratory_V02_0_0'

Listing 16.2.8: Study subject data
Laboratory values

Part A

Cohort	Subject ID	Visit	Type	Measurement	Result		Flagged	Sign.
		FUP	Haemostasis	Prothrombin Time (INR)	1.05	N/A		
				Prothrombin Time (PT)	91.5	%		
			Urine	Beta-HCG, urine	negative			
	22	SC	Clinical Chemistry	ALT, 37 °C	20.0	U/L		
				Alkaline Phosphatase, 37 °C	54.5	U/L		
				AST, 37 °C	29.8	U/L		
				Bicarbonate	28.1	mmol/L		
				Bilirubin, total	14.1	umol/L		
				Urea/BUN	3.43	mmol/L		
				Calcium	2.37	mmol/L		
				Creatinine	67.1	umol/L		
				Glucose, serum	4.66	mmol/L		
				Gamma-GT, 37 °C	10.4	U/L		
				Potassium	4.12	mmol/L		
				Creatinine Clearance MDRD	92	ml/min/1.73m		
				Magnesium	0.80	mmol/L		
				Sodium	138.8	mmol/L		
			Drugs	Amphetamines, Urine	negative			
				Barbiturates, Urine	negative			
				Benzodiazepines, Urine	negative			
				Cannabin., Urine	negative			
				Cocaine, Urine	negative			
				Methadone, Urine	negative			
				Opiates, Urine	negative			
			Haematology	Basophils, abs.	0.05	10^9/L		
				Basophils, %	1.2	%		
				Eosinophils, abs.	0.15	10^9/L		
				Eosinophils, %	3.7	%		
				Haemoglobin	141.0	g/L		
				Haematocrit	0.41	L/L		

Sign.: Significant finding; L: Result considered low; H: Result considered high; SC: Screening; BL: Baseline; D: Day; FUP: Follow-up; Cohort A3: Treatment was applied under fasting and fed conditions in the same subjects;

Output generated by program 'NIC002_L16_2_8_Laboratory_V02_0_0'

Listing 16.2.8: Study subject data
Laboratory values

Part A

Cohort	Subject ID	Visit	Type	Measurement	Result		Flagged	Sign.
	22	SC	Haematology	Lymphocytes, abs.	1.73	10^9/L		
				Lymphocytes, %	42.8	%		
				Monocytes, abs.	0.27	10^9/L		
				Monocytes, %	6.7	%		
				Neutrophils, abs.	1.84	10^9/L		
				Neutrophils, %	45.6	%		
				Platelets	247	10^9/L		
				Erythrocytes	4.60	10^{12}/L		
				Leucocytes	4.04	10^9/L		
			Haemostasis	APTT	24.1	s		
				Prothrombin Time (INR)	0.97	N/A		
				Prothrombin Time (PT)	109.9	%		
			Infectious Diseases	HBs-Ag (Hep. B Surf. Ag)	negative	N/A		
				Anti-HCV (Hep. C-AB)	non-reactive	N/A		
				HIV 1+2, AG/AB	negative	N/A		
			Urine	Bilirubin, urine (Stix)	negative			
				Blood (Ery/Hb), urine (Stix)	negative			
				Glucose, urine (Stix)	negative			
				Beta-HCG, urine	negative			
				Ketone, urine (Stix)	negative			
				Leucocytes, urine (Stix)	negative			
				Nitrite, urine (Stix)	negative			
				pH, urine (Stix)	5.5	neg.log[H+]		
				Protein, total, urine (Stix)	negative			
				Specific Gravity	1.010			
				Urobilinogen, urine (Stix)	0.2	mg/dl		
		BL	Clinical Chemistry	ALT, 37 °C	18.2	U/L		
				Alkaline Phosphatase, 37 °C	51.3	U/L		
				AST, 37 °C	26.6	U/L		
				Bicarbonate	25.6	mmol/L		

Sign.: Significant finding; L: Result considered low; H: Result considered high; SC: Screening; BL: Baseline; D: Day; FUP: Follow-up; Cohort A3: Treatment was applied under fasting and fed conditions in the same subjects;

Output generated by program 'NIC002_L16_2_8_Laboratory_V02_0_0'

Listing 16.2.8: Study subject data
Laboratory values

Part A

Cohort	Subject ID	Visit	Type	Measurement	Result		Flagged	Sign.
		BL	Clinical Chemistry	Bilirubin, total	9.9	umol/L		
				Urea/BUN	3.16	mmol/L		
				Calcium	2.36	mmol/L		
				Creatinine	67.4	umol/L		
				Glucose, serum	5.01	mmol/L		
				Gamma-GT, 37 °C	8.9	U/L		
				Potassium	3.99	mmol/L		
				Magnesium	0.81	mmol/L		
				Sodium	139.7	mmol/L		
			Drugs	Amphetamines, Urine	negative			
				Barbiturates, Urine	negative			
				Benzodiazepines, Urine	negative			
				Cannabin., Urine	negative			
				Cocaine, Urine	negative			
				Methadone, Urine	negative			
				Opiates, Urine	negative			
			Haematology	Basophils, abs.	0.05	10^9/L		
				Basophils, %	1.0	%		
				Eosinophils, abs.	0.14	10^9/L		
				Eosinophils, %	2.8	%		
				Haemoglobin	144.0	g/L		
				Haematocrit	0.41	L/L		
				Lymphocytes, abs.	2.16	10^9/L		
				Lymphocytes, %	43.0	%		
				Monocytes, abs.	0.56	10^9/L		
				Monocytes, %	11.2	%		
				Neutrophils, abs.	2.11	10^9/L		
				Neutrophils, %	42.0	%		
				Platelets	260	10^9/L		
				Erythrocytes	4.64	10^{12}/L		
				Leucocytes	5.02	10^9/L		

Sign.: Significant finding; L: Result considered low; H: Result considered high; SC: Screening; BL: Baseline; D: Day; FUP: Follow-up; Cohort A3: Treatment was applied under fasting and fed conditions in the same subjects;

Output generated by program 'NIC002_L16_2_8_Laboratory_V02_0_0'

Listing 16.2.8: Study subject data
Laboratory values

Part A

Cohort	Subject ID	Visit	Type	Measurement	Result		Flagged	Sign.
		BL	Haemostasis	APTT	24.5	s		
				Prothrombin Time (INR)	0.95	N/A		
				Prothrombin Time (PT)	116.1	%		
			Urine	Beta-HCG, urine	negative			
		D02	Clinical Chemistry	ALT, 37 °C	15.4	U/L		
				Alkaline Phosphatase, 37 °C	48.1	U/L		
				AST, 37 °C	22.6	U/L		
				Bicarbonate	26.0	mmol/L		
				Bilirubin, total	14.1	umol/L		
				Urea/BUN	3.66	mmol/L		
				Calcium	2.34	mmol/L		
				Creatinine	69.3	umol/L		
				Glucose, serum	4.63	mmol/L		
				Gamma-GT, 37 °C	8.3	U/L		
				Potassium	4.08	mmol/L		
				Magnesium	0.79	mmol/L		
				Sodium	137.1	mmol/L		
			Haematology	Basophils, abs.	0.02	10^9/L		
				Basophils, %	0.4	%		
				Eosinophils, abs.	0.13	10^9/L		
				Eosinophils, %	2.8	%		
				Haemoglobin	145.0	g/L		
				Haematocrit	0.41	L/L		
				Lymphocytes, abs.	1.47	10^9/L		
				Lymphocytes, %	31.5	%		
				Monocytes, abs.	0.33	10^9/L		
				Monocytes, %	7.1	%		
				Neutrophils, abs.	2.71	10^9/L		
				Neutrophils, %	58.2	%		
				Platelets	238	10^9/L		

Sign.: Significant finding; L: Result considered low; H: Result considered high; SC: Screening; BL: Baseline; D: Day; FUP: Follow-up; Cohort A3: Treatment was applied under fasting and fed conditions in the same subjects;

Output generated by program 'NIC002_L16_2_8_Laboratory_V02_0_0'

Listing 16.2.8: Study subject data
Laboratory values

Part A

Cohort	Subject ID	Visit	Type	Measurement	Result		Flagged	Sign.
		D02	Haematology	Erythrocytes	4.66	10^{12}/L		
				Leucocytes	4.66	10^{9}/L		
			Haemostasis	APTT	23.9	s		
				Prothrombin Time (INR)	1.00	N/A		
				Prothrombin Time (PT)	101.5	%		
		FUP	Clinical Chemistry	ALT, 37 °C	18.8	U/L		
				Alkaline Phosphatase, 37 °C	50.5	U/L		
				AST, 37 °C	32.7	U/L		
				Bicarbonate	23.1	mmol/L		
				Bilirubin, total	12.5	umol/L		
				Urea/BUN	4.31	mmol/L		
				Calcium	2.27	mmol/L		
				Creatinine	67.6	umol/L		
				Glucose, serum	4.34	mmol/L		
				Gamma-GT, 37 °C	8.1	U/L		
				Potassium	4.02	mmol/L		
				Magnesium	0.86	mmol/L		
				Sodium	138.9	mmol/L		
			Haematology	Basophils, abs.	0.04	10^{9}/L		
				Basophils, %	0.7	%		
				Eosinophils, abs.	0.20	10^{9}/L		
				Eosinophils, %	3.7	%		
				Haemoglobin	135.0	g/L		
				Haematocrit	0.38	L/L		
				Lymphocytes, abs.	2.30	10^{9}/L		
				Lymphocytes, %	43.0	%		
				Monocytes, abs.	0.54	10^{9}/L		
				Monocytes, %	10.1	%		
				Neutrophils, abs.	2.27	10^{9}/L		
				Neutrophils, %	42.5	%		

Sign.: Significant finding; L: Result considered low; H: Result considered high; SC: Screening; BL: Baseline; D: Day; FUP: Follow-up; Cohort A3: Treatment was applied under fasting and fed conditions in the same subjects;

Output generated by program 'NIC002_L16_2_8_Laboratory_V02_0_0'

Listing 16.2.8: Study subject data
Laboratory values

Part A

Cohort	Subject ID	Visit	Type	Measurement	Result		Flagged	Sign.
		FUP	Haematology	Platelets	244	10^9/L		
				Erythrocytes	4.28	10^{12}/L		
				Leucocytes	5.35	10^9/L		
			Haemostasis	APTT	24.6	s		
				Prothrombin Time (INR)	0.93	N/A		
				Prothrombin Time (PT)	122.8	%		
			Urine	Beta-HCG, urine	negative			
Cohort A3 Fast	24	SC	Clinical Chemistry	ALT, 37 °C	13.5	U/L		
				Alkaline Phosphatase, 37 °C	41.2	U/L		
				AST, 37 °C	23.6	U/L		
				Bicarbonate	26.2	mmol/L		
				Bilirubin, total	7.7	umol/L		
				Urea/BUN	4.26	mmol/L		
				Calcium	2.39	mmol/L		
				Creatinine	68.2	umol/L		
				Glucose, serum	4.39	mmol/L		
				Gamma-GT, 37 °C	16.8	U/L		
				Potassium	4.14	mmol/L		
				Creatinine Clearance MDRD	91	ml/min/1.73m		
				Magnesium	0.75	mmol/L	L	No
				Sodium	136.9	mmol/L		
			Drugs	Amphetamines, Urine	negative			
				Barbiturates, Urine	negative			
				Benzodiazepines, Urine	negative			
				Cannabin., Urine	negative			
				Cocaine, Urine	negative			
				Methadone, Urine	negative			
				Opiates, Urine	negative			
			Haematology	Basophils, abs.	0.04	10^9/L		

Sign.: Significant finding; L: Result considered low; H: Result considered high; SC: Screening; BL: Baseline; D: Day; FUP: Follow-up; Cohort A3: Treatment was applied under fasting and fed conditions in the same subjects;

Output generated by program 'NIC002_L16_2_8_Laboratory_V02_0_0'

Listing 16.2.8: Study subject data
Laboratory values

Part A

Cohort	Subject ID	Visit	Type	Measurement	Result		Flagged	Sign.
Cohort A3 Fast	24	SC	Haematology	Basophils, %	0.7	%		
				Eosinophils, abs.	0.15	10^9/L		
				Eosinophils, %	2.5	%		
				Haemoglobin	118.0	g/L		
				Haematocrit	0.35	L/L		
				Lymphocytes, abs.	2.72	10^9/L		
				Lymphocytes, %	45.2	%		
				Monocytes, abs.	0.53	10^9/L		
				Monocytes, %	8.8	%		
				Neutrophils, abs.	2.58	10^9/L		
				Neutrophils, %	42.8	%		
				Platelets	267	10^9/L		
				Erythrocytes	4.04	10^{12}/L		
				Leucocytes	6.02	10^9/L		
			Haemostasis	APTT	23.8	s		
				Prothrombin Time (INR)	0.97	N/A		
				Prothrombin Time (PT)	109.9	%		
			Infectious Diseases	HBs-Ag (Hep. B Surf. Ag)	negative	N/A		
				Anti-HCV (Hep. C-AB)	non-reactive	N/A		
				HIV 1+2, AG/AB	negative	N/A		
			Urine	Bilirubin, urine (Stix)	negative			
				Blood (Ery/Hb), urine (Stix)	negative			
				Glucose, urine (Stix)	negative			
				Beta-HCG, urine	negative			
				Ketone, urine (Stix)	negative			
				Leucocytes, urine (Stix)	1+		H	No
				Nitrite, urine (Stix)	negative			
				pH, urine (Stix)	6.5	neg.log[H+]		
				Protein, total, urine (Stix)	negative			
				Bacteria, Sediment	positive		H	No
				Carbonate, Sediment	negative			

Sign.: Significant finding; L: Result considered low; H: Result considered high; SC: Screening; BL: Baseline; D: Day; FUP: Follow-up; Cohort A3: Treatment was applied under fasting and fed conditions in the same subjects;

Output generated by program 'NIC002_L16_2_8_Laboratory_V02_0_0'

Listing 16.2.8: Study subject data
Laboratory values

Part A

Cohort	Subject ID	Visit	Type	Measurement	Result		Flagged	Sign.
Cohort A3 Fast	24	SC	Urine	Epithelial Cells, Sediment	5	per field		
				Erythrocytes, Sediment	1	per field		
				Casts granul., Sediment	0	per field		
				Casts hyaline, Sediment	0	per field		
				Leucocytes, Sediment	2	per field		
				Oxalate, Sediment	negative			
				Specific Gravity	<=1.005			
				Mucus, Sediment	negative			
				Triple Phosphate, Sediment	negative			
				Urates, Sediment	negative			
				Urobilinogen, urine (Stix)	0.2	mg/dl		
		BL	Clinical Chemistry	ALT, 37 °C	12.2	U/L		
				Alkaline Phosphatase, 37 °C	45.0	U/L		
				AST, 37 °C	24.1	U/L		
				Bicarbonate	25.4	mmol/L		
				Bilirubin, total	14.2	umol/L		
				Urea/BUN	4.06	mmol/L		
				Calcium	2.31	mmol/L		
				Creatinine	86.0	umol/L	H	No
				Glucose, serum	4.13	mmol/L		
				Gamma-GT, 37 °C	16.9	U/L		
				Potassium	4.30	mmol/L		
				Magnesium	0.72	mmol/L	L	No
				Sodium	135.8	mmol/L	L	No
			Drugs	Amphetamines, Urine	negative			
				Barbiturates, Urine	negative			
				Benzodiazepines, Urine	negative			
				Cannabin., Urine	negative			
				Cocaine, Urine	negative			
				Methadone, Urine	negative			

Sign.: Significant finding; L: Result considered low; H: Result considered high; SC: Screening; BL: Baseline; D: Day; FUP: Follow-up; Cohort A3: Treatment was applied under fasting and fed conditions in the same subjects;

Output generated by program 'NIC002_L16_2_8_Laboratory_V02_0_0'

Listing 16.2.8: Study subject data
Laboratory values

Part A

Cohort	Subject ID	Visit	Type	Measurement	Result		Flagged	Sign.
		BL	Drugs	Opiates, Urine	negative			
			Haematology	Basophils, abs.	0.03	10^9/L		
				Basophils, %	0.3	%		
				Eosinophils, abs.	0.10	10^9/L		
				Eosinophils, %	1.0	%		
				Haemoglobin	116.0	g/L		
				Haematocrit	0.34	L/L	L	No
				Lymphocytes, abs.	2.41	10^9/L		
				Lymphocytes, %	24.7	%		
				Monocytes, abs.	0.78	10^9/L		
				Monocytes, %	8.0	%		
				Neutrophils, abs.	6.44	10^9/L		
				Neutrophils, %	66.0	%		
				Platelets	234	10^9/L		
				Erythrocytes	3.92	10^{12}/L	L	No
				Leucocytes	9.76	10^9/L		
			Haemostasis	APTT	24.9	s		
				Prothrombin Time (INR)	1.04	N/A		
				Prothrombin Time (PT)	93.9	%		
			Urine	Beta-HCG, urine	negative			
		D02	Clinical Chemistry	ALT, 37 °C	11.8	U/L		
				Alkaline Phosphatase, 37 °C	51.4	U/L		
				AST, 37 °C	26.9	U/L		
				Bicarbonate	24.9	mmol/L		
				Bilirubin, total	6.6	umol/L		
				Urea/BUN	3.73	mmol/L		
				Calcium	2.49	mmol/L		
				Creatinine	77.7	umol/L		
				Glucose, serum	4.26	mmol/L		
				Gamma-GT, 37 °C	21.0	U/L		

Sign.: Significant finding; L: Result considered low; H: Result considered high; SC: Screening; BL: Baseline; D: Day; FUP: Follow-up; Cohort A3: Treatment was applied under fasting and fed conditions in the same subjects;

Output generated by program 'NIC002_L16_2_8_Laboratory_V02_0_0'

Listing 16.2.8: Study subject data
Laboratory values

Part A

Cohort	Subject ID	Visit	Type	Measurement	Result		Flagged	Sign.
		D02	Clinical Chemistry	Potassium	4.35	mmol/L		
				Magnesium	0.87	mmol/L		
				Sodium	140.3	mmol/L		
			Haematology	Basophils, abs.	0.05	10^9/L		
				Basophils, %	0.7	%		
				Eosinophils, abs.	0.08	10^9/L		
				Eosinophils, %	1.2	%		
				Haemoglobin	130.0	g/L		
				Haematocrit	0.39	L/L		
				Lymphocytes, abs.	2.34	10^9/L		
				Lymphocytes, %	34.1	%		
				Monocytes, abs.	0.44	10^9/L		
				Monocytes, %	6.4	%		
				Neutrophils, abs.	3.96	10^9/L		
				Neutrophils, %	57.6	%		
				Platelets	268	10^9/L		
				Erythrocytes	4.45	10^{12}/L		
				Leucocytes	6.87	10^9/L		
			Haemostasis	APTT	23.5	s		
				Prothrombin Time (INR)	0.98	N/A		
				Prothrombin Time (PT)	107.0	%		
		FUP	Clinical Chemistry	ALT, 37 °C	11.0	U/L		
				Alkaline Phosphatase, 37 °C	50.1	U/L		
				AST, 37 °C	23.1	U/L		
				Bicarbonate	26.6	mmol/L		
				Bilirubin, total	6.9	umol/L		
				Urea/BUN	3.92	mmol/L		
				Calcium	2.38	mmol/L		
				Creatinine	72.1	umol/L		
				Glucose, serum	4.30	mmol/L		

Sign.: Significant finding; L: Result considered low; H: Result considered high; SC: Screening; BL: Baseline; D: Day; FUP: Follow-up; Cohort A3: Treatment was applied under fasting and fed conditions in the same subjects;

Output generated by program 'NIC002_L16_2_8_Laboratory_V02_0_0'

Listing 16.2.8: Study subject data
Laboratory values

Part A

Cohort	Subject ID	Visit	Type	Measurement	Result		Flagged	Sign.
		FUP	Clinical Chemistry	Gamma-GT, 37 °C	19.1	U/L		
				Potassium	4.36	mmol/L		
				Magnesium	0.79	mmol/L		
				Sodium	137.4	mmol/L		
			Haematology	Basophils, abs.	0.04	10^9/L		
				Basophils, %	0.7	%		
				Eosinophils, abs.	0.09	10^9/L		
				Eosinophils, %	1.5	%		
				Haemoglobin	118.0	g/L		
				Haematocrit	0.35	L/L		
				Lymphocytes, abs.	2.54	10^9/L		
				Lymphocytes, %	42.9	%		
				Monocytes, abs.	0.42	10^9/L		
				Monocytes, %	7.1	%		
				Neutrophils, abs.	2.83	10^9/L		
				Neutrophils, %	47.8	%		
				Platelets	260	10^9/L		
				Erythrocytes	4.08	10^{12}/L		
				Leucocytes	5.92	10^9/L		
			Haemostasis	APTT	24.6	s		
				Prothrombin Time (INR)	0.98	N/A		
				Prothrombin Time (PT)	107.0	%		
			Urine	Beta-HCG, urine	negative			
	25	SC	Clinical Chemistry	ALT, 37 °C	10.1	U/L		
				Alkaline Phosphatase, 37 °C	52.7	U/L		
				AST, 37 °C	21.3	U/L		
				Bicarbonate	25.9	mmol/L		
				Bilirubin, total	13.1	umol/L		
				Urea/BUN	3.17	mmol/L		
				Calcium	2.40	mmol/L		

Sign.: Significant finding; L: Result considered low; H: Result considered high; SC: Screening; BL: Baseline; D: Day; FUP: Follow-up; Cohort A3: Treatment was applied under fasting and fed conditions in the same subjects;

Output generated by program 'NIC002_L16_2_8_Laboratory_V02_0_0'

Listing 16.2.8: Study subject data
Laboratory values

Part A

Cohort	Subject ID	Visit	Type	Measurement	Result		Flagged	Sign.
	25	SC	Clinical Chemistry	Creatinine	56.3	umol/L		
				Glucose, serum	5.29	mmol/L		
				Gamma-GT, 37 °C	7.9	U/L		
				Potassium	4.09	mmol/L		
				Creatinine Clearance MDRD	115	ml/min/1.73m		
				Magnesium	0.78	mmol/L		
				Sodium	137.6	mmol/L		
			Drugs	Amphetamines, Urine	negative			
				Barbiturates, Urine	negative			
				Benzodiazepines, Urine	negative			
				Cannabin., Urine	negative			
				Cocaine, Urine	negative			
				Methadone, Urine	negative			
				Opiates, Urine	negative			
			Haematology	Basophils, abs.	0.02	10^9/L		
				Basophils, %	0.4	%		
				Eosinophils, abs.	0.08	10^9/L		
				Eosinophils, %	1.8	%		
				Haemoglobin	124.0	g/L		
				Haematocrit	0.37	L/L		
				Lymphocytes, abs.	1.83	10^9/L		
				Lymphocytes, %	40.8	%		
				Monocytes, abs.	0.42	10^9/L		
				Monocytes, %	9.4	%		
				Neutrophils, abs.	2.14	10^9/L		
				Neutrophils, %	47.6	%		
				Platelets	225	10^9/L		
				Erythrocytes	4.39	10^{12}/L		
				Leucocytes	4.49	10^9/L		
			Haemostasis	APTT	23.5	s		
				Prothrombin Time (INR)	1.00	N/A		

Sign.: Significant finding; L: Result considered low; H: Result considered high; SC: Screening; BL: Baseline; D: Day; FUP: Follow-up; Cohort A3: Treatment was applied under fasting and fed conditions in the same subjects;

Output generated by program 'NIC002_L16_2_8_Laboratory_V02_0_0'

Listing 16.2.8: Study subject data
Laboratory values

Part A

Cohort	Subject ID	Visit	Type	Measurement	Result		Flagged	Sign.
	25	SC	Haemostasis	Prothrombin Time (PT)	101.5	%		
			Infectious Diseases	HBs-Ag (Hep. B Surf. Ag)	negative	N/A		
				Anti-HCV (Hep. C-AB)	non-reactive	N/A		
				HIV 1+2, AG/AB	negative	N/A		
			Urine	Bilirubin, urine (Stix)	negative			
				Blood (Ery/Hb), urine (Stix)	negative			
				Glucose, urine (Stix)	negative			
				Beta-HCG, urine	negative			
				Ketone, urine (Stix)	negative			
				Leucocytes, urine (Stix)	negative			
				Nitrite, urine (Stix)	negative			
				pH, urine (Stix)	6.0	neg.log[H+]		
				Protein, total, urine (Stix)	negative			
				Bacteria, Sediment	negative			
				Carbonate, Sediment	negative			
				Epithelial Cells, Sediment	8	per field		
				Erythrocytes, Sediment	2	per field	H	No
				Casts granul., Sediment	0	per field		
				Casts hyaline, Sediment	0	per field		
				Leucocytes, Sediment	3	per field		
				Oxalate, Sediment	negative			
				Specific Gravity	<=1.005			
				Mucus, Sediment	negative			
				Triple Phosphate, Sediment	negative			
				Urates, Sediment	negative			
				Urobilinogen, urine (Stix)	0.2	mg/dl		
		BL	Clinical Chemistry	ALT, 37 °C	10.3	U/L		
				Alkaline Phosphatase, 37 °C	55.7	U/L		
				AST, 37 °C	20.1	U/L		
				Bicarbonate	24.1	mmol/L		

Sign.: Significant finding; L: Result considered low; H: Result considered high; SC: Screening; BL: Baseline; D: Day; FUP: Follow-up; Cohort A3: Treatment was applied under fasting and fed conditions in the same subjects;

Output generated by program 'NIC002_L16_2_8_Laboratory_V02_0_0'

Listing 16.2.8: Study subject data
Laboratory values

Part A

Cohort	Subject ID	Visit	Type	Measurement	Result		Flagged	Sign.
		BL	Clinical Chemistry	Bilirubin, total	7.3	umol/L		
				Urea/BUN	5.38	mmol/L		
				Calcium	2.38	mmol/L		
				Creatinine	56.2	umol/L		
				Glucose, serum	5.12	mmol/L		
				Gamma-GT, 37 °C	8.8	U/L		
				Potassium	4.29	mmol/L		
				Magnesium	0.80	mmol/L		
				Sodium	137.2	mmol/L		
			Drugs	Amphetamines, Urine	negative			
				Barbiturates, Urine	negative			
				Benzodiazepines, Urine	negative			
				Cannabin., Urine	negative			
				Cocaine, Urine	negative			
				Methadone, Urine	negative			
				Opiates, Urine	negative			
			Haematology	Basophils, abs.	0.02	10^9/L		
				Basophils, %	0.4	%		
				Eosinophils, abs.	0.10	10^9/L		
				Eosinophils, %	2.1	%		
				Haemoglobin	126.0	g/L		
				Haematocrit	0.37	L/L		
				Lymphocytes, abs.	2.01	10^9/L		
				Lymphocytes, %	41.7	%		
				Monocytes, abs.	0.44	10^9/L		
				Monocytes, %	9.1	%		
				Neutrophils, abs.	2.25	10^9/L		
				Neutrophils, %	46.7	%		
				Platelets	271	10^9/L		
				Erythrocytes	4.44	10^{12}/L		
				Leucocytes	4.82	10^9/L		

Sign.: Significant finding; L: Result considered low; H: Result considered high; SC: Screening; BL: Baseline; D: Day; FUP: Follow-up; Cohort A3: Treatment was applied under fasting and fed conditions in the same subjects;

Output generated by program 'NIC002_L16_2_8_Laboratory_V02_0_0'

Listing 16.2.8: Study subject data
Laboratory values

Part A

Cohort	Subject ID	Visit	Type	Measurement	Result		Flagged	Sign.
		BL	Haemostasis	APTT	24.6	s		
				Prothrombin Time (INR)	1.02	N/A		
				Prothrombin Time (PT)	98.8	%		
			Urine	Beta-HCG, urine	negative			
		D02	Clinical Chemistry	ALT, 37 °C	10.0	U/L		
				Alkaline Phosphatase, 37 °C	64.3	U/L		
				AST, 37 °C	19.2	U/L		
				Bicarbonate	29.1	mmol/L		
				Bilirubin, total	6.7	umol/L		
				Urea/BUN	4.09	mmol/L		
				Calcium	2.39	mmol/L		
				Creatinine	55.4	umol/L		
				Glucose, serum	5.02	mmol/L		
				Gamma-GT, 37 °C	8.7	U/L		
				Potassium	4.44	mmol/L		
				Magnesium	0.79	mmol/L		
				Sodium	137.0	mmol/L		
			Haematology	Basophils, abs.	0.02	10^9/L		
				Basophils, %	0.4	%		
				Eosinophils, abs.	0.07	10^9/L		
				Eosinophils, %	1.3	%		
				Haemoglobin	125.0	g/L		
				Haematocrit	0.37	L/L		
				Lymphocytes, abs.	1.99	10^9/L		
				Lymphocytes, %	36.5	%		
				Monocytes, abs.	0.51	10^9/L		
				Monocytes, %	9.4	%		
				Neutrophils, abs.	2.86	10^9/L		
				Neutrophils, %	52.4	%		
				Platelets	261	10^9/L		

Sign.: Significant finding; L: Result considered low; H: Result considered high; SC: Screening; BL: Baseline; D: Day; FUP: Follow-up; Cohort A3: Treatment was applied under fasting and fed conditions in the same subjects;

Output generated by program 'NIC002_L16_2_8_Laboratory_V02_0_0'

Listing 16.2.8: Study subject data
Laboratory values

Part A

Cohort	Subject ID	Visit	Type	Measurement	Result		Flagged	Sign.
		D02	Haematology	Erythrocytes	4.39	10^{12}/L		
				Leucocytes	5.45	10^{9}/L		
			Haemostasis	APTT	24.7	s		
				Prothrombin Time (INR)	1.05	N/A		
				Prothrombin Time (PT)	91.5	%		
		FUP	Clinical Chemistry	ALT, 37 °C	13.0	U/L		
				Alkaline Phosphatase, 37 °C	59.5	U/L		
				AST, 37 °C	21.8	U/L		
				Bicarbonate	26.9	mmol/L		
				Bilirubin, total	6.9	umol/L		
				Urea/BUN	4.98	mmol/L		
				Calcium	2.33	mmol/L		
				Creatinine	58.0	umol/L		
				Glucose, serum	4.52	mmol/L		
				Gamma-GT, 37 °C	8.5	U/L		
				Potassium	4.19	mmol/L		
				Magnesium	0.80	mmol/L		
				Sodium	136.4	mmol/L		
			Haematology	Basophils, abs.	0.03	10^{9}/L		
				Basophils, %	0.6	%		
				Eosinophils, abs.	0.05	10^{9}/L		
				Eosinophils, %	1.1	%		
				Haemoglobin	120.0	g/L		
				Haematocrit	0.35	L/L		
				Lymphocytes, abs.	2.00	10^{9}/L		
				Lymphocytes, %	42.3	%		
				Monocytes, abs.	0.51	10^{9}/L		
				Monocytes, %	10.8	%		
				Neutrophils, abs.	2.14	10^{9}/L		
				Neutrophils, %	45.2	%		

Sign.: Significant finding; L: Result considered low; H: Result considered high; SC: Screening; BL: Baseline; D: Day; FUP: Follow-up; Cohort A3: Treatment was applied under fasting and fed conditions in the same subjects;

Output generated by program 'NIC002_L16_2_8_Laboratory_V02_0_0'

Listing 16.2.8: Study subject data
Laboratory values

Part A

Cohort	Subject ID	Visit	Type	Measurement	Result		Flagged	Sign.
		FUP	Haematology	Platelets	244	10^9/L		
				Erythrocytes	4.18	10^{12}/L		
				Leucocytes	4.73	10^9/L		
			Haemostasis	APTT	25.1	s		
				Prothrombin Time (INR)	1.03	N/A		
				Prothrombin Time (PT)	96.3	%		
			Urine	Beta-HCG, urine	negative			
	27	SC	Clinical Chemistry	ALT, 37 °C	24.1	U/L		
				Alkaline Phosphatase, 37 °C	50.9	U/L		
				AST, 37 °C	30.9	U/L		
				Bicarbonate	27.4	mmol/L		
				Bilirubin, total	11.8	umol/L		
				Urea/BUN	3.25	mmol/L		
				Calcium	2.34	mmol/L		
				Creatinine	58.9	umol/L		
				Glucose, serum	4.16	mmol/L		
				Gamma-GT, 37 °C	14.8	U/L		
				Potassium	4.20	mmol/L		
				Creatinine Clearance MDRD	100	ml/min/1.73m		
				Magnesium	0.82	mmol/L		
				Sodium	136.8	mmol/L		
			Drugs	Amphetamines, Urine	negative			
				Barbiturates, Urine	negative			
				Benzodiazepines, Urine	negative			
				Cannabin., Urine	negative			
				Cocaine, Urine	negative			
				Methadone, Urine	negative			
				Opiates, Urine	negative			
			Haematology	Basophils, abs.	0.06	10^9/L		
				Basophils, %	0.8	%		

Sign.: Significant finding; L: Result considered low; H: Result considered high; SC: Screening; BL: Baseline; D: Day; FUP: Follow-up; Cohort A3: Treatment was applied under fasting and fed conditions in the same subjects;

Output generated by program 'NIC002_L16_2_8_Laboratory_V02_0_0'

Listing 16.2.8: Study subject data
Laboratory values

Part A

Cohort	Subject ID	Visit	Type	Measurement	Result		Flagged	Sign.
	27	SC	Haematology	Eosinophils, abs.	0.35	10^9/L		
				Eosinophils, %	4.9	%		
				Haemoglobin	123.0	g/L		
				Haematocrit	0.37	L/L		
				Lymphocytes, abs.	2.17	10^9/L		
				Lymphocytes, %	30.5	%		
				Monocytes, abs.	0.59	10^9/L		
				Monocytes, %	8.3	%		
				Neutrophils, abs.	3.94	10^9/L		
				Neutrophils, %	55.5	%		
				Platelets	268	10^9/L		
				Erythrocytes	4.12	10^{12}/L		
				Leucocytes	7.11	10^9/L		
			Haemostasis	APTT	23.8	s		
				Prothrombin Time (INR)	1.13	N/A		
				Prothrombin Time (PT)	75.2	%		
			Infectious Diseases	HBs-Ag (Hep. B Surf. Ag)	negative	N/A		
				Anti-HCV (Hep. C-AB)	non-reactive	N/A		
				HIV 1+2, AG/AB	negative	N/A		
			Urine	Bilirubin, urine (Stix)	negative			
				Blood (Ery/Hb), urine (Stix)	negative			
				Glucose, urine (Stix)	negative			
				Beta-HCG, urine	negative			
				Ketone, urine (Stix)	negative			
				Leucocytes, urine (Stix)	negative			
				Nitrite, urine (Stix)	negative			
				pH, urine (Stix)	8.0	neg.log[H+]		
				Protein, total, urine (Stix)	negative			
				Specific Gravity	1.015			
				Urobilinogen, urine (Stix)	0.2	mg/dl		

Sign.: Significant finding; L: Result considered low; H: Result considered high; SC: Screening; BL: Baseline; D: Day; FUP: Follow-up; Cohort A3: Treatment was applied under fasting and fed conditions in the same subjects;

Output generated by program 'NIC002_L16_2_8_Laboratory_V02_0_0'

Listing 16.2.8: Study subject data
Laboratory values

Part A

Cohort	Subject ID	Visit	Type	Measurement	Result		Flagged	Sign.
		BL	Clinical Chemistry	ALT, 37 °C	12.0	U/L		
				Alkaline Phosphatase, 37 °C	50.8	U/L		
				AST, 37 °C	21.9	U/L		
				Bicarbonate	29.3	mmol/L		
				Bilirubin, total	12.1	umol/L		
				Urea/BUN	3.04	mmol/L		
				Calcium	2.38	mmol/L		
				Creatinine	59.3	umol/L		
				Glucose, serum	4.79	mmol/L		
				Gamma-GT, 37 °C	14.6	U/L		
				Potassium	4.15	mmol/L		
				Magnesium	0.82	mmol/L		
				Sodium	139.3	mmol/L		
			Drugs	Amphetamines, Urine	negative			
				Barbiturates, Urine	negative			
				Benzodiazepines, Urine	negative			
				Cannabin., Urine	negative			
				Cocaine, Urine	negative			
				Methadone, Urine	negative			
				Opiates, Urine	negative			
			Haematology	Basophils, abs.	0.06	10^9/L		
				Basophils, %	0.9	%		
				Eosinophils, abs.	0.38	10^9/L		
				Eosinophils, %	5.5	%		
				Haemoglobin	124.0	g/L		
				Haematocrit	0.37	L/L		
				Lymphocytes, abs.	1.78	10^9/L		
				Lymphocytes, %	26.0	%		
				Monocytes, abs.	0.46	10^9/L		
				Monocytes, %	6.7	%		
				Neutrophils, abs.	4.17	10^9/L		

Sign.: Significant finding; L: Result considered low; H: Result considered high; SC: Screening; BL: Baseline; D: Day; FUP: Follow-up; Cohort A3: Treatment was applied under fasting and fed conditions in the same subjects;

Output generated by program 'NIC002_L16_2_8_Laboratory_V02_0_0'

Listing 16.2.8: Study subject data
Laboratory values

Part A

Cohort	Subject ID	Visit	Type	Measurement	Result		Flagged	Sign.
		BL	Haematology	Neutrophils, %	60.9	%		
				Platelets	288	10^9/L		
				Erythrocytes	4.17	10^{12}/L		
				Leucocytes	6.85	10^9/L		
			Haemostasis	APTT	23.7	s		
				Prothrombin Time (INR)	1.12	N/A		
				Prothrombin Time (PT)	77.0	%		
			Urine	Beta-HCG, urine	negative			
		D02	Clinical Chemistry	ALT, 37 °C	15.7	U/L		
				Alkaline Phosphatase, 37 °C	56.9	U/L		
				AST, 37 °C	27.0	U/L		
				Bicarbonate	28.8	mmol/L		
				Bilirubin, total	12.6	umol/L		
				Urea/BUN	3.26	mmol/L		
				Calcium	2.38	mmol/L		
				Creatinine	56.7	umol/L		
				Glucose, serum	4.60	mmol/L		
				Gamma-GT, 37 °C	12.9	U/L		
				Potassium	4.50	mmol/L		
				Magnesium	0.82	mmol/L		
				Sodium	137.9	mmol/L		
			Haematology	Basophils, abs.	0.04	10^9/L		
				Basophils, %	0.5	%		
				Eosinophils, abs.	0.43	10^9/L		
				Eosinophils, %	5.8	%		
				Haemoglobin	124.0	g/L		
				Haematocrit	0.37	L/L		
				Lymphocytes, abs.	1.95	10^9/L		
				Lymphocytes, %	26.5	%		
				Monocytes, abs.	0.53	10^9/L		

Sign.: Significant finding; L: Result considered low; H: Result considered high; SC: Screening; BL: Baseline; D: Day; FUP: Follow-up; Cohort A3: Treatment was applied under fasting and fed conditions in the same subjects;

Output generated by program 'NIC002_L16_2_8_Laboratory_V02_0_0'

Listing 16.2.8: Study subject data
Laboratory values

Part A

Cohort	Subject ID	Visit	Type	Measurement	Result		Flagged	Sign.
		D02	Haematology	Monocytes, %	7.2	%		
				Neutrophils, abs.	4.41	10^9/L		
				Neutrophils, %	60.0	%		
				Platelets	277	10^9/L		
				Erythrocytes	4.18	10^{12}/L		
				Leucocytes	7.36	10^9/L		
			Haemostasis	APTT	24.0	s		
				Prothrombin Time (INR)	1.18	N/A	H	No
				Prothrombin Time (PT)	68.5	%	L	No
		FUP	Clinical Chemistry	ALT, 37 °C	17.6	U/L		
				Alkaline Phosphatase, 37 °C	56.5	U/L		
				AST, 37 °C	26.7	U/L		
				Bicarbonate	30.9	mmol/L		
				Bilirubin, total	11.1	umol/L		
				Urea/BUN	2.85	mmol/L		
				Calcium	2.40	mmol/L		
				Creatinine	55.4	umol/L		
				Glucose, serum	4.34	mmol/L		
				Gamma-GT, 37 °C	13.9	U/L		
				Potassium	4.51	mmol/L		
				Magnesium	0.82	mmol/L		
				Sodium	136.6	mmol/L		
			Haematology	Basophils, abs.	0.06	10^9/L		
				Basophils, %	0.8	%		
				Eosinophils, abs.	0.46	10^9/L	H	No
				Eosinophils, %	6.0	%		
				Haemoglobin	127.0	g/L		
				Haematocrit	0.38	L/L		
				Lymphocytes, abs.	1.97	10^9/L		
				Lymphocytes, %	25.8	%		

Sign.: Significant finding; L: Result considered low; H: Result considered high; SC: Screening; BL: Baseline; D: Day; FUP: Follow-up; Cohort A3: Treatment was applied under fasting and fed conditions in the same subjects;

Output generated by program 'NIC002_L16_2_8_Laboratory_V02_0_0'

Listing 16.2.8: Study subject data
Laboratory values

Part A

Cohort	Subject ID	Visit	Type	Measurement	Result		Flagged	Sign.
		FUP	Haematology	Monocytes, abs.	0.50	10^9/L		
				Monocytes, %	6.5	%		
				Neutrophils, abs.	4.65	10^9/L		
				Neutrophils, %	60.9	%		
				Platelets	265	10^9/L		
				Erythrocytes	4.26	10^{12}/L		
				Leucocytes	7.64	10^9/L		
			Haemostasis	APTT	24.0	s		
				Prothrombin Time (INR)	1.13	N/A		
				Prothrombin Time (PT)	75.2	%		
			Urine	Beta-HCG, urine	negative			
	28	SC	Clinical Chemistry	ALT, 37 °C	9.6	U/L		
				Alkaline Phosphatase, 37 °C	59.3	U/L		
				AST, 37 °C	15.8	U/L		
				Bicarbonate	28.9	mmol/L		
				Bilirubin, total	8.2	umol/L		
				Urea/BUN	2.43	mmol/L	L	No
				Calcium	2.36	mmol/L		
				Creatinine	58.3	umol/L		
				Glucose, serum	4.52	mmol/L		
				Gamma-GT, 37 °C	10.3	U/L		
				Potassium	3.66	mmol/L		
				Creatinine Clearance MDRD	113	ml/min/1.73m		
				Magnesium	0.72	mmol/L	L	No
				Sodium	139.3	mmol/L		
			Drugs	Amphetamines, Urine	negative			
				Barbiturates, Urine	negative			
				Benzodiazepines, Urine	negative			
				Cannabin., Urine	negative			
				Cocaine, Urine	negative			

Sign.: Significant finding; L: Result considered low; H: Result considered high; SC: Screening; BL: Baseline; D: Day; FUP: Follow-up; Cohort A3: Treatment was applied under fasting and fed conditions in the same subjects;

Output generated by program 'NIC002_L16_2_8_Laboratory_V02_0_0'

Listing 16.2.8: Study subject data
Laboratory values

Part A

Cohort	Subject ID	Visit	Type	Measurement	Result		Flagged	Sign.
	28	SC	Drugs	Methadone, Urine	negative			
				Opiates, Urine	negative			
			Haematology	Basophils, abs.	0.05	10^9/L		
				Basophils, %	1.1	%		
				Eosinophils, abs.	0.23	10^9/L		
				Eosinophils, %	5.1	%		
				Haemoglobin	116.0	g/L		
				Haematocrit	0.32	L/L	L	No
				Lymphocytes, abs.	1.35	10^9/L		
				Lymphocytes, %	29.7	%		
				Monocytes, abs.	0.28	10^9/L		
				Monocytes, %	6.2	%		
				Neutrophils, abs.	2.63	10^9/L		
				Neutrophils, %	57.9	%		
				Platelets	333	10^9/L		
				Erythrocytes	3.73	10^{12}/L	L	No
				Leucocytes	4.54	10^9/L		
			Haemostasis	APTT	26.9	s		
				Prothrombin Time (INR)	1.07	N/A		
				Prothrombin Time (PT)	87.0	%		
			Infectious Diseases	HBs-Ag (Hep. B Surf. Ag)	negative	N/A		
				Anti-HCV (Hep. C-AB)	non-reactive	N/A		
				HIV 1+2, AG/AB	negative	N/A		
			Urine	Bilirubin, urine (Stix)	negative			
				Blood (Ery/Hb), urine (Stix)	1+		H	No
				Glucose, urine (Stix)	negative			
				Beta-HCG, urine	negative			
				Ketone, urine (Stix)	negative			
				Leucocytes, urine (Stix)	negative			
				Nitrite, urine (Stix)	negative			
				pH, urine (Stix)	6.5	neg.log[H+]		

Sign.: Significant finding; L: Result considered low; H: Result considered high; SC: Screening; BL: Baseline; D: Day; FUP: Follow-up; Cohort A3: Treatment was applied under fasting and fed conditions in the same subjects;

Output generated by program 'NIC002_L16_2_8_Laboratory_V02_0_0'

Listing 16.2.8: Study subject data
Laboratory values

Part A

Cohort	Subject ID	Visit	Type	Measurement	Result		Flagged	Sign.
	28	SC	Urine	Protein, total, urine (Stix)	negative			
				Bacteria, Sediment	positive		H	No
				Carbonate, Sediment	negative			
				Epithelial Cells, Sediment	8	per field		
				Erythrocytes, Sediment	2	per field	H	No
				Casts granul., Sediment	0	per field		
				Casts hyaline, Sediment	0	per field		
				Leucocytes, Sediment	3	per field		
				Oxalate, Sediment	negative			
				Specific Gravity	<=1.005			
				Mucus, Sediment	negative			
				Triple Phosphate, Sediment	negative			
				Urates, Sediment	negative			
				Urobilinogen, urine (Stix)	0.2	mg/dl		
		BL	Clinical Chemistry	ALT, 37 °C	15.7	U/L		
				Alkaline Phosphatase, 37 °C	61.1	U/L		
				AST, 37 °C	19.0	U/L		
				Bicarbonate	27.6	mmol/L		
				Bilirubin, total	8.8	umol/L		
				Urea/BUN	1.82	mmol/L	L	No
				Calcium	2.53	mmol/L		
				Creatinine	58.6	umol/L		
				Glucose, serum	4.79	mmol/L		
				Gamma-GT, 37 °C	11.2	U/L		
				Potassium	4.50	mmol/L		
				Magnesium	0.76	mmol/L	L	No
				Sodium	140.0	mmol/L		
			Drugs	Amphetamines, Urine	negative			
				Barbiturates, Urine	negative			
				Benzodiazepines, Urine	negative			

Sign.: Significant finding; L: Result considered low; H: Result considered high; SC: Screening; BL: Baseline; D: Day; FUP: Follow-up; Cohort A3: Treatment was applied under fasting and fed conditions in the same subjects;

Output generated by program 'NIC002_L16_2_8_Laboratory_V02_0_0'

Listing 16.2.8: Study subject data
Laboratory values

Part A

Cohort	Subject ID	Visit	Type	Measurement	Result		Flagged	Sign.
		BL	Drugs	Cannabin., Urine	negative			
				Cocaine, Urine	negative			
				Methadone, Urine	negative			
				Opiates, Urine	negative			
			Haematology	Basophils, abs.	0.03	10^9/L		
				Basophils, %	0.6	%		
				Eosinophils, abs.	0.14	10^9/L		
				Eosinophils, %	2.8	%		
				Haemoglobin	123.0	g/L		
				Haematocrit	0.34	L/L	L	No
				Lymphocytes, abs.	1.20	10^9/L		
				Lymphocytes, %	24.4	%		
				Monocytes, abs.	0.33	10^9/L		
				Monocytes, %	6.7	%		
				Neutrophils, abs.	3.22	10^9/L		
				Neutrophils, %	65.5	%		
				Platelets	329	10^9/L		
				Erythrocytes	3.95	10^{12}/L	L	No
				Leucocytes	4.92	10^9/L		
			Haemostasis	APTT	26.8	s		
				Prothrombin Time (INR)	1.09	N/A		
				Prothrombin Time (PT)	82.8	%		
			Urine	Beta-HCG, urine	negative			
		D02	Clinical Chemistry	ALT, 37 °C	12.0	U/L		
				Alkaline Phosphatase, 37 °C	65.0	U/L		
				AST, 37 °C	15.7	U/L		
				Bicarbonate	25.6	mmol/L		
				Bilirubin, total	9.8	umol/L		
				Urea/BUN	2.27	mmol/L	L	No
				Calcium	2.40	mmol/L		

Sign.: Significant finding; L: Result considered low; H: Result considered high; SC: Screening; BL: Baseline; D: Day; FUP: Follow-up; Cohort A3: Treatment was applied under fasting and fed conditions in the same subjects;

Output generated by program 'NIC002_L16_2_8_Laboratory_V02_0_0'

Listing 16.2.8: Study subject data
Laboratory values

Part A

Cohort	Subject ID	Visit	Type	Measurement	Result		Flagged	Sign.
		D02	Clinical Chemistry	Creatinine	59.9	umol/L		
				Glucose, serum	4.98	mmol/L		
				Gamma-GT, 37 °C	10.5	U/L		
				Potassium	4.22	mmol/L		
				Magnesium	0.71	mmol/L	L	No
				Sodium	137.6	mmol/L		
			Haematology	Basophils, abs.	0.04	10^9/L		
				Basophils, %	0.9	%		
				Eosinophils, abs.	0.13	10^9/L		
				Eosinophils, %	2.9	%		
				Haemoglobin	115.0	g/L		
				Haematocrit	0.32	L/L	L	No
				Lymphocytes, abs.	1.49	10^9/L		
				Lymphocytes, %	32.8	%		
				Monocytes, abs.	0.30	10^9/L		
				Monocytes, %	6.6	%		
				Neutrophils, abs.	2.58	10^9/L		
				Neutrophils, %	56.8	%		
				Platelets	324	10^9/L		
				Erythrocytes	3.70	10^{12}/L	L	No
				Leucocytes	4.54	10^9/L		
			Haemostasis	APTT	27.4	s		
				Prothrombin Time (INR)	1.15	N/A		
				Prothrombin Time (PT)	71.8	%		
		FUP	Clinical Chemistry	ALT, 37 °C	10.8	U/L		
				Alkaline Phosphatase, 37 °C	59.2	U/L		
				AST, 37 °C	16.2	U/L		
				Bicarbonate	27.7	mmol/L		
				Bilirubin, total	12.9	umol/L		
				Urea/BUN	2.51	mmol/L	L	No

Sign.: Significant finding; L: Result considered low; H: Result considered high; SC: Screening; BL: Baseline; D: Day; FUP: Follow-up; Cohort A3: Treatment was applied under fasting and fed conditions in the same subjects;

Output generated by program 'NIC002_L16_2_8_Laboratory_V02_0_0'

Listing 16.2.8: Study subject data
Laboratory values

Part A

Cohort	Subject ID	Visit	Type	Measurement	Result		Flagged	Sign.
		FUP	Clinical Chemistry	Calcium	2.34	mmol/L		
				Creatinine	56.7	umol/L		
				Glucose, serum	4.49	mmol/L		
				Gamma-GT, 37 °C	10.5	U/L		
				Potassium	3.94	mmol/L		
				Magnesium	0.70	mmol/L	L	No
				Sodium	137.4	mmol/L		
			Haematology	Basophils, abs.	0.05	10^9/L		
				Basophils, %	1.3	%		
				Eosinophils, abs.	0.14	10^9/L		
				Eosinophils, %	3.5	%		
				Haemoglobin	108.0	g/L	L	No
				Haematocrit	0.30	L/L	L	No
				Lymphocytes, abs.	1.13	10^9/L		
				Lymphocytes, %	28.5	%		
				Monocytes, abs.	0.27	10^9/L		
				Monocytes, %	6.8	%		
				Neutrophils, abs.	2.37	10^9/L		
				Neutrophils, %	59.9	%		
				Platelets	329	10^9/L		
				Erythrocytes	3.52	10^{12}/L	L	No
				Leucocytes	3.96	10^9/L		
			Haemostasis	APTT	26.7	s		
				Prothrombin Time (INR)	1.09	N/A		
				Prothrombin Time (PT)	82.8	%		
			Urine	Beta-HCG, urine	negative			
Cohort A3 Fed	24	BL	Clinical Chemistry	ALT, 37 °C	9.4	U/L		
				Alkaline Phosphatase, 37 °C	45.8	U/L		
				AST, 37 °C	21.5	U/L		

Sign.: Significant finding; L: Result considered low; H: Result considered high; SC: Screening; BL: Baseline; D: Day; FUP: Follow-up; Cohort A3: Treatment was applied under fasting and fed conditions in the same subjects;

Output generated by program 'NIC002_L16_2_8_Laboratory_V02_0_0'

Listing 16.2.8: Study subject data
Laboratory values

Part A

Cohort	Subject ID	Visit	Type	Measurement	Result		Flagged	Sign.
Cohort A3 Fed	24	BL	Clinical Chemistry	Bicarbonate	23.1	mmol/L		
				Bilirubin, total	7.2	umol/L		
				Urea/BUN	4.44	mmol/L		
				Calcium	2.34	mmol/L		
				Creatinine	70.9	umol/L		
				Glucose, serum	4.19	mmol/L		
				Gamma-GT, 37 °C	17.1	U/L		
				Potassium	3.92	mmol/L		
				Magnesium	0.80	mmol/L		
				Sodium	135.9	mmol/L	L	No
			Drugs	Amphetamines, Urine	negative			
				Barbiturates, Urine	negative			
				Benzodiazepines, Urine	negative			
				Cannabin., Urine	negative			
				Cocaine, Urine	negative			
				Methadone, Urine	negative			
				Opiates, Urine	negative			
			Haematology	Basophils, abs.	0.02	10^9/L		
				Basophils, %	0.3	%		
				Eosinophils, abs.	0.11	10^9/L		
				Eosinophils, %	1.7	%		
				Haemoglobin	119.0	g/L		
				Haematocrit	0.35	L/L		
				Lymphocytes, abs.	2.73	10^9/L		
				Lymphocytes, %	42.2	%		
				Monocytes, abs.	0.47	10^9/L		
				Monocytes, %	7.3	%		
				Neutrophils, abs.	3.14	10^9/L		
				Neutrophils, %	48.5	%		
				Platelets	267	10^9/L		
				Erythrocytes	4.04	10^{12}/L		

Sign.: Significant finding; L: Result considered low; H: Result considered high; SC: Screening; BL: Baseline; D: Day; FUP: Follow-up; Cohort A3: Treatment was applied under fasting and fed conditions in the same subjects;

Output generated by program 'NIC002_L16_2_8_Laboratory_V02_0_0'

Listing 16.2.8: Study subject data
Laboratory values

Part A

Cohort	Subject ID	Visit	Type	Measurement	Result		Flagged	Sign.
Cohort A3 Fed	24	BL	Haematology	Leucocytes	6.47	10^9/L		
			Haemostasis	APTT	25.4	s		
				Prothrombin Time (INR)	0.98	N/A		
				Prothrombin Time (PT)	107.0	%		
			Urine	Beta-HCG, urine	negative			
		D02	Clinical Chemistry	ALT, 37 °C	10.5	U/L		
				Alkaline Phosphatase, 37 °C	46.0	U/L		
				AST, 37 °C	22.0	U/L		
				Bicarbonate	23.0	mmol/L		
				Bilirubin, total	8.2	umol/L		
				Urea/BUN	4.61	mmol/L		
				Calcium	2.40	mmol/L		
				Creatinine	74.8	umol/L		
				Glucose, serum	4.04	mmol/L	L	No
				Gamma-GT, 37 °C	16.9	U/L		
				Potassium	4.44	mmol/L		
				Magnesium	0.84	mmol/L		
				Sodium	135.0	mmol/L	L	No
			Haematology	Basophils, abs.	0.05	10^9/L		
				Basophils, %	0.7	%		
				Eosinophils, abs.	0.12	10^9/L		
				Eosinophils, %	1.6	%		
				Haemoglobin	126.0	g/L		
				Haematocrit	0.37	L/L		
				Lymphocytes, abs.	2.69	10^9/L		
				Lymphocytes, %	35.2	%		
				Monocytes, abs.	0.47	10^9/L		
				Monocytes, %	6.2	%		
				Neutrophils, abs.	4.31	10^9/L		
				Neutrophils, %	56.3	%		

Sign.: Significant finding; L: Result considered low; H: Result considered high; SC: Screening; BL: Baseline; D: Day; FUP: Follow-up; Cohort A3: Treatment was applied under fasting and fed conditions in the same subjects;

Output generated by program 'NIC002_L16_2_8_Laboratory_V02_0_0'

Listing 16.2.8: Study subject data
Laboratory values

Part A

Cohort	Subject ID	Visit	Type	Measurement	Result		Flagged	Sign.
		D02	Haematology	Platelets	236	10^9/L		
				Erythrocytes	4.32	10^{12}/L		
				Leucocytes	7.64	10^9/L		
			Haemostasis	APTT	24.2	s		
				Prothrombin Time (INR)	1.02	N/A		
				Prothrombin Time (PT)	98.8	%		
		FUP	Clinical Chemistry	ALT, 37 °C	12.1	U/L		
				Alkaline Phosphatase, 37 °C	52.7	U/L		
				AST, 37 °C	25.0	U/L		
				Bicarbonate	26.1	mmol/L		
				Bilirubin, total	8.0	umol/L		
				Urea/BUN	3.85	mmol/L		
				Calcium	2.45	mmol/L		
				Creatinine	70.9	umol/L		
				Glucose, serum	4.18	mmol/L		
				Gamma-GT, 37 °C	17.6	U/L		
				Potassium	3.57	mmol/L		
				Magnesium	0.77	mmol/L		
				Sodium	137.6	mmol/L		
			Haematology	Basophils, abs.	0.07	10^9/L		
				Basophils, %	1.0	%		
				Eosinophils, abs.	0.15	10^9/L		
				Eosinophils, %	2.2	%		
				Haemoglobin	127.0	g/L		
				Haematocrit	0.37	L/L		
				Lymphocytes, abs.	2.52	10^9/L		
				Lymphocytes, %	37.0	%		
				Monocytes, abs.	0.46	10^9/L		
				Monocytes, %	6.8	%		
				Neutrophils, abs.	3.61	10^9/L		

Sign.: Significant finding; L: Result considered low; H: Result considered high; SC: Screening; BL: Baseline; D: Day; FUP: Follow-up; Cohort A3: Treatment was applied under fasting and fed conditions in the same subjects;

Output generated by program 'NIC002_L16_2_8_Laboratory_V02_0_0'

Listing 16.2.8: Study subject data
Laboratory values

Part A

Cohort	Subject ID	Visit	Type	Measurement	Result		Flagged	Sign.
		FUP	Haematology	Neutrophils, %	53.0	%		
				Platelets	295	10^9/L		
				Erythrocytes	4.35	10^{12}/L		
				Leucocytes	6.81	10^9/L		
			Haemostasis	APTT	*	s		
				Prothrombin Time (INR)	*	N/A		
				Prothrombin Time (PT)	*	%		
			Urine	Beta-HCG, urine	negative			
	25	BL	Clinical Chemistry	ALT, 37 °C	14.5	U/L		
				Alkaline Phosphatase, 37 °C	54.5	U/L		
				AST, 37 °C	21.7	U/L		
				Bicarbonate	26.4	mmol/L		
				Bilirubin, total	7.6	umol/L		
				Urea/BUN	4.37	mmol/L		
				Calcium	2.30	mmol/L		
				Creatinine	56.9	umol/L		
				Glucose, serum	4.89	mmol/L		
				Gamma-GT, 37 °C	6.9	U/L		
				Potassium	4.17	mmol/L		
				Magnesium	0.76	mmol/L	L	No
				Sodium	134.6	mmol/L	L	No
			Drugs	Amphetamines, Urine	negative			
				Barbiturates, Urine	negative			
				Benzodiazepines, Urine	negative			
				Cannabin., Urine	negative			
				Cocaine, Urine	negative			
				Methadone, Urine	negative			
				Opiates, Urine	negative			
			Haematology	Basophils, abs.	0.02	10^9/L		
				Basophils, %	0.3	%		

Sign.: Significant finding; L: Result considered low; H: Result considered high; SC: Screening; BL: Baseline; D: Day; FUP: Follow-up; Cohort A3: Treatment was applied under fasting and fed conditions in the same subjects;

Output generated by program 'NIC002_L16_2_8_Laboratory_V02_0_0'

Listing 16.2.8: Study subject data
Laboratory values

Part A

Cohort	Subject ID	Visit	Type	Measurement	Result		Flagged	Sign.
	25	BL	Haematology	Eosinophils, abs.	0.10	10^9/L		
				Eosinophils, %	1.7	%		
				Haemoglobin	118.0	g/L		
				Haematocrit	0.35	L/L		
				Lymphocytes, abs.	2.20	10^9/L		
				Lymphocytes, %	36.5	%		
				Monocytes, abs.	0.59	10^9/L		
				Monocytes, %	9.8	%		
				Neutrophils, abs.	3.11	10^9/L		
				Neutrophils, %	51.7	%		
				Platelets	247	10^9/L		
				Erythrocytes	4.12	10^{12}/L		
				Leucocytes	6.02	10^9/L		
			Haemostasis	APTT	25.1	s		
				Prothrombin Time (INR)	1.03	N/A		
				Prothrombin Time (PT)	96.3	%		
			Urine	Beta-HCG, urine	negative			
		D02	Clinical Chemistry	ALT, 37 °C	13.2	U/L		
				Alkaline Phosphatase, 37 °C	50.2	U/L		
				AST, 37 °C	18.9	U/L		
				Bicarbonate	23.9	mmol/L		
				Bilirubin, total	8.7	umol/L		
				Urea/BUN	4.05	mmol/L		
				Calcium	2.29	mmol/L		
				Creatinine	56.7	umol/L		
				Glucose, serum	4.92	mmol/L		
				Gamma-GT, 37 °C	7.2	U/L		
				Potassium	4.09	mmol/L		
				Magnesium	0.74	mmol/L	L	No
				Sodium	134.9	mmol/L	L	No

Sign.: Significant finding; L: Result considered low; H: Result considered high; SC: Screening; BL: Baseline; D: Day; FUP: Follow-up; Cohort A3: Treatment was applied under fasting and fed conditions in the same subjects;

Output generated by program 'NIC002_L16_2_8_Laboratory_V02_0_0'

Listing 16.2.8: Study subject data
Laboratory values

Part A

Cohort	Subject ID	Visit	Type	Measurement	Result		Flagged	Sign.
		D02	Haematology	Basophils, abs.	0.03	10^9/L		
				Basophils, %	0.7	%		
				Eosinophils, abs.	0.07	10^9/L		
				Eosinophils, %	1.6	%		
				Haemoglobin	122.0	g/L		
				Haematocrit	0.37	L/L		
				Lymphocytes, abs.	1.68	10^9/L		
				Lymphocytes, %	39.3	%		
				Monocytes, abs.	0.38	10^9/L		
				Monocytes, %	8.9	%		
				Neutrophils, abs.	2.12	10^9/L		
				Neutrophils, %	49.5	%		
				Platelets	228	10^9/L		
				Erythrocytes	4.33	10^{12}/L		
				Leucocytes	4.28	10^9/L		
			Haemostasis	APTT	25.4	s		
				Prothrombin Time (INR)	1.05	N/A		
				Prothrombin Time (PT)	91.5	%		
		FUP	Clinical Chemistry	ALT, 37 °C	15.6	U/L		
				Alkaline Phosphatase, 37 °C	56.3	U/L		
				AST, 37 °C	22.8	U/L		
				Bicarbonate	26.1	mmol/L		
				Bilirubin, total	12.2	umol/L		
				Urea/BUN	4.00	mmol/L		
				Calcium	2.28	mmol/L		
				Creatinine	55.2	umol/L		
				Glucose, serum	4.62	mmol/L		
				Gamma-GT, 37 °C	6.9	U/L		
				Potassium	4.06	mmol/L		
				Magnesium	0.77	mmol/L		

Sign.: Significant finding; L: Result considered low; H: Result considered high; SC: Screening; BL: Baseline; D: Day; FUP: Follow-up; Cohort A3: Treatment was applied under fasting and fed conditions in the same subjects;

Output generated by program 'NIC002_L16_2_8_Laboratory_V02_0_0'

Listing 16.2.8: Study subject data
Laboratory values

Part A

Cohort	Subject ID	Visit	Type	Measurement	Result		Flagged	Sign.
		FUP	Clinical Chemistry	Sodium	137.4	mmol/L		
			Haematology	Basophils, abs.	0.03	10^9/L		
				Basophils, %	0.8	%		
				Eosinophils, abs.	0.08	10^9/L		
				Eosinophils, %	2.2	%		
				Haemoglobin	114.0	g/L		
				Haematocrit	0.33	L/L	L	No
				Lymphocytes, abs.	1.57	10^9/L		
				Lymphocytes, %	43.1	%		
				Monocytes, abs.	0.40	10^9/L		
				Monocytes, %	11.0	%		
				Neutrophils, abs.	1.56	10^9/L	L	No
				Neutrophils, %	42.9	%		
				Platelets	227	10^9/L		
				Erythrocytes	3.98	10^{12}/L	L	No
				Leucocytes	3.64	10^9/L	L	No
			Haemostasis	APTT	25.5	s		
				Prothrombin Time (INR)	1.05	N/A		
				Prothrombin Time (PT)	91.5	%		
			Urine	Beta-HCG, urine	negative			
	27	BL	Clinical Chemistry	ALT, 37 °C	14.7	U/L		
				Alkaline Phosphatase, 37 °C	56.3	U/L		
				AST, 37 °C	24.2	U/L		
				Bicarbonate	27.3	mmol/L		
				Bilirubin, total	10.4	umol/L		
				Urea/BUN	2.49	mmol/L	L	No
				Calcium	2.37	mmol/L		
				Creatinine	62.5	umol/L		
				Glucose, serum	4.59	mmol/L		
				Gamma-GT, 37 °C	13.3	U/L		

Sign.: Significant finding; L: Result considered low; H: Result considered high; SC: Screening; BL: Baseline; D: Day; FUP: Follow-up; Cohort A3: Treatment was applied under fasting and fed conditions in the same subjects;

Output generated by program 'NIC002_L16_2_8_Laboratory_V02_0_0'

Listing 16.2.8: Study subject data
Laboratory values

Part A

Cohort	Subject ID	Visit	Type	Measurement	Result		Flagged	Sign.
	27	BL	Clinical Chemistry	Potassium	4.17	mmol/L		
				Magnesium	0.86	mmol/L		
				Sodium	138.7	mmol/L		
			Drugs	Amphetamines, Urine	negative			
				Barbiturates, Urine	negative			
				Benzodiazepines, Urine	negative			
				Cannabin., Urine	negative			
				Cocaine, Urine	negative			
				Methadone, Urine	negative			
				Opiates, Urine	negative			
			Haematology	Basophils, abs.	0.05	10^9/L		
				Basophils, %	0.7	%		
				Eosinophils, abs.	0.40	10^9/L		
				Eosinophils, %	5.4	%		
				Haemoglobin	125.0	g/L		
				Haematocrit	0.37	L/L		
				Lymphocytes, abs.	1.90	10^9/L		
				Lymphocytes, %	25.6	%		
				Monocytes, abs.	0.61	10^9/L		
				Monocytes, %	8.2	%		
				Neutrophils, abs.	4.45	10^9/L		
				Neutrophils, %	60.1	%		
				Platelets	273	10^9/L		
				Erythrocytes	4.20	10^{12}/L		
				Leucocytes	7.41	10^9/L		
			Haemostasis	APTT	24.0	s		
				Prothrombin Time (INR)	1.13	N/A		
				Prothrombin Time (PT)	75.2	%		
			Urine	Beta-HCG, urine	negative			
		D02	Clinical Chemistry	ALT, 37 °C	13.4	U/L		

Sign.: Significant finding; L: Result considered low; H: Result considered high; SC: Screening; BL: Baseline; D: Day; FUP: Follow-up; Cohort A3: Treatment was applied under fasting and fed conditions in the same subjects;

Output generated by program 'NIC002_L16_2_8_Laboratory_V02_0_0'

Listing 16.2.8: Study subject data
Laboratory values

Part A

Cohort	Subject ID	Visit	Type	Measurement	Result		Flagged	Sign.
		D02	Clinical Chemistry	Alkaline Phosphatase, 37 °C	51.5	U/L		
				AST, 37 °C	22.2	U/L		
				Bicarbonate	26.2	mmol/L		
				Bilirubin, total	11.1	umol/L		
				Urea/BUN	3.46	mmol/L		
				Calcium	2.37	mmol/L		
				Creatinine	60.0	umol/L		
				Glucose, serum	4.64	mmol/L		
				Gamma-GT, 37 °C	13.1	U/L		
				Potassium	4.20	mmol/L		
				Magnesium	0.84	mmol/L		
				Sodium	137.9	mmol/L		
			Haematology	Basophils, abs.	0.05	10^9/L		
				Basophils, %	0.8	%		
				Eosinophils, abs.	0.40	10^9/L		
				Eosinophils, %	6.8	%		
				Haemoglobin	126.0	g/L		
				Haematocrit	0.37	L/L		
				Lymphocytes, abs.	1.81	10^9/L		
				Lymphocytes, %	30.6	%		
				Monocytes, abs.	0.41	10^9/L		
				Monocytes, %	6.9	%		
				Neutrophils, abs.	3.25	10^9/L		
				Neutrophils, %	54.9	%		
				Platelets	271	10^9/L		
				Erythrocytes	4.18	10^{12}/L		
				Leucocytes	5.92	10^9/L		
			Haemostasis	APTT	24.6	s		
				Prothrombin Time (INR)	1.16	N/A	H	No
				Prothrombin Time (PT)	70.1	%		

Sign.: Significant finding; L: Result considered low; H: Result considered high; SC: Screening; BL: Baseline; D: Day; FUP: Follow-up; Cohort A3: Treatment was applied under fasting and fed conditions in the same subjects;

Output generated by program 'NIC002_L16_2_8_Laboratory_V02_0_0'

Listing 16.2.8: Study subject data
Laboratory values

Part A

Cohort	Subject ID	Visit	Type	Measurement	Result		Flagged	Sign.
		FUP	Clinical Chemistry	ALT, 37 °C	16.0	U/L		
				Alkaline Phosphatase, 37 °C	62.2	U/L		
				AST, 37 °C	25.9	U/L		
				Bicarbonate	28.4	mmol/L		
				Bilirubin, total	13.5	umol/L		
				Urea/BUN	3.17	mmol/L		
				Calcium	2.44	mmol/L		
				Creatinine	64.1	umol/L		
				Glucose, serum	4.72	mmol/L		
				Gamma-GT, 37 °C	12.8	U/L		
				Potassium	4.54	mmol/L		
				Magnesium	0.85	mmol/L		
				Sodium	137.8	mmol/L		
			Haematology	Basophils, abs.	0.07	10^9/L		
				Basophils, %	0.9	%		
				Eosinophils, abs.	0.55	10^9/L	H	No
				Eosinophils, %	7.5	%		
				Haemoglobin	128.0	g/L		
				Haematocrit	0.38	L/L		
				Lymphocytes, abs.	2.39	10^9/L		
				Lymphocytes, %	32.4	%		
				Monocytes, abs.	0.49	10^9/L		
				Monocytes, %	6.6	%		
				Neutrophils, abs.	3.87	10^9/L		
				Neutrophils, %	52.6	%		
				Platelets	314	10^9/L		
				Erythrocytes	4.37	10^{12}/L		
				Leucocytes	7.37	10^9/L		
			Haemostasis	APTT	24.2	s		
				Prothrombin Time (INR)	1.15	N/A		
				Prothrombin Time (PT)	71.8	%		

Sign.: Significant finding; L: Result considered low; H: Result considered high; SC: Screening; BL: Baseline; D: Day; FUP: Follow-up; Cohort A3: Treatment was applied under fasting and fed conditions in the same subjects;

Output generated by program 'NIC002_L16_2_8_Laboratory_V02_0_0'

Listing 16.2.8: Study subject data
Laboratory values

Part A

Cohort	Subject ID	Visit	Type	Measurement	Result		Flagged	Sign.
		FUP	Urine	Beta-HCG, urine	negative			
	28	BL	Clinical Chemistry	ALT, 37 °C	15.5	U/L		
				Alkaline Phosphatase, 37 °C	73.8	U/L		
				AST, 37 °C	19.8	U/L		
				Bicarbonate	28.8	mmol/L		
				Bilirubin, total	11.9	umol/L		
				Urea/BUN	1.95	mmol/L	L	No
				Calcium	2.50	mmol/L		
				Creatinine	62.5	umol/L		
				Glucose, serum	4.47	mmol/L		
				Gamma-GT, 37 °C	11.5	U/L		
				Potassium	3.80	mmol/L		
				Magnesium	0.75	mmol/L	L	No
				Sodium	138.1	mmol/L		
			Drugs	Amphetamines, Urine	negative			
				Barbiturates, Urine	negative			
				Benzodiazepines, Urine	negative			
				Cannabin., Urine	negative			
				Cocaine, Urine	negative			
				Methadone, Urine	negative			
				Opiates, Urine	negative			
			Haematology	Basophils, abs.	0.04	10^9/L		
				Basophils, %	0.8	%		
				Eosinophils, abs.	0.15	10^9/L		
				Eosinophils, %	2.9	%		
				Haemoglobin	118.0	g/L		
				Haematocrit	0.33	L/L	L	No
				Lymphocytes, abs.	1.72	10^9/L		
				Lymphocytes, %	33.1	%		
				Monocytes, abs.	0.36	10^9/L		

Sign.: Significant finding; L: Result considered low; H: Result considered high; SC: Screening; BL: Baseline; D: Day; FUP: Follow-up; Cohort A3: Treatment was applied under fasting and fed conditions in the same subjects;

Output generated by program 'NIC002_L16_2_8_Laboratory_V02_0_0'

Listing 16.2.8: Study subject data
Laboratory values

Part A

Cohort	Subject ID	Visit	Type	Measurement	Result		Flagged	Sign.
	28	BL	Haematology	Monocytes, %	6.9	%		
				Neutrophils, abs.	2.93	10^9/L		
				Neutrophils, %	56.3	%		
				Platelets	405	10^9/L	H	No
				Erythrocytes	3.78	10^{12}/L	L	No
				Leucocytes	5.20	10^9/L		
			Haemostasis	APTT	26.5	s		
				Prothrombin Time (INR)	1.07	N/A		
				Prothrombin Time (PT)	87.0	%		
			Urine	Beta-HCG, urine	negative			
		D02	Clinical Chemistry	ALT, 37 °C	14.2	U/L		
				Alkaline Phosphatase, 37 °C	69.0	U/L		
				AST, 37 °C	17.6	U/L		
				Bicarbonate	26.3	mmol/L		
				Bilirubin, total	10.0	umol/L		
				Urea/BUN	2.17	mmol/L	L	No
				Calcium	2.46	mmol/L		
				Creatinine	55.4	umol/L		
				Glucose, serum	4.57	mmol/L		
				Gamma-GT, 37 °C	11.6	U/L		
				Potassium	4.54	mmol/L		
				Magnesium	0.71	mmol/L	L	No
				Sodium	138.1	mmol/L		
			Haematology	Basophils, abs.	0.07	10^9/L		
				Basophils, %	1.2	%		
				Eosinophils, abs.	0.19	10^9/L		
				Eosinophils, %	3.2	%		
				Haemoglobin	128.0	g/L		
				Haematocrit	0.36	L/L		
				Lymphocytes, abs.	1.60	10^9/L		

Sign.: Significant finding; L: Result considered low; H: Result considered high; SC: Screening; BL: Baseline; D: Day; FUP: Follow-up; Cohort A3: Treatment was applied under fasting and fed conditions in the same subjects;

Output generated by program 'NIC002_L16_2_8_Laboratory_V02_0_0'

Listing 16.2.8: Study subject data
Laboratory values

Part A

Cohort	Subject ID	Visit	Type	Measurement	Result		Flagged	Sign.
		D02	Haematology	Lymphocytes, %	26.6	%		
				Monocytes, abs.	0.40	10^9/L		
				Monocytes, %	6.6	%		
				Neutrophils, abs.	3.76	10^9/L		
				Neutrophils, %	62.4	%		
				Platelets	396	10^9/L	H	No
				Erythrocytes	4.15	10^{12}/L		
				Leucocytes	6.02	10^9/L		
			Haemostasis	APTT	26.2	s		
				Prothrombin Time (INR)	1.07	N/A		
				Prothrombin Time (PT)	87.0	%		
		FUP	Clinical Chemistry	ALT, 37 °C	16.8	U/L		
				Alkaline Phosphatase, 37 °C	73.7	U/L		
				AST, 37 °C	21.7	U/L		
				Bicarbonate	27.7	mmol/L		
				Bilirubin, total	13.4	umol/L		
				Urea/BUN	2.05	mmol/L	L	No
				Calcium	2.43	mmol/L		
				Creatinine	59.3	umol/L		
				Glucose, serum	4.63	mmol/L		
				Gamma-GT, 37 °C	11.2	U/L		
				Potassium	3.80	mmol/L		
				Magnesium	0.68	mmol/L	L	No
				Sodium	138.0	mmol/L		
			Haematology	Basophils, abs.	0.05	10^9/L		
				Basophils, %	0.8	%		
				Eosinophils, abs.	0.16	10^9/L		
				Eosinophils, %	2.7	%		
				Haemoglobin	120.0	g/L		
				Haematocrit	0.33	L/L	L	No

Sign.: Significant finding; L: Result considered low; H: Result considered high; SC: Screening; BL: Baseline; D: Day; FUP: Follow-up; Cohort A3: Treatment was applied under fasting and fed conditions in the same subjects;

Output generated by program 'NIC002_L16_2_8_Laboratory_V02_0_0'

Listing 16.2.8: Study subject data
Laboratory values

Part A

Cohort	Subject ID	Visit	Type	Measurement	Result		Flagged	Sign.
		FUP	Haematology	Lymphocytes, abs.	1.41	10^9/L		
				Lymphocytes, %	23.9	%		
				Monocytes, abs.	0.41	10^9/L		
				Monocytes, %	7.0	%		
				Neutrophils, abs.	3.86	10^9/L		
				Neutrophils, %	65.6	%		
				Platelets	385	10^9/L	H	No
				Erythrocytes	3.87	10^{12}/L	L	No
				Leucocytes	5.89	10^9/L		
			Haemostasis	APTT	27.1	s		
				Prothrombin Time (INR)	1.06	N/A		
				Prothrombin Time (PT)	89.2	%		
			Urine	Beta-HCG, urine	negative			

Sign.: Significant finding; L: Result considered low; H: Result considered high; SC: Screening; BL: Baseline; D: Day; FUP: Follow-up; Cohort A3: Treatment was applied under fasting and fed conditions in the same subjects;

Output generated by program 'NIC002_L16_2_8_Laboratory_V02_0_0'

Listing 16.2.8: Study subject data
Laboratory values

Part B

Cohort	Subject ID	Visit	Type	Measurement	Result		Flagged	Sign.
Chewing tablet 2000 mg	48	D04	Clinical Chemistry	ALT, 37°C	15.2	U/L		
				Alkaline Phosphatase, 37°C	52.7	U/L		
				AST, 37°C	21.7	U/L		
				Bicarbonate	26.4	mmol/L		
				Bilirubin, total	6.6	umol/L		
				Urea/BUN	2.93	mmol/L		
				Calcium	2.37	mmol/L		
				Creatinine	46.9	umol/L		
				Glucose, serum	4.47	mmol/L		
				Gamma-GT, 37°C	10.6	U/L		
				Potassium	3.80	mmol/L		
				Magnesium	0.87	mmol/L		
				Sodium	139.5	mmol/L		
			Haematology	Basophils, abs.	0.03	10^9/L		
				Basophils, %	0.5	%		
				Eosinophils, abs.	0.10	10^9/L		
				Eosinophils, %	1.5	%		
				Haemoglobin	131.0	g/L		
				Haematocrit	0.38	L/L		
				Lymphocytes, abs.	2.40	10^9/L		
				Lymphocytes, %	36.2	%		
				Monocytes, abs.	0.51	10^9/L		
				Monocytes, %	7.7	%		
				Neutrophils, abs.	3.59	10^9/L		
				Neutrophils, %	54.1	%		
				Platelets	301	10^9/L		
				Erythrocytes	4.09	10^{12}/L		
				Leucocytes	6.63	10^9/L		
			Haemostasis	APTT	27.1	s		

Sign.: Significant finding; L: Result considered low; H: Result considered high; SC: Screening; BL: Baseline; D: Day; FUP: Follow-up; Part B used a cross-over design;

Output generated by program 'NIC002_L16_2_8_Laboratory_V02_0_0'

Listing 16.2.8: Study subject data
Laboratory values

Part B

Cohort	Subject ID	Visit	Type	Measurement	Result		Flagged	Sign.
Chewing tablet 2000 mg	48	D04	Haemostasis	Prothrombin Time (INR)	0.99	N/A		
				Prothrombin Time (PT)	104.2	%		
		FUP	Clinical Chemistry	ALT, 37°C	17.4	U/L		
				Alkaline Phosphatase, 37°C	56.5	U/L		
				AST, 37°C	26.1	U/L		
				Bicarbonate	25.4	mmol/L		
				Bilirubin, total	8.8	umol/L		
				Urea/BUN	2.15	mmol/L	L	No
				Calcium	2.20	mmol/L		
				Creatinine	49.0	umol/L		
				Glucose, serum	4.37	mmol/L		
				Gamma-GT, 37°C	10.9	U/L		
				Potassium	4.28	mmol/L		
				Magnesium	0.96	mmol/L		
				Sodium	133.5	mmol/L	L	No
			Haematology	Basophils, abs.	0.03	10^9/L		
				Basophils, %	0.5	%		
				Eosinophils, abs.	0.10	10^9/L		
				Eosinophils, %	1.6	%		
				Haemoglobin	134.0	g/L		
				Haematocrit	0.38	L/L		
				Lymphocytes, abs.	1.67	10^9/L		
				Lymphocytes, %	27.1	%		
				Monocytes, abs.	0.52	10^9/L		
				Monocytes, %	8.4	%		
				Neutrophils, abs.	3.84	10^9/L		
				Neutrophils, %	62.4	%		
				Platelets	331	10^9/L		
				Erythrocytes	4.16	10^{12}/L		
				Leucocytes	6.16	10^9/L		

Sign.: Significant finding; L: Result considered low; H: Result considered high; SC: Screening; BL: Baseline; D: Day; FUP: Follow-up; Part B used a cross-over design;

Output generated by program 'NIC002_L16_2_8_Laboratory_V02_0_0'

Listing 16.2.8: Study subject data
Laboratory values

Part B

Cohort	Subject ID	Visit	Type	Measurement	Result		Flagged	Sign.
		FUP	Haemostasis	APTT	27.1	s		
				Prothrombin Time (INR)	0.96	N/A		
				Prothrombin Time (PT)	113.0	%		
			Urine	Beta-HCG, urine	negative			
	49	SC	Clinical Chemistry	ALT, 37°C	8.6	U/L		
				Alkaline Phosphatase, 37°C	94.2	U/L		
				AST, 37°C	16.4	U/L		
				Bicarbonate	27.6	mmol/L		
				Bilirubin, total	6.2	umol/L		
				Urea/BUN	2.30	mmol/L	L	No
				Calcium	2.40	mmol/L		
				Creatinine	54.7	umol/L		
				Glucose, serum	4.87	mmol/L		
				Gamma-GT, 37°C	10.6	U/L		
				Potassium	3.94	mmol/L		
				Creatinine Clearance MDRD	114	ml/min/1.73m		
				Magnesium	0.79	mmol/L		
				Sodium	138.0	mmol/L		
			Drugs	Amphetamines, Urine	negative			
				Barbiturates, Urine	negative			
				Benzodiazepines, Urine	negative			
				Cannabin., Urine	negative			
				Cocaine, Urine	negative			
				Methadone, Urine	negative			
				Opiates, Urine	negative			
			Haematology	Basophils, abs.	0.04	$10^9/L$		
				Basophils, %	0.8	%		
				Eosinophils, abs.	0.16	$10^9/L$		
				Eosinophils, %	3.1	%		
				Haemoglobin	125.0	g/L		

Sign.: Significant finding; L: Result considered low; H: Result considered high; SC: Screening; BL: Baseline; D: Day; FUP: Follow-up; Part B used a cross-over design;

Output generated by program 'NIC002_L16_2_8_Laboratory_V02_0_0'

Listing 16.2.8: Study subject data
Laboratory values

Part B

Cohort	Subject ID	Visit	Type	Measurement	Result		Flagged	Sign.
	49	SC	Haematology	Haematocrit	0.37	L/L		
				Lymphocytes, abs.	1.46	10^9/L		
				Lymphocytes, %	27.9	%		
				Monocytes, abs.	0.48	10^9/L		
				Monocytes, %	9.2	%		
				Neutrophils, abs.	3.09	10^9/L		
				Neutrophils, %	59.0	%		
				Platelets	246	10^9/L		
				Erythrocytes	4.31	10^{12}/L		
				Leucocytes	5.23	10^9/L		
			Haemostasis	APTT	27.9	s		
				Prothrombin Time (INR)	1.01	N/A		
				Prothrombin Time (PT)	98.8	%		
			Infectious Diseases	HBs-Ag (Hep. B Surf. Ag)	negative	N/A		
				Anti-HCV (Hep. C-AB)	non-reactive	N/A		
				HIV 1+2, AG/AB	negative	N/A		
			Urine	Bilirubin, urine (Stix)	negative			
				Bilirubin, urine (Stix)	negative			
				Blood (Ery/Hb), urine (Stix)	2+		H	No
				Blood (Ery/Hb), urine (Stix)	1+		H	No
				Glucose, urine (Stix)	negative			
				Glucose, urine (Stix)	negative			
				Beta-HCG, urine	negative			
				Beta-HCG, urine	negative			
				Ketone, urine (Stix)	negative			
				Ketone, urine (Stix)	negative			
				Leucocytes, urine (Stix)	trace		H	No
				Leucocytes, urine (Stix)	2+		H	No
				Nitrite, urine (Stix)	positive		H	No
				Nitrite, urine (Stix)	negative			
				pH, urine (Stix)	6.5	neg.log[H+]		

Sign.: Significant finding; L: Result considered low; H: Result considered high; SC: Screening; BL: Baseline; D: Day; FUP: Follow-up; Part B used a cross-over design;

Output generated by program 'NIC002_L16_2_8_Laboratory_V02_0_0'

Listing 16.2.8: Study subject data
Laboratory values

Part B

Cohort	Subject ID	Visit	Type	Measurement	Result		Flagged	Sign.
	49	SC	Urine	pH, urine (Stix)	6.0	neg.log[H+]		
				Protein, total, urine (Stix)	negative			
				Protein, total, urine (Stix)	negative			
				Bacteria, Sediment	positive		H	No
				Bacteria, Sediment	positive		H	No
				Carbonate, Sediment	negative			
				Carbonate, Sediment	negative			
				Epithelial Cells, Sediment	> 20	per field	H	No
				Epithelial Cells, Sediment	30	per field	H	No
				Erythrocytes, Sediment	2	per field	H	No
				Erythrocytes, Sediment	1	per field		
				Casts granul., Sediment	0	per field		
				Casts granul., Sediment	0	per field		
				Casts hyaline, Sediment	0	per field		
				Casts hyaline, Sediment	0	per field		
				Leucocytes, Sediment	> 30	per field	H	No
				Leucocytes, Sediment	20	per field	H	No
				Oxalate, Sediment	negative			
				Oxalate, Sediment	negative			
				Specific Gravity	1.015			
				Specific Gravity	<=1.005			
				Mucus, Sediment	positive		H	No
				Mucus, Sediment	negative			
				Triple Phosphate, Sediment	negative			
				Triple Phosphate, Sediment	negative			
				Urates, Sediment	negative			
				Urates, Sediment	negative			
				Urobilinogen, urine (Stix)	0.2	mg/dl		
				Urobilinogen, urine (Stix)	0.2	mg/dl		
		Unscheduled	Urine	Bilirubin, urine (Stix)	negative			

Sign.: Significant finding; L: Result considered low; H: Result considered high; SC: Screening; BL: Baseline; D: Day; FUP: Follow-up; Part B used a cross-over design;

Output generated by program 'NIC002_L16_2_8_Laboratory_V02_0_0'

Listing 16.2.8: Study subject data
Laboratory values

Part B

Cohort	Subject ID	Visit	Type	Measurement	Result		Flagged	Sign.
		Unscheduled	Urine	Blood (Ery/Hb), urine (Stix)	1+		H	No
				Glucose, urine (Stix)	negative			
				Beta-HCG, urine	negative			
				Ketone, urine (Stix)	negative			
				Leucocytes, urine (Stix)	2+		H	No
				Nitrite, urine (Stix)	negative			
				pH, urine (Stix)	6.0	neg.log[H+]		
				Protein, total, urine (Stix)	negative			
				Bacteria, Sediment	positive		H	No
				Carbonate, Sediment	negative			
				Epithelial Cells, Sediment	30	per field	H	No
				Erythrocytes, Sediment	1	per field		
				Casts granul., Sediment	0	per field		
				Casts hyaline, Sediment	0	per field		
				Leucocytes, Sediment	20	per field	H	No
				Oxalate, Sediment	negative			
				Specific Gravity	<=1.005			
				Mucus, Sediment	negative			
				Triple Phosphate, Sediment	negative			
				Urates, Sediment	negative			
				Urobilinogen, urine (Stix)	0.2	mg/dl		
		BL	Clinical Chemistry	ALT, 37°C	9.8	U/L		
				Alkaline Phosphatase, 37°C	93.5	U/L		
				AST, 37°C	18.7	U/L		
				Bicarbonate	25.5	mmol/L		
				Bilirubin, total	9.3	umol/L		
				Urea/BUN	2.82	mmol/L		
				Calcium	2.36	mmol/L		
				Creatinine	50.4	umol/L		
				Glucose, serum	4.81	mmol/L		

Sign.: Significant finding; L: Result considered low; H: Result considered high; SC: Screening; BL: Baseline; D: Day; FUP: Follow-up; Part B used a cross-over design;

Output generated by program 'NIC002_L16_2_8_Laboratory_V02_0_0'

Listing 16.2.8: Study subject data
Laboratory values

Part B

Cohort	Subject ID	Visit	Type	Measurement	Result		Flagged	Sign.
		BL	Clinical Chemistry	Gamma-GT, 37°C	10.2	U/L		
				Potassium	3.87	mmol/L		
				Magnesium	0.71	mmol/L	L	No
				Sodium	135.1	mmol/L	L	No
			Drugs	Amphetamines, Urine	negative			
				Barbiturates, Urine	negative			
				Benzodiazepines, Urine	negative			
				Cannabin., Urine	negative			
				Cocaine, Urine	negative			
				Methadone, Urine	negative			
				Opiates, Urine	negative			
			Haematology	Basophils, abs.	0.04	10^9/L		
				Basophils, %	0.7	%		
				Eosinophils, abs.	0.18	10^9/L		
				Eosinophils, %	3.0	%		
				Haemoglobin	125.0	g/L		
				Haematocrit	0.36	L/L		
				Lymphocytes, abs.	1.78	10^9/L		
				Lymphocytes, %	29.8	%		
				Monocytes, abs.	0.47	10^9/L		
				Monocytes, %	7.9	%		
				Neutrophils, abs.	3.50	10^9/L		
				Neutrophils, %	58.6	%		
				Platelets	233	10^9/L		
				Erythrocytes	4.25	10^{12}/L		
				Leucocytes	5.97	10^9/L		
			Haemostasis	APTT	27.5	s		
				Prothrombin Time (INR)	1.01	N/A		
				Prothrombin Time (PT)	98.8	%		
			Urine	Beta-HCG, urine	negative			

Sign.: Significant finding; L: Result considered low; H: Result considered high; SC: Screening; BL: Baseline; D: Day; FUP: Follow-up; Part B used a cross-over design;

Output generated by program 'NIC002_L16_2_8_Laboratory_V02_0_0'

Listing 16.2.8: Study subject data
Laboratory values

Part B

Cohort	Subject ID	Visit	Type	Measurement	Result		Flagged	Sign.
		D02	Clinical Chemistry	ALT, 37°C	8.0	U/L		
				Alkaline Phosphatase, 37°C	82.5	U/L		
				AST, 37°C	16.0	U/L		
				Bicarbonate	23.9	mmol/L		
				Bilirubin, total	8.8	umol/L		
				Urea/BUN	3.05	mmol/L		
				Calcium	2.26	mmol/L		
				Creatinine	46.7	umol/L		
				Glucose, serum	4.36	mmol/L		
				Gamma-GT, 37°C	9.8	U/L		
				Potassium	4.12	mmol/L		
				Magnesium	0.79	mmol/L		
				Sodium	135.8	mmol/L	L	No
			Haematology	Basophils, abs.	0.04	$10^9/L$		
				Basophils, %	0.8	%		
				Eosinophils, abs.	0.14	$10^9/L$		
				Eosinophils, %	2.7	%		
				Haemoglobin	122.0	g/L		
				Haematocrit	0.35	L/L		
				Lymphocytes, abs.	1.44	$10^9/L$		
				Lymphocytes, %	27.5	%		
				Monocytes, abs.	0.39	$10^9/L$		
				Monocytes, %	7.4	%		
				Neutrophils, abs.	3.23	$10^9/L$		
				Neutrophils, %	61.6	%		
				Platelets	241	$10^9/L$		
				Erythrocytes	4.14	$10^{12}/L$		
				Leucocytes	5.24	$10^9/L$		
			Haemostasis	APTT	28.0	s		
				Prothrombin Time (INR)	1.05	N/A		
				Prothrombin Time (PT)	91.5	%		

Sign.: Significant finding; L: Result considered low; H: Result considered high; SC: Screening; BL: Baseline; D: Day; FUP: Follow-up; Part B used a cross-over design;

Output generated by program 'NIC002_L16_2_8_Laboratory_V02_0_0'

Listing 16.2.8: Study subject data
Laboratory values

Part B

Cohort	Subject ID	Visit	Type	Measurement	Result		Flagged	Sign.
	51	D04	Clinical Chemistry	ALT, 37°C	12.3	U/L		
				Alkaline Phosphatase, 37°C	36.4	U/L		
				AST, 37°C	19.3	U/L		
				Bicarbonate	26.5	mmol/L		
				Bilirubin, total	11.4	umol/L		
				Urea/BUN	3.79	mmol/L		
				Calcium	2.35	mmol/L		
				Creatinine	44.9	umol/L	L	No
				Glucose, serum	4.63	mmol/L		
				Gamma-GT, 37°C	8.7	U/L		
				Potassium	3.78	mmol/L		
				Magnesium	0.80	mmol/L		
				Sodium	138.5	mmol/L		
			Haematology	Basophils, abs.	0.02	10^9/L		
				Basophils, %	0.4	%		
				Eosinophils, abs.	0.11	10^9/L		
				Eosinophils, %	2.1	%		
				Haemoglobin	129.0	g/L		
				Haematocrit	0.36	L/L		
				Lymphocytes, abs.	2.02	10^9/L		
				Lymphocytes, %	38.3	%		
				Monocytes, abs.	0.40	10^9/L		
				Monocytes, %	7.6	%		
				Neutrophils, abs.	2.73	10^9/L		
				Neutrophils, %	51.6	%		
				Platelets	268	10^9/L		
				Erythrocytes	4.00	10^{12}/L	L	No
				Leucocytes	5.28	10^9/L		
			Haemostasis	APTT	27.7	s		
				Prothrombin Time (INR)	1.06	N/A		

Sign.: Significant finding; L: Result considered low; H: Result considered high; SC: Screening; BL: Baseline; D: Day; FUP: Follow-up; Part B used a cross-over design;

Output generated by program 'NIC002_L16_2_8_Laboratory_V02_0_0'

Listing 16.2.8: Study subject data
Laboratory values

Part B

Cohort	Subject ID	Visit	Type	Measurement	Result		Flagged	Sign.
	51	D04	Haemostasis	Prothrombin Time (PT)	89.2	%		
		FUP	Clinical Chemistry	ALT, 37°C	18.2	U/L		
				Alkaline Phosphatase, 37°C	35.7	U/L		
				AST, 37°C	24.8	U/L		
				Bicarbonate	28.7	mmol/L		
				Bilirubin, total	11.3	umol/L		
				Urea/BUN	3.42	mmol/L		
				Calcium	2.32	mmol/L		
				Creatinine	49.0	umol/L		
				Glucose, serum	4.60	mmol/L		
				Gamma-GT, 37°C	9.0	U/L		
				Potassium	4.46	mmol/L		
				Magnesium	0.77	mmol/L		
				Sodium	137.1	mmol/L		
			Haematology	Basophils, abs.	0.03	10^9/L		
				Basophils, %	0.7	%		
				Eosinophils, abs.	0.09	10^9/L		
				Eosinophils, %	2.1	%		
				Haemoglobin	127.0	g/L		
				Haematocrit	0.35	L/L		
				Lymphocytes, abs.	1.77	10^9/L		
				Lymphocytes, %	41.0	%		
				Monocytes, abs.	0.33	10^9/L		
				Monocytes, %	7.6	%		
				Neutrophils, abs.	2.10	10^9/L		
				Neutrophils, %	48.6	%		
				Platelets	264	10^9/L		
				Erythrocytes	3.93	10^{12}/L	L	No
				Leucocytes	4.32	10^9/L		
			Haemostasis	APTT	27.5	s		

Sign.: Significant finding; L: Result considered low; H: Result considered high; SC: Screening; BL: Baseline; D: Day; FUP: Follow-up; Part B used a cross-over design;

Output generated by program 'NIC002_L16_2_8_Laboratory_V02_0_0'

Listing 16.2.8: Study subject data
Laboratory values

Part B

Cohort	Subject ID	Visit	Type	Measurement	Result		Flagged	Sign.
		FUP	Haemostasis	Prothrombin Time (INR)	1.05	N/A		
				Prothrombin Time (PT)	91.5	%		
			Urine	Beta-HCG, urine	negative			
	76	SC	Clinical Chemistry	ALT, 37°C	9.3	U/L		
				Alkaline Phosphatase, 37°C	48.0	U/L		
				AST, 37°C	17.5	U/L		
				Bicarbonate	27.7	mmol/L		
				Bilirubin, total	16.3	umol/L		
				Urea/BUN	4.25	mmol/L		
				Calcium	2.35	mmol/L		
				Creatinine	50.6	umol/L		
				Glucose, serum	3.79	mmol/L	L	No
				Gamma-GT, 37°C	10.4	U/L		
				Potassium	3.66	mmol/L		
				Creatinine Clearance MDRD	128	ml/min/1.73m		
				Magnesium	0.81	mmol/L		
				Sodium	135.8	mmol/L	L	No
			Drugs	Amphetamines, Urine	negative			
				Barbiturates, Urine	negative			
				Benzodiazepines, Urine	negative			
				Cannabin., Urine	negative			
				Cocaine, Urine	negative			
				Methadone, Urine	negative			
				Opiates, Urine	negative			
			Haematology	Basophils, abs.	0.03	10^9/L		
				Basophils, %	0.5	%		
				Eosinophils, abs.	0.27	10^9/L		
				Eosinophils, %	4.2	%		
				Haemoglobin	125.0	g/L		
				Haematocrit	0.36	L/L		

Sign.: Significant finding; L: Result considered low; H: Result considered high; SC: Screening; BL: Baseline; D: Day; FUP: Follow-up; Part B used a cross-over design;

Output generated by program 'NIC002_L16_2_8_Laboratory_V02_0_0'

Listing 16.2.8: Study subject data
Laboratory values

Part B

Cohort	Subject ID	Visit	Type	Measurement	Result		Flagged	Sign.
	76	SC	Haematology	Lymphocytes, abs.	2.77	10^9/L		
				Lymphocytes, %	43.1	%		
				Monocytes, abs.	0.49	10^9/L		
				Monocytes, %	7.6	%		
				Neutrophils, abs.	2.87	10^9/L		
				Neutrophils, %	44.6	%		
				Platelets	323	10^9/L		
				Erythrocytes	3.92	10^{12}/L	L	No
				Leucocytes	6.43	10^9/L		
			Haemostasis	APTT	26.8	s		
				Prothrombin Time (INR)	1.07	N/A		
				Prothrombin Time (PT)	87.0	%		
			Infectious Diseases	HBs-Ag (Hep. B Surf. Ag)	negative	N/A		
				Anti-HCV (Hep. C-AB)	non-reactive	N/A		
				HIV 1+2, AG/AB	negative	N/A		
			Urine	Bilirubin, urine (Stix)	negative			
				Blood (Ery/Hb), urine (Stix)	3+		H	No
				Glucose, urine (Stix)	negative			
				Beta-HCG, urine	negative			
				Ketone, urine (Stix)	negative			
				Leucocytes, urine (Stix)	negative			
				Nitrite, urine (Stix)	negative			
				pH, urine (Stix)	5.5	neg.log[H+]		
				Protein, total, urine (Stix)	negative			
				Bacteria, Sediment	positive		H	No
				Carbonate, Sediment	negative			
				Epithelial Cells, Sediment	10	per field		
				Erythrocytes, Sediment	3	per field	H	No
				Casts granul., Sediment	0	per field		
				Casts hyaline, Sediment	0	per field		
				Leucocytes, Sediment	1	per field		

Sign.: Significant finding; L: Result considered low; H: Result considered high; SC: Screening; BL: Baseline; D: Day; FUP: Follow-up; Part B used a cross-over design;

Output generated by program 'NIC002_L16_2_8_Laboratory_V02_0_0'

Listing 16.2.8: Study subject data
Laboratory values

Part B

Cohort	Subject ID	Visit	Type	Measurement	Result		Flagged	Sign.
	76	SC	Urine	Oxalate, Sediment	negative			
				Specific Gravity	<=1.005			
				Mucus, Sediment	positive		H	No
				Triple Phosphate, Sediment	negative			
				Urates, Sediment	negative			
				Urobilinogen, urine (Stix)	0.2	mg/dl		
		BL	Clinical Chemistry	ALT, 37°C	8.3	U/L		
				Alkaline Phosphatase, 37°C	47.6	U/L		
				AST, 37°C	13.1	U/L		
				Bicarbonate	27.6	mmol/L		
				Bilirubin, total	13.1	umol/L		
				Urea/BUN	5.07	mmol/L		
				Calcium	2.34	mmol/L		
				Creatinine	51.1	umol/L		
				Glucose, serum	4.70	mmol/L		
				Gamma-GT, 37°C	10.5	U/L		
				Potassium	3.91	mmol/L		
				Magnesium	0.73	mmol/L	L	No
				Sodium	136.4	mmol/L		
			Drugs	Amphetamines, Urine	negative			
				Barbiturates, Urine	negative			
				Benzodiazepines, Urine	negative			
				Cannabin., Urine	negative			
				Cocaine, Urine	negative			
				Methadone, Urine	negative			
				Opiates, Urine	negative			
			Haematology	Basophils, abs.	0.03	10^9/L		
				Basophils, %	0.4	%		
				Eosinophils, abs.	0.48	10^9/L	H	No
				Eosinophils, %	6.6	%		

Sign.: Significant finding; L: Result considered low; H: Result considered high; SC: Screening; BL: Baseline; D: Day; FUP: Follow-up; Part B used a cross-over design;

Output generated by program 'NIC002_L16_2_8_Laboratory_V02_0_0'

Listing 16.2.8: Study subject data
Laboratory values

Part B

Cohort	Subject ID	Visit	Type	Measurement	Result		Flagged	Sign.
		BL	Haematology	Haemoglobin	123.0	g/L		
				Haematocrit	0.36	L/L		
				Lymphocytes, abs.	3.24	10^9/L	H	No
				Lymphocytes, %	44.5	%		
				Monocytes, abs.	0.72	10^9/L		
				Monocytes, %	9.9	%		
				Neutrophils, abs.	2.81	10^9/L		
				Neutrophils, %	38.6	%		
				Platelets	323	10^9/L		
				Erythrocytes	3.95	10^{12}/L	L	No
				Leucocytes	7.28	10^9/L		
			Haemostasis	APTT	26.8	s		
				Prothrombin Time (INR)	1.02	N/A		
				Prothrombin Time (PT)	97.2	%		
			Urine	Beta-HCG, urine	negative			
		D02	Clinical Chemistry	ALT, 37°C	10.2	U/L		
				Alkaline Phosphatase, 37°C	48.7	U/L		
				AST, 37°C	14.6	U/L		
				Bicarbonate	25.9	mmol/L		
				Bilirubin, total	14.2	umol/L		
				Urea/BUN	4.90	mmol/L		
				Calcium	2.32	mmol/L		
				Creatinine	53.3	umol/L		
				Glucose, serum	4.96	mmol/L		
				Gamma-GT, 37°C	10.9	U/L		
				Potassium	4.02	mmol/L		
				Magnesium	0.75	mmol/L	L	No
				Sodium	133.7	mmol/L	L	No
			Haematology	Basophils, abs.	0.01	10^9/L		
				Basophils, %	0.1	%	L	No

Sign.: Significant finding; L: Result considered low; H: Result considered high; SC: Screening; BL: Baseline; D: Day; FUP: Follow-up; Part B used a cross-over design;

Output generated by program 'NIC002_L16_2_8_Laboratory_V02_0_0'

Listing 16.2.8: Study subject data
Laboratory values

Part B

Cohort	Subject ID	Visit	Type	Measurement	Result		Flagged	Sign.
		D02	Haematology	Eosinophils, abs.	0.48	10^9/L	H	No
				Eosinophils, %	5.4	%		
				Haemoglobin	133.0	g/L		
				Haematocrit	0.39	L/L		
				Lymphocytes, abs.	4.07	10^9/L	H	No
				Lymphocytes, %	45.6	%		
				Monocytes, abs.	0.72	10^9/L		
				Monocytes, %	8.1	%		
				Neutrophils, abs.	3.65	10^9/L		
				Neutrophils, %	40.8	%		
				Platelets	350	10^9/L		
				Erythrocytes	4.26	10^{12}/L		
				Leucocytes	8.93	10^9/L		
			Haemostasis	APTT	26.6	s		
				Prothrombin Time (INR)	1.02	N/A		
				Prothrombin Time (PT)	97.2	%		
Solution 1600 mg	48	SC	Clinical Chemistry	ALT, 37°C	16.0	U/L		
				Alkaline Phosphatase, 37°C	59.1	U/L		
				AST, 37°C	24.9	U/L		
				Bicarbonate	31.3	mmol/L	H	No
				Bilirubin, total	8.2	umol/L		
				Urea/BUN	1.85	mmol/L	L	No
				Calcium	2.38	mmol/L		
				Creatinine	50.5	umol/L		
				Glucose, serum	5.26	mmol/L		
				Gamma-GT, 37°C	11.0	U/L		
				Potassium	4.76	mmol/L		
				Creatinine Clearance MDRD	123	ml/min/1.73m		
				Magnesium	0.87	mmol/L		

Sign.: Significant finding; L: Result considered low; H: Result considered high; SC: Screening; BL: Baseline; D: Day; FUP: Follow-up; Part B used a cross-over design;

Output generated by program 'NIC002_L16_2_8_Laboratory_V02_0_0'

Listing 16.2.8: Study subject data
Laboratory values

Part B

Cohort	Subject ID	Visit	Type	Measurement	Result		Flagged	Sign.
Solution 1600 mg	48	SC	Clinical Chemistry	Sodium	136.9	mmol/L		
			Drugs	Amphetamines, Urine	negative			
				Barbiturates, Urine	negative			
				Benzodiazepines, Urine	negative			
				Cannabin., Urine	negative			
				Cocaine, Urine	negative			
				Methadone, Urine	negative			
				Opiates, Urine	negative			
			Haematology	Basophils, abs.	0.03	10^9/L		
				Basophils, %	0.5	%		
				Eosinophils, abs.	0.10	10^9/L		
				Eosinophils, %	1.6	%		
				Haemoglobin	128.0	g/L		
				Haematocrit	0.38	L/L		
				Lymphocytes, abs.	1.91	10^9/L		
				Lymphocytes, %	30.0	%		
				Monocytes, abs.	0.52	10^9/L		
				Monocytes, %	8.2	%		
				Neutrophils, abs.	3.81	10^9/L		
				Neutrophils, %	59.7	%		
				Platelets	317	10^9/L		
				Erythrocytes	4.12	10^{12}/L		
				Leucocytes	6.37	10^9/L		
			Haemostasis	APTT	24.8	s		
				Prothrombin Time (INR)	0.97	N/A		
				Prothrombin Time (PT)	109.9	%		
			Infectious Diseases	HBs-Ag (Hep. B Surf. Ag)	negative	N/A		
				Anti-HCV (Hep. C-AB)	non-reactive	N/A		
				HIV 1+2, AG/AB	negative	N/A		
			Urine	Bilirubin, urine (Stix)	negative			
				Blood (Ery/Hb), urine (Stix)	2+		H	No

Sign.: Significant finding; L: Result considered low; H: Result considered high; SC: Screening; BL: Baseline; D: Day; FUP: Follow-up; Part B used a cross-over design;

Output generated by program 'NIC002_L16_2_8_Laboratory_V02_0_0'

Listing 16.2.8: Study subject data
Laboratory values

Part B

Cohort	Subject ID	Visit	Type	Measurement	Result		Flagged	Sign.
Solution 1600 mg	48	SC	Urine	Glucose, urine (Stix)	negative			
				Beta-HCG, urine	negative			
				Ketone, urine (Stix)	negative			
				Leucocytes, urine (Stix)	negative			
				Nitrite, urine (Stix)	negative			
				pH, urine (Stix)	7.0	neg.log[H+]		
				Protein, total, urine (Stix)	negative			
				Bacteria, Sediment	negative			
				Carbonate, Sediment	negative			
				Epithelial Cells, Sediment	3	per field	L	No
				Erythrocytes, Sediment	0	per field		
				Casts granul., Sediment	0	per field		
				Casts hyaline, Sediment	0	per field		
				Leucocytes, Sediment	0	per field		
				Oxalate, Sediment	negative			
				Specific Gravity	<=1.005			
				Mucus, Sediment	negative			
				Triple Phosphate, Sediment	negative			
				Urates, Sediment	negative			
				Urobilinogen, urine (Stix)	0.2	mg/dl		
		BL	Clinical Chemistry	ALT, 37°C	16.3	U/L		
				Alkaline Phosphatase, 37°C	54.9	U/L		
				AST, 37°C	26.9	U/L		
				Bicarbonate	26.5	mmol/L		
				Bilirubin, total	7.7	umol/L		
				Urea/BUN	1.65	mmol/L	L	No
				Calcium	2.20	mmol/L		
				Creatinine	44.1	umol/L	L	No
				Glucose, serum	5.23	mmol/L		
				Gamma-GT, 37°C	10.9	U/L		

Sign.: Significant finding; L: Result considered low; H: Result considered high; SC: Screening; BL: Baseline; D: Day; FUP: Follow-up; Part B used a cross-over design;

Output generated by program 'NIC002_L16_2_8_Laboratory_V02_0_0'

Listing 16.2.8: Study subject data
Laboratory values

Part B

Cohort	Subject ID	Visit	Type	Measurement	Result		Flagged	Sign.
		BL	Clinical Chemistry	Potassium	4.62	mmol/L		
				Magnesium	0.87	mmol/L		
				Sodium	133.9	mmol/L	L	No
			Drugs	Amphetamines, Urine	negative			
				Barbiturates, Urine	negative			
				Benzodiazepines, Urine	negative			
				Cannabin., Urine	negative			
				Cocaine, Urine	negative			
				Methadone, Urine	negative			
				Opiates, Urine	negative			
			Haematology	Basophils, abs.	0.03	10^9/L		
				Basophils, %	0.5	%		
				Eosinophils, abs.	0.11	10^9/L		
				Eosinophils, %	1.7	%		
				Haemoglobin	121.0	g/L		
				Haematocrit	0.35	L/L		
				Lymphocytes, abs.	1.76	10^9/L		
				Lymphocytes, %	27.3	%		
				Monocytes, abs.	0.52	10^9/L		
				Monocytes, %	8.1	%		
				Neutrophils, abs.	4.03	10^9/L		
				Neutrophils, %	62.4	%		
				Platelets	270	10^9/L		
				Erythrocytes	3.84	10^{12}/L	L	No
				Leucocytes	6.45	10^9/L		
			Haemostasis	APTT	25.9	s		
				Prothrombin Time (INR)	0.96	N/A		
				Prothrombin Time (PT)	113.0	%		
			Urine	Beta-HCG, urine	negative			
		D02	Clinical Chemistry	ALT, 37°C	17.1	U/L		

Sign.: Significant finding; L: Result considered low; H: Result considered high; SC: Screening; BL: Baseline; D: Day; FUP: Follow-up; Part B used a cross-over design;

Output generated by program 'NIC002_L16_2_8_Laboratory_V02_0_0'

Listing 16.2.8: Study subject data
Laboratory values

Part B

Cohort	Subject ID	Visit	Type	Measurement	Result		Flagged	Sign.
		D02	Clinical Chemistry	Alkaline Phosphatase, 37°C	51.5	U/L		
				AST, 37°C	24.3	U/L		
				Bicarbonate	27.5	mmol/L		
				Bilirubin, total	5.5	umol/L		
				Urea/BUN	2.84	mmol/L		
				Calcium	2.39	mmol/L		
				Creatinine	54.0	umol/L		
				Glucose, serum	4.41	mmol/L		
				Gamma-GT, 37°C	11.2	U/L		
				Potassium	3.96	mmol/L		
				Magnesium	0.80	mmol/L		
				Sodium	138.0	mmol/L		
			Haematology	Basophils, abs.	0.02	10^9/L		
				Basophils, %	0.4	%		
				Eosinophils, abs.	0.07	10^9/L		
				Eosinophils, %	1.2	%		
				Haemoglobin	133.0	g/L		
				Haematocrit	0.39	L/L		
				Lymphocytes, abs.	1.92	10^9/L		
				Lymphocytes, %	34.2	%		
				Monocytes, abs.	0.41	10^9/L		
				Monocytes, %	7.3	%		
				Neutrophils, abs.	3.20	10^9/L		
				Neutrophils, %	56.9	%		
				Platelets	297	10^9/L		
				Erythrocytes	4.18	10^{12}/L		
				Leucocytes	5.62	10^9/L		
			Haemostasis	APTT	25.6	s		
				Prothrombin Time (INR)	0.98	N/A		
				Prothrombin Time (PT)	107.0	%		

Sign.: Significant finding; L: Result considered low; H: Result considered high; SC: Screening; BL: Baseline; D: Day; FUP: Follow-up; Part B used a cross-over design;

Output generated by program 'NIC002_L16_2_8_Laboratory_V02_0_0'

Listing 16.2.8: Study subject data
Laboratory values

Part B

Cohort	Subject ID	Visit	Type	Measurement	Result		Flagged	Sign.
	49	D04	Clinical Chemistry	ALT, 37°C	10.4	U/L		
				Alkaline Phosphatase, 37°C	85.7	U/L		
				AST, 37°C	16.6	U/L		
				Bicarbonate	24.9	mmol/L		
				Bilirubin, total	6.4	umol/L		
				Urea/BUN	2.92	mmol/L		
				Calcium	2.37	mmol/L		
				Creatinine	49.5	umol/L		
				Glucose, serum	4.43	mmol/L		
				Gamma-GT, 37°C	9.9	U/L		
				Potassium	3.93	mmol/L		
				Magnesium	0.77	mmol/L		
				Sodium	136.6	mmol/L		
			Haematology	Basophils, abs.	0.05	10^9/L		
				Basophils, %	0.8	%		
				Eosinophils, abs.	0.14	10^9/L		
				Eosinophils, %	2.2	%		
				Haemoglobin	123.0	g/L		
				Haematocrit	0.36	L/L		
				Lymphocytes, abs.	1.61	10^9/L		
				Lymphocytes, %	25.8	%		
				Monocytes, abs.	0.54	10^9/L		
				Monocytes, %	8.6	%		
				Neutrophils, abs.	3.91	10^9/L		
				Neutrophils, %	62.6	%		
				Platelets	278	10^9/L		
				Erythrocytes	4.21	10^{12}/L		
				Leucocytes	6.25	10^9/L		
			Haemostasis	APTT	28.5	s	H	No
				Prothrombin Time (INR)	1.05	N/A		
				Prothrombin Time (PT)	91.5	%		

Sign.: Significant finding; L: Result considered low; H: Result considered high; SC: Screening; BL: Baseline; D: Day; FUP: Follow-up; Part B used a cross-over design;

Output generated by program 'NIC002_L16_2_8_Laboratory_V02_0_0'

Listing 16.2.8: Study subject data
Laboratory values

Part B

Cohort	Subject ID	Visit	Type	Measurement	Result		Flagged	Sign.
		FUP	Clinical Chemistry	ALT, 37°C	14.8	U/L		
				Alkaline Phosphatase, 37°C	81.1	U/L		
				AST, 37°C	21.1	U/L		
				Bicarbonate	23.8	mmol/L		
				Bilirubin, total	7.8	umol/L		
				Urea/BUN	3.64	mmol/L		
				Calcium	2.34	mmol/L		
				Creatinine	47.8	umol/L		
				Glucose, serum	4.85	mmol/L		
				Gamma-GT, 37°C	8.6	U/L		
				Potassium	4.18	mmol/L		
				Magnesium	0.78	mmol/L		
				Sodium	134.8	mmol/L	L	No
			Haematology	Basophils, abs.	0.03	10^9/L		
				Basophils, %	0.7	%		
				Eosinophils, abs.	0.11	10^9/L		
				Eosinophils, %	2.5	%		
				Haemoglobin	121.0	g/L		
				Haematocrit	0.34	L/L	L	No
				Lymphocytes, abs.	1.28	10^9/L		
				Lymphocytes, %	29.4	%		
				Monocytes, abs.	0.40	10^9/L		
				Monocytes, %	9.2	%		
				Neutrophils, abs.	2.54	10^9/L		
				Neutrophils, %	58.2	%		
				Platelets	273	10^9/L		
				Erythrocytes	4.02	10^{12}/L		
				Leucocytes	4.36	10^9/L		
			Haemostasis	APTT	29.1	s	H	No
				Prothrombin Time (INR)	1.05	N/A		

Sign.: Significant finding; L: Result considered low; H: Result considered high; SC: Screening; BL: Baseline; D: Day; FUP: Follow-up; Part B used a cross-over design;

Output generated by program 'NIC002_L16_2_8_Laboratory_V02_0_0'

Listing 16.2.8: Study subject data
Laboratory values

Part B

Cohort	Subject ID	Visit	Type	Measurement	Result		Flagged	Sign.
		FUP	Haemostasis	Prothrombin Time (PT)	91.5	%		
			Urine	Beta-HCG, urine	negative			
	51	SC	Clinical Chemistry	ALT, 37°C	13.7	U/L		
				Alkaline Phosphatase, 37°C	33.3	U/L		
				AST, 37°C	20.5	U/L		
				Bicarbonate	29.1	mmol/L		
				Bilirubin, total	11.4	umol/L		
				Urea/BUN	3.27	mmol/L		
				Calcium	2.33	mmol/L		
				Creatinine	51.7	umol/L		
				Glucose, serum	4.90	mmol/L		
				Gamma-GT, 37°C	9.2	U/L		
				Potassium	4.51	mmol/L		
				Creatinine Clearance MDRD	119	ml/min/1.73m		
				Magnesium	0.82	mmol/L		
				Sodium	138.8	mmol/L		
			Drugs	Amphetamines, Urine	negative			
				Barbiturates, Urine	negative			
				Benzodiazepines, Urine	negative			
				Cannabin., Urine	negative			
				Cocaine, Urine	negative			
				Methadone, Urine	negative			
				Opiates, Urine	negative			
			Haematology	Basophils, abs.	0.02	10^9/L		
				Basophils, %	0.5	%		
				Eosinophils, abs.	0.09	10^9/L		
				Eosinophils, %	2.0	%		
				Haemoglobin	130.0	g/L		
				Haematocrit	0.38	L/L		
				Lymphocytes, abs.	1.72	10^9/L		

Sign.: Significant finding; L: Result considered low; H: Result considered high; SC: Screening; BL: Baseline; D: Day; FUP: Follow-up; Part B used a cross-over design;

Output generated by program 'NIC002_L16_2_8_Laboratory_V02_0_0'

Listing 16.2.8: Study subject data
Laboratory values

Part B

Cohort	Subject ID	Visit	Type	Measurement	Result		Flagged	Sign.
	51	SC	Haematology	Lymphocytes, %	38.8	%		
				Monocytes, abs.	0.31	10^9/L		
				Monocytes, %	7.0	%		
				Neutrophils, abs.	2.29	10^9/L		
				Neutrophils, %	51.7	%		
				Platelets	270	10^9/L		
				Erythrocytes	4.14	10^{12}/L		
				Leucocytes	4.43	10^9/L		
			Haemostasis	APTT	26.1	s		
				Prothrombin Time (INR)	1.08	N/A		
				Prothrombin Time (PT)	84.9	%		
			Infectious Diseases	HBs-Ag (Hep. B Surf. Ag)	negative	N/A		
				Anti-HCV (Hep. C-AB)	non-reactive	N/A		
				HIV 1+2, AG/AB	negative	N/A		
			Urine	Bilirubin, urine (Stix)	negative			
				Blood (Ery/Hb), urine (Stix)	trace-intact		H	No
				Glucose, urine (Stix)	negative			
				Beta-HCG, urine	negative			
				Ketone, urine (Stix)	negative			
				Leucocytes, urine (Stix)	negative			
				Nitrite, urine (Stix)	negative			
				pH, urine (Stix)	6.0	neg.log[H+]		
				Protein, total, urine (Stix)	negative			
				Bacteria, Sediment	negative			
				Carbonate, Sediment	negative			
				Epithelial Cells, Sediment	3	per field	L	No
				Erythrocytes, Sediment	0	per field		
				Casts granul., Sediment	0	per field		
				Casts hyaline, Sediment	0	per field		
				Leucocytes, Sediment	0	per field		
				Oxalate, Sediment	negative			

Sign.: Significant finding; L: Result considered low; H: Result considered high; SC: Screening; BL: Baseline; D: Day; FUP: Follow-up; Part B used a cross-over design;

Output generated by program 'NIC002_L16_2_8_Laboratory_V02_0_0'

Listing 16.2.8: Study subject data
Laboratory values

Part B

Cohort	Subject ID	Visit	Type	Measurement	Result	Flagged	Sign.
	51	SC	Urine	Specific Gravity	1.010		
				Mucus, Sediment	negative		
				Triple Phosphate, Sediment	negative		
				Urates, Sediment	negative		
				Urobilinogen, urine (Stix)	0.2 mg/dl		
		BL	Clinical Chemistry	ALT, 37°C	11.6 U/L		
				Alkaline Phosphatase, 37°C	34.2 U/L		
				AST, 37°C	17.1 U/L		
				Bicarbonate	27.5 mmol/L		
				Bilirubin, total	9.9 umol/L		
				Urea/BUN	3.82 mmol/L		
				Calcium	2.37 mmol/L		
				Creatinine	51.0 umol/L		
				Glucose, serum	5.51 mmol/L		
				Gamma-GT, 37°C	9.2 U/L		
				Potassium	4.61 mmol/L		
				Magnesium	0.79 mmol/L		
				Sodium	136.9 mmol/L		
			Drugs	Amphetamines, Urine	negative		
				Barbiturates, Urine	negative		
				Benzodiazepines, Urine	negative		
				Cannabin., Urine	negative		
				Cocaine, Urine	negative		
				Methadone, Urine	negative		
				Opiates, Urine	negative		
			Haematology	Basophils, abs.	0.03 10^9/L		
				Basophils, %	0.6 %		
				Eosinophils, abs.	0.15 10^9/L		
				Eosinophils, %	3.2 %		
				Haemoglobin	131.0 g/L		

Sign.: Significant finding; L: Result considered low; H: Result considered high; SC: Screening; BL: Baseline; D: Day; FUP: Follow-up; Part B used a cross-over design;

Output generated by program 'NIC002_L16_2_8_Laboratory_V02_0_0'

Listing 16.2.8: Study subject data
Laboratory values

Part B

Cohort	Subject ID	Visit	Type	Measurement	Result		Flagged	Sign.
		BL	Haematology	Haematocrit	0.37	L/L		
				Lymphocytes, abs.	2.01	10^9/L		
				Lymphocytes, %	42.6	%		
				Monocytes, abs.	0.39	10^9/L		
				Monocytes, %	8.3	%		
				Neutrophils, abs.	2.14	10^9/L		
				Neutrophils, %	45.3	%		
				Platelets	258	10^9/L		
				Erythrocytes	4.16	10^{12}/L		
				Leucocytes	4.72	10^9/L		
			Haemostasis	APTT	25.2	s		
				Prothrombin Time (INR)	1.05	N/A		
				Prothrombin Time (PT)	91.5	%		
			Urine	Beta-HCG, urine	negative			
		D02	Clinical Chemistry	ALT, 37°C	11.0	U/L		
				Alkaline Phosphatase, 37°C	36.4	U/L		
				AST, 37°C	18.5	U/L		
				Bicarbonate	27.1	mmol/L		
				Bilirubin, total	10.7	umol/L		
				Urea/BUN	4.02	mmol/L		
				Calcium	2.37	mmol/L		
				Creatinine	50.9	umol/L		
				Glucose, serum	4.62	mmol/L		
				Gamma-GT, 37°C	9.3	U/L		
				Potassium	4.44	mmol/L		
				Magnesium	0.80	mmol/L		
				Sodium	137.5	mmol/L		
			Haematology	Basophils, abs.	0.03	10^9/L		
				Basophils, %	0.5	%		
				Eosinophils, abs.	0.11	10^9/L		

Sign.: Significant finding; L: Result considered low; H: Result considered high; SC: Screening; BL: Baseline; D: Day; FUP: Follow-up; Part B used a cross-over design;

Output generated by program 'NIC002_L16_2_8_Laboratory_V02_0_0'

Listing 16.2.8: Study subject data
Laboratory values

Part B

Cohort	Subject ID	Visit	Type	Measurement	Result		Flagged	Sign.
		D02	Haematology	Eosinophils, %	2.0	%		
				Haemoglobin	138.0	g/L		
				Haematocrit	0.39	L/L		
				Lymphocytes, abs.	2.09	10^9/L		
				Lymphocytes, %	37.2	%		
				Monocytes, abs.	0.40	10^9/L		
				Monocytes, %	7.1	%		
				Neutrophils, abs.	2.99	10^9/L		
				Neutrophils, %	53.2	%		
				Platelets	291	10^9/L		
				Erythrocytes	4.32	10^{12}/L		
				Leucocytes	5.62	10^9/L		
			Haemostasis	APTT	26.3	s		
				Prothrombin Time (INR)	1.07	N/A		
				Prothrombin Time (PT)	87.0	%		
	76	D04	Clinical Chemistry	ALT, 37°C	10.2	U/L		
				Alkaline Phosphatase, 37°C	49.4	U/L		
				AST, 37°C	16.0	U/L		
				Bicarbonate	27.3	mmol/L		
				Bilirubin, total	12.2	umol/L		
				Urea/BUN	5.52	mmol/L		
				Calcium	2.41	mmol/L		
				Creatinine	51.9	umol/L		
				Glucose, serum	4.33	mmol/L		
				Gamma-GT, 37°C	11.0	U/L		
				Potassium	4.22	mmol/L		
				Magnesium	0.76	mmol/L	L	No
				Sodium	135.0	mmol/L	L	No
			Haematology	Basophils, abs.	0.03	10^9/L		
				Basophils, %	0.4	%		

Sign.: Significant finding; L: Result considered low; H: Result considered high; SC: Screening; BL: Baseline; D: Day; FUP: Follow-up; Part B used a cross-over design;

Output generated by program 'NIC002_L16_2_8_Laboratory_V02_0_0'

Listing 16.2.8: Study subject data
Laboratory values

Part B

Cohort	Subject ID	Visit	Type	Measurement	Result		Flagged	Sign.
	76	D04	Haematology	Eosinophils, abs.	0.29	10^9/L		
				Eosinophils, %	4.1	%		
				Haemoglobin	134.0	g/L		
				Haematocrit	0.39	L/L		
				Lymphocytes, abs.	2.59	10^9/L		
				Lymphocytes, %	37.1	%		
				Monocytes, abs.	0.58	10^9/L		
				Monocytes, %	8.3	%		
				Neutrophils, abs.	3.50	10^9/L		
				Neutrophils, %	50.1	%		
				Platelets	333	10^9/L		
				Erythrocytes	4.26	10^{12}/L		
				Leucocytes	6.99	10^9/L		
			Haemostasis	APTT	26.8	s		
				Prothrombin Time (INR)	1.02	N/A		
				Prothrombin Time (PT)	97.2	%		
		FUP	Clinical Chemistry	ALT, 37°C	11.8	U/L		
				Alkaline Phosphatase, 37°C	51.2	U/L		
				AST, 37°C	18.3	U/L		
				Bicarbonate	28.2	mmol/L		
				Bilirubin, total	10.8	umol/L		
				Urea/BUN	4.76	mmol/L		
				Calcium	2.29	mmol/L		
				Creatinine	48.8	umol/L		
				Glucose, serum	5.06	mmol/L		
				Gamma-GT, 37°C	9.5	U/L		
				Potassium	4.29	mmol/L		
				Magnesium	0.78	mmol/L		
				Sodium	135.8	mmol/L	L	No
			Haematology	Basophils, abs.	0.03	10^9/L		

Sign.: Significant finding; L: Result considered low; H: Result considered high; SC: Screening; BL: Baseline; D: Day; FUP: Follow-up; Part B used a cross-over design;

Output generated by program 'NIC002_L16_2_8_Laboratory_V02_0_0'

Listing 16.2.8: Study subject data
Laboratory values

Part B

Cohort	Subject ID	Visit	Type	Measurement	Result		Flagged	Sign.
		FUP	Haematology	Basophils, %	0.4	%		
				Eosinophils, abs.	0.43	10^9/L		
				Eosinophils, %	5.3	%		
				Haemoglobin	120.0	g/L		
				Haematocrit	0.34	L/L	L	No
				Lymphocytes, abs.	3.13	10^9/L	H	No
				Lymphocytes, %	38.4	%		
				Monocytes, abs.	0.70	10^9/L		
				Monocytes, %	8.6	%		
				Neutrophils, abs.	3.86	10^9/L		
				Neutrophils, %	47.3	%		
				Platelets	330	10^9/L		
				Erythrocytes	3.80	10^{12}/L	L	No
				Leucocytes	8.15	10^9/L		
			Haemostasis	APTT	26.1	s		
				Prothrombin Time (INR)	1.02	N/A		
				Prothrombin Time (PT)	97.2	%		

Sign.: Significant finding; L: Result considered low; H: Result considered high; SC: Screening; BL: Baseline; D: Day; FUP: Follow-up; Part B used a cross-over design;

Output generated by program 'NIC002_L16_2_8_Laboratory_V02_0_0'

Listing 16.2.8: Study subject data
Laboratory values

Part C

Cohort	Subject ID	Visit	Type	Measurement	Result		Flagged	Sign.
Group 1	54	SC	Clinical Chemistry	ALT, 37°C	24.7	U/L		
				Alkaline Phosphatase, 37°C	69.5	U/L		
				AST, 37°C	23.5	U/L		
				Bicarbonate	26.8	mmol/L		
				Bilirubin, total	7.1	umol/L		
				Urea/BUN	3.77	mmol/L		
				Calcium	2.40	mmol/L		
				Creatinine	57.9	umol/L		
				Glucose, serum	5.08	mmol/L		
				Gamma-GT, 37°C	16.0	U/L		
				Potassium	4.63	mmol/L		
				Creatinine Clearance MDRD	103	ml/min/1.73m		
				Magnesium	0.82	mmol/L		
				Sodium	138.6	mmol/L		
			Drugs	Amphetamines, Urine	negative			
				Barbiturates, Urine	negative			
				Benzodiazepines, Urine	negative			
				Cannabin., Urine	negative			
				Cocaine, Urine	negative			
				Methadone, Urine	negative			
				Opiates, Urine	negative			
			Haematology	Basophils, abs.	0.03	10^9/L		
				Basophils, %	0.4	%		
				Eosinophils, abs.	0.44	10^9/L	H	No
				Eosinophils, %	6.5	%		
				Haemoglobin	140.0	g/L		
				Haematocrit	0.40	L/L		
				Lymphocytes, abs.	1.80	10^9/L		
				Lymphocytes, %	26.5	%		
				Monocytes, abs.	0.46	10^9/L		

Sign.: Significant finding; L: Result considered low; H: Result considered high; SC: Screening; BL: Baseline; D: Day; FUP: Follow-up;

Output generated by program 'NIC002_L16_2_8_Laboratory_V02_0_0'

Listing 16.2.8: Study subject data
Laboratory values

Part C

Cohort	Subject ID	Visit	Type	Measurement	Result		Flagged	Sign.
Group 1	54	SC	Haematology	Monocytes, %	6.8	%		
				Neutrophils, abs.	4.07	10^9/L		
				Neutrophils, %	59.8	%		
				Platelets	296	10^9/L		
				Erythrocytes	4.28	10^{12}/L		
				Leucocytes	6.80	10^9/L		
			Haemostasis	APTT	24.6	s		
				Prothrombin Time (INR)	0.98	N/A		
				Prothrombin Time (PT)	107.0	%		
			Infectious Diseases	HBs-Ag (Hep. B Surf. Ag)	negative	N/A		
				Anti-HCV (Hep. C-AB)	non-reactive	N/A		
				HIV 1+2, AG/AB	negative	N/A		
			Urine	Bilirubin, urine (Stix)	negative			
				Blood (Ery/Hb), urine (Stix)	1+		H	No
				Glucose, urine (Stix)	negative			
				Beta-HCG, urine	negative			
				Ketone, urine (Stix)	negative			
				Leucocytes, urine (Stix)	negative			
				Nitrite, urine (Stix)	negative			
				pH, urine (Stix)	5.5	neg.log[H+]		
				Protein, total, urine (Stix)	negative			
				Bacteria, Sediment	positive		H	No
				Carbonate, Sediment	negative			
				Epithelial Cells, Sediment	1	per field	L	No
				Erythrocytes, Sediment	0	per field		
				Casts granul., Sediment	0	per field		
				Casts hyaline, Sediment	0	per field		
				Leucocytes, Sediment	1	per field		
				Oxalate, Sediment	negative			
				Specific Gravity	<=1.005			
				Mucus, Sediment	negative			

Sign.: Significant finding; L: Result considered low; H: Result considered high; SC: Screening; BL: Baseline; D: Day; FUP: Follow-up;

Output generated by program 'NIC002_L16_2_8_Laboratory_V02_0_0'

Listing 16.2.8: Study subject data
Laboratory values

Part C

Cohort	Subject ID	Visit	Type	Measurement	Result		Flagged	Sign.
Group 1	54	SC	Urine	Triple Phosphate, Sediment	negative			
				Urates, Sediment	negative			
				Urobilinogen, urine (Stix)	0.2	mg/dl		
		BL	Clinical Chemistry	ALT, 37°C	28.7	U/L		
				Alkaline Phosphatase, 37°C	66.2	U/L		
				AST, 37°C	26.2	U/L		
				Bicarbonate	26.2	mmol/L		
				Bilirubin, total	6.1	umol/L		
				Urea/BUN	2.64	mmol/L	L	No
				Calcium	2.30	mmol/L		
				Creatinine	56.7	umol/L		
				Glucose, serum	4.93	mmol/L		
				Gamma-GT, 37°C	12.4	U/L		
				Potassium	3.99	mmol/L		
				Magnesium	0.75	mmol/L	L	No
				Sodium	137.5	mmol/L		
			Drugs	Amphetamines, Urine	negative			
				Barbiturates, Urine	negative			
				Benzodiazepines, Urine	negative			
				Cannabin., Urine	negative			
				Cocaine, Urine	negative			
				Methadone, Urine	negative			
				Opiates, Urine	negative			
			Haematology	Basophils, abs.	0.05	10^9/L		
				Basophils, %	0.6	%		
				Eosinophils, abs.	0.56	10^9/L	H	No
				Eosinophils, %	6.9	%		
				Haemoglobin	135.0	g/L		
				Haematocrit	0.39	L/L		
				Lymphocytes, abs.	2.57	10^9/L		

Sign.: Significant finding; L: Result considered low; H: Result considered high; SC: Screening; BL: Baseline; D: Day; FUP: Follow-up;

Output generated by program 'NIC002_L16_2_8_Laboratory_V02_0_0'

Listing 16.2.8: Study subject data
Laboratory values

Part C

Cohort	Subject ID	Visit	Type	Measurement	Result		Flagged	Sign.
		BL	Haematology	Lymphocytes, %	31.7	%		
				Monocytes, abs.	0.73	10^9/L		
				Monocytes, %	9.0	%		
				Neutrophils, abs.	4.21	10^9/L		
				Neutrophils, %	51.8	%		
				Platelets	286	10^9/L		
				Erythrocytes	4.17	10^{12}/L		
				Leucocytes	8.12	10^9/L		
			Haemostasis	APTT	25.3	s		
				Prothrombin Time (INR)	0.98	N/A		
				Prothrombin Time (PT)	107.0	%		
			Urine	Beta-HCG, urine	negative			
		D03	Clinical Chemistry	ALT, 37°C	28.3	U/L		
				Alkaline Phosphatase, 37°C	64.9	U/L		
				AST, 37°C	23.3	U/L		
				Bicarbonate	24.7	mmol/L		
				Bilirubin, total	8.4	umol/L		
				Urea/BUN	3.26	mmol/L		
				Calcium	2.35	mmol/L		
				Creatinine	56.4	umol/L		
				Glucose, serum	4.21	mmol/L		
				Gamma-GT, 37°C	13.2	U/L		
				Potassium	4.29	mmol/L		
				Magnesium	0.81	mmol/L		
				Sodium	137.3	mmol/L		
			Haematology	Basophils, abs.	0.04	10^9/L		
				Basophils, %	0.5	%		
				Eosinophils, abs.	0.58	10^9/L	H	No
				Eosinophils, %	7.1	%		
				Haemoglobin	142.0	g/L		

Sign.: Significant finding; L: Result considered low; H: Result considered high; SC: Screening; BL: Baseline; D: Day; FUP: Follow-up;

Output generated by program 'NIC002_L16_2_8_Laboratory_V02_0_0'

Listing 16.2.8: Study subject data
Laboratory values

Part C

Cohort	Subject ID	Visit	Type	Measurement	Result		Flagged	Sign.
		D03	Haematology	Haematocrit	0.41	L/L		
				Lymphocytes, abs.	2.74	10^9/L		
				Lymphocytes, %	33.3	%		
				Monocytes, abs.	0.69	10^9/L		
				Monocytes, %	8.4	%		
				Neutrophils, abs.	4.17	10^9/L		
				Neutrophils, %	50.7	%		
				Platelets	299	10^9/L		
				Erythrocytes	4.42	10^{12}/L		
				Leucocytes	8.22	10^9/L		
			Haemostasis	APTT	26.1	s		
				Prothrombin Time (INR)	1.02	N/A		
				Prothrombin Time (PT)	96.3	%		
		D08	Clinical Chemistry	ALT, 37°C	23.7	U/L		
				Alkaline Phosphatase, 37°C	60.9	U/L		
				AST, 37°C	20.7	U/L		
				Bicarbonate	25.0	mmol/L		
				Bilirubin, total	11.4	umol/L		
				Urea/BUN	3.30	mmol/L		
				Calcium	2.35	mmol/L		
				Creatinine	55.9	umol/L		
				Glucose, serum	4.48	mmol/L		
				Gamma-GT, 37°C	10.8	U/L		
				Potassium	3.99	mmol/L		
				Magnesium	0.82	mmol/L		
				Sodium	137.7	mmol/L		
			Haematology	Basophils, abs.	0.04	10^9/L		
				Basophils, %	0.5	%		
				Eosinophils, abs.	0.51	10^9/L	H	No
				Eosinophils, %	6.4	%		

Sign.: Significant finding; L: Result considered low; H: Result considered high; SC: Screening; BL: Baseline; D: Day; FUP: Follow-up;

Output generated by program 'NIC002_L16_2_8_Laboratory_V02_0_0'

Listing 16.2.8: Study subject data
Laboratory values

Part C

Cohort	Subject ID	Visit	Type	Measurement	Result		Flagged	Sign.
		D08	Haematology	Haemoglobin	134.0	g/L		
				Haematocrit	0.38	L/L		
				Lymphocytes, abs.	2.62	10^9/L		
				Lymphocytes, %	32.6	%		
				Monocytes, abs.	0.60	10^9/L		
				Monocytes, %	7.5	%		
				Neutrophils, abs.	4.26	10^9/L		
				Neutrophils, %	53.0	%		
				Platelets	297	10^9/L		
				Erythrocytes	4.04	10^{12}/L		
				Leucocytes	8.03	10^9/L		
			Haemostasis	APTT	25.7	s		
				Prothrombin Time (INR)	1.00	N/A		
				Prothrombin Time (PT)	101.5	%		
		FUP	Clinical Chemistry	ALT, 37°C	28.4	U/L		
				Alkaline Phosphatase, 37°C	62.7	U/L		
				AST, 37°C	25.8	U/L		
				Bicarbonate	27.0	mmol/L		
				Bilirubin, total	8.7	umol/L		
				Urea/BUN	2.83	mmol/L		
				Calcium	2.36	mmol/L		
				Creatinine	51.5	umol/L		
				Glucose, serum	3.72	mmol/L	L	No
				Gamma-GT, 37°C	10.4	U/L		
				Potassium	4.12	mmol/L		
				Magnesium	0.79	mmol/L		
				Sodium	136.4	mmol/L		
			Haematology	Basophils, abs.	0.05	10^9/L		
				Basophils, %	0.7	%		
				Eosinophils, abs.	0.59	10^9/L	H	No

Sign.: Significant finding; L: Result considered low; H: Result considered high; SC: Screening; BL: Baseline; D: Day; FUP: Follow-up;

Output generated by program 'NIC002_L16_2_8_Laboratory_V02_0_0'

Listing 16.2.8: Study subject data
Laboratory values

Part C

Cohort	Subject ID	Visit	Type	Measurement	Result		Flagged	Sign.
		FUP	Haematology	Eosinophils, %	8.3	%	H	No
				Haemoglobin	127.0	g/L		
				Haematocrit	0.37	L/L		
				Lymphocytes, abs.	1.82	10^9/L		
				Lymphocytes, %	25.7	%		
				Monocytes, abs.	0.58	10^9/L		
				Monocytes, %	8.2	%		
				Neutrophils, abs.	4.04	10^9/L		
				Neutrophils, %	57.1	%		
				Platelets	316	10^9/L		
				Erythrocytes	3.92	10^{12}/L	L	No
				Leucocytes	7.08	10^9/L		
			Haemostasis	APTT	24.8	s		
				Prothrombin Time (INR)	0.98	N/A		
				Prothrombin Time (PT)	111.0	%		
	63	SC	Clinical Chemistry	ALT, 37°C	10.1	U/L		
				Alkaline Phosphatase, 37°C	39.6	U/L		
				AST, 37°C	16.5	U/L		
				Bicarbonate	25.9	mmol/L		
				Bilirubin, total	6.4	umol/L		
				Urea/BUN	3.03	mmol/L		
				Calcium	2.41	mmol/L		
				Creatinine	57.7	umol/L		
				Glucose, serum	4.54	mmol/L		
				Gamma-GT, 37°C	8.6	U/L		
				Potassium	3.65	mmol/L		
				Creatinine Clearance MDRD	105	ml/min/1.73m		
				Magnesium	0.82	mmol/L		
				Sodium	137.7	mmol/L		
			Drugs	Amphetamines, Urine	negative			

Sign.: Significant finding; L: Result considered low; H: Result considered high; SC: Screening; BL: Baseline; D: Day; FUP: Follow-up;

Output generated by program 'NIC002_L16_2_8_Laboratory_V02_0_0'

Listing 16.2.8: Study subject data
Laboratory values

Part C

Cohort	Subject ID	Visit	Type	Measurement	Result		Flagged	Sign.
	63	SC	Drugs	Barbiturates, Urine	negative			
				Benzodiazepines, Urine	negative			
				Cannabin., Urine	negative			
				Cocaine, Urine	negative			
				Methadone, Urine	negative			
				Opiates, Urine	negative			
			Haematology	Basophils, abs.	0.05	10^9/L		
				Basophils, %	0.8	%		
				Eosinophils, abs.	0.46	10^9/L	H	No
				Eosinophils, %	7.8	%		
				Haemoglobin	138.0	g/L		
				Haematocrit	0.39	L/L		
				Lymphocytes, abs.	2.56	10^9/L		
				Lymphocytes, %	43.4	%		
				Monocytes, abs.	0.39	10^9/L		
				Monocytes, %	6.6	%		
				Neutrophils, abs.	2.44	10^9/L		
				Neutrophils, %	41.4	%		
				Platelets	286	10^9/L		
				Erythrocytes	4.44	10^{12}/L		
				Leucocytes	5.90	10^9/L		
			Haemostasis	APTT	28.0	s		
				Prothrombin Time (INR)	1.04	N/A		
				Prothrombin Time (PT)	93.9	%		
			Infectious Diseases	HBs-Ag (Hep. B Surf. Ag)	negative	N/A		
				Anti-HCV (Hep. C-AB)	non-reactive	N/A		
				HIV 1+2, AG/AB	negative	N/A		
			Urine	Bilirubin, urine (Stix)	negative			
				Blood (Ery/Hb), urine (Stix)	negative			
				Glucose, urine (Stix)	negative			
				Beta-HCG, urine	negative			

Sign.: Significant finding; L: Result considered low; H: Result considered high; SC: Screening; BL: Baseline; D: Day; FUP: Follow-up;

Output generated by program 'NIC002_L16_2_8_Laboratory_V02_0_0'

Listing 16.2.8: Study subject data
Laboratory values

Part C

Cohort	Subject ID	Visit	Type	Measurement	Result		Flagged	Sign.
	63	SC	Urine	Ketone, urine (Stix)	negative			
				Leucocytes, urine (Stix)	negative			
				Nitrite, urine (Stix)	negative			
				pH, urine (Stix)	5.5	neg.log[H+]		
				Protein, total, urine (Stix)	negative			
				Specific Gravity	1.020			
				Urobilinogen, urine (Stix)	0.2	mg/dl		
		BL	Clinical Chemistry	ALT, 37°C	11.8	U/L		
				Alkaline Phosphatase, 37°C	39.2	U/L		
				AST, 37°C	18.5	U/L		
				Bicarbonate	24.6	mmol/L		
				Bilirubin, total	9.9	umol/L		
				Urea/BUN	1.80	mmol/L	L	No
				Calcium	2.35	mmol/L		
				Creatinine	58.5	umol/L		
				Glucose, serum	4.32	mmol/L		
				Gamma-GT, 37°C	7.8	U/L		
				Potassium	3.27	mmol/L	L	No
				Magnesium	0.79	mmol/L		
				Sodium	134.9	mmol/L	L	No
			Drugs	Amphetamines, Urine	negative			
				Barbiturates, Urine	negative			
				Benzodiazepines, Urine	negative			
				Cannabin., Urine	negative			
				Cocaine, Urine	negative			
				Methadone, Urine	negative			
				Opiates, Urine	negative			
			Haematology	Basophils, abs.	0.04	10^9/L		
				Basophils, %	0.6	%		
				Eosinophils, abs.	0.41	10^9/L		

Sign.: Significant finding; L: Result considered low; H: Result considered high; SC: Screening; BL: Baseline; D: Day; FUP: Follow-up;

Output generated by program 'NIC002_L16_2_8_Laboratory_V02_0_0'

Listing 16.2.8: Study subject data
Laboratory values

Part C

Cohort	Subject ID	Visit	Type	Measurement	Result		Flagged	Sign.
		BL	Haematology	Eosinophils, %	6.0	%		
				Haemoglobin	134.0	g/L		
				Haematocrit	0.39	L/L		
				Lymphocytes, abs.	2.86	10^9/L		
				Lymphocytes, %	42.1	%		
				Monocytes, abs.	0.45	10^9/L		
				Monocytes, %	6.6	%		
				Neutrophils, abs.	3.03	10^9/L		
				Neutrophils, %	44.7	%		
				Platelets	279	10^9/L		
				Erythrocytes	4.40	10^{12}/L		
				Leucocytes	6.79	10^9/L		
			Haemostasis	APTT	29.2	s	H	No
				Prothrombin Time (INR)	1.06	N/A		
				Prothrombin Time (PT)	89.2	%		
			Urine	Beta-HCG, urine	negative			
		D03	Clinical Chemistry	ALT, 37°C	10.1	U/L		
				Alkaline Phosphatase, 37°C	32.2	U/L		
				AST, 37°C	16.1	U/L		
				Bicarbonate	25.6	mmol/L		
				Bilirubin, total	12.6	umol/L		
				Urea/BUN	3.18	mmol/L		
				Calcium	2.39	mmol/L		
				Creatinine	49.8	umol/L		
				Glucose, serum	3.37	mmol/L	L	No
				Gamma-GT, 37°C	7.4	U/L		
				Potassium	3.74	mmol/L		
				Magnesium	0.80	mmol/L		
				Sodium	138.7	mmol/L		
			Haematology	Basophils, abs.	0.03	10^9/L		

Sign.: Significant finding; L: Result considered low; H: Result considered high; SC: Screening; BL: Baseline; D: Day; FUP: Follow-up;

Output generated by program 'NIC002_L16_2_8_Laboratory_V02_0_0'

Listing 16.2.8: Study subject data
Laboratory values

Part C

Cohort	Subject ID	Visit	Type	Measurement	Result		Flagged	Sign.
		D03	Haematology	Basophils, %	0.5	%		
				Eosinophils, abs.	0.36	10^9/L		
				Eosinophils, %	6.1	%		
				Haemoglobin	139.0	g/L		
				Haematocrit	0.41	L/L		
				Lymphocytes, abs.	2.48	10^9/L		
				Lymphocytes, %	42.0	%		
				Monocytes, abs.	0.32	10^9/L		
				Monocytes, %	5.4	%		
				Neutrophils, abs.	2.72	10^9/L		
				Neutrophils, %	46.0	%		
				Platelets	270	10^9/L		
				Erythrocytes	4.52	10^{12}/L		
				Leucocytes	5.91	10^9/L		
			Haemostasis	APTT	30.3	s	H	No
				Prothrombin Time (INR)	1.09	N/A		
				Prothrombin Time (PT)	82.8	%		
		D08	Clinical Chemistry	ALT, 37°C	16.1	U/L		
				Alkaline Phosphatase, 37°C	33.0	U/L		
				AST, 37°C	21.8	U/L		
				Bicarbonate	23.9	mmol/L		
				Bilirubin, total	14.1	umol/L		
				Urea/BUN	3.18	mmol/L		
				Calcium	2.37	mmol/L		
				Creatinine	54.6	umol/L		
				Glucose, serum	3.71	mmol/L	L	No
				Gamma-GT, 37°C	7.8	U/L		
				Potassium	3.83	mmol/L		
				Magnesium	0.85	mmol/L		
				Sodium	137.6	mmol/L		

Sign.: Significant finding; L: Result considered low; H: Result considered high; SC: Screening; BL: Baseline; D: Day; FUP: Follow-up;

Output generated by program 'NIC002_L16_2_8_Laboratory_V02_0_0'

Listing 16.2.8: Study subject data
Laboratory values

Part C

Cohort	Subject ID	Visit	Type	Measurement	Result		Flagged	Sign.
		D08	Haematology	Basophils, abs.	0.03	10^9/L		
				Basophils, %	0.6	%		
				Eosinophils, abs.	0.41	10^9/L		
				Eosinophils, %	7.9	%		
				Haemoglobin	132.0	g/L		
				Haematocrit	0.38	L/L		
				Lymphocytes, abs.	2.13	10^9/L		
				Lymphocytes, %	40.8	%		
				Monocytes, abs.	0.33	10^9/L		
				Monocytes, %	6.3	%		
				Neutrophils, abs.	2.32	10^9/L		
				Neutrophils, %	44.4	%		
				Platelets	236	10^9/L		
				Erythrocytes	4.28	10^{12}/L		
				Leucocytes	5.22	10^9/L		
			Haemostasis	APTT	31.1	s	H	No
				Prothrombin Time (INR)	1.08	N/A		
				Prothrombin Time (PT)	84.9	%		
		FUP	Clinical Chemistry	ALT, 37°C	14.5	U/L		
				Alkaline Phosphatase, 37°C	34.6	U/L		
				AST, 37°C	18.1	U/L		
				Bicarbonate	25.2	mmol/L		
				Bilirubin, total	10.3	umol/L		
				Urea/BUN	3.36	mmol/L		
				Calcium	2.39	mmol/L		
				Creatinine	58.9	umol/L		
				Glucose, serum	3.58	mmol/L	L	No
				Gamma-GT, 37°C	7.3	U/L		
				Potassium	4.27	mmol/L		
				Magnesium	0.83	mmol/L		

Sign.: Significant finding; L: Result considered low; H: Result considered high; SC: Screening; BL: Baseline; D: Day; FUP: Follow-up;

Output generated by program 'NIC002_L16_2_8_Laboratory_V02_0_0'

Listing 16.2.8: Study subject data
Laboratory values

Part C

Cohort	Subject ID	Visit	Type	Measurement	Result		Flagged	Sign.
		FUP	Clinical Chemistry	Sodium	136.5	mmol/L		
			Haematology	Basophils, abs.	0.03	10^9/L		
				Basophils, %	0.6	%		
				Eosinophils, abs.	0.36	10^9/L		
				Eosinophils, %	6.8	%		
				Haemoglobin	124.0	g/L		
				Haematocrit	0.36	L/L		
				Lymphocytes, abs.	1.79	10^9/L		
				Lymphocytes, %	33.8	%		
				Monocytes, abs.	0.50	10^9/L		
				Monocytes, %	9.4	%		
				Neutrophils, abs.	2.62	10^9/L		
				Neutrophils, %	49.4	%		
				Platelets	225	10^9/L		
				Erythrocytes	4.05	10^{12}/L		
				Leucocytes	5.30	10^9/L		
			Haemostasis	APTT	27.7	s		
				Prothrombin Time (INR)	0.99	N/A		
				Prothrombin Time (PT)	107.8	%		
	67	SC	Clinical Chemistry	ALT, 37°C	11.5	U/L		
				Alkaline Phosphatase, 37°C	60.7	U/L		
				AST, 37°C	15.7	U/L		
				Bicarbonate	27.4	mmol/L		
				Bilirubin, total	13.6	umol/L		
				Urea/BUN	2.99	mmol/L		
				Calcium	2.30	mmol/L		
				Creatinine	56.7	umol/L		
				Glucose, serum	4.82	mmol/L		
				Gamma-GT, 37°C	9.5	U/L		
				Potassium	3.98	mmol/L		

Sign.: Significant finding; L: Result considered low; H: Result considered high; SC: Screening; BL: Baseline; D: Day; FUP: Follow-up;

Output generated by program 'NIC002_L16_2_8_Laboratory_V02_0_0'

Listing 16.2.8: Study subject data
Laboratory values

Part C

Cohort	Subject ID	Visit	Type	Measurement	Result		Flagged	Sign.
	67	SC	Clinical Chemistry	Creatinine Clearance MDRD	106	ml/min/1.73m		
				Magnesium	0.85	mmol/L		
				Sodium	138.5	mmol/L		
			Drugs	Amphetamines, Urine	negative			
				Barbiturates, Urine	negative			
				Benzodiazepines, Urine	negative			
				Cannabin., Urine	negative			
				Cocaine, Urine	negative			
				Methadone, Urine	negative			
				Opiates, Urine	negative			
			Haematology	Basophils, abs.	0.05	10^9/L		
				Basophils, %	1.3	%		
				Eosinophils, abs.	0.05	10^9/L		
				Eosinophils, %	1.3	%		
				Haemoglobin	122.0	g/L		
				Haematocrit	0.36	L/L		
				Lymphocytes, abs.	1.54	10^9/L		
				Lymphocytes, %	38.8	%		
				Monocytes, abs.	0.24	10^9/L	L	No
				Monocytes, %	6.0	%		
				Neutrophils, abs.	2.09	10^9/L		
				Neutrophils, %	52.6	%		
				Platelets	277	10^9/L		
				Erythrocytes	3.81	10^{12}/L	L	No
				Leucocytes	3.97	10^9/L		
			Haemostasis	APTT	27.7	s		
				Prothrombin Time (INR)	1.05	N/A		
				Prothrombin Time (PT)	91.5	%		
			Infectious Diseases	HBs-Ag (Hep. B Surf. Ag)	negative	N/A		
				Anti-HCV (Hep. C-AB)	non-reactive	N/A		
				HIV 1+2, AG/AB	negative	N/A		

Sign.: Significant finding; L: Result considered low; H: Result considered high; SC: Screening; BL: Baseline; D: Day; FUP: Follow-up;

Output generated by program 'NIC002_L16_2_8_Laboratory_V02_0_0'

Listing 16.2.8: Study subject data
Laboratory values

Part C

Cohort	Subject ID	Visit	Type	Measurement	Result		Flagged	Sign.
	67	SC	Urine	Bilirubin, urine (Stix)	negative			
				Blood (Ery/Hb), urine (Stix)	trace-intact		H	No
				Glucose, urine (Stix)	negative			
				Beta-HCG, urine	negative			
				Ketone, urine (Stix)	negative			
				Leucocytes, urine (Stix)	negative			
				Nitrite, urine (Stix)	negative			
				pH, urine (Stix)	6.5	neg.log[H+]		
				Protein, total, urine (Stix)	negative			
				Bacteria, Sediment	negative			
				Carbonate, Sediment	negative			
				Epithelial Cells, Sediment	6	per field		
				Erythrocytes, Sediment	0	per field		
				Casts granul., Sediment	0	per field		
				Casts hyaline, Sediment	0	per field		
				Leucocytes, Sediment	0	per field		
				Oxalate, Sediment	negative			
				Specific Gravity	<=1.005			
				Mucus, Sediment	positive		H	No
				Triple Phosphate, Sediment	negative			
				Urates, Sediment	negative			
				Urobilinogen, urine (Stix)	0.2	mg/dl		
		BL	Clinical Chemistry	ALT, 37°C	9.5	U/L		
				Alkaline Phosphatase, 37°C	65.3	U/L		
				AST, 37°C	15.9	U/L		
				Bicarbonate	26.9	mmol/L		
				Bilirubin, total	18.0	umol/L		
				Urea/BUN	2.87	mmol/L		
				Calcium	2.41	mmol/L		
				Creatinine	53.8	umol/L		

Sign.: Significant finding; L: Result considered low; H: Result considered high; SC: Screening; BL: Baseline; D: Day; FUP: Follow-up;

Output generated by program 'NIC002_L16_2_8_Laboratory_V02_0_0'

Listing 16.2.8: Study subject data
Laboratory values

Part C

Cohort	Subject ID	Visit	Type	Measurement	Result		Flagged	Sign.
		BL	Clinical Chemistry	Glucose, serum	4.89	mmol/L		
				Gamma-GT, 37°C	10.0	U/L		
				Potassium	3.98	mmol/L		
				Magnesium	0.80	mmol/L		
				Sodium	134.6	mmol/L	L	No
			Drugs	Amphetamines, Urine	negative			
				Barbiturates, Urine	negative			
				Benzodiazepines, Urine	negative			
				Cannabin., Urine	negative			
				Cocaine, Urine	negative			
				Methadone, Urine	negative			
				Opiates, Urine	negative			
			Haematology	Basophils, abs.	0.04	10^9/L		
				Basophils, %	0.7	%		
				Eosinophils, abs.	0.05	10^9/L		
				Eosinophils, %	0.9	%		
				Haemoglobin	128.0	g/L		
				Haematocrit	0.37	L/L		
				Lymphocytes, abs.	1.92	10^9/L		
				Lymphocytes, %	34.2	%		
				Monocytes, abs.	0.51	10^9/L		
				Monocytes, %	9.1	%		
				Neutrophils, abs.	3.09	10^9/L		
				Neutrophils, %	55.1	%		
				Platelets	256	10^9/L		
				Erythrocytes	3.96	10^{12}/L	L	No
				Leucocytes	5.61	10^9/L		
			Haemostasis	APTT	28.0	s		
				Prothrombin Time (INR)	1.06	N/A		
				Prothrombin Time (PT)	90.8	%		
			Urine	Beta-HCG, urine	negative			

Sign.: Significant finding; L: Result considered low; H: Result considered high; SC: Screening; BL: Baseline; D: Day; FUP: Follow-up;

Output generated by program 'NIC002_L16_2_8_Laboratory_V02_0_0'

Listing 16.2.8: Study subject data
Laboratory values

Part C

Cohort	Subject ID	Visit	Type	Measurement	Result		Flagged	Sign.
		D03	Clinical Chemistry	ALT, 37°C	7.3	U/L		
				Alkaline Phosphatase, 37°C	61.3	U/L		
				AST, 37°C	14.1	U/L		
				Bicarbonate	24.0	mmol/L		
				Bilirubin, total	15.0	umol/L		
				Urea/BUN	4.22	mmol/L		
				Calcium	2.34	mmol/L		
				Creatinine	56.0	umol/L		
				Glucose, serum	4.18	mmol/L		
				Gamma-GT, 37°C	9.6	U/L		
				Potassium	4.07	mmol/L		
				Magnesium	0.76	mmol/L	L	No
				Sodium	136.2	mmol/L		
			Haematology	Basophils, abs.	0.05	10^9/L		
				Basophils, %	1.2	%		
				Eosinophils, abs.	0.05	10^9/L		
				Eosinophils, %	1.2	%		
				Haemoglobin	126.0	g/L		
				Haematocrit	0.36	L/L		
				Lymphocytes, abs.	1.36	10^9/L		
				Lymphocytes, %	33.3	%		
				Monocytes, abs.	0.38	10^9/L		
				Monocytes, %	9.3	%		
				Neutrophils, abs.	2.24	10^9/L		
				Neutrophils, %	55.0	%		
				Platelets	230	10^9/L		
				Erythrocytes	3.86	10^{12}/L	L	No
				Leucocytes	4.08	10^9/L		
			Haemostasis	APTT	29.0	s	H	No
				Prothrombin Time (INR)	1.13	N/A		

Sign.: Significant finding; L: Result considered low; H: Result considered high; SC: Screening; BL: Baseline; D: Day; FUP: Follow-up;

Output generated by program 'NIC002_L16_2_8_Laboratory_V02_0_0'

Listing 16.2.8: Study subject data
Laboratory values

Part C

Cohort	Subject ID	Visit	Type	Measurement	Result		Flagged	Sign.
		D03	Haemostasis	Prothrombin Time (PT)	77.6	%		
		D08	Clinical Chemistry	ALT, 37°C	6.4	U/L		
				Alkaline Phosphatase, 37°C	50.3	U/L		
				AST, 37°C	17.5	U/L		
				Bicarbonate	23.7	mmol/L		
				Bilirubin, total	6.9	umol/L		
				Urea/BUN	3.53	mmol/L		
				Calcium	2.27	mmol/L		
				Creatinine	59.4	umol/L		
				Glucose, serum	4.81	mmol/L		
				Gamma-GT, 37°C	7.7	U/L		
				Potassium	3.84	mmol/L		
				Magnesium	0.73	mmol/L	L	No
				Sodium	135.9	mmol/L	L	No
			Haematology	Basophils, abs.	0.03	10^9/L		
				Basophils, %	1.0	%		
				Eosinophils, abs.	0.03	10^9/L	L	No
				Eosinophils, %	1.0	%		
				Haemoglobin	120.0	g/L		
				Haematocrit	0.35	L/L		
				Lymphocytes, abs.	1.04	10^9/L		
				Lymphocytes, %	33.9	%		
				Monocytes, abs.	0.23	10^9/L	L	No
				Monocytes, %	7.5	%		
				Neutrophils, abs.	1.74	10^9/L		
				Neutrophils, %	56.6	%		
				Platelets	197	10^9/L		
				Erythrocytes	3.71	10^{12}/L	L	No
				Leucocytes	3.07	10^9/L	L	No
			Haemostasis	APTT	30.3	s	H	No

Sign.: Significant finding; L: Result considered low; H: Result considered high; SC: Screening; BL: Baseline; D: Day; FUP: Follow-up;

Output generated by program 'NIC002_L16_2_8_Laboratory_V02_0_0'

Listing 16.2.8: Study subject data
Laboratory values

Part C

Cohort	Subject ID	Visit	Type	Measurement	Result		Flagged	Sign.
		D08	Haemostasis	Prothrombin Time (INR)	1.09	N/A		
				Prothrombin Time (PT)	83.5	%		
		FUP	Clinical Chemistry	ALT, 37°C	11.4	U/L		
				Alkaline Phosphatase, 37°C	59.7	U/L		
				AST, 37°C	18.7	U/L		
				Bicarbonate	26.7	mmol/L		
				Bilirubin, total	7.0	umol/L		
				Urea/BUN	2.36	mmol/L	L	No
				Calcium	2.33	mmol/L		
				Creatinine	54.6	umol/L		
				Glucose, serum	5.44	mmol/L		
				Gamma-GT, 37°C	8.4	U/L		
				Potassium	4.82	mmol/L		
				Magnesium	0.82	mmol/L		
				Sodium	138.1	mmol/L		
			Haematology	Basophils, abs.	0.04	10^9/L		
				Basophils, %	0.8	%		
				Eosinophils, abs.	0.06	10^9/L		
				Eosinophils, %	1.3	%		
				Haemoglobin	125.0	g/L		
				Haematocrit	0.37	L/L		
				Lymphocytes, abs.	1.24	10^9/L		
				Lymphocytes, %	25.9	%		
				Monocytes, abs.	0.39	10^9/L		
				Monocytes, %	8.2	%		
				Neutrophils, abs.	3.05	10^9/L		
				Neutrophils, %	63.8	%		
				Platelets	210	10^9/L		
				Erythrocytes	3.87	10^{12}/L	L	No
				Leucocytes	4.78	10^9/L		

Sign.: Significant finding; L: Result considered low; H: Result considered high; SC: Screening; BL: Baseline; D: Day; FUP: Follow-up;

Output generated by program 'NIC002_L16_2_8_Laboratory_V02_0_0'

Listing 16.2.8: Study subject data
Laboratory values

Part C

Cohort	Subject ID	Visit	Type	Measurement	Result		Flagged	Sign.
		FUP	Haemostasis	APTT	26.5	s		
				Prothrombin Time (INR)	0.98	N/A		
				Prothrombin Time (PT)	108.0	%		
	71	SC	Clinical Chemistry	ALT, 37°C	11.1	U/L		
				Alkaline Phosphatase, 37°C	57.8	U/L		
				AST, 37°C	16.8	U/L		
				Bicarbonate	24.4	mmol/L		
				Bilirubin, total	14.2	umol/L		
				Urea/BUN	3.49	mmol/L		
				Calcium	2.34	mmol/L		
				Creatinine	60.9	umol/L		
				Glucose, serum	4.42	mmol/L		
				Gamma-GT, 37°C	10.7	U/L		
				Potassium	4.02	mmol/L		
				Creatinine Clearance MDRD	106	ml/min/1.73m		
				Magnesium	0.76	mmol/L	L	No
				Sodium	134.6	mmol/L	L	No
			Drugs	Amphetamines, Urine	negative			
				Barbiturates, Urine	negative			
				Benzodiazepines, Urine	negative			
				Cannabin., Urine	negative			
				Cocaine, Urine	negative			
				Methadone, Urine	negative			
				Opiates, Urine	negative			
			Haematology	Basophils, abs.	0.02	10^9/L		
				Basophils, %	0.3	%		
				Eosinophils, abs.	0.11	10^9/L		
				Eosinophils, %	1.6	%		
				Haemoglobin	135.0	g/L		
				Haematocrit	0.40	L/L		

Sign.: Significant finding; L: Result considered low; H: Result considered high; SC: Screening; BL: Baseline; D: Day; FUP: Follow-up;

Output generated by program 'NIC002_L16_2_8_Laboratory_V02_0_0'

Listing 16.2.8: Study subject data
Laboratory values

Part C

Cohort	Subject ID	Visit	Type	Measurement	Result		Flagged	Sign.
	71	SC	Haematology	Lymphocytes, abs.	2.00	10^9/L		
				Lymphocytes, %	29.5	%		
				Monocytes, abs.	0.44	10^9/L		
				Monocytes, %	6.5	%		
				Neutrophils, abs.	4.21	10^9/L		
				Neutrophils, %	62.1	%		
				Platelets	320	10^9/L		
				Erythrocytes	4.53	10^{12}/L		
				Leucocytes	6.78	10^9/L		
			Haemostasis	APTT	24.4	s		
				Prothrombin Time (INR)	0.99	N/A		
				Prothrombin Time (PT)	104.2	%		
			Infectious Diseases	HBs-Ag (Hep. B Surf. Ag)	negative	N/A		
				Anti-HCV (Hep. C-AB)	non-reactive	N/A		
				HIV 1+2, AG/AB	negative	N/A		
			Urine	Bilirubin, urine (Stix)	negative			
				Blood (Ery/Hb), urine (Stix)	negative			
				Glucose, urine (Stix)	negative			
				Beta-HCG, urine	negative			
				Ketone, urine (Stix)	negative			
				Leucocytes, urine (Stix)	1+		H	No
				Nitrite, urine (Stix)	negative			
				pH, urine (Stix)	6.5	neg.log[H+]		
				Protein, total, urine (Stix)	negative			
				Bacteria, Sediment	positive		H	No
				Carbonate, Sediment	negative			
				Epithelial Cells, Sediment	9	per field		
				Erythrocytes, Sediment	3	per field	H	No
				Casts granul., Sediment	0	per field		
				Casts hyaline, Sediment	0	per field		
				Leucocytes, Sediment	5	per field	H	No

Sign.: Significant finding; L: Result considered low; H: Result considered high; SC: Screening; BL: Baseline; D: Day; FUP: Follow-up;

Output generated by program 'NIC002_L16_2_8_Laboratory_V02_0_0'

Listing 16.2.8: Study subject data
Laboratory values

Part C

Cohort	Subject ID	Visit	Type	Measurement	Result		Flagged	Sign.
	71	SC	Urine	Oxalate, Sediment	negative			
				Specific Gravity	<=1.005			
				Mucus, Sediment	negative			
				Triple Phosphate, Sediment	negative			
				Urates, Sediment	negative			
				Urobilinogen, urine (Stix)	0.2	mg/dl		
		BL	Clinical Chemistry	ALT, 37°C	15.2	U/L		
				Alkaline Phosphatase, 37°C	71.1	U/L		
				AST, 37°C	21.6	U/L		
				Bicarbonate	26.2	mmol/L		
				Bilirubin, total	12.0	umol/L		
				Urea/BUN	4.02	mmol/L		
				Calcium	2.38	mmol/L		
				Creatinine	63.5	umol/L		
				Glucose, serum	4.43	mmol/L		
				Gamma-GT, 37°C	10.9	U/L		
				Potassium	4.50	mmol/L		
				Magnesium	0.80	mmol/L		
				Sodium	136.9	mmol/L		
			Drugs	Amphetamines, Urine	negative			
				Barbiturates, Urine	negative			
				Benzodiazepines, Urine	negative			
				Cannabin., Urine	negative			
				Cocaine, Urine	negative			
				Methadone, Urine	negative			
				Opiates, Urine	negative			
			Haematology	Basophils, abs.	0.01	10^9/L		
				Basophils, %	0.2	%		
				Eosinophils, abs.	0.15	10^9/L		
				Eosinophils, %	2.7	%		

Sign.: Significant finding; L: Result considered low; H: Result considered high; SC: Screening; BL: Baseline; D: Day; FUP: Follow-up;

Output generated by program 'NIC002_L16_2_8_Laboratory_V02_0_0'

Listing 16.2.8: Study subject data
Laboratory values

Part C

Cohort	Subject ID	Visit	Type	Measurement	Result		Flagged	Sign.
		BL	Haematology	Haemoglobin	135.0	g/L		
				Haematocrit	0.40	L/L		
				Lymphocytes, abs.	1.92	10^9/L		
				Lymphocytes, %	34.6	%		
				Monocytes, abs.	0.43	10^9/L		
				Monocytes, %	7.7	%		
				Neutrophils, abs.	3.04	10^9/L		
				Neutrophils, %	54.8	%		
				Platelets	302	10^9/L		
				Erythrocytes	4.48	10^{12}/L		
				Leucocytes	5.55	10^9/L		
			Haemostasis	APTT	25.9	s		
				Prothrombin Time (INR)	0.99	N/A		
				Prothrombin Time (PT)	107.8	%		
			Urine	Beta-HCG, urine	negative			
		D03	Clinical Chemistry	ALT, 37°C	12.8	U/L		
				Alkaline Phosphatase, 37°C	65.4	U/L		
				AST, 37°C	16.2	U/L		
				Bicarbonate	24.1	mmol/L		
				Bilirubin, total	14.2	umol/L		
				Urea/BUN	4.61	mmol/L		
				Calcium	2.27	mmol/L		
				Creatinine	61.5	umol/L		
				Glucose, serum	3.95	mmol/L	L	No
				Gamma-GT, 37°C	10.1	U/L		
				Potassium	4.21	mmol/L		
				Magnesium	0.74	mmol/L	L	No
				Sodium	137.4	mmol/L		
			Haematology	Basophils, abs.	0.01	10^9/L		
				Basophils, %	0.2	%		

Sign.: Significant finding; L: Result considered low; H: Result considered high; SC: Screening; BL: Baseline; D: Day; FUP: Follow-up;

Output generated by program 'NIC002_L16_2_8_Laboratory_V02_0_0'

Listing 16.2.8: Study subject data
Laboratory values

Part C

Cohort	Subject ID	Visit	Type	Measurement	Result		Flagged	Sign.
		D03	Haematology	Eosinophils, abs.	0.12	10^9/L		
				Eosinophils, %	2.8	%		
				Haemoglobin	132.0	g/L		
				Haematocrit	0.40	L/L		
				Lymphocytes, abs.	1.93	10^9/L		
				Lymphocytes, %	45.3	%		
				Monocytes, abs.	0.27	10^9/L		
				Monocytes, %	6.3	%		
				Neutrophils, abs.	1.93	10^9/L		
				Neutrophils, %	45.4	%		
				Platelets	287	10^9/L		
				Erythrocytes	4.37	10^{12}/L		
				Leucocytes	4.26	10^9/L		
			Haemostasis	APTT	27.1	s		
				Prothrombin Time (INR)	1.01	N/A		
				Prothrombin Time (PT)	99.7	%		
		D08	Clinical Chemistry	ALT, 37°C	9.6	U/L		
				Alkaline Phosphatase, 37°C	53.5	U/L		
				AST, 37°C	16.4	U/L		
				Bicarbonate	22.7	mmol/L		
				Bilirubin, total	9.6	umol/L		
				Urea/BUN	3.82	mmol/L		
				Calcium	2.32	mmol/L		
				Creatinine	61.1	umol/L		
				Glucose, serum	4.27	mmol/L		
				Gamma-GT, 37°C	8.5	U/L		
				Potassium	4.32	mmol/L		
				Magnesium	0.74	mmol/L	L	No
				Sodium	138.0	mmol/L		
			Haematology	Basophils, abs.	0.02	10^9/L		

Sign.: Significant finding; L: Result considered low; H: Result considered high; SC: Screening; BL: Baseline; D: Day; FUP: Follow-up;

Output generated by program 'NIC002_L16_2_8_Laboratory_V02_0_0'

Listing 16.2.8: Study subject data
Laboratory values

Part C

Cohort	Subject ID	Visit	Type	Measurement	Result		Flagged	Sign.
		D08	Haematology	Basophils, %	0.4	%		
				Eosinophils, abs.	0.13	10^9/L		
				Eosinophils, %	2.3	%		
				Haemoglobin	123.0	g/L		
				Haematocrit	0.37	L/L		
				Lymphocytes, abs.	1.68	10^9/L		
				Lymphocytes, %	30.1	%		
				Monocytes, abs.	0.30	10^9/L		
				Monocytes, %	5.4	%		
				Neutrophils, abs.	3.46	10^9/L		
				Neutrophils, %	61.8	%		
				Platelets	242	10^9/L		
				Erythrocytes	4.14	10^{12}/L		
				Leucocytes	5.59	10^9/L		
			Haemostasis	APTT	26.1	s		
				Prothrombin Time (INR)	0.96	N/A		
				Prothrombin Time (PT)	114.1	%		
		FUP	Clinical Chemistry	ALT, 37°C	17.1	U/L		
				Alkaline Phosphatase, 37°C	60.5	U/L		
				AST, 37°C	20.6	U/L		
				Bicarbonate	21.0	mmol/L		
				Bilirubin, total	8.0	umol/L		
				Urea/BUN	3.05	mmol/L		
				Calcium	2.24	mmol/L		
				Creatinine	54.6	umol/L		
				Glucose, serum	4.07	mmol/L	L	No
				Gamma-GT, 37°C	9.7	U/L		
				Potassium	3.92	mmol/L		
				Magnesium	0.72	mmol/L	L	No
				Sodium	136.5	mmol/L		

Sign.: Significant finding; L: Result considered low; H: Result considered high; SC: Screening; BL: Baseline; D: Day; FUP: Follow-up;

Output generated by program 'NIC002_L16_2_8_Laboratory_V02_0_0'

Listing 16.2.8: Study subject data
Laboratory values

Part C

Cohort	Subject ID	Visit	Type	Measurement	Result		Flagged	Sign.
		FUP	Haematology	Basophils, abs.	0.03	10^9/L		
				Basophils, %	0.6	%		
				Eosinophils, abs.	0.19	10^9/L		
				Eosinophils, %	3.9	%		
				Haemoglobin	127.0	g/L		
				Haematocrit	0.37	L/L		
				Lymphocytes, abs.	1.68	10^9/L		
				Lymphocytes, %	34.4	%		
				Monocytes, abs.	0.33	10^9/L		
				Monocytes, %	6.7	%		
				Neutrophils, abs.	2.66	10^9/L		
				Neutrophils, %	54.4	%		
				Platelets	253	10^9/L		
				Erythrocytes	4.22	10^{12}/L		
				Leucocytes	4.89	10^9/L		
			Haemostasis	APTT	23.3	s		
				Prothrombin Time (INR)	0.89	N/A		
				Prothrombin Time (PT)	> 130.0	%	H	No
Group 2	57	SC	Clinical Chemistry	ALT, 37°C	19.2	U/L		
				Alkaline Phosphatase, 37°C	57.6	U/L		
				AST, 37°C	19.1	U/L		
				Bicarbonate	26.2	mmol/L		
				Bilirubin, total	13.3	umol/L		
				Urea/BUN	5.44	mmol/L		
				Calcium	2.31	mmol/L		
				Creatinine	49.5	umol/L		
				Glucose, serum	5.43	mmol/L		
				Gamma-GT, 37°C	16.2	U/L		
				Potassium	4.76	mmol/L		
				Creatinine Clearance MDRD	126	ml/min/1.73m		

Sign.: Significant finding; L: Result considered low; H: Result considered high; SC: Screening; BL: Baseline; D: Day; FUP: Follow-up;

Output generated by program 'NIC002_L16_2_8_Laboratory_V02_0_0'

Listing 16.2.8: Study subject data
Laboratory values

Part C

Cohort	Subject ID	Visit	Type	Measurement	Result		Flagged	Sign.
Group 2	57	SC	Clinical Chemistry	Magnesium	0.77	mmol/L		
				Sodium	137.8	mmol/L		
			Drugs	Amphetamines, Urine	negative			
				Barbiturates, Urine	negative			
				Benzodiazepines, Urine	negative			
				Cannabin., Urine	negative			
				Cocaine, Urine	negative			
				Methadone, Urine	negative			
				Opiates, Urine	negative			
			Haematology	Basophils, abs.	0.03	10^9/L		
				Basophils, %	0.5	%		
				Eosinophils, abs.	0.11	10^9/L		
				Eosinophils, %	1.8	%		
				Haemoglobin	135.0	g/L		
				Haematocrit	0.38	L/L		
				Lymphocytes, abs.	1.83	10^9/L		
				Lymphocytes, %	30.6	%		
				Monocytes, abs.	0.46	10^9/L		
				Monocytes, %	7.7	%		
				Neutrophils, abs.	3.56	10^9/L		
				Neutrophils, %	59.4	%		
				Platelets	301	10^9/L		
				Erythrocytes	4.39	10^{12}/L		
				Leucocytes	5.99	10^9/L		
			Haemostasis	APTT	26.3	s		
				Prothrombin Time (INR)	1.07	N/A		
				Prothrombin Time (PT)	87.0	%		
			Infectious Diseases	HBs-Ag (Hep. B Surf. Ag)	negative	N/A		
				Anti-HCV (Hep. C-AB)	non-reactive	N/A		
				HIV 1+2, AG/AB	negative	N/A		
			Urine	Bilirubin, urine (Stix)	negative			

Sign.: Significant finding; L: Result considered low; H: Result considered high; SC: Screening; BL: Baseline; D: Day; FUP: Follow-up;

Output generated by program 'NIC002_L16_2_8_Laboratory_V02_0_0'

Listing 16.2.8: Study subject data
Laboratory values

Part C

Cohort	Subject ID	Visit	Type	Measurement	Result		Flagged	Sign.
Group 2	57	SC	Urine	Blood (Ery/Hb), urine (Stix)	negative			
				Glucose, urine (Stix)	negative			
				Beta-HCG, urine	negative			
				Ketone, urine (Stix)	negative			
				Leucocytes, urine (Stix)	negative			
				Nitrite, urine (Stix)	negative			
				pH, urine (Stix)	6.5	neg.log[H+]		
				Protein, total, urine (Stix)	negative			
				Specific Gravity	1.020			
				Urobilinogen, urine (Stix)	0.2	mg/dl		
		BL	Clinical Chemistry	ALT, 37°C	18.5	U/L		
				Alkaline Phosphatase, 37°C	58.8	U/L		
				AST, 37°C	18.8	U/L		
				Bicarbonate	25.9	mmol/L		
				Bilirubin, total	16.4	umol/L		
				Urea/BUN	5.79	mmol/L		
				Calcium	2.21	mmol/L		
				Creatinine	50.2	umol/L		
				Glucose, serum	5.86	mmol/L		
				Gamma-GT, 37°C	16.2	U/L		
				Potassium	4.45	mmol/L		
				Magnesium	0.79	mmol/L		
				Sodium	137.9	mmol/L		
			Drugs	Amphetamines, Urine	negative			
				Barbiturates, Urine	negative			
				Benzodiazepines, Urine	negative			
				Cannabin., Urine	negative			
				Cocaine, Urine	negative			
				Methadone, Urine	negative			
				Opiates, Urine	negative			

Sign.: Significant finding; L: Result considered low; H: Result considered high; SC: Screening; BL: Baseline; D: Day; FUP: Follow-up;

Output generated by program 'NIC002_L16_2_8_Laboratory_V02_0_0'

Listing 16.2.8: Study subject data
Laboratory values

Part C

Cohort	Subject ID	Visit	Type	Measurement	Result		Flagged	Sign.
		BL	Haematology	Basophils, abs.	0.05	10^9/L		
				Basophils, %	0.7	%		
				Eosinophils, abs.	0.27	10^9/L		
				Eosinophils, %	3.7	%		
				Haemoglobin	135.0	g/L		
				Haematocrit	0.39	L/L		
				Lymphocytes, abs.	1.76	10^9/L		
				Lymphocytes, %	23.9	%		
				Monocytes, abs.	0.54	10^9/L		
				Monocytes, %	7.3	%		
				Neutrophils, abs.	4.74	10^9/L		
				Neutrophils, %	64.4	%		
				Platelets	285	10^9/L		
				Erythrocytes	4.41	10^{12}/L		
				Leucocytes	7.36	10^9/L		
			Haemostasis	APTT	27.0	s		
				Prothrombin Time (INR)	1.06	N/A		
				Prothrombin Time (PT)	89.2	%		
			Urine	Beta-HCG, urine	negative			
		D03	Clinical Chemistry	ALT, 37°C	16.5	U/L		
				Alkaline Phosphatase, 37°C	55.4	U/L		
				AST, 37°C	16.3	U/L		
				Bicarbonate	25.3	mmol/L		
				Bilirubin, total	25.1	umol/L	H	No
				Urea/BUN	5.20	mmol/L		
				Calcium	2.30	mmol/L		
				Creatinine	53.1	umol/L		
				Glucose, serum	4.93	mmol/L		
				Gamma-GT, 37°C	16.1	U/L		
				Potassium	4.43	mmol/L		

Sign.: Significant finding; L: Result considered low; H: Result considered high; SC: Screening; BL: Baseline; D: Day; FUP: Follow-up;

Output generated by program 'NIC002_L16_2_8_Laboratory_V02_0_0'

Listing 16.2.8: Study subject data
Laboratory values

Part C

Cohort	Subject ID	Visit	Type	Measurement	Result		Flagged	Sign.
		D03	Clinical Chemistry	Magnesium	0.83	mmol/L		
				Sodium	138.2	mmol/L		
			Haematology	Basophils, abs.	0.04	10^9/L		
				Basophils, %	0.7	%		
				Eosinophils, abs.	0.19	10^9/L		
				Eosinophils, %	3.3	%		
				Haemoglobin	140.0	g/L		
				Haematocrit	0.41	L/L		
				Lymphocytes, abs.	1.97	10^9/L		
				Lymphocytes, %	34.5	%		
				Monocytes, abs.	0.40	10^9/L		
				Monocytes, %	7.0	%		
				Neutrophils, abs.	3.11	10^9/L		
				Neutrophils, %	54.5	%		
				Platelets	292	10^9/L		
				Erythrocytes	4.48	10^{12}/L		
				Leucocytes	5.71	10^9/L		
			Haemostasis	APTT	28.2	s	H	No
				Prothrombin Time (INR)	1.10	N/A		
				Prothrombin Time (PT)	80.8	%		
		D08	Clinical Chemistry	ALT, 37°C	31.7	U/L		
				Alkaline Phosphatase, 37°C	55.1	U/L		
				AST, 37°C	24.3	U/L		
				Bicarbonate	24.6	mmol/L		
				Bilirubin, total	17.3	umol/L		
				Urea/BUN	4.63	mmol/L		
				Calcium	2.31	mmol/L		
				Creatinine	54.0	umol/L		
				Glucose, serum	4.90	mmol/L		
				Gamma-GT, 37°C	13.9	U/L		

Sign.: Significant finding; L: Result considered low; H: Result considered high; SC: Screening; BL: Baseline; D: Day; FUP: Follow-up;

Output generated by program 'NIC002_L16_2_8_Laboratory_V02_0_0'

Listing 16.2.8: Study subject data
Laboratory values

Part C

Cohort	Subject ID	Visit	Type	Measurement	Result		Flagged	Sign.
		D08	Clinical Chemistry	Potassium	4.41	mmol/L		
				Magnesium	0.79	mmol/L		
				Sodium	137.9	mmol/L		
			Haematology	Basophils, abs.	0.03	10^9/L		
				Basophils, %	0.6	%		
				Eosinophils, abs.	0.14	10^9/L		
				Eosinophils, %	2.7	%		
				Haemoglobin	133.0	g/L		
				Haematocrit	0.38	L/L		
				Lymphocytes, abs.	1.72	10^9/L		
				Lymphocytes, %	32.7	%		
				Monocytes, abs.	0.36	10^9/L		
				Monocytes, %	6.8	%		
				Neutrophils, abs.	3.01	10^9/L		
				Neutrophils, %	57.2	%		
				Platelets	307	10^9/L		
				Erythrocytes	4.25	10^{12}/L		
				Leucocytes	5.26	10^9/L		
			Haemostasis	APTT	28.9	s	H	No
				Prothrombin Time (INR)	1.10	N/A		
				Prothrombin Time (PT)	80.8	%		
		FUP	Clinical Chemistry	ALT, 37°C	31.2	U/L		
				Alkaline Phosphatase, 37°C	56.5	U/L		
				AST, 37°C	26.7	U/L		
				Bicarbonate	26.7	mmol/L		
				Bilirubin, total	13.5	umol/L		
				Urea/BUN	4.64	mmol/L		
				Calcium	2.31	mmol/L		
				Creatinine	52.0	umol/L		
				Glucose, serum	4.83	mmol/L		

Sign.: Significant finding; L: Result considered low; H: Result considered high; SC: Screening; BL: Baseline; D: Day; FUP: Follow-up;

Output generated by program 'NIC002_L16_2_8_Laboratory_V02_0_0'

Listing 16.2.8: Study subject data
Laboratory values

Part C

Cohort	Subject ID	Visit	Type	Measurement	Result		Flagged	Sign.
		FUP	Clinical Chemistry	Gamma-GT, 37°C	14.3	U/L		
				Potassium	4.19	mmol/L		
				Magnesium	0.78	mmol/L		
				Sodium	136.9	mmol/L		
			Haematology	Basophils, abs.	0.06	10^9/L		
				Basophils, %	1.0	%		
				Eosinophils, abs.	0.19	10^9/L		
				Eosinophils, %	3.3	%		
				Haemoglobin	134.0	g/L		
				Haematocrit	0.39	L/L		
				Lymphocytes, abs.	2.10	10^9/L		
				Lymphocytes, %	36.3	%		
				Monocytes, abs.	0.43	10^9/L		
				Monocytes, %	7.4	%		
				Neutrophils, abs.	3.01	10^9/L		
				Neutrophils, %	52.0	%		
				Platelets	333	10^9/L		
				Erythrocytes	4.35	10^{12}/L		
				Leucocytes	5.79	10^9/L		
			Haemostasis	APTT	28.3	s	H	No
				Prothrombin Time (INR)	1.08	N/A		
				Prothrombin Time (PT)	86.0	%		
	58	SC	Clinical Chemistry	ALT, 37°C	11.0	U/L		
				Alkaline Phosphatase, 37°C	40.4	U/L		
				AST, 37°C	14.6	U/L		
				Bicarbonate	25.4	mmol/L		
				Bilirubin, total	11.2	umol/L		
				Urea/BUN	5.57	mmol/L		
				Calcium	2.26	mmol/L		
				Creatinine	52.7	umol/L		

Sign.: Significant finding; L: Result considered low; H: Result considered high; SC: Screening; BL: Baseline; D: Day; FUP: Follow-up;

Output generated by program 'NIC002_L16_2_8_Laboratory_V02_0_0'

Listing 16.2.8: Study subject data
Laboratory values

Part C

Cohort	Subject ID	Visit	Type	Measurement	Result		Flagged	Sign.
	58	SC	Clinical Chemistry	Glucose, serum	5.12	mmol/L		
				Gamma-GT, 37°C	9.4	U/L		
				Potassium	4.31	mmol/L		
				Creatinine Clearance MDRD	110	ml/min/1.73m		
				Magnesium	0.78	mmol/L		
				Sodium	136.9	mmol/L		
			Drugs	Amphetamines, Urine	negative			
				Barbiturates, Urine	negative			
				Benzodiazepines, Urine	negative			
				Cannabin., Urine	negative			
				Cocaine, Urine	negative			
				Methadone, Urine	negative			
				Opiates, Urine	negative			
			Haematology	Basophils, abs.	0.03	10^9/L		
				Basophils, %	0.5	%		
				Eosinophils, abs.	0.04	10^9/L		
				Eosinophils, %	0.6	%		
				Haemoglobin	126.0	g/L		
				Haematocrit	0.36	L/L		
				Lymphocytes, abs.	1.84	10^9/L		
				Lymphocytes, %	29.3	%		
				Monocytes, abs.	0.35	10^9/L		
				Monocytes, %	5.6	%		
				Neutrophils, abs.	4.03	10^9/L		
				Neutrophils, %	64.0	%		
				Platelets	268	10^9/L		
				Erythrocytes	3.99	10^{12}/L	L	No
				Leucocytes	6.29	10^9/L		
			Haemostasis	APTT	22.9	s		
				Prothrombin Time (INR)	1.04	N/A		
				Prothrombin Time (PT)	93.9	%		

Sign.: Significant finding; L: Result considered low; H: Result considered high; SC: Screening; BL: Baseline; D: Day; FUP: Follow-up;

Output generated by program 'NIC002_L16_2_8_Laboratory_V02_0_0'

Listing 16.2.8: Study subject data
Laboratory values

Part C

Cohort	Subject ID	Visit	Type	Measurement	Result		Flagged	Sign.
	58	SC	Infectious Diseases	HBs-Ag (Hep. B Surf. Ag)	negative	N/A		
				Anti-HCV (Hep. C-AB)	non-reactive	N/A		
				HIV 1+2, AG/AB	negative	N/A		
			Urine	Bilirubin, urine (Stix)	negative			
				Blood (Ery/Hb), urine (Stix)	negative			
				Glucose, urine (Stix)	negative			
				Beta-HCG, urine	negative			
				Ketone, urine (Stix)	negative			
				Leucocytes, urine (Stix)	negative			
				Nitrite, urine (Stix)	negative			
				pH, urine (Stix)	5.5	neg.log[H+]		
				Protein, total, urine (Stix)	negative			
				Specific Gravity	1.025			
				Urobilinogen, urine (Stix)	0.2	mg/dl		
		BL	Clinical Chemistry	ALT, 37°C	11.0	U/L		
				Alkaline Phosphatase, 37°C	41.1	U/L		
				AST, 37°C	13.8	U/L		
				Bicarbonate	24.7	mmol/L		
				Bilirubin, total	12.7	umol/L		
				Urea/BUN	5.75	mmol/L		
				Calcium	2.24	mmol/L		
				Creatinine	54.4	umol/L		
				Glucose, serum	5.11	mmol/L		
				Gamma-GT, 37°C	10.1	U/L		
				Potassium	4.15	mmol/L		
				Magnesium	0.79	mmol/L		
				Sodium	137.4	mmol/L		
			Drugs	Amphetamines, Urine	negative			
				Barbiturates, Urine	negative			
				Benzodiazepines, Urine	negative			

Sign.: Significant finding; L: Result considered low; H: Result considered high; SC: Screening; BL: Baseline; D: Day; FUP: Follow-up;

Output generated by program 'NIC002_L16_2_8_Laboratory_V02_0_0'

Listing 16.2.8: Study subject data
Laboratory values

Part C

Cohort	Subject ID	Visit	Type	Measurement	Result		Flagged	Sign.
		BL	Drugs	Cannabin., Urine	negative			
				Cocaine, Urine	negative			
				Methadone, Urine	negative			
				Opiates, Urine	negative			
			Haematology	Basophils, abs.	0.03	10^9/L		
				Basophils, %	0.4	%		
				Eosinophils, abs.	0.06	10^9/L		
				Eosinophils, %	0.7	%		
				Haemoglobin	131.0	g/L		
				Haematocrit	0.38	L/L		
				Lymphocytes, abs.	1.93	10^9/L		
				Lymphocytes, %	23.7	%		
				Monocytes, abs.	0.41	10^9/L		
				Monocytes, %	5.0	%	L	No
				Neutrophils, abs.	5.70	10^9/L		
				Neutrophils, %	70.2	%		
				Platelets	246	10^9/L		
				Erythrocytes	4.24	10^{12}/L		
				Leucocytes	8.13	10^9/L		
			Haemostasis	APTT	23.0	s		
				Prothrombin Time (INR)	1.02	N/A		
				Prothrombin Time (PT)	96.3	%		
			Urine	Beta-HCG, urine	negative			
		D03	Clinical Chemistry	ALT, 37°C	9.1	U/L		
				Alkaline Phosphatase, 37°C	40.3	U/L		
				AST, 37°C	12.9	U/L		
				Bicarbonate	24.9	mmol/L		
				Bilirubin, total	14.4	umol/L		
				Urea/BUN	5.05	mmol/L		
				Calcium	2.28	mmol/L		

Sign.: Significant finding; L: Result considered low; H: Result considered high; SC: Screening; BL: Baseline; D: Day; FUP: Follow-up;

Output generated by program 'NIC002_L16_2_8_Laboratory_V02_0_0'

Listing 16.2.8: Study subject data
Laboratory values

Part C

Cohort	Subject ID	Visit	Type	Measurement	Result		Flagged	Sign.
		D03	Clinical Chemistry	Creatinine	53.8	umol/L		
				Glucose, serum	4.66	mmol/L		
				Gamma-GT, 37°C	10.0	U/L		
				Potassium	4.04	mmol/L		
				Magnesium	0.81	mmol/L		
				Sodium	137.7	mmol/L		
			Haematology	Basophils, abs.	0.02	10^9/L		
				Basophils, %	0.3	%		
				Eosinophils, abs.	0.06	10^9/L		
				Eosinophils, %	1.0	%		
				Haemoglobin	130.0	g/L		
				Haematocrit	0.38	L/L		
				Lymphocytes, abs.	2.16	10^9/L		
				Lymphocytes, %	34.6	%		
				Monocytes, abs.	0.34	10^9/L		
				Monocytes, %	5.4	%		
				Neutrophils, abs.	3.67	10^9/L		
				Neutrophils, %	58.7	%		
				Platelets	253	10^9/L		
				Erythrocytes	4.20	10^{12}/L		
				Leucocytes	6.25	10^9/L		
			Haemostasis	APTT	23.7	s		
				Prothrombin Time (INR)	1.08	N/A		
				Prothrombin Time (PT)	84.9	%		
		D08	Clinical Chemistry	ALT, 37°C	20.4	U/L		
				Alkaline Phosphatase, 37°C	41.2	U/L		
				AST, 37°C	17.8	U/L		
				Bicarbonate	23.5	mmol/L		
				Bilirubin, total	6.8	umol/L		
				Urea/BUN	4.99	mmol/L		

Sign.: Significant finding; L: Result considered low; H: Result considered high; SC: Screening; BL: Baseline; D: Day; FUP: Follow-up;

Output generated by program 'NIC002_L16_2_8_Laboratory_V02_0_0'

Listing 16.2.8: Study subject data
Laboratory values

Part C

Cohort	Subject ID	Visit	Type	Measurement	Result		Flagged	Sign.
		D08	Clinical Chemistry	Calcium	2.19	mmol/L	L	No
				Creatinine	55.2	umol/L		
				Glucose, serum	4.99	mmol/L		
				Gamma-GT, 37°C	9.3	U/L		
				Potassium	3.88	mmol/L		
				Magnesium	0.79	mmol/L		
				Sodium	138.8	mmol/L		
			Haematology	Basophils, abs.	0.03	10^9/L		
				Basophils, %	0.8	%		
				Eosinophils, abs.	0.05	10^9/L		
				Eosinophils, %	1.3	%		
				Haemoglobin	124.0	g/L		
				Haematocrit	0.36	L/L		
				Lymphocytes, abs.	1.14	10^9/L		
				Lymphocytes, %	28.9	%		
				Monocytes, abs.	0.54	10^9/L		
				Monocytes, %	13.7	%		
				Neutrophils, abs.	2.19	10^9/L		
				Neutrophils, %	55.3	%		
				Platelets	213	10^9/L		
				Erythrocytes	3.94	10^{12}/L	L	No
				Leucocytes	3.95	10^9/L		
			Haemostasis	APTT	24.4	s		
				Prothrombin Time (INR)	1.05	N/A		
				Prothrombin Time (PT)	91.5	%		
		FUP	Clinical Chemistry	ALT, 37°C	25.8	U/L		
				Alkaline Phosphatase, 37°C	43.0	U/L		
				AST, 37°C	20.2	U/L		
				Bicarbonate	25.7	mmol/L		
				Bilirubin, total	10.5	umol/L		

Sign.: Significant finding; L: Result considered low; H: Result considered high; SC: Screening; BL: Baseline; D: Day; FUP: Follow-up;

Output generated by program 'NIC002_L16_2_8_Laboratory_V02_0_0'

Listing 16.2.8: Study subject data
Laboratory values

Part C

Cohort	Subject ID	Visit	Type	Measurement	Result		Flagged	Sign.
		FUP	Clinical Chemistry	Urea/BUN	6.16	mmol/L		
				Calcium	2.24	mmol/L		
				Creatinine	54.9	umol/L		
				Glucose, serum	5.16	mmol/L		
				Gamma-GT, 37°C	10.2	U/L		
				Potassium	3.89	mmol/L		
				Magnesium	0.82	mmol/L		
				Sodium	137.9	mmol/L		
			Haematology	Basophils, abs.	0.02	10^9/L		
				Basophils, %	0.4	%		
				Eosinophils, abs.	0.08	10^9/L		
				Eosinophils, %	1.7	%		
				Haemoglobin	123.0	g/L		
				Haematocrit	0.36	L/L		
				Lymphocytes, abs.	2.04	10^9/L		
				Lymphocytes, %	42.3	%		
				Monocytes, abs.	0.33	10^9/L		
				Monocytes, %	6.8	%		
				Neutrophils, abs.	2.35	10^9/L		
				Neutrophils, %	48.8	%		
				Platelets	240	10^9/L		
				Erythrocytes	3.91	10^{12}/L	L	No
				Leucocytes	4.82	10^9/L		
			Haemostasis	APTT	22.8	s		
				Prothrombin Time (INR)	0.98	N/A		
				Prothrombin Time (PT)	111.0	%		
	68	SC	Clinical Chemistry	ALT, 37°C	11.6	U/L		
				Alkaline Phosphatase, 37°C	34.9	U/L		
				AST, 37°C	18.0	U/L		
				Bicarbonate	27.0	mmol/L		

Sign.: Significant finding; L: Result considered low; H: Result considered high; SC: Screening; BL: Baseline; D: Day; FUP: Follow-up;

Output generated by program 'NIC002_L16_2_8_Laboratory_V02_0_0'

Listing 16.2.8: Study subject data
Laboratory values

Part C

Cohort	Subject ID	Visit	Type	Measurement	Result		Flagged	Sign.
	68	SC	Clinical Chemistry	Bilirubin, total	10.5	umol/L		
				Urea/BUN	3.29	mmol/L		
				Calcium	2.34	mmol/L		
				Creatinine	61.5	umol/L		
				Glucose, serum	4.34	mmol/L		
				Gamma-GT, 37°C	15.1	U/L		
				Potassium	3.99	mmol/L		
				Creatinine Clearance MDRD	100	ml/min/1.73m		
				Magnesium	0.81	mmol/L		
				Sodium	137.1	mmol/L		
			Drugs	Amphetamines, Urine	negative			
				Barbiturates, Urine	negative			
				Benzodiazepines, Urine	negative			
				Cannabin., Urine	negative			
				Cocaine, Urine	negative			
				Methadone, Urine	negative			
				Opiates, Urine	negative			
			Haematology	Basophils, abs.	0.06	10^9/L		
				Basophils, %	0.9	%		
				Eosinophils, abs.	0.11	10^9/L		
				Eosinophils, %	1.7	%		
				Haemoglobin	139.0	g/L		
				Haematocrit	0.41	L/L		
				Lymphocytes, abs.	2.86	10^9/L		
				Lymphocytes, %	44.5	%		
				Monocytes, abs.	0.53	10^9/L		
				Monocytes, %	8.2	%		
				Neutrophils, abs.	2.87	10^9/L		
				Neutrophils, %	44.7	%		
				Platelets	284	10^9/L		
				Erythrocytes	4.46	10^{12}/L		

Sign.: Significant finding; L: Result considered low; H: Result considered high; SC: Screening; BL: Baseline; D: Day; FUP: Follow-up;

Output generated by program 'NIC002_L16_2_8_Laboratory_V02_0_0'

Listing 16.2.8: Study subject data
Laboratory values

Part C

Cohort	Subject ID	Visit	Type	Measurement	Result		Flagged	Sign.
	68	SC	Haematology	Leucocytes	6.43	10^9/L		
			Haemostasis	APTT	25.7	s		
				Prothrombin Time (INR)	1.01	N/A		
				Prothrombin Time (PT)	98.8	%		
			Infectious Diseases	HBs-Ag (Hep. B Surf. Ag)	negative	N/A		
				Anti-HCV (Hep. C-AB)	non-reactive	N/A		
				HIV 1+2, AG/AB	negative	N/A		
			Urine	Bilirubin, urine (Stix)	negative			
				Blood (Ery/Hb), urine (Stix)	3+		H	No
				Glucose, urine (Stix)	negative			
				Beta-HCG, urine	negative			
				Ketone, urine (Stix)	negative			
				Leucocytes, urine (Stix)	negative			
				Nitrite, urine (Stix)	negative			
				pH, urine (Stix)	6.0	neg.log[H+]		
				Protein, total, urine (Stix)	negative			
				Bacteria, Sediment	negative			
				Carbonate, Sediment	negative			
				Epithelial Cells, Sediment	6	per field		
				Erythrocytes, Sediment	2	per field	H	No
				Casts granul., Sediment	0	per field		
				Casts hyaline, Sediment	0	per field		
				Leucocytes, Sediment	2	per field		
				Oxalate, Sediment	negative			
				Specific Gravity	1.020			
				Mucus, Sediment	positive		H	No
				Triple Phosphate, Sediment	negative			
				Urates, Sediment	negative			
				Urobilinogen, urine (Stix)	0.2	mg/dl		
		BL	Clinical Chemistry	ALT, 37°C	10.5	U/L		

Sign.: Significant finding; L: Result considered low; H: Result considered high; SC: Screening; BL: Baseline; D: Day; FUP: Follow-up;

Output generated by program 'NIC002_L16_2_8_Laboratory_V02_0_0'

Listing 16.2.8: Study subject data
Laboratory values

Part C

Cohort	Subject ID	Visit	Type	Measurement	Result		Flagged	Sign.
		BL	Clinical Chemistry	Alkaline Phosphatase, 37°C	33.5	U/L		
				AST, 37°C	18.8	U/L		
				Bicarbonate	25.6	mmol/L		
				Bilirubin, total	6.8	umol/L		
				Urea/BUN	3.81	mmol/L		
				Calcium	2.31	mmol/L		
				Creatinine	62.3	umol/L		
				Glucose, serum	4.79	mmol/L		
				Gamma-GT, 37°C	12.9	U/L		
				Potassium	4.31	mmol/L		
				Magnesium	0.83	mmol/L		
				Sodium	136.8	mmol/L		
			Drugs	Amphetamines, Urine	negative			
				Barbiturates, Urine	negative			
				Benzodiazepines, Urine	negative			
				Cannabin., Urine	negative			
				Cocaine, Urine	negative			
				Methadone, Urine	negative			
				Opiates, Urine	negative			
			Haematology	Basophils, abs.	0.04	10^9/L		
				Basophils, %	0.6	%		
				Eosinophils, abs.	0.08	10^9/L		
				Eosinophils, %	1.2	%		
				Haemoglobin	136.0	g/L		
				Haematocrit	0.39	L/L		
				Lymphocytes, abs.	2.08	10^9/L		
				Lymphocytes, %	30.3	%		
				Monocytes, abs.	0.54	10^9/L		
				Monocytes, %	7.9	%		
				Neutrophils, abs.	4.13	10^9/L		
				Neutrophils, %	60.0	%		

Sign.: Significant finding; L: Result considered low; H: Result considered high; SC: Screening; BL: Baseline; D: Day; FUP: Follow-up;

Output generated by program 'NIC002_L16_2_8_Laboratory_V02_0_0'

Listing 16.2.8: Study subject data
Laboratory values

Part C

Cohort	Subject ID	Visit	Type	Measurement	Result		Flagged	Sign.
		BL	Haematology	Platelets	278	10^9/L		
				Erythrocytes	4.34	10^{12}/L		
				Leucocytes	6.87	10^9/L		
			Haemostasis	APTT	24.0	s		
				Prothrombin Time (INR)	0.98	N/A		
				Prothrombin Time (PT)	111.0	%		
			Urine	Beta-HCG, urine	negative			
		D03	Clinical Chemistry	ALT, 37°C	8.9	U/L		
				Alkaline Phosphatase, 37°C	30.1	U/L		
				AST, 37°C	14.3	U/L		
				Bicarbonate	22.6	mmol/L		
				Bilirubin, total	7.2	umol/L		
				Urea/BUN	3.55	mmol/L		
				Calcium	2.31	mmol/L		
				Creatinine	62.8	umol/L		
				Glucose, serum	3.98	mmol/L	L	No
				Gamma-GT, 37°C	13.1	U/L		
				Potassium	4.23	mmol/L		
				Magnesium	0.80	mmol/L		
				Sodium	136.9	mmol/L		
			Haematology	Basophils, abs.	0.05	10^9/L		
				Basophils, %	0.7	%		
				Eosinophils, abs.	0.09	10^9/L		
				Eosinophils, %	1.2	%		
				Haemoglobin	142.0	g/L		
				Haematocrit	0.41	L/L		
				Lymphocytes, abs.	2.77	10^9/L		
				Lymphocytes, %	37.1	%		
				Monocytes, abs.	0.49	10^9/L		
				Monocytes, %	6.6	%		

Sign.: Significant finding; L: Result considered low; H: Result considered high; SC: Screening; BL: Baseline; D: Day; FUP: Follow-up;

Output generated by program 'NIC002_L16_2_8_Laboratory_V02_0_0'

Listing 16.2.8: Study subject data
Laboratory values

Part C

Cohort	Subject ID	Visit	Type	Measurement	Result		Flagged	Sign.
		D03	Haematology	Neutrophils, abs.	4.07	10^9/L		
				Neutrophils, %	54.4	%		
				Platelets	287	10^9/L		
				Erythrocytes	4.51	10^{12}/L		
				Leucocytes	7.47	10^9/L		
			Haemostasis	APTT	24.8	s		
				Prothrombin Time (INR)	0.98	N/A		
				Prothrombin Time (PT)	108.0	%		
		D08	Clinical Chemistry	ALT, 37°C	9.9	U/L		
				Alkaline Phosphatase, 37°C	27.1	U/L	L	No
				AST, 37°C	15.6	U/L		
				Bicarbonate	21.3	mmol/L		
				Bilirubin, total	8.3	umol/L		
				Urea/BUN	3.71	mmol/L		
				Calcium	2.26	mmol/L		
				Creatinine	64.7	umol/L		
				Glucose, serum	4.49	mmol/L		
				Gamma-GT, 37°C	11.6	U/L		
				Potassium	3.92	mmol/L		
				Magnesium	0.79	mmol/L		
				Sodium	134.7	mmol/L	L	No
			Haematology	Basophils, abs.	0.06	10^9/L		
				Basophils, %	0.8	%		
				Eosinophils, abs.	0.07	10^9/L		
				Eosinophils, %	0.9	%		
				Haemoglobin	139.0	g/L		
				Haematocrit	0.40	L/L		
				Lymphocytes, abs.	2.16	10^9/L		
				Lymphocytes, %	28.8	%		
				Monocytes, abs.	0.56	10^9/L		

Sign.: Significant finding; L: Result considered low; H: Result considered high; SC: Screening; BL: Baseline; D: Day; FUP: Follow-up;

Output generated by program 'NIC002_L16_2_8_Laboratory_V02_0_0'

Listing 16.2.8: Study subject data
Laboratory values

Part C

Cohort	Subject ID	Visit	Type	Measurement	Result		Flagged	Sign.
		D08	Haematology	Monocytes, %	7.5	%		
				Neutrophils, abs.	4.64	10^9/L		
				Neutrophils, %	62.0	%		
				Platelets	279	10^9/L		
				Erythrocytes	4.47	10^{12}/L		
				Leucocytes	7.49	10^9/L		
			Haemostasis	APTT	26.4	s		
				Prothrombin Time (INR)	0.97	N/A		
				Prothrombin Time (PT)	111.0	%		
		FUP	Clinical Chemistry	ALT, 37°C	10.6	U/L		
				Alkaline Phosphatase, 37°C	29.7	U/L	L	No
				AST, 37°C	16.5	U/L		
				Bicarbonate	25.7	mmol/L		
				Bilirubin, total	7.4	umol/L		
				Urea/BUN	2.55	mmol/L	L	No
				Calcium	2.29	mmol/L		
				Creatinine	57.9	umol/L		
				Glucose, serum	4.40	mmol/L		
				Gamma-GT, 37°C	11.4	U/L		
				Potassium	4.02	mmol/L		
				Magnesium	0.82	mmol/L		
				Sodium	136.9	mmol/L		
			Haematology	Basophils, abs.	0.05	10^9/L		
				Basophils, %	0.8	%		
				Eosinophils, abs.	0.10	10^9/L		
				Eosinophils, %	1.6	%		
				Haemoglobin	136.0	g/L		
				Haematocrit	0.39	L/L		
				Lymphocytes, abs.	2.34	10^9/L		
				Lymphocytes, %	38.6	%		

Sign.: Significant finding; L: Result considered low; H: Result considered high; SC: Screening; BL: Baseline; D: Day; FUP: Follow-up;

Output generated by program 'NIC002_L16_2_8_Laboratory_V02_0_0'

Listing 16.2.8: Study subject data
Laboratory values

Part C

Cohort	Subject ID	Visit	Type	Measurement	Result		Flagged	Sign.
		FUP	Haematology	Monocytes, abs.	0.46	10^9/L		
				Monocytes, %	7.6	%		
				Neutrophils, abs.	3.12	10^9/L		
				Neutrophils, %	51.4	%		
				Platelets	268	10^9/L		
				Erythrocytes	4.31	10^{12}/L		
				Leucocytes	6.07	10^9/L		
			Haemostasis	APTT	22.7	s		
				Prothrombin Time (INR)	0.88	N/A		
				Prothrombin Time (PT)	> 130.0	%	H	No
	69	SC	Clinical Chemistry	ALT, 37°C	24.6	U/L		
				Alkaline Phosphatase, 37°C	42.0	U/L		
				AST, 37°C	20.5	U/L		
				Bicarbonate	25.2	mmol/L		
				Bilirubin, total	12.2	umol/L		
				Urea/BUN	2.04	mmol/L	L	No
				Calcium	2.29	mmol/L		
				Creatinine	57.2	umol/L		
				Glucose, serum	4.73	mmol/L		
				Gamma-GT, 37°C	27.0	U/L		
				Potassium	3.90	mmol/L		
				Creatinine Clearance MDRD	104	ml/min/1.73m		
				Magnesium	0.82	mmol/L		
				Sodium	136.9	mmol/L		
			Drugs	Amphetamines, Urine	negative			
				Barbiturates, Urine	negative			
				Benzodiazepines, Urine	negative			
				Cannabin., Urine	negative			
				Cocaine, Urine	negative			
				Methadone, Urine	negative			

Sign.: Significant finding; L: Result considered low; H: Result considered high; SC: Screening; BL: Baseline; D: Day; FUP: Follow-up;

Output generated by program 'NIC002_L16_2_8_Laboratory_V02_0_0'

Listing 16.2.8: Study subject data
Laboratory values

Part C

Cohort	Subject ID	Visit	Type	Measurement	Result		Flagged	Sign.
	69	SC	Drugs	Opiates, Urine	negative			
			Haematology	Basophils, abs.	0.03	10^9/L		
				Basophils, %	0.6	%		
				Eosinophils, abs.	0.06	10^9/L		
				Eosinophils, %	1.1	%		
				Haemoglobin	131.0	g/L		
				Haematocrit	0.38	L/L		
				Lymphocytes, abs.	2.11	10^9/L		
				Lymphocytes, %	38.7	%		
				Monocytes, abs.	0.55	10^9/L		
				Monocytes, %	10.1	%		
				Neutrophils, abs.	2.70	10^9/L		
				Neutrophils, %	49.5	%		
				Platelets	216	10^9/L		
				Erythrocytes	4.04	10^{12}/L		
				Leucocytes	5.45	10^9/L		
			Haemostasis	APTT	23.5	s		
				Prothrombin Time (INR)	0.96	N/A		
				Prothrombin Time (PT)	113.0	%		
			Infectious Diseases	HBs-Ag (Hep. B Surf. Ag)	negative	N/A		
				Anti-HCV (Hep. C-AB)	non-reactive	N/A		
				HIV 1+2, AG/AB	negative	N/A		
			Urine	Bilirubin, urine (Stix)	negative			
				Blood (Ery/Hb), urine (Stix)	negative			
				Glucose, urine (Stix)	negative			
				Beta-HCG, urine	negative			
				Ketone, urine (Stix)	negative			
				Leucocytes, urine (Stix)	negative			
				Nitrite, urine (Stix)	negative			
				pH, urine (Stix)	6.0	neg.log[H+]		
				Protein, total, urine (Stix)	negative			

Sign.: Significant finding; L: Result considered low; H: Result considered high; SC: Screening; BL: Baseline; D: Day; FUP: Follow-up;

Output generated by program 'NIC002_L16_2_8_Laboratory_V02_0_0'

Listing 16.2.8: Study subject data
Laboratory values

Part C

Cohort	Subject ID	Visit	Type	Measurement	Result		Flagged	Sign.
	69	SC	Urine	Bacteria, Sediment	negative			
				Carbonate, Sediment	negative			
				Epithelial Cells, Sediment	4	per field	L	No
				Erythrocytes, Sediment	2	per field	H	No
				Casts granul., Sediment	0	per field		
				Casts hyaline, Sediment	0	per field		
				Leucocytes, Sediment	5	per field	H	No
				Oxalate, Sediment	negative			
				Specific Gravity	<=1.005			
				Mucus, Sediment	negative			
				Triple Phosphate, Sediment	negative			
				Urates, Sediment	negative			
				Urobilinogen, urine (Stix)	0.2	mg/dl		
		BL	Clinical Chemistry	ALT, 37°C	25.6	U/L		
				Alkaline Phosphatase, 37°C	41.6	U/L		
				AST, 37°C	20.5	U/L		
				Bicarbonate	23.8	mmol/L		
				Bilirubin, total	6.2	umol/L		
				Urea/BUN	2.73	mmol/L	L	No
				Calcium	2.21	mmol/L		
				Creatinine	60.1	umol/L		
				Glucose, serum	4.72	mmol/L		
				Gamma-GT, 37°C	23.1	U/L		
				Potassium	3.95	mmol/L		
				Magnesium	0.79	mmol/L		
				Sodium	137.3	mmol/L		
			Drugs	Amphetamines, Urine	negative			
				Barbiturates, Urine	negative			
				Benzodiazepines, Urine	negative			
				Cannabin., Urine	negative			

Sign.: Significant finding; L: Result considered low; H: Result considered high; SC: Screening; BL: Baseline; D: Day; FUP: Follow-up;

Output generated by program 'NIC002_L16_2_8_Laboratory_V02_0_0'

Listing 16.2.8: Study subject data
Laboratory values

Part C

Cohort	Subject ID	Visit	Type	Measurement	Result		Flagged	Sign.
		BL	Drugs	Cocaine, Urine	negative			
				Methadone, Urine	negative			
				Opiates, Urine	negative			
			Haematology	Basophils, abs.	0.02	10^9/L		
				Basophils, %	0.4	%		
				Eosinophils, abs.	0.05	10^9/L		
				Eosinophils, %	0.9	%		
				Haemoglobin	122.0	g/L		
				Haematocrit	0.35	L/L		
				Lymphocytes, abs.	1.94	10^9/L		
				Lymphocytes, %	35.5	%		
				Monocytes, abs.	0.41	10^9/L		
				Monocytes, %	7.5	%		
				Neutrophils, abs.	3.05	10^9/L		
				Neutrophils, %	55.7	%		
				Platelets	209	10^9/L		
				Erythrocytes	3.75	10^{12}/L	L	No
				Leucocytes	5.47	10^9/L		
			Haemostasis	APTT	23.9	s		
				Prothrombin Time (INR)	0.93	N/A		
				Prothrombin Time (PT)	125.3	%		
			Urine	Beta-HCG, urine	negative			
		D03	Clinical Chemistry	ALT, 37°C	17.5	U/L		
				Alkaline Phosphatase, 37°C	41.2	U/L		
				AST, 37°C	17.0	U/L		
				Bicarbonate	23.5	mmol/L		
				Bilirubin, total	9.4	umol/L		
				Urea/BUN	2.74	mmol/L	L	No
				Calcium	2.21	mmol/L		
				Creatinine	62.2	umol/L		

Sign.: Significant finding; L: Result considered low; H: Result considered high; SC: Screening; BL: Baseline; D: Day; FUP: Follow-up;

Output generated by program 'NIC002_L16_2_8_Laboratory_V02_0_0'

Listing 16.2.8: Study subject data
Laboratory values

Part C

Cohort	Subject ID	Visit	Type	Measurement	Result		Flagged	Sign.
		D03	Clinical Chemistry	Glucose, serum	4.32	mmol/L		
				Gamma-GT, 37°C	20.8	U/L		
				Potassium	3.72	mmol/L		
				Magnesium	0.80	mmol/L		
				Sodium	137.5	mmol/L		
			Haematology	Basophils, abs.	0.02	10^9/L		
				Basophils, %	0.5	%		
				Eosinophils, abs.	0.06	10^9/L		
				Eosinophils, %	1.4	%		
				Haemoglobin	131.0	g/L		
				Haematocrit	0.37	L/L		
				Lymphocytes, abs.	1.43	10^9/L		
				Lymphocytes, %	32.4	%		
				Monocytes, abs.	0.39	10^9/L		
				Monocytes, %	8.8	%		
				Neutrophils, abs.	2.51	10^9/L		
				Neutrophils, %	56.9	%		
				Platelets	209	10^9/L		
				Erythrocytes	3.96	10^{12}/L	L	No
				Leucocytes	4.41	10^9/L		
			Haemostasis	APTT	24.8	s		
				Prothrombin Time (INR)	0.95	N/A		
				Prothrombin Time (PT)	117.3	%		
		D08	Clinical Chemistry	ALT, 37°C	13.6	U/L		
				Alkaline Phosphatase, 37°C	41.4	U/L		
				AST, 37°C	19.7	U/L		
				Bicarbonate	21.6	mmol/L		
				Bilirubin, total	9.8	umol/L		
				Urea/BUN	2.36	mmol/L	L	No
				Calcium	2.32	mmol/L		

Sign.: Significant finding; L: Result considered low; H: Result considered high; SC: Screening; BL: Baseline; D: Day; FUP: Follow-up;

Output generated by program 'NIC002_L16_2_8_Laboratory_V02_0_0'

Listing 16.2.8: Study subject data
Laboratory values

Part C

Cohort	Subject ID	Visit	Type	Measurement	Result		Flagged	Sign.
		D08	Clinical Chemistry	Creatinine	61.1	umol/L		
				Glucose, serum	4.42	mmol/L		
				Gamma-GT, 37°C	19.4	U/L		
				Potassium	3.80	mmol/L		
				Magnesium	0.81	mmol/L		
				Sodium	135.9	mmol/L	L	No
			Haematology	Basophils, abs.	0.02	10^9/L		
				Basophils, %	0.4	%		
				Eosinophils, abs.	0.05	10^9/L		
				Eosinophils, %	1.1	%		
				Haemoglobin	132.0	g/L		
				Haematocrit	0.38	L/L		
				Lymphocytes, abs.	1.07	10^9/L		
				Lymphocytes, %	22.9	%		
				Monocytes, abs.	0.34	10^9/L		
				Monocytes, %	7.3	%		
				Neutrophils, abs.	3.20	10^9/L		
				Neutrophils, %	68.3	%		
				Platelets	229	10^9/L		
				Erythrocytes	4.03	10^{12}/L		
				Leucocytes	4.68	10^9/L		
			Haemostasis	APTT	26.8	s		
				Prothrombin Time (INR)	0.94	N/A		
				Prothrombin Time (PT)	120.6	%		
		FUP	Clinical Chemistry	ALT, 37°C	19.4	U/L		
				Alkaline Phosphatase, 37°C	39.2	U/L		
				AST, 37°C	22.0	U/L		
				Bicarbonate	20.8	mmol/L	L	No
				Bilirubin, total	10.5	umol/L		
				Urea/BUN	2.48	mmol/L	L	No

Sign.: Significant finding; L: Result considered low; H: Result considered high; SC: Screening; BL: Baseline; D: Day; FUP: Follow-up;

Output generated by program 'NIC002_L16_2_8_Laboratory_V02_0_0'

Listing 16.2.8: Study subject data
Laboratory values

Part C

Cohort	Subject ID	Visit	Type	Measurement	Result		Flagged	Sign.
		FUP	Clinical Chemistry	Calcium	2.22	mmol/L		
				Creatinine	57.2	umol/L		
				Glucose, serum	5.04	mmol/L		
				Gamma-GT, 37°C	19.4	U/L		
				Potassium	3.67	mmol/L		
				Magnesium	0.79	mmol/L		
				Sodium	135.4	mmol/L	L	No
			Haematology	Basophils, abs.	0.02	10^9/L		
				Basophils, %	0.4	%		
				Eosinophils, abs.	0.04	10^9/L		
				Eosinophils, %	0.8	%		
				Haemoglobin	129.0	g/L		
				Haematocrit	0.36	L/L		
				Lymphocytes, abs.	1.44	10^9/L		
				Lymphocytes, %	29.2	%		
				Monocytes, abs.	0.40	10^9/L		
				Monocytes, %	8.1	%		
				Neutrophils, abs.	3.03	10^9/L		
				Neutrophils, %	61.5	%		
				Platelets	249	10^9/L		
				Erythrocytes	3.93	10^{12}/L	L	No
				Leucocytes	4.93	10^9/L		
			Haemostasis	APTT	24.5	s		
				Prothrombin Time (INR)	0.93	N/A		
				Prothrombin Time (PT)	124.1	%		
Group 3	55	SC	Clinical Chemistry	ALT, 37°C	13.3	U/L		
				Alkaline Phosphatase, 37°C	64.5	U/L		
				AST, 37°C	22.7	U/L		
				Bicarbonate	27.4	mmol/L		
				Bilirubin, total	8.2	umol/L		

Sign.: Significant finding; L: Result considered low; H: Result considered high; SC: Screening; BL: Baseline; D: Day; FUP: Follow-up;

Output generated by program 'NIC002_L16_2_8_Laboratory_V02_0_0'

Listing 16.2.8: Study subject data
Laboratory values

Part C

Cohort	Subject ID	Visit	Type	Measurement	Result		Flagged	Sign.
Group 3	55	SC	Clinical Chemistry	Urea/BUN	3.74	mmol/L		
				Calcium	2.37	mmol/L		
				Creatinine	72.3	umol/L		
				Creatinine	59.8	umol/L		
				Glucose, serum	5.64	mmol/L		
				Gamma-GT, 37°C	11.5	U/L		
				Potassium	4.50	mmol/L		
				Creatinine Clearance MDRD	80	ml/min/1.73m		
				Creatinine Clearance MDRD	99	ml/min/1.73m		
				Magnesium	0.87	mmol/L		
				Sodium	138.4	mmol/L		
			Drugs	Amphetamines, Urine	negative			
				Barbiturates, Urine	negative			
				Benzodiazepines, Urine	negative			
				Cannabin., Urine	negative			
				Cocaine, Urine	negative			
				Methadone, Urine	negative			
				Opiates, Urine	negative			
			Haematology	Basophils, abs.	0.01	10^9/L		
				Basophils, %	0.2	%		
				Eosinophils, abs.	0.15	10^9/L		
				Eosinophils, %	2.9	%		
				Haemoglobin	121.0	g/L		
				Haematocrit	0.35	L/L		
				Lymphocytes, abs.	1.41	10^9/L		
				Lymphocytes, %	27.6	%		
				Monocytes, abs.	0.35	10^9/L		
				Monocytes, %	6.9	%		
				Neutrophils, abs.	3.18	10^9/L		
				Neutrophils, %	62.4	%		
				Platelets	213	10^9/L		

Sign.: Significant finding; L: Result considered low; H: Result considered high; SC: Screening; BL: Baseline; D: Day; FUP: Follow-up;

Output generated by program 'NIC002_L16_2_8_Laboratory_V02_0_0'

Listing 16.2.8: Study subject data
Laboratory values

Part C

Cohort	Subject ID	Visit	Type	Measurement	Result		Flagged	Sign.
Group 3	55	SC	Haematology	Erythrocytes	3.80	10^{12}/L	L	No
				Leucocytes	5.10	10^{9}/L		
			Haemostasis	APTT	22.6	s		
				Prothrombin Time (INR)	1.02	N/A		
				Prothrombin Time (PT)	96.3	%		
			Infectious Diseases	HBs-Ag (Hep. B Surf. Ag)	negative	N/A		
				Anti-HCV (Hep. C-AB)	non-reactive	N/A		
				HIV 1+2, AG/AB	negative	N/A		
			Urine	Bilirubin, urine (Stix)	negative			
				Blood (Ery/Hb), urine (Stix)	negative			
				Glucose, urine (Stix)	negative			
				Beta-HCG, urine	negative			
				Ketone, urine (Stix)	negative			
				Leucocytes, urine (Stix)	negative			
				Nitrite, urine (Stix)	negative			
				pH, urine (Stix)	6.0	neg.log[H+]		
				Protein, total, urine (Stix)	negative			
				Specific Gravity	1.025			
				Urobilinogen, urine (Stix)	0.2	mg/dl		
		Unscheduled	Clinical Chemistry	Creatinine	59.8	umol/L		
				Creatinine Clearance MDRD	99	ml/min/1.73m		
		BL	Clinical Chemistry	ALT, 37°C	14.2	U/L		
				Alkaline Phosphatase, 37°C	67.7	U/L		
				AST, 37°C	23.6	U/L		
				Bicarbonate	25.7	mmol/L		
				Bilirubin, total	6.1	umol/L		
				Urea/BUN	3.63	mmol/L		
				Calcium	2.31	mmol/L		
				Creatinine	64.4	umol/L		

Sign.: Significant finding; L: Result considered low; H: Result considered high; SC: Screening; BL: Baseline; D: Day; FUP: Follow-up;

Output generated by program 'NIC002_L16_2_8_Laboratory_V02_0_0'

Listing 16.2.8: Study subject data
Laboratory values

Part C

Cohort	Subject ID	Visit	Type	Measurement	Result		Flagged	Sign.
		BL	Clinical Chemistry	Glucose, serum	4.92	mmol/L		
				Gamma-GT, 37°C	10.8	U/L		
				Potassium	3.80	mmol/L		
				Magnesium	0.85	mmol/L		
				Sodium	134.2	mmol/L	L	No
			Drugs	Amphetamines, Urine	negative			
				Barbiturates, Urine	negative			
				Benzodiazepines, Urine	negative			
				Cannabin., Urine	negative			
				Cocaine, Urine	negative			
				Methadone, Urine	negative			
				Opiates, Urine	negative			
			Haematology	Basophils, abs.	0.03	10^9/L		
				Basophils, %	0.5	%		
				Eosinophils, abs.	0.19	10^9/L		
				Eosinophils, %	3.0	%		
				Haemoglobin	122.0	g/L		
				Haematocrit	0.36	L/L		
				Lymphocytes, abs.	1.57	10^9/L		
				Lymphocytes, %	24.5	%		
				Monocytes, abs.	0.52	10^9/L		
				Monocytes, %	8.1	%		
				Neutrophils, abs.	4.11	10^9/L		
				Neutrophils, %	63.9	%		
				Platelets	182	10^9/L		
				Erythrocytes	3.94	10^{12}/L	L	No
				Leucocytes	6.42	10^9/L		
			Haemostasis	APTT	23.6	s		
				Prothrombin Time (INR)	1.00	N/A		
				Prothrombin Time (PT)	101.5	%		
			Urine	Beta-HCG, urine	negative			

Sign.: Significant finding; L: Result considered low; H: Result considered high; SC: Screening; BL: Baseline; D: Day; FUP: Follow-up;

Output generated by program 'NIC002_L16_2_8_Laboratory_V02_0_0'

Listing 16.2.8: Study subject data
Laboratory values

Part C

Cohort	Subject ID	Visit	Type	Measurement	Result		Flagged	Sign.
		D03	Clinical Chemistry	ALT, 37°C	11.6	U/L		
				Alkaline Phosphatase, 37°C	61.4	U/L		
				AST, 37°C	19.3	U/L		
				Bicarbonate	28.7	mmol/L		
				Bilirubin, total	7.8	umol/L		
				Urea/BUN	3.35	mmol/L		
				Calcium	2.38	mmol/L		
				Creatinine	56.4	umol/L		
				Glucose, serum	4.86	mmol/L		
				Gamma-GT, 37°C	11.4	U/L		
				Potassium	4.26	mmol/L		
				Magnesium	0.89	mmol/L		
				Sodium	137.3	mmol/L		
			Haematology	Basophils, abs.	0.03	10^9/L		
				Basophils, %	0.5	%		
				Eosinophils, abs.	0.13	10^9/L		
				Eosinophils, %	2.1	%		
				Haemoglobin	125.0	g/L		
				Haematocrit	0.37	L/L		
				Lymphocytes, abs.	1.51	10^9/L		
				Lymphocytes, %	24.9	%		
				Monocytes, abs.	0.45	10^9/L		
				Monocytes, %	7.4	%		
				Neutrophils, abs.	3.94	10^9/L		
				Neutrophils, %	65.1	%		
				Platelets	194	10^9/L		
				Erythrocytes	3.98	10^{12}/L	L	No
				Leucocytes	6.06	10^9/L		
			Haemostasis	APTT	23.5	s		
				Prothrombin Time (INR)	1.02	N/A		

Sign.: Significant finding; L: Result considered low; H: Result considered high; SC: Screening; BL: Baseline; D: Day; FUP: Follow-up;

Output generated by program 'NIC002_L16_2_8_Laboratory_V02_0_0'

Listing 16.2.8: Study subject data
Laboratory values

Part C

Cohort	Subject ID	Visit	Type	Measurement	Result		Flagged	Sign.
		D03	Haemostasis	Prothrombin Time (PT)	96.3	%		
		D08	Clinical Chemistry	ALT, 37°C	13.1	U/L		
				Alkaline Phosphatase, 37°C	60.0	U/L		
				AST, 37°C	20.4	U/L		
				Bicarbonate	26.0	mmol/L		
				Bilirubin, total	7.3	umol/L		
				Urea/BUN	3.11	mmol/L		
				Calcium	2.39	mmol/L		
				Creatinine	54.6	umol/L		
				Glucose, serum	5.09	mmol/L		
				Gamma-GT, 37°C	10.8	U/L		
				Potassium	3.86	mmol/L		
				Magnesium	0.85	mmol/L		
				Sodium	137.8	mmol/L		
			Haematology	Basophils, abs.	0.02	10^9/L		
				Basophils, %	0.3	%		
				Eosinophils, abs.	0.08	10^9/L		
				Eosinophils, %	1.3	%		
				Haemoglobin	119.0	g/L		
				Haematocrit	0.34	L/L	L	No
				Lymphocytes, abs.	1.44	10^9/L		
				Lymphocytes, %	22.7	%		
				Monocytes, abs.	0.41	10^9/L		
				Monocytes, %	6.5	%		
				Neutrophils, abs.	4.40	10^9/L		
				Neutrophils, %	69.2	%		
				Platelets	195	10^9/L		
				Erythrocytes	3.72	10^{12}/L	L	No
				Leucocytes	6.35	10^9/L		
			Haemostasis	APTT	23.3	s		

Sign.: Significant finding; L: Result considered low; H: Result considered high; SC: Screening; BL: Baseline; D: Day; FUP: Follow-up;

Output generated by program 'NIC002_L16_2_8_Laboratory_V02_0_0'

Listing 16.2.8: Study subject data
Laboratory values

Part C

Cohort	Subject ID	Visit	Type	Measurement	Result		Flagged	Sign.
		D08	Haemostasis	Prothrombin Time (INR)	1.01	N/A		
				Prothrombin Time (PT)	98.8	%		
		FUP	Clinical Chemistry	ALT, 37°C	16.5	U/L		
				Alkaline Phosphatase, 37°C	62.3	U/L		
				AST, 37°C	24.9	U/L		
				Bicarbonate	28.0	mmol/L		
				Bilirubin, total	9.9	umol/L		
				Urea/BUN	3.31	mmol/L		
				Calcium	2.40	mmol/L		
				Creatinine	59.5	umol/L		
				Glucose, serum	4.71	mmol/L		
				Gamma-GT, 37°C	10.7	U/L		
				Potassium	3.95	mmol/L		
				Magnesium	0.85	mmol/L		
				Sodium	137.2	mmol/L		
			Haematology	Basophils, abs.	0.02	10^9/L		
				Basophils, %	0.4	%		
				Eosinophils, abs.	0.06	10^9/L		
				Eosinophils, %	1.2	%		
				Haemoglobin	115.0	g/L		
				Haematocrit	0.33	L/L	L	No
				Lymphocytes, abs.	1.31	10^9/L		
				Lymphocytes, %	26.7	%		
				Monocytes, abs.	0.39	10^9/L		
				Monocytes, %	8.0	%		
				Neutrophils, abs.	3.12	10^9/L		
				Neutrophils, %	63.7	%		
				Platelets	201	10^9/L		
				Erythrocytes	3.65	10^{12}/L	L	No
				Leucocytes	4.90	10^9/L		

Sign.: Significant finding; L: Result considered low; H: Result considered high; SC: Screening; BL: Baseline; D: Day; FUP: Follow-up;

Output generated by program 'NIC002_L16_2_8_Laboratory_V02_0_0'

Listing 16.2.8: Study subject data
Laboratory values

Part C

Cohort	Subject ID	Visit	Type	Measurement	Result		Flagged	Sign.
		FUP	Haemostasis	APTT	22.7	s		
				Prothrombin Time (INR)	1.01	N/A		
				Prothrombin Time (PT)	101.7	%		
	62	SC	Clinical Chemistry	ALT, 37°C	11.8	U/L		
				Alkaline Phosphatase, 37°C	44.6	U/L		
				AST, 37°C	16.1	U/L		
				Bicarbonate	25.6	mmol/L		
				Bilirubin, total	10.6	umol/L		
				Urea/BUN	2.54	mmol/L	L	No
				Calcium	2.30	mmol/L		
				Creatinine	59.5	umol/L		
				Glucose, serum	4.69	mmol/L		
				Gamma-GT, 37°C	11.2	U/L		
				Potassium	4.40	mmol/L		
				Creatinine Clearance MDRD	97	ml/min/1.73m		
				Magnesium	0.73	mmol/L	L	No
				Sodium	134.6	mmol/L	L	No
			Drugs	Amphetamines, Urine	negative			
				Barbiturates, Urine	negative			
				Benzodiazepines, Urine	negative			
				Cannabin., Urine	negative			
				Cocaine, Urine	negative			
				Methadone, Urine	negative			
				Opiates, Urine	negative			
			Haematology	Basophils, abs.	0.04	10^9/L		
				Basophils, %	0.6	%		
				Eosinophils, abs.	0.12	10^9/L		
				Eosinophils, %	1.8	%		
				Haemoglobin	118.0	g/L		
				Haematocrit	0.34	L/L	L	No

Sign.: Significant finding; L: Result considered low; H: Result considered high; SC: Screening; BL: Baseline; D: Day; FUP: Follow-up;

Output generated by program 'NIC002_L16_2_8_Laboratory_V02_0_0'

Listing 16.2.8: Study subject data
Laboratory values

Part C

Cohort	Subject ID	Visit	Type	Measurement	Result		Flagged	Sign.
	62	SC	Haematology	Lymphocytes, abs.	2.07	10^9/L		
				Lymphocytes, %	30.5	%		
				Monocytes, abs.	0.62	10^9/L		
				Monocytes, %	9.1	%		
				Neutrophils, abs.	3.93	10^9/L		
				Neutrophils, %	58.0	%		
				Platelets	197	10^9/L		
				Erythrocytes	3.70	10^{12}/L	L	No
				Leucocytes	6.78	10^9/L		
			Haemostasis	APTT	23.4	s		
				Prothrombin Time (INR)	1.08	N/A		
				Prothrombin Time (PT)	84.9	%		
			Infectious Diseases	HBs-Ag (Hep. B Surf. Ag)	negative	N/A		
				Anti-HCV (Hep. C-AB)	non-reactive	N/A		
				HIV 1+2, AG/AB	negative	N/A		
			Urine	Bilirubin, urine (Stix)	negative			
				Blood (Ery/Hb), urine (Stix)	trace-intact		H	No
				Glucose, urine (Stix)	negative			
				Beta-HCG, urine	negative			
				Ketone, urine (Stix)	negative			
				Leucocytes, urine (Stix)	negative			
				Nitrite, urine (Stix)	negative			
				pH, urine (Stix)	6.0	neg.log[H+]		
				Protein, total, urine (Stix)	negative			
				Bacteria, Sediment	positive		H	No
				Carbonate, Sediment	negative			
				Epithelial Cells, Sediment	3	per field	L	No
				Erythrocytes, Sediment	0	per field		
				Casts granul., Sediment	0	per field		
				Casts hyaline, Sediment	0	per field		
				Leucocytes, Sediment	0	per field		

Sign.: Significant finding; L: Result considered low; H: Result considered high; SC: Screening; BL: Baseline; D: Day; FUP: Follow-up;

Output generated by program 'NIC002_L16_2_8_Laboratory_V02_0_0'

Listing 16.2.8: Study subject data
Laboratory values

Part C

Cohort	Subject ID	Visit	Type	Measurement	Result		Flagged	Sign.
	62	SC	Urine	Oxalate, Sediment	negative			
				Specific Gravity	<=1.005			
				Mucus, Sediment	negative			
				Triple Phosphate, Sediment	negative			
				Urates, Sediment	negative			
				Urobilinogen, urine (Stix)	0.2	mg/dl		
		BL	Clinical Chemistry	ALT, 37°C	13.4	U/L		
				Alkaline Phosphatase, 37°C	46.3	U/L		
				AST, 37°C	15.5	U/L		
				Bicarbonate	25.4	mmol/L		
				Bilirubin, total	7.2	umol/L		
				Urea/BUN	2.22	mmol/L	L	No
				Calcium	2.18	mmol/L	L	No
				Creatinine	56.7	umol/L		
				Glucose, serum	4.33	mmol/L		
				Gamma-GT, 37°C	10.1	U/L		
				Potassium	4.51	mmol/L		
				Magnesium	0.83	mmol/L		
				Sodium	133.3	mmol/L	L	No
			Drugs	Amphetamines, Urine	negative			
				Barbiturates, Urine	negative			
				Benzodiazepines, Urine	negative			
				Cannabin., Urine	negative			
				Cocaine, Urine	negative			
				Methadone, Urine	negative			
				Opiates, Urine	negative			
			Haematology	Basophils, abs.	0.05	10^9/L		
				Basophils, %	0.7	%		
				Eosinophils, abs.	0.13	10^9/L		
				Eosinophils, %	1.7	%		

Sign.: Significant finding; L: Result considered low; H: Result considered high; SC: Screening; BL: Baseline; D: Day; FUP: Follow-up;

Output generated by program 'NIC002_L16_2_8_Laboratory_V02_0_0'

Listing 16.2.8: Study subject data
Laboratory values

Part C

Cohort	Subject ID	Visit	Type	Measurement	Result		Flagged	Sign.
		BL	Haematology	Haemoglobin	124.0	g/L		
				Haematocrit	0.37	L/L		
				Lymphocytes, abs.	2.03	10^9/L		
				Lymphocytes, %	26.5	%		
				Monocytes, abs.	0.56	10^9/L		
				Monocytes, %	7.3	%		
				Neutrophils, abs.	4.88	10^9/L		
				Neutrophils, %	63.8	%		
				Platelets	215	10^9/L		
				Erythrocytes	3.94	10^{12}/L	L	No
				Leucocytes	7.65	10^9/L		
			Haemostasis	APTT	22.7	s		
				Prothrombin Time (INR)	1.02	N/A		
				Prothrombin Time (PT)	96.3	%		
			Urine	Beta-HCG, urine	negative			
		D03	Clinical Chemistry	ALT, 37°C	11.1	U/L		
				Alkaline Phosphatase, 37°C	42.1	U/L		
				AST, 37°C	13.0	U/L		
				Bicarbonate	27.7	mmol/L		
				Bilirubin, total	7.8	umol/L		
				Urea/BUN	2.98	mmol/L		
				Calcium	2.22	mmol/L		
				Creatinine	51.1	umol/L		
				Glucose, serum	4.11	mmol/L		
				Gamma-GT, 37°C	9.4	U/L		
				Potassium	4.55	mmol/L		
				Magnesium	0.74	mmol/L	L	No
				Sodium	137.4	mmol/L		
			Haematology	Basophils, abs.	0.03	10^9/L		
				Basophils, %	0.5	%		

Sign.: Significant finding; L: Result considered low; H: Result considered high; SC: Screening; BL: Baseline; D: Day; FUP: Follow-up;

Output generated by program 'NIC002_L16_2_8_Laboratory_V02_0_0'

Listing 16.2.8: Study subject data
Laboratory values

Part C

Cohort	Subject ID	Visit	Type	Measurement	Result		Flagged	Sign.
		D03	Haematology	Eosinophils, abs.	0.16	10^9/L		
				Eosinophils, %	2.8	%		
				Haemoglobin	119.0	g/L		
				Haematocrit	0.35	L/L		
				Lymphocytes, abs.	2.05	10^9/L		
				Lymphocytes, %	35.8	%		
				Monocytes, abs.	0.44	10^9/L		
				Monocytes, %	7.7	%		
				Neutrophils, abs.	3.04	10^9/L		
				Neutrophils, %	53.2	%		
				Platelets	212	10^9/L		
				Erythrocytes	3.80	10^{12}/L	L	No
				Leucocytes	5.72	10^9/L		
			Haemostasis	APTT	23.6	s		
				Prothrombin Time (INR)	1.06	N/A		
				Prothrombin Time (PT)	89.2	%		
		D08	Clinical Chemistry	ALT, 37°C	22.0	U/L		
				Alkaline Phosphatase, 37°C	38.4	U/L		
				AST, 37°C	19.7	U/L		
				Bicarbonate	25.7	mmol/L		
				Bilirubin, total	8.5	umol/L		
				Urea/BUN	3.05	mmol/L		
				Calcium	2.14	mmol/L	L	No
				Creatinine	52.0	umol/L		
				Glucose, serum	4.13	mmol/L		
				Gamma-GT, 37°C	8.0	U/L		
				Potassium	3.92	mmol/L		
				Magnesium	0.73	mmol/L	L	No
				Sodium	137.3	mmol/L		
			Haematology	Basophils, abs.	0.03	10^9/L		

Sign.: Significant finding; L: Result considered low; H: Result considered high; SC: Screening; BL: Baseline; D: Day; FUP: Follow-up;

Output generated by program 'NIC002_L16_2_8_Laboratory_V02_0_0'

Listing 16.2.8: Study subject data
Laboratory values

Part C

Cohort	Subject ID	Visit	Type	Measurement	Result		Flagged	Sign.
		D08	Haematology	Basophils, %	0.5	%		
				Eosinophils, abs.	0.13	10^9/L		
				Eosinophils, %	2.2	%		
				Haemoglobin	109.0	g/L	L	No
				Haematocrit	0.32	L/L	L	No
				Lymphocytes, abs.	1.86	10^9/L		
				Lymphocytes, %	31.2	%		
				Monocytes, abs.	0.57	10^9/L		
				Monocytes, %	9.6	%		
				Neutrophils, abs.	3.37	10^9/L		
				Neutrophils, %	56.5	%		
				Platelets	211	10^9/L		
				Erythrocytes	3.42	10^{12}/L	L	No
				Leucocytes	5.96	10^9/L		
			Haemostasis	APTT	24.0	s		
				Prothrombin Time (INR)	1.02	N/A		
				Prothrombin Time (PT)	96.3	%		
		FUP	Clinical Chemistry	ALT, 37°C	20.2	U/L		
				Alkaline Phosphatase, 37°C	40.3	U/L		
				AST, 37°C	17.1	U/L		
				Bicarbonate	28.0	mmol/L		
				Bilirubin, total	9.2	umol/L		
				Urea/BUN	2.49	mmol/L	L	No
				Calcium	2.17	mmol/L	L	No
				Creatinine	52.6	umol/L		
				Glucose, serum	4.08	mmol/L	L	No
				Gamma-GT, 37°C	9.0	U/L		
				Potassium	3.92	mmol/L		
				Magnesium	0.74	mmol/L	L	No
				Sodium	135.1	mmol/L	L	No

Sign.: Significant finding; L: Result considered low; H: Result considered high; SC: Screening; BL: Baseline; D: Day; FUP: Follow-up;

Output generated by program 'NIC002_L16_2_8_Laboratory_V02_0_0'

Listing 16.2.8: Study subject data
Laboratory values

Part C

Cohort	Subject ID	Visit	Type	Measurement	Result		Flagged	Sign.
		FUP	Haematology	Basophils, abs.	0.04	10^9/L		
				Basophils, %	0.7	%		
				Eosinophils, abs.	0.15	10^9/L		
				Eosinophils, %	2.8	%		
				Haemoglobin	112.0	g/L		
				Haematocrit	0.33	L/L	L	No
				Lymphocytes, abs.	1.82	10^9/L		
				Lymphocytes, %	33.6	%		
				Monocytes, abs.	0.60	10^9/L		
				Monocytes, %	11.1	%		
				Neutrophils, abs.	2.81	10^9/L		
				Neutrophils, %	51.8	%		
				Platelets	212	10^9/L		
				Erythrocytes	3.50	10^{12}/L	L	No
				Leucocytes	5.42	10^9/L		
			Haemostasis	APTT	23.8	s		
				Prothrombin Time (INR)	1.03	N/A		
				Prothrombin Time (PT)	98.8	%		
	65	SC	Clinical Chemistry	ALT, 37°C	12.4	U/L		
				Alkaline Phosphatase, 37°C	38.1	U/L		
				AST, 37°C	21.8	U/L		
				Bicarbonate	25.7	mmol/L		
				Bilirubin, total	21.6	umol/L	H	No
				Urea/BUN	2.72	mmol/L	L	No
				Calcium	2.31	mmol/L		
				Creatinine	51.1	umol/L		
				Glucose, serum	4.99	mmol/L		
				Gamma-GT, 37°C	10.0	U/L		
				Potassium	3.98	mmol/L		
				Creatinine Clearance MDRD	119	ml/min/1.73m		

Sign.: Significant finding; L: Result considered low; H: Result considered high; SC: Screening; BL: Baseline; D: Day; FUP: Follow-up;

Output generated by program 'NIC002_L16_2_8_Laboratory_V02_0_0'

Listing 16.2.8: Study subject data
Laboratory values

Part C

Cohort	Subject ID	Visit	Type	Measurement	Result		Flagged	Sign.
	65	SC	Clinical Chemistry	Magnesium	0.82	mmol/L		
				Sodium	136.0	mmol/L		
			Drugs	Amphetamines, Urine	negative			
				Barbiturates, Urine	negative			
				Benzodiazepines, Urine	negative			
				Cannabin., Urine	negative			
				Cocaine, Urine	negative			
				Methadone, Urine	negative			
				Opiates, Urine	negative			
			Haematology	Basophils, abs.	0.05	10^9/L		
				Basophils, %	1.0	%		
				Eosinophils, abs.	0.20	10^9/L		
				Eosinophils, %	4.1	%		
				Haemoglobin	116.0	g/L		
				Haematocrit	0.33	L/L	L	No
				Lymphocytes, abs.	1.71	10^9/L		
				Lymphocytes, %	35.4	%		
				Monocytes, abs.	0.65	10^9/L		
				Monocytes, %	13.5	%		
				Neutrophils, abs.	2.22	10^9/L		
				Neutrophils, %	46.0	%		
				Platelets	346	10^9/L		
				Erythrocytes	3.74	10^{12}/L	L	No
				Leucocytes	4.83	10^9/L		
			Haemostasis	APTT	28.8	s	H	No
				Prothrombin Time (INR)	1.08	N/A		
				Prothrombin Time (PT)	84.9	%		
			Infectious Diseases	HBs-Ag (Hep. B Surf. Ag)	negative	N/A		
				Anti-HCV (Hep. C-AB)	non-reactive	N/A		
				HIV 1+2, AG/AB	negative	N/A		
			Urine	Bilirubin, urine (Stix)	negative			

Sign.: Significant finding; L: Result considered low; H: Result considered high; SC: Screening; BL: Baseline; D: Day; FUP: Follow-up;

Output generated by program 'NIC002_L16_2_8_Laboratory_V02_0_0'

Listing 16.2.8: Study subject data
Laboratory values

Part C

Cohort	Subject ID	Visit	Type	Measurement	Result		Flagged	Sign.
	65	SC	Urine	Blood (Ery/Hb), urine (Stix)	1+		H	No
				Glucose, urine (Stix)	negative			
				Beta-HCG, urine	negative			
				Ketone, urine (Stix)	negative			
				Leucocytes, urine (Stix)	negative			
				Nitrite, urine (Stix)	negative			
				pH, urine (Stix)	5.5	neg.log[H+]		
				Protein, total, urine (Stix)	negative			
				Bacteria, Sediment	positive		H	No
				Carbonate, Sediment	negative			
				Epithelial Cells, Sediment	6	per field		
				Erythrocytes, Sediment	2	per field	H	No
				Casts granul., Sediment	0	per field		
				Casts hyaline, Sediment	0	per field		
				Leucocytes, Sediment	2	per field		
				Oxalate, Sediment	negative			
				Specific Gravity	1.025			
				Mucus, Sediment	positive		H	No
				Triple Phosphate, Sediment	negative			
				Urates, Sediment	negative			
				Urobilinogen, urine (Stix)	0.2	mg/dl		
		BL	Clinical Chemistry	ALT, 37°C	12.7	U/L		
				Alkaline Phosphatase, 37°C	34.6	U/L		
				AST, 37°C	22.3	U/L		
				Bicarbonate	27.7	mmol/L		
				Bilirubin, total	16.1	umol/L		
				Urea/BUN	2.63	mmol/L	L	No
				Calcium	2.28	mmol/L		
				Creatinine	50.3	umol/L		
				Glucose, serum	4.74	mmol/L		

Sign.: Significant finding; L: Result considered low; H: Result considered high; SC: Screening; BL: Baseline; D: Day; FUP: Follow-up;

Output generated by program 'NIC002_L16_2_8_Laboratory_V02_0_0'

Listing 16.2.8: Study subject data
Laboratory values

Part C

Cohort	Subject ID	Visit	Type	Measurement	Result		Flagged	Sign.
		BL	Clinical Chemistry	Gamma-GT, 37°C	9.2	U/L		
				Potassium	3.96	mmol/L		
				Magnesium	0.80	mmol/L		
				Sodium	137.9	mmol/L		
			Drugs	Amphetamines, Urine	negative			
				Barbiturates, Urine	negative			
				Benzodiazepines, Urine	negative			
				Cannabin., Urine	negative			
				Cocaine, Urine	negative			
				Methadone, Urine	negative			
				Opiates, Urine	negative			
			Haematology	Basophils, abs.	0.07	10^9/L		
				Basophils, %	1.7	%	H	No
				Eosinophils, abs.	0.16	10^9/L		
				Eosinophils, %	3.8	%		
				Haemoglobin	107.0	g/L	L	No
				Haematocrit	0.32	L/L	L	No
				Lymphocytes, abs.	1.58	10^9/L		
				Lymphocytes, %	37.5	%		
				Monocytes, abs.	0.43	10^9/L		
				Monocytes, %	10.2	%		
				Neutrophils, abs.	1.97	10^9/L		
				Neutrophils, %	46.8	%		
				Platelets	392	10^9/L	H	No
				Erythrocytes	3.50	10^{12}/L	L	No
				Leucocytes	4.21	10^9/L		
			Haemostasis	APTT	28.0	s		
				Prothrombin Time (INR)	1.09	N/A		
				Prothrombin Time (PT)	83.7	%		
			Urine	Beta-HCG, urine	negative			

Sign.: Significant finding; L: Result considered low; H: Result considered high; SC: Screening; BL: Baseline; D: Day; FUP: Follow-up;

Output generated by program 'NIC002_L16_2_8_Laboratory_V02_0_0'

Listing 16.2.8: Study subject data
Laboratory values

Part C

Cohort	Subject ID	Visit	Type	Measurement	Result		Flagged	Sign.
		D03	Clinical Chemistry	ALT, 37°C	13.0	U/L		
				Alkaline Phosphatase, 37°C	34.6	U/L		
				AST, 37°C	19.0	U/L		
				Bicarbonate	27.6	mmol/L		
				Bilirubin, total	16.3	umol/L		
				Urea/BUN	3.31	mmol/L		
				Calcium	2.28	mmol/L		
				Creatinine	46.7	umol/L		
				Glucose, serum	4.37	mmol/L		
				Gamma-GT, 37°C	9.2	U/L		
				Potassium	4.46	mmol/L		
				Magnesium	0.74	mmol/L	L	No
				Sodium	137.2	mmol/L		
			Haematology	Basophils, abs.	0.06	10^9/L		
				Basophils, %	1.3	%		
				Eosinophils, abs.	0.19	10^9/L		
				Eosinophils, %	4.3	%		
				Haemoglobin	116.0	g/L		
				Haematocrit	0.34	L/L	L	No
				Lymphocytes, abs.	1.53	10^9/L		
				Lymphocytes, %	34.3	%		
				Monocytes, abs.	0.48	10^9/L		
				Monocytes, %	10.8	%		
				Neutrophils, abs.	2.20	10^9/L		
				Neutrophils, %	49.3	%		
				Platelets	399	10^9/L	H	No
				Erythrocytes	3.73	10^{12}/L	L	No
				Leucocytes	4.46	10^9/L		
			Haemostasis	APTT	28.3	s	H	No
				Prothrombin Time (INR)	1.05	N/A		
				Prothrombin Time (PT)	92.3	%		

Sign.: Significant finding; L: Result considered low; H: Result considered high; SC: Screening; BL: Baseline; D: Day; FUP: Follow-up;

Output generated by program 'NIC002_L16_2_8_Laboratory_V02_0_0'

Listing 16.2.8: Study subject data
Laboratory values

Part C

Cohort	Subject ID	Visit	Type	Measurement	Result		Flagged	Sign.
		D08	Clinical Chemistry	ALT, 37°C	17.7	U/L		
				Alkaline Phosphatase, 37°C	34.3	U/L		
				AST, 37°C	25.0	U/L		
				Bicarbonate	27.5	mmol/L		
				Bilirubin, total	16.7	umol/L		
				Urea/BUN	2.93	mmol/L		
				Calcium	2.32	mmol/L		
				Creatinine	49.2	umol/L		
				Glucose, serum	4.67	mmol/L		
				Gamma-GT, 37°C	9.8	U/L		
				Potassium	4.35	mmol/L		
				Magnesium	0.77	mmol/L		
				Sodium	138.4	mmol/L		
			Haematology	Basophils, abs.	see remark	10^9/L		
				Basophils, %	see remark	%		
				Eosinophils, abs.	see remark	10^9/L		
				Eosinophils, %	see remark	%		
				Haemoglobin	see remark	g/L		
				Haematocrit	see remark	L/L		
				Lymphocytes, abs.	see remark	10^9/L		
				Lymphocytes, %	see remark	%		
				Monocytes, abs.	see remark	10^9/L		
				Monocytes, %	see remark	%		
				Neutrophils, abs.	see remark	10^9/L		
				Neutrophils, %	see remark	%		
				Platelets	see remark	10^9/L		
				Erythrocytes	see remark	10^{12}/L		
				Leucocytes	see remark	10^9/L		
			Haemostasis	APTT	28.8	s	H	No
				Prothrombin Time (INR)	1.02	N/A		

Sign.: Significant finding; L: Result considered low; H: Result considered high; SC: Screening; BL: Baseline; D: Day; FUP: Follow-up;

Output generated by program 'NIC002_L16_2_8_Laboratory_V02_0_0'

Listing 16.2.8: Study subject data
Laboratory values

Part C

Cohort	Subject ID	Visit	Type	Measurement	Result		Flagged	Sign.
		D08	Haemostasis	Prothrombin Time (PT)	97.2	%		
		FUP	Clinical Chemistry	ALT, 37°C	16.3	U/L		
				Alkaline Phosphatase, 37°C	33.9	U/L		
				AST, 37°C	22.7	U/L		
				Bicarbonate	26.0	mmol/L		
				Bilirubin, total	24.8	umol/L	H	No
				Urea/BUN	3.23	mmol/L		
				Calcium	2.27	mmol/L		
				Creatinine	54.0	umol/L		
				Glucose, serum	4.98	mmol/L		
				Gamma-GT, 37°C	9.3	U/L		
				Potassium	4.08	mmol/L		
				Magnesium	0.81	mmol/L		
				Sodium	137.7	mmol/L		
			Haematology	Basophils, abs.	0.06	10^9/L		
				Basophils, %	1.3	%		
				Eosinophils, abs.	0.19	10^9/L		
				Eosinophils, %	4.1	%		
				Haemoglobin	105.0	g/L	L	No
				Haematocrit	0.31	L/L	L	No
				Lymphocytes, abs.	1.92	10^9/L		
				Lymphocytes, %	41.1	%		
				Monocytes, abs.	0.74	10^9/L		
				Monocytes, %	15.8	%	H	No
				Neutrophils, abs.	1.76	10^9/L		
				Neutrophils, %	37.7	%	L	No
				Platelets	378	10^9/L	H	No
				Erythrocytes	3.38	10^{12}/L	L	No
				Leucocytes	4.67	10^9/L		
			Haemostasis	APTT	27.9	s		

Sign.: Significant finding; L: Result considered low; H: Result considered high; SC: Screening; BL: Baseline; D: Day; FUP: Follow-up;

Output generated by program 'NIC002_L16_2_8_Laboratory_V02_0_0'

Listing 16.2.8: Study subject data
Laboratory values

Part C

Cohort	Subject ID	Visit	Type	Measurement	Result		Flagged	Sign.
		FUP	Haemostasis	Prothrombin Time (INR)	1.04	N/A		
				Prothrombin Time (PT)	94.7	%		
	66	SC	Clinical Chemistry	ALT, 37°C	10.1	U/L		
				Alkaline Phosphatase, 37°C	53.5	U/L		
				AST, 37°C	18.5	U/L		
				Bicarbonate	24.4	mmol/L		
				Bilirubin, total	20.3	umol/L		
				Urea/BUN	3.34	mmol/L		
				Calcium	2.18	mmol/L	L	No
				Creatinine	60.4	umol/L		
				Glucose, serum	5.04	mmol/L		
				Gamma-GT, 37°C	8.1	U/L		
				Potassium	4.06	mmol/L		
				Creatinine Clearance MDRD	105	ml/min/1.73m		
				Magnesium	0.80	mmol/L		
				Sodium	136.9	mmol/L		
			Drugs	Amphetamines, Urine	negative			
				Barbiturates, Urine	negative			
				Benzodiazepines, Urine	negative			
				Cannabin., Urine	negative			
				Cocaine, Urine	negative			
				Methadone, Urine	negative			
				Opiates, Urine	negative			
			Haematology	Basophils, abs.	0.05	10^9/L		
				Basophils, %	0.8	%		
				Eosinophils, abs.	0.10	10^9/L		
				Eosinophils, %	1.6	%		
				Haemoglobin	121.0	g/L		
				Haematocrit	0.35	L/L		
				Lymphocytes, abs.	2.33	10^9/L		

Sign.: Significant finding; L: Result considered low; H: Result considered high; SC: Screening; BL: Baseline; D: Day; FUP: Follow-up;

Output generated by program 'NIC002_L16_2_8_Laboratory_V02_0_0'

Listing 16.2.8: Study subject data
Laboratory values

Part C

Cohort	Subject ID	Visit	Type	Measurement	Result		Flagged	Sign.
	66	SC	Haematology	Lymphocytes, %	37.5	%		
				Monocytes, abs.	0.46	10^9/L		
				Monocytes, %	7.4	%		
				Neutrophils, abs.	3.28	10^9/L		
				Neutrophils, %	52.7	%		
				Platelets	317	10^9/L		
				Erythrocytes	3.77	10^{12}/L	L	No
				Leucocytes	6.22	10^9/L		
			Haemostasis	APTT	26.3	s		
				Prothrombin Time (INR)	1.00	N/A		
				Prothrombin Time (PT)	101.5	%		
			Infectious Diseases	HBs-Ag (Hep. B Surf. Ag)	negative	N/A		
				Anti-HCV (Hep. C-AB)	non-reactive	N/A		
				HIV 1+2, AG/AB	negative	N/A		
			Urine	Bilirubin, urine (Stix)	negative			
				Blood (Ery/Hb), urine (Stix)	negative			
				Glucose, urine (Stix)	negative			
				Beta-HCG, urine	negative			
				Ketone, urine (Stix)	negative			
				Leucocytes, urine (Stix)	negative			
				Nitrite, urine (Stix)	negative			
				pH, urine (Stix)	5.5	neg.log[H+]		
				Protein, total, urine (Stix)	negative			
				Bacteria, Sediment	positive		H	No
				Carbonate, Sediment	negative			
				Epithelial Cells, Sediment	5	per field		
				Erythrocytes, Sediment	0	per field		
				Casts granul., Sediment	0	per field		
				Casts hyaline, Sediment	0	per field		
				Leucocytes, Sediment	2	per field		
				Oxalate, Sediment	negative			

Sign.: Significant finding; L: Result considered low; H: Result considered high; SC: Screening; BL: Baseline; D: Day; FUP: Follow-up;

Output generated by program 'NIC002_L16_2_8_Laboratory_V02_0_0'

Listing 16.2.8: Study subject data
Laboratory values

Part C

Cohort	Subject ID	Visit	Type	Measurement	Result		Flagged	Sign.
	66	SC	Urine	Specific Gravity	>=1.030		H	No
				Mucus, Sediment	positive		H	No
				Triple Phosphate, Sediment	negative			
				Urates, Sediment	negative			
				Urobilinogen, urine (Stix)	0.2	mg/dl		
		BL	Clinical Chemistry	ALT, 37°C	8.2	U/L		
				Alkaline Phosphatase, 37°C	49.9	U/L		
				AST, 37°C	17.9	U/L		
				Bicarbonate	24.2	mmol/L		
				Bilirubin, total	15.1	umol/L		
				Urea/BUN	4.66	mmol/L		
				Calcium	2.17	mmol/L	L	No
				Creatinine	61.2	umol/L		
				Glucose, serum	4.94	mmol/L		
				Gamma-GT, 37°C	7.2	U/L		
				Potassium	4.08	mmol/L		
				Magnesium	0.80	mmol/L		
				Sodium	135.6	mmol/L	L	No
			Drugs	Amphetamines, Urine	negative			
				Barbiturates, Urine	negative			
				Benzodiazepines, Urine	negative			
				Cannabin., Urine	negative			
				Cocaine, Urine	negative			
				Methadone, Urine	negative			
				Opiates, Urine	negative			
			Haematology	Basophils, abs.	0.05	10^9/L		
				Basophils, %	0.9	%		
				Eosinophils, abs.	0.24	10^9/L		
				Eosinophils, %	4.3	%		
				Haemoglobin	118.0	g/L		

Sign.: Significant finding; L: Result considered low; H: Result considered high; SC: Screening; BL: Baseline; D: Day; FUP: Follow-up;

Output generated by program 'NIC002_L16_2_8_Laboratory_V02_0_0'

Listing 16.2.8: Study subject data
Laboratory values

Part C

Cohort	Subject ID	Visit	Type	Measurement	Result		Flagged	Sign.
		BL	Haematology	Haematocrit	0.35	L/L		
				Lymphocytes, abs.	2.71	10^9/L		
				Lymphocytes, %	48.0	%		
				Monocytes, abs.	0.50	10^9/L		
				Monocytes, %	8.9	%		
				Neutrophils, abs.	2.14	10^9/L		
				Neutrophils, %	37.9	%		
				Platelets	315	10^9/L		
				Erythrocytes	3.74	10^{12}/L	L	No
				Leucocytes	5.64	10^9/L		
			Haemostasis	APTT	26.5	s		
				Prothrombin Time (INR)	0.98	N/A		
				Prothrombin Time (PT)	111.0	%		
			Urine	Beta-HCG, urine	negative			
		D03	Clinical Chemistry	ALT, 37°C	8.6	U/L		
				Alkaline Phosphatase, 37°C	48.4	U/L		
				AST, 37°C	15.3	U/L		
				Bicarbonate	23.8	mmol/L		
				Bilirubin, total	15.6	umol/L		
				Urea/BUN	5.34	mmol/L		
				Calcium	2.13	mmol/L	L	No
				Creatinine	59.1	umol/L		
				Glucose, serum	3.87	mmol/L	L	No
				Gamma-GT, 37°C	7.3	U/L		
				Potassium	4.19	mmol/L		
				Magnesium	0.77	mmol/L		
				Sodium	135.7	mmol/L	L	No
			Haematology	Basophils, abs.	0.04	10^9/L		
				Basophils, %	0.7	%		
				Eosinophils, abs.	0.18	10^9/L		

Sign.: Significant finding; L: Result considered low; H: Result considered high; SC: Screening; BL: Baseline; D: Day; FUP: Follow-up;

Output generated by program 'NIC002_L16_2_8_Laboratory_V02_0_0'

Listing 16.2.8: Study subject data
Laboratory values

Part C

Cohort	Subject ID	Visit	Type	Measurement	Result		Flagged	Sign.
		D03	Haematology	Eosinophils, %	3.1	%		
				Haemoglobin	121.0	g/L		
				Haematocrit	0.36	L/L		
				Lymphocytes, abs.	2.67	10^9/L		
				Lymphocytes, %	46.5	%		
				Monocytes, abs.	0.44	10^9/L		
				Monocytes, %	7.7	%		
				Neutrophils, abs.	2.41	10^9/L		
				Neutrophils, %	42.0	%		
				Platelets	290	10^9/L		
				Erythrocytes	3.77	10^{12}/L	L	No
				Leucocytes	5.74	10^9/L		
			Haemostasis	APTT	27.1	s		
				Prothrombin Time (INR)	0.97	N/A		
				Prothrombin Time (PT)	111.0	%		
		D08	Clinical Chemistry	ALT, 37°C	13.4	U/L		
				Alkaline Phosphatase, 37°C	47.7	U/L		
				AST, 37°C	20.1	U/L		
				Bicarbonate	23.3	mmol/L		
				Bilirubin, total	16.7	umol/L		
				Urea/BUN	4.39	mmol/L		
				Calcium	2.20	mmol/L		
				Creatinine	58.8	umol/L		
				Glucose, serum	4.35	mmol/L		
				Gamma-GT, 37°C	7.8	U/L		
				Potassium	4.14	mmol/L		
				Magnesium	0.79	mmol/L		
				Sodium	135.9	mmol/L	L	No
			Haematology	Basophils, abs.	0.05	10^9/L		
				Basophils, %	0.8	%		

Sign.: Significant finding; L: Result considered low; H: Result considered high; SC: Screening; BL: Baseline; D: Day; FUP: Follow-up;

Output generated by program 'NIC002_L16_2_8_Laboratory_V02_0_0'

Listing 16.2.8: Study subject data
Laboratory values

Part C

Cohort	Subject ID	Visit	Type	Measurement	Result		Flagged	Sign.
		D08	Haematology	Eosinophils, abs.	0.13	10^9/L		
				Eosinophils, %	2.0	%		
				Haemoglobin	118.0	g/L		
				Haematocrit	0.35	L/L		
				Lymphocytes, abs.	2.28	10^9/L		
				Lymphocytes, %	34.9	%		
				Monocytes, abs.	0.44	10^9/L		
				Monocytes, %	6.7	%		
				Neutrophils, abs.	3.63	10^9/L		
				Neutrophils, %	55.6	%		
				Platelets	327	10^9/L		
				Erythrocytes	3.72	10^{12}/L	L	No
				Leucocytes	6.53	10^9/L		
			Haemostasis	APTT	27.2	s		
				Prothrombin Time (INR)	0.96	N/A		
				Prothrombin Time (PT)	114.1	%		
		FUP	Clinical Chemistry	ALT, 37°C	15.2	U/L		
				Alkaline Phosphatase, 37°C	49.0	U/L		
				AST, 37°C	19.5	U/L		
				Bicarbonate	24.2	mmol/L		
				Bilirubin, total	15.7	umol/L		
				Urea/BUN	3.98	mmol/L		
				Calcium	2.16	mmol/L	L	No
				Creatinine	61.7	umol/L		
				Glucose, serum	4.68	mmol/L		
				Gamma-GT, 37°C	7.9	U/L		
				Potassium	4.66	mmol/L		
				Magnesium	0.78	mmol/L		
				Sodium	134.5	mmol/L	L	No
			Haematology	Basophils, abs.	0.05	10^9/L		

Sign.: Significant finding; L: Result considered low; H: Result considered high; SC: Screening; BL: Baseline; D: Day; FUP: Follow-up;

Output generated by program 'NIC002_L16_2_8_Laboratory_V02_0_0'

Listing 16.2.8: Study subject data
Laboratory values

Part C

Cohort	Subject ID	Visit	Type	Measurement	Result		Flagged	Sign.
		FUP	Haematology	Basophils, %	0.9	%		
				Eosinophils, abs.	0.19	10^9/L		
				Eosinophils, %	3.5	%		
				Haemoglobin	109.0	g/L	L	No
				Haematocrit	0.33	L/L	L	No
				Lymphocytes, abs.	2.78	10^9/L		
				Lymphocytes, %	50.7	%	H	No
				Monocytes, abs.	0.45	10^9/L		
				Monocytes, %	8.2	%		
				Neutrophils, abs.	2.01	10^9/L		
				Neutrophils, %	36.7	%	L	No
				Platelets	341	10^9/L		
				Erythrocytes	3.45	10^{12}/L	L	No
				Leucocytes	5.48	10^9/L		
			Haemostasis	APTT	26.0	s		
				Prothrombin Time (INR)	0.94	N/A		
				Prothrombin Time (PT)	120.6	%		

Sign.: Significant finding; L: Result considered low; H: Result considered high; SC: Screening; BL: Baseline; D: Day; FUP: Follow-up;

Output generated by program 'NIC002_L16_2_8_Laboratory_V02_0_0'

Listing 16.2.9: Study subject data
Vital signs

Part A

Cohort	Subject ID	Visit	Done	TP	Heart rate [beats/min]	Bloodpressure [mmHg] Systolic	Bloodpressure [mmHg] Diastolic	Respiration [breaths/min]	Body tempe-rature [°C]
Cohort A1	2	SC	Yes		68	90	54	16	36.7
		BL	Yes		71	87	49	13	36.6
		D01 Fast	Yes	Pre-dose	69	90	50	14	36.8
				1 h	64	91	55	13	
				2 h	65	90	50	14	
				3 h					36.8
				4 h	69	95	54	14	
				8 h	68	91	51	13	
				12 h	75	93	50	18	
		FUP	Yes		78	99	51	14	35.3
	5	SC	Yes		84	107	63	12	37.1
		BL	Yes		84	113	70	17	37.1
		D01 Fast	Yes	Pre-dose	86	112	67	14	36.7
				1 h	80	116	71	13	
				2 h	71	119	71	14	
				3 h					36.7
				4 h	77	108	75	13	
				8 h	58	105	59	14	
				12 h	82	101	60	13	
		FUP	Yes		94	112	62	12	36.5
	7	SC	Yes		77	110	63	12	36.6
		BL	Yes		77	112	65	13	36.9
		D01 Fast	Yes	Pre-dose	65	110	62	13	36.8
				1 h	58	102	58	14	
				2 h	60	103	62	13	
				3 h					36.9
				4 h	54	102	57	13	

TP: Timepoint of measurement; Cohort A3: Treatment was applied under fasting and fed conditions in the same subjects;

Output generated by program 'NIC002_L16_2_9_VitalSigns_V02_0_0'

Listing 16.2.9: Study subject data
Vital signs

Part A

Cohort	Subject ID	Visit	Done	TP	Heart rate [beats/min]	Bloodpressure [mmHg] Systolic	Bloodpressure [mmHg] Diastolic	Respiration [breaths/min]	Body temperature [°C]
	7	D01 Fast	Yes	8 h	62	103	60	13	
				12 h	72	113	65	14	
		FUP	Yes		70	113	62	12	35.4
	8	SC	Yes		73	106	61	14	36.6
		BL	Yes		77	109	61	13	36.7
		D01 Fast	Yes	Pre-dose	68	102	65	12	37.2
				1 h	80	114	71	13	
				2 h	68	96	60	13	
				3 h					37
				4 h	62	109	63	12	
				8 h	69	101	63	14	
				12 h	70	110	65	13	
		FUP	Yes		72	101	57	12	35.6
Cohort A2	13	SC	Yes		72	98	59	12	37.1
		BL	Yes		68	99	56	15	36.9
		D01 Fast	Yes	Pre-dose	67	100	56	10	37
				1 h	61	101	65	11	
				2 h	65	101	61	11	
				3 h					36.9
				4 h	69	101	61	13	
				8 h	69	97	57	13	
				12 h	68	93	52	15	
		FUP	Yes		73	101	58	12	36.9
	19	SC	Yes		62	130	80	12	36.9
		BL	Yes		76	117	69	12	37.2
		D01 Fast	Yes	Pre-dose	52	112	62	14	37.1
				1 h	56	110	63	15	

TP: Timepoint of measurement; Cohort A3: Treatment was applied under fasting and fed conditions in the same subjects;

Output generated by program 'NIC002_L16_2_9_VitalSigns_V02_0_0'

Listing 16.2.9: Study subject data
Vital signs

Part A

Cohort	Subject ID	Visit	Done	TP	Heart rate [beats/min]	Bloodpressure [mmHg] Systolic	Bloodpressure [mmHg] Diastolic	Respiration [breaths/min]	Body temperature [°C]
	19	D01 Fast	Yes	2 h	58	109	63	13	
				3 h					37.1
				4 h	58	109	58	10	
				8 h	63	107	59	11	
				12 h	66	108	55	14	
		FUP	Yes		80	112	69	13	36.8
	21	SC	Yes		63	103	64	16	36.6
		BL	Yes		71	131	80	13	36.6
		D01 Fast	Yes	Pre-dose	71	106	64	13	37
				1 h	68	108	70	15	
				2 h	62	106	66	14	
				3 h					36.9
				4 h	68	103	70	13	
				8 h	71	111	67	12	
				12 h	72	111	69	14	
		FUP	Yes		73	104	68	14	35.3
	22	SC	Yes		59	108	69	16	36.4
		BL	Yes		65	108	68	12	36.8
		D01 Fast	Yes	Pre-dose	62	109	68	15	37
				1 h	66	103	67	13	
				2 h	70	109	56	15	
				3 h					37.4
				4 h	64	107	61	12	
				8 h	68	105	68	11	
				12 h	70	104	59	13	
		FUP	Yes		69	110	66	14	37
Cohort A3 Fast	24	SC	Yes		62	115	66	12	36.5

TP: Timepoint of measurement; Cohort A3: Treatment was applied under fasting and fed conditions in the same subjects;

Output generated by program 'NIC002_L16_2_9_VitalSigns_V02_0_0'

Listing 16.2.9: Study subject data
Vital signs

Part A

Cohort	Subject ID	Visit	Done	TP	Heart rate [beats/min]	Bloodpressure [mmHg] Systolic	Bloodpressure [mmHg] Diastolic	Respiration [breaths/min]	Body temperature [°C]
Cohort A3 Fast	24	BL	Yes		76	112	63	16	36.8
		D01 Fast	Yes	Pre-dose	60	107	60	15	37
				1 h	79	102	61	16	
				2 h	75	105	57	16	
				3 h					37.1
				4 h	75	129	70	16	
				8 h	70	98	57	15	
				12 h	70	136	76	15	
		FUP Fast	Yes		77	122	80	12	36.6
	25	SC	Yes		72	114	55	15	37.1
		BL	Yes		57	113	58	15	36.4
		D01 Fast	Yes	Pre-dose	51	107	56	17	37
				1 h	52	105	63	16	
				2 h	53	103	58	16	
				3 h					36.3
				4 h	53	110	58	20	
				8 h	62	106	55	15	
				12 h	62	110	57	14	
		FUP Fast	Yes		77	108	58	13	36.6
	27	SC	Yes		64	114	70	12	36.8
		BL	Yes		65	121	71	16	36
		D01 Fast	Yes	Pre-dose	56	125	78	16	36.5
				1 h	55	113	67	16	
				2 h	51	118	65	16	
				3 h					36.9
				4 h	52	113	66	15	
				8 h	58	117	72	12	
				12 h	57	117	69	14	

TP: Timepoint of measurement; Cohort A3: Treatment was applied under fasting and fed conditions in the same subjects;

Output generated by program 'NIC002_L16_2_9_VitalSigns_V02_0_0'

Listing 16.2.9: Study subject data
Vital signs

Part A

Cohort	Subject ID	Visit	Done	TP	Heart rate [beats/min]	Bloodpressure [mmHg] Systolic	Bloodpressure [mmHg] Diastolic	Respiration [breaths/min]	Body temperature [°C]
	27	FUP Fast	Yes		67	117	66	12	36.4
	28	SC	Yes		60	103	59	12	36.7
		Unscheduled	No	Pre-dose					
				1 h					
				2 h					
				4 h					
				8 h					
				12 h					
			Yes	3 h					37.2
		BL	Yes		60	101	57	16	36.8
		D01 Fast	Yes	Pre-dose	64	125	71	17	36.5
				1 h	71	112	64	16	
				2 h	69	106	57	16	
				3 h					37.6
				4 h	67	100	59	15	
				8 h	64	98	54	16	
				12 h	70	105	58	15	
		FUP Fast	Yes		53	107	58	20	36.7
Cohort A3 Fed	24	BL Fed	Yes		65	120	71	14	36.9
		D01 Fed	Yes	Pre-dose	64	116	69	18	37.1
				1 h	77	109	67	16	
				2 h	79	105	64	16	
				3 h					37.4
				4 h	69	105	60	16	
				8 h	81	121	62	12	
				12 h	67	125	74	13	
		FUP	Yes		68	120	71	14	36.8

TP: Timepoint of measurement; Cohort A3: Treatment was applied under fasting and fed conditions in the same subjects;

Output generated by program 'NIC002_L16_2_9_VitalSigns_V02_0_0'

Listing 16.2.9: Study subject data
Vital signs

Part A

Cohort	Subject ID	Visit	Done	TP	Heart rate [beats/min]	Bloodpressure [mmHg] Systolic	Bloodpressure [mmHg] Diastolic	Respiration [breaths/min]	Body temperature [°C]
	25	BL Fed	Yes		62	111	56	14	36.1
		D01 Fed	Yes	Pre-dose	59	113	55	15	36.8
				1 h	68	107	60	16	
				2 h	74	115	55	15	
				3 h					36.9
				4 h	72	112	58	14	
				8 h	65	110	55	13	
				12 h	64	109	53	12	
		FUP	Yes		57	105	56	12	35.6
	27	BL Fed	Yes		67	120	63	15	35
		D01 Fed	Yes	Pre-dose	62	120	74	16	36.6
				1 h	61	122	68	15	
				2 h	60	116	59	16	
				3 h					36.3
				4 h	50	111	70	13	
				8 h	56	117	72	14	
				12 h	65	119	71	15	
		FUP	Yes		60	131	58	13	36.5
	28	BL Fed	Yes		63	112	57	14	35.7
		D01 Fed	Yes	Pre-dose	56	109	58	17	36.9
				1 h	70	99	52	13	
				2 h	73	101	59	15	
				3 h					37.3
				4 h	65	112	56	14	
				8 h	67	110	63	13	
				12 h	66	95	55	12	
		FUP	Yes		79	109	56	14	36.3

TP: Timepoint of measurement; Cohort A3: Treatment was applied under fasting and fed conditions in the same subjects;

Output generated by program 'NIC002_L16_2_9_VitalSigns_V02_0_0'

Listing 16.2.9: Study subject data
Vital signs

Part B

Treatment	Subject ID	Visit	Done	TP	Heart rate [beats/min]	Bloodpressure [mmHg] Systolic	Bloodpressure [mmHg] Diastolic	Respiration [breaths/min]	Body temperature [°C]
Chewing tablet 2000 mg	48	D03	Yes	Pre-dose	51	109	63	16	36.8
				1 h	57	98	58	18	
				2 h	53	97	52	15	
				3 h					36.9
				4 h	50	103	66	13	
				8 h	50	96	59	14	
				12 h	56	104	56	14	
		FUP	Yes		62	110	65	13	36.1
	49	SC	Yes		79	127	79	14	36.9
		BL	Yes		92	134	78	12	36.5
		D01 Fast	Yes	Pre-dose	76	126	81	16	37.5
				1 h	77	129	72	15	
				2 h	79	125	62	14	
				3 h					37.4
				4 h	71	114	69	14	
				8 h	68	122	73	13	
				12 h	86	118	57	14	
	51	D03	Yes	Pre-dose	76	114	70	15	37.2
				1 h	79	103	60	12	
				2 h	72	108	65	12	
				3 h					37.2
				4 h	85	126	77	14	
				8 h	94	100	69	14	
				12 h	78	107	67	15	
		FUP	Yes		84	108	69	12	36
	76	SC	Yes		63	107	59	15	36.9

TP: Timepoint of measurement; Part B used a cross-over design;

Output generated by program 'NIC002_L16_2_9_VitalSigns_V02_0_0'

Listing 16.2.9: Study subject data
Vital signs

Part B

Treatment	Subject ID	Visit	Done	TP	Heart rate [beats/min]	Bloodpressure [mmHg] Systolic	Bloodpressure [mmHg] Diastolic	Respiration [breaths/min]	Body temperature [°C]
	76	BL	Yes		68	113	63	12	36.3
		D01 Fast	Yes	Pre-dose	61	107	56	13	36.7
				1 h	64	108	58	14	
				2 h	63	109	55	14	
				3 h					36.7
				4 h	65	118	61	14	
				8 h	70	113	66	14	
				12 h	63	123	59	13	
Solution 1600 mg	48	SC	Yes		59	121	74	14	36.5
		BL	Yes		58	104	64	14	36
		D01 Fast	Yes	Pre-dose	51	104	56	14	36.8
				1 h	55	104	65	13	
				2 h	54	107	65	14	
				3 h					36.8
				4 h	48	103	53	13	
				8 h	55	102	65	14	
				12 h	60	96	52	15	
	49	D03	Yes	Pre-dose	73	115	69	15	37.3
				1 h	84	123	78	14	
				2 h	77	117	64	11	
				3 h					37.2
				4 h	72	110	68	13	
				8 h	75	115	65	13	
				12 h	75	120	75	14	
		FUP	Yes		88	127	73	16	35.9
	51	SC	Yes		87	111	78	13	36.6
		BL	Yes		98	120	78	18	36.4

TP: Timepoint of measurement; Part B used a cross-over design;

Output generated by program 'NIC002_L16_2_9_VitalSigns_V02_0_0'

Listing 16.2.9: Study subject data
Vital signs

Part B

Treatment	Subject ID	Visit	Done	TP	Heart rate [beats/min]	Bloodpressure [mmHg] Systolic	Bloodpressure [mmHg] Diastolic	Respiration [breaths/min]	Body temperature [°C]
	51	D01 Fast	Yes	Pre-dose	81	111	72	13	37.5
				1 h	87	106	68	15	
				2 h	86	112	70	13	
				3 h					37.3
				4 h	79	110	69	14	
				8 h	80	113	74	13	
				12 h	84	122	72	15	
	76	D03	Yes	Pre-dose	73	105	52	13	36.7
				1 h	65	106	56	13	
				2 h	62	92	54	13	
				3 h					37.5
				4 h	61	95	51	14	
				8 h	64	100	49	13	
				12 h	58	108	55	13	
		FUP	Yes		80	105	57	11	35.8

TP: Timepoint of measurement; Part B used a cross-over design;

Output generated by program 'NIC002_L16_2_9_VitalSigns_V02_0_0'

Listing 16.2.9: Study subject data
Vital signs

Part C

Group	Subject ID	Visit	Done	TP	Heart rate [beats/min]	Bloodpressure [mmHg] Systolic	Bloodpressure [mmHg] Diastolic	Respiration [breaths/min]	Body temperature [°C]
Group 1	54	SC	Yes		82	116	62	16	36.8
		BL	Yes		81	136	58	14	36.2
		D01 Fast	Yes	Pre-dose	83	112	57	13	37.3
				1 h	79	109	59	15	37.3
				2 h	81	111	57	14	37.3
				4 h	80	108	61	12	37.4
				8 h	78	113	62	13	37.1
				12 h	73	106	65	13	36.9
		D03	Yes		72	109	63	15	37.3
		D08	Yes		64	108	61	16	
		FUP	Yes		65	108	54	13	35.6
	63	SC	Yes		74	119	69	13	36.4
		BL	Yes		71	130	80	14	36.4
		D01 Fast	Yes	Pre-dose	58	125	79	14	36.4
				1 h	81	116	74	14	36.4
				2 h	89	114	74	13	36.7
				4 h	80	113	75	14	36.4
				8 h	70	107	65	15	36.9
				12 h	69	113	72	15	36.9
		D03	Yes		76	117	73	14	36.4
		D08	Yes		71	108	67	15	
		FUP	Yes		78	120	65	15	36.1
	67	SC	Yes		67	106	66	12	36.7
		BL	Yes		66	110	57	11	36.8
		D01 Fast	Yes	Pre-dose	69	109	51	14	36.5
				1 h	70	104	57	13	36.6
				2 h	61	99	52	12	37.5

TP: Timepoint of measurement;

Output generated by program 'NIC002_L16_2_9_VitalSigns_V02_0_0'

Listing 16.2.9: Study subject data
Vital signs

Part C

Group	Subject ID	Visit	Done	TP	Heart rate [beats/min]	Bloodpressure [mmHg] Systolic	Bloodpressure [mmHg] Diastolic	Respiration [breaths/min]	Body temperature [°C]
	67	D01 Fast	Yes	4 h	52	94	52	15	37.1
				8 h	62	90	54	13	37.3
				12 h	67	103	56	12	37.3
		D03	Yes		74	100	62	14	37.2
		D08	Yes		61	96	54	12	
		FUP	Yes		60	103	78	14	35.7
	71	SC	Yes		84	120	72	14	37.1
		BL	Yes		80	119	74	12	37.4
		D01 Fast	Yes	Pre-dose	76	110	65	13	36.7
				1 h	81	113	63	14	37.1
				2 h	77	118	64	12	37.3
				4 h	83	108	63	14	37.2
				8 h	76	115	68	14	37.1
				12 h	84	115	72	15	37.5
		D03	Yes		72	108	62	14	37
		D08	Yes		80	111	62	11	
		FUP	Yes		83	120	71	17	36.3
Group 2	57	SC	Yes		75	120	76	14	36.4
		BL	Yes		61	116	77	13	36.7
		D01 Fast	Yes	Pre-dose	82	103	67	14	36.4
				1 h	88	117	68	14	36.4
				2 h	75	107	69	15	36.4
				4 h	82	117	60	14	36.6
				8 h	79	112	64	16	37
				12 h	75	112	72	18	37.3
		D03	Yes		73	118	75	14	37
		D08	Yes		71	116	76	16	
		FUP	Yes		78	123	75	14	36.1

TP: Timepoint of measurement;

Output generated by program 'NIC002_L16_2_9_VitalSigns_V02_0_0'

Listing 16.2.9: Study subject data
Vital signs

Part C

Group	Subject ID	Visit	Done	TP	Heart rate [beats/min]	Bloodpressure [mmHg] Systolic	Bloodpressure [mmHg] Diastolic	Respiration [breaths/min]	Body temperature [°C]
	58	SC	Yes		80	107	69	13	36
		BL	Yes		80	109	65	14	36.4
		D01 Fast	Yes	Pre-dose	84	117	74	14	36.8
				1 h	91	104	66	14	36.6
				2 h	95	100	61	14	36.4
				4 h	80	109	62	13	37
				8 h	77	102	63	15	36.8
				12 h	82	101	65	13	36.8
		D03	Yes		81	110	66	14	36.4
		D08	Yes		70	104	64	16	
		FUP	Yes		73	113	63	18	35.6
	68	SC	Yes		63	112	71	12	36.9
		BL	Yes		63	121	77	14	36.7
		D01 Fast	Yes	Pre-dose	51	107	63	13	37.2
				1 h	84	119	66	14	37.2
				2 h	87	118	62	14	37.5
				4 h	68	114	68	13	37.2
				8 h	67	109	66	14	37.1
				12 h	67	115	71	15	36.9
		D03	Yes		71	108	60	16	37
		D08	Yes		68	115	65	16	
		FUP	Yes		73	110	61	17	36.5
	69	SC	Yes		54	106	65	12	37.1
		BL	Yes		54	109	63	12	36.8
		D01 Fast	Yes	Pre-dose	58	106	60	14	37.1
				1 h	60	114	69	13	37.3
				2 h	57	117	64	13	37

TP: Timepoint of measurement;

Output generated by program 'NIC002_L16_2_9_VitalSigns_V02_0_0'

Listing 16.2.9: Study subject data
Vital signs

Part C

Group	Subject ID	Visit	Done	TP	Heart rate [beats/min]	Bloodpressure [mmHg] Systolic	Bloodpressure [mmHg] Diastolic	Respiration [breaths/min]	Body temperature [°C]
	69	D01 Fast	Yes	4 h	58	111	55	13	37.3
				8 h	55	100	63	14	37.4
				12 h	55	105	61	14	37.2
		D03	Yes		60	110	64	14	37.2
		D08	Yes		58	113	60	18	
		FUP	Yes		65	112	61	18	36.3
Group 3	55	SC	Yes		69	109	66	13	36.3
		BL	Yes		52	101	65	13	36.1
		D01 Fast	Yes	Pre-dose	60	109	68	12	37.3
				1 h	70	110	62	13	37.1
				2 h	66	102	57	12	37.1
				4 h	71	104	63	15	37.1
				8 h	60	102	59	13	37.4
				12 h	66	108	66	12	37.2
		D03	Yes		51	101	58	14	37
		D08	Yes		69	113	64	17	
		FUP	Yes		71	108	61	14	35.5
	62	SC	Yes		55	101	61	13	36.1
		BL	Yes		53	108	64	12	36.4
		D01 Fast	Yes	Pre-dose	55	111	64	11	36.8
				1 h	61	92	54	10	36.8
				2 h	60	92	55	11	36.7
				4 h	59	102	62	12	36.9
				8 h	61	93	48	15	36.6
				12 h	58	97	52	18	36
		D03	Yes		54	98	57	14	36.8
		D08	Yes		58	101	60	16	
		FUP	Yes		53	109	61	12	35.2

TP: Timepoint of measurement;

Output generated by program 'NIC002_L16_2_9_VitalSigns_V02_0_0'

Listing 16.2.9: Study subject data
Vital signs

Part C

Group	Subject ID	Visit	Done	TP	Heart rate [beats/min]	Bloodpressure [mmHg] Systolic	Bloodpressure [mmHg] Diastolic	Respiration [breaths/min]	Body temperature [°C]
	65	SC	Yes		76	105	66	12	36.9
		BL	Yes		80	111	63	15	36
		D01 Fast	Yes	Pre-dose	68	116	67	13	36.7
				1 h	76	119	67	14	36.7
				2 h	73	102	57	13	36.8
				4 h	67	119	77	15	36.5
				8 h	65	110	68	12	36.9
				12 h	77	110	58	13	36.8
		D03	Yes		72	106	63	12	36.4
		D08	Yes		68	102	58	14	
		FUP	Yes		73	105	64	15	35.3
	66	SC	Yes		75	107	61	14	37.1
		BL	Yes		76	108	65	13	36.7
		D01 Fast	Yes	Pre-dose	72	108	59	12	36.6
				1 h	78	114	58	13	37
				2 h	72	106	56	14	36.9
				4 h	71	113	65	14	36.2
				8 h	73	109	66	14	37
				12 h	80	106	59	15	36.5
		D03	Yes		70	104	63	12	36.4
		D08	Yes		75	104	61	16	
		FUP	Yes		82	113	63	16	36.5

TP: Timepoint of measurement;

Output generated by program 'NIC002_L16_2_9_VitalSigns_V02_0_0'

Listing 16.2.10: Study subject data
Other data

Part A

Cohort	Subject ID	Visit	COVID-19 test		Pregnancy test		
			Date	Result	Date	Test type	Result
Cohort A1	2	SC	14NOV2020	Negative	05NOV2020	Urine/β-HCG	Negative
		BL			16NOV2020	Urine/β-HCG	Negative
		FUP			20NOV2020	Urine/β-HCG	Negative
	5	SC	16NOV2020	Negative	05NOV2020	Urine/β-HCG	Negative
		BL			18NOV2020	Urine/β-HCG	Negative
		FUP			22NOV2020	Urine/β-HCG	Negative
	7	SC	16NOV2020	Negative	05NOV2020	Urine/β-HCG	Negative
		BL			18NOV2020	Urine/β-HCG	Negative
		FUP			22NOV2020	Urine/β-HCG	Negative
	8	SC	16NOV2020	Negative	06NOV2020	Urine/β-HCG	Negative
		BL			18NOV2020	Urine/β-HCG	Negative
		FUP			22NOV2020	Urine/β-HCG	Negative
Cohort A2	13	SC	28NOV2020	Negative	11NOV2020	Urine/β-HCG	Negative
		BL			30NOV2020	Urine/β-HCG	Negative
		FUP			04DEC2020	Urine/β-HCG	Negative

SC: Screening; BL: Baseline; FUP: Follow-up; Fast: Under fasting conditions;

Output generated by program 'NIC002_L16_2_10_OtherData_V01_0_0'

Listing 16.2.10: Study subject data
Other data

Part A

Cohort	Subject ID	Visit	COVID-19 test		Pregnancy test		
			Date	Result	Date	Test type	Result
	19	SC	30NOV2020	Negative	18NOV2020	Urine/β-HCG	Negative
		BL			02DEC2020	Urine/β-HCG	Negative
		FUP			08DEC2020	Urine/β-HCG	Negative
	21	SC	30NOV2020	Negative	23NOV2020	Urine/β-HCG	Negative
		BL			02DEC2020	Urine/β-HCG	Negative
		FUP			06DEC2020	Urine/β-HCG	Negative
	22	SC	30NOV2020	Negative	25NOV2020	Urine/β-HCG	Negative
		BL			02DEC2020	Urine/β-HCG	Negative
		FUP			06DEC2020	Urine/β-HCG	Negative
Cohort A3	24	SC	03JAN2021	Negative	21DEC2020	Urine/β-HCG	Negative
		BL			05JAN2021	Urine/β-HCG	Negative
		FUP Fast			09JAN2021	Urine/β-HCG	Negative
		BL Fed			19JAN2021	Urine/β-HCG	Negative
		FUP			25JAN2021	Urine/β-HCG	Negative

SC: Screening; BL: Baseline; FUP: Follow-up; Fast: Under fasting conditions;

Output generated by program 'NIC002_L16_2_10_OtherData_V01_0_0'

Listing 16.2.10: Study subject data
Other data

Part A

Cohort	Subject ID	Visit	COVID-19 test		Pregnancy test		
			Date	Result	Date	Test type	Result
	25	SC	05JAN2021	Negative	21DEC2020	Urine/β-HCG	Negative
		BL			07JAN2021	Urine/β-HCG	Negative
		FUP Fast			11JAN2021	Urine/β-HCG	Negative
		BL Fed			21JAN2021	Urine/β-HCG	Negative
		FUP			25JAN2021	Urine/β-HCG	Negative
	27	SC	05JAN2021	Negative	21DEC2020	Urine/β-HCG	Negative
		BL			07JAN2021	Urine/β-HCG	Negative
		FUP Fast			11JAN2021	Urine/β-HCG	Negative
		BL Fed			21JAN2021	Urine/β-HCG	Negative
		FUP			25JAN2021	Urine/β-HCG	Negative
	28	SC	05JAN2021	Negative	21DEC2020	Urine/β-HCG	Negative
		BL			07JAN2021	Urine/β-HCG	Negative

SC: Screening; BL: Baseline; FUP: Follow-up; Fast: Under fasting conditions;

Output generated by program 'NIC002_L16_2_10_OtherData_V01_0_0'

Listing 16.2.10: Study subject data
Other data

Part A

Cohort	Subject ID	Visit	COVID-19 test		Pregnancy test		
			Date	Result	Date	Test type	Result
		FUP Fast			11JAN2021	Urine/β-HCG	Negative
		BL Fed			21JAN2021	Urine/β-HCG	Negative
		FUP			25JAN2021	Urine/β-HCG	Negative

SC: Screening; BL: Baseline; FUP: Follow-up; Fast: Under fasting conditions;

Output generated by program 'NIC002_L16_2_10_OtherData_V01_0_0'

Listing 16.2.10: Study subject data
Other data

Part B

Sequence	Subject ID	Visit	COVID-19 test		Pregnancy test		
			Date	Result	Date	Test type	Result
Sequence 1	48	SC	26MAR2021	Negative	23MAR2021	Urine/β-HCG	Negative
		BL			28MAR2021	Urine/β-HCG	Negative
		FUP			03APR2021	Urine/β-HCG	Negative
	51	SC	26MAR2021	Negative	23MAR2021	Urine/β-HCG	Negative
		BL			28MAR2021	Urine/β-HCG	Negative
		FUP			03APR2021	Urine/β-HCG	Negative
Sequence 2	49	SC	26MAR2021	Negative	23MAR2021	Urine/β-HCG	Negative
		BL			28MAR2021	Urine/β-HCG	Negative
		FUP			03APR2021	Urine/β-HCG	Negative
	76	SC	25APR2021	Negative	15APR2021	Urine/β-HCG	Negative
		BL			27APR2021	Urine/β-HCG	Negative
		FUP			03MAY2021	Urine/β-HCG	Negative

SC: Screening; BL: Baseline; FUP: Follow-up; Fast: Under fasting conditions;

Output generated by program 'NIC002_L16_2_10_OtherData_V01_0_0'

Listing 16.2.10: Study subject data
Other data

Part C

Group	Subject ID	Visit	COVID-19 test		Pregnancy test		
			Date	Result	Date	Test type	Result
Group 1	54	SC	10APR2021	Negative	01APR2021	Urine/β-HCG	Negative
		BL			12APR2021	Urine/β-HCG	Negative
		FUP			22APR2021	Urine/β-HCG	Negative
	63	SC	10APR2021	Negative	06APR2021	Urine/β-HCG	Negative
		BL			12APR2021	Urine/β-HCG	Negative
		FUP			22APR2021	Urine/β-HCG	Negative
	67	SC	20APR2021	Negative	08APR2021	Urine/β-HCG	Negative
		BL			22APR2021	Urine/β-HCG	Negative
		FUP			03MAY2021	Urine/β-HCG	Negative
	71	SC	20APR2021	Negative	14APR2021	Urine/β-HCG	Negative
		BL			22APR2021	Urine/β-HCG	Negative
		FUP			03MAY2021	Urine/β-HCG	Negative
Group 2	57	SC	10APR2021	Negative	01APR2021	Urine/β-HCG	Negative
		BL			12APR2021	Urine/β-HCG	Negative
		FUP			22APR2021	Urine/β-HCG	Negative

SC: Screening; BL: Baseline; FUP: Follow-up; Fast: Under fasting conditions;

Output generated by program 'NIC002_L16_2_10_OtherData_V01_0_0'

Listing 16.2.10: Study subject data
Other data

Part C

Group	Subject ID	Visit	COVID-19 test		Pregnancy test		
			Date	Result	Date	Test type	Result
	58	SC	10APR2021	Negative	01APR2021	Urine/β-HCG	Negative
		BL			12APR2021	Urine/β-HCG	Negative
		FUP			22APR2021	Urine/β-HCG	Negative
	68	SC	20APR2021	Negative	14APR2021	Urine/β-HCG	Negative
		BL			22APR2021	Urine/β-HCG	Negative
		FUP			03MAY2021	Urine/β-HCG	Negative
	69	SC	20APR2021	Negative	14APR2021	Urine/β-HCG	Negative
		BL			22APR2021	Urine/β-HCG	Negative
		FUP			03MAY2021	Urine/β-HCG	Negative
Group 3	55	SC	10APR2021	Negative	01APR2021	Urine/β-HCG	Negative
		BL			12APR2021	Urine/β-HCG	Negative
		FUP			22APR2021	Urine/β-HCG	Negative
	62	SC	10APR2021	Negative	06APR2021	Urine/β-HCG	Negative
		BL			12APR2021	Urine/β-HCG	Negative
		FUP			22APR2021	Urine/β-HCG	Negative

SC: Screening; BL: Baseline; FUP: Follow-up; Fast: Under fasting conditions;

Output generated by program 'NIC002_L16_2_10_OtherData_V01_0_0'

Listing 16.2.10: Study subject data
Other data

Part C

Group	Subject ID	Visit	COVID-19 test		Pregnancy test		
			Date	Result	Date	Test type	Result
	65	SC	20APR2021	Negative	08APR2021	Urine/β-HCG	Negative
		BL			22APR2021	Urine/β-HCG	Negative
		FUP			03MAY2021	Urine/β-HCG	Negative
	66	SC	20APR2021	Negative	08APR2021	Urine/β-HCG	Negative
		BL			22APR2021	Urine/β-HCG	Negative
		FUP			03MAY2021	Urine/β-HCG	Negative

SC: Screening; BL: Baseline; FUP: Follow-up; Fast: Under fasting conditions;

Output generated by program 'NIC002_L16_2_10_OtherData_V01_0_0'
