# Supplementary material for: Clinical safety and pharmacokinetics of a novel oral niclosamide formulation compared with marketed niclosamide chewing tablets in healthy volunteers: A three-part randomized, double-blind, placebo-controlled trial
Source: PLoS One. 2025 Feb 25;20(2):e0303924. doi: 10.1371/journal.pone.0303924 (PMC11856320; doi:10.1371/journal.pone.0303924)
Supplement: S1 Tables — (PDF) [file pone.0303924.s005.pdf]

Statistical Output

Tables

Version 0.2 (03DEC2021)

Study: NIC-002

A 3-part study to investigate the safety and pharmacokinetics of a novel niclosamide solution as a treatment option for COVID-19 in combination with camostat

Sponsor: Charité Research GmbH

Author: GCP-Service Int. Ltd. & Co.KG
Matthes Metz
Anne-Conway-Straße 2
28359 Bremen, Germany

Table of content

Table 14.1: Subject disposition 3
Table 14.2: Demographics 4
Table 14.3.1: Medical history by MedDRA SOC and PT 10
Table 14.3.2.1: Prior medication by ATC levels 2 and 3 16
Table 14.3.2.2: Concomitant medication by ATC levels 2 and 3 19
Table 14.4.1: Brief summary of adverse events 22
Table 14.4.2: TEAEs by MedDRA SOC and PT 23
Table 14.4.3: Related TEAEs by MedDRA SOC and PT 27
Table 14.4.4 Deaths and Other Serious Adverse Events 30
Table 14.5.1: Safety Laboratory, Clinical Chemistry 33
Table 14.5.2: Safety Laboratory, Haematology 74
Table 14.6: Vital signs 120
Table 14.7: ECG Results 157

Table 14.1: Subject disposition

Part	Cohort/ Treatment/ Group	Treatment	Cond.	Randomized n	Randomized (%)	Treated n	Treated (%)	Completed n	Completed (%)	Premature Discontinuation n	Premature Discontinuation (%)
A	Cohort A1	200mg oral dose niclosamide		3	(100.0)	3	(100.0)	3	(100.0)	0	(0.0)
	Cohort A2	600mg oral dose niclosamide		3	(100.0)	3	(100.0)	3	(100.0)	0	(0.0)
	Cohort A3	1600mg oral dose niclosamide	Fast	3	(100.0)	3	(100.0)	3	(100.0)	0	(0.0)
			Fed	3	(100.0)	3	(100.0)	3	(100.0)	0	(0.0)
	Placebo			3	(100.0)	3	(100.0)	3	(100.0)	0	(0.0)
	Overall			12	(100.0)	12	(100.0)	12	(100.0)	0	(0.0)
B	Treatment 1	Solution 1600 mg		4	(100.0)	4	(100.0)	4	(100.0)	0	(0.0)
	Treatment 2	Chewing tablet 2000 mg		4	(100.0)	4	(100.0)	4	(100.0)	0	(0.0)
	Overall			4	(100.0)	4	(100.0)	4	(100.0)	0	(0.0)
C	Group 1	1200 mg niclosamide solution		4	(100.0)	4	(100.0)	4	(100.0)	0	(0.0)
	Group 2	1600 mg niclosamide solution		4	(100.0)	4	(100.0)	4	(100.0)	0	(0.0)
	Group 3	Placebo		4	(100.0)	4	(100.0)	4	(100.0)	0	(0.0)
	Overall			12	(100.0)	12	(100.0)	12	(100.0)	0	(0.0)

n: Number of non-missing observations; %: Percentage based on randomized subjects; Cond.: For cohort A3 the treatment administration was conducted under fasting ('fast') and fed conditions with the same subjects; Part B was a cross-over design;

Output generated by program 'NIC002_T14_1_Disposition_V02_0_0'

Table 14.2: Demographics

Part A

		Cohort A1		Cohort A2		Cohort A3		Placebo		Overall	
		n	(%)	n	(%)	n	(%)	n	(%)	n	(%)
Sex	Male	0	(0.0)	0	(0.0)	0	(0.0)	0	(0.0)	0	(0.0)
	Female	3	(100.0)	3	(100.0)	3	(100.0)	3	(100.0)	12	(100.0)
	Missing	0		0		0		0		0	
Age [yrs]	N	3		3		3		3		12	
	Mean	28.7		28.0		23.7		31.0		27.8	
	SD	9.1		2.0		2.5		7.8		6.0	
	Min	22		26		21		22		21	
	Max	39		30		26		36		39	
	Median	25.0		28.0		24.0		35.0		26.0	
Race	White/caucasian	3	(100.0)	3	(100.0)	3	(100.0)	3	(100.0)	12	(100.0)
	American indian or alaska native	0	(0.0)	0	(0.0)	0	(0.0)	0	(0.0)	0	(0.0)
	Native hawaiian or other pacific islander	0	(0.0)	0	(0.0)	0	(0.0)	0	(0.0)	0	(0.0)
	Asian	0	(0.0)	0	(0.0)	0	(0.0)	0	(0.0)	0	(0.0)
	Black or african american	0	(0.0)	0	(0.0)	0	(0.0)	0	(0.0)	0	(0.0)
	Other	0	(0.0)	0	(0.0)	0	(0.0)	0	(0.0)	0	(0.0)
	Missing	0		0		0		0		0	
Ethnicity	Not hispanic or latino	3	(100.0)	3	(100.0)	3	(100.0)	3	(100.0)	12	(100.0)
	Hispanic or latino	0	(0.0)	0	(0.0)	0	(0.0)	0	(0.0)	0	(0.0)
	Missing	0		0		0		0		0	
Smoking history	Current	0	(0.0)	0	(0.0)	0	(0.0)	0	(0.0)	0	(0.0)
	Former	2	(66.7)	0	(0.0)	0	(0.0)	1	(33.3)	3	(25.0)
	Never	1	(33.3)	3	(100.0)	3	(100.0)	2	(66.7)	9	(75.0)

n: Number of non-missing observations; %: Percentage based on non-missing observations; SD: Standard deviation; Min: Minimum; Max: Maximum; Cohort A1: 200mg oral dose niclosamide; Cohort A2: 600mg oral dose niclosamide; Cohort A3: 1600mg oral dose niclosamide;

Output generated by program 'NIC002_T14_2_Demographics_V02_0_0'

Table 14.2: Demographics

Part A

		Cohort A1		Cohort A2		Cohort A3		Placebo		Overall	
		n	(%)	n	(%)	n	(%)	n	(%)	n	(%)
Smoking history	Missing	0		0		0		0		0	
Weight [kg]	N	3		3		3		3		12	
	Mean	58.07		65.77		61.00		64.63		62.37	
	SD	0.80		13.23		2.15		14.51		9.01	
	Min	57.3		56.4		58.8		53.7		53.7	
	Max	58.9		80.9		63.1		81.1		81.1	
	Median	58.00		60.00		61.10		59.10		59.00	
Height [cm]	N	3		3		3		3		12	
	Mean	168.3		173.7		170.7		165.7		169.6	
	SD	3.1		3.8		2.1		11.6		6.2	
	Min	165		171		169		155		155	
	Max	171		178		173		178		178	
	Median	169.0		172.0		170.0		164.0		170.5	
Body mass index [kg/m^2]	N	3		3		3		3		12	
	Mean	20.47		21.70		20.93		23.33		21.61	
	SD	0.47		3.36		1.01		1.97		2.07	
	Min	20.1		19.1		20.3		22.0		19.1	
	Max	21.0		25.5		22.1		25.6		25.6	
	Median	20.30		20.50		20.40		22.40		20.75	

n: Number of non-missing observations; %: Percentage based on non-missing observations; SD: Standard deviation; Min: Minimum; Max: Maximum; Cohort A1: 200mg oral dose niclosamide; Cohort A2: 600mg oral dose niclosamide; Cohort A3: 1600mg oral dose niclosamide;

Output generated by program 'NIC002_T14_2_Demographics_V02_0_0'

Table 14.2: Demographics

Part B

		Overall	
		n	(%)
Sex	Male	0	(0.0)
	Female	4	(100.0)
	Missing	0	
Age [yrs]	N	4	
	Mean	28.8	
	SD	2.9	
	Min	25	
	Max	31	
	Median	29.5	
Race	White/caucasian	4	(100.0)
	American indian or alaska native	0	(0.0)
	Native hawaiian or other pacific islander	0	(0.0)
	Asian	0	(0.0)
	Black or african american	0	(0.0)
	Other	0	(0.0)
	Missing	0	
Ethnicity	Not hispanic or latino	4	(100.0)
	Hispanic or latino	0	(0.0)
	Missing	0	
Smoking history	Current	3	(75.0)
	Former	0	(0.0)
	Never	1	(25.0)

n: Number of non-missing observations; %: Percentage based on non-missing observations; SD: Standard deviation; Min: Minimum; Max: Maximum; Part B was cross-over design: Sequence 1: Solution 1600 mg - Chewing tablet 2000 mg; Sequence 2: Chewing tablet 2000 mg - Solution 1600 mg;

Output generated by program NIC002_T14_2_Demographics_V02_0_0

Table 14.2: Demographics

Part B

		Overall	
		n	(%)
Smoking history	Missing	0	
Weight [kg]	N	4	
	Mean	70.50	
	SD	16.02	
	Min	54.8	
	Max	91.8	
	Median	67.70	
Height [cm]	N	4	
	Mean	169.5	
	SD	6.7	
	Min	164	
	Max	179	
	Median	167.5	
Body mass index [kg/m ^{2}]	N	4	
	Mean	24.33	
	SD	3.59	
	Min	20.4	
	Max	28.7	
	Median	24.10	

n: Number of non-missing observations; %: Percentage based on non-missing observations; SD: Standard deviation; Min: Minimum; Max: Maximum; Part B was cross-over design: Sequence 1: Solution 1600 mg - Chewing tablet 2000 mg; Sequence 2: Chewing tablet 2000 mg - Solution 1600 mg;

Output generated by program NIC002_T14_2_Demographics_V02_0_0

Table 14.2: Demographics

Part C

		Group 1		Group 2		Group 3		Overall	
		n	(%)	n	(%)	n	(%)	n	(%)
Sex	Male	0	(0.0)	0	(0.0)	0	(0.0)	0	(0.0)
	Female	4	(100.0)	4	(100.0)	4	(100.0)	12	(100.0)
	Missing	0		0		0		0	
Age [yrs]	N	4		4		4		12	
	Mean	30.3		33.8		32.8		32.3	
	SD	5.7		6.4		6.3		5.8	
	Min	22		27		24		22	
	Max	35		42		39		42	
	Median	32.0		33.0		34.0		33.5	
Race	White/caucasian	4	(100.0)	4	(100.0)	4	(100.0)	12	(100.0)
	American indian or alaska native	0	(0.0)	0	(0.0)	0	(0.0)	0	(0.0)
	Native hawaiian or other pacific islander	0	(0.0)	0	(0.0)	0	(0.0)	0	(0.0)
	Asian	0	(0.0)	0	(0.0)	0	(0.0)	0	(0.0)
	Black or african american	0	(0.0)	0	(0.0)	0	(0.0)	0	(0.0)
	Other	0	(0.0)	0	(0.0)	0	(0.0)	0	(0.0)
	Missing	0		0		0		0	
Ethnicity	Not hispanic or latino	4	(100.0)	4	(100.0)	4	(100.0)	12	(100.0)
	Hispanic or latino	0	(0.0)	0	(0.0)	0	(0.0)	0	(0.0)
	Missing	0		0		0		0	
Smoking history	Current	1	(25.0)	0	(0.0)	1	(25.0)	2	(16.7)
	Former	1	(25.0)	3	(75.0)	1	(25.0)	5	(41.7)
	Never	2	(50.0)	1	(25.0)	2	(50.0)	5	(41.7)

n: Number of non-missing observations; %: Percentage based on non-missing observations; SD: Standard deviation; Min: Minimum; Max: Maximum; Group 1: 1200 mg niclosamide solution; Group 2: 1600 mg niclosamide solution; Group 3: Placebo;

Output generated by program NIC002_T14_2_Demographics_V02_0_0

Table 14.2: Demographics

Part C

		Group 1		Group 2		Group 3		Overall	
		n	(%)	n	(%)	n	(%)	n	(%)
Smoking history	Missing	0		0		0		0	
Weight [kg]	N	4		4		4		12	
	Mean	63.70		70.75		62.45		65.63	
	SD	16.56		7.10		7.31		10.85	
	Min	49.4		63.5		55.8		49.4	
	Max	82.8		78.7		71.5		82.8	
	Median	61.30		70.40		61.25		65.70	
Height [cm]	N	4		4		4		12	
	Mean	166.8		164.8		164.8		165.4	
	SD	5.7		4.4		5.1		4.7	
	Min	159		160		159		159	
	Max	171		169		171		171	
	Median	168.5		165.0		164.5		166.0	
Body mass index [kg/m^2]	N	4		4		4		12	
	Mean	22.70		26.00		23.08		23.92	
	SD	4.65		1.26		3.06		3.36	
	Min	18.3		24.8		19.6		18.3	
	Max	28.3		27.6		26.9		28.3	
	Median	22.10		25.80		22.90		24.75	

n: Number of non-missing observations; %: Percentage based on non-missing observations; SD: Standard deviation; Min: Minimum; Max: Maximum; Group 1: 1200 mg niclosamide solution; Group 2: 1600 mg niclosamide solution; Group 3: Placebo;

Output generated by program NIC002_T14_2_Demographics_V02_0_0

Table 14.3.1: Medical history and prior and concomitant medication
Medical history by MedDRA SOC and PT

PART A

MedDRA System organ class<br>Preferred term	Cohort A1 (N = 3)			Cohort A2 (N = 3)			Cohort A3 (N = 3)			Placebo (N = 3)			Part A Overall (N = 12)		
	n	(%)	m	n	(%)	m	n	(%)	m	n	(%)	m	n	(%)	m
Any	2	(66.7)	3	2	(66.7)	9	3	(100.0)	6	2	(66.7)	9	9	(75.0)	27
Surgical and medical procedures	2	(66.7)	2	2	(66.7)	4	3	(100.0)	4	2	(66.7)	5	9	(75.0)	15
Appendicectomy	1	(33.3)	1	1	(33.3)	1	0	(0.0)	0	0	(0.0)	0	2	(16.7)	2
Nasal septal operation	1	(33.3)	1	0	(0.0)	0	1	(33.3)	1	0	(0.0)	0	2	(16.7)	2
Tonsillectomy	0	(0.0)	0	0	(0.0)	0	1	(33.3)	1	1	(33.3)	1	2	(16.7)	2
Ear tube insertion	0	(0.0)	0	1	(33.3)	1	0	(0.0)	0	0	(0.0)	0	1	(8.3)	1
Foot operation	0	(0.0)	0	0	(0.0)	0	0	(0.0)	0	1	(33.3)	1	1	(8.3)	1
Hernia repair	0	(0.0)	0	1	(33.3)	1	0	(0.0)	0	0	(0.0)	0	1	(8.3)	1
Joint surgery	0	(0.0)	0	0	(0.0)	0	0	(0.0)	0	1	(33.3)	1	1	(8.3)	1
Keratomileusis	0	(0.0)	0	0	(0.0)	0	0	(0.0)	0	1	(33.3)	1	1	(8.3)	1
Mammoplasty	0	(0.0)	0	0	(0.0)	0	1	(33.3)	1	0	(0.0)	0	1	(8.3)	1
Nasal operation	0	(0.0)	0	0	(0.0)	0	1	(33.3)	1	0	(0.0)	0	1	(8.3)	1
Tympanoplasty	0	(0.0)	0	0	(0.0)	0	0	(0.0)	0	1	(33.3)	1	1	(8.3)	1
Wisdom teeth removal	0	(0.0)	0	1	(33.3)	1	0	(0.0)	0	0	(0.0)	0	1	(8.3)	1
Infections and infestations	1	(33.3)	1	2	(66.7)	2	1	(33.3)	1	1	(33.3)	2	5	(41.7)	6
Appendicitis	1	(33.3)	1	1	(33.3)	1	0	(0.0)	0	0	(0.0)	0	2	(16.7)	2
Tonsillitis	0	(0.0)	0	0	(0.0)	0	1	(33.3)	1	1	(33.3)	1	2	(16.7)	2
Ear infection	0	(0.0)	0	0	(0.0)	0	0	(0.0)	0	1	(33.3)	1	1	(8.3)	1
Otitis media	0	(0.0)	0	1	(33.3)	1	0	(0.0)	0	0	(0.0)	0	1	(8.3)	1
Injury, poisoning and procedural complications	0	(0.0)	0	1	(33.3)	2	0	(0.0)	0	1	(33.3)	1	2	(16.7)	3
Hand fracture	0	(0.0)	0	1	(33.3)	1	0	(0.0)	0	0	(0.0)	0	1	(8.3)	1

N: Number of subjects in respective cohort; n: Number of patients with at least one medical history in respective category; %: Percentage based on N; m: Number of medical history entries; Cohort A1: 200mg oral dose niclosamide; Cohort A2: 600mg oral dose niclosamide; Cohort A3: 1600mg oral dose niclosamide;

Output generated by program 'NIC002_T14_3_1_MH_V02_0_0'

Table 14.3.1: Medical history and prior and concomitant medication
Medical history by MedDRA SOC and PT

PART A

MedDRA System organ class<br>Preferred term	Cohort A1 (N = 3)			Cohort A2 (N = 3)			Cohort A3 (N = 3)			Placebo (N = 3)			Part A Overall (N = 12)		
	n	(%)	m	n	(%)	m	n	(%)	m	n	(%)	m	n	(%)	m
Jaw fracture	0	(0.0)	0	1	(33.3)	1	0	(0.0)	0	0	(0.0)	0	1	(8.3)	1
Upper limb fracture	0	(0.0)	0	0	(0.0)	0	0	(0.0)	0	1	(33.3)	1	1	(8.3)	1
Gastrointestinal disorders	0	(0.0)	0	1	(33.3)	1	0	(0.0)	0	0	(0.0)	0	1	(8.3)	1
Umbilical hernia	0	(0.0)	0	1	(33.3)	1	0	(0.0)	0	0	(0.0)	0	1	(8.3)	1
Immune system disorders	0	(0.0)	0	0	(0.0)	0	1	(33.3)	1	0	(0.0)	0	1	(8.3)	1
Drug hypersensitivity	0	(0.0)	0	0	(0.0)	0	1	(33.3)	1	0	(0.0)	0	1	(8.3)	1
Skin and subcutaneous tissue disorders	0	(0.0)	0	0	(0.0)	0	0	(0.0)	0	1	(33.3)	1	1	(8.3)	1
Dermatitis contact	0	(0.0)	0	0	(0.0)	0	0	(0.0)	0	1	(33.3)	1	1	(8.3)	1

N: Number of subjects in respective cohort; n: Number of patients with at least one medical history in respective category; %: Percentage based on N; m: Number of medical history entries; Cohort A1: 200mg oral dose niclosamide; Cohort A2: 600mg oral dose niclosamide; Cohort A3: 1600mg oral dose niclosamide;

Output generated by program 'NIC002_T14_3_1_MH_V02_0_0'

Table 14.3.1: Medical history and prior and concomitant medication
Medical history by MedDRA SOC and PT

PART B

MedDRA System organ class<br>Preferred term	Part B Overall (N = 4)		
	n	(%)	m
Any	4	(100.0)	16
Surgical and medical procedures	4	(100.0)	6
Cervical conisation	1	(25.0)	1
Hernia repair	1	(25.0)	1
Internal fixation of fracture	1	(25.0)	1
Limb reconstructive surgery	1	(25.0)	1
Mammoplasty	1	(25.0)	1
Tonsillectomy	1	(25.0)	1
Infections and infestations	2	(50.0)	4
Bacterial vaginosis	1	(25.0)	1
Meningitis	1	(25.0)	2
Tonsillitis	1	(25.0)	1
Congenital, familial and genetic disorders	1	(25.0)	1
Limb malformation	1	(25.0)	1
Gastrointestinal disorders	1	(25.0)	1
Umbilical hernia	1	(25.0)	1
Injury, poisoning and procedural complications	1	(25.0)	2
Hand fracture	1	(25.0)	1

N: Number of subjects in respective cohort; n: Number of patients with at least one medical history in respective category; %: Percentage based on N; m: Number of medical history entries; Part B used a cross-over design; Sequence 1: Solution 1600 mg - Chewing tablet 2000 mg; Sequence 2: Chewing tablet 2000 mg - Solution 1600 mg;

Output generated by program 'NIC002_T14_3_1_MH_V02_0_0'

Table 14.3.1: Medical history and prior and concomitant medication
Medical history by MedDRA SOC and PT

PART B

MedDRA System organ class<br>Preferred term	Part B Overall (N = 4)		
	n	(%)	m
Skin injury	1	(25.0)	1
Nervous system disorders	1	(25.0)	1
Headache	1	(25.0)	1
Reproductive system and breast disorders	1	(25.0)	1
Cervical dysplasia	1	(25.0)	1

N: Number of subjects in respective cohort; n: Number of patients with at least one medical history in respective category; %: Percentage based on N; m: Number of medical history entries; Part B used a cross-over design; Sequence 1: Solution 1600 mg - Chewing tablet 2000 mg; Sequence 2: Chewing tablet 2000 mg - Solution 1600 mg;

Output generated by program 'NIC002_T14_3_1_MH_V02_0_0'

Table 14.3.1: Medical history and prior and concomitant medication
Medical history by MedDRA SOC and PT

PART C

MedDRA System organ class<br>Preferred term	Group 1 (N = 4)			Group 2 (N = 4)			Group 3 (N = 4)			Part C Overall (N = 12)		
	n	(%)	m	n	(%)	m	n	(%)	m	n	(%)	m
Any	4	(100.0)	8	2	(50.0)	4	3	(75.0)	6	9	(75.0)	18
Surgical and medical procedures	3	(75.0)	5	2	(50.0)	2	2	(50.0)	3	7	(58.3)	10
Arthroscopic surgery	1	(25.0)	1	0	(0.0)	0	1	(25.0)	1	2	(16.7)	2
Appendicectomy	0	(0.0)	0	1	(25.0)	1	0	(0.0)	0	1	(8.3)	1
Caesarean section	0	(0.0)	0	1	(25.0)	1	0	(0.0)	0	1	(8.3)	1
Internal fixation of fracture	1	(25.0)	1	0	(0.0)	0	0	(0.0)	0	1	(8.3)	1
Liposuction	1	(25.0)	2	0	(0.0)	0	0	(0.0)	0	1	(8.3)	2
Mammoplasty	0	(0.0)	0	0	(0.0)	0	1	(25.0)	1	1	(8.3)	1
Rhinoplasty	1	(25.0)	1	0	(0.0)	0	0	(0.0)	0	1	(8.3)	1
Tonsillectomy	0	(0.0)	0	0	(0.0)	0	1	(25.0)	1	1	(8.3)	1
Injury, poisoning and procedural complications	2	(50.0)	2	0	(0.0)	0	1	(25.0)	1	3	(25.0)	3
Ligament injury	0	(0.0)	0	0	(0.0)	0	1	(25.0)	1	1	(8.3)	1
Meniscus injury	1	(25.0)	1	0	(0.0)	0	0	(0.0)	0	1	(8.3)	1
Radius fracture	1	(25.0)	1	0	(0.0)	0	0	(0.0)	0	1	(8.3)	1
Infections and infestations	0	(0.0)	0	1	(25.0)	1	1	(25.0)	1	2	(16.7)	2
Appendicitis	0	(0.0)	0	1	(25.0)	1	0	(0.0)	0	1	(8.3)	1
Tonsillitis	0	(0.0)	0	0	(0.0)	0	1	(25.0)	1	1	(8.3)	1
Eye disorders	1	(25.0)	1	0	(0.0)	0	0	(0.0)	0	1	(8.3)	1
Pupils unequal	1	(25.0)	1	0	(0.0)	0	0	(0.0)	0	1	(8.3)	1

N: Number of subjects in respective cohort; n: Number of patients with at least one medical history in respective category; %: Percentage based on N; m: Number of medical history entries; Group 1: 1200 mg niclosamide solution; Group 2: 1600 mg niclosamide solution; Group 3: Placebo;

Output generated by program 'NIC002_T14_3_1_MH_V02_0_0'

Table 14.3.1: Medical history and prior and concomitant medication
Medical history by MedDRA SOC and PT

PART C

MedDRA System organ class<br>Preferred term	Group 1 (N = 4)			Group 2 (N = 4)			Group 3 (N = 4)			Part C Overall (N = 12)		
	n	(%)	m	n	(%)	m	n	(%)	m	n	(%)	m
Psychiatric disorders	0	(0.0)	0	1	(25.0)	1	0	(0.0)	0	1	(8.3)	1
Intentional self-injury	0	(0.0)	0	1	(25.0)	1	0	(0.0)	0	1	(8.3)	1
Skin and subcutaneous tissue disorders	0	(0.0)	0	0	(0.0)	0	1	(25.0)	1	1	(8.3)	1
Acne	0	(0.0)	0	0	(0.0)	0	1	(25.0)	1	1	(8.3)	1

N: Number of subjects in respective cohort; n: Number of patients with at least one medical history in respective category; %: Percentage based on N; m: Number of medical history entries; Group 1: 1200 mg niclosamide solution; Group 2: 1600 mg niclosamide solution; Group 3: Placebo;

Output generated by program 'NIC002_T14_3_1_MH_V02_0_0'

Table 14.3.2.1: Medical history and prior and concomitant medication
Prior and concomitant medication
Prior medication by ATC levels 2 and 3

PART A

ATC Level 2	Cohort A1			Cohort A2			Cohort A3						Placebo			Part A Overall		
ATC Level 3	(N = 3)			(N = 3)			(N_{Fast} = 3)			(N_{Fed} = 3)			(N = 3)			(N = 12)		
	n	(%)	m	n	(%)	m	n	(%)	m	n	(%)	m	n	(%)	m	n	(%)	m

There were no findings in prior medication for part A.

N: Number of subjects in respective cohort; n: Number of patients with at least one medical history in respective category; %: Percentage based on N; m: Number of medication entries; Cohort A1: 200mg oral dose niclosamide; Cohort A2: 600mg oral dose niclosamide; Cohort A3: 1600mg oral dose niclosamide; Fast/Fed: Treatment in cohort A3 was applied under fasting and fed conditions in the same subjects;

Output generated by program 'NIC002_T14_3_2_ConMed_V02_0_0'

Table 14.3.2.1: Medical history and prior and concomitant medication
Prior and concomitant medication
Prior medication by ATC levels 2 and 3

PART B

ATC Level 2<br>ATC Level 3	Part B Overall (N = 4)		
	n	(%)	m
Any	2	(50.0)	2
ANTIINFLAMMATORY AND ANTIRHEUMATIC PRODUCTS	1	(25.0)	1
ANTIINFLAMMATORY AND ANTIRHEUMATIC PRODUCTS, NON-STEROIDS	1	(25.0)	1
GYNECOLOGICAL ANTIINFECTIVES AND ANTISEPTICS	1	(25.0)	1
ANTIINFECTIVES AND ANTISEPTICS, EXCL. COMBINATIONS WITH CORTICOSTEROIDS	1	(25.0)	1

N: Number of subjects in respective cohort; n: Number of patients with at least one medical history in respective category; %: Percentage based on N; m: Number of medication entries; Part B used a cross-over design;

Output generated by program 'NIC002_T14_3_2_ConMed_V02_0_0'

Table 14.3.2.1: Medical history and prior and concomitant medication
Prior and concomitant medication
Prior medication by ATC levels 2 and 3

PART C

ATC Level 2<br>ATC Level 3	Group 1 (N = 4)			Group 2 (N = 4)			Group 3 (N = 4)			Part C Overall (N = 12)		
	n	(%)	m	n	(%)	m	n	(%)	m	n	(%)	m
Any	0	(0.0)	0	1	(25.0)	2	0	(0.0)	0	1	(8.3)	2
SEX HORMONES AND MODULATORS OF THE GENITAL SYSTEM	0	(0.0)	0	1	(25.0)	2	0	(0.0)	0	1	(8.3)	2
HORMONAL CONTRACEPTIVES FOR SYSTEMIC USE	0	(0.0)	0	1	(25.0)	2	0	(0.0)	0	1	(8.3)	2

N: Number of subjects in respective cohort; n: Number of patients with at least one medical history in respective category; %: Percentage based on N; m: Number of medication entries; Group 1: 1200 mg niclosamide solution; Group 2: 1600 mg niclosamide solution; Group 3: Placebo;

Output generated by program 'NIC002_T14_3_2_ConMed_V02_0_0'

Table 14.3.2.2: Medical history and prior and concomitant medication
Prior and concomitant medication
Concomitant medication by ATC levels 2 and 3

PART A

ATC Level 2<br>ATC Level 3	Cohort A1 (N = 3)			Cohort A2 (N = 3)			Cohort A3 (N_{Fast} = 3)			Cohort A3 (N_{Fed} = 3)			Placebo (N = 3)			Part A Overall (N = 12)		
	n	(%)	m	n	(%)	m	n	(%)	m	n	(%)	m	n	(%)	m	n	(%)	m
Any	0	(0.0)	0	2	(66.7)	3	0	(0.0)	0	0	(0.0)	0	0	(0.0)	0	2	(16.7)	3
OTHER GYNECOLOGICALS	0	(0.0)	0	2	(66.7)	2	0	(0.0)	0	0	(0.0)	0	0	(0.0)	0	2	(16.7)	2
CONTRACEPTIVES FOR TOPICAL USE	0	(0.0)	0	2	(66.7)	2	0	(0.0)	0	0	(0.0)	0	0	(0.0)	0	2	(16.7)	2
ANTIINFLAMMATORY AND ANTIRHEUMATIC PRODUCTS	0	(0.0)	0	1	(33.3)	1	0	(0.0)	0	0	(0.0)	0	0	(0.0)	0	1	(8.3)	1
ANTIINFLAMMATORY AND ANTIRHEUMATIC PRODUCTS, NON-STEROIDS	0	(0.0)	0	1	(33.3)	1	0	(0.0)	0	0	(0.0)	0	0	(0.0)	0	1	(8.3)	1

N: Number of subjects in respective cohort; n: Number of patients with at least one medical history in respective category; %: Percentage based on N; m: Number of medication entries; Cohort A1: 200mg oral dose niclosamide; Cohort A2: 600mg oral dose niclosamide; Cohort A3: 1600mg oral dose niclosamide; Fast/Fed: Treatment in cohort A3 was applied under fasting and fed conditions in the same subjects;

Output generated by program 'NIC002_T14_3_2_ConMed_V02_0_0'

Table 14.3.2.2: Medical history and prior and concomitant medication
Prior and concomitant medication
Concomitant medication by ATC levels 2 and 3

PART B

ATC Level 2<br>ATC Level 3	Solution 1600mg (N = 4)			Chewing tablet 2000mg (N = 4)			Part B Overall (N = 4)		
	n	(%)	m	n	(%)	m	n	(%)	m
Any	1	(25.0)	1	1	(25.0)	1	1	(25.0)	1
OTHER GYNECOLOGICALS	1	(25.0)	1	1	(25.0)	1	1	(25.0)	1
CONTRACEPTIVES FOR TOPICAL USE	1	(25.0)	1	1	(25.0)	1	1	(25.0)	1

N: Number of subjects in respective cohort; n: Number of patients with at least one medical history in respective category; %: Percentage based on N; m: Number of medication entries; Part B used a cross-over design;

Output generated by program 'NIC002_T14_3_2_ConMed_V02_0_0'

Table 14.3.2.2: Medical history and prior and concomitant medication
Prior and concomitant medication
Concomitant medication by ATC levels 2 and 3

PART C

ATC Level 2<br>ATC Level 3	Group 1 (N = 4)			Group 2 (N = 4)			Group 3 (N = 4)			Part C Overall (N = 12)		
	n	(%)	m	n	(%)	m	n	(%)	m	n	(%)	m
Any	1	(25.0)	2	2	(50.0)	4	2	(50.0)	3	5	(41.7)	9
SEX HORMONES AND MODULATORS OF THE GENITAL SYSTEM	1	(25.0)	2	2	(50.0)	4	2	(50.0)	3	5	(41.7)	9
HORMONAL CONTRACEPTIVES FOR SYSTEMIC USE	1	(25.0)	2	2	(50.0)	4	2	(50.0)	3	5	(41.7)	9

N: Number of subjects in respective cohort; n: Number of patients with at least one medical history in respective category; %: Percentage based on N; m: Number of medication entries; Group 1: 1200 mg niclosamide solution; Group 2: 1600 mg niclosamide solution; Group 3: Placebo;

Output generated by program 'NIC002_T14_3_2_ConMed_V02_0_0'

Table 14.4.1: Adverse events
Brief summary of adverse events

Part			Any pre-treatment AE			Any TEAE			Any related TEAE			Any severe TEAE			Any serious TEAE			Any serious related TEAE			Any fatal TEAE		
		N	n	(%)	m	n	(%)	m	n	(%)	m	n	(%)	m	n	(%)	m	n	(%)	m	n	(%)	m
A	Cohort A1	3	0	(0.0)	0	0	(0.0)	0	0	(0.0)	0	0	(0.0)	0	0	(0.0)	0	0	(0.0)	0	0	(0.0)	0
	Cohort A2	3	0	(0.0)	0	2	(66.7)	7	2	(66.7)	7	0	(0.0)	0	0	(0.0)	0	0	(0.0)	0	0	(0.0)	0
	Cohort A3 Fast	3	0	(0.0)	0	3	(100.0)	7	3	(100.0)	6	0	(0.0)	0	0	(0.0)	0	0	(0.0)	0	0	(0.0)	0
	Fed	3	0	(0.0)	0	2	(66.7)	3	2	(66.7)	3	0	(0.0)	0	0	(0.0)	0	0	(0.0)	0	0	(0.0)	0
	Placebo	3	1	(33.3)	1	2	(66.7)	2	2	(66.7)	2	0	(0.0)	0	0	(0.0)	0	0	(0.0)	0	0	(0.0)	0
	Overall	12	1	(8.3)	1	7	(58.3)	19	7	(58.3)	18	0	(0.0)	0	0	(0.0)	0	0	(0.0)	0	0	(0.0)	0
B	Chewing tablet 2000 mg	4	0	(0.0)	0	0	(0.0)	0	0	(0.0)	0	0	(0.0)	0	0	(0.0)	0	0	(0.0)	0	0	(0.0)	0
	Solution 1600 mg	4	0	(0.0)	0	2	(50.0)	3	2	(50.0)	3	0	(0.0)	0	0	(0.0)	0	0	(0.0)	0	0	(0.0)	0
	Overall	4	0	(0.0)	0	2	(50.0)	3	2	(50.0)	3	0	(0.0)	0	0	(0.0)	0	0	(0.0)	0	0	(0.0)	0
C	Group 1	4	0	(0.0)	0	4	(100.0)	13	4	(100.0)	11	0	(0.0)	0	0	(0.0)	0	0	(0.0)	0	0	(0.0)	0
	Group 2	4	0	(0.0)	0	4	(100.0)	14	4	(100.0)	10	0	(0.0)	0	0	(0.0)	0	0	(0.0)	0	0	(0.0)	0
	Group 3	4	0	(0.0)	0	4	(100.0)	9	4	(100.0)	7	0	(0.0)	0	0	(0.0)	0	0	(0.0)	0	0	(0.0)	0
	Overall	12	0	(0.0)	0	12	(100.0)	36	12	(100.0)	28	0	(0.0)	0	0	(0.0)	0	0	(0.0)	0	0	(0.0)	0

N: Number of subjects in respective cohort; n: Number of patients with at least one adverse event in respective category; %: Percentage based on N; m: Number of events; AE: Adverse events; TEAE: Treatment-emergent adverse events;
Part A: Cohort A1: 200mg oral dose niclosamide; Cohort A2: 600mg oral dose niclosamide; Cohort A3: 1600mg oral dose niclosamide; Fast/Fed: Treatment in cohort A3 was applied under fasting and fed conditions in the same subjects; Subject 27, who was treated with placebo in cohort A3 had mild, related AE during the treatment phase under fasting condition;
Part B was a cross-over design; AEs were allocated to the treatment periods by using the start date of the AEs
Part C: Group 1: 1200 mg niclosamide solution; Group 2: 1600 mg niclosamide solution; Group 3: Placebo;

Output generated by program 'NIC002_T14_4_1_AEs_Summary_V02_0_0'

Table 14.4.2: Adverse events
TEAEs by MedDRA SOC and PT

PART A

MedDRA System organ class<br>Preferred term	Cohort A1<br>(N = 3)			Cohort A2<br>(N = 3)			Cohort A3<br>(N_{Fast} = 3)			Cohort A3<br>(N_{Fed} = 3)			Placebo<br>(N = 3)			Part A Overall<br>(N = 12)		
	n	(%)	m	n	(%)	m	n	(%)	m	n	(%)	m	n	(%)	m	n	(%)	m
Any	0	(0.0)	0	2	(66.7)	7	3	(100.0)	7	2	(66.7)	3	2	(66.7)	2	7	(58.3)	19
Gastrointestinal disorders	0	(0.0)	0	1	(33.3)	4	3	(100.0)	6	2	(66.7)	3	2	(66.7)	2	6	(50.0)	15
Diarrhoea	0	(0.0)	0	1	(33.3)	1	1	(33.3)	1	1	(33.3)	1	0	(0.0)	0	3	(25.0)	3
Nausea	0	(0.0)	0	1	(33.3)	1	2	(66.7)	2	1	(33.3)	1	0	(0.0)	0	3	(25.0)	4
Faeces soft	0	(0.0)	0	0	(0.0)	0	1	(33.3)	1	0	(0.0)	0	1	(33.3)	1	2	(16.7)	2
Oral disorder	0	(0.0)	0	0	(0.0)	0	1	(33.3)	1	1	(33.3)	1	1	(33.3)	1	2	(16.7)	3
Abdominal pain	0	(0.0)	0	1	(33.3)	1	0	(0.0)	0	0	(0.0)	0	0	(0.0)	0	1	(8.3)	1
Dyspepsia	0	(0.0)	0	0	(0.0)	0	1	(33.3)	1	0	(0.0)	0	0	(0.0)	0	1	(8.3)	1
Vomiting	0	(0.0)	0	1	(33.3)	1	0	(0.0)	0	0	(0.0)	0	0	(0.0)	0	1	(8.3)	1
Musculoskeletal and connective tissue disorders	0	(0.0)	0	0	(0.0)	0	1	(33.3)	1	0	(0.0)	0	0	(0.0)	0	1	(8.3)	1
Back pain	0	(0.0)	0	0	(0.0)	0	1	(33.3)	1	0	(0.0)	0	0	(0.0)	0	1	(8.3)	1
Respiratory, thoracic and mediastinal disorders	0	(0.0)	0	1	(33.3)	2	0	(0.0)	0	0	(0.0)	0	0	(0.0)	0	1	(8.3)	2
Dry throat	0	(0.0)	0	1	(33.3)	1	0	(0.0)	0	0	(0.0)	0	0	(0.0)	0	1	(8.3)	1
Throat clearing	0	(0.0)	0	1	(33.3)	1	0	(0.0)	0	0	(0.0)	0	0	(0.0)	0	1	(8.3)	1
Skin and subcutaneous tissue disorders	0	(0.0)	0	1	(33.3)	1	0	(0.0)	0	0	(0.0)	0	0	(0.0)	0	1	(8.3)	1

N: Number of subjects in respective cohort; n: Number of patients with at least one adverse event in respective category; %: Percentage based on N; m: Number of events; TEAE: Treatment-emergent adverse events; Cohort A1: 200mg oral dose niclosamide; Cohort A2: 600mg oral dose niclosamide; Cohort A3: 1600mg oral dose niclosamide; Fast/Fed: Treatment in cohort A3 was applied under fasting and fed conditions in the same subjects; Subject 27, who was treated with placebo in cohort A3 had one AE (Gastrointestinal disorders: Faeces soft) under fasting condition;

Output generated by program 'NIC002_T14_4_2_TEAEs_SocPT_V02_0_0'

Table 14.4.2: Adverse events
TEAEs by MedDRA SOC and PT

PART A

MedDRA System organ class<br>Preferred term	Cohort A1 (N = 3)			Cohort A2 (N = 3)			Cohort A3 (N_{Fast} = 3)			Cohort A3 (N_{Fed} = 3)			Placebo (N = 3)			Part A Overall (N = 12)		
	n	(%)	m	n	(%)	m	n	(%)	m	n	(%)	m	n	(%)	m	n	(%)	m
Erythema	0	(0.0)	0	1	(33.3)	1	0	(0.0)	0	0	(0.0)	0	0	(0.0)	0	1	(8.3)	1

N: Number of subjects in respective cohort; n: Number of patients with at least one adverse event in respective category; %: Percentage based on N; m: Number of events; TEAE: Treatment-emergent adverse events; Cohort A1: 200mg oral dose niclosamide; Cohort A2: 600mg oral dose niclosamide; Cohort A3: 1600mg oral dose niclosamide; Fast/Fed: Treatment in cohort A3 was applied under fasting and fed conditions in the same subjects; Subject 27, who was treated with placebo in cohort A3 had one AE (Gastrointestinal disorders: Faeces soft) under fasting condition;

Output generated by program 'NIC002_T14_4_2_TEAEs_SocPT_V02_0_0'

Table 14.4.2: Adverse events
TEAEs by MedDRA SOC and PT

PART B

MedDRA System organ class<br>Preferred term	Solution 1600mg (N = 4)			Chewing tablet 2000mg (N = 4)			Part B Overall (N = 4)		
	n	(%)	m	n	(%)	m	n	(%)	m
Any	2	(50.0)	3	0	(0.0)	0	2	(50.0)	3
Gastrointestinal disorders	2	(50.0)	3	0	(0.0)	0	2	(50.0)	3
Diarrhoea	1	(25.0)	1	0	(0.0)	0	1	(25.0)	1
Nausea	1	(25.0)	1	0	(0.0)	0	1	(25.0)	1
Vomiting	1	(25.0)	1	0	(0.0)	0	1	(25.0)	1

N: Number of subjects in respective cohort; n: Number of patients with at least one adverse event in respective category; %: Percentage based on N; m: Number of events; TEAE: Treatment-emergent adverse events; Part B was a cross-over design; AEs were allocated to the treatment periods by using the start date of the AEs;

Output generated by program 'NIC002_T14_4_2_TEAEs_SocPT_V02_0_0'

Table 14.4.2: Adverse events
TEAEs by MedDRA SOC and PT

PART C

MedDRA System organ class<br>Preferred term	Group 1<br>(N = 4)			Group 2<br>(N = 4)			Group 3<br>(N = 4)			Part C Overall<br>(N = 12)		
	n	(%)	m	n	(%)	m	n	(%)	m	n	(%)	m
Any	4	(100.0)	13	4	(100.0)	14	4	(100.0)	9	12	(100.0)	36
Gastrointestinal disorders	4	(100.0)	9	4	(100.0)	11	4	(100.0)	7	12	(100.0)	27
Diarrhoea	4	(100.0)	4	4	(100.0)	5	3	(75.0)	3	11	(91.7)	12
Nausea	2	(50.0)	2	4	(100.0)	4	1	(25.0)	1	7	(58.3)	7
Flatulence	1	(25.0)	1	0	(0.0)	0	2	(50.0)	2	3	(25.0)	3
Vomiting	0	(0.0)	0	2	(50.0)	2	0	(0.0)	0	2	(16.7)	2
Dry mouth	1	(25.0)	1	0	(0.0)	0	0	(0.0)	0	1	(8.3)	1
Faeces soft	0	(0.0)	0	0	(0.0)	0	1	(25.0)	1	1	(8.3)	1
Rectal haemorrhage	1	(25.0)	1	0	(0.0)	0	0	(0.0)	0	1	(8.3)	1
Nervous system disorders	2	(50.0)	2	1	(25.0)	2	1	(25.0)	1	4	(33.3)	5
Headache	2	(50.0)	2	1	(25.0)	2	1	(25.0)	1	4	(33.3)	5
General disorders and administration site conditions	1	(25.0)	1	0	(0.0)	0	1	(25.0)	1	2	(16.7)	2
Fatigue	0	(0.0)	0	0	(0.0)	0	1	(25.0)	1	1	(8.3)	1
Puncture site pain	1	(25.0)	1	0	(0.0)	0	0	(0.0)	0	1	(8.3)	1
Infections and infestations	0	(0.0)	0	1	(25.0)	1	0	(0.0)	0	1	(8.3)	1
Rhinitis	0	(0.0)	0	1	(25.0)	1	0	(0.0)	0	1	(8.3)	1
Respiratory, thoracic and mediastinal disorders	1	(25.0)	1	0	(0.0)	0	0	(0.0)	0	1	(8.3)	1
Cough	1	(25.0)	1	0	(0.0)	0	0	(0.0)	0	1	(8.3)	1

N: Number of subjects in respective cohort; n: Number of patients with at least one adverse event in respective category; %: Percentage based on N; m: Number of events; TEAE: Treatment-emergent adverse events; Group 1: 1200 mg niclosamide solution; Group 2: 1600 mg niclosamide solution; Group 3: Placebo;

Output generated by program 'NIC002_T14_4_2_TEAEs_SocPT_V02_0_0'

Table 14.4.3: Adverse events
Related TEAEs by MedDRA SOC and PT

PART A

MedDRA System organ class<br>Preferred term	Cohort A1 (N = 3)			Cohort A2 (N = 3)			Cohort A3 (N_{Fast} = 3)			Cohort A3 (N_{Fed} = 3)			Placebo (N = 3)			Part A Overall (N = 12)		
	n	(%)	m	n	(%)	m	n	(%)	m	n	(%)	m	n	(%)	m	n	(%)	m
Any	0	(0.0)	0	2	(66.7)	7	3	(100.0)	6	2	(66.7)	3	2	(66.7)	2	7	(58.3)	18
Gastrointestinal disorders	0	(0.0)	0	1	(33.3)	4	3	(100.0)	6	2	(66.7)	3	2	(66.7)	2	6	(50.0)	15
Diarrhoea	0	(0.0)	0	1	(33.3)	1	1	(33.3)	1	1	(33.3)	1	0	(0.0)	0	3	(25.0)	3
Nausea	0	(0.0)	0	1	(33.3)	1	2	(66.7)	2	1	(33.3)	1	0	(0.0)	0	3	(25.0)	4
Faeces soft	0	(0.0)	0	0	(0.0)	0	1	(33.3)	1	0	(0.0)	0	1	(33.3)	1	2	(16.7)	2
Oral disorder	0	(0.0)	0	0	(0.0)	0	1	(33.3)	1	1	(33.3)	1	1	(33.3)	1	2	(16.7)	3
Abdominal pain	0	(0.0)	0	1	(33.3)	1	0	(0.0)	0	0	(0.0)	0	0	(0.0)	0	1	(8.3)	1
Dyspepsia	0	(0.0)	0	0	(0.0)	0	1	(33.3)	1	0	(0.0)	0	0	(0.0)	0	1	(8.3)	1
Vomiting	0	(0.0)	0	1	(33.3)	1	0	(0.0)	0	0	(0.0)	0	0	(0.0)	0	1	(8.3)	1
Respiratory, thoracic and mediastinal disorders	0	(0.0)	0	1	(33.3)	2	0	(0.0)	0	0	(0.0)	0	0	(0.0)	0	1	(8.3)	2
Dry throat	0	(0.0)	0	1	(33.3)	1	0	(0.0)	0	0	(0.0)	0	0	(0.0)	0	1	(8.3)	1
Throat clearing	0	(0.0)	0	1	(33.3)	1	0	(0.0)	0	0	(0.0)	0	0	(0.0)	0	1	(8.3)	1
Skin and subcutaneous tissue disorders	0	(0.0)	0	1	(33.3)	1	0	(0.0)	0	0	(0.0)	0	0	(0.0)	0	1	(8.3)	1
Erythema	0	(0.0)	0	1	(33.3)	1	0	(0.0)	0	0	(0.0)	0	0	(0.0)	0	1	(8.3)	1

N: Number of subjects in respective cohort; n: Number of patients with at least one adverse event in respective category; %: Percentage based on N; m: Number of events; TEAE: Treatment-emergent adverse events; Cohort A1: 200mg oral dose niclosamide; Cohort A2: 600mg oral dose niclosamide; Cohort A3: 1600mg oral dose niclosamide; Fast/Fed: Treatment in cohort A3 was applied under fasting and fed conditions in the same subjects; Subject 27, who was treated with placebo in cohort A3 had one related AE (Gastrointestinal disorders: Faeces soft) under fasting condition;

Output generated by program 'NIC002_T14_4_3_TEAEs_SocPT_rel_V02_0_0'

Table 14.4.3: Adverse events
Related TEAEs by MedDRA SOC and PT

PART B

MedDRA System organ class<br>Preferred term	Solution 1600mg (N = 4)			Chewing tablet 2000mg (N = 4)			Part B Overall (N = 4)		
	n	(%)	m	n	(%)	m	n	(%)	m
Any	2	(50.0)	3	0	(0.0)	0	2	(50.0)	3
Gastrointestinal disorders	2	(50.0)	3	0	(0.0)	0	2	(50.0)	3
Diarrhoea	1	(25.0)	1	0	(0.0)	0	1	(25.0)	1
Nausea	1	(25.0)	1	0	(0.0)	0	1	(25.0)	1
Vomiting	1	(25.0)	1	0	(0.0)	0	1	(25.0)	1

N: Number of subjects in respective cohort; n: Number of patients with at least one adverse event in respective category; %: Percentage based on N; m: Number of events; TEAE: Treatment-emergent adverse events; Part B was a cross-over design; AEs were allocated to the treatment periods by using the start date of the AEs;

Output generated by program 'NIC002_T14_4_3_TEAEs_SocPT_rel_V02_0_0'

Table 14.4.3: Adverse events
Related TEAEs by MedDRA SOC and PT

PART C

MedDRA System organ class<br>Preferred term	Group 1 (N = 4)			Group 2 (N = 4)			Group 3 (N = 4)			Part C Overall (N = 12)		
	n	(%)	m	n	(%)	m	n	(%)	m	n	(%)	m
Any	4	(100.0)	11	4	(100.0)	10	4	(100.0)	7	12	(100.0)	28
Gastrointestinal disorders	4	(100.0)	8	4	(100.0)	9	4	(100.0)	6	12	(100.0)	23
Diarrhoea	4	(100.0)	4	4	(100.0)	5	3	(75.0)	3	11	(91.7)	12
Nausea	1	(25.0)	1	3	(75.0)	3	0	(0.0)	0	4	(33.3)	4
Flatulence	1	(25.0)	1	0	(0.0)	0	2	(50.0)	2	3	(25.0)	3
Dry mouth	1	(25.0)	1	0	(0.0)	0	0	(0.0)	0	1	(8.3)	1
Faeces soft	0	(0.0)	0	0	(0.0)	0	1	(25.0)	1	1	(8.3)	1
Rectal haemorrhage	1	(25.0)	1	0	(0.0)	0	0	(0.0)	0	1	(8.3)	1
Vomiting	0	(0.0)	0	1	(25.0)	1	0	(0.0)	0	1	(8.3)	1
Nervous system disorders	2	(50.0)	2	1	(25.0)	1	0	(0.0)	0	3	(25.0)	3
Headache	2	(50.0)	2	1	(25.0)	1	0	(0.0)	0	3	(25.0)	3
General disorders and administration site conditions	0	(0.0)	0	0	(0.0)	0	1	(25.0)	1	1	(8.3)	1
Fatigue	0	(0.0)	0	0	(0.0)	0	1	(25.0)	1	1	(8.3)	1
Respiratory, thoracic and mediastinal disorders	1	(25.0)	1	0	(0.0)	0	0	(0.0)	0	1	(8.3)	1
Cough	1	(25.0)	1	0	(0.0)	0	0	(0.0)	0	1	(8.3)	1

N: Number of subjects in respective cohort; n: Number of patients with at least one adverse event in respective category; %: Percentage based on N; m: Number of events; TEAE: Treatment-emergent adverse events; Group 1: 1200 mg niclosamide solution; Group 2: 1600 mg niclosamide solution; Group 3: Placebo;

Output generated by program 'NIC002_T14_4_3_TEAEs_SocPT_rel_V02_0_0'

Table 14.4.4: Adverse events
Deaths and Other Serious Adverse Events

Part A

Subject ID<br>Cohort<br>Treatment	Age [yrs]<br>Sex	MedDRA SOC<br>Preferred Term<br>Investigator Term	Date of onset<br>or worsening<br>Stop date<br>Duration [d]#	Date of first<br>treatment<br>Severity /<br>Rel. to IMP	Seriousness category<br>Outcome<br>AE leading to discontinuation<br>Comment	Time<br>to<br>onset<br>[d]
No serious adverse events occurred during this part of the investigation						

Coding based on MedDRA version 24.0; yrs: Years; #: derived value; AE: Adverse Event; SOC: System Organ Class; IMP: Investigational medical product; Rel.: Relationship;
Duration was calculated as (Stop date) – (Date of onset or worsening) +1; Time to onset was calculated as (Date of onset or worsening) – (Date of first treatment) +1;

Output generated by program 'NIC002_T14_4_4_SAE_Death_V01_0_0'

Table 14.4.4: Adverse events
Deaths and Other Serious Adverse Events

Part B

Subject ID<br>Treatment	Age [yrs]<br>Sex	MedDRA SOC<br>Preferred Term<br>Investigator Term	Date of onset<br>or worsening<br>Stop date<br>Duration [d]#	Date of first<br>treatment<br>Severity /<br>Rel. to IMP	Seriousness category<br>Outcome<br>AE leading to discontinuation<br>Comment	Time<br>to<br>onset<br>[d]
No serious adverse events occurred during this part of the investigation						

Coding based on MedDRA version 24.0; yrs: Years; #: derived value; AE: Adverse Event; SOC: System Organ Class; IMP: Investigational medical product; Rel.: Relationship;
Duration was calculated as (Stop date) – (Date of onset or worsening) +1; Time to onset was calculated as (Date of onset or worsening) – (Date of first treatment) +1;

Output generated by program 'NIC002_T14_4_4_SAE_Death_V01_0_0'

Table 14.4.4: Adverse events
Deaths and Other Serious Adverse Events

Part C

Subject ID<br>Group<br>Treatment	Age [yrs]<br>Sex	MedDRA SOC<br>Preferred Term<br>Investigator Term	Date of onset<br>or worsening<br>Stop date<br>Duration [d]#	Date of first<br>treatment<br>Severity /<br>Rel. to IMP	Seriousness category<br>Outcome<br>AE leading to discontinuation<br>Comment	Time<br>to<br>onset<br>[d]
No serious adverse events occurred during this part of the investigation						

Coding based on MedDRA version 24.0; yrs: Years; #: derived value; AE: Adverse Event; SOC: System Organ Class; IMP: Investigational medical product; Rel.: Relationship;
Duration was calculated as (Stop date) – (Date of onset or worsening) +1; Time to onset was calculated as (Date of onset or worsening) – (Date of first treatment) +1;

Output generated by program 'NIC002_T14_4_4_SAE_Death_V01_0_0'

Table 14.5.1: Safety Laboratory
Clinical Chemistry

Part A

Cohort	Parameter	Visit		n	Miss	Mean	SD	Minimum	Lower quartile	Median	Upper quartile	Maximum
Cohort A1	ALT, 37 °C [U/L]	SC	Raw	3	0	12.33	5.98	7.0	7.00	11.20	18.80	18.8
		BL	Raw	3	0	13.20	4.07	8.9	8.90	13.70	17.00	17.0
		D02	Raw	3	0	14.40	4.71	11.1	11.10	12.30	19.80	19.8
			CFB	3	0	1.20	2.27	-1.4	-1.40	2.20	2.80	2.8
		FUP	Raw	3	0	14.37	3.07	11.9	11.90	13.40	17.80	17.8
			CFB	3	0	1.17	1.68	-0.3	-0.30	0.80	3.00	3.0
	AST, 37 °C [U/L]	SC	Raw	3	0	21.33	5.06	15.5	15.50	24.00	24.50	24.5
		BL	Raw	3	0	21.07	4.55	15.9	15.90	22.80	24.50	24.5
		D02	Raw	3	0	22.77	2.89	21.0	21.00	21.20	26.10	26.1
			CFB	3	0	1.70	3.55	-1.8	-1.80	1.60	5.30	5.3
		FUP	Raw	3	0	21.77	2.72	18.7	18.70	22.70	23.90	23.9
			CFB	3	0	0.70	1.84	-0.6	-0.60	-0.10	2.80	2.8
	Alkaline Phosphatase, 37 °C [U/L]	SC	Raw	3	0	43.40	10.38	32.0	32.00	45.90	52.30	52.3
		BL	Raw	3	0	44.43	9.94	33.3	33.30	47.60	52.40	52.4
		D02	Raw	3	0	44.67	9.85	34.3	34.30	45.80	53.90	53.9
			CFB	3	0	0.23	1.78	-1.8	-1.80	1.00	1.50	1.5
		FUP	Raw	3	0	46.00	9.40	35.4	35.40	49.30	53.30	53.3
			CFB	3	0	1.57	0.61	0.9	0.90	1.70	2.10	2.1
	Bicarbonate [mmol/L]	SC	Raw	3	0	25.97	1.91	24.2	24.20	25.70	28.00	28.0
		BL	Raw	3	0	27.27	2.25	25.1	25.10	27.10	29.60	29.6

n: Number of non-missing observations; %: Percentage based on non-missing observations; Miss: Missing observations; SD: Standard deviation; TP: Timepoint of measurement; SC: Screening; BL: Baseline; FUP: Follow-up; D: Day; Raw: Raw values; CFB: Change from baseline; Cohort A1: 200mg oral dose niclosamide; Cohort A2: 600mg oral dose niclosamide; Cohort A3: 1600mg oral dose niclosamide; Fast/Fed: Treatment in cohort A3 was applied under fasting and fed conditions in the same subjects;

Output generated by program 'NIC002_T14_5_SafetyLaboratory_V02_0_0'

Table 14.5.1: Safety Laboratory
Clinical Chemistry

Part A

Cohort	Parameter	Visit		n	Miss	Mean	SD	Minimum	Lower quartile	Median	Upper quartile	Maximum
	Bicarbonate [mmol/L]	D02	Raw	3	0	26.07	2.32	24.3	24.30	25.20	28.70	28.7
			CFB	3	0	-1.20	1.47	-2.8	-2.80	-0.90	0.10	0.1
		FUP	Raw	3	0	27.23	2.42	25.0	25.00	26.90	29.80	29.8
			CFB	3	0	-0.03	0.21	-0.2	-0.20	-0.10	0.20	0.2
	Bilirubin, total [umol/L]	SC	Raw	3	0	9.23	3.04	6.7	6.70	8.40	12.60	12.6
		BL	Raw	3	0	10.40	3.22	7.0	7.00	10.80	13.40	13.4
		D02	Raw	3	0	9.77	1.59	8.0	8.00	10.20	11.10	11.1
			CFB	3	0	-0.63	4.10	-3.2	-3.20	-2.80	4.10	4.1
		FUP	Raw	3	0	9.13	1.83	7.7	7.70	8.50	11.20	11.2
			CFB	3	0	-1.27	2.44	-3.1	-3.10	-2.20	1.50	1.5
	Calcium [mmol/L]	SC	Raw	3	0	2.297	0.155	2.12	2.120	2.360	2.410	2.41
		BL	Raw	3	0	2.327	0.101	2.21	2.210	2.380	2.390	2.39
		D02	Raw	3	0	2.323	0.125	2.18	2.180	2.380	2.410	2.41
			CFB	3	0	-0.003	0.031	-0.03	-0.030	-0.010	0.030	0.03
		FUP	Raw	3	0	2.337	0.127	2.19	2.190	2.400	2.420	2.42
			CFB	3	0	0.010	0.026	-0.02	-0.020	0.020	0.030	0.03
	Creatinine Clearance MDRD [ml/min/1.73m]	SC	Raw	3	0	107.7	12.0	96	96.0	107.0	120.0	120
	Creatinine [umol/L]	SC	Raw	3	0	58.13	5.95	54.4	54.40	55.00	65.00	65.0
		BL	Raw	3	0	55.30	9.62	49.5	49.50	50.00	66.40	66.4

n: Number of non-missing observations; %: Percentage based on non-missing observations; Miss: Missing observations; SD: Standard deviation; TP: Timepoint of measurement; SC: Screening; BL: Baseline; FUP: Follow-up; D: Day; Raw: Raw values; CFB: Change from baseline; Cohort A1: 200mg oral dose niclosamide; Cohort A2: 600mg oral dose niclosamide; Cohort A3: 1600mg oral dose niclosamide; Fast/Fed: Treatment in cohort A3 was applied under fasting and fed conditions in the same subjects;

Output generated by program 'NIC002_T14_5_SafetyLaboratory_V02_0_0'

Table 14.5.1: Safety Laboratory
Clinical Chemistry

Part A

Cohort	Parameter	Visit		n	Miss	Mean	SD	Minimum	Lower quartile	Median	Upper quartile	Maximum
	Creatinine [umol/L]	D02	Raw	3	0	58.13	7.42	53.7	53.70	54.00	66.70	66.7
			CFB	3	0	2.83	2.20	0.3	0.30	4.00	4.20	4.2
		FUP	Raw	3	0	53.87	8.61	47.7	47.70	50.20	63.70	63.7
			CFB	3	0	-1.43	1.86	-2.7	-2.70	-2.30	0.70	0.7
	Gamma-GT, 37 °C [U/L]	SC	Raw	3	0	10.63	3.42	7.0	7.00	11.10	13.80	13.8
		BL	Raw	3	0	10.53	2.99	7.3	7.30	11.10	13.20	13.2
		D02	Raw	3	0	10.10	3.41	6.2	6.20	11.60	12.50	12.5
			CFB	3	0	-0.43	0.83	-1.1	-1.10	-0.70	0.50	0.5
		FUP	Raw	3	0	10.87	3.12	7.3	7.30	12.20	13.10	13.1
			CFB	3	0	0.33	0.67	-0.1	-0.10	0.00	1.10	1.1
	Glucose, serum [mmol/L]	SC	Raw	3	0	4.970	0.348	4.57	4.570	5.140	5.200	5.20
		BL	Raw	3	0	4.977	0.157	4.80	4.800	5.030	5.100	5.10
		D02	Raw	3	0	4.750	0.135	4.61	4.610	4.760	4.880	4.88
			CFB	3	0	-0.227	0.190	-0.42	-0.420	-0.220	-0.040	-0.04
		FUP	Raw	3	0	4.427	0.221	4.22	4.220	4.400	4.660	4.66
			CFB	3	0	-0.550	0.226	-0.81	-0.810	-0.440	-0.400	-0.40
	Magnesium [mmol/L]	SC	Raw	3	0	0.817	0.012	0.81	0.810	0.810	0.830	0.83
		BL	Raw	3	0	0.813	0.023	0.80	0.800	0.800	0.840	0.84
		D02	Raw	3	0	0.803	0.029	0.77	0.770	0.820	0.820	0.82
			CFB	3	0	-0.010	0.026	-0.03	-0.030	-0.020	0.020	0.02
		FUP	Raw	3	0	0.820	0.044	0.79	0.790	0.800	0.870	0.87
			CFB	3	0	0.007	0.021	-0.01	-0.010	0.000	0.030	0.03

n: Number of non-missing observations; %: Percentage based on non-missing observations; Miss: Missing observations; SD: Standard deviation; TP: Timepoint of measurement; SC: Screening; BL: Baseline; FUP: Follow-up; D: Day; Raw: Raw values; CFB: Change from baseline; Cohort A1: 200mg oral dose niclosamide; Cohort A2: 600mg oral dose niclosamide; Cohort A3: 1600mg oral dose niclosamide; Fast/Fed: Treatment in cohort A3 was applied under fasting and fed conditions in the same subjects;

Output generated by program 'NIC002_T14_5_SafetyLaboratory_V02_0_0'

Table 14.5.1: Safety Laboratory
Clinical Chemistry

Part A

Cohort	Parameter	Visit		n	Miss	Mean	SD	Minimum	Lower quartile	Median	Upper quartile	Maximum
	Potassium [mmol/L]	SC	Raw	3	0	3.900	0.289	3.69	3.690	3.780	4.230	4.23
		BL	Raw	3	0	3.863	0.021	3.84	3.840	3.870	3.880	3.88
		D02	Raw	3	0	3.917	0.190	3.72	3.720	3.930	4.100	4.10
			CFB	3	0	0.053	0.170	-0.12	-0.120	0.060	0.220	0.22
		FUP	Raw	3	0	4.030	0.277	3.86	3.860	3.880	4.350	4.35
			CFB	3	0	0.167	0.297	-0.01	-0.010	0.000	0.510	0.51
	Sodium [mmol/L]	SC	Raw	3	0	138.23	0.93	137.6	137.60	137.80	139.30	139.3
		BL	Raw	3	0	139.50	0.95	138.6	138.60	139.40	140.50	140.5
		D02	Raw	3	0	138.23	0.71	137.6	137.60	138.10	139.00	139.0
			CFB	3	0	-1.27	1.42	-2.9	-2.90	-0.50	-0.40	-0.4
		FUP	Raw	3	0	138.43	0.86	137.5	137.50	138.60	139.20	139.2
			CFB	3	0	-1.07	0.25	-1.3	-1.30	-1.10	-0.80	-0.8
	Urea/BUN [mmol/L]	SC	Raw	3	0	3.400	0.546	2.77	2.770	3.690	3.740	3.74
		BL	Raw	3	0	3.507	0.853	2.58	2.580	3.680	4.260	4.26
		D02	Raw	3	0	4.117	0.298	3.92	3.920	3.970	4.460	4.46
			CFB	3	0	0.610	0.676	0.20	0.200	0.240	1.390	1.39
		FUP	Raw	3	0	3.327	0.440	2.89	2.890	3.320	3.770	3.77
			CFB	3	0	-0.180	1.081	-1.37	-1.370	0.090	0.740	0.74
Cohort A2	ALT, 37 °C [U/L]	SC	Raw	3	0	16.57	3.27	13.5	13.50	16.20	20.00	20.0
		BL	Raw	3	0	14.63	5.50	8.3	8.30	17.40	18.20	18.2
		D02	Raw	3	0	12.60	4.26	7.7	7.70	14.70	15.40	15.4
			CFB	3	0	-2.03	1.24	-2.8	-2.80	-2.70	-0.60	-0.6

n: Number of non-missing observations; %: Percentage based on non-missing observations; Miss: Missing observations; SD: Standard deviation; TP: Timepoint of measurement; SC: Screening; BL: Baseline; FUP: Follow-up; D: Day; Raw: Raw values; CFB: Change from baseline; Cohort A1: 200mg oral dose niclosamide; Cohort A2: 600mg oral dose niclosamide; Cohort A3: 1600mg oral dose niclosamide; Fast/Fed: Treatment in cohort A3 was applied under fasting and fed conditions in the same subjects;

Output generated by program 'NIC002_T14_5_SafetyLaboratory_V02_0_0'

Table 14.5.1: Safety Laboratory
Clinical Chemistry

Part A

Cohort	Parameter	Visit		n	Miss	Mean	SD	Minimum	Lower quartile	Median	Upper quartile	Maximum
Cohort A2	ALT, 37 °C [U/L]	FUP	Raw	3	0	14.73	4.83	9.4	9.40	16.00	18.80	18.8
			CFB	3	0	0.10	1.32	-1.4	-1.40	0.60	1.10	1.1
	AST, 37 °C [U/L]	SC	Raw	3	0	24.03	5.12	20.0	20.00	22.30	29.80	29.8
		BL	Raw	3	0	21.40	4.75	17.3	17.30	20.30	26.60	26.6
		D02	Raw	3	0	20.63	2.67	17.6	17.60	21.70	22.60	22.6
			CFB	3	0	-0.77	2.85	-4.0	-4.00	0.30	1.40	1.4
		FUP	Raw	3	0	25.43	6.77	19.3	19.30	24.30	32.70	32.7
			CFB	3	0	4.03	2.05	2.0	2.00	4.00	6.10	6.1
	Alkaline Phosphatase, 37 °C [U/L]	SC	Raw	3	0	55.17	10.32	45.2	45.20	54.50	65.80	65.8
		BL	Raw	3	0	53.43	4.59	50.3	50.30	51.30	58.70	58.7
		D02	Raw	3	0	52.40	5.88	48.1	48.10	50.00	59.10	59.1
			CFB	3	0	-1.03	1.91	-3.2	-3.20	-0.30	0.40	0.4
		FUP	Raw	3	0	54.53	3.54	50.5	50.50	56.00	57.10	57.1
			CFB	3	0	1.10	4.00	-1.6	-1.60	-0.80	5.70	5.7
	Bicarbonate [mmol/L]	SC	Raw	3	0	25.30	2.46	23.5	23.50	24.30	28.10	28.1
		BL	Raw	3	0	25.60	0.10	25.5	25.50	25.60	25.70	25.7
		D02	Raw	3	0	24.13	1.72	22.6	22.60	23.80	26.00	26.0
			CFB	3	0	-1.47	1.69	-2.9	-2.90	-1.90	0.40	0.4
		FUP	Raw	3	0	24.10	1.11	23.1	23.10	23.90	25.30	25.3
			CFB	3	0	-1.50	1.05	-2.5	-2.50	-1.60	-0.40	-0.4

n: Number of non-missing observations; %: Percentage based on non-missing observations; Miss: Missing observations; SD: Standard deviation; TP: Timepoint of measurement; SC: Screening; BL: Baseline; FUP: Follow-up; D: Day; Raw: Raw values; CFB: Change from baseline; Cohort A1: 200mg oral dose niclosamide; Cohort A2: 600mg oral dose niclosamide; Cohort A3: 1600mg oral dose niclosamide; Fast/Fed: Treatment in cohort A3 was applied under fasting and fed conditions in the same subjects;

Output generated by program 'NIC002_T14_5_SafetyLaboratory_V02_0_0'

Table 14.5.1: Safety Laboratory
Clinical Chemistry

Part A

Cohort	Parameter	Visit		n	Miss	Mean	SD	Minimum	Lower quartile	Median	Upper quartile	Maximum
	Bilirubin, total [umol/L]	SC	Raw	3	0	13.47	2.12	11.1	11.10	14.10	15.20	15.2
		BL	Raw	3	0	11.33	6.08	6.1	6.10	9.90	18.00	18.0
		D02	Raw	3	0	11.63	2.45	9.2	9.20	11.60	14.10	14.1
			CFB	3	0	0.30	5.83	-6.4	-6.40	3.10	4.20	4.2
		FUP	Raw	3	0	10.20	4.16	5.4	5.40	12.50	12.70	12.7
			CFB	3	0	-1.13	3.97	-5.3	-5.30	-0.70	2.60	2.6
	Calcium [mmol/L]	SC	Raw	3	0	2.377	0.012	2.37	2.370	2.370	2.390	2.39
		BL	Raw	3	0	2.380	0.062	2.33	2.330	2.360	2.450	2.45
		D02	Raw	3	0	2.357	0.038	2.33	2.330	2.340	2.400	2.40
			CFB	3	0	-0.023	0.025	-0.05	-0.050	-0.020	0.000	0.00
		FUP	Raw	3	0	2.307	0.072	2.26	2.260	2.270	2.390	2.39
			CFB	3	0	-0.073	0.015	-0.09	-0.090	-0.070	-0.060	-0.06
	Creatinine Clearance MDRD [ml/min/1.73m]	SC	Raw	3	0	100.0	15.6	90	90.0	92.0	118.0	118
	Creatinine [umol/L]	SC	Raw	3	0	62.57	7.77	53.6	53.60	67.00	67.10	67.1
		BL	Raw	3	0	61.27	9.19	50.7	50.70	65.70	67.40	67.4
		D02	Raw	3	0	60.43	10.00	49.6	49.60	62.40	69.30	69.3
			CFB	3	0	-0.83	2.61	-3.3	-3.30	-1.10	1.90	1.9
		FUP	Raw	3	0	64.17	14.56	48.2	48.20	67.60	76.70	76.7
			CFB	3	0	2.90	7.14	-2.5	-2.50	0.20	11.00	11.0

n: Number of non-missing observations; %: Percentage based on non-missing observations; Miss: Missing observations; SD: Standard deviation; TP: Timepoint of measurement; SC: Screening; BL: Baseline; FUP: Follow-up; D: Day; Raw: Raw values; CFB: Change from baseline; Cohort A1: 200mg oral dose niclosamide; Cohort A2: 600mg oral dose niclosamide; Cohort A3: 1600mg oral dose niclosamide; Fast/Fed: Treatment in cohort A3 was applied under fasting and fed conditions in the same subjects;

Output generated by program 'NIC002_T14_5_SafetyLaboratory_V02_0_0'

Table 14.5.1: Safety Laboratory
Clinical Chemistry

Part A

Cohort	Parameter	Visit		n	Miss	Mean	SD	Minimum	Lower quartile	Median	Upper quartile	Maximum
	Gamma-GT, 37 °C [U/L]	SC	Raw	3	0	12.17	2.18	10.4	10.40	11.50	14.60	14.6
		BL	Raw	3	0	12.07	3.31	8.9	8.90	11.80	15.50	15.5
		D02	Raw	3	0	11.67	3.56	8.3	8.30	11.30	15.40	15.4
			CFB	3	0	-0.40	0.26	-0.6	-0.60	-0.50	-0.10	-0.1
		FUP	Raw	3	0	11.10	3.89	8.1	8.10	9.70	15.50	15.5
			CFB	3	0	-0.97	1.06	-2.1	-2.10	-0.80	0.00	0.0
	Glucose, serum [mmol/L]	SC	Raw	3	0	4.683	0.146	4.55	4.550	4.660	4.840	4.84
		BL	Raw	3	0	4.873	0.393	4.43	4.430	5.010	5.180	5.18
		D02	Raw	3	0	4.503	0.237	4.23	4.230	4.630	4.650	4.65
			CFB	3	0	-0.370	0.165	-0.53	-0.530	-0.380	-0.200	-0.20
		FUP	Raw	3	0	4.503	0.217	4.34	4.340	4.420	4.750	4.75
			CFB	3	0	-0.370	0.599	-0.76	-0.760	-0.670	0.320	0.32
	Magnesium [mmol/L]	SC	Raw	3	0	0.813	0.023	0.80	0.800	0.800	0.840	0.84
		BL	Raw	3	0	0.813	0.035	0.78	0.780	0.810	0.850	0.85
		D02	Raw	3	0	0.783	0.012	0.77	0.770	0.790	0.790	0.79
			CFB	3	0	-0.030	0.046	-0.08	-0.080	-0.020	0.010	0.01
		FUP	Raw	3	0	0.833	0.038	0.79	0.790	0.850	0.860	0.86
			CFB	3	0	0.020	0.026	0.00	0.000	0.010	0.050	0.05
	Potassium [mmol/L]	SC	Raw	3	0	4.400	0.271	4.12	4.120	4.420	4.660	4.66
		BL	Raw	3	0	4.080	0.267	3.87	3.870	3.990	4.380	4.38
		D02	Raw	3	0	4.077	0.105	3.97	3.970	4.080	4.180	4.18
			CFB	3	0	-0.003	0.369	-0.41	-0.410	0.090	0.310	0.31

n: Number of non-missing observations; %: Percentage based on non-missing observations; Miss: Missing observations; SD: Standard deviation; TP: Timepoint of measurement; SC: Screening; BL: Baseline; FUP: Follow-up; D: Day; Raw: Raw values; CFB: Change from baseline; Cohort A1: 200mg oral dose niclosamide; Cohort A2: 600mg oral dose niclosamide; Cohort A3: 1600mg oral dose niclosamide; Fast/Fed: Treatment in cohort A3 was applied under fasting and fed conditions in the same subjects;

Output generated by program 'NIC002_T14_5_SafetyLaboratory_V02_0_0'

Table 14.5.1: Safety Laboratory
Clinical Chemistry

Part A

Cohort	Parameter	Visit		n	Miss	Mean	SD	Minimum	Lower quartile	Median	Upper quartile	Maximum
	Potassium [mmol/L]	FUP	Raw	3	0	4.140	0.106	4.02	4.020	4.180	4.220	4.22
			CFB	3	0	0.060	0.236	-0.16	-0.160	0.030	0.310	0.31
	Sodium [mmol/L]	SC	Raw	3	0	137.97	1.19	136.6	136.60	138.50	138.80	138.8
		BL	Raw	3	0	139.07	1.10	137.8	137.80	139.70	139.70	139.7
		D02	Raw	3	0	136.97	0.91	136.0	136.00	137.10	137.80	137.8
			CFB	3	0	-2.10	0.44	-2.6	-2.60	-1.90	-1.80	-1.8
		FUP	Raw	3	0	138.20	0.70	137.5	137.50	138.20	138.90	138.9
			CFB	3	0	-0.87	0.60	-1.5	-1.50	-0.80	-0.30	-0.3
	Urea/BUN [mmol/L]	SC	Raw	3	0	3.173	0.296	2.85	2.850	3.240	3.430	3.43
		BL	Raw	3	0	3.720	1.022	3.10	3.100	3.160	4.900	4.90
		D02	Raw	3	0	3.313	0.302	3.11	3.110	3.170	3.660	3.66
			CFB	3	0	-0.407	1.217	-1.79	-1.790	0.070	0.500	0.50
		FUP	Raw	3	0	3.457	0.743	2.95	2.950	3.110	4.310	4.31
			CFB	3	0	-0.263	1.568	-1.95	-1.950	0.010	1.150	1.15
Cohort A3 Fast	ALT, 37 °C [U/L]	SC	Raw	3	0	11.07	2.12	9.6	9.60	10.10	13.50	13.5
		BL	Raw	3	0	12.73	2.74	10.3	10.30	12.20	15.70	15.7
		D02	Raw	3	0	11.27	1.10	10.0	10.00	11.80	12.00	12.0
			CFB	3	0	-1.47	1.93	-3.7	-3.70	-0.40	-0.30	-0.3
		FUP	Raw	3	0	11.60	1.22	10.8	10.80	11.00	13.00	13.0
			CFB	3	0	-1.13	3.80	-4.9	-4.90	-1.20	2.70	2.7
	AST, 37 °C [U/L]	SC	Raw	3	0	20.23	4.01	15.8	15.80	21.30	23.60	23.6

n: Number of non-missing observations; %: Percentage based on non-missing observations; Miss: Missing observations; SD: Standard deviation; TP: Timepoint of measurement; SC: Screening; BL: Baseline; FUP: Follow-up; D: Day; Raw: Raw values; CFB: Change from baseline; Cohort A1: 200mg oral dose niclosamide; Cohort A2: 600mg oral dose niclosamide; Cohort A3: 1600mg oral dose niclosamide; Fast/Fed: Treatment in cohort A3 was applied under fasting and fed conditions in the same subjects;

Output generated by program 'NIC002_T14_5_SafetyLaboratory_V02_0_0'

Table 14.5.1: Safety Laboratory
Clinical Chemistry

Part A

Cohort	Parameter	Visit		n	Miss	Mean	SD	Minimum	Lower quartile	Median	Upper quartile	Maximum
	AST, 37 °C [U/L]	BL	Raw	3	0	21.07	2.68	19.0	19.00	20.10	24.10	24.1
		D02	Raw	3	0	20.60	5.73	15.7	15.70	19.20	26.90	26.9
			CFB	3	0	-0.47	3.07	-3.3	-3.30	-0.90	2.80	2.8
		FUP	Raw	3	0	20.37	3.67	16.2	16.20	21.80	23.10	23.1
			CFB	3	0	-0.70	2.26	-2.8	-2.80	-1.00	1.70	1.7
	Alkaline Phosphatase, 37 °C [U/L]	SC	Raw	3	0	51.07	9.16	41.2	41.20	52.70	59.30	59.3
		BL	Raw	3	0	53.93	8.19	45.0	45.00	55.70	61.10	61.1
		D02	Raw	3	0	60.23	7.66	51.4	51.40	64.30	65.00	65.0
			CFB	3	0	6.30	2.35	3.9	3.90	6.40	8.60	8.6
		FUP	Raw	3	0	56.27	5.34	50.1	50.10	59.20	59.50	59.5
			CFB	3	0	2.33	3.72	-1.9	-1.90	3.80	5.10	5.1
	Bicarbonate [mmol/L]	SC	Raw	3	0	27.00	1.65	25.9	25.90	26.20	28.90	28.9
		BL	Raw	3	0	25.70	1.77	24.1	24.10	25.40	27.60	27.6
		D02	Raw	3	0	26.53	2.25	24.9	24.90	25.60	29.10	29.1
			CFB	3	0	0.83	3.69	-2.0	-2.00	-0.50	5.00	5.0
		FUP	Raw	3	0	27.07	0.57	26.6	26.60	26.90	27.70	27.7
			CFB	3	0	1.37	1.36	0.1	0.10	1.20	2.80	2.8
	Bilirubin, total [umol/L]	SC	Raw	3	0	9.67	2.98	7.7	7.70	8.20	13.10	13.1
		BL	Raw	3	0	10.10	3.63	7.3	7.30	8.80	14.20	14.2
		D02	Raw	3	0	7.70	1.82	6.6	6.60	6.70	9.80	9.8
			CFB	3	0	-2.40	4.57	-7.6	-7.60	-0.60	1.00	1.0
		FUP	Raw	3	0	8.90	3.46	6.9	6.90	6.90	12.90	12.9

n: Number of non-missing observations; %: Percentage based on non-missing observations; Miss: Missing observations; SD: Standard deviation; TP: Timepoint of measurement; SC: Screening; BL: Baseline; FUP: Follow-up; D: Day; Raw: Raw values; CFB: Change from baseline; Cohort A1: 200mg oral dose niclosamide; Cohort A2: 600mg oral dose niclosamide; Cohort A3: 1600mg oral dose niclosamide; Fast/Fed: Treatment in cohort A3 was applied under fasting and fed conditions in the same subjects;

Output generated by program 'NIC002_T14_5_SafetyLaboratory_V02_0_0'

Table 14.5.1: Safety Laboratory
Clinical Chemistry

Part A

Cohort	Parameter	Visit		n	Miss	Mean	SD	Minimum	Lower quartile	Median	Upper quartile	Maximum
	Bilirubin, total [umol/L]	FUP	CFB	3	0	-1.20	5.74	-7.3	-7.30	-0.40	4.10	4.1
	Calcium [mmol/L]	SC	Raw	3	0	2.383	0.021	2.36	2.360	2.390	2.400	2.40
		BL	Raw	3	0	2.407	0.112	2.31	2.310	2.380	2.530	2.53
		D02	Raw	3	0	2.427	0.055	2.39	2.390	2.400	2.490	2.49
			CFB	3	0	0.020	0.155	-0.13	-0.130	0.010	0.180	0.18
		FUP	Raw	3	0	2.350	0.026	2.33	2.330	2.340	2.380	2.38
			CFB	3	0	-0.057	0.130	-0.19	-0.190	-0.050	0.070	0.07
	Creatinine Clearance MDRD [ml/min/1.73m]	SC	Raw	3	0	106.3	13.3	91	91.0	113.0	115.0	115
	Creatinine [umol/L]	SC	Raw	3	0	60.93	6.37	56.3	56.30	58.30	68.20	68.2
		BL	Raw	3	0	66.93	16.56	56.2	56.20	58.60	86.00	86.0
		D02	Raw	3	0	64.33	11.79	55.4	55.40	59.90	77.70	77.7
			CFB	3	0	-2.60	5.05	-8.3	-8.30	-0.80	1.30	1.3
		FUP	Raw	3	0	62.27	8.54	56.7	56.70	58.00	72.10	72.1
			CFB	3	0	-4.67	8.21	-13.9	-13.90	-1.90	1.80	1.8
	Gamma-GT, 37 °C [U/L]	SC	Raw	3	0	11.67	4.60	7.9	7.90	10.30	16.80	16.8
		BL	Raw	3	0	12.30	4.16	8.8	8.80	11.20	16.90	16.9
		D02	Raw	3	0	13.40	6.64	8.7	8.70	10.50	21.00	21.0
			CFB	3	0	1.10	2.62	-0.7	-0.70	-0.10	4.10	4.1
		FUP	Raw	3	0	12.70	5.63	8.5	8.50	10.50	19.10	19.1

n: Number of non-missing observations; %: Percentage based on non-missing observations; Miss: Missing observations; SD: Standard deviation; TP: Timepoint of measurement; SC: Screening; BL: Baseline; FUP: Follow-up; D: Day; Raw: Raw values; CFB: Change from baseline; Cohort A1: 200mg oral dose niclosamide; Cohort A2: 600mg oral dose niclosamide; Cohort A3: 1600mg oral dose niclosamide; Fast/Fed: Treatment in cohort A3 was applied under fasting and fed conditions in the same subjects;

Output generated by program 'NIC002_T14_5_SafetyLaboratory_V02_0_0'

Table 14.5.1: Safety Laboratory
Clinical Chemistry

Part A

Cohort	Parameter	Visit		n	Miss	Mean	SD	Minimum	Lower quartile	Median	Upper quartile	Maximum
	Gamma-GT, 37 °C [U/L]	FUP	CFB	3	0	0.40	1.57	-0.7	-0.70	-0.30	2.20	2.2
	Glucose, serum [mmol/L]	SC	Raw	3	0	4.733	0.486	4.39	4.390	4.520	5.290	5.29
		BL	Raw	3	0	4.680	0.504	4.13	4.130	4.790	5.120	5.12
		D02	Raw	3	0	4.753	0.428	4.26	4.260	4.980	5.020	5.02
			CFB	3	0	0.073	0.153	-0.10	-0.100	0.130	0.190	0.19
		FUP	Raw	3	0	4.437	0.119	4.30	4.300	4.490	4.520	4.52
			CFB	3	0	-0.243	0.388	-0.60	-0.600	-0.300	0.170	0.17
	Magnesium [mmol/L]	SC	Raw	3	0	0.750	0.030	0.72	0.720	0.750	0.780	0.78
		BL	Raw	3	0	0.760	0.040	0.72	0.720	0.760	0.800	0.80
		D02	Raw	3	0	0.790	0.080	0.71	0.710	0.790	0.870	0.87
			CFB	3	0	0.030	0.106	-0.05	-0.050	-0.010	0.150	0.15
		FUP	Raw	3	0	0.763	0.055	0.70	0.700	0.790	0.800	0.80
			CFB	3	0	0.003	0.065	-0.06	-0.060	0.000	0.070	0.07
	Potassium [mmol/L]	SC	Raw	3	0	3.963	0.264	3.66	3.660	4.090	4.140	4.14
		BL	Raw	3	0	4.363	0.118	4.29	4.290	4.300	4.500	4.50
		D02	Raw	3	0	4.337	0.111	4.22	4.220	4.350	4.440	4.44
			CFB	3	0	-0.027	0.225	-0.28	-0.280	0.050	0.150	0.15
		FUP	Raw	3	0	4.163	0.211	3.94	3.940	4.190	4.360	4.36
			CFB	3	0	-0.200	0.322	-0.56	-0.560	-0.100	0.060	0.06
	Sodium [mmol/L]	SC	Raw	3	0	137.93	1.23	136.9	136.90	137.60	139.30	139.3
		BL	Raw	3	0	137.67	2.14	135.8	135.80	137.20	140.00	140.0

n: Number of non-missing observations; %: Percentage based on non-missing observations; Miss: Missing observations; SD: Standard deviation; TP: Timepoint of measurement; SC: Screening; BL: Baseline; FUP: Follow-up; D: Day; Raw: Raw values; CFB: Change from baseline; Cohort A1: 200mg oral dose niclosamide; Cohort A2: 600mg oral dose niclosamide; Cohort A3: 1600mg oral dose niclosamide; Fast/Fed: Treatment in cohort A3 was applied under fasting and fed conditions in the same subjects;

Output generated by program 'NIC002_T14_5_SafetyLaboratory_V02_0_0'

Table 14.5.1: Safety Laboratory
Clinical Chemistry

Part A

Cohort	Parameter	Visit		n	Miss	Mean	SD	Minimum	Lower quartile	Median	Upper quartile	Maximum
	Sodium [mmol/L]	D02	Raw	3	0	138.30	1.76	137.0	137.00	137.60	140.30	140.3
			CFB	3	0	0.63	3.52	-2.4	-2.40	-0.20	4.50	4.5
		FUP	Raw	3	0	137.07	0.58	136.4	136.40	137.40	137.40	137.4
			CFB	3	0	-0.60	2.11	-2.6	-2.60	-0.80	1.60	1.6
	Urea/BUN [mmol/L]	SC	Raw	3	0	3.287	0.921	2.43	2.430	3.170	4.260	4.26
		BL	Raw	3	0	3.753	1.800	1.82	1.820	4.060	5.380	5.38
		D02	Raw	3	0	3.363	0.964	2.27	2.270	3.730	4.090	4.09
			CFB	3	0	-0.390	0.872	-1.29	-1.290	-0.330	0.450	0.45
		FUP	Raw	3	0	3.803	1.239	2.51	2.510	3.920	4.980	4.98
			CFB	3	0	0.050	0.569	-0.40	-0.400	-0.140	0.690	0.69
Cohort A3 Fed	ALT, 37 °C [U/L]	BL	Raw	3	0	13.13	3.27	9.4	9.40	14.50	15.50	15.5
		D02	Raw	3	0	12.63	1.91	10.5	10.50	13.20	14.20	14.2
			CFB	3	0	-0.50	1.39	-1.3	-1.30	-1.30	1.10	1.1
		FUP	Raw	3	0	14.83	2.44	12.1	12.10	15.60	16.80	16.8
			CFB	3	0	1.70	0.87	1.1	1.10	1.30	2.70	2.7
	AST, 37 °C [U/L]	BL	Raw	3	0	21.00	1.04	19.8	19.80	21.50	21.70	21.7
		D02	Raw	3	0	19.50	2.26	17.6	17.60	18.90	22.00	22.0
			CFB	3	0	-1.50	1.76	-2.8	-2.80	-2.20	0.50	0.5
		FUP	Raw	3	0	23.17	1.68	21.7	21.70	22.80	25.00	25.0
			CFB	3	0	2.17	1.22	1.1	1.10	1.90	3.50	3.5

n: Number of non-missing observations; %: Percentage based on non-missing observations; Miss: Missing observations; SD: Standard deviation; TP: Timepoint of measurement; SC: Screening; BL: Baseline; FUP: Follow-up; D: Day; Raw: Raw values; CFB: Change from baseline; Cohort A1: 200mg oral dose niclosamide; Cohort A2: 600mg oral dose niclosamide; Cohort A3: 1600mg oral dose niclosamide; Fast/Fed: Treatment in cohort A3 was applied under fasting and fed conditions in the same subjects;

Output generated by program 'NIC002_T14_5_SafetyLaboratory_V02_0_0'

Table 14.5.1: Safety Laboratory
Clinical Chemistry

Part A

Cohort	Parameter	Visit		n	Miss	Mean	SD	Minimum	Lower quartile	Median	Upper quartile	Maximum
	Alkaline Phosphatase, 37 °C [U/L]	BL	Raw	3	0	58.03	14.33	45.8	45.80	54.50	73.80	73.8
		D02	Raw	3	0	55.07	12.25	46.0	46.00	50.20	69.00	69.0
			CFB	3	0	-2.97	2.75	-4.8	-4.80	-4.30	0.20	0.2
		FUP	Raw	3	0	60.90	11.23	52.7	52.70	56.30	73.70	73.7
			CFB	3	0	2.87	3.62	-0.1	-0.10	1.80	6.90	6.9
	Bicarbonate [mmol/L]	BL	Raw	3	0	26.10	2.86	23.1	23.10	26.40	28.80	28.8
		D02	Raw	3	0	24.40	1.71	23.0	23.00	23.90	26.30	26.3
			CFB	3	0	-1.70	1.39	-2.5	-2.50	-2.50	-0.10	-0.1
		FUP	Raw	3	0	26.63	0.92	26.1	26.10	26.10	27.70	27.7
			CFB	3	0	0.53	2.17	-1.1	-1.10	-0.30	3.00	3.0
	Bilirubin, total [umol/L]	BL	Raw	3	0	8.90	2.61	7.2	7.20	7.60	11.90	11.9
		D02	Raw	3	0	8.97	0.93	8.2	8.20	8.70	10.00	10.0
			CFB	3	0	0.07	1.70	-1.9	-1.90	1.00	1.10	1.1
		FUP	Raw	3	0	11.20	2.84	8.0	8.00	12.20	13.40	13.4
			CFB	3	0	2.30	2.02	0.8	0.80	1.50	4.60	4.6
	Calcium [mmol/L]	BL	Raw	3	0	2.380	0.106	2.30	2.300	2.340	2.500	2.50
		D02	Raw	3	0	2.383	0.086	2.29	2.290	2.400	2.460	2.46
			CFB	3	0	0.003	0.051	-0.04	-0.040	-0.010	0.060	0.06
		FUP	Raw	3	0	2.387	0.093	2.28	2.280	2.430	2.450	2.45
			CFB	3	0	0.007	0.093	-0.07	-0.070	-0.020	0.110	0.11

n: Number of non-missing observations; %: Percentage based on non-missing observations; Miss: Missing observations; SD: Standard deviation; TP: Timepoint of measurement; SC: Screening; BL: Baseline; FUP: Follow-up; D: Day; Raw: Raw values; CFB: Change from baseline; Cohort A1: 200mg oral dose niclosamide; Cohort A2: 600mg oral dose niclosamide; Cohort A3: 1600mg oral dose niclosamide; Fast/Fed: Treatment in cohort A3 was applied under fasting and fed conditions in the same subjects;

Output generated by program 'NIC002_T14_5_SafetyLaboratory_V02_0_0'

Table 14.5.1: Safety Laboratory
Clinical Chemistry

Part A

Cohort	Parameter	Visit		n	Miss	Mean	SD	Minimum	Lower quartile	Median	Upper quartile	Maximum
	Creatinine [umol/L]	BL	Raw	3	0	63.43	7.05	56.9	56.90	62.50	70.90	70.9
		D02	Raw	3	0	62.30	10.84	55.4	55.40	56.70	74.80	74.8
			CFB	3	0	-1.13	5.56	-7.1	-7.10	-0.20	3.90	3.9
		FUP	Raw	3	0	61.80	8.14	55.2	55.20	59.30	70.90	70.9
			CFB	3	0	-1.63	1.60	-3.2	-3.20	-1.70	0.00	0.0
	Gamma-GT, 37 °C [U/L]	BL	Raw	3	0	11.83	5.11	6.9	6.90	11.50	17.10	17.1
		D02	Raw	3	0	11.90	4.86	7.2	7.20	11.60	16.90	16.9
			CFB	3	0	0.07	0.25	-0.2	-0.20	0.10	0.30	0.3
		FUP	Raw	3	0	11.90	5.38	6.9	6.90	11.20	17.60	17.6
			CFB	3	0	0.07	0.40	-0.3	-0.30	0.00	0.50	0.5
	Glucose, serum [mmol/L]	BL	Raw	3	0	4.517	0.352	4.19	4.190	4.470	4.890	4.89
		D02	Raw	3	0	4.510	0.443	4.04	4.040	4.570	4.920	4.92
			CFB	3	0	-0.007	0.129	-0.15	-0.150	0.030	0.100	0.10
		FUP	Raw	3	0	4.477	0.257	4.18	4.180	4.620	4.630	4.63
			CFB	3	0	-0.040	0.217	-0.27	-0.270	-0.010	0.160	0.16
	Magnesium [mmol/L]	BL	Raw	3	0	0.770	0.026	0.75	0.750	0.760	0.800	0.80
		D02	Raw	3	0	0.763	0.068	0.71	0.710	0.740	0.840	0.84
			CFB	3	0	-0.007	0.042	-0.04	-0.040	-0.020	0.040	0.04
		FUP	Raw	3	0	0.740	0.052	0.68	0.680	0.770	0.770	0.77
			CFB	3	0	-0.030	0.040	-0.07	-0.070	-0.030	0.010	0.01

n: Number of non-missing observations; %: Percentage based on non-missing observations; Miss: Missing observations; SD: Standard deviation; TP: Timepoint of measurement; SC: Screening; BL: Baseline; FUP: Follow-up; D: Day; Raw: Raw values; CFB: Change from baseline; Cohort A1: 200mg oral dose niclosamide; Cohort A2: 600mg oral dose niclosamide; Cohort A3: 1600mg oral dose niclosamide; Fast/Fed: Treatment in cohort A3 was applied under fasting and fed conditions in the same subjects;

Output generated by program 'NIC002_T14_5_SafetyLaboratory_V02_0_0'

Table 14.5.1: Safety Laboratory
Clinical Chemistry

Part A

Cohort	Parameter	Visit		n	Miss	Mean	SD	Minimum	Lower quartile	Median	Upper quartile	Maximum
	Potassium [mmol/L]	BL	Raw	3	0	3.963	0.189	3.80	3.800	3.920	4.170	4.17
		D02	Raw	3	0	4.357	0.236	4.09	4.090	4.440	4.540	4.54
			CFB	3	0	0.393	0.424	-0.08	-0.080	0.520	0.740	0.74
		FUP	Raw	3	0	3.810	0.245	3.57	3.570	3.800	4.060	4.06
			CFB	3	0	-0.153	0.179	-0.35	-0.350	-0.110	0.000	0.00
	Sodium [mmol/L]	BL	Raw	3	0	136.20	1.77	134.6	134.60	135.90	138.10	138.1
		D02	Raw	3	0	136.00	1.82	134.9	134.90	135.00	138.10	138.1
			CFB	3	0	-0.20	0.62	-0.9	-0.90	0.00	0.30	0.3
		FUP	Raw	3	0	137.67	0.31	137.4	137.40	137.60	138.00	138.0
			CFB	3	0	1.47	1.46	-0.1	-0.10	1.70	2.80	2.8
	Urea/BUN [mmol/L]	BL	Raw	3	0	3.587	1.418	1.95	1.950	4.370	4.440	4.44
		D02	Raw	3	0	3.610	1.278	2.17	2.170	4.050	4.610	4.61
			CFB	3	0	0.023	0.298	-0.32	-0.320	0.170	0.220	0.22
		FUP	Raw	3	0	3.300	1.085	2.05	2.050	3.850	4.000	4.00
			CFB	3	0	-0.287	0.352	-0.59	-0.590	-0.370	0.100	0.10
Placebo	ALT, 37 °C [U/L]	SC	Raw	3	0	16.43	6.68	11.9	11.90	13.30	24.10	24.1
		BL	Raw	3	0	14.27	3.75	12.0	12.00	12.20	18.60	18.6
		D02	Raw	3	0	15.60	2.15	13.4	13.40	15.70	17.70	17.7
			CFB	3	0	1.33	2.30	-0.9	-0.90	1.20	3.70	3.7
		FUP	Raw	3	0	17.53	4.40	13.1	13.10	17.60	21.90	21.9
			CFB	3	0	3.27	2.35	0.9	0.90	3.30	5.60	5.6

n: Number of non-missing observations; %: Percentage based on non-missing observations; Miss: Missing observations; SD: Standard deviation; TP: Timepoint of measurement; SC: Screening; BL: Baseline; FUP: Follow-up; D: Day; Raw: Raw values; CFB: Change from baseline; Cohort A1: 200mg oral dose niclosamide; Cohort A2: 600mg oral dose niclosamide; Cohort A3: 1600mg oral dose niclosamide; Fast/Fed: Treatment in cohort A3 was applied under fasting and fed conditions in the same subjects;

Output generated by program 'NIC002_T14_5_SafetyLaboratory_V02_0_0'

Table 14.5.1: Safety Laboratory
Clinical Chemistry

Part A

Cohort	Parameter	Visit		n	Miss	Mean	SD	Minimum	Lower quartile	Median	Upper quartile	Maximum
	AST, 37 °C [U/L]	SC	Raw	3	0	25.33	5.55	19.8	19.80	25.30	30.90	30.9
		BL	Raw	3	0	21.53	3.56	17.8	17.80	21.90	24.90	24.9
		D02	Raw	3	0	22.70	5.14	17.0	17.00	24.10	27.00	27.0
			CFB	3	0	1.17	3.41	-0.8	-0.80	-0.80	5.10	5.1
		FUP	Raw	3	0	26.43	6.80	19.5	19.50	26.70	33.10	33.1
			CFB	3	0	4.90	3.25	1.7	1.70	4.80	8.20	8.2
	Alkaline Phosphatase, 37 °C [U/L]	SC	Raw	3	0	61.37	10.93	50.9	50.90	60.50	72.70	72.7
		BL	Raw	3	0	64.13	11.74	50.8	50.80	68.70	72.90	72.9
		D02	Raw	3	0	65.53	7.48	56.9	56.90	69.70	70.00	70.0
			CFB	3	0	1.40	4.65	-3.2	-3.20	1.30	6.10	6.1
		FUP	Raw	3	0	65.97	9.60	56.5	56.50	65.70	75.70	75.7
			CFB	3	0	1.83	4.43	-3.0	-3.00	2.80	5.70	5.7
	Bicarbonate [mmol/L]	SC	Raw	3	0	26.30	1.99	24.0	24.00	27.40	27.50	27.5
		BL	Raw	3	0	26.93	2.70	24.0	24.00	27.50	29.30	29.3
		D02	Raw	3	0	27.57	1.43	26.0	26.00	27.90	28.80	28.8
			CFB	3	0	0.63	1.27	-0.5	-0.50	0.40	2.00	2.0
		FUP	Raw	3	0	27.57	3.25	24.4	24.40	27.40	30.90	30.9
			CFB	3	0	0.63	0.87	-0.1	-0.10	0.40	1.60	1.6
	Bilirubin, total [umol/L]	SC	Raw	3	0	10.20	1.65	8.5	8.50	10.30	11.80	11.8
		BL	Raw	3	0	9.53	2.38	7.4	7.40	9.10	12.10	12.1
		D02	Raw	3	0	9.73	2.51	7.9	7.90	8.70	12.60	12.6

n: Number of non-missing observations; %: Percentage based on non-missing observations; Miss: Missing observations; SD: Standard deviation; TP: Timepoint of measurement; SC: Screening; BL: Baseline; FUP: Follow-up; D: Day; Raw: Raw values; CFB: Change from baseline; Cohort A1: 200mg oral dose niclosamide; Cohort A2: 600mg oral dose niclosamide; Cohort A3: 1600mg oral dose niclosamide; Fast/Fed: Treatment in cohort A3 was applied under fasting and fed conditions in the same subjects;

Output generated by program 'NIC002_T14_5_SafetyLaboratory_V02_0_0'

Table 14.5.1: Safety Laboratory
Clinical Chemistry

Part A

Cohort	Parameter	Visit		n	Miss	Mean	SD	Minimum	Lower quartile	Median	Upper quartile	Maximum
	Bilirubin, total [umol/L]	D02	CFB	3	0	0.20	1.28	-1.2	-1.20	0.50	1.30	1.3
		FUP	Raw	3	0	9.63	2.04	7.3	7.30	10.50	11.10	11.1
			CFB	3	0	0.10	2.63	-1.8	-1.80	-1.00	3.10	3.1
	Calcium [mmol/L]	SC	Raw	3	0	2.317	0.097	2.21	2.210	2.340	2.400	2.40
		BL	Raw	3	0	2.330	0.095	2.22	2.220	2.380	2.390	2.39
		D02	Raw	3	0	2.327	0.061	2.26	2.260	2.340	2.380	2.38
			CFB	3	0	-0.003	0.045	-0.05	-0.050	0.000	0.040	0.04
		FUP	Raw	3	0	2.333	0.115	2.20	2.200	2.400	2.400	2.40
			CFB	3	0	0.003	0.021	-0.02	-0.020	0.010	0.020	0.02
	Creatinine Clearance MDRD [ml/min/1.73m]	SC	Raw	3	0	103.3	3.1	100	100.0	104.0	106.0	106
	Creatinine [umol/L]	SC	Raw	3	0	59.10	3.20	56.0	56.00	58.90	62.40	62.4
		BL	Raw	3	0	61.37	1.81	59.3	59.30	62.10	62.70	62.7
		D02	Raw	3	0	59.43	5.71	55.6	55.60	56.70	66.00	66.0
			CFB	3	0	-1.93	5.53	-7.1	-7.10	-2.60	3.90	3.9
		FUP	Raw	3	0	60.30	7.07	55.4	55.40	57.10	68.40	68.4
			CFB	3	0	-1.07	6.44	-5.6	-5.60	-3.90	6.30	6.3
	Gamma-GT, 37 °C [U/L]	SC	Raw	3	0	12.07	2.80	9.2	9.20	12.20	14.80	14.8
		BL	Raw	3	0	12.47	3.03	9.0	9.00	13.80	14.60	14.6
		D02	Raw	3	0	12.33	2.89	9.2	9.20	12.90	14.90	14.9

n: Number of non-missing observations; %: Percentage based on non-missing observations; Miss: Missing observations; SD: Standard deviation; TP: Timepoint of measurement; SC: Screening; BL: Baseline; FUP: Follow-up; D: Day; Raw: Raw values; CFB: Change from baseline; Cohort A1: 200mg oral dose niclosamide; Cohort A2: 600mg oral dose niclosamide; Cohort A3: 1600mg oral dose niclosamide; Fast/Fed: Treatment in cohort A3 was applied under fasting and fed conditions in the same subjects;

Output generated by program 'NIC002_T14_5_SafetyLaboratory_V02_0_0'

Table 14.5.1: Safety Laboratory
Clinical Chemistry

Part A

Cohort	Parameter	Visit		n	Miss	Mean	SD	Minimum	Lower quartile	Median	Upper quartile	Maximum
	Gamma-GT, 37 °C [U/L]	D02	CFB	3	0	-0.13	1.43	-1.7	-1.70	0.20	1.10	1.1
		FUP	Raw	3	0	12.77	3.54	8.8	8.80	13.90	15.60	15.6
			CFB	3	0	0.30	1.32	-0.7	-0.70	-0.20	1.80	1.8
	Glucose, serum [mmol/L]	SC	Raw	3	0	4.390	0.364	4.16	4.160	4.200	4.810	4.81
		BL	Raw	3	0	4.897	0.136	4.79	4.790	4.850	5.050	5.05
		D02	Raw	3	0	4.550	0.070	4.47	4.470	4.580	4.600	4.60
			CFB	3	0	-0.347	0.143	-0.47	-0.470	-0.380	-0.190	-0.19
		FUP	Raw	3	0	4.447	0.105	4.34	4.340	4.450	4.550	4.55
			CFB	3	0	-0.450	0.150	-0.60	-0.600	-0.450	-0.300	-0.30
	Magnesium [mmol/L]	SC	Raw	3	0	0.817	0.006	0.81	0.810	0.820	0.820	0.82
		BL	Raw	3	0	0.807	0.015	0.79	0.790	0.810	0.820	0.82
		D02	Raw	3	0	0.843	0.032	0.82	0.820	0.830	0.880	0.88
			CFB	3	0	0.037	0.047	0.00	0.000	0.020	0.090	0.09
		FUP	Raw	3	0	0.850	0.026	0.82	0.820	0.860	0.870	0.87
			CFB	3	0	0.043	0.040	0.00	0.000	0.050	0.080	0.08
	Potassium [mmol/L]	SC	Raw	3	0	4.167	0.212	3.94	3.940	4.200	4.360	4.36
		BL	Raw	3	0	4.230	0.147	4.14	4.140	4.150	4.400	4.40
		D02	Raw	3	0	4.370	0.279	4.05	4.050	4.500	4.560	4.56
			CFB	3	0	0.140	0.426	-0.35	-0.350	0.350	0.420	0.42
		FUP	Raw	3	0	4.320	0.320	3.95	3.950	4.500	4.510	4.51
			CFB	3	0	0.090	0.468	-0.45	-0.450	0.360	0.360	0.36

n: Number of non-missing observations; %: Percentage based on non-missing observations; Miss: Missing observations; SD: Standard deviation; TP: Timepoint of measurement; SC: Screening; BL: Baseline; FUP: Follow-up; D: Day; Raw: Raw values; CFB: Change from baseline; Cohort A1: 200mg oral dose niclosamide; Cohort A2: 600mg oral dose niclosamide; Cohort A3: 1600mg oral dose niclosamide; Fast/Fed: Treatment in cohort A3 was applied under fasting and fed conditions in the same subjects;

Output generated by program 'NIC002_T14_5_SafetyLaboratory_V02_0_0'

Table 14.5.1: Safety Laboratory
Clinical Chemistry

Part A

Cohort	Parameter	Visit		n	Miss	Mean	SD	Minimum	Lower quartile	Median	Upper quartile	Maximum
	Sodium [mmol/L]	SC	Raw	3	0	137.03	1.17	136.0	136.00	136.80	138.30	138.3
		BL	Raw	3	0	138.90	1.35	137.4	137.40	139.30	140.00	140.0
		D02	Raw	3	0	137.67	1.07	136.5	136.50	137.90	138.60	138.6
			CFB	3	0	-1.23	0.29	-1.4	-1.40	-1.40	-0.90	-0.9
		FUP	Raw	3	0	138.17	1.36	136.6	136.60	138.90	139.00	139.0
			CFB	3	0	-0.73	2.11	-2.7	-2.70	-1.00	1.50	1.5
	Urea/BUN [mmol/L]	SC	Raw	3	0	4.003	1.009	3.25	3.250	3.610	5.150	5.15
		BL	Raw	3	0	4.363	1.189	3.04	3.040	4.710	5.340	5.34
		D02	Raw	3	0	4.210	0.871	3.26	3.260	4.400	4.970	4.97
			CFB	3	0	-0.153	0.325	-0.37	-0.370	-0.310	0.220	0.22
		FUP	Raw	3	0	4.137	1.124	2.85	2.850	4.630	4.930	4.93
			CFB	3	0	-0.227	0.466	-0.71	-0.710	-0.190	0.220	0.22
Placebo Fed	ALT, 37 °C [U/L]	BL	Raw	1	0	14.70		14.7	14.70	14.70	14.70	14.7
		D02	Raw	1	0	13.40		13.4	13.40	13.40	13.40	13.4
			CFB	1	0	-1.30		-1.3	-1.30	-1.30	-1.30	-1.3
		FUP	Raw	1	0	16.00		16.0	16.00	16.00	16.00	16.0
			CFB	1	0	1.30		1.3	1.30	1.30	1.30	1.3
	AST, 37 °C [U/L]	BL	Raw	1	0	24.20		24.2	24.20	24.20	24.20	24.2
		D02	Raw	1	0	22.20		22.2	22.20	22.20	22.20	22.2
			CFB	1	0	-2.00		-2.0	-2.00	-2.00	-2.00	-2.0
		FUP	Raw	1	0	25.90		25.9	25.90	25.90	25.90	25.9
			CFB	1	0	1.70		1.7	1.70	1.70	1.70	1.7

n: Number of non-missing observations; %: Percentage based on non-missing observations; Miss: Missing observations; SD: Standard deviation; TP: Timepoint of measurement; SC: Screening; BL: Baseline; FUP: Follow-up; D: Day; Raw: Raw values; CFB: Change from baseline; Cohort A1: 200mg oral dose niclosamide; Cohort A2: 600mg oral dose niclosamide; Cohort A3: 1600mg oral dose niclosamide; Fast/Fed: Treatment in cohort A3 was applied under fasting and fed conditions in the same subjects;

Output generated by program 'NIC002_T14_5_SafetyLaboratory_V02_0_0'

Table 14.5.1: Safety Laboratory
Clinical Chemistry

Part A

Cohort	Parameter	Visit		n	Miss	Mean	SD	Minimum	Lower quartile	Median	Upper quartile	Maximum
	Alkaline Phosphatase, 37 °C [U/L]	BL	Raw	1	0	56.30		56.3	56.30	56.30	56.30	56.3
		D02	Raw	1	0	51.50		51.5	51.50	51.50	51.50	51.5
			CFB	1	0	-4.80		-4.8	-4.80	-4.80	-4.80	-4.8
		FUP	Raw	1	0	62.20		62.2	62.20	62.20	62.20	62.2
			CFB	1	0	5.90		5.9	5.90	5.90	5.90	5.9
	Bicarbonate [mmol/L]	BL	Raw	1	0	27.30		27.3	27.30	27.30	27.30	27.3
		D02	Raw	1	0	26.20		26.2	26.20	26.20	26.20	26.2
			CFB	1	0	-1.10		-1.1	-1.10	-1.10	-1.10	-1.1
		FUP	Raw	1	0	28.40		28.4	28.40	28.40	28.40	28.4
			CFB	1	0	1.10		1.1	1.10	1.10	1.10	1.1
	Bilirubin, total [umol/L]	BL	Raw	1	0	10.40		10.4	10.40	10.40	10.40	10.4
		D02	Raw	1	0	11.10		11.1	11.10	11.10	11.10	11.1
			CFB	1	0	0.70		0.7	0.70	0.70	0.70	0.7
		FUP	Raw	1	0	13.50		13.5	13.50	13.50	13.50	13.5
			CFB	1	0	3.10		3.1	3.10	3.10	3.10	3.1
	Calcium [mmol/L]	BL	Raw	1	0	2.370		2.37	2.370	2.370	2.370	2.37
		D02	Raw	1	0	2.370		2.37	2.370	2.370	2.370	2.37
			CFB	1	0	0.000		0.00	0.000	0.000	0.000	0.00
		FUP	Raw	1	0	2.440		2.44	2.440	2.440	2.440	2.44
			CFB	1	0	0.070		0.07	0.070	0.070	0.070	0.07

n: Number of non-missing observations; %: Percentage based on non-missing observations; Miss: Missing observations; SD: Standard deviation; TP: Timepoint of measurement; SC: Screening; BL: Baseline; FUP: Follow-up; D: Day; Raw: Raw values; CFB: Change from baseline; Cohort A1: 200mg oral dose niclosamide; Cohort A2: 600mg oral dose niclosamide; Cohort A3: 1600mg oral dose niclosamide; Fast/Fed: Treatment in cohort A3 was applied under fasting and fed conditions in the same subjects;

Output generated by program 'NIC002_T14_5_SafetyLaboratory_V02_0_0'

Table 14.5.1: Safety Laboratory
Clinical Chemistry

Part A

Cohort	Parameter	Visit		n	Miss	Mean	SD	Minimum	Lower quartile	Median	Upper quartile	Maximum
	Creatinine [umol/L]	BL	Raw	1	0	62.50		62.5	62.50	62.50	62.50	62.5
		D02	Raw	1	0	60.00		60.0	60.00	60.00	60.00	60.0
			CFB	1	0	-2.50		-2.5	-2.50	-2.50	-2.50	-2.5
		FUP	Raw	1	0	64.10		64.1	64.10	64.10	64.10	64.1
			CFB	1	0	1.60		1.6	1.60	1.60	1.60	1.6
	Gamma-GT, 37 °C [U/L]	BL	Raw	1	0	13.30		13.3	13.30	13.30	13.30	13.3
		D02	Raw	1	0	13.10		13.1	13.10	13.10	13.10	13.1
			CFB	1	0	-0.20		-0.2	-0.20	-0.20	-0.20	-0.2
		FUP	Raw	1	0	12.80		12.8	12.80	12.80	12.80	12.8
			CFB	1	0	-0.50		-0.5	-0.50	-0.50	-0.50	-0.5
	Glucose, serum [mmol/L]	BL	Raw	1	0	4.590		4.59	4.590	4.590	4.590	4.59
		D02	Raw	1	0	4.640		4.64	4.640	4.640	4.640	4.64
			CFB	1	0	0.050		0.05	0.050	0.050	0.050	0.05
		FUP	Raw	1	0	4.720		4.72	4.720	4.720	4.720	4.72
			CFB	1	0	0.130		0.13	0.130	0.130	0.130	0.13
	Magnesium [mmol/L]	BL	Raw	1	0	0.860		0.86	0.860	0.860	0.860	0.86
		D02	Raw	1	0	0.840		0.84	0.840	0.840	0.840	0.84
			CFB	1	0	-0.020		-0.02	-0.020	-0.020	-0.020	-0.02
		FUP	Raw	1	0	0.850		0.85	0.850	0.850	0.850	0.85
			CFB	1	0	-0.010		-0.01	-0.010	-0.010	-0.010	-0.01

n: Number of non-missing observations; %: Percentage based on non-missing observations; Miss: Missing observations; SD: Standard deviation; TP: Timepoint of measurement; SC: Screening; BL: Baseline; FUP: Follow-up; D: Day; Raw: Raw values; CFB: Change from baseline; Cohort A1: 200mg oral dose niclosamide; Cohort A2: 600mg oral dose niclosamide; Cohort A3: 1600mg oral dose niclosamide; Fast/Fed: Treatment in cohort A3 was applied under fasting and fed conditions in the same subjects;

Output generated by program 'NIC002_T14_5_SafetyLaboratory_V02_0_0'

Table 14.5.1: Safety Laboratory
Clinical Chemistry

Part A

Cohort	Parameter	Visit		n	Miss	Mean	SD	Minimum	Lower quartile	Median	Upper quartile	Maximum
	Potassium [mmol/L]	BL	Raw	1	0	4.170		4.17	4.170	4.170	4.170	4.17
		D02	Raw	1	0	4.200		4.20	4.200	4.200	4.200	4.20
			CFB	1	0	0.030		0.03	0.030	0.030	0.030	0.03
		FUP	Raw	1	0	4.540		4.54	4.540	4.540	4.540	4.54
			CFB	1	0	0.370		0.37	0.370	0.370	0.370	0.37
	Sodium [mmol/L]	BL	Raw	1	0	138.70		138.7	138.70	138.70	138.70	138.7
		D02	Raw	1	0	137.90		137.9	137.90	137.90	137.90	137.9
			CFB	1	0	-0.80		-0.8	-0.80	-0.80	-0.80	-0.8
		FUP	Raw	1	0	137.80		137.8	137.80	137.80	137.80	137.8
			CFB	1	0	-0.90		-0.9	-0.90	-0.90	-0.90	-0.9
	Urea/BUN [mmol/L]	BL	Raw	1	0	2.490		2.49	2.490	2.490	2.490	2.49
		D02	Raw	1	0	3.460		3.46	3.460	3.460	3.460	3.46
			CFB	1	0	0.970		0.97	0.970	0.970	0.970	0.97
		FUP	Raw	1	0	3.170		3.17	3.170	3.170	3.170	3.17
			CFB	1	0	0.680		0.68	0.680	0.680	0.680	0.68

n: Number of non-missing observations; %: Percentage based on non-missing observations; Miss: Missing observations; SD: Standard deviation; TP: Timepoint of measurement; SC: Screening; BL: Baseline; FUP: Follow-up; D: Day; Raw: Raw values; CFB: Change from baseline; Cohort A1: 200mg oral dose niclosamide; Cohort A2: 600mg oral dose niclosamide; Cohort A3: 1600mg oral dose niclosamide; Fast/Fed: Treatment in cohort A3 was applied under fasting and fed conditions in the same subjects;

Output generated by program 'NIC002_T14_5_SafetyLaboratory_V02_0_0'

Table 14.5.1: Safety Laboratory
Clinical Chemistry

Part B

Parameter	Visit	Treatment		n	Miss	Mean	SD	Minimum	Lower quartile	Median	Upper quartile	Maximum
ALT, 37 °C [U/L]	SC		Raw	4	0	11.90	3.54	8.6	8.95	11.50	14.85	16.0
	BL		Raw	4	0	11.50	3.47	8.3	9.05	10.70	13.95	16.3
	D02	Chewing tablet 2000 mg	Raw	4	0	11.43	3.07	8.0	9.10	11.25	13.75	15.2
			CFB	4	0	-0.08	1.69	-1.8	-1.45	-0.20	1.30	1.9
		Solution 1600 mg	Raw	4	0	12.18	3.30	10.2	10.30	10.70	14.05	17.1
			CFB	4	0	0.67	1.02	-0.6	0.00	0.70	1.35	1.9
	FUP		Raw	4	0	15.55	2.89	11.8	13.30	16.10	17.80	18.2
			CFB	4	0	4.05	2.34	1.1	2.30	4.25	5.80	6.6
AST, 37 °C [U/L]	SC		Raw	4	0	19.83	3.80	16.4	16.95	19.00	22.70	24.9
	BL		Raw	4	0	18.95	5.80	13.1	15.10	17.90	22.80	26.9
	D02	Chewing tablet 2000 mg	Raw	4	0	17.90	3.21	14.6	15.30	17.65	20.50	21.7
			CFB	4	0	-1.05	3.51	-5.2	-3.95	-0.60	1.85	2.2
		Solution 1600 mg	Raw	4	0	18.85	3.79	16.0	16.30	17.55	21.40	24.3
			CFB	4	0	-0.10	2.68	-2.6	-2.35	-0.35	2.15	2.9
	FUP		Raw	4	0	22.58	3.55	18.3	19.70	22.95	25.45	26.1
			CFB	4	0	3.63	3.66	-0.8	0.80	3.80	6.45	7.7
Alkaline Phosphatase, 37 °C [U/L]	SC		Raw	4	0	58.65	25.95	33.3	40.65	53.55	76.65	94.2
	BL		Raw	4	0	57.55	25.45	34.2	40.90	51.25	74.20	93.5
	D02	Chewing tablet 2000 mg	Raw	4	0	55.08	19.55	36.4	42.55	50.70	67.60	82.5
			CFB	4	0	-2.47	5.98	-11.0	-6.60	-0.55	1.65	2.2

n: Number of non-missing observations; %: Percentage based on non-missing observations; Miss: Missing observations; SD: Standard deviation; TP: Timepoint of measurement; SC: Screening; BL: Baseline; FUP: Follow-up; D: Day; Raw: Raw values; CFB: Change from baseline; Part B used a cross-over design;

Output generated by program 'NIC002_T14_5_SafetyLaboratory_V02_0_0'

Table 14.5.1: Safety Laboratory
Clinical Chemistry

Part B

Parameter	Visit	Treatment		n	Miss	Mean	SD	Minimum	Lower quartile	Median	Upper quartile	Maximum
Alkaline Phosphatase, 37 °C [U/L]		Solution 1600 mg	Raw	4	0	55.75	21.05	36.4	42.90	50.45	68.60	85.7
			CFB	4	0	-1.80	4.74	-7.8	-5.60	-0.80	2.00	2.2
	FUP		Raw	4	0	56.13	18.84	35.7	43.45	53.85	68.80	81.1
			CFB	4	0	-1.43	7.38	-12.4	-5.45	1.55	2.60	3.6
Bicarbonate [mmol/L]	SC		Raw	4	0	28.93	1.73	27.6	27.65	28.40	30.20	31.3
	BL		Raw	4	0	26.78	0.98	25.5	26.00	27.00	27.55	27.6
	D02	Chewing tablet 2000 mg	Raw	4	0	25.68	1.21	23.9	24.90	26.15	26.45	26.5
			CFB	4	0	-1.10	0.73	-1.7	-1.65	-1.30	-0.55	-0.1
		Solution 1600 mg	Raw	4	0	26.70	1.21	24.9	26.00	27.20	27.40	27.5
			CFB	4	0	-0.08	0.73	-0.6	-0.50	-0.35	0.35	1.0
	FUP		Raw	4	0	26.53	2.33	23.8	24.60	26.80	28.45	28.7
			CFB	4	0	-0.25	1.37	-1.7	-1.40	-0.25	0.90	1.2
Bilirubin, total [umol/L]	SC		Raw	4	0	10.53	4.41	6.2	7.20	9.80	13.85	16.3
	BL		Raw	4	0	10.00	2.27	7.7	8.50	9.60	11.50	13.1
	D02	Chewing tablet 2000 mg	Raw	4	0	10.25	3.28	6.6	7.70	10.10	12.80	14.2
			CFB	4	0	0.25	1.25	-1.1	-0.80	0.30	1.30	1.5
		Solution 1600 mg	Raw	4	0	8.70	3.25	5.5	5.95	8.55	11.45	12.2
			CFB	4	0	-1.30	1.63	-2.9	-2.55	-1.55	-0.05	0.8
	FUP		Raw	4	0	9.68	1.65	7.8	8.30	9.80	11.05	11.3
			CFB	4	0	-0.32	1.85	-2.3	-1.90	-0.20	1.25	1.4
Calcium [mmol/L]	SC		Raw	4	0	2.365	0.031	2.33	2.340	2.365	2.390	2.40

n: Number of non-missing observations; %: Percentage based on non-missing observations; Miss: Missing observations; SD: Standard deviation; TP: Timepoint of measurement; SC: Screening; BL: Baseline; FUP: Follow-up; D: Day; Raw: Raw values; CFB: Change from baseline; Part B used a cross-over design;

Output generated by program 'NIC002_T14_5_SafetyLaboratory_V02_0_0'

Table 14.5.1: Safety Laboratory
Clinical Chemistry

Part B

Parameter	Visit	Treatment		n	Miss	Mean	SD	Minimum	Lower quartile	Median	Upper quartile	Maximum
Calcium [mmol/L]	BL		Raw	4	0	2.318	0.079	2.20	2.270	2.350	2.365	2.37
	D02	Chewing tablet 2000 mg	Raw	4	0	2.325	0.048	2.26	2.290	2.335	2.360	2.37
			CFB	4	0	0.007	0.115	-0.10	-0.060	-0.020	0.075	0.17
		Solution 1600 mg	Raw	4	0	2.385	0.019	2.37	2.370	2.380	2.400	2.41
			CFB	4	0	0.068	0.087	0.00	0.005	0.040	0.130	0.19
	FUP		Raw	4	0	2.288	0.062	2.20	2.245	2.305	2.330	2.34
			CFB	4	0	-0.030	0.024	-0.05	-0.050	-0.035	-0.010	0.00
Creatinine Clearance MDRD [ml/min/1.73m]	SC		Raw	4	0	121.0	5.9	114	116.5	121.0	125.5	128
Creatinine [umol/L]	SC		Raw	4	0	51.88	1.96	50.5	50.55	51.15	53.20	54.7
	BL		Raw	4	0	49.15	3.38	44.1	47.25	50.70	51.05	51.1
	D02	Chewing tablet 2000 mg	Raw	4	0	47.95	3.68	44.9	45.80	46.80	50.10	53.3
			CFB	4	0	-1.20	4.39	-6.1	-4.90	-0.75	2.50	2.8
		Solution 1600 mg	Raw	4	0	51.58	1.89	49.5	50.20	51.40	52.95	54.0
			CFB	4	0	2.42	5.03	-0.9	-0.50	0.35	5.35	9.9
	FUP		Raw	4	0	48.65	0.57	47.8	48.30	48.90	49.00	49.0
			CFB	4	0	-0.50	3.61	-2.6	-2.45	-2.15	1.45	4.9
Gamma-GT, 37 °C [U/L]	SC		Raw	4	0	10.30	0.77	9.2	9.80	10.50	10.80	11.0
	BL		Raw	4	0	10.20	0.73	9.2	9.70	10.35	10.70	10.9
	D02	Chewing tablet 2000 mg	Raw	4	0	10.00	0.98	8.7	9.25	10.20	10.75	10.9
			CFB	4	0	-0.20	0.41	-0.5	-0.45	-0.35	0.05	0.4

n: Number of non-missing observations; %: Percentage based on non-missing observations; Miss: Missing observations; SD: Standard deviation; TP: Timepoint of measurement; SC: Screening; BL: Baseline; FUP: Follow-up; D: Day; Raw: Raw values; CFB: Change from baseline; Part B used a cross-over design;

Output generated by program 'NIC002_T14_5_SafetyLaboratory_V02_0_0'

Table 14.5.1: Safety Laboratory
Clinical Chemistry

Part B

Parameter	Visit	Treatment		n	Miss	Mean	SD	Minimum	Lower quartile	Median	Upper quartile	Maximum
Gamma-GT, 37 °C [U/L]		Solution 1600 mg	Raw	4	0	10.35	0.90	9.3	9.60	10.45	11.10	11.2
			CFB	4	0	0.15	0.34	-0.3	-0.10	0.20	0.40	0.5
	FUP		Raw	4	0	9.50	1.00	8.6	8.80	9.25	10.20	10.9
			CFB	4	0	-0.70	0.74	-1.6	-1.30	-0.60	-0.10	0.0
Glucose, serum [mmol/L]	SC		Raw	4	0	4.705	0.635	3.79	4.330	4.885	5.080	5.26
	BL		Raw	4	0	5.063	0.376	4.70	4.755	5.020	5.370	5.51
	D02	Chewing tablet 2000 mg	Raw	4	0	4.605	0.261	4.36	4.415	4.550	4.795	4.96
			CFB	4	0	-0.458	0.511	-0.88	-0.820	-0.605	-0.095	0.26
		Solution 1600 mg	Raw	4	0	4.448	0.123	4.33	4.370	4.420	4.525	4.62
			CFB	4	0	-0.615	0.279	-0.89	-0.855	-0.600	-0.375	-0.37
	FUP		Raw	4	0	4.720	0.300	4.37	4.485	4.725	4.955	5.06
			CFB	4	0	-0.343	0.640	-0.91	-0.885	-0.410	0.200	0.36
Magnesium [mmol/L]	SC		Raw	4	0	0.823	0.034	0.79	0.800	0.815	0.845	0.87
	BL		Raw	4	0	0.775	0.072	0.71	0.720	0.760	0.830	0.87
	D02	Chewing tablet 2000 mg	Raw	4	0	0.803	0.050	0.75	0.770	0.795	0.835	0.87
			CFB	4	0	0.028	0.036	0.00	0.005	0.015	0.050	0.08
		Solution 1600 mg	Raw	4	0	0.783	0.021	0.76	0.765	0.785	0.800	0.80
			CFB	4	0	0.008	0.056	-0.07	-0.030	0.020	0.045	0.06
	FUP		Raw	4	0	0.823	0.092	0.77	0.775	0.780	0.870	0.96
			CFB	4	0	0.048	0.048	-0.02	0.015	0.060	0.080	0.09
Potassium [mmol/L]	SC		Raw	4	0	4.217	0.506	3.66	3.800	4.225	4.635	4.76

n: Number of non-missing observations; %: Percentage based on non-missing observations; Miss: Missing observations; SD: Standard deviation; TP: Timepoint of measurement; SC: Screening; BL: Baseline; FUP: Follow-up; D: Day; Raw: Raw values; CFB: Change from baseline; Part B used a cross-over design;

Output generated by program 'NIC002_T14_5_SafetyLaboratory_V02_0_0'

Table 14.5.1: Safety Laboratory
Clinical Chemistry

Part B

Parameter	Visit	Treatment		n	Miss	Mean	SD	Minimum	Lower quartile	Median	Upper quartile	Maximum
Potassium [mmol/L]	BL		Raw	4	0	4.253	0.419	3.87	3.890	4.260	4.615	4.62
	D02	Chewing tablet 2000 mg	Raw	4	0	3.930	0.167	3.78	3.790	3.910	4.070	4.12
			CFB	4	0	-0.323	0.583	-0.83	-0.825	-0.355	0.180	0.25
		Solution 1600 mg	Raw	4	0	4.138	0.240	3.93	3.945	4.090	4.330	4.44
			CFB	4	0	-0.115	0.413	-0.66	-0.415	-0.055	0.185	0.31
	FUP		Raw	4	0	4.303	0.116	4.18	4.230	4.285	4.375	4.46
			CFB	4	0	0.050	0.351	-0.34	-0.245	0.080	0.345	0.38
Sodium [mmol/L]	SC		Raw	4	0	137.38	1.31	135.8	136.35	137.45	138.40	138.8
	BL		Raw	4	0	135.58	1.35	133.9	134.50	135.75	136.65	136.9
	D02	Chewing tablet 2000 mg	Raw	4	0	136.88	2.63	133.7	134.75	137.15	139.00	139.5
			CFB	4	0	1.30	3.41	-2.7	-1.00	1.15	3.60	5.6
		Solution 1600 mg	Raw	4	0	136.78	1.32	135.0	135.80	137.05	137.75	138.0
			CFB	4	0	1.20	2.28	-1.4	-0.40	1.05	2.80	4.1
	FUP		Raw	4	0	135.30	1.53	133.5	134.15	135.30	136.45	137.1
			CFB	4	0	-0.27	0.34	-0.6	-0.50	-0.35	-0.05	0.2
Urea/BUN [mmol/L]	SC		Raw	4	0	2.918	1.068	1.85	2.075	2.785	3.760	4.25
	BL		Raw	4	0	3.340	1.455	1.65	2.235	3.320	4.445	5.07
	D02	Chewing tablet 2000 mg	Raw	4	0	3.668	0.905	2.93	2.990	3.420	4.345	4.90
			CFB	4	0	0.328	0.656	-0.17	-0.100	0.100	0.755	1.28
		Solution 1600 mg	Raw	4	0	3.825	1.252	2.84	2.880	3.470	4.770	5.52
			CFB	4	0	0.485	0.493	0.10	0.150	0.325	0.820	1.19
	FUP		Raw	4	0	3.493	1.070	2.15	2.785	3.530	4.200	4.76
			CFB	4	0	0.152	0.602	-0.40	-0.355	0.095	0.660	0.82

n: Number of non-missing observations; %: Percentage based on non-missing observations; Miss: Missing observations; SD: Standard deviation; TP: Timepoint of measurement; SC: Screening; BL: Baseline; FUP: Follow-up; D: Day; Raw: Raw values; CFB: Change from baseline; Part B used a cross-over design;

Output generated by program 'NIC002_T14_5_SafetyLaboratory_V02_0_0'

Table 14.5.1: Safety Laboratory
Clinical Chemistry

Part C

Group	Parameter	Visit		n	Miss	Mean	SD	Minimum	Lower quartile	Median	Upper quartile	Maximum
Group 1	ALT, 37 °C [U/L]	SC	Raw	4	0	14.35	6.93	10.1	10.60	11.30	18.10	24.7
		BL	Raw	4	0	16.30	8.59	9.5	10.65	13.50	21.95	28.7
		D03	Raw	4	0	14.63	9.39	7.3	8.70	11.45	20.55	28.3
			CFB	4	0	-1.67	0.90	-2.4	-2.30	-1.95	-1.05	-0.4
		D08	Raw	4	0	13.95	7.65	6.4	8.00	12.85	19.90	23.7
			CFB	4	0	-2.35	4.56	-5.6	-5.30	-4.05	0.60	4.3
		FUP	Raw	4	0	17.85	7.41	11.4	12.95	15.80	22.75	28.4
			CFB	4	0	1.55	1.29	-0.3	0.80	1.90	2.30	2.7
	AST, 37 °C [U/L]	SC	Raw	4	0	18.13	3.61	15.7	16.10	16.65	20.15	23.5
		BL	Raw	4	0	20.55	4.43	15.9	17.20	20.05	23.90	26.2
		D03	Raw	4	0	17.43	4.03	14.1	15.10	16.15	19.75	23.3
			CFB	4	0	-3.13	1.58	-5.4	-4.15	-2.65	-2.10	-1.8
		D08	Raw	4	0	19.10	2.56	16.4	16.95	19.10	21.25	21.8
			CFB	4	0	-1.45	4.56	-5.5	-5.35	-1.80	2.45	3.3
		FUP	Raw	4	0	20.80	3.50	18.1	18.40	19.65	23.20	25.8
			CFB	4	0	0.25	1.72	-1.0	-0.70	-0.40	1.20	2.8
	Alkaline Phosphatase, 37 °C [U/L]	SC	Raw	4	0	56.90	12.56	39.6	48.70	59.25	65.10	69.5
		BL	Raw	4	0	60.45	14.39	39.2	52.25	65.75	68.65	71.1
		D03	Raw	4	0	55.95	15.94	32.2	46.75	63.10	65.15	65.4
			CFB	4	0	-4.50	2.46	-7.0	-6.35	-4.85	-2.65	-1.3
		D08	Raw	4	0	49.43	11.82	33.0	41.65	51.90	57.20	60.9
			CFB	4	0	-11.03	6.19	-17.6	-16.30	-10.60	-5.75	-5.3

n: Number of non-missing observations; %: Percentage based on non-missing observations; Miss: Missing observations; SD: Standard deviation; TP: Timepoint of measurement; SC: Screening; BL: Baseline; FUP: Follow-up; D: Day; Raw: Raw values; CFB: Change from baseline; Group 1: 1200 mg niclosamide solution; Group 2: 1600 mg niclosamide solution; Group 3: Placebo;

Output generated by program 'NIC002_T14_5_SafetyLaboratory_V02_0_0'

Table 14.5.1: Safety Laboratory
Clinical Chemistry

Part C

Group	Parameter	Visit		n	Miss	Mean	SD	Minimum	Lower quartile	Median	Upper quartile	Maximum
	Alkaline Phosphatase, 37 °C [U/L]	FUP	Raw	4	0	54.38	13.24	34.6	47.15	60.10	61.60	62.7
			CFB	4	0	-6.07	3.14	-10.6	-8.10	-5.10	-4.05	-3.5
	Bicarbonate [mmol/L]	SC	Raw	4	0	26.13	1.30	24.4	25.15	26.35	27.10	27.4
		BL	Raw	4	0	25.98	0.97	24.6	25.40	26.20	26.55	26.9
		D03	Raw	4	0	24.60	0.73	24.0	24.05	24.40	25.15	25.6
			CFB	4	0	-1.37	1.68	-2.9	-2.50	-1.80	-0.25	1.0
		D08	Raw	4	0	23.83	0.94	22.7	23.20	23.80	24.45	25.0
			CFB	4	0	-2.15	1.41	-3.5	-3.35	-2.20	-0.95	-0.7
		FUP	Raw	4	0	24.98	2.76	21.0	23.10	25.95	26.85	27.0
			CFB	4	0	-1.00	2.83	-5.2	-2.70	0.20	0.70	0.8
	Bilirubin, total [umol/L]	SC	Raw	4	0	10.33	4.15	6.4	6.75	10.35	13.90	14.2
		BL	Raw	4	0	11.50	4.97	6.1	8.00	10.95	15.00	18.0
		D03	Raw	4	0	12.55	2.94	8.4	10.50	13.40	14.60	15.0
			CFB	4	0	1.05	2.71	-3.0	-0.40	2.25	2.50	2.7
		D08	Raw	4	0	10.50	3.03	6.9	8.25	10.50	12.75	14.1
			CFB	4	0	-1.00	7.54	-11.1	-6.75	0.90	4.75	5.3
		FUP	Raw	4	0	8.50	1.39	7.0	7.50	8.35	9.50	10.3
			CFB	4	0	-3.00	6.00	-11.0	-7.50	-1.80	1.50	2.6
	Calcium [mmol/L]	SC	Raw	4	0	2.363	0.052	2.30	2.320	2.370	2.405	2.41
		BL	Raw	4	0	2.360	0.047	2.30	2.325	2.365	2.395	2.41
		D03	Raw	4	0	2.338	0.050	2.27	2.305	2.345	2.370	2.39
			CFB	4	0	-0.022	0.080	-0.11	-0.090	-0.015	0.045	0.05
		D08	Raw	4	0	2.328	0.043	2.27	2.295	2.335	2.360	2.37

n: Number of non-missing observations; %: Percentage based on non-missing observations; Miss: Missing observations; SD: Standard deviation; TP: Timepoint of measurement; SC: Screening; BL: Baseline; FUP: Follow-up; D: Day; Raw: Raw values; CFB: Change from baseline; Group 1: 1200 mg niclosamide solution; Group 2: 1600 mg niclosamide solution; Group 3: Placebo;

Output generated by program 'NIC002_T14_5_SafetyLaboratory_V02_0_0'

Table 14.5.1: Safety Laboratory
Clinical Chemistry

Part C

Group	Parameter	Visit		n	Miss	Mean	SD	Minimum	Lower quartile	Median	Upper quartile	Maximum
	Calcium [mmol/L]	D08	CFB	4	0	-0.032	0.085	-0.14	-0.100	-0.020	0.035	0.05
		FUP	Raw	4	0	2.330	0.065	2.24	2.285	2.345	2.375	2.39
			CFB	4	0	-0.030	0.096	-0.14	-0.110	-0.020	0.050	0.06
	Creatinine Clearance MDRD [ml/min/1.73m]	SC	Raw	4	0	105.0	1.4	103	104.0	105.5	106.0	106
	Creatinine [umol/L]	SC	Raw	4	0	58.30	1.81	56.7	57.20	57.80	59.40	60.9
		BL	Raw	4	0	58.13	4.07	53.8	55.25	57.60	61.00	63.5
		D03	Raw	4	0	55.93	4.79	49.8	52.90	56.20	58.95	61.5
			CFB	4	0	-2.20	4.66	-8.7	-5.35	-1.15	0.95	2.2
		D08	Raw	4	0	57.75	3.02	54.6	55.25	57.65	60.25	61.1
			CFB	4	0	-0.38	4.18	-3.9	-3.15	-1.60	2.40	5.6
		FUP	Raw	4	0	54.90	3.04	51.5	53.05	54.60	56.75	58.9
			CFB	4	0	-3.23	4.67	-8.9	-7.05	-2.40	0.60	0.8
	Gamma-GT, 37 °C [U/L]	SC	Raw	4	0	11.20	3.31	8.6	9.05	10.10	13.35	16.0
		BL	Raw	4	0	10.28	1.92	7.8	8.90	10.45	11.65	12.4
		D03	Raw	4	0	10.08	2.39	7.4	8.50	9.85	11.65	13.2
			CFB	4	0	-0.20	0.69	-0.8	-0.60	-0.40	0.20	0.8
		D08	Raw	4	0	8.70	1.44	7.7	7.75	8.15	9.65	10.8
			CFB	4	0	-1.58	1.11	-2.4	-2.35	-1.95	-0.80	0.0
		FUP	Raw	4	0	8.95	1.38	7.3	7.85	9.05	10.05	10.4
			CFB	4	0	-1.33	0.64	-2.0	-1.80	-1.40	-0.85	-0.5

n: Number of non-missing observations; %: Percentage based on non-missing observations; Miss: Missing observations; SD: Standard deviation; TP: Timepoint of measurement; SC: Screening; BL: Baseline; FUP: Follow-up; D: Day; Raw: Raw values; CFB: Change from baseline; Group 1: 1200 mg niclosamide solution; Group 2: 1600 mg niclosamide solution; Group 3: Placebo;

Output generated by program 'NIC002_T14_5_SafetyLaboratory_V02_0_0'

Table 14.5.1: Safety Laboratory
Clinical Chemistry

Part C

Group	Parameter	Visit		n	Miss	Mean	SD	Minimum	Lower quartile	Median	Upper quartile	Maximum
	Glucose, serum [mmol/L]	SC	Raw	4	0	4.715	0.295	4.42	4.480	4.680	4.950	5.08
		BL	Raw	4	0	4.643	0.313	4.32	4.375	4.660	4.910	4.93
		D03	Raw	4	0	3.928	0.389	3.37	3.660	4.065	4.195	4.21
			CFB	4	0	-0.715	0.192	-0.95	-0.835	-0.715	-0.595	-0.48
		D08	Raw	4	0	4.318	0.462	3.71	3.990	4.375	4.645	4.81
			CFB	4	0	-0.325	0.248	-0.61	-0.530	-0.305	-0.120	-0.08
		FUP	Raw	4	0	4.203	0.850	3.58	3.650	3.895	4.755	5.44
			CFB	4	0	-0.440	0.746	-1.21	-0.975	-0.550	0.095	0.55
	Magnesium [mmol/L]	SC	Raw	4	0	0.813	0.038	0.76	0.790	0.820	0.835	0.85
		BL	Raw	4	0	0.785	0.024	0.75	0.770	0.795	0.800	0.80
		D03	Raw	4	0	0.778	0.033	0.74	0.750	0.780	0.805	0.81
			CFB	4	0	-0.008	0.054	-0.06	-0.050	-0.015	0.035	0.06
		D08	Raw	4	0	0.785	0.059	0.73	0.735	0.780	0.835	0.85
			CFB	4	0	-0.000	0.075	-0.07	-0.065	-0.000	0.065	0.07
		FUP	Raw	4	0	0.790	0.050	0.72	0.755	0.805	0.825	0.83
			CFB	4	0	0.005	0.057	-0.08	-0.030	0.030	0.040	0.04
	Potassium [mmol/L]	SC	Raw	4	0	4.070	0.408	3.65	3.815	4.000	4.325	4.63
		BL	Raw	4	0	3.935	0.505	3.27	3.625	3.985	4.245	4.50
		D03	Raw	4	0	4.078	0.243	3.74	3.905	4.140	4.250	4.29
			CFB	4	0	0.143	0.328	-0.29	-0.100	0.195	0.385	0.47
		D08	Raw	4	0	3.995	0.229	3.83	3.835	3.915	4.155	4.32
			CFB	4	0	0.060	0.342	-0.18	-0.160	-0.070	0.280	0.56
		FUP	Raw	4	0	4.283	0.386	3.92	4.020	4.195	4.545	4.82
			CFB	4	0	0.347	0.725	-0.58	-0.225	0.485	0.920	1.00

n: Number of non-missing observations; %: Percentage based on non-missing observations; Miss: Missing observations; SD: Standard deviation; TP: Timepoint of measurement; SC: Screening; BL: Baseline; FUP: Follow-up; D: Day; Raw: Raw values; CFB: Change from baseline; Group 1: 1200 mg niclosamide solution; Group 2: 1600 mg niclosamide solution; Group 3: Placebo;

Output generated by program 'NIC002_T14_5_SafetyLaboratory_V02_0_0'

Table 14.5.1: Safety Laboratory
Clinical Chemistry

Part C

Group	Parameter	Visit		n	Miss	Mean	SD	Minimum	Lower quartile	Median	Upper quartile	Maximum
	Sodium [mmol/L]	SC	Raw	4	0	137.35	1.88	134.6	136.15	138.10	138.55	138.6
		BL	Raw	4	0	135.98	1.44	134.6	134.75	135.90	137.20	137.5
		D03	Raw	4	0	137.40	1.02	136.2	136.75	137.35	138.05	138.7
			CFB	4	0	1.42	1.75	-0.2	0.15	1.05	2.70	3.8
		D08	Raw	4	0	137.30	0.95	135.9	136.75	137.65	137.85	138.0
			CFB	4	0	1.32	1.03	0.2	0.65	1.20	2.00	2.7
		FUP	Raw	4	0	136.88	0.82	136.4	136.45	136.50	137.30	138.1
			CFB	4	0	0.90	2.08	-1.1	-0.75	0.60	2.55	3.5
	Urea/BUN [mmol/L]	SC	Raw	4	0	3.320	0.376	2.99	3.010	3.260	3.630	3.77
		BL	Raw	4	0	2.833	0.916	1.80	2.220	2.755	3.445	4.02
		D03	Raw	4	0	3.818	0.709	3.18	3.220	3.740	4.415	4.61
			CFB	4	0	0.985	0.439	0.59	0.605	0.985	1.365	1.38
		D08	Raw	4	0	3.458	0.282	3.18	3.240	3.415	3.675	3.82
			CFB	4	0	0.625	0.646	-0.20	0.230	0.660	1.020	1.38
		FUP	Raw	4	0	2.900	0.421	2.36	2.595	2.940	3.205	3.36
			CFB	4	0	0.067	1.103	-0.97	-0.740	-0.160	0.875	1.56
Group 2	ALT, 37 °C [U/L]	SC	Raw	4	0	16.60	6.51	11.0	11.30	15.40	21.90	24.6
		BL	Raw	4	0	16.40	7.14	10.5	10.75	14.75	22.05	25.6
		D03	Raw	4	0	13.00	4.64	8.9	9.00	12.80	17.00	17.5
			CFB	4	0	-3.40	3.14	-8.1	-5.05	-1.95	-1.75	-1.6
		D08	Raw	4	0	18.90	9.58	9.9	11.75	17.00	26.05	31.7
			CFB	4	0	2.50	11.28	-12.0	-6.30	4.40	11.30	13.2
		FUP	Raw	4	0	21.75	8.86	10.6	15.00	22.60	28.50	31.2

n: Number of non-missing observations; %: Percentage based on non-missing observations; Miss: Missing observations; SD: Standard deviation; TP: Timepoint of measurement; SC: Screening; BL: Baseline; FUP: Follow-up; D: Day; Raw: Raw values; CFB: Change from baseline; Group 1: 1200 mg niclosamide solution; Group 2: 1600 mg niclosamide solution; Group 3: Placebo;

Output generated by program 'NIC002_T14_5_SafetyLaboratory_V02_0_0'

Table 14.5.1: Safety Laboratory
Clinical Chemistry

Part C

Group	Parameter	Visit		n	Miss	Mean	SD	Minimum	Lower quartile	Median	Upper quartile	Maximum
Group 2	ALT, 37 °C [U/L]	FUP	CFB	4	0	5.35	10.07	-6.2	-3.05	6.40	13.75	14.8
	AST, 37 °C [U/L]	SC	Raw	4	0	18.05	2.52	14.6	16.30	18.55	19.80	20.5
		BL	Raw	4	0	17.98	2.90	13.8	16.30	18.80	19.65	20.5
		D03	Raw	4	0	15.13	1.87	12.9	13.60	15.30	16.65	17.0
			CFB	4	0	-2.85	1.54	-4.5	-4.00	-3.00	-1.70	-0.9
		D08	Raw	4	0	19.35	3.70	15.6	16.70	18.75	22.00	24.3
			CFB	4	0	1.37	4.06	-3.2	-2.00	1.60	4.75	5.5
		FUP	Raw	4	0	21.35	4.24	16.5	18.35	21.10	24.35	26.7
			CFB	4	0	3.37	4.67	-2.3	-0.40	3.95	7.15	7.9
	Alkaline Phosphatase, 37 °C [U/L]	SC	Raw	4	0	43.73	9.74	34.9	37.65	41.20	49.80	57.6
		BL	Raw	4	0	43.75	10.70	33.5	37.30	41.35	50.20	58.8
		D03	Raw	4	0	41.75	10.40	30.1	35.20	40.75	48.30	55.4
			CFB	4	0	-2.00	1.62	-3.4	-3.40	-2.10	-0.60	-0.4
		D08	Raw	4	0	41.20	11.43	27.1	34.15	41.30	48.25	55.1
			CFB	4	0	-2.55	3.09	-6.4	-5.05	-1.95	-0.05	0.1
		FUP	Raw	4	0	42.10	11.11	29.7	34.45	41.10	49.75	56.5
			CFB	4	0	-1.65	2.46	-3.8	-3.10	-2.35	-0.20	1.9
	Bicarbonate [mmol/L]	SC	Raw	4	0	25.95	0.82	25.2	25.30	25.80	26.60	27.0
		BL	Raw	4	0	25.00	0.95	23.8	24.25	25.15	25.75	25.9
		D03	Raw	4	0	24.08	1.25	22.6	23.05	24.20	25.10	25.3
			CFB	4	0	-0.93	1.42	-3.0	-1.80	-0.45	-0.05	0.2
		D08	Raw	4	0	22.75	1.57	21.3	21.45	22.55	24.05	24.6

n: Number of non-missing observations; %: Percentage based on non-missing observations; Miss: Missing observations; SD: Standard deviation; TP: Timepoint of measurement; SC: Screening; BL: Baseline; FUP: Follow-up; D: Day; Raw: Raw values; CFB: Change from baseline; Group 1: 1200 mg niclosamide solution; Group 2: 1600 mg niclosamide solution; Group 3: Placebo;

Output generated by program 'NIC002_T14_5_SafetyLaboratory_V02_0_0'

Table 14.5.1: Safety Laboratory
Clinical Chemistry

Part C

Group	Parameter	Visit		n	Miss	Mean	SD	Minimum	Lower quartile	Median	Upper quartile	Maximum
	Bicarbonate [mmol/L]	D08	CFB	4	0	-2.25	1.44	-4.3	-3.25	-1.75	-1.25	-1.2
		FUP	Raw	4	0	24.73	2.66	20.8	23.25	25.70	26.20	26.7
			CFB	4	0	-0.28	1.86	-3.0	-1.45	0.45	0.90	1.0
	Bilirubin, total [umol/L]	SC	Raw	4	0	11.80	1.22	10.5	10.85	11.70	12.75	13.3
		BL	Raw	4	0	10.53	4.89	6.2	6.50	9.75	14.55	16.4
		D03	Raw	4	0	14.03	7.97	7.2	8.30	11.90	19.75	25.1
			CFB	4	0	3.50	3.65	0.4	1.05	2.45	5.95	8.7
		D08	Raw	4	0	10.55	4.66	6.8	7.55	9.05	13.55	17.3
			CFB	4	0	0.03	4.12	-5.9	-2.50	1.20	2.55	3.6
		FUP	Raw	4	0	10.48	2.49	7.4	8.95	10.50	12.00	13.5
			CFB	4	0	-0.05	3.27	-2.9	-2.55	-0.80	2.45	4.3
	Calcium [mmol/L]	SC	Raw	4	0	2.300	0.034	2.26	2.275	2.300	2.325	2.34
		BL	Raw	4	0	2.243	0.047	2.21	2.210	2.225	2.275	2.31
		D03	Raw	4	0	2.275	0.045	2.21	2.245	2.290	2.305	2.31
			CFB	4	0	0.032	0.043	0.00	0.000	0.020	0.065	0.09
		D08	Raw	4	0	2.270	0.059	2.19	2.225	2.285	2.315	2.32
			CFB	4	0	0.027	0.090	-0.05	-0.050	0.025	0.105	0.11
		FUP	Raw	4	0	2.265	0.042	2.22	2.230	2.265	2.300	2.31
			CFB	4	0	0.023	0.053	-0.02	-0.010	0.005	0.055	0.10
	Creatinine Clearance MDRD [ml/min/1.73m]	SC	Raw	4	0	110.0	11.4	100	102.0	107.0	118.0	126

n: Number of non-missing observations; %: Percentage based on non-missing observations; Miss: Missing observations; SD: Standard deviation; TP: Timepoint of measurement; SC: Screening; BL: Baseline; FUP: Follow-up; D: Day; Raw: Raw values; CFB: Change from baseline; Group 1: 1200 mg niclosamide solution; Group 2: 1600 mg niclosamide solution; Group 3: Placebo;

Output generated by program 'NIC002_T14_5_SafetyLaboratory_V02_0_0'

Table 14.5.1: Safety Laboratory
Clinical Chemistry

Part C

Group	Parameter	Visit		n	Miss	Mean	SD	Minimum	Lower quartile	Median	Upper quartile	Maximum
	Creatinine [umol/L]	SC	Raw	4	0	55.23	5.24	49.5	51.10	54.95	59.35	61.5
		BL	Raw	4	0	56.75	5.49	50.2	52.30	57.25	61.20	62.3
		D03	Raw	4	0	57.98	5.24	53.1	53.45	58.00	62.50	62.8
			CFB	4	0	1.22	1.57	-0.6	-0.05	1.30	2.50	2.9
		D08	Raw	4	0	58.75	5.04	54.0	54.60	58.15	62.90	64.7
			CFB	4	0	2.00	1.40	0.8	0.90	1.70	3.10	3.8
		FUP	Raw	4	0	55.50	2.66	52.0	53.45	56.05	57.55	57.9
			CFB	4	0	-1.25	2.89	-4.4	-3.65	-1.20	1.15	1.8
	Gamma-GT, 37 °C [U/L]	SC	Raw	4	0	16.93	7.35	9.4	12.25	15.65	21.60	27.0
		BL	Raw	4	0	15.58	5.60	10.1	11.50	14.55	19.65	23.1
		D03	Raw	4	0	15.00	4.60	10.0	11.55	14.60	18.45	20.8
			CFB	4	0	-0.57	1.16	-2.3	-1.20	-0.10	0.05	0.2
		D08	Raw	4	0	13.55	4.33	9.3	10.45	12.75	16.65	19.4
			CFB	4	0	-2.03	1.28	-3.7	-3.00	-1.80	-1.05	-0.8
		FUP	Raw	4	0	13.83	4.10	10.2	10.80	12.85	16.85	19.4
			CFB	4	0	-1.75	1.56	-3.7	-2.80	-1.70	-0.70	0.1
	Glucose, serum [mmol/L]	SC	Raw	4	0	4.905	0.473	4.34	4.535	4.925	5.275	5.43
		BL	Raw	4	0	5.120	0.522	4.72	4.755	4.950	5.485	5.86
		D03	Raw	4	0	4.473	0.412	3.98	4.150	4.490	4.795	4.93
			CFB	4	0	-0.648	0.262	-0.93	-0.870	-0.630	-0.425	-0.40
		D08	Raw	4	0	4.700	0.287	4.42	4.455	4.695	4.945	4.99
			CFB	4	0	-0.420	0.370	-0.96	-0.630	-0.300	-0.210	-0.12
		FUP	Raw	4	0	4.858	0.334	4.40	4.615	4.935	5.100	5.16
			CFB	4	0	-0.263	0.589	-1.03	-0.710	-0.170	0.185	0.32

n: Number of non-missing observations; %: Percentage based on non-missing observations; Miss: Missing observations; SD: Standard deviation; TP: Timepoint of measurement; SC: Screening; BL: Baseline; FUP: Follow-up; D: Day; Raw: Raw values; CFB: Change from baseline; Group 1: 1200 mg niclosamide solution; Group 2: 1600 mg niclosamide solution; Group 3: Placebo;

Output generated by program 'NIC002_T14_5_SafetyLaboratory_V02_0_0'

Table 14.5.1: Safety Laboratory
Clinical Chemistry

Part C

Group	Parameter	Visit		n	Miss	Mean	SD	Minimum	Lower quartile	Median	Upper quartile	Maximum
	Magnesium [mmol/L]	SC	Raw	4	0	0.795	0.024	0.77	0.775	0.795	0.815	0.82
		BL	Raw	4	0	0.800	0.020	0.79	0.790	0.790	0.810	0.83
		D03	Raw	4	0	0.810	0.014	0.80	0.800	0.805	0.820	0.83
			CFB	4	0	0.010	0.029	-0.03	-0.010	0.015	0.030	0.04
		D08	Raw	4	0	0.795	0.010	0.79	0.790	0.790	0.800	0.81
			CFB	4	0	-0.005	0.025	-0.04	-0.020	0.000	0.010	0.02
		FUP	Raw	4	0	0.803	0.021	0.78	0.785	0.805	0.820	0.82
			CFB	4	0	0.002	0.019	-0.01	-0.010	-0.005	0.015	0.03
	Potassium [mmol/L]	SC	Raw	4	0	4.240	0.389	3.90	3.945	4.150	4.535	4.76
		BL	Raw	4	0	4.215	0.215	3.95	4.050	4.230	4.380	4.45
		D03	Raw	4	0	4.105	0.302	3.72	3.880	4.135	4.330	4.43
			CFB	4	0	-0.110	0.088	-0.23	-0.170	-0.095	-0.050	-0.02
		D08	Raw	4	0	4.003	0.276	3.80	3.840	3.900	4.165	4.41
			CFB	4	0	-0.213	0.151	-0.39	-0.330	-0.210	-0.095	-0.04
		FUP	Raw	4	0	3.943	0.219	3.67	3.780	3.955	4.105	4.19
			CFB	4	0	-0.273	0.015	-0.29	-0.285	-0.270	-0.260	-0.26
	Sodium [mmol/L]	SC	Raw	4	0	137.18	0.43	136.9	136.90	137.00	137.45	137.8
		BL	Raw	4	0	137.35	0.45	136.8	137.05	137.35	137.65	137.9
		D03	Raw	4	0	137.58	0.54	136.9	137.20	137.60	137.95	138.2
			CFB	4	0	0.22	0.10	0.1	0.15	0.25	0.30	0.3
		D08	Raw	4	0	136.83	1.86	134.7	135.30	136.90	138.35	138.8
			CFB	4	0	-0.53	1.55	-2.1	-1.75	-0.70	0.70	1.4
		FUP	Raw	4	0	136.78	1.03	135.4	136.15	136.90	137.40	137.9

n: Number of non-missing observations; %: Percentage based on non-missing observations; Miss: Missing observations; SD: Standard deviation; TP: Timepoint of measurement; SC: Screening; BL: Baseline; FUP: Follow-up; D: Day; Raw: Raw values; CFB: Change from baseline; Group 1: 1200 mg niclosamide solution; Group 2: 1600 mg niclosamide solution; Group 3: Placebo;

Output generated by program 'NIC002_T14_5_SafetyLaboratory_V02_0_0'

Table 14.5.1: Safety Laboratory
Clinical Chemistry

Part C

Group	Parameter	Visit		n	Miss	Mean	SD	Minimum	Lower quartile	Median	Upper quartile	Maximum
	Sodium [mmol/L]	FUP	CFB	4	0	-0.58	1.09	-1.9	-1.45	-0.45	0.30	0.5
	Urea/BUN [mmol/L]	SC	Raw	4	0	4.085	1.718	2.04	2.665	4.365	5.505	5.57
		BL	Raw	4	0	4.520	1.509	2.73	3.270	4.780	5.770	5.79
		D03	Raw	4	0	4.135	1.192	2.74	3.145	4.300	5.125	5.20
			CFB	4	0	-0.385	0.323	-0.70	-0.645	-0.425	-0.125	0.01
		D08	Raw	4	0	3.923	1.173	2.36	3.035	4.170	4.810	4.99
			CFB	4	0	-0.598	0.463	-1.16	-0.960	-0.565	-0.235	-0.10
		FUP	Raw	4	0	3.958	1.778	2.48	2.515	3.595	5.400	6.16
			CFB	4	0	-0.563	0.791	-1.26	-1.205	-0.700	0.080	0.41
Group 3	ALT, 37 °C [U/L]	SC	Raw	4	0	11.90	1.35	10.1	10.95	12.10	12.85	13.3
		BL	Raw	4	0	12.13	2.69	8.2	10.45	13.05	13.80	14.2
		D03	Raw	4	0	11.08	1.84	8.6	9.85	11.35	12.30	13.0
			CFB	4	0	-1.05	1.62	-2.6	-2.45	-1.00	0.35	0.4
		D08	Raw	4	0	16.55	4.20	13.1	13.25	15.55	19.85	22.0
			CFB	4	0	4.43	4.04	-1.1	1.95	5.10	6.90	8.6
		FUP	Raw	4	0	17.05	2.18	15.2	15.75	16.40	18.35	20.2
			CFB	4	0	4.93	2.34	2.3	2.95	5.20	6.90	7.0
	AST, 37 °C [U/L]	SC	Raw	4	0	19.78	3.04	16.1	17.30	20.15	22.25	22.7
		BL	Raw	4	0	19.83	3.78	15.5	16.70	20.10	22.95	23.6
		D03	Raw	4	0	16.65	3.04	13.0	14.15	17.15	19.15	19.3
			CFB	4	0	-3.18	0.83	-4.3	-3.80	-2.95	-2.55	-2.5
		D08	Raw	4	0	21.30	2.48	19.7	19.90	20.25	22.70	25.0
			CFB	4	0	1.47	3.23	-3.2	-0.50	2.45	3.45	4.2

n: Number of non-missing observations; %: Percentage based on non-missing observations; Miss: Missing observations; SD: Standard deviation; TP: Timepoint of measurement; SC: Screening; BL: Baseline; FUP: Follow-up; D: Day; Raw: Raw values; CFB: Change from baseline; Group 1: 1200 mg niclosamide solution; Group 2: 1600 mg niclosamide solution; Group 3: Placebo;

Output generated by program 'NIC002_T14_5_SafetyLaboratory_V02_0_0'

Table 14.5.1: Safety Laboratory
Clinical Chemistry

Part C

Group	Parameter	Visit		n	Miss	Mean	SD	Minimum	Lower quartile	Median	Upper quartile	Maximum
	AST, 37 °C [U/L]	FUP	Raw	4	0	21.05	3.44	17.1	18.30	21.10	23.80	24.9
			CFB	4	0	1.22	0.57	0.4	0.85	1.45	1.60	1.6
	Alkaline Phosphatase, 37 °C [U/L]	SC	Raw	4	0	50.18	11.45	38.1	41.35	49.05	59.00	64.5
		BL	Raw	4	0	49.63	13.71	34.6	40.45	48.10	58.80	67.7
		D03	Raw	4	0	46.63	11.35	34.6	38.35	45.25	54.90	61.4
			CFB	4	0	-3.00	2.80	-6.3	-5.25	-2.85	-0.75	0.0
		D08	Raw	4	0	45.10	11.41	34.3	36.35	43.05	53.85	60.0
			CFB	4	0	-4.53	3.86	-7.9	-7.80	-4.95	-1.25	-0.3
		FUP	Raw	4	0	46.38	12.29	33.9	37.10	44.65	55.65	62.3
			CFB	4	0	-3.25	2.84	-6.0	-5.70	-3.15	-0.80	-0.7
	Bicarbonate [mmol/L]	SC	Raw	4	0	25.78	1.23	24.4	25.00	25.65	26.55	27.4
		BL	Raw	4	0	25.75	1.45	24.2	24.80	25.55	26.70	27.7
		D03	Raw	4	0	26.95	2.16	23.8	25.70	27.65	28.20	28.7
			CFB	4	0	1.20	1.70	-0.4	-0.25	1.10	2.65	3.0
		D08	Raw	4	0	25.63	1.74	23.3	24.50	25.85	26.75	27.5
			CFB	4	0	-0.12	0.57	-0.9	-0.55	0.05	0.30	0.3
		FUP	Raw	4	0	26.55	1.83	24.2	25.10	27.00	28.00	28.0
			CFB	4	0	0.80	2.03	-1.7	-0.85	1.15	2.45	2.6
	Bilirubin, total [umol/L]	SC	Raw	4	0	15.18	6.76	8.2	9.40	15.45	20.95	21.6
		BL	Raw	4	0	11.13	5.20	6.1	6.65	11.15	15.60	16.1
		D03	Raw	4	0	11.88	4.71	7.8	7.80	11.70	15.95	16.3
			CFB	4	0	0.75	0.66	0.2	0.35	0.55	1.15	1.7

n: Number of non-missing observations; %: Percentage based on non-missing observations; Miss: Missing observations; SD: Standard deviation; TP: Timepoint of measurement; SC: Screening; BL: Baseline; FUP: Follow-up; D: Day; Raw: Raw values; CFB: Change from baseline; Group 1: 1200 mg niclosamide solution; Group 2: 1600 mg niclosamide solution; Group 3: Placebo;

Output generated by program 'NIC002_T14_5_SafetyLaboratory_V02_0_0'

Table 14.5.1: Safety Laboratory
Clinical Chemistry

Part C

Group	Parameter	Visit		n	Miss	Mean	SD	Minimum	Lower quartile	Median	Upper quartile	Maximum
	Bilirubin, total [umol/L]	D08	Raw	4	0	12.30	5.10	7.3	7.90	12.60	16.70	16.7
			CFB	4	0	1.17	0.42	0.6	0.90	1.25	1.45	1.6
		FUP	Raw	4	0	14.90	7.21	9.2	9.55	12.80	20.25	24.8
			CFB	4	0	3.78	3.53	0.6	1.30	2.90	6.25	8.7
	Calcium [mmol/L]	SC	Raw	4	0	2.290	0.080	2.18	2.240	2.305	2.340	2.37
		BL	Raw	4	0	2.235	0.070	2.17	2.175	2.230	2.295	2.31
		D03	Raw	4	0	2.253	0.105	2.13	2.175	2.250	2.330	2.38
			CFB	4	0	0.017	0.048	-0.04	-0.020	0.020	0.055	0.07
		D08	Raw	4	0	2.263	0.113	2.14	2.170	2.260	2.355	2.39
			CFB	4	0	0.028	0.050	-0.04	-0.005	0.035	0.060	0.08
		FUP	Raw	4	0	2.250	0.112	2.16	2.165	2.220	2.335	2.40
			CFB	4	0	0.015	0.050	-0.01	-0.010	-0.010	0.040	0.09
	Creatinine Clearance MDRD [ml/min/1.73m]	SC	Raw	4	0	100.3	16.3	80	88.5	101.0	112.0	119
	Creatinine [umol/L]	SC	Raw	4	0	60.83	8.72	51.1	55.30	59.95	66.35	72.3
		BL	Raw	4	0	58.15	6.11	50.3	53.50	58.95	62.80	64.4
		D03	Raw	4	0	53.33	5.53	46.7	48.90	53.75	57.75	59.1
			CFB	4	0	-4.83	2.56	-8.0	-6.80	-4.60	-2.85	-2.1
		D08	Raw	4	0	53.65	4.08	49.2	50.60	53.30	56.70	58.8
			CFB	4	0	-4.50	3.83	-9.8	-7.25	-3.55	-1.75	-1.1
		FUP	Raw	4	0	56.95	4.35	52.6	53.30	56.75	60.60	61.7
			CFB	4	0	-1.20	4.04	-4.9	-4.50	-1.80	2.10	3.7

n: Number of non-missing observations; %: Percentage based on non-missing observations; Miss: Missing observations; SD: Standard deviation; TP: Timepoint of measurement; SC: Screening; BL: Baseline; FUP: Follow-up; D: Day; Raw: Raw values; CFB: Change from baseline; Group 1: 1200 mg niclosamide solution; Group 2: 1600 mg niclosamide solution; Group 3: Placebo;

Output generated by program 'NIC002_T14_5_SafetyLaboratory_V02_0_0'

Table 14.5.1: Safety Laboratory
Clinical Chemistry

Part C

Group	Parameter	Visit		n	Miss	Mean	SD	Minimum	Lower quartile	Median	Upper quartile	Maximum
	Gamma-GT, 37 °C [U/L]	SC	Raw	4	0	10.20	1.54	8.1	9.05	10.60	11.35	11.5
		BL	Raw	4	0	9.33	1.56	7.2	8.20	9.65	10.45	10.8
		D03	Raw	4	0	9.33	1.68	7.3	8.25	9.30	10.40	11.4
			CFB	4	0	0.00	0.54	-0.7	-0.35	0.05	0.35	0.6
		D08	Raw	4	0	9.10	1.45	7.8	7.90	8.90	10.30	10.8
			CFB	4	0	-0.22	1.28	-2.1	-1.05	0.30	0.60	0.6
		FUP	Raw	4	0	9.23	1.15	7.9	8.45	9.15	10.00	10.7
			CFB	4	0	-0.10	0.75	-1.1	-0.60	0.00	0.40	0.7
	Glucose, serum [mmol/L]	SC	Raw	4	0	5.090	0.398	4.69	4.840	5.015	5.340	5.64
		BL	Raw	4	0	4.733	0.283	4.33	4.535	4.830	4.930	4.94
		D03	Raw	4	0	4.303	0.424	3.87	3.990	4.240	4.615	4.86
			CFB	4	0	-0.430	0.445	-1.07	-0.720	-0.295	-0.140	-0.06
		D08	Raw	4	0	4.560	0.417	4.13	4.240	4.510	4.880	5.09
			CFB	4	0	-0.173	0.318	-0.59	-0.395	-0.135	0.050	0.17
		FUP	Raw	4	0	4.613	0.380	4.08	4.380	4.695	4.845	4.98
			CFB	4	0	-0.120	0.241	-0.26	-0.255	-0.230	0.015	0.24
	Magnesium [mmol/L]	SC	Raw	4	0	0.805	0.058	0.73	0.765	0.810	0.845	0.87
		BL	Raw	4	0	0.820	0.024	0.80	0.800	0.815	0.840	0.85
		D03	Raw	4	0	0.785	0.071	0.74	0.740	0.755	0.830	0.89
			CFB	4	0	-0.035	0.056	-0.09	-0.075	-0.045	0.005	0.04
		D08	Raw	4	0	0.785	0.050	0.73	0.750	0.780	0.820	0.85
			CFB	4	0	-0.035	0.045	-0.10	-0.065	-0.020	-0.005	0.00
		FUP	Raw	4	0	0.795	0.047	0.74	0.760	0.795	0.830	0.85

n: Number of non-missing observations; %: Percentage based on non-missing observations; Miss: Missing observations; SD: Standard deviation; TP: Timepoint of measurement; SC: Screening; BL: Baseline; FUP: Follow-up; D: Day; Raw: Raw values; CFB: Change from baseline; Group 1: 1200 mg niclosamide solution; Group 2: 1600 mg niclosamide solution; Group 3: Placebo;

Output generated by program 'NIC002_T14_5_SafetyLaboratory_V02_0_0'

Table 14.5.1: Safety Laboratory
Clinical Chemistry

Part C

Group	Parameter	Visit		n	Miss	Mean	SD	Minimum	Lower quartile	Median	Upper quartile	Maximum
	Magnesium [mmol/L]	FUP	CFB	4	0	-0.025	0.045	-0.09	-0.055	-0.010	0.005	0.01
	Potassium [mmol/L]	SC	Raw	4	0	4.235	0.254	3.98	4.020	4.230	4.450	4.50
		BL	Raw	4	0	4.088	0.304	3.80	3.880	4.020	4.295	4.51
		D03	Raw	4	0	4.365	0.168	4.19	4.225	4.360	4.505	4.55
			CFB	4	0	0.278	0.236	0.04	0.075	0.285	0.480	0.50
		D08	Raw	4	0	4.068	0.224	3.86	3.890	4.030	4.245	4.35
			CFB	4	0	-0.020	0.411	-0.59	-0.265	0.060	0.225	0.39
		FUP	Raw	4	0	4.153	0.345	3.92	3.935	4.015	4.370	4.66
			CFB	4	0	0.065	0.485	-0.59	-0.235	0.135	0.365	0.58
	Sodium [mmol/L]	SC	Raw	4	0	136.48	1.59	134.6	135.30	136.45	137.65	138.4
		BL	Raw	4	0	135.25	2.00	133.3	133.75	134.90	136.75	137.9
		D03	Raw	4	0	136.90	0.80	135.7	136.45	137.25	137.35	137.4
			CFB	4	0	1.65	2.31	-0.7	-0.30	1.60	3.60	4.1
		D08	Raw	4	0	137.35	1.07	135.9	136.60	137.55	138.10	138.4
			CFB	4	0	2.10	1.97	0.3	0.40	2.05	3.80	4.0
		FUP	Raw	4	0	136.13	1.56	134.5	134.80	136.15	137.45	137.7
			CFB	4	0	0.87	1.86	-1.1	-0.65	0.80	2.40	3.0
	Urea/BUN [mmol/L]	SC	Raw	4	0	3.085	0.555	2.54	2.630	3.030	3.540	3.74
		BL	Raw	4	0	3.285	1.091	2.22	2.425	3.130	4.145	4.66
		D03	Raw	4	0	3.745	1.076	2.98	3.145	3.330	4.345	5.34
			CFB	4	0	0.460	0.495	-0.28	0.200	0.680	0.720	0.76
		D08	Raw	4	0	3.370	0.684	2.93	2.990	3.080	3.750	4.39
			CFB	4	0	0.085	0.604	-0.52	-0.395	0.015	0.565	0.83
		FUP	Raw	4	0	3.253	0.610	2.49	2.860	3.270	3.645	3.98
			CFB	4	0	-0.032	0.575	-0.68	-0.500	-0.025	0.435	0.60

n: Number of non-missing observations; %: Percentage based on non-missing observations; Miss: Missing observations; SD: Standard deviation; TP: Timepoint of measurement; SC: Screening; BL: Baseline; FUP: Follow-up; D: Day; Raw: Raw values; CFB: Change from baseline; Group 1: 1200 mg niclosamide solution; Group 2: 1600 mg niclosamide solution; Group 3: Placebo;

Output generated by program 'NIC002_T14_5_SafetyLaboratory_V02_0_0'

Table 14.5.2: Safety Laboratory
Haematology

Part A

Cohort	Parameter	Visit		n	Miss	Mean	SD	Minimum	Lower quartile	Median	Upper quartile	Maximum
Cohort A1	Basophils, % [%]	SC	Raw	3	0	0.93	0.40	0.5	0.50	1.00	1.30	1.3
		BL	Raw	3	0	1.00	0.17	0.8	0.80	1.10	1.10	1.1
		D02	Raw	3	0	1.03	0.25	0.8	0.80	1.00	1.30	1.3
			CFB	3	0	0.03	0.15	-0.1	-0.10	0.00	0.20	0.2
		FUP	Raw	3	0	1.03	0.06	1.0	1.00	1.00	1.10	1.1
			CFB	3	0	0.03	0.15	-0.1	-0.10	0.00	0.20	0.2
	Basophils, abs. [10^9/L]	SC	Raw	3	0	0.050	0.017	0.03	0.030	0.060	0.060	0.06
		BL	Raw	3	0	0.047	0.006	0.04	0.040	0.050	0.050	0.05
		D02	Raw	3	0	0.053	0.015	0.04	0.040	0.050	0.070	0.07
			CFB	3	0	0.007	0.012	0.00	0.000	0.000	0.020	0.02
		FUP	Raw	3	0	0.047	0.006	0.04	0.040	0.050	0.050	0.05
			CFB	3	0	0.000	0.010	-0.01	-0.010	0.000	0.010	0.01
	Eosinophils, % [%]	SC	Raw	3	0	3.13	1.64	1.9	1.90	2.50	5.00	5.0
		BL	Raw	3	0	3.50	2.25	2.1	2.10	2.30	6.10	6.1
		D02	Raw	3	0	2.20	0.75	1.4	1.40	2.30	2.90	2.9
			CFB	3	0	-1.30	1.68	-3.2	-3.20	-0.70	0.00	0.0
		FUP	Raw	3	0	3.13	0.91	2.3	2.30	3.00	4.10	4.1
			CFB	3	0	-0.37	1.48	-2.0	-2.00	0.00	0.90	0.9
	Eosinophils, abs. [10^9/L]	SC	Raw	3	0	0.177	0.107	0.11	0.110	0.120	0.300	0.30
		BL	Raw	3	0	0.160	0.095	0.10	0.100	0.110	0.270	0.27
		D02	Raw	3	0	0.110	0.040	0.07	0.070	0.110	0.150	0.15

n: Number of non-missing observations; %: Percentage based on non-missing observations; Miss: Missing observations; SD: Standard deviation; TP: Timepoint of measurement; SC: Screening; BL: Baseline; FUP: Follow-up; D: Day; Raw: Raw values; CFB: Change from baseline; Cohort A1: 200mg oral dose niclosamide; Cohort A2: 600mg oral dose niclosamide; Cohort A3: 1600mg oral dose niclosamide; Fast/Fed: Treatment in cohort A3 was applied under fasting and fed conditions in the same subjects;

Output generated by program 'NIC002_T14_5_SafetyLaboratory_V02_0_0'

Table 14.5.2: Safety Laboratory
Haematology

Part A

Cohort	Parameter	Visit		n	Miss	Mean	SD	Minimum	Lower quartile	Median	Upper quartile	Maximum
	Eosinophils, abs. $[10^9/L]$	D02	CFB	3	0	-0.050	0.066	-0.12	-0.120	-0.040	0.010	0.01
		FUP	Raw	3	0	0.143	0.038	0.10	0.100	0.160	0.170	0.17
			CFB	3	0	-0.017	0.076	-0.10	-0.100	0.000	0.050	0.05
	Erythrocytes $[10^{12}/L]$	SC	Raw	3	0	3.813	0.139	3.66	3.660	3.850	3.930	3.93
		BL	Raw	3	0	3.750	0.155	3.60	3.600	3.740	3.910	3.91
		D02	Raw	3	0	3.827	0.278	3.53	3.530	3.870	4.080	4.08
			CFB	3	0	0.077	0.129	-0.07	-0.070	0.130	0.170	0.17
		FUP	Raw	3	0	3.717	0.251	3.43	3.430	3.820	3.900	3.90
			CFB	3	0	-0.033	0.127	-0.17	-0.170	-0.010	0.080	0.08
	Haematocrit [L/L]	SC	Raw	3	0	0.350	0.000	0.35	0.350	0.350	0.350	0.35
		BL	Raw	3	0	0.343	0.006	0.34	0.340	0.340	0.350	0.35
		D02	Raw	3	0	0.347	0.015	0.33	0.330	0.350	0.360	0.36
			CFB	3	0	0.003	0.012	-0.01	-0.010	0.010	0.010	0.01
		FUP	Raw	3	0	0.340	0.010	0.33	0.330	0.340	0.350	0.35
			CFB	3	0	-0.003	0.006	-0.01	-0.010	0.000	0.000	0.00
	Haemoglobin [g/L]	SC	Raw	3	0	118.3	2.5	116	116.0	118.0	121.0	121
		BL	Raw	3	0	116.7	3.5	113	113.0	117.0	120.0	120
		D02	Raw	3	0	119.7	6.4	115	115.0	117.0	127.0	127
			CFB	3	0	3.0	4.6	-2	-2.0	4.0	7.0	7
		FUP	Raw	3	0	115.3	4.5	111	111.0	115.0	120.0	120
			CFB	3	0	-1.3	4.2	-6	-6.0	0.0	2.0	2

n: Number of non-missing observations; %: Percentage based on non-missing observations; Miss: Missing observations; SD: Standard deviation; TP: Timepoint of measurement; SC: Screening; BL: Baseline; FUP: Follow-up; D: Day; Raw: Raw values; CFB: Change from baseline; Cohort A1: 200mg oral dose niclosamide; Cohort A2: 600mg oral dose niclosamide; Cohort A3: 1600mg oral dose niclosamide; Fast/Fed: Treatment in cohort A3 was applied under fasting and fed conditions in the same subjects;

Output generated by program 'NIC002_T14_5_SafetyLaboratory_V02_0_0'

Table 14.5.2: Safety Laboratory
Haematology

Part A

Cohort	Parameter	Visit		n	Miss	Mean	SD	Minimum	Lower quartile	Median	Upper quartile	Maximum
	Leucocytes [10^9/L]	SC	Raw	3	0	5.503	0.676	4.73	4.730	5.800	5.980	5.98
		BL	Raw	3	0	4.657	0.438	4.36	4.360	4.450	5.160	5.16
		D02	Raw	3	0	4.947	0.256	4.77	4.770	4.830	5.240	5.24
			CFB	3	0	0.290	0.570	-0.33	-0.330	0.410	0.790	0.79
		FUP	Raw	3	0	4.580	0.603	4.11	4.110	4.370	5.260	5.26
			CFB	3	0	-0.077	0.232	-0.34	-0.340	0.010	0.100	0.10
	Lymphocytes, % [%]	SC	Raw	3	0	32.77	4.12	29.5	29.50	31.40	37.40	37.4
		BL	Raw	3	0	35.23	7.23	26.9	26.90	38.90	39.90	39.9
		D02	Raw	3	0	34.30	4.55	29.4	29.40	35.10	38.40	38.4
			CFB	3	0	-0.93	3.19	-3.8	-3.80	-1.50	2.50	2.5
		FUP	Raw	3	0	34.97	2.95	32.1	32.10	34.80	38.00	38.0
			CFB	3	0	-0.27	7.48	-6.8	-6.80	-1.90	7.90	7.9
	Lymphocytes, abs. [10^9/L]	SC	Raw	3	0	1.787	0.086	1.71	1.710	1.770	1.880	1.88
		BL	Raw	3	0	1.620	0.199	1.39	1.390	1.730	1.740	1.74
		D02	Raw	3	0	1.697	0.240	1.42	1.420	1.830	1.840	1.84
			CFB	3	0	0.077	0.042	0.03	0.030	0.090	0.110	0.11
		FUP	Raw	3	0	1.603	0.260	1.32	1.320	1.660	1.830	1.83
			CFB	3	0	-0.017	0.429	-0.41	-0.410	-0.080	0.440	0.44
	Monocytes, % [%]	SC	Raw	3	0	7.90	1.18	6.6	6.60	8.20	8.90	8.9
		BL	Raw	3	0	7.30	1.41	6.0	6.00	7.10	8.80	8.8
		D02	Raw	3	0	6.47	1.12	5.2	5.20	6.90	7.30	7.3
			CFB	3	0	-0.83	0.65	-1.5	-1.50	-0.80	-0.20	-0.2

n: Number of non-missing observations; %: Percentage based on non-missing observations; Miss: Missing observations; SD: Standard deviation; TP: Timepoint of measurement; SC: Screening; BL: Baseline; FUP: Follow-up; D: Day; Raw: Raw values; CFB: Change from baseline; Cohort A1: 200mg oral dose niclosamide; Cohort A2: 600mg oral dose niclosamide; Cohort A3: 1600mg oral dose niclosamide; Fast/Fed: Treatment in cohort A3 was applied under fasting and fed conditions in the same subjects;

Output generated by program 'NIC002_T14_5_SafetyLaboratory_V02_0_0'

Table 14.5.2: Safety Laboratory
Haematology

Part A

Cohort	Parameter	Visit		n	Miss	Mean	SD	Minimum	Lower quartile	Median	Upper quartile	Maximum
	Monocytes, % [%]	FUP	Raw	3	0	6.97	0.23	6.7	6.70	7.10	7.10	7.1
			CFB	3	0	-0.33	1.23	-1.7	-1.70	0.00	0.70	0.7
	Monocytes, abs. [10^9/L]	SC	Raw	3	0	0.433	0.084	0.38	0.380	0.390	0.530	0.53
		BL	Raw	3	0	0.337	0.046	0.31	0.310	0.310	0.390	0.39
		D02	Raw	3	0	0.320	0.066	0.25	0.250	0.330	0.380	0.38
			CFB	3	0	-0.017	0.040	-0.06	-0.060	-0.010	0.020	0.02
		FUP	Raw	3	0	0.317	0.031	0.29	0.290	0.310	0.350	0.35
			CFB	3	0	-0.020	0.072	-0.10	-0.100	0.000	0.040	0.04
	Neutrophils, % [%]	SC	Raw	3	0	55.27	5.62	50.6	50.60	53.70	61.50	61.5
		BL	Raw	3	0	52.97	9.99	45.1	45.10	49.60	64.20	64.2
		D02	Raw	3	0	56.00	6.32	51.4	51.40	53.40	63.20	63.2
			CFB	3	0	3.03	4.77	-1.0	-1.00	1.80	8.30	8.3
		FUP	Raw	3	0	53.90	2.16	51.5	51.50	54.50	55.70	55.7
			CFB	3	0	0.93	10.18	-9.7	-9.70	1.90	10.60	10.6
	Neutrophils, abs. [10^9/L]	SC	Raw	3	0	3.057	0.605	2.39	2.390	3.210	3.570	3.57
		BL	Raw	3	0	2.493	0.711	2.01	2.010	2.160	3.310	3.31
		D02	Raw	3	0	2.767	0.301	2.45	2.450	2.800	3.050	3.05
			CFB	3	0	0.273	0.525	-0.26	-0.260	0.290	0.790	0.79
		FUP	Raw	3	0	2.470	0.347	2.25	2.250	2.290	2.870	2.87
			CFB	3	0	-0.023	0.373	-0.44	-0.440	0.090	0.280	0.28
	Platelets [10^9/L]	SC	Raw	3	0	228.7	44.8	178	178.0	245.0	263.0	263

n: Number of non-missing observations; %: Percentage based on non-missing observations; Miss: Missing observations; SD: Standard deviation; TP: Timepoint of measurement; SC: Screening; BL: Baseline; FUP: Follow-up; D: Day; Raw: Raw values; CFB: Change from baseline; Cohort A1: 200mg oral dose niclosamide; Cohort A2: 600mg oral dose niclosamide; Cohort A3: 1600mg oral dose niclosamide; Fast/Fed: Treatment in cohort A3 was applied under fasting and fed conditions in the same subjects;

Output generated by program 'NIC002_T14_5_SafetyLaboratory_V02_0_0'

Table 14.5.2: Safety Laboratory
Haematology

Part A

Cohort	Parameter	Visit		n	Miss	Mean	SD	Minimum	Lower quartile	Median	Upper quartile	Maximum
	Platelets [10^9/L]	BL	Raw	3	0	220.3	41.1	184	184.0	212.0	265.0	265
		D02	Raw	3	0	236.0	49.1	183	183.0	245.0	280.0	280
			CFB	3	0	15.7	17.0	-1	-1.0	15.0	33.0	33
		FUP	Raw	3	0	234.7	52.9	177	177.0	246.0	281.0	281
			CFB	3	0	14.3	20.6	-7	-7.0	16.0	34.0	34
Cohort A2	Basophils, % [%]	SC	Raw	3	0	0.70	0.44	0.4	0.40	0.50	1.20	1.2
		BL	Raw	3	0	0.77	0.32	0.4	0.40	0.90	1.00	1.0
		D02	Raw	3	0	0.47	0.12	0.4	0.40	0.40	0.60	0.6
			CFB	3	0	-0.30	0.44	-0.6	-0.60	-0.50	0.20	0.2
		FUP	Raw	3	0	0.53	0.15	0.4	0.40	0.50	0.70	0.7
			CFB	3	0	-0.23	0.21	-0.4	-0.40	-0.30	0.00	0.0
	Basophils, abs. [10^9/L]	SC	Raw	3	0	0.033	0.015	0.02	0.020	0.030	0.050	0.05
		BL	Raw	3	0	0.040	0.010	0.03	0.030	0.040	0.050	0.05
		D02	Raw	3	0	0.027	0.012	0.02	0.020	0.020	0.040	0.04
			CFB	3	0	-0.013	0.021	-0.03	-0.030	-0.020	0.010	0.01
		FUP	Raw	3	0	0.030	0.010	0.02	0.020	0.030	0.040	0.04
			CFB	3	0	-0.010	0.010	-0.02	-0.020	-0.010	0.000	0.00
	Eosinophils, % [%]	SC	Raw	3	0	3.07	0.60	2.5	2.50	3.00	3.70	3.7
		BL	Raw	3	0	2.97	0.29	2.8	2.80	2.80	3.30	3.3
		D02	Raw	3	0	2.13	0.61	1.6	1.60	2.00	2.80	2.8
			CFB	3	0	-0.83	0.85	-1.7	-1.70	-0.80	0.00	0.0
		FUP	Raw	3	0	2.87	0.91	1.9	1.90	3.00	3.70	3.7
			CFB	3	0	-0.10	0.92	-0.9	-0.90	-0.30	0.90	0.9

n: Number of non-missing observations; %: Percentage based on non-missing observations; Miss: Missing observations; SD: Standard deviation; TP: Timepoint of measurement; SC: Screening; BL: Baseline; FUP: Follow-up; D: Day; Raw: Raw values; CFB: Change from baseline; Cohort A1: 200mg oral dose niclosamide; Cohort A2: 600mg oral dose niclosamide; Cohort A3: 1600mg oral dose niclosamide; Fast/Fed: Treatment in cohort A3 was applied under fasting and fed conditions in the same subjects;

Output generated by program 'NIC002_T14_5_SafetyLaboratory_V02_0_0'

Table 14.5.2: Safety Laboratory
Haematology

Part A

Cohort	Parameter	Visit		n	Miss	Mean	SD	Minimum	Lower quartile	Median	Upper quartile	Maximum
	Eosinophils, abs. [10^9/L]	SC	Raw	3	0	0.147	0.025	0.12	0.120	0.150	0.170	0.17
		BL	Raw	3	0	0.170	0.044	0.14	0.140	0.150	0.220	0.22
		D02	Raw	3	0	0.117	0.032	0.08	0.080	0.130	0.140	0.14
			CFB	3	0	-0.053	0.038	-0.08	-0.080	-0.070	-0.010	-0.01
		FUP	Raw	3	0	0.147	0.047	0.11	0.110	0.130	0.200	0.20
			CFB	3	0	-0.023	0.076	-0.09	-0.090	-0.040	0.060	0.06
	Erythrocytes [10^{12}/L]	SC	Raw	3	0	4.130	0.407	3.89	3.890	3.900	4.600	4.60
		BL	Raw	3	0	4.200	0.384	3.93	3.930	4.030	4.640	4.64
		D02	Raw	3	0	4.223	0.480	3.71	3.710	4.300	4.660	4.66
			CFB	3	0	0.023	0.245	-0.22	-0.220	0.020	0.270	0.27
		FUP	Raw	3	0	3.980	0.295	3.69	3.690	3.970	4.280	4.28
			CFB	3	0	-0.220	0.151	-0.36	-0.360	-0.240	-0.060	-0.06
	Haematocrit [L/L]	SC	Raw	3	0	0.373	0.032	0.35	0.350	0.360	0.410	0.41
		BL	Raw	3	0	0.377	0.029	0.36	0.360	0.360	0.410	0.41
		D02	Raw	3	0	0.377	0.035	0.34	0.340	0.380	0.410	0.41
			CFB	3	0	0.000	0.020	-0.02	-0.020	0.000	0.020	0.02
		FUP	Raw	3	0	0.357	0.021	0.34	0.340	0.350	0.380	0.38
			CFB	3	0	-0.020	0.010	-0.03	-0.030	-0.020	-0.010	-0.01
	Haemoglobin [g/L]	SC	Raw	3	0	128.7	11.2	119	119.0	126.0	141.0	141
		BL	Raw	3	0	131.3	11.2	123	123.0	127.0	144.0	144
		D02	Raw	3	0	132.0	12.1	121	121.0	130.0	145.0	145

n: Number of non-missing observations; %: Percentage based on non-missing observations; Miss: Missing observations; SD: Standard deviation; TP: Timepoint of measurement; SC: Screening; BL: Baseline; FUP: Follow-up; D: Day; Raw: Raw values; CFB: Change from baseline; Cohort A1: 200mg oral dose niclosamide; Cohort A2: 600mg oral dose niclosamide; Cohort A3: 1600mg oral dose niclosamide; Fast/Fed: Treatment in cohort A3 was applied under fasting and fed conditions in the same subjects;

Output generated by program 'NIC002_T14_5_SafetyLaboratory_V02_0_0'

Table 14.5.2: Safety Laboratory
Haematology

Part A

Cohort	Parameter	Visit		n	Miss	Mean	SD	Minimum	Lower quartile	Median	Upper quartile	Maximum
	Haemoglobin [g/L]	D02	CFB	3	0	0.7	6.5	-6	-6.0	1.0	7.0	7
		FUP	Raw	3	0	125.0	8.7	119	119.0	121.0	135.0	135
			CFB	3	0	-6.3	3.8	-9	-9.0	-8.0	-2.0	-2
	Leucocytes [10^9/L]	SC	Raw	3	0	4.967	1.666	3.97	3.970	4.040	6.890	6.89
		BL	Raw	3	0	5.773	1.737	4.54	4.540	5.020	7.760	7.76
		D02	Raw	3	0	5.540	1.314	4.66	4.660	4.910	7.050	7.05
			CFB	3	0	-0.233	0.551	-0.71	-0.710	-0.360	0.370	0.37
		FUP	Raw	3	0	5.347	1.655	3.69	3.690	5.350	7.000	7.00
			CFB	3	0	-0.427	0.657	-0.85	-0.850	-0.760	0.330	0.33
	Lymphocytes, % [%]	SC	Raw	3	0	34.27	7.58	28.3	28.30	31.70	42.80	42.8
		BL	Raw	3	0	32.30	9.89	23.5	23.50	30.40	43.00	43.0
		D02	Raw	3	0	25.80	8.04	16.6	16.60	29.30	31.50	31.5
			CFB	3	0	-6.50	5.21	-11.5	-11.50	-6.90	-1.10	-1.1
		FUP	Raw	3	0	31.37	12.59	18.0	18.00	33.10	43.00	43.0
			CFB	3	0	-0.93	4.18	-5.5	-5.50	0.00	2.70	2.7
	Lymphocytes, abs. [10^9/L]	SC	Raw	3	0	1.647	0.352	1.26	1.260	1.730	1.950	1.95
		BL	Raw	3	0	1.787	0.391	1.38	1.380	1.820	2.160	2.16
		D02	Raw	3	0	1.360	0.165	1.17	1.170	1.440	1.470	1.47
			CFB	3	0	-0.427	0.422	-0.69	-0.690	-0.650	0.060	0.06
		FUP	Raw	3	0	1.593	0.612	1.22	1.220	1.260	2.300	2.30
			CFB	3	0	-0.193	0.351	-0.56	-0.560	-0.160	0.140	0.14

n: Number of non-missing observations; %: Percentage based on non-missing observations; Miss: Missing observations; SD: Standard deviation; TP: Timepoint of measurement; SC: Screening; BL: Baseline; FUP: Follow-up; D: Day; Raw: Raw values; CFB: Change from baseline; Cohort A1: 200mg oral dose niclosamide; Cohort A2: 600mg oral dose niclosamide; Cohort A3: 1600mg oral dose niclosamide; Fast/Fed: Treatment in cohort A3 was applied under fasting and fed conditions in the same subjects;

Output generated by program 'NIC002_T14_5_SafetyLaboratory_V02_0_0'

Table 14.5.2: Safety Laboratory
Haematology

Part A

Cohort	Parameter	Visit		n	Miss	Mean	SD	Minimum	Lower quartile	Median	Upper quartile	Maximum
	Monocytes, % [%]	SC	Raw	3	0	7.27	0.74	6.7	6.70	7.00	8.10	8.1
		BL	Raw	3	0	9.50	3.21	5.8	5.80	11.20	11.50	11.5
		D02	Raw	3	0	7.30	0.35	7.1	7.10	7.10	7.70	7.7
			CFB	3	0	-2.20	3.03	-4.1	-4.10	-3.80	1.30	1.3
		FUP	Raw	3	0	10.10	1.00	9.1	9.10	10.10	11.10	11.1
			CFB	3	0	0.60	2.36	-1.1	-1.10	-0.40	3.30	3.3
	Monocytes, abs. [10^9/L]	SC	Raw	3	0	0.357	0.110	0.27	0.270	0.320	0.480	0.48
		BL	Raw	3	0	0.510	0.056	0.45	0.450	0.520	0.560	0.56
		D02	Raw	3	0	0.403	0.087	0.33	0.330	0.380	0.500	0.50
			CFB	3	0	-0.107	0.143	-0.23	-0.230	-0.140	0.050	0.05
		FUP	Raw	3	0	0.530	0.115	0.41	0.410	0.540	0.640	0.64
			CFB	3	0	0.020	0.154	-0.11	-0.110	-0.020	0.190	0.19
	Neutrophils, % [%]	SC	Raw	3	0	54.70	8.28	45.6	45.60	56.70	61.80	61.8
		BL	Raw	3	0	54.47	12.76	42.0	42.00	53.90	67.50	67.5
		D02	Raw	3	0	64.30	8.26	58.2	58.20	61.00	73.70	73.7
			CFB	3	0	9.83	5.53	6.2	6.20	7.10	16.20	16.2
		FUP	Raw	3	0	55.13	14.26	42.5	42.50	52.30	70.60	70.6
			CFB	3	0	0.67	2.35	-1.6	-1.60	0.50	3.10	3.1
	Neutrophils, abs. [10^9/L]	SC	Raw	3	0	2.783	1.295	1.84	1.840	2.250	4.260	4.26
		BL	Raw	3	0	3.267	1.717	2.11	2.110	2.450	5.240	5.24
		D02	Raw	3	0	3.633	1.364	2.71	2.710	2.990	5.200	5.20
			CFB	3	0	0.367	0.353	-0.04	-0.040	0.540	0.600	0.60

n: Number of non-missing observations; %: Percentage based on non-missing observations; Miss: Missing observations; SD: Standard deviation; TP: Timepoint of measurement; SC: Screening; BL: Baseline; FUP: Follow-up; D: Day; Raw: Raw values; CFB: Change from baseline; Cohort A1: 200mg oral dose niclosamide; Cohort A2: 600mg oral dose niclosamide; Cohort A3: 1600mg oral dose niclosamide; Fast/Fed: Treatment in cohort A3 was applied under fasting and fed conditions in the same subjects;

Output generated by program 'NIC002_T14_5_SafetyLaboratory_V02_0_0'

Table 14.5.2: Safety Laboratory
Haematology

Part A

Cohort	Parameter	Visit		n	Miss	Mean	SD	Minimum	Lower quartile	Median	Upper quartile	Maximum
	Neutrophils, abs. [10^9/L]	FUP	Raw	3	0	3.047	1.648	1.93	1.930	2.270	4.940	4.94
			CFB	3	0	-0.220	0.347	-0.52	-0.520	-0.300	0.160	0.16
	Platelets [10^9/L]	SC	Raw	3	0	260.0	11.3	247	247.0	266.0	267.0	267
		BL	Raw	3	0	266.3	25.1	245	245.0	260.0	294.0	294
		D02	Raw	3	0	269.0	36.3	238	238.0	260.0	309.0	309
			CFB	3	0	2.7	21.4	-22	-22.0	15.0	15.0	15
		FUP	Raw	3	0	259.3	18.6	244	244.0	254.0	280.0	280
			CFB	3	0	-7.0	13.9	-16	-16.0	-14.0	9.0	9
Cohort A3 Fast	Basophils, % [%]	SC	Raw	3	0	0.73	0.35	0.4	0.40	0.70	1.10	1.1
		BL	Raw	3	0	0.43	0.15	0.3	0.30	0.40	0.60	0.6
		D02	Raw	3	0	0.67	0.25	0.4	0.40	0.70	0.90	0.9
			CFB	3	0	0.23	0.21	0.0	0.00	0.30	0.40	0.4
		FUP	Raw	3	0	0.87	0.38	0.6	0.60	0.70	1.30	1.3
			CFB	3	0	0.43	0.25	0.2	0.20	0.40	0.70	0.7
	Basophils, abs. [10^9/L]	SC	Raw	3	0	0.037	0.015	0.02	0.020	0.040	0.050	0.05
		BL	Raw	3	0	0.027	0.006	0.02	0.020	0.030	0.030	0.03
		D02	Raw	3	0	0.037	0.015	0.02	0.020	0.040	0.050	0.05
			CFB	3	0	0.010	0.010	0.00	0.000	0.010	0.020	0.02
		FUP	Raw	3	0	0.040	0.010	0.03	0.030	0.040	0.050	0.05
			CFB	3	0	0.013	0.006	0.01	0.010	0.010	0.020	0.02
	Eosinophils, % [%]	SC	Raw	3	0	3.13	1.74	1.8	1.80	2.50	5.10	5.1

n: Number of non-missing observations; %: Percentage based on non-missing observations; Miss: Missing observations; SD: Standard deviation; TP: Timepoint of measurement; SC: Screening; BL: Baseline; FUP: Follow-up; D: Day; Raw: Raw values; CFB: Change from baseline; Cohort A1: 200mg oral dose niclosamide; Cohort A2: 600mg oral dose niclosamide; Cohort A3: 1600mg oral dose niclosamide; Fast/Fed: Treatment in cohort A3 was applied under fasting and fed conditions in the same subjects;

Output generated by program 'NIC002_T14_5_SafetyLaboratory_V02_0_0'

Table 14.5.2: Safety Laboratory
Haematology

Part A

Cohort	Parameter	Visit		n	Miss	Mean	SD	Minimum	Lower quartile	Median	Upper quartile	Maximum
	Eosinophils, % [%]	BL	Raw	3	0	1.97	0.91	1.0	1.00	2.10	2.80	2.8
		D02	Raw	3	0	1.80	0.95	1.2	1.20	1.30	2.90	2.9
			CFB	3	0	-0.17	0.55	-0.8	-0.80	0.10	0.20	0.2
		FUP	Raw	3	0	2.03	1.29	1.1	1.10	1.50	3.50	3.5
			CFB	3	0	0.07	0.93	-1.0	-1.00	0.50	0.70	0.7
	Eosinophils, abs. [10^9/L]	SC	Raw	3	0	0.153	0.075	0.08	0.080	0.150	0.230	0.23
		BL	Raw	3	0	0.113	0.023	0.10	0.100	0.100	0.140	0.14
		D02	Raw	3	0	0.093	0.032	0.07	0.070	0.080	0.130	0.13
			CFB	3	0	-0.020	0.010	-0.03	-0.030	-0.020	-0.010	-0.01
		FUP	Raw	3	0	0.093	0.045	0.05	0.050	0.090	0.140	0.14
			CFB	3	0	-0.020	0.026	-0.05	-0.050	-0.010	0.000	0.00
	Erythrocytes [10^{12}/L]	SC	Raw	3	0	4.053	0.330	3.73	3.730	4.040	4.390	4.39
		BL	Raw	3	0	4.103	0.292	3.92	3.920	3.950	4.440	4.44
		D02	Raw	3	0	4.180	0.417	3.70	3.700	4.390	4.450	4.45
			CFB	3	0	0.077	0.405	-0.25	-0.250	-0.050	0.530	0.53
		FUP	Raw	3	0	3.927	0.356	3.52	3.520	4.080	4.180	4.18
			CFB	3	0	-0.177	0.304	-0.43	-0.430	-0.260	0.160	0.16
	Haematocrit [L/L]	SC	Raw	3	0	0.347	0.025	0.32	0.320	0.350	0.370	0.37
		BL	Raw	3	0	0.350	0.017	0.34	0.340	0.340	0.370	0.37
		D02	Raw	3	0	0.360	0.036	0.32	0.320	0.370	0.390	0.39
			CFB	3	0	0.010	0.036	-0.02	-0.020	0.000	0.050	0.05
		FUP	Raw	3	0	0.333	0.029	0.30	0.300	0.350	0.350	0.35
			CFB	3	0	-0.017	0.025	-0.04	-0.040	-0.020	0.010	0.01

n: Number of non-missing observations; %: Percentage based on non-missing observations; Miss: Missing observations; SD: Standard deviation; TP: Timepoint of measurement; SC: Screening; BL: Baseline; FUP: Follow-up; D: Day; Raw: Raw values; CFB: Change from baseline; Cohort A1: 200mg oral dose niclosamide; Cohort A2: 600mg oral dose niclosamide; Cohort A3: 1600mg oral dose niclosamide; Fast/Fed: Treatment in cohort A3 was applied under fasting and fed conditions in the same subjects;

Output generated by program 'NIC002_T14_5_SafetyLaboratory_V02_0_0'

Table 14.5.2: Safety Laboratory
Haematology

Part A

Cohort	Parameter	Visit		n	Miss	Mean	SD	Minimum	Lower quartile	Median	Upper quartile	Maximum
	Haemoglobin [g/L]	SC	Raw	3	0	119.3	4.2	116	116.0	118.0	124.0	124
		BL	Raw	3	0	121.7	5.1	116	116.0	123.0	126.0	126
		D02	Raw	3	0	123.3	7.6	115	115.0	125.0	130.0	130
			CFB	3	0	1.7	11.2	-8	-8.0	-1.0	14.0	14
		FUP	Raw	3	0	115.3	6.4	108	108.0	118.0	120.0	120
			CFB	3	0	-6.3	8.5	-15	-15.0	-6.0	2.0	2
	Leucocytes [10^9/L]	SC	Raw	3	0	5.017	0.869	4.49	4.490	4.540	6.020	6.02
		BL	Raw	3	0	6.500	2.824	4.82	4.820	4.920	9.760	9.76
		D02	Raw	3	0	5.620	1.174	4.54	4.540	5.450	6.870	6.87
			CFB	3	0	-0.880	1.812	-2.89	-2.890	-0.380	0.630	0.63
		FUP	Raw	3	0	4.870	0.987	3.96	3.960	4.730	5.920	5.92
			CFB	3	0	-1.630	1.963	-3.84	-3.840	-0.960	-0.090	-0.09
	Lymphocytes, % [%]	SC	Raw	3	0	38.57	7.99	29.7	29.70	40.80	45.20	45.2
		BL	Raw	3	0	30.27	9.90	24.4	24.40	24.70	41.70	41.7
		D02	Raw	3	0	34.47	1.88	32.8	32.80	34.10	36.50	36.5
			CFB	3	0	4.20	8.16	-5.2	-5.20	8.40	9.40	9.4
		FUP	Raw	3	0	37.90	8.15	28.5	28.50	42.30	42.90	42.9
			CFB	3	0	7.63	9.32	0.6	0.60	4.10	18.20	18.2
	Lymphocytes, abs. [10^9/L]	SC	Raw	3	0	1.967	0.695	1.35	1.350	1.830	2.720	2.72
		BL	Raw	3	0	1.873	0.616	1.20	1.200	2.010	2.410	2.41
		D02	Raw	3	0	1.940	0.427	1.49	1.490	1.990	2.340	2.34

n: Number of non-missing observations; %: Percentage based on non-missing observations; Miss: Missing observations; SD: Standard deviation; TP: Timepoint of measurement; SC: Screening; BL: Baseline; FUP: Follow-up; D: Day; Raw: Raw values; CFB: Change from baseline; Cohort A1: 200mg oral dose niclosamide; Cohort A2: 600mg oral dose niclosamide; Cohort A3: 1600mg oral dose niclosamide; Fast/Fed: Treatment in cohort A3 was applied under fasting and fed conditions in the same subjects;

Output generated by program 'NIC002_T14_5_SafetyLaboratory_V02_0_0'

Table 14.5.2: Safety Laboratory
Haematology

Part A

Cohort	Parameter	Visit		n	Miss	Mean	SD	Minimum	Lower quartile	Median	Upper quartile	Maximum
	Lymphocytes, abs. [10^9/L]	D02	CFB	3	0	0.067	0.195	-0.07	-0.070	-0.020	0.290	0.29
		FUP	Raw	3	0	1.890	0.711	1.13	1.130	2.000	2.540	2.54
			CFB	3	0	0.017	0.103	-0.07	-0.070	-0.010	0.130	0.13
	Monocytes, % [%]	SC	Raw	3	0	8.13	1.70	6.2	6.20	8.80	9.40	9.4
		BL	Raw	3	0	7.93	1.20	6.7	6.70	8.00	9.10	9.1
		D02	Raw	3	0	7.47	1.68	6.4	6.40	6.60	9.40	9.4
			CFB	3	0	-0.47	1.00	-1.6	-1.60	-0.10	0.30	0.3
		FUP	Raw	3	0	8.23	2.23	6.8	6.80	7.10	10.80	10.8
			CFB	3	0	0.30	1.31	-0.9	-0.90	0.10	1.70	1.7
	Monocytes, abs. [10^9/L]	SC	Raw	3	0	0.410	0.125	0.28	0.280	0.420	0.530	0.53
		BL	Raw	3	0	0.517	0.235	0.33	0.330	0.440	0.780	0.78
		D02	Raw	3	0	0.417	0.107	0.30	0.300	0.440	0.510	0.51
			CFB	3	0	-0.100	0.214	-0.34	-0.340	-0.030	0.070	0.07
		FUP	Raw	3	0	0.400	0.121	0.27	0.270	0.420	0.510	0.51
			CFB	3	0	-0.117	0.221	-0.36	-0.360	-0.060	0.070	0.07
	Neutrophils, % [%]	SC	Raw	3	0	49.43	7.72	42.8	42.80	47.60	57.90	57.9
		BL	Raw	3	0	59.40	11.00	46.7	46.70	65.50	66.00	66.0
		D02	Raw	3	0	55.60	2.80	52.4	52.40	56.80	57.60	57.6
			CFB	3	0	-3.80	8.23	-8.7	-8.70	-8.40	5.70	5.7
		FUP	Raw	3	0	50.97	7.84	45.2	45.20	47.80	59.90	59.9
			CFB	3	0	-8.43	8.70	-18.2	-18.20	-5.60	-1.50	-1.5

n: Number of non-missing observations; %: Percentage based on non-missing observations; Miss: Missing observations; SD: Standard deviation; TP: Timepoint of measurement; SC: Screening; BL: Baseline; FUP: Follow-up; D: Day; Raw: Raw values; CFB: Change from baseline; Cohort A1: 200mg oral dose niclosamide; Cohort A2: 600mg oral dose niclosamide; Cohort A3: 1600mg oral dose niclosamide; Fast/Fed: Treatment in cohort A3 was applied under fasting and fed conditions in the same subjects;

Output generated by program 'NIC002_T14_5_SafetyLaboratory_V02_0_0'

Table 14.5.2: Safety Laboratory
Haematology

Part A

Cohort	Parameter	Visit		n	Miss	Mean	SD	Minimum	Lower quartile	Median	Upper quartile	Maximum
	Neutrophils, abs. [10^9/L]	SC	Raw	3	0	2.450	0.270	2.14	2.140	2.580	2.630	2.63
		BL	Raw	3	0	3.970	2.193	2.25	2.250	3.220	6.440	6.44
		D02	Raw	3	0	3.133	0.729	2.58	2.580	2.860	3.960	3.96
			CFB	3	0	-0.837	1.554	-2.48	-2.480	-0.640	0.610	0.61
		FUP	Raw	3	0	2.447	0.351	2.14	2.140	2.370	2.830	2.83
			CFB	3	0	-1.523	1.845	-3.61	-3.610	-0.850	-0.110	-0.11
	Platelets [10^9/L]	SC	Raw	3	0	275.0	54.4	225	225.0	267.0	333.0	333
		BL	Raw	3	0	278.0	47.9	234	234.0	271.0	329.0	329
		D02	Raw	3	0	284.3	34.5	261	261.0	268.0	324.0	324
			CFB	3	0	6.3	24.1	-10	-10.0	-5.0	34.0	34
		FUP	Raw	3	0	277.7	45.2	244	244.0	260.0	329.0	329
			CFB	3	0	-0.3	26.5	-27	-27.0	0.0	26.0	26
Cohort A3 Fed	Basophils, % [%]	BL	Raw	3	0	0.47	0.29	0.3	0.30	0.30	0.80	0.8
		D02	Raw	3	0	0.87	0.29	0.7	0.70	0.70	1.20	1.2
			CFB	3	0	0.40	0.00	0.4	0.40	0.40	0.40	0.4
		FUP	Raw	3	0	0.87	0.12	0.8	0.80	0.80	1.00	1.0
			CFB	3	0	0.40	0.36	0.0	0.00	0.50	0.70	0.7
	Basophils, abs. [10^9/L]	BL	Raw	3	0	0.027	0.012	0.02	0.020	0.020	0.040	0.04
		D02	Raw	3	0	0.050	0.020	0.03	0.030	0.050	0.070	0.07
			CFB	3	0	0.023	0.012	0.01	0.010	0.030	0.030	0.03
		FUP	Raw	3	0	0.050	0.020	0.03	0.030	0.050	0.070	0.07
			CFB	3	0	0.023	0.023	0.01	0.010	0.010	0.050	0.05

n: Number of non-missing observations; %: Percentage based on non-missing observations; Miss: Missing observations; SD: Standard deviation; TP: Timepoint of measurement; SC: Screening; BL: Baseline; FUP: Follow-up; D: Day; Raw: Raw values; CFB: Change from baseline; Cohort A1: 200mg oral dose niclosamide; Cohort A2: 600mg oral dose niclosamide; Cohort A3: 1600mg oral dose niclosamide; Fast/Fed: Treatment in cohort A3 was applied under fasting and fed conditions in the same subjects;

Output generated by program 'NIC002_T14_5_SafetyLaboratory_V02_0_0'

Table 14.5.2: Safety Laboratory
Haematology

Part A

Cohort	Parameter	Visit		n	Miss	Mean	SD	Minimum	Lower quartile	Median	Upper quartile	Maximum
	Eosinophils, % [%]	BL	Raw	3	0	2.10	0.69	1.7	1.70	1.70	2.90	2.9
		D02	Raw	3	0	2.13	0.92	1.6	1.60	1.60	3.20	3.2
			CFB	3	0	0.03	0.23	-0.1	-0.10	-0.10	0.30	0.3
		FUP	Raw	3	0	2.37	0.29	2.2	2.20	2.20	2.70	2.7
			CFB	3	0	0.27	0.40	-0.2	-0.20	0.50	0.50	0.5
	Eosinophils, abs. [10^9/L]	BL	Raw	3	0	0.120	0.026	0.10	0.100	0.110	0.150	0.15
		D02	Raw	3	0	0.127	0.060	0.07	0.070	0.120	0.190	0.19
			CFB	3	0	0.007	0.035	-0.03	-0.030	0.010	0.040	0.04
		FUP	Raw	3	0	0.130	0.044	0.08	0.080	0.150	0.160	0.16
			CFB	3	0	0.010	0.030	-0.02	-0.020	0.010	0.040	0.04
	Erythrocytes [10^{12}/L]	BL	Raw	3	0	3.980	0.178	3.78	3.780	4.040	4.120	4.12
		D02	Raw	3	0	4.267	0.101	4.15	4.150	4.320	4.330	4.33
			CFB	3	0	0.287	0.080	0.21	0.210	0.280	0.370	0.37
		FUP	Raw	3	0	4.067	0.251	3.87	3.870	3.980	4.350	4.35
			CFB	3	0	0.087	0.225	-0.14	-0.140	0.090	0.310	0.31
	Haematocrit [L/L]	BL	Raw	3	0	0.343	0.012	0.33	0.330	0.350	0.350	0.35
		D02	Raw	3	0	0.367	0.006	0.36	0.360	0.370	0.370	0.37
			CFB	3	0	0.023	0.006	0.02	0.020	0.020	0.030	0.03
		FUP	Raw	3	0	0.343	0.023	0.33	0.330	0.330	0.370	0.37
			CFB	3	0	0.000	0.020	-0.02	-0.020	0.000	0.020	0.02

n: Number of non-missing observations; %: Percentage based on non-missing observations; Miss: Missing observations; SD: Standard deviation; TP: Timepoint of measurement; SC: Screening; BL: Baseline; FUP: Follow-up; D: Day; Raw: Raw values; CFB: Change from baseline; Cohort A1: 200mg oral dose niclosamide; Cohort A2: 600mg oral dose niclosamide; Cohort A3: 1600mg oral dose niclosamide; Fast/Fed: Treatment in cohort A3 was applied under fasting and fed conditions in the same subjects;

Output generated by program 'NIC002_T14_5_SafetyLaboratory_V02_0_0'

Table 14.5.2: Safety Laboratory
Haematology

Part A

Cohort	Parameter	Visit		n	Miss	Mean	SD	Minimum	Lower quartile	Median	Upper quartile	Maximum
	Haemoglobin [g/L]	BL	Raw	3	0	118.3	0.6	118	118.0	118.0	119.0	119
		D02	Raw	3	0	125.3	3.1	122	122.0	126.0	128.0	128
			CFB	3	0	7.0	3.0	4	4.0	7.0	10.0	10
		FUP	Raw	3	0	120.3	6.5	114	114.0	120.0	127.0	127
			CFB	3	0	2.0	6.0	-4	-4.0	2.0	8.0	8
	Leucocytes [10^9/L]	BL	Raw	3	0	5.897	0.644	5.20	5.200	6.020	6.470	6.47
		D02	Raw	3	0	5.980	1.680	4.28	4.280	6.020	7.640	7.64
			CFB	3	0	0.083	1.589	-1.74	-1.740	0.820	1.170	1.17
		FUP	Raw	3	0	5.447	1.631	3.64	3.640	5.890	6.810	6.81
			CFB	3	0	-0.450	1.681	-2.38	-2.380	0.340	0.690	0.69
	Lymphocytes, % [%]	BL	Raw	3	0	37.27	4.60	33.1	33.10	36.50	42.20	42.2
		D02	Raw	3	0	33.70	6.48	26.6	26.60	35.20	39.30	39.3
			CFB	3	0	-3.57	5.52	-7.0	-7.00	-6.50	2.80	2.8
		FUP	Raw	3	0	34.67	9.81	23.9	23.90	37.00	43.10	43.1
			CFB	3	0	-2.60	8.21	-9.2	-9.20	-5.20	6.60	6.6
	Lymphocytes, abs. [10^9/L]	BL	Raw	3	0	2.217	0.505	1.72	1.720	2.200	2.730	2.73
		D02	Raw	3	0	1.990	0.608	1.60	1.600	1.680	2.690	2.69
			CFB	3	0	-0.227	0.257	-0.52	-0.520	-0.120	-0.040	-0.04
		FUP	Raw	3	0	1.833	0.600	1.41	1.410	1.570	2.520	2.52
			CFB	3	0	-0.383	0.219	-0.63	-0.630	-0.310	-0.210	-0.21

n: Number of non-missing observations; %: Percentage based on non-missing observations; Miss: Missing observations; SD: Standard deviation; TP: Timepoint of measurement; SC: Screening; BL: Baseline; FUP: Follow-up; D: Day; Raw: Raw values; CFB: Change from baseline; Cohort A1: 200mg oral dose niclosamide; Cohort A2: 600mg oral dose niclosamide; Cohort A3: 1600mg oral dose niclosamide; Fast/Fed: Treatment in cohort A3 was applied under fasting and fed conditions in the same subjects;

Output generated by program 'NIC002_T14_5_SafetyLaboratory_V02_0_0'

Table 14.5.2: Safety Laboratory
Haematology

Part A

Cohort	Parameter	Visit		n	Miss	Mean	SD	Minimum	Lower quartile	Median	Upper quartile	Maximum
	Monocytes, % [%]	BL	Raw	3	0	8.00	1.57	6.9	6.90	7.30	9.80	9.8
		D02	Raw	3	0	7.23	1.46	6.2	6.20	6.60	8.90	8.9
			CFB	3	0	-0.77	0.42	-1.1	-1.10	-0.90	-0.30	-0.3
		FUP	Raw	3	0	8.27	2.37	6.8	6.80	7.00	11.00	11.0
			CFB	3	0	0.27	0.86	-0.5	-0.50	0.10	1.20	1.2
	Monocytes, abs. [10^9/L]	BL	Raw	3	0	0.473	0.115	0.36	0.360	0.470	0.590	0.59
		D02	Raw	3	0	0.417	0.047	0.38	0.380	0.400	0.470	0.47
			CFB	3	0	-0.057	0.134	-0.21	-0.210	0.000	0.040	0.04
		FUP	Raw	3	0	0.423	0.032	0.40	0.400	0.410	0.460	0.46
			CFB	3	0	-0.050	0.125	-0.19	-0.190	-0.010	0.050	0.05
	Neutrophils, % [%]	BL	Raw	3	0	52.17	3.92	48.5	48.50	51.70	56.30	56.3
		D02	Raw	3	0	56.07	6.45	49.5	49.50	56.30	62.40	62.4
			CFB	3	0	3.90	5.35	-2.2	-2.20	6.10	7.80	7.8
		FUP	Raw	3	0	53.83	11.37	42.9	42.90	53.00	65.60	65.6
			CFB	3	0	1.67	9.38	-8.8	-8.80	4.50	9.30	9.3
	Neutrophils, abs. [10^9/L]	BL	Raw	3	0	3.060	0.114	2.93	2.930	3.110	3.140	3.14
		D02	Raw	3	0	3.397	1.139	2.12	2.120	3.760	4.310	4.31
			CFB	3	0	0.337	1.161	-0.99	-0.990	0.830	1.170	1.17
		FUP	Raw	3	0	3.010	1.262	1.56	1.560	3.610	3.860	3.86
			CFB	3	0	-0.050	1.319	-1.55	-1.550	0.470	0.930	0.93

n: Number of non-missing observations; %: Percentage based on non-missing observations; Miss: Missing observations; SD: Standard deviation; TP: Timepoint of measurement; SC: Screening; BL: Baseline; FUP: Follow-up; D: Day; Raw: Raw values; CFB: Change from baseline; Cohort A1: 200mg oral dose niclosamide; Cohort A2: 600mg oral dose niclosamide; Cohort A3: 1600mg oral dose niclosamide; Fast/Fed: Treatment in cohort A3 was applied under fasting and fed conditions in the same subjects;

Output generated by program 'NIC002_T14_5_SafetyLaboratory_V02_0_0'

Table 14.5.2: Safety Laboratory
Haematology

Part A

Cohort	Parameter	Visit		n	Miss	Mean	SD	Minimum	Lower quartile	Median	Upper quartile	Maximum
	Platelets [10^9/L]	BL	Raw	3	0	306.3	86.0	247	247.0	267.0	405.0	405
		D02	Raw	3	0	286.7	94.8	228	228.0	236.0	396.0	396
			CFB	3	0	-19.7	11.0	-31	-31.0	-19.0	-9.0	-9
		FUP	Raw	3	0	302.3	79.3	227	227.0	295.0	385.0	385
			CFB	3	0	-4.0	27.7	-20	-20.0	-20.0	28.0	28
Placebo	Basophils, % [%]	SC	Raw	3	0	0.60	0.35	0.2	0.20	0.80	0.80	0.8
		BL	Raw	3	0	0.60	0.36	0.2	0.20	0.70	0.90	0.9
		D02	Raw	3	0	0.67	0.57	0.2	0.20	0.50	1.30	1.3
			CFB	3	0	0.07	0.50	-0.4	-0.40	0.00	0.60	0.6
		FUP	Raw	3	0	0.80	0.30	0.5	0.50	0.80	1.10	1.1
			CFB	3	0	0.20	0.26	-0.1	-0.10	0.30	0.40	0.4
	Basophils, abs. [10^9/L]	SC	Raw	3	0	0.033	0.025	0.01	0.010	0.030	0.060	0.06
		BL	Raw	3	0	0.033	0.025	0.01	0.010	0.030	0.060	0.06
		D02	Raw	3	0	0.033	0.021	0.01	0.010	0.040	0.050	0.05
			CFB	3	0	0.000	0.020	-0.02	-0.020	0.000	0.020	0.02
		FUP	Raw	3	0	0.037	0.021	0.02	0.020	0.030	0.060	0.06
			CFB	3	0	0.003	0.006	0.00	0.000	0.000	0.010	0.01
	Eosinophils, % [%]	SC	Raw	3	0	3.17	1.55	1.9	1.90	2.70	4.90	4.9
		BL	Raw	3	0	3.63	1.69	2.2	2.20	3.20	5.50	5.5
		D02	Raw	3	0	4.03	1.59	2.7	2.70	3.60	5.80	5.8
			CFB	3	0	0.40	0.10	0.3	0.30	0.40	0.50	0.5
		FUP	Raw	3	0	4.63	1.18	3.9	3.90	4.00	6.00	6.0

n: Number of non-missing observations; %: Percentage based on non-missing observations; Miss: Missing observations; SD: Standard deviation; TP: Timepoint of measurement; SC: Screening; BL: Baseline; FUP: Follow-up; D: Day; Raw: Raw values; CFB: Change from baseline; Cohort A1: 200mg oral dose niclosamide; Cohort A2: 600mg oral dose niclosamide; Cohort A3: 1600mg oral dose niclosamide; Fast/Fed: Treatment in cohort A3 was applied under fasting and fed conditions in the same subjects;

Output generated by program 'NIC002_T14_5_SafetyLaboratory_V02_0_0'

Table 14.5.2: Safety Laboratory
Haematology

Part A

Cohort	Parameter	Visit		n	Miss	Mean	SD	Minimum	Lower quartile	Median	Upper quartile	Maximum
	Eosinophils, % [%]	FUP	CFB	3	0	1.00	0.70	0.5	0.50	0.70	1.80	1.8
	Eosinophils, abs. [10^9/L]	SC	Raw	3	0	0.177	0.151	0.07	0.070	0.110	0.350	0.35
		BL	Raw	3	0	0.207	0.150	0.11	0.110	0.130	0.380	0.38
		D02	Raw	3	0	0.227	0.177	0.11	0.110	0.140	0.430	0.43
			CFB	3	0	0.020	0.026	0.00	0.000	0.010	0.050	0.05
		FUP	Raw	3	0	0.240	0.192	0.11	0.110	0.150	0.460	0.46
			CFB	3	0	0.033	0.050	-0.02	-0.020	0.040	0.080	0.08
	Erythrocytes [10^{12}/L]	SC	Raw	3	0	4.110	0.195	3.91	3.910	4.120	4.300	4.30
		BL	Raw	3	0	4.133	0.178	3.94	3.940	4.170	4.290	4.29
		D02	Raw	3	0	4.137	0.179	3.94	3.940	4.180	4.290	4.29
			CFB	3	0	0.003	0.006	0.00	0.000	0.000	0.010	0.01
		FUP	Raw	3	0	4.017	0.287	3.70	3.700	4.090	4.260	4.26
			CFB	3	0	-0.117	0.180	-0.24	-0.240	-0.200	0.090	0.09
	Haematocrit [L/L]	SC	Raw	3	0	0.370	0.010	0.36	0.360	0.370	0.380	0.38
		BL	Raw	3	0	0.370	0.010	0.36	0.360	0.370	0.380	0.38
		D02	Raw	3	0	0.370	0.010	0.36	0.360	0.370	0.380	0.38
			CFB	3	0	0.000	0.000	0.00	0.000	0.000	0.000	0.00
		FUP	Raw	3	0	0.360	0.020	0.34	0.340	0.360	0.380	0.38
			CFB	3	0	-0.010	0.017	-0.02	-0.020	-0.020	0.010	0.01
	Haemoglobin [g/L]	SC	Raw	3	0	126.0	3.0	123	123.0	126.0	129.0	129
		BL	Raw	3	0	126.7	2.5	124	124.0	127.0	129.0	129

n: Number of non-missing observations; %: Percentage based on non-missing observations; Miss: Missing observations; SD: Standard deviation; TP: Timepoint of measurement; SC: Screening; BL: Baseline; FUP: Follow-up; D: Day; Raw: Raw values; CFB: Change from baseline; Cohort A1: 200mg oral dose niclosamide; Cohort A2: 600mg oral dose niclosamide; Cohort A3: 1600mg oral dose niclosamide; Fast/Fed: Treatment in cohort A3 was applied under fasting and fed conditions in the same subjects;

Output generated by program 'NIC002_T14_5_SafetyLaboratory_V02_0_0'

Table 14.5.2: Safety Laboratory
Haematology

Part A

Cohort	Parameter	Visit		n	Miss	Mean	SD	Minimum	Lower quartile	Median	Upper quartile	Maximum
	Haemoglobin [g/L]	D02	Raw	3	0	126.3	2.5	124	124.0	126.0	129.0	129
			CFB	3	0	-0.3	0.6	-1	-1.0	0.0	0.0	0
		FUP	Raw	3	0	124.0	3.6	120	120.0	125.0	127.0	127
			CFB	3	0	-2.7	5.1	-7	-7.0	-4.0	3.0	3
	Leucocytes [10^9/L]	SC	Raw	3	0	4.930	1.902	3.61	3.610	4.070	7.110	7.11
		BL	Raw	3	0	5.293	1.436	4.02	4.020	5.010	6.850	6.85
		D02	Raw	3	0	5.127	1.938	3.89	3.890	4.130	7.360	7.36
			CFB	3	0	-0.167	0.696	-0.88	-0.880	-0.130	0.510	0.51
		FUP	Raw	3	0	4.730	2.563	2.81	2.810	3.740	7.640	7.64
			CFB	3	0	-0.563	1.172	-1.27	-1.270	-1.210	0.790	0.79
	Lymphocytes, % [%]	SC	Raw	3	0	33.90	3.11	30.5	30.50	34.60	36.60	36.6
		BL	Raw	3	0	28.77	3.57	26.0	26.00	27.50	32.80	32.8
		D02	Raw	3	0	35.40	7.86	26.5	26.50	38.30	41.40	41.4
			CFB	3	0	6.63	5.42	0.5	0.50	8.60	10.80	10.8
		FUP	Raw	3	0	36.70	9.52	25.8	25.80	40.90	43.40	43.4
			CFB	3	0	7.93	7.18	-0.2	-0.20	10.60	13.40	13.4
	Lymphocytes, abs. [10^9/L]	SC	Raw	3	0	1.633	0.467	1.32	1.320	1.410	2.170	2.17
		BL	Raw	3	0	1.493	0.250	1.32	1.320	1.380	1.780	1.78
		D02	Raw	3	0	1.713	0.206	1.58	1.580	1.610	1.950	1.95
			CFB	3	0	0.220	0.062	0.17	0.170	0.200	0.290	0.29
		FUP	Raw	3	0	1.573	0.377	1.22	1.220	1.530	1.970	1.97
			CFB	3	0	0.080	0.157	-0.10	-0.100	0.150	0.190	0.19

n: Number of non-missing observations; %: Percentage based on non-missing observations; Miss: Missing observations; SD: Standard deviation; TP: Timepoint of measurement; SC: Screening; BL: Baseline; FUP: Follow-up; D: Day; Raw: Raw values; CFB: Change from baseline; Cohort A1: 200mg oral dose niclosamide; Cohort A2: 600mg oral dose niclosamide; Cohort A3: 1600mg oral dose niclosamide; Fast/Fed: Treatment in cohort A3 was applied under fasting and fed conditions in the same subjects;

Output generated by program 'NIC002_T14_5_SafetyLaboratory_V02_0_0'

Table 14.5.2: Safety Laboratory
Haematology

Part A

Cohort	Parameter	Visit		n	Miss	Mean	SD	Minimum	Lower quartile	Median	Upper quartile	Maximum
	Monocytes, % [%]	SC	Raw	3	0	10.47	1.88	8.3	8.30	11.50	11.60	11.6
		BL	Raw	3	0	8.63	1.78	6.7	6.70	9.00	10.20	10.2
		D02	Raw	3	0	9.23	1.96	7.2	7.20	9.40	11.10	11.1
			CFB	3	0	0.60	0.26	0.4	0.40	0.50	0.90	0.9
		FUP	Raw	3	0	9.93	3.92	6.5	6.50	9.10	14.20	14.2
			CFB	3	0	1.30	2.34	-0.2	-0.20	0.10	4.00	4.0
	Monocytes, abs. [10^9/L]	SC	Raw	3	0	0.493	0.087	0.42	0.420	0.470	0.590	0.59
		BL	Raw	3	0	0.440	0.026	0.41	0.410	0.450	0.460	0.46
		D02	Raw	3	0	0.450	0.072	0.39	0.390	0.430	0.530	0.53
			CFB	3	0	0.010	0.066	-0.06	-0.060	0.020	0.070	0.07
		FUP	Raw	3	0	0.413	0.081	0.34	0.340	0.400	0.500	0.50
			CFB	3	0	-0.027	0.076	-0.11	-0.110	-0.010	0.040	0.04
	Neutrophils, % [%]	SC	Raw	3	0	51.87	3.29	49.1	49.10	51.00	55.50	55.5
		BL	Raw	3	0	58.37	4.56	53.1	53.10	60.90	61.10	61.1
		D02	Raw	3	0	50.67	8.77	42.6	42.60	49.40	60.00	60.0
			CFB	3	0	-7.70	5.92	-11.7	-11.70	-10.50	-0.90	-0.9
		FUP	Raw	3	0	47.93	11.94	37.4	37.40	45.50	60.90	60.9
			CFB	3	0	-10.43	9.04	-15.7	-15.70	-15.60	0.00	0.0
	Neutrophils, abs. [10^9/L]	SC	Raw	3	0	2.593	1.176	1.77	1.770	2.070	3.940	3.94
		BL	Raw	3	0	3.120	1.021	2.13	2.130	3.060	4.170	4.17
		D02	Raw	3	0	2.703	1.490	1.66	1.660	2.040	4.410	4.41
			CFB	3	0	-0.417	0.632	-1.02	-1.020	-0.470	0.240	0.24

n: Number of non-missing observations; %: Percentage based on non-missing observations; Miss: Missing observations; SD: Standard deviation; TP: Timepoint of measurement; SC: Screening; BL: Baseline; FUP: Follow-up; D: Day; Raw: Raw values; CFB: Change from baseline; Cohort A1: 200mg oral dose niclosamide; Cohort A2: 600mg oral dose niclosamide; Cohort A3: 1600mg oral dose niclosamide; Fast/Fed: Treatment in cohort A3 was applied under fasting and fed conditions in the same subjects;

Output generated by program 'NIC002_T14_5_SafetyLaboratory_V02_0_0'

Table 14.5.2: Safety Laboratory
Haematology

Part A

Cohort	Parameter	Visit		n	Miss	Mean	SD	Minimum	Lower quartile	Median	Upper quartile	Maximum
	Neutrophils, abs. [10^9/L]	FUP	Raw	3	0	2.467	1.919	1.05	1.050	1.700	4.650	4.65
			CFB	3	0	-0.653	0.991	-1.36	-1.360	-1.080	0.480	0.48
	Platelets [10^9/L]	SC	Raw	3	0	255.7	83.2	167	167.0	268.0	332.0	332
		BL	Raw	3	0	256.7	70.4	176	176.0	288.0	306.0	306
		D02	Raw	3	0	260.0	57.4	196	196.0	277.0	307.0	307
			CFB	3	0	3.3	15.6	-11	-11.0	1.0	20.0	20
		FUP	Raw	3	0	249.3	64.0	179	179.0	265.0	304.0	304
			CFB	3	0	-7.3	13.8	-23	-23.0	-2.0	3.0	3
Placebo Fed	Basophils, % [%]	BL	Raw	1	0	0.70		0.7	0.70	0.70	0.70	0.7
		D02	Raw	1	0	0.80		0.8	0.80	0.80	0.80	0.8
			CFB	1	0	0.10		0.1	0.10	0.10	0.10	0.1
		FUP	Raw	1	0	0.90		0.9	0.90	0.90	0.90	0.9
			CFB	1	0	0.20		0.2	0.20	0.20	0.20	0.2
	Basophils, abs. [10^9/L]	BL	Raw	1	0	0.050		0.05	0.050	0.050	0.050	0.05
		D02	Raw	1	0	0.050		0.05	0.050	0.050	0.050	0.05
			CFB	1	0	0.000		0.00	0.000	0.000	0.000	0.00
		FUP	Raw	1	0	0.070		0.07	0.070	0.070	0.070	0.07
			CFB	1	0	0.020		0.02	0.020	0.020	0.020	0.02
	Eosinophils, % [%]	BL	Raw	1	0	5.40		5.4	5.40	5.40	5.40	5.4
		D02	Raw	1	0	6.80		6.8	6.80	6.80	6.80	6.8
			CFB	1	0	1.40		1.4	1.40	1.40	1.40	1.4

n: Number of non-missing observations; %: Percentage based on non-missing observations; Miss: Missing observations; SD: Standard deviation; TP: Timepoint of measurement; SC: Screening; BL: Baseline; FUP: Follow-up; D: Day; Raw: Raw values; CFB: Change from baseline; Cohort A1: 200mg oral dose niclosamide; Cohort A2: 600mg oral dose niclosamide; Cohort A3: 1600mg oral dose niclosamide; Fast/Fed: Treatment in cohort A3 was applied under fasting and fed conditions in the same subjects;

Output generated by program 'NIC002_T14_5_SafetyLaboratory_V02_0_0'

Table 14.5.2: Safety Laboratory
Haematology

Part A

Cohort	Parameter	Visit		n	Miss	Mean	SD	Minimum	Lower quartile	Median	Upper quartile	Maximum
	Eosinophils, % [%]	FUP	Raw	1	0	7.50		7.5	7.50	7.50	7.50	7.5
			CFB	1	0	2.10		2.1	2.10	2.10	2.10	2.1
	Eosinophils, abs. [10^9/L]	BL	Raw	1	0	0.400		0.40	0.400	0.400	0.400	0.40
		D02	Raw	1	0	0.400		0.40	0.400	0.400	0.400	0.40
			CFB	1	0	0.000		0.00	0.000	0.000	0.000	0.00
		FUP	Raw	1	0	0.550		0.55	0.550	0.550	0.550	0.55
			CFB	1	0	0.150		0.15	0.150	0.150	0.150	0.15
	Erythrocytes [10^{12}/L]	BL	Raw	1	0	4.200		4.20	4.200	4.200	4.200	4.20
		D02	Raw	1	0	4.180		4.18	4.180	4.180	4.180	4.18
			CFB	1	0	-0.020		-0.02	-0.020	-0.020	-0.020	-0.02
		FUP	Raw	1	0	4.370		4.37	4.370	4.370	4.370	4.37
			CFB	1	0	0.170		0.17	0.170	0.170	0.170	0.17
	Haematocrit [L/L]	BL	Raw	1	0	0.370		0.37	0.370	0.370	0.370	0.37
		D02	Raw	1	0	0.370		0.37	0.370	0.370	0.370	0.37
			CFB	1	0	0.000		0.00	0.000	0.000	0.000	0.00
		FUP	Raw	1	0	0.380		0.38	0.380	0.380	0.380	0.38
			CFB	1	0	0.010		0.01	0.010	0.010	0.010	0.01
	Haemoglobin [g/L]	BL	Raw	1	0	125.0		125	125.0	125.0	125.0	125
		D02	Raw	1	0	126.0		126	126.0	126.0	126.0	126
			CFB	1	0	1.0		1	1.0	1.0	1.0	1
		FUP	Raw	1	0	128.0		128	128.0	128.0	128.0	128

n: Number of non-missing observations; %: Percentage based on non-missing observations; Miss: Missing observations; SD: Standard deviation; TP: Timepoint of measurement; SC: Screening; BL: Baseline; FUP: Follow-up; D: Day; Raw: Raw values; CFB: Change from baseline; Cohort A1: 200mg oral dose niclosamide; Cohort A2: 600mg oral dose niclosamide; Cohort A3: 1600mg oral dose niclosamide; Fast/Fed: Treatment in cohort A3 was applied under fasting and fed conditions in the same subjects;

Output generated by program 'NIC002_T14_5_SafetyLaboratory_V02_0_0'

Table 14.5.2: Safety Laboratory
Haematology

Part A

Cohort	Parameter	Visit		n	Miss	Mean	SD	Minimum	Lower quartile	Median	Upper quartile	Maximum
	Haemoglobin [g/L]	FUP	CFB	1	0	3.0		3	3.0	3.0	3.0	3
	Leucocytes [10^9/L]	BL	Raw	1	0	7.410		7.41	7.410	7.410	7.410	7.41
		D02	Raw	1	0	5.920		5.92	5.920	5.920	5.920	5.92
			CFB	1	0	-1.490		-1.49	-1.490	-1.490	-1.490	-1.49
		FUP	Raw	1	0	7.370		7.37	7.370	7.370	7.370	7.37
			CFB	1	0	-0.040		-0.04	-0.040	-0.040	-0.040	-0.04
	Lymphocytes, % [%]	BL	Raw	1	0	25.60		25.6	25.60	25.60	25.60	25.6
		D02	Raw	1	0	30.60		30.6	30.60	30.60	30.60	30.6
			CFB	1	0	5.00		5.0	5.00	5.00	5.00	5.0
		FUP	Raw	1	0	32.40		32.4	32.40	32.40	32.40	32.4
			CFB	1	0	6.80		6.8	6.80	6.80	6.80	6.8
	Lymphocytes, abs. [10^9/L]	BL	Raw	1	0	1.900		1.90	1.900	1.900	1.900	1.90
		D02	Raw	1	0	1.810		1.81	1.810	1.810	1.810	1.81
			CFB	1	0	-0.090		-0.09	-0.090	-0.090	-0.090	-0.09
		FUP	Raw	1	0	2.390		2.39	2.390	2.390	2.390	2.39
			CFB	1	0	0.490		0.49	0.490	0.490	0.490	0.49
	Monocytes, % [%]	BL	Raw	1	0	8.20		8.2	8.20	8.20	8.20	8.2
		D02	Raw	1	0	6.90		6.9	6.90	6.90	6.90	6.9
			CFB	1	0	-1.30		-1.3	-1.30	-1.30	-1.30	-1.3
		FUP	Raw	1	0	6.60		6.6	6.60	6.60	6.60	6.6
			CFB	1	0	-1.60		-1.6	-1.60	-1.60	-1.60	-1.6

n: Number of non-missing observations; %: Percentage based on non-missing observations; Miss: Missing observations; SD: Standard deviation; TP: Timepoint of measurement; SC: Screening; BL: Baseline; FUP: Follow-up; D: Day; Raw: Raw values; CFB: Change from baseline; Cohort A1: 200mg oral dose niclosamide; Cohort A2: 600mg oral dose niclosamide; Cohort A3: 1600mg oral dose niclosamide; Fast/Fed: Treatment in cohort A3 was applied under fasting and fed conditions in the same subjects;

Output generated by program 'NIC002_T14_5_SafetyLaboratory_V02_0_0'

Table 14.5.2: Safety Laboratory
Haematology

Part A

Cohort	Parameter	Visit		n	Miss	Mean	SD	Minimum	Lower quartile	Median	Upper quartile	Maximum
	Monocytes, abs. [10^9/L]	BL	Raw	1	0	0.610		0.61	0.610	0.610	0.610	0.61
		D02	Raw	1	0	0.410		0.41	0.410	0.410	0.410	0.41
			CFB	1	0	-0.200		-0.20	-0.200	-0.200	-0.200	-0.20
		FUP	Raw	1	0	0.490		0.49	0.490	0.490	0.490	0.49
			CFB	1	0	-0.120		-0.12	-0.120	-0.120	-0.120	-0.12
	Neutrophils, % [%]	BL	Raw	1	0	60.10		60.1	60.10	60.10	60.10	60.1
		D02	Raw	1	0	54.90		54.9	54.90	54.90	54.90	54.9
			CFB	1	0	-5.20		-5.2	-5.20	-5.20	-5.20	-5.2
		FUP	Raw	1	0	52.60		52.6	52.60	52.60	52.60	52.6
			CFB	1	0	-7.50		-7.5	-7.50	-7.50	-7.50	-7.5
	Neutrophils, abs. [10^9/L]	BL	Raw	1	0	4.450		4.45	4.450	4.450	4.450	4.45
		D02	Raw	1	0	3.250		3.25	3.250	3.250	3.250	3.25
			CFB	1	0	-1.200		-1.20	-1.200	-1.200	-1.200	-1.20
		FUP	Raw	1	0	3.870		3.87	3.870	3.870	3.870	3.87
			CFB	1	0	-0.580		-0.58	-0.580	-0.580	-0.580	-0.58
	Platelets [10^9/L]	BL	Raw	1	0	273.0		273	273.0	273.0	273.0	273
		D02	Raw	1	0	271.0		271	271.0	271.0	271.0	271
			CFB	1	0	-2.0		-2	-2.0	-2.0	-2.0	-2
		FUP	Raw	1	0	314.0		314	314.0	314.0	314.0	314
			CFB	1	0	41.0		41	41.0	41.0	41.0	41

n: Number of non-missing observations; %: Percentage based on non-missing observations; Miss: Missing observations; SD: Standard deviation; TP: Timepoint of measurement; SC: Screening; BL: Baseline; FUP: Follow-up; D: Day; Raw: Raw values; CFB: Change from baseline; Cohort A1: 200mg oral dose niclosamide; Cohort A2: 600mg oral dose niclosamide; Cohort A3: 1600mg oral dose niclosamide; Fast/Fed: Treatment in cohort A3 was applied under fasting and fed conditions in the same subjects;

Output generated by program 'NIC002_T14_5_SafetyLaboratory_V02_0_0'

Table 14.5.2: Safety Laboratory
Haematology

Part B

Parameter	Visit	Treatment		n	Miss	Mean	SD	Minimum	Lower quartile	Median	Upper quartile	Maximum
Basophils, % [%]	SC		Raw	4	0	0.58	0.15	0.5	0.50	0.50	0.65	0.8
	BL		Raw	4	0	0.55	0.13	0.4	0.45	0.55	0.65	0.7
	D02	Chewing tablet 2000 mg	Raw	4	0	0.45	0.29	0.1	0.25	0.45	0.65	0.8
			CFB	4	0	-0.10	0.18	-0.3	-0.25	-0.10	0.05	0.1
		Solution 1600 mg	Raw	4	0	0.53	0.19	0.4	0.40	0.45	0.65	0.8
			CFB	4	0	-0.02	0.10	-0.1	-0.10	-0.05	0.05	0.1
	FUP		Raw	4	0	0.58	0.15	0.4	0.45	0.60	0.70	0.7
			CFB	4	0	0.03	0.05	0.0	0.00	0.00	0.05	0.1
Basophils, abs. [10^9/L]	SC		Raw	4	0	0.030	0.008	0.02	0.025	0.030	0.035	0.04
	BL		Raw	4	0	0.033	0.005	0.03	0.030	0.030	0.035	0.04
	D02	Chewing tablet 2000 mg	Raw	4	0	0.025	0.013	0.01	0.015	0.025	0.035	0.04
			CFB	4	0	-0.008	0.010	-0.02	-0.015	-0.005	0.000	0.00
		Solution 1600 mg	Raw	4	0	0.033	0.013	0.02	0.025	0.030	0.040	0.05
			CFB	4	0	0.000	0.008	-0.01	-0.005	0.000	0.005	0.01
	FUP		Raw	4	0	0.030	0.000	0.03	0.030	0.030	0.030	0.03
			CFB	4	0	-0.003	0.005	-0.01	-0.005	0.000	0.000	0.00
Eosinophils, % [%]	SC		Raw	4	0	2.73	1.17	1.6	1.80	2.55	3.65	4.2
	BL		Raw	4	0	3.63	2.09	1.7	2.35	3.10	4.90	6.6
	D02	Chewing tablet 2000 mg	Raw	4	0	2.93	1.72	1.5	1.80	2.40	4.05	5.4
			CFB	4	0	-0.70	0.52	-1.2	-1.15	-0.70	-0.25	-0.2

n: Number of non-missing observations; %: Percentage based on non-missing observations; Miss: Missing observations; SD: Standard deviation; TP: Timepoint of measurement; SC: Screening; BL: Baseline; FUP: Follow-up; D: Day; Raw: Raw values; CFB: Change from baseline; Part B used a cross-over design;

Output generated by program 'NIC002_T14_5_SafetyLaboratory_V02_0_0'

Table 14.5.2: Safety Laboratory
Haematology

Part B

Parameter	Visit	Treatment		n	Miss	Mean	SD	Minimum	Lower quartile	Median	Upper quartile	Maximum
Eosinophils, % [%]		Solution 1600 mg	Raw	4	0	2.38	1.23	1.2	1.60	2.10	3.15	4.1
			CFB	4	0	-1.25	0.88	-2.5	-1.85	-1.00	-0.65	-0.5
	FUP		Raw	4	0	2.88	1.66	1.6	1.85	2.30	3.90	5.3
			CFB	4	0	-0.75	0.55	-1.3	-1.20	-0.80	-0.30	-0.1
Eosinophils, abs. [10^9/L]	SC		Raw	4	0	0.155	0.083	0.09	0.095	0.130	0.215	0.27
	BL		Raw	4	0	0.230	0.169	0.11	0.130	0.165	0.330	0.48
	D02	Chewing tablet 2000 mg	Raw	4	0	0.208	0.182	0.10	0.105	0.125	0.310	0.48
			CFB	4	0	-0.022	0.021	-0.04	-0.040	-0.025	-0.005	0.00
		Solution 1600 mg	Raw	4	0	0.153	0.096	0.07	0.090	0.125	0.215	0.29
			CFB	4	0	-0.077	0.075	-0.19	-0.115	-0.040	-0.040	-0.04
	FUP		Raw	4	0	0.183	0.165	0.09	0.095	0.105	0.270	0.43
			CFB	4	0	-0.048	0.026	-0.07	-0.065	-0.055	-0.030	-0.01
Erythrocytes [10^{12}/L]	SC		Raw	4	0	4.123	0.160	3.92	4.020	4.130	4.225	4.31
	BL		Raw	4	0	4.050	0.188	3.84	3.895	4.055	4.205	4.25
	D02	Chewing tablet 2000 mg	Raw	4	0	4.123	0.108	4.00	4.045	4.115	4.200	4.26
			CFB	4	0	0.072	0.242	-0.16	-0.135	0.070	0.280	0.31
		Solution 1600 mg	Raw	4	0	4.243	0.061	4.18	4.195	4.235	4.290	4.32
			CFB	4	0	0.192	0.174	-0.04	0.060	0.235	0.325	0.34
	FUP		Raw	4	0	3.978	0.152	3.80	3.865	3.975	4.090	4.16
			CFB	4	0	-0.073	0.264	-0.23	-0.230	-0.190	0.085	0.32
Haematocrit [L/L]	SC		Raw	4	0	0.372	0.010	0.36	0.365	0.375	0.380	0.38

n: Number of non-missing observations; %: Percentage based on non-missing observations; Miss: Missing observations; SD: Standard deviation; TP: Timepoint of measurement; SC: Screening; BL: Baseline; FUP: Follow-up; D: Day; Raw: Raw values; CFB: Change from baseline; Part B used a cross-over design;

Output generated by program 'NIC002_T14_5_SafetyLaboratory_V02_0_0'

Table 14.5.2: Safety Laboratory
Haematology

Part B

Parameter	Visit	Treatment		n	Miss	Mean	SD	Minimum	Lower quartile	Median	Upper quartile	Maximum
Haematocrit [L/L]	BL		Raw	4	0	0.360	0.008	0.35	0.355	0.360	0.365	0.37
	D02	Chewing tablet 2000 mg	Raw	4	0	0.370	0.018	0.35	0.355	0.370	0.385	0.39
			CFB	4	0	0.010	0.023	-0.01	-0.010	0.010	0.030	0.03
		Solution 1600 mg	Raw	4	0	0.383	0.015	0.36	0.375	0.390	0.390	0.39
			CFB	4	0	0.023	0.017	0.00	0.010	0.025	0.035	0.04
	FUP		Raw	4	0	0.353	0.019	0.34	0.340	0.345	0.365	0.38
			CFB	4	0	-0.007	0.025	-0.02	-0.020	-0.020	0.005	0.03
Haemoglobin [g/L]	SC		Raw	4	0	127.0	2.4	125	125.0	126.5	129.0	130
	BL		Raw	4	0	125.0	4.3	121	122.0	124.0	128.0	131
	D02	Chewing tablet 2000 mg	Raw	4	0	128.8	4.8	122	125.5	130.0	132.0	133
			CFB	4	0	3.8	7.2	-3	-2.5	4.0	10.0	10
		Solution 1600 mg	Raw	4	0	132.0	6.4	123	128.0	133.5	136.0	138
			CFB	4	0	7.0	6.4	-2	2.5	9.0	11.5	12
	FUP		Raw	4	0	125.5	6.5	120	120.5	124.0	130.5	134
			CFB	4	0	0.5	8.3	-4	-4.0	-3.5	5.0	13
Leucocytes [10^9/L]	SC		Raw	4	0	5.615	0.964	4.43	4.830	5.800	6.400	6.43
	BL		Raw	4	0	6.105	1.070	4.72	5.345	6.210	6.865	7.28
	D02	Chewing tablet 2000 mg	Raw	4	0	6.520	1.732	5.24	5.260	5.955	7.780	8.93
			CFB	4	0	0.415	0.985	-0.73	-0.275	0.370	1.105	1.65
		Solution 1600 mg	Raw	4	0	6.120	0.652	5.62	5.620	5.935	6.620	6.99
			CFB	4	0	0.015	0.744	-0.83	-0.560	-0.005	0.590	0.90
	FUP		Raw	4	0	5.748	1.817	4.32	4.340	5.260	7.155	8.15

n: Number of non-missing observations; %: Percentage based on non-missing observations; Miss: Missing observations; SD: Standard deviation; TP: Timepoint of measurement; SC: Screening; BL: Baseline; FUP: Follow-up; D: Day; Raw: Raw values; CFB: Change from baseline; Part B used a cross-over design;

Output generated by program 'NIC002_T14_5_SafetyLaboratory_V02_0_0'

Table 14.5.2: Safety Laboratory
Haematology

Part B

Parameter	Visit	Treatment		n	Miss	Mean	SD	Minimum	Lower quartile	Median	Upper quartile	Maximum
Leucocytes [10^9/L]	FUP		CFB	4	0	-0.357	1.014	-1.61	-1.005	-0.345	0.290	0.87
Lymphocytes, % [%]	SC		Raw	4	0	34.95	7.20	27.9	28.95	34.40	40.95	43.1
	BL		Raw	4	0	36.05	8.75	27.3	28.55	36.20	43.55	44.5
	D02	Chewing tablet 2000 mg	Raw	4	0	36.90	7.45	27.5	31.85	37.25	41.95	45.6
			CFB	4	0	0.85	5.81	-4.3	-3.30	-0.60	5.00	8.9
		Solution 1600 mg	Raw	4	0	33.58	5.37	25.8	30.00	35.65	37.15	37.2
			CFB	4	0	-2.47	6.40	-7.4	-6.40	-4.70	1.45	6.9
	FUP		Raw	4	0	33.98	6.76	27.1	28.25	33.90	39.70	41.0
			CFB	4	0	-2.08	2.75	-6.1	-3.85	-1.00	-0.30	-0.2
Lymphocytes, abs. [10^9/L]	SC		Raw	4	0	1.965	0.567	1.46	1.590	1.815	2.340	2.77
	BL		Raw	4	0	2.198	0.704	1.76	1.770	1.895	2.625	3.24
	D02	Chewing tablet 2000 mg	Raw	4	0	2.483	1.130	1.44	1.730	2.210	3.235	4.07
			CFB	4	0	0.285	0.544	-0.34	-0.165	0.325	0.735	0.83
		Solution 1600 mg	Raw	4	0	2.053	0.410	1.61	1.765	2.005	2.340	2.59
			CFB	4	0	-0.145	0.365	-0.65	-0.410	-0.045	0.120	0.16
	FUP		Raw	4	0	1.963	0.807	1.28	1.475	1.720	2.450	3.13
			CFB	4	0	-0.235	0.189	-0.50	-0.370	-0.175	-0.100	-0.09
Monocytes, % [%]	SC		Raw	4	0	8.00	0.94	7.0	7.30	7.90	8.70	9.2
	BL		Raw	4	0	8.55	0.91	7.9	8.00	8.20	9.10	9.9
	D02	Chewing tablet 2000 mg	Raw	4	0	7.70	0.29	7.4	7.50	7.65	7.90	8.1
			CFB	4	0	-0.85	0.65	-1.8	-1.25	-0.60	-0.45	-0.4

n: Number of non-missing observations; %: Percentage based on non-missing observations; Miss: Missing observations; SD: Standard deviation; TP: Timepoint of measurement; SC: Screening; BL: Baseline; FUP: Follow-up; D: Day; Raw: Raw values; CFB: Change from baseline; Part B used a cross-over design;

Output generated by program 'NIC002_T14_5_SafetyLaboratory_V02_0_0'

Table 14.5.2: Safety Laboratory
Haematology

Part B

Parameter	Visit	Treatment		n	Miss	Mean	SD	Minimum	Lower quartile	Median	Upper quartile	Maximum
Monocytes, % [%]	D02	Solution 1600 mg	Raw	4	0	7.83	0.74	7.1	7.20	7.80	8.45	8.6
			CFB	4	0	-0.73	1.00	-1.6	-1.40	-1.00	-0.05	0.7
	FUP		Raw	4	0	8.45	0.66	7.6	8.00	8.50	8.90	9.2
			CFB	4	0	-0.10	1.14	-1.3	-1.00	-0.20	0.80	1.3
Monocytes, abs. [10^9/L]	SC		Raw	4	0	0.450	0.095	0.31	0.395	0.485	0.505	0.52
	BL		Raw	4	0	0.525	0.141	0.39	0.430	0.495	0.620	0.72
	D02	Chewing tablet 2000 mg	Raw	4	0	0.505	0.153	0.39	0.395	0.455	0.615	0.72
			CFB	4	0	-0.020	0.041	-0.08	-0.045	-0.005	0.005	0.01
		Solution 1600 mg	Raw	4	0	0.483	0.091	0.40	0.405	0.475	0.560	0.58
			CFB	4	0	-0.043	0.099	-0.14	-0.125	-0.050	0.040	0.07
	FUP		Raw	4	0	0.488	0.162	0.33	0.365	0.460	0.610	0.70
			CFB	4	0	-0.037	0.033	-0.07	-0.065	-0.040	-0.010	0.00
Neutrophils, % [%]	SC		Raw	4	0	53.75	7.09	44.6	48.15	55.35	59.35	59.7
	BL		Raw	4	0	51.23	11.16	38.6	41.95	51.95	60.50	62.4
	D02	Chewing tablet 2000 mg	Raw	4	0	52.03	8.61	40.8	46.20	52.85	57.85	61.6
			CFB	4	0	0.80	6.32	-8.3	-3.05	2.60	4.65	6.3
		Solution 1600 mg	Raw	4	0	55.70	5.37	50.1	51.65	55.05	59.75	62.6
			CFB	4	0	4.48	7.32	-5.5	-0.75	5.95	9.70	11.5
	FUP		Raw	4	0	54.13	7.35	47.3	47.95	53.40	60.30	62.4
			CFB	4	0	2.90	4.21	-0.4	-0.20	1.65	6.00	8.7
Neutrophils, abs. [10^9/L]	SC		Raw	4	0	3.015	0.628	2.29	2.580	2.980	3.450	3.81

n: Number of non-missing observations; %: Percentage based on non-missing observations; Miss: Missing observations; SD: Standard deviation; TP: Timepoint of measurement; SC: Screening; BL: Baseline; FUP: Follow-up; D: Day; Raw: Raw values; CFB: Change from baseline; Part B used a cross-over design;

Output generated by program 'NIC002_T14_5_SafetyLaboratory_V02_0_0'

Table 14.5.2: Safety Laboratory
Haematology

Part B

Parameter	Visit	Treatment		n	Miss	Mean	SD	Minimum	Lower quartile	Median	Upper quartile	Maximum
Neutrophils, abs. [10^9/L]	BL		Raw	4	0	3.120	0.822	2.14	2.475	3.155	3.765	4.03
	D02	Chewing tablet 2000 mg	Raw	4	0	3.300	0.423	2.73	2.980	3.410	3.620	3.65
			CFB	4	0	0.180	0.630	-0.44	-0.355	0.160	0.715	0.84
		Solution 1600 mg	Raw	4	0	3.400	0.399	2.99	3.095	3.350	3.705	3.91
			CFB	4	0	0.280	0.762	-0.83	-0.210	0.550	0.770	0.85
	FUP		Raw	4	0	3.085	0.901	2.10	2.320	3.190	3.850	3.86
			CFB	4	0	-0.035	0.828	-0.96	-0.575	-0.115	0.505	1.05
Platelets [10^9/L]	SC		Raw	4	0	289.0	37.2	246	258.0	293.5	320.0	323
	BL		Raw	4	0	271.0	37.9	233	245.5	264.0	296.5	323
	D02	Chewing tablet 2000 mg	Raw	4	0	290.0	46.9	241	254.5	284.5	325.5	350
			CFB	4	0	19.0	11.7	8	9.0	18.5	29.0	31
		Solution 1600 mg	Raw	4	0	299.8	23.5	278	284.5	294.0	315.0	333
			CFB	4	0	28.8	14.6	10	18.5	30.0	39.0	45
	FUP		Raw	4	0	299.5	36.0	264	268.5	301.5	330.5	331
			CFB	4	0	28.5	26.8	6	6.5	23.5	50.5	61

n: Number of non-missing observations; %: Percentage based on non-missing observations; Miss: Missing observations; SD: Standard deviation; TP: Timepoint of measurement; SC: Screening; BL: Baseline; FUP: Follow-up; D: Day; Raw: Raw values; CFB: Change from baseline; Part B used a cross-over design;

Output generated by program 'NIC002_T14_5_SafetyLaboratory_V02_0_0'

Table 14.5.2: Safety Laboratory
Haematology

Part C

Group	Parameter	Visit		n	Miss	Mean	SD	Minimum	Lower quartile	Median	Upper quartile	Maximum
Group 1	Basophils, % [%]	SC	Raw	4	0	0.70	0.45	0.3	0.35	0.60	1.05	1.3
		BL	Raw	4	0	0.53	0.22	0.2	0.40	0.60	0.65	0.7
		D03	Raw	4	0	0.60	0.42	0.2	0.35	0.50	0.85	1.2
			CFB	4	0	0.08	0.29	-0.1	-0.10	-0.05	0.25	0.5
		D08	Raw	4	0	0.63	0.26	0.4	0.45	0.55	0.80	1.0
			CFB	4	0	0.10	0.18	-0.1	-0.05	0.10	0.25	0.3
		FUP	Raw	4	0	0.68	0.10	0.6	0.60	0.65	0.75	0.8
			CFB	4	0	0.15	0.17	0.0	0.05	0.10	0.25	0.4
	Basophils, abs. [10^9/L]	SC	Raw	4	0	0.038	0.015	0.02	0.025	0.040	0.050	0.05
		BL	Raw	4	0	0.035	0.017	0.01	0.025	0.040	0.045	0.05
		D03	Raw	4	0	0.033	0.017	0.01	0.020	0.035	0.045	0.05
			CFB	4	0	-0.003	0.010	-0.01	-0.010	-0.005	0.005	0.01
		D08	Raw	4	0	0.030	0.008	0.02	0.025	0.030	0.035	0.04
			CFB	4	0	-0.005	0.010	-0.01	-0.010	-0.010	-0.000	0.01
		FUP	Raw	4	0	0.038	0.010	0.03	0.030	0.035	0.045	0.05
			CFB	4	0	0.002	0.013	-0.01	-0.005	0.000	0.010	0.02
	Eosinophils, % [%]	SC	Raw	4	0	4.30	3.34	1.3	1.45	4.05	7.15	7.8
		BL	Raw	4	0	4.13	2.81	0.9	1.80	4.35	6.45	6.9
		D03	Raw	4	0	4.30	2.77	1.2	2.00	4.45	6.60	7.1
			CFB	4	0	0.17	0.10	0.1	0.10	0.15	0.25	0.3
		D08	Raw	4	0	4.40	3.28	1.0	1.65	4.35	7.15	7.9
			CFB	4	0	0.28	1.11	-0.5	-0.45	-0.15	1.00	1.9
		FUP	Raw	4	0	5.08	3.11	1.3	2.60	5.35	7.55	8.3

n: Number of non-missing observations; %: Percentage based on non-missing observations; Miss: Missing observations; SD: Standard deviation; TP: Timepoint of measurement; SC: Screening; BL: Baseline; FUP: Follow-up; D: Day; Raw: Raw values; CFB: Change from baseline; Group 1: 1200 mg niclosamide solution; Group 2: 1600 mg niclosamide solution; Group 3: Placebo;

Output generated by program 'NIC002_T14_5_SafetyLaboratory_V02_0_0'

Table 14.5.2: Safety Laboratory
Haematology

Part C

Group	Parameter	Visit		n	Miss	Mean	SD	Minimum	Lower quartile	Median	Upper quartile	Maximum
	Eosinophils, % [%]	FUP	CFB	4	0	0.95	0.44	0.4	0.60	1.00	1.30	1.4
	Eosinophils, abs. [10^9/L]	SC	Raw	4	0	0.265	0.215	0.05	0.080	0.275	0.450	0.46
		BL	Raw	4	0	0.293	0.234	0.05	0.100	0.280	0.485	0.56
		D03	Raw	4	0	0.278	0.241	0.05	0.085	0.240	0.470	0.58
			CFB	4	0	-0.015	0.031	-0.05	-0.040	-0.015	0.010	0.02
		D08	Raw	4	0	0.270	0.227	0.03	0.080	0.270	0.460	0.51
			CFB	4	0	-0.023	0.021	-0.05	-0.035	-0.020	-0.010	0.00
		FUP	Raw	4	0	0.300	0.229	0.06	0.125	0.275	0.475	0.59
			CFB	4	0	0.007	0.040	-0.05	-0.020	0.020	0.035	0.04
	Erythrocytes [10^{12}/L]	SC	Raw	4	0	4.265	0.320	3.81	4.045	4.360	4.485	4.53
		BL	Raw	4	0	4.253	0.235	3.96	4.065	4.285	4.440	4.48
		D03	Raw	4	0	4.293	0.295	3.86	4.115	4.395	4.470	4.52
			CFB	4	0	0.040	0.176	-0.11	-0.105	0.010	0.185	0.25
		D08	Raw	4	0	4.043	0.243	3.71	3.875	4.090	4.210	4.28
			CFB	4	0	-0.210	0.105	-0.34	-0.295	-0.190	-0.125	-0.12
		FUP	Raw	4	0	4.015	0.156	3.87	3.895	3.985	4.135	4.22
			CFB	4	0	-0.238	0.108	-0.35	-0.305	-0.255	-0.170	-0.09
	Haematocrit [L/L]	SC	Raw	4	0	0.388	0.019	0.36	0.375	0.395	0.400	0.40
		BL	Raw	4	0	0.388	0.013	0.37	0.380	0.390	0.395	0.40
		D03	Raw	4	0	0.395	0.024	0.36	0.380	0.405	0.410	0.41
			CFB	4	0	0.007	0.015	-0.01	-0.005	0.010	0.020	0.02
		D08	Raw	4	0	0.370	0.014	0.35	0.360	0.375	0.380	0.38
			CFB	4	0	-0.018	0.010	-0.03	-0.025	-0.015	-0.010	-0.01

n: Number of non-missing observations; %: Percentage based on non-missing observations; Miss: Missing observations; SD: Standard deviation; TP: Timepoint of measurement; SC: Screening; BL: Baseline; FUP: Follow-up; D: Day; Raw: Raw values; CFB: Change from baseline; Group 1: 1200 mg niclosamide solution; Group 2: 1600 mg niclosamide solution; Group 3: Placebo;

Output generated by program 'NIC002_T14_5_SafetyLaboratory_V02_0_0'

Table 14.5.2: Safety Laboratory
Haematology

Part C

Group	Parameter	Visit		n	Miss	Mean	SD	Minimum	Lower quartile	Median	Upper quartile	Maximum
	Haematocrit [L/L]	FUP	Raw	4	0	0.368	0.005	0.36	0.365	0.370	0.370	0.37
			CFB	4	0	-0.020	0.014	-0.03	-0.030	-0.025	-0.010	0.00
	Haemoglobin [g/L]	SC	Raw	4	0	133.8	8.1	122	128.5	136.5	139.0	140
		BL	Raw	4	0	133.0	3.4	128	131.0	134.5	135.0	135
		D03	Raw	4	0	134.8	7.2	126	129.0	135.5	140.5	142
			CFB	4	0	1.8	5.0	-3	-2.5	1.5	6.0	7
		D08	Raw	4	0	127.3	6.8	120	121.5	127.5	133.0	134
			CFB	4	0	-5.8	5.2	-12	-10.0	-5.0	-1.5	-1
		FUP	Raw	4	0	125.8	1.5	124	124.5	126.0	127.0	127
			CFB	4	0	-7.3	3.0	-10	-9.0	-8.0	-5.5	-3
	Leucocytes [10^9/L]	SC	Raw	4	0	5.863	1.330	3.97	4.935	6.340	6.790	6.80
		BL	Raw	4	0	6.518	1.211	5.55	5.580	6.200	7.455	8.12
		D03	Raw	4	0	5.618	1.921	4.08	4.170	5.085	7.065	8.22
			CFB	4	0	-0.900	0.719	-1.53	-1.410	-1.085	-0.390	0.10
		D08	Raw	4	0	5.478	2.032	3.07	4.145	5.405	6.810	8.03
			CFB	4	0	-1.040	1.238	-2.54	-2.055	-0.830	-0.025	0.04
		FUP	Raw	4	0	5.513	1.069	4.78	4.835	5.095	6.190	7.08
			CFB	4	0	-1.005	0.359	-1.49	-1.265	-0.935	-0.745	-0.66
	Lymphocytes, % [%]	SC	Raw	4	0	34.55	7.89	26.5	28.00	34.15	41.10	43.4
		BL	Raw	4	0	35.65	4.49	31.7	32.95	34.40	38.35	42.1
		D03	Raw	4	0	38.48	6.13	33.3	33.30	37.65	43.65	45.3
			CFB	4	0	2.82	5.35	-0.9	-0.50	0.75	6.15	10.7
		D08	Raw	4	0	34.35	4.58	30.1	31.35	33.25	37.35	40.8

n: Number of non-missing observations; %: Percentage based on non-missing observations; Miss: Missing observations; SD: Standard deviation; TP: Timepoint of measurement; SC: Screening; BL: Baseline; FUP: Follow-up; D: Day; Raw: Raw values; CFB: Change from baseline; Group 1: 1200 mg niclosamide solution; Group 2: 1600 mg niclosamide solution; Group 3: Placebo;

Output generated by program 'NIC002_T14_5_SafetyLaboratory_V02_0_0'

Table 14.5.2: Safety Laboratory
Haematology

Part C

Group	Parameter	Visit		n	Miss	Mean	SD	Minimum	Lower quartile	Median	Upper quartile	Maximum
	Lymphocytes, % [%]	D08	CFB	4	0	-1.30	2.32	-4.5	-2.90	-0.80	0.30	0.9
		FUP	Raw	4	0	29.95	4.80	25.7	25.80	29.85	34.10	34.4
			CFB	4	0	-5.70	3.82	-8.3	-8.30	-7.15	-3.10	-0.2
	Lymphocytes, abs. [10^9/L]	SC	Raw	4	0	1.975	0.433	1.54	1.670	1.900	2.280	2.56
		BL	Raw	4	0	2.318	0.474	1.92	1.920	2.245	2.715	2.86
		D03	Raw	4	0	2.128	0.613	1.36	1.645	2.205	2.610	2.74
			CFB	4	0	-0.190	0.338	-0.56	-0.470	-0.185	0.090	0.17
		D08	Raw	4	0	1.868	0.672	1.04	1.360	1.905	2.375	2.62
			CFB	4	0	-0.450	0.431	-0.88	-0.805	-0.485	-0.095	0.05
		FUP	Raw	4	0	1.633	0.268	1.24	1.460	1.735	1.805	1.82
			CFB	4	0	-0.685	0.342	-1.07	-0.910	-0.715	-0.460	-0.24
	Monocytes, % [%]	SC	Raw	4	0	6.48	0.34	6.0	6.25	6.55	6.70	6.8
		BL	Raw	4	0	8.10	1.19	6.6	7.15	8.35	9.05	9.1
		D03	Raw	4	0	7.35	1.81	5.4	5.85	7.35	8.85	9.3
			CFB	4	0	-0.75	0.72	-1.4	-1.30	-0.90	-0.20	0.2
		D08	Raw	4	0	6.68	1.02	5.4	5.85	6.90	7.50	7.5
			CFB	4	0	-1.42	0.83	-2.3	-1.95	-1.55	-0.90	-0.3
		FUP	Raw	4	0	8.13	1.11	6.7	7.45	8.20	8.80	9.4
			CFB	4	0	0.02	1.85	-1.0	-0.95	-0.85	1.00	2.8
	Monocytes, abs. [10^9/L]	SC	Raw	4	0	0.383	0.099	0.24	0.315	0.415	0.450	0.46
		BL	Raw	4	0	0.530	0.138	0.43	0.440	0.480	0.620	0.73
		D03	Raw	4	0	0.415	0.189	0.27	0.295	0.350	0.535	0.69
			CFB	4	0	-0.115	0.052	-0.16	-0.145	-0.130	-0.085	-0.04

n: Number of non-missing observations; %: Percentage based on non-missing observations; Miss: Missing observations; SD: Standard deviation; TP: Timepoint of measurement; SC: Screening; BL: Baseline; FUP: Follow-up; D: Day; Raw: Raw values; CFB: Change from baseline; Group 1: 1200 mg niclosamide solution; Group 2: 1600 mg niclosamide solution; Group 3: Placebo;

Output generated by program 'NIC002_T14_5_SafetyLaboratory_V02_0_0'

Table 14.5.2: Safety Laboratory
Haematology

Part C

Group	Parameter	Visit		n	Miss	Mean	SD	Minimum	Lower quartile	Median	Upper quartile	Maximum
	Monocytes, abs. [10^9/L]	D08	Raw	4	0	0.365	0.162	0.23	0.265	0.315	0.465	0.60
			CFB	4	0	-0.165	0.077	-0.28	-0.205	-0.130	-0.125	-0.12
		FUP	Raw	4	0	0.450	0.112	0.33	0.360	0.445	0.540	0.58
			CFB	4	0	-0.080	0.089	-0.15	-0.135	-0.110	-0.025	0.05
	Neutrophils, % [%]	SC	Raw	4	0	53.98	9.31	41.4	47.00	56.20	60.95	62.1
		BL	Raw	4	0	51.60	4.84	44.7	48.25	53.30	54.95	55.1
		D03	Raw	4	0	49.28	4.49	45.4	45.70	48.35	52.85	55.0
			CFB	4	0	-2.32	4.82	-9.4	-5.25	-0.60	0.60	1.3
		D08	Raw	4	0	53.95	7.32	44.4	48.70	54.80	59.20	61.8
			CFB	4	0	2.35	3.20	-0.3	0.45	1.35	4.25	7.0
		FUP	Raw	4	0	56.18	6.00	49.4	51.90	55.75	60.45	63.8
			CFB	4	0	4.58	3.76	-0.4	2.15	5.00	7.00	8.7
	Neutrophils, abs. [10^9/L]	SC	Raw	4	0	3.203	1.093	2.09	2.265	3.255	4.140	4.21
		BL	Raw	4	0	3.343	0.579	3.03	3.035	3.065	3.650	4.21
		D03	Raw	4	0	2.765	0.991	1.93	2.085	2.480	3.445	4.17
			CFB	4	0	-0.577	0.489	-1.11	-0.980	-0.580	-0.175	-0.04
		D08	Raw	4	0	2.945	1.131	1.74	2.030	2.890	3.860	4.26
			CFB	4	0	-0.398	0.790	-1.35	-1.030	-0.330	0.235	0.42
		FUP	Raw	4	0	3.093	0.661	2.62	2.640	2.855	3.545	4.04
			CFB	4	0	-0.250	0.176	-0.41	-0.395	-0.275	-0.105	-0.04
	Platelets [10^9/L]	SC	Raw	4	0	294.8	18.5	277	281.5	291.0	308.0	320
		BL	Raw	4	0	280.8	19.1	256	267.5	282.5	294.0	302
		D03	Raw	4	0	271.5	30.1	230	250.0	278.5	293.0	299

n: Number of non-missing observations; %: Percentage based on non-missing observations; Miss: Missing observations; SD: Standard deviation; TP: Timepoint of measurement; SC: Screening; BL: Baseline; FUP: Follow-up; D: Day; Raw: Raw values; CFB: Change from baseline; Group 1: 1200 mg niclosamide solution; Group 2: 1600 mg niclosamide solution; Group 3: Placebo;

Output generated by program 'NIC002_T14_5_SafetyLaboratory_V02_0_0'

Table 14.5.2: Safety Laboratory
Haematology

Part C

Group	Parameter	Visit		n	Miss	Mean	SD	Minimum	Lower quartile	Median	Upper quartile	Maximum
	Platelets [10^9/L]	D03	CFB	4	0	-9.3	16.4	-26	-20.5	-12.0	2.0	13
		D08	Raw	4	0	243.0	41.2	197	216.5	239.0	269.5	297
			CFB	4	0	-37.8	33.4	-60	-59.5	-51.0	-16.0	11
		FUP	Raw	4	0	251.0	46.9	210	217.5	239.0	284.5	316
			CFB	4	0	-29.8	40.0	-54	-51.5	-47.5	-8.0	30
Group 2	Basophils, % [%]	SC	Raw	4	0	0.63	0.19	0.5	0.50	0.55	0.75	0.9
		BL	Raw	4	0	0.53	0.15	0.4	0.40	0.50	0.65	0.7
		D03	Raw	4	0	0.55	0.19	0.3	0.40	0.60	0.70	0.7
			CFB	4	0	0.02	0.10	-0.1	-0.05	0.05	0.10	0.1
		D08	Raw	4	0	0.65	0.19	0.4	0.50	0.70	0.80	0.8
			CFB	4	0	0.13	0.22	-0.1	-0.05	0.10	0.30	0.4
		FUP	Raw	4	0	0.65	0.30	0.4	0.40	0.60	0.90	1.0
			CFB	4	0	0.13	0.15	0.0	0.00	0.10	0.25	0.3
	Basophils, abs. [10^9/L]	SC	Raw	4	0	0.038	0.015	0.03	0.030	0.030	0.045	0.06
		BL	Raw	4	0	0.035	0.013	0.02	0.025	0.035	0.045	0.05
		D03	Raw	4	0	0.033	0.015	0.02	0.020	0.030	0.045	0.05
			CFB	4	0	-0.003	0.010	-0.01	-0.010	-0.005	0.005	0.01
		D08	Raw	4	0	0.035	0.017	0.02	0.025	0.030	0.045	0.06
			CFB	4	0	-0.000	0.016	-0.02	-0.010	0.000	0.010	0.02
		FUP	Raw	4	0	0.038	0.021	0.02	0.020	0.035	0.055	0.06
			CFB	4	0	0.003	0.010	-0.01	-0.005	0.005	0.010	0.01
	Eosinophils, % [%]	SC	Raw	4	0	1.30	0.56	0.6	0.85	1.40	1.75	1.8
		BL	Raw	4	0	1.63	1.40	0.7	0.80	1.05	2.45	3.7

n: Number of non-missing observations; %: Percentage based on non-missing observations; Miss: Missing observations; SD: Standard deviation; TP: Timepoint of measurement; SC: Screening; BL: Baseline; FUP: Follow-up; D: Day; Raw: Raw values; CFB: Change from baseline; Group 1: 1200 mg niclosamide solution; Group 2: 1600 mg niclosamide solution; Group 3: Placebo;

Output generated by program 'NIC002_T14_5_SafetyLaboratory_V02_0_0'

Table 14.5.2: Safety Laboratory
Haematology

Part C

Group	Parameter	Visit		n	Miss	Mean	SD	Minimum	Lower quartile	Median	Upper quartile	Maximum
	Eosinophils, % [%]	D03	Raw	4	0	1.73	1.06	1.0	1.10	1.30	2.35	3.3
			CFB	4	0	0.10	0.39	-0.4	-0.20	0.15	0.40	0.5
		D08	Raw	4	0	1.50	0.82	0.9	1.00	1.20	2.00	2.7
			CFB	4	0	-0.12	0.69	-1.0	-0.65	-0.05	0.40	0.6
		FUP	Raw	4	0	1.85	1.05	0.8	1.20	1.65	2.50	3.3
			CFB	4	0	0.23	0.61	-0.4	-0.25	0.15	0.70	1.0
	Eosinophils, abs. $[10^9/L]$	SC	Raw	4	0	0.080	0.036	0.04	0.050	0.085	0.110	0.11
		BL	Raw	4	0	0.115	0.104	0.05	0.055	0.070	0.175	0.27
		D03	Raw	4	0	0.100	0.062	0.06	0.060	0.075	0.140	0.19
			CFB	4	0	-0.015	0.044	-0.08	-0.040	0.005	0.010	0.01
		D08	Raw	4	0	0.078	0.043	0.05	0.050	0.060	0.105	0.14
			CFB	4	0	-0.038	0.062	-0.13	-0.070	-0.010	-0.005	0.00
		FUP	Raw	4	0	0.103	0.063	0.04	0.060	0.090	0.145	0.19
			CFB	4	0	-0.013	0.047	-0.08	-0.045	0.005	0.020	0.02
	Erythrocytes $[10^{12}/L]$	SC	Raw	4	0	4.220	0.239	3.99	4.015	4.215	4.425	4.46
		BL	Raw	4	0	4.185	0.298	3.75	3.995	4.290	4.375	4.41
		D03	Raw	4	0	4.288	0.259	3.96	4.080	4.340	4.495	4.51
			CFB	4	0	0.103	0.112	-0.04	0.015	0.120	0.190	0.21
		D08	Raw	4	0	4.173	0.237	3.94	3.985	4.140	4.360	4.47
			CFB	4	0	-0.013	0.265	-0.30	-0.230	-0.015	0.205	0.28
		FUP	Raw	4	0	4.125	0.237	3.91	3.920	4.120	4.330	4.35
			CFB	4	0	-0.060	0.209	-0.33	-0.195	-0.045	0.075	0.18
	Haematocrit [L/L]	SC	Raw	4	0	0.383	0.021	0.36	0.370	0.380	0.395	0.41

n: Number of non-missing observations; %: Percentage based on non-missing observations; Miss: Missing observations; SD: Standard deviation; TP: Timepoint of measurement; SC: Screening; BL: Baseline; FUP: Follow-up; D: Day; Raw: Raw values; CFB: Change from baseline; Group 1: 1200 mg niclosamide solution; Group 2: 1600 mg niclosamide solution; Group 3: Placebo;

Output generated by program 'NIC002_T14_5_SafetyLaboratory_V02_0_0'

Table 14.5.2: Safety Laboratory
Haematology

Part C

Group	Parameter	Visit		n	Miss	Mean	SD	Minimum	Lower quartile	Median	Upper quartile	Maximum
	Haematocrit [L/L]	BL	Raw	4	0	0.378	0.019	0.35	0.365	0.385	0.390	0.39
		D03	Raw	4	0	0.393	0.021	0.37	0.375	0.395	0.410	0.41
			CFB	4	0	0.015	0.010	0.00	0.010	0.020	0.020	0.02
		D08	Raw	4	0	0.380	0.016	0.36	0.370	0.380	0.390	0.40
			CFB	4	0	0.003	0.022	-0.02	-0.015	0.000	0.020	0.03
		FUP	Raw	4	0	0.375	0.017	0.36	0.360	0.375	0.390	0.39
			CFB	4	0	-0.003	0.013	-0.02	-0.010	0.000	0.005	0.01
	Haemoglobin [g/L]	SC	Raw	4	0	132.8	5.6	126	128.5	133.0	137.0	139
		BL	Raw	4	0	131.0	6.4	122	126.5	133.0	135.5	136
		D03	Raw	4	0	135.8	6.1	130	130.5	135.5	141.0	142
			CFB	4	0	4.8	4.2	-1	2.0	5.5	7.5	9
		D08	Raw	4	0	132.0	6.2	124	128.0	132.5	136.0	139
			CFB	4	0	1.0	7.3	-7	-4.5	0.5	6.5	10
		FUP	Raw	4	0	130.5	5.8	123	126.0	131.5	135.0	136
			CFB	4	0	-0.5	6.1	-8	-4.5	-0.5	3.5	7
	Leucocytes [10^9/L]	SC	Raw	4	0	6.040	0.434	5.45	5.720	6.140	6.360	6.43
		BL	Raw	4	0	6.958	1.119	5.47	6.170	7.115	7.745	8.13
		D03	Raw	4	0	5.960	1.269	4.41	5.060	5.980	6.860	7.47
			CFB	4	0	-0.998	1.120	-1.88	-1.765	-1.355	-0.230	0.60
		D08	Raw	4	0	5.345	1.527	3.95	4.315	4.970	6.375	7.49
			CFB	4	0	-1.613	2.040	-4.18	-3.140	-1.445	-0.085	0.62
		FUP	Raw	4	0	5.403	0.621	4.82	4.875	5.360	5.930	6.07
			CFB	4	0	-1.555	1.249	-3.31	-2.440	-1.185	-0.670	-0.54

n: Number of non-missing observations; %: Percentage based on non-missing observations; Miss: Missing observations; SD: Standard deviation; TP: Timepoint of measurement; SC: Screening; BL: Baseline; FUP: Follow-up; D: Day; Raw: Raw values; CFB: Change from baseline; Group 1: 1200 mg niclosamide solution; Group 2: 1600 mg niclosamide solution; Group 3: Placebo;

Output generated by program 'NIC002_T14_5_SafetyLaboratory_V02_0_0'

Table 14.5.2: Safety Laboratory
Haematology

Part C

Group	Parameter	Visit		n	Miss	Mean	SD	Minimum	Lower quartile	Median	Upper quartile	Maximum
	Lymphocytes, % [%]	SC	Raw	4	0	35.78	7.15	29.3	29.95	34.65	41.60	44.5
		BL	Raw	4	0	28.35	5.67	23.7	23.80	27.10	32.90	35.5
		D03	Raw	4	0	34.65	1.92	32.4	33.45	34.55	35.85	37.1
			CFB	4	0	6.30	6.54	-3.1	1.85	8.70	10.75	10.9
		D08	Raw	4	0	28.33	4.05	22.9	25.85	28.85	30.80	32.7
			CFB	4	0	-0.02	9.41	-12.6	-7.05	1.85	7.00	8.8
		FUP	Raw	4	0	36.60	5.52	29.2	32.75	37.45	40.45	42.3
			CFB	4	0	8.25	10.58	-6.3	1.00	10.35	15.50	18.6
	Lymphocytes, abs. [10^9/L]	SC	Raw	4	0	2.160	0.484	1.83	1.835	1.975	2.485	2.86
		BL	Raw	4	0	1.928	0.131	1.76	1.845	1.935	2.010	2.08
		D03	Raw	4	0	2.083	0.553	1.43	1.700	2.065	2.465	2.77
			CFB	4	0	0.155	0.496	-0.51	-0.150	0.220	0.460	0.69
		D08	Raw	4	0	1.523	0.515	1.07	1.105	1.430	1.940	2.16
			CFB	4	0	-0.405	0.494	-0.87	-0.830	-0.415	0.020	0.08
		FUP	Raw	4	0	1.980	0.383	1.44	1.740	2.070	2.220	2.34
			CFB	4	0	0.053	0.380	-0.50	-0.195	0.185	0.300	0.34
	Monocytes, % [%]	SC	Raw	4	0	7.90	1.85	5.6	6.65	7.95	9.15	10.1
		BL	Raw	4	0	6.93	1.31	5.0	6.15	7.40	7.70	7.9
		D03	Raw	4	0	6.95	1.41	5.4	6.00	6.80	7.90	8.8
			CFB	4	0	0.03	1.10	-1.3	-0.80	0.05	0.85	1.3
		D08	Raw	4	0	8.83	3.26	6.8	7.05	7.40	10.60	13.7
			CFB	4	0	1.90	4.54	-0.5	-0.45	-0.30	4.25	8.7
		FUP	Raw	4	0	7.48	0.54	6.8	7.10	7.50	7.85	8.1
			CFB	4	0	0.55	0.91	-0.3	-0.10	0.35	1.20	1.8

n: Number of non-missing observations; %: Percentage based on non-missing observations; Miss: Missing observations; SD: Standard deviation; TP: Timepoint of measurement; SC: Screening; BL: Baseline; FUP: Follow-up; D: Day; Raw: Raw values; CFB: Change from baseline; Group 1: 1200 mg niclosamide solution; Group 2: 1600 mg niclosamide solution; Group 3: Placebo;

Output generated by program 'NIC002_T14_5_SafetyLaboratory_V02_0_0'

Table 14.5.2: Safety Laboratory
Haematology

Part C

Group	Parameter	Visit		n	Miss	Mean	SD	Minimum	Lower quartile	Median	Upper quartile	Maximum
	Monocytes, abs. [10^9/L]	SC	Raw	4	0	0.473	0.090	0.35	0.405	0.495	0.540	0.55
		BL	Raw	4	0	0.475	0.075	0.41	0.410	0.475	0.540	0.54
		D03	Raw	4	0	0.405	0.062	0.34	0.365	0.395	0.445	0.49
			CFB	4	0	-0.070	0.051	-0.14	-0.105	-0.060	-0.035	-0.02
		D08	Raw	4	0	0.450	0.116	0.34	0.350	0.450	0.550	0.56
			CFB	4	0	-0.025	0.132	-0.18	-0.125	-0.025	0.075	0.13
		FUP	Raw	4	0	0.405	0.056	0.33	0.365	0.415	0.445	0.46
			CFB	4	0	-0.070	0.042	-0.11	-0.095	-0.080	-0.045	-0.01
	Neutrophils, % [%]	SC	Raw	4	0	54.40	8.86	44.7	47.10	54.45	61.70	64.0
		BL	Raw	4	0	62.58	6.20	55.7	57.85	62.20	67.30	70.2
		D03	Raw	4	0	56.13	2.07	54.4	54.45	55.70	57.80	58.7
			CFB	4	0	-6.45	5.68	-11.5	-10.70	-7.75	-2.20	1.2
		D08	Raw	4	0	60.70	5.80	55.3	56.25	59.60	65.15	68.3
			CFB	4	0	-1.88	11.87	-14.9	-11.05	-2.60	7.30	12.6
		FUP	Raw	4	0	53.43	5.56	48.8	50.10	51.70	56.75	61.5
			CFB	4	0	-9.15	11.32	-21.4	-16.90	-10.50	-1.40	5.8
	Neutrophils, abs. [10^9/L]	SC	Raw	4	0	3.290	0.618	2.70	2.785	3.215	3.795	4.03
		BL	Raw	4	0	4.405	1.111	3.05	3.590	4.435	5.220	5.70
		D03	Raw	4	0	3.340	0.679	2.51	2.810	3.390	3.870	4.07
			CFB	4	0	-1.065	0.919	-2.03	-1.830	-1.085	-0.300	-0.06
		D08	Raw	4	0	3.260	1.019	2.19	2.600	3.105	3.920	4.64
			CFB	4	0	-1.145	1.858	-3.51	-2.620	-0.790	0.330	0.51
		FUP	Raw	4	0	2.878	0.355	2.35	2.680	3.020	3.075	3.12

n: Number of non-missing observations; %: Percentage based on non-missing observations; Miss: Missing observations; SD: Standard deviation; TP: Timepoint of measurement; SC: Screening; BL: Baseline; FUP: Follow-up; D: Day; Raw: Raw values; CFB: Change from baseline; Group 1: 1200 mg niclosamide solution; Group 2: 1600 mg niclosamide solution; Group 3: Placebo;

Output generated by program 'NIC002_T14_5_SafetyLaboratory_V02_0_0'

Table 14.5.2: Safety Laboratory
Haematology

Part C

Group	Parameter	Visit		n	Miss	Mean	SD	Minimum	Lower quartile	Median	Upper quartile	Maximum
	Neutrophils, abs. [10^9/L]	FUP	CFB	4	0	-1.528	1.403	-3.35	-2.540	-1.370	-0.515	-0.02
	Platelets [10^9/L]	SC	Raw	4	0	267.3	36.7	216	242.0	276.0	292.5	301
		BL	Raw	4	0	254.5	34.8	209	227.5	262.0	281.5	285
		D03	Raw	4	0	260.3	38.3	209	231.0	270.0	289.5	292
			CFB	4	0	5.8	3.9	0	3.5	7.0	8.0	9
		D08	Raw	4	0	257.0	43.6	213	221.0	254.0	293.0	307
			CFB	4	0	2.5	25.5	-33	-16.0	10.5	21.0	22
		FUP	Raw	4	0	272.5	42.0	240	244.5	258.5	300.5	333
			CFB	4	0	18.0	30.2	-10	-8.0	17.0	44.0	48
Group 3	Basophils, % [%]	SC	Raw	4	0	0.65	0.34	0.2	0.40	0.70	0.90	1.0
		BL	Raw	4	0	0.95	0.53	0.5	0.60	0.80	1.30	1.7
		D03	Raw	4	0	0.75	0.38	0.5	0.50	0.60	1.00	1.3
			CFB	4	0	-0.20	0.16	-0.4	-0.30	-0.20	-0.10	0.0
		D08	Raw	3	1	0.53	0.25	0.3	0.30	0.50	0.80	0.8
			CFB	3	1	-0.17	0.06	-0.2	-0.20	-0.20	-0.10	-0.1
		FUP	Raw	4	0	0.83	0.38	0.4	0.55	0.80	1.10	1.3
			CFB	4	0	-0.13	0.19	-0.4	-0.25	-0.05	0.00	0.0
	Basophils, abs. [10^9/L]	SC	Raw	4	0	0.038	0.019	0.01	0.025	0.045	0.050	0.05
		BL	Raw	4	0	0.050	0.016	0.03	0.040	0.050	0.060	0.07
		D03	Raw	4	0	0.040	0.014	0.03	0.030	0.035	0.050	0.06
			CFB	4	0	-0.010	0.008	-0.02	-0.015	-0.010	-0.005	0.00
		D08	Raw	3	1	0.033	0.015	0.02	0.020	0.030	0.050	0.05
			CFB	3	1	-0.010	0.010	-0.02	-0.020	-0.010	0.000	0.00

n: Number of non-missing observations; %: Percentage based on non-missing observations; Miss: Missing observations; SD: Standard deviation; TP: Timepoint of measurement; SC: Screening; BL: Baseline; FUP: Follow-up; D: Day; Raw: Raw values; CFB: Change from baseline; Group 1: 1200 mg niclosamide solution; Group 2: 1600 mg niclosamide solution; Group 3: Placebo;

Output generated by program 'NIC002_T14_5_SafetyLaboratory_V02_0_0'

Table 14.5.2: Safety Laboratory
Haematology

Part C

Group	Parameter	Visit		n	Miss	Mean	SD	Minimum	Lower quartile	Median	Upper quartile	Maximum
	Basophils, abs. [10^9/L]	FUP	Raw	4	0	0.043	0.017	0.02	0.030	0.045	0.055	0.06
			CFB	4	0	-0.008	0.005	-0.01	-0.010	-0.010	-0.005	0.00
	Eosinophils, % [%]	SC	Raw	4	0	2.60	1.15	1.6	1.70	2.35	3.50	4.1
		BL	Raw	4	0	3.20	1.13	1.7	2.35	3.40	4.05	4.3
		D03	Raw	4	0	3.08	0.92	2.1	2.45	2.95	3.70	4.3
			CFB	4	0	-0.12	1.10	-1.2	-1.05	-0.20	0.80	1.1
		D08	Raw	3	1	1.83	0.47	1.3	1.30	2.00	2.20	2.2
			CFB	3	1	-1.17	1.47	-2.3	-2.30	-1.70	0.50	0.5
		FUP	Raw	4	0	2.90	1.25	1.2	2.00	3.15	3.80	4.1
			CFB	4	0	-0.30	1.27	-1.8	-1.30	-0.25	0.70	1.1
	Eosinophils, abs. [10^9/L]	SC	Raw	4	0	0.143	0.043	0.10	0.110	0.135	0.175	0.20
		BL	Raw	4	0	0.180	0.047	0.13	0.145	0.175	0.215	0.24
		D03	Raw	4	0	0.165	0.026	0.13	0.145	0.170	0.185	0.19
			CFB	4	0	-0.015	0.052	-0.06	-0.060	-0.015	0.030	0.03
		D08	Raw	3	1	0.113	0.029	0.08	0.080	0.130	0.130	0.13
			CFB	3	1	-0.073	0.064	-0.11	-0.110	-0.110	0.000	0.00
		FUP	Raw	4	0	0.148	0.061	0.06	0.105	0.170	0.190	0.19
			CFB	4	0	-0.033	0.074	-0.13	-0.090	-0.015	0.025	0.03
	Erythrocytes [10^{12}/L]	SC	Raw	4	0	3.753	0.043	3.70	3.720	3.755	3.785	3.80
		BL	Raw	4	0	3.780	0.209	3.50	3.620	3.840	3.940	3.94
		D03	Raw	4	0	3.820	0.110	3.73	3.750	3.785	3.890	3.98
			CFB	4	0	0.040	0.151	-0.14	-0.055	0.035	0.135	0.23
		D08	Raw	3	1	3.620	0.173	3.42	3.420	3.720	3.720	3.72

n: Number of non-missing observations; %: Percentage based on non-missing observations; Miss: Missing observations; SD: Standard deviation; TP: Timepoint of measurement; SC: Screening; BL: Baseline; FUP: Follow-up; D: Day; Raw: Raw values; CFB: Change from baseline; Group 1: 1200 mg niclosamide solution; Group 2: 1600 mg niclosamide solution; Group 3: Placebo;

Output generated by program 'NIC002_T14_5_SafetyLaboratory_V02_0_0'

Table 14.5.2: Safety Laboratory
Haematology

Part C

Group	Parameter	Visit		n	Miss	Mean	SD	Minimum	Lower quartile	Median	Upper quartile	Maximum
	Erythrocytes [10^{12}/L]	D08	CFB	3	1	-0.253	0.252	-0.52	-0.520	-0.220	-0.020	-0.02
		FUP	Raw	4	0	3.495	0.114	3.38	3.415	3.475	3.575	3.65
			CFB	4	0	-0.285	0.131	-0.44	-0.365	-0.290	-0.205	-0.12
	Haematocrit [L/L]	SC	Raw	4	0	0.343	0.010	0.33	0.335	0.345	0.350	0.35
		BL	Raw	4	0	0.350	0.022	0.32	0.335	0.355	0.365	0.37
		D03	Raw	4	0	0.355	0.013	0.34	0.345	0.355	0.365	0.37
			CFB	4	0	0.005	0.017	-0.02	-0.005	0.010	0.015	0.02
		D08	Raw	3	1	0.337	0.015	0.32	0.320	0.340	0.350	0.35
			CFB	3	1	-0.023	0.025	-0.05	-0.050	-0.020	0.000	0.00
		FUP	Raw	4	0	0.325	0.010	0.31	0.320	0.330	0.330	0.33
			CFB	4	0	-0.025	0.013	-0.04	-0.035	-0.025	-0.015	-0.01
	Haemoglobin [g/L]	SC	Raw	4	0	119.0	2.4	116	117.0	119.5	121.0	121
		BL	Raw	4	0	117.8	7.6	107	112.5	120.0	123.0	124
		D03	Raw	4	0	120.3	3.8	116	117.5	120.0	123.0	125
			CFB	4	0	2.5	5.7	-5	-1.0	3.0	6.0	9
		D08	Raw	3	1	115.3	5.5	109	109.0	118.0	119.0	119
			CFB	3	1	-6.0	7.9	-15	-15.0	-3.0	0.0	0
		FUP	Raw	4	0	110.3	4.3	105	107.0	110.5	113.5	115
			CFB	4	0	-7.5	4.2	-12	-10.5	-8.0	-4.5	-2
	Leucocytes [10^9/L]	SC	Raw	4	0	5.733	0.922	4.83	4.965	5.660	6.500	6.78
		BL	Raw	4	0	5.980	1.441	4.21	4.925	6.030	7.035	7.65
		D03	Raw	4	0	5.495	0.707	4.46	5.090	5.730	5.900	6.06
			CFB	4	0	-0.485	0.998	-1.93	-1.145	-0.130	0.175	0.25

n: Number of non-missing observations; %: Percentage based on non-missing observations; Miss: Missing observations; SD: Standard deviation; TP: Timepoint of measurement; SC: Screening; BL: Baseline; FUP: Follow-up; D: Day; Raw: Raw values; CFB: Change from baseline; Group 1: 1200 mg niclosamide solution; Group 2: 1600 mg niclosamide solution; Group 3: Placebo;

Output generated by program 'NIC002_T14_5_SafetyLaboratory_V02_0_0'

Table 14.5.2: Safety Laboratory
Haematology

Part C

Group	Parameter	Visit		n	Miss	Mean	SD	Minimum	Lower quartile	Median	Upper quartile	Maximum
	Leucocytes [10^9/L]	D08	Raw	3	1	6.280	0.291	5.96	5.960	6.350	6.530	6.53
			CFB	3	1	-0.290	1.304	-1.69	-1.690	-0.070	0.890	0.89
		FUP	Raw	4	0	5.118	0.396	4.67	4.785	5.160	5.450	5.48
			CFB	4	0	-0.862	1.231	-2.23	-1.875	-0.840	0.150	0.46
	Lymphocytes, % [%]	SC	Raw	4	0	32.75	4.52	27.6	29.05	32.95	36.45	37.5
		BL	Raw	4	0	34.13	10.87	24.5	25.50	32.00	42.75	48.0
		D03	Raw	4	0	35.38	8.85	24.9	29.60	35.05	41.15	46.5
			CFB	4	0	1.25	5.56	-3.2	-2.35	-0.55	4.85	9.3
		D08	Raw	3	1	29.60	6.26	22.7	22.70	31.20	34.90	34.9
			CFB	3	1	-3.40	9.01	-13.1	-13.10	-1.80	4.70	4.7
		FUP	Raw	4	0	38.03	10.29	26.7	30.15	37.35	45.90	50.7
			CFB	4	0	3.90	2.21	2.2	2.45	3.15	5.35	7.1
	Lymphocytes, abs. [10^9/L]	SC	Raw	4	0	1.880	0.403	1.41	1.560	1.890	2.200	2.33
		BL	Raw	4	0	1.973	0.536	1.57	1.575	1.805	2.370	2.71
		D03	Raw	4	0	1.940	0.547	1.51	1.520	1.790	2.360	2.67
			CFB	4	0	-0.033	0.036	-0.06	-0.055	-0.045	-0.010	0.02
		D08	Raw	3	1	1.860	0.420	1.44	1.440	1.860	2.280	2.28
			CFB	3	1	-0.243	0.163	-0.43	-0.430	-0.170	-0.130	-0.13
		FUP	Raw	4	0	1.958	0.610	1.31	1.565	1.870	2.350	2.78
			CFB	4	0	-0.015	0.278	-0.26	-0.235	-0.070	0.205	0.34
	Monocytes, % [%]	SC	Raw	4	0	9.23	3.00	6.9	7.15	8.25	11.30	13.5
		BL	Raw	4	0	8.63	1.24	7.3	7.70	8.50	9.55	10.2
		D03	Raw	4	0	8.40	1.61	7.4	7.55	7.70	9.25	10.8

n: Number of non-missing observations; %: Percentage based on non-missing observations; Miss: Missing observations; SD: Standard deviation; TP: Timepoint of measurement; SC: Screening; BL: Baseline; FUP: Follow-up; D: Day; Raw: Raw values; CFB: Change from baseline; Group 1: 1200 mg niclosamide solution; Group 2: 1600 mg niclosamide solution; Group 3: Placebo;

Output generated by program 'NIC002_T14_5_SafetyLaboratory_V02_0_0'

Table 14.5.2: Safety Laboratory
Haematology

Part C

Group	Parameter	Visit		n	Miss	Mean	SD	Minimum	Lower quartile	Median	Upper quartile	Maximum
	Monocytes, % [%]	D03	CFB	4	0	-0.22	0.87	-1.2	-0.95	-0.15	0.50	0.6
		D08	Raw	3	1	7.60	1.73	6.5	6.50	6.70	9.60	9.6
			CFB	3	1	-0.50	2.44	-2.2	-2.20	-1.60	2.30	2.3
		FUP	Raw	4	0	10.78	3.64	8.0	8.10	9.65	13.45	15.8
			CFB	4	0	2.15	3.04	-0.7	-0.40	1.85	4.70	5.6
	Monocytes, abs. [10^9/L]	SC	Raw	4	0	0.520	0.141	0.35	0.405	0.540	0.635	0.65
		BL	Raw	4	0	0.503	0.054	0.43	0.465	0.510	0.540	0.56
		D03	Raw	4	0	0.453	0.019	0.44	0.440	0.445	0.465	0.48
			CFB	4	0	-0.050	0.072	-0.12	-0.095	-0.065	-0.005	0.05
		D08	Raw	3	1	0.473	0.085	0.41	0.410	0.440	0.570	0.57
			CFB	3	1	-0.053	0.060	-0.11	-0.110	-0.060	0.010	0.01
		FUP	Raw	4	0	0.545	0.157	0.39	0.420	0.525	0.670	0.74
			CFB	4	0	0.042	0.191	-0.13	-0.090	-0.005	0.175	0.31
	Neutrophils, % [%]	SC	Raw	4	0	54.78	7.07	46.0	49.35	55.35	60.20	62.4
		BL	Raw	4	0	53.10	12.93	37.9	42.35	55.30	63.85	63.9
		D03	Raw	4	0	52.40	9.66	42.0	45.65	51.25	59.15	65.1
			CFB	4	0	-0.70	6.71	-10.6	-4.70	1.85	3.30	4.1
		D08	Raw	3	1	60.43	7.61	55.6	55.60	56.50	69.20	69.2
			CFB	3	1	5.23	12.50	-7.3	-7.30	5.30	17.70	17.7
		FUP	Raw	4	0	47.48	12.83	36.7	37.20	44.75	57.75	63.7
			CFB	4	0	-5.62	5.82	-12.0	-10.55	-5.15	-0.70	-0.2
	Neutrophils, abs. [10^9/L]	SC	Raw	4	0	3.153	0.705	2.22	2.700	3.230	3.605	3.93
		BL	Raw	4	0	3.275	1.445	1.97	2.055	3.125	4.495	4.88

n: Number of non-missing observations; %: Percentage based on non-missing observations; Miss: Missing observations; SD: Standard deviation; TP: Timepoint of measurement; SC: Screening; BL: Baseline; FUP: Follow-up; D: Day; Raw: Raw values; CFB: Change from baseline; Group 1: 1200 mg niclosamide solution; Group 2: 1600 mg niclosamide solution; Group 3: Placebo;

Output generated by program 'NIC002_T14_5_SafetyLaboratory_V02_0_0'

Table 14.5.2: Safety Laboratory
Haematology

Part C

Group	Parameter	Visit		n	Miss	Mean	SD	Minimum	Lower quartile	Median	Upper quartile	Maximum
	Neutrophils, abs. [10^9/L]	D03	Raw	4	0	2.898	0.781	2.20	2.305	2.725	3.490	3.94
			CFB	4	0	-0.378	0.995	-1.84	-1.005	0.030	0.250	0.27
		D08	Raw	3	1	3.800	0.536	3.37	3.370	3.630	4.400	4.40
			CFB	3	1	0.090	1.510	-1.51	-1.510	0.290	1.490	1.49
		FUP	Raw	4	0	2.425	0.644	1.76	1.885	2.410	2.965	3.12
			CFB	4	0	-0.850	0.901	-2.07	-1.530	-0.600	-0.170	-0.13
	Platelets [10^9/L]	SC	Raw	4	0	268.3	74.3	197	205.0	265.0	331.5	346
		BL	Raw	4	0	276.0	95.8	182	198.5	265.0	353.5	392
		D03	Raw	4	0	273.8	93.3	194	203.0	251.0	344.5	399
			CFB	4	0	-2.3	16.4	-25	-14.0	2.0	9.5	12
		D08	Raw	3	1	244.3	72.0	195	195.0	211.0	327.0	327
			CFB	3	1	7.0	9.5	-4	-4.0	12.0	13.0	13
		FUP	Raw	4	0	283.0	89.7	201	206.5	276.5	359.5	378
			CFB	4	0	7.0	18.7	-14	-8.5	8.0	22.5	26

n: Number of non-missing observations; %: Percentage based on non-missing observations; Miss: Missing observations; SD: Standard deviation; TP: Timepoint of measurement; SC: Screening; BL: Baseline; FUP: Follow-up; D: Day; Raw: Raw values; CFB: Change from baseline; Group 1: 1200 mg niclosamide solution; Group 2: 1600 mg niclosamide solution; Group 3: Placebo;

Output generated by program 'NIC002_T14_5_SafetyLaboratory_V02_0_0'

Table 14.6: Vital signs

Part A

Cohort	Parameter	Visit	TP		n	Miss	Mean	SD	Minimum	Lower quartile	Median	Upper quartile	Maximum
Cohort A1	Systolic blood pressure [mmHg]	SC		Raw	3	0	101.0	9.5	90	90.0	106.0	107.0	107
		BL		Raw	3	0	103.0	14.0	87	87.0	109.0	113.0	113
		D01	Pre-dose	Raw	3	0	101.3	11.0	90	90.0	102.0	112.0	112
				CFB	3	0	-1.7	5.0	-7	-7.0	-1.0	3.0	3
			1 h	Raw	3	0	107.0	13.9	91	91.0	114.0	116.0	116
				CFB	3	0	4.0	1.0	3	3.0	4.0	5.0	5
			2 h	Raw	3	0	101.7	15.3	90	90.0	96.0	119.0	119
				CFB	3	0	-1.3	10.2	-13	-13.0	3.0	6.0	6
			4 h	Raw	3	0	104.0	7.8	95	95.0	108.0	109.0	109
				CFB	3	0	1.0	6.6	-5	-5.0	0.0	8.0	8
			8 h	Raw	3	0	99.0	7.2	91	91.0	101.0	105.0	105
				CFB	3	0	-4.0	6.9	-8	-8.0	-8.0	4.0	4
			12 h	Raw	3	0	101.3	8.5	93	93.0	101.0	110.0	110
				CFB	3	0	-1.7	9.3	-12	-12.0	1.0	6.0	6
		FUP		Raw	3	0	104.0	7.0	99	99.0	101.0	112.0	112
				CFB	3	0	1.0	10.1	-8	-8.0	-1.0	12.0	12
	Diastolic blood pressure [mmHg]	SC		Raw	3	0	59.3	4.7	54	54.0	61.0	63.0	63
		BL		Raw	3	0	60.0	10.5	49	49.0	61.0	70.0	70
		D01	Pre-dose	Raw	3	0	60.7	9.3	50	50.0	65.0	67.0	67

n: Number of non-missing observations; %: Percentage based on non-missing observations; Miss: Missing observations; SD: Standard deviation; TP: Timepoint of measurement; SC: Screening; BL: Baseline; FUP: Follow-up; D: Day; Raw: Raw values; CFB: Change from baseline; Cohort A1: 200mg oral dose niclosamide; Cohort A2: 600mg oral dose niclosamide; Cohort A3: 1600mg oral dose niclosamide; Fast/Fed: Treatment in cohort A3 was applied under fasting and fed conditions in the same subjects;

Output generated by program 'NIC002_T14_6_VitalSigns_V02_0_0'

Table 14.6: Vital signs

Part A

Cohort	Parameter	Visit	TP		n	Miss	Mean	SD	Minimum	Lower quartile	Median	Upper quartile	Maximum
	Diastolic blood pressure [mmHg]	D01	Pre-dose	CFB	3	0	0.7	3.5	-3	-3.0	1.0	4.0	4
			1 h	Raw	3	0	65.7	9.2	55	55.0	71.0	71.0	71
				CFB	3	0	5.7	4.5	1	1.0	6.0	10.0	10
			2 h	Raw	3	0	60.3	10.5	50	50.0	60.0	71.0	71
				CFB	3	0	0.3	1.2	-1	-1.0	1.0	1.0	1
			4 h	Raw	3	0	64.0	10.5	54	54.0	63.0	75.0	75
				CFB	3	0	4.0	1.7	2	2.0	5.0	5.0	5
			8 h	Raw	3	0	57.7	6.1	51	51.0	59.0	63.0	63
				CFB	3	0	-2.3	7.5	-11	-11.0	2.0	2.0	2
			12 h	Raw	3	0	58.3	7.6	50	50.0	60.0	65.0	65
				CFB	3	0	-1.7	7.4	-10	-10.0	1.0	4.0	4
		FUP		Raw	3	0	56.7	5.5	51	51.0	57.0	62.0	62
				CFB	3	0	-3.3	5.0	-8	-8.0	-4.0	2.0	2
	Heart rate [beats/min]	SC		Raw	3	0	75.0	8.2	68	68.0	73.0	84.0	84
		BL		Raw	3	0	77.3	6.5	71	71.0	77.0	84.0	84
		D01	Pre-dose	Raw	3	0	74.3	10.1	68	68.0	69.0	86.0	86
				CFB	3	0	-3.0	5.6	-9	-9.0	-2.0	2.0	2
			1 h	Raw	3	0	74.7	9.2	64	64.0	80.0	80.0	80
				CFB	3	0	-2.7	5.1	-7	-7.0	-4.0	3.0	3
			2 h	Raw	3	0	68.0	3.0	65	65.0	68.0	71.0	71
				CFB	3	0	-9.3	3.5	-13	-13.0	-9.0	-6.0	-6
			4 h	Raw	3	0	69.3	7.5	62	62.0	69.0	77.0	77
				CFB	3	0	-8.0	6.6	-15	-15.0	-7.0	-2.0	-2
			8 h	Raw	3	0	65.0	6.1	58	58.0	68.0	69.0	69
				CFB	3	0	-12.3	12.1	-26	-26.0	-8.0	-3.0	-3

n: Number of non-missing observations; %: Percentage based on non-missing observations; Miss: Missing observations; SD: Standard deviation; TP: Timepoint of measurement; SC: Screening; BL: Baseline; FUP: Follow-up; D: Day; Raw: Raw values; CFB: Change from baseline; Cohort A1: 200mg oral dose niclosamide; Cohort A2: 600mg oral dose niclosamide; Cohort A3: 1600mg oral dose niclosamide; Fast/Fed: Treatment in cohort A3 was applied under fasting and fed conditions in the same subjects;

Output generated by program 'NIC002_T14_6_VitalSigns_V02_0_0'

Table 14.6: Vital signs

Part A

Cohort	Parameter	Visit	TP		n	Miss	Mean	SD	Minimum	Lower quartile	Median	Upper quartile	Maximum
	Heart rate [beats/min]		12 h	Raw	3	0	75.7	6.0	70	70.0	75.0	82.0	82
				CFB	3	0	-1.7	5.5	-7	-7.0	-2.0	4.0	4
		FUP		Raw	3	0	81.3	11.4	72	72.0	78.0	94.0	94
				CFB	3	0	4.0	7.9	-5	-5.0	7.0	10.0	10
	Body temperature [°C]	SC		Raw	3	0	36.80	0.26	36.6	36.60	36.70	37.10	37.1
		BL		Raw	3	0	36.80	0.26	36.6	36.60	36.70	37.10	37.1
		D01	Pre-dose	Raw	3	0	36.90	0.26	36.7	36.70	36.80	37.20	37.2
				CFB	3	0	0.10	0.46	-0.4	-0.40	0.20	0.50	0.5
			3 h	Raw	3	0	36.83	0.15	36.7	36.70	36.80	37.00	37.0
				CFB	3	0	0.03	0.38	-0.4	-0.40	0.20	0.30	0.3
		FUP		Raw	3	0	35.80	0.62	35.3	35.30	35.60	36.50	36.5
				CFB	3	0	-1.00	0.36	-1.3	-1.30	-1.10	-0.60	-0.6
	Respiration [breaths/min]	SC		Raw	3	0	14.0	2.0	12	12.0	14.0	16.0	16
		BL		Raw	3	0	14.3	2.3	13	13.0	13.0	17.0	17
		D01	Pre-dose	Raw	3	0	13.3	1.2	12	12.0	14.0	14.0	14
				CFB	3	0	-1.0	2.0	-3	-3.0	-1.0	1.0	1
			1 h	Raw	3	0	13.0	0.0	13	13.0	13.0	13.0	13
				CFB	3	0	-1.3	2.3	-4	-4.0	0.0	0.0	0
			2 h	Raw	3	0	13.7	0.6	13	13.0	14.0	14.0	14
				CFB	3	0	-0.7	2.1	-3	-3.0	0.0	1.0	1
			4 h	Raw	3	0	13.0	1.0	12	12.0	13.0	14.0	14
				CFB	3	0	-1.3	2.5	-4	-4.0	-1.0	1.0	1

n: Number of non-missing observations; %: Percentage based on non-missing observations; Miss: Missing observations; SD: Standard deviation; TP: Timepoint of measurement; SC: Screening; BL: Baseline; FUP: Follow-up; D: Day; Raw: Raw values; CFB: Change from baseline; Cohort A1: 200mg oral dose niclosamide; Cohort A2: 600mg oral dose niclosamide; Cohort A3: 1600mg oral dose niclosamide; Fast/Fed: Treatment in cohort A3 was applied under fasting and fed conditions in the same subjects;

Output generated by program 'NIC002_T14_6_VitalSigns_V02_0_0'

Table 14.6: Vital signs

Part A

Cohort	Parameter	Visit	TP		n	Miss	Mean	SD	Minimum	Lower quartile	Median	Upper quartile	Maximum
	Respiration [breaths/min]	D01	8 h	Raw	3	0	13.7	0.6	13	13.0	14.0	14.0	14
				CFB	3	0	-0.7	2.1	-3	-3.0	0.0	1.0	1
			12 h	Raw	3	0	14.7	2.9	13	13.0	13.0	18.0	18
				CFB	3	0	0.3	4.5	-4	-4.0	0.0	5.0	5
		FUP		Raw	3	0	12.7	1.2	12	12.0	12.0	14.0	14
				CFB	3	0	-1.7	3.1	-5	-5.0	-1.0	1.0	1
Cohort A2	Systolic blood pressure [mmHg]	SC		Raw	3	0	112.0	16.4	98	98.0	108.0	130.0	130
		BL		Raw	3	0	108.0	9.0	99	99.0	108.0	117.0	117
		D01	Pre-dose	Raw	3	0	107.0	6.2	100	100.0	109.0	112.0	112
				CFB	3	0	-1.0	3.5	-5	-5.0	1.0	1.0	1
			1 h	Raw	3	0	104.7	4.7	101	101.0	103.0	110.0	110
				CFB	3	0	-3.3	4.7	-7	-7.0	-5.0	2.0	2
			2 h	Raw	3	0	106.3	4.6	101	101.0	109.0	109.0	109
				CFB	3	0	-1.7	5.5	-8	-8.0	1.0	2.0	2
			4 h	Raw	3	0	105.7	4.2	101	101.0	107.0	109.0	109
				CFB	3	0	-2.3	5.1	-8	-8.0	-1.0	2.0	2
			8 h	Raw	3	0	103.0	5.3	97	97.0	105.0	107.0	107
				CFB	3	0	-5.0	4.4	-10	-10.0	-3.0	-2.0	-2
			12 h	Raw	3	0	101.7	7.8	93	93.0	104.0	108.0	108
				CFB	3	0	-6.3	2.5	-9	-9.0	-6.0	-4.0	-4
		FUP		Raw	3	0	107.7	5.9	101	101.0	110.0	112.0	112
				CFB	3	0	-0.3	4.0	-5	-5.0	2.0	2.0	2

n: Number of non-missing observations; %: Percentage based on non-missing observations; Miss: Missing observations; SD: Standard deviation; TP: Timepoint of measurement; SC: Screening; BL: Baseline; FUP: Follow-up; D: Day; Raw: Raw values; CFB: Change from baseline; Cohort A1: 200mg oral dose niclosamide; Cohort A2: 600mg oral dose niclosamide; Cohort A3: 1600mg oral dose niclosamide; Fast/Fed: Treatment in cohort A3 was applied under fasting and fed conditions in the same subjects;

Output generated by program 'NIC002_T14_6_VitalSigns_V02_0_0'

Table 14.6: Vital signs

Part A

Cohort	Parameter	Visit	TP		n	Miss	Mean	SD	Minimum	Lower quartile	Median	Upper quartile	Maximum
	Diastolic blood pressure [mmHg]	SC		Raw	3	0	69.3	10.5	59	59.0	69.0	80.0	80
		BL		Raw	3	0	64.3	7.2	56	56.0	68.0	69.0	69
		D01	Pre-dose	Raw	3	0	62.0	6.0	56	56.0	62.0	68.0	68
				CFB	3	0	-2.3	4.0	-7	-7.0	0.0	0.0	0
			1 h	Raw	3	0	65.0	2.0	63	63.0	65.0	67.0	67
				CFB	3	0	0.7	7.6	-6	-6.0	-1.0	9.0	9
			2 h	Raw	3	0	60.0	3.6	56	56.0	61.0	63.0	63
				CFB	3	0	-4.3	8.6	-12	-12.0	-6.0	5.0	5
			4 h	Raw	3	0	60.0	1.7	58	58.0	61.0	61.0	61
				CFB	3	0	-4.3	8.3	-11	-11.0	-7.0	5.0	5
			8 h	Raw	3	0	61.3	5.9	57	57.0	59.0	68.0	68
				CFB	3	0	-3.0	6.1	-10	-10.0	0.0	1.0	1
			12 h	Raw	3	0	55.3	3.5	52	52.0	55.0	59.0	59
				CFB	3	0	-9.0	5.0	-14	-14.0	-9.0	-4.0	-4
		FUP		Raw	3	0	64.3	5.7	58	58.0	66.0	69.0	69
				CFB	3	0	0.0	2.0	-2	-2.0	0.0	2.0	2
	Heart rate [beats/min]	SC		Raw	3	0	64.3	6.8	59	59.0	62.0	72.0	72
		BL		Raw	3	0	69.7	5.7	65	65.0	68.0	76.0	76
		D01	Pre-dose	Raw	3	0	60.3	7.6	52	52.0	62.0	67.0	67
				CFB	3	0	-9.3	12.7	-24	-24.0	-3.0	-1.0	-1
			1 h	Raw	3	0	61.0	5.0	56	56.0	61.0	66.0	66
				CFB	3	0	-8.7	10.6	-20	-20.0	-7.0	1.0	1

n: Number of non-missing observations; %: Percentage based on non-missing observations; Miss: Missing observations; SD: Standard deviation; TP: Timepoint of measurement; SC: Screening; BL: Baseline; FUP: Follow-up; D: Day; Raw: Raw values; CFB: Change from baseline; Cohort A1: 200mg oral dose niclosamide; Cohort A2: 600mg oral dose niclosamide; Cohort A3: 1600mg oral dose niclosamide; Fast/Fed: Treatment in cohort A3 was applied under fasting and fed conditions in the same subjects;

Output generated by program 'NIC002_T14_6_VitalSigns_V02_0_0'

Table 14.6: Vital signs

Part A

Cohort	Parameter	Visit	TP		n	Miss	Mean	SD	Minimum	Lower quartile	Median	Upper quartile	Maximum
	Heart rate [beats/min]	D01	2 h	Raw	3	0	64.3	6.0	58	58.0	65.0	70.0	70
				CFB	3	0	-5.3	11.7	-18	-18.0	-3.0	5.0	5
			4 h	Raw	3	0	63.7	5.5	58	58.0	64.0	69.0	69
				CFB	3	0	-6.0	10.4	-18	-18.0	-1.0	1.0	1
			8 h	Raw	3	0	66.7	3.2	63	63.0	68.0	69.0	69
				CFB	3	0	-3.0	8.7	-13	-13.0	1.0	3.0	3
			12 h	Raw	3	0	68.0	2.0	66	66.0	68.0	70.0	70
				CFB	3	0	-1.7	7.6	-10	-10.0	0.0	5.0	5
		FUP		Raw	3	0	74.0	5.6	69	69.0	73.0	80.0	80
				CFB	3	0	4.3	0.6	4	4.0	4.0	5.0	5
	Body temperature [°C]	SC		Raw	3	0	36.80	0.36	36.4	36.40	36.90	37.10	37.1
		BL		Raw	3	0	36.97	0.21	36.8	36.80	36.90	37.20	37.2
		D01	Pre-dose	Raw	3	0	37.03	0.06	37.0	37.00	37.00	37.10	37.1
				CFB	3	0	0.07	0.15	-0.1	-0.10	0.10	0.20	0.2
			3 h	Raw	3	0	37.13	0.25	36.9	36.90	37.10	37.40	37.4
				CFB	3	0	0.17	0.38	-0.1	-0.10	0.00	0.60	0.6
		FUP		Raw	3	0	36.90	0.10	36.8	36.80	36.90	37.00	37.0
				CFB	3	0	-0.07	0.31	-0.4	-0.40	0.00	0.20	0.2
	Respiration [breaths/min]	SC		Raw	3	0	13.3	2.3	12	12.0	12.0	16.0	16
		BL		Raw	3	0	13.0	1.7	12	12.0	12.0	15.0	15
		D01	Pre-dose	Raw	3	0	13.0	2.6	10	10.0	14.0	15.0	15
				CFB	3	0	0.0	4.4	-5	-5.0	2.0	3.0	3

n: Number of non-missing observations; %: Percentage based on non-missing observations; Miss: Missing observations; SD: Standard deviation; TP: Timepoint of measurement; SC: Screening; BL: Baseline; FUP: Follow-up; D: Day; Raw: Raw values; CFB: Change from baseline; Cohort A1: 200mg oral dose niclosamide; Cohort A2: 600mg oral dose niclosamide; Cohort A3: 1600mg oral dose niclosamide; Fast/Fed: Treatment in cohort A3 was applied under fasting and fed conditions in the same subjects;

Output generated by program 'NIC002_T14_6_VitalSigns_V02_0_0'

Table 14.6: Vital signs

Part A

Cohort	Parameter	Visit	TP		n	Miss	Mean	SD	Minimum	Lower quartile	Median	Upper quartile	Maximum
	Respiration [breaths/min]	D01	1 h	Raw	3	0	13.0	2.0	11	11.0	13.0	15.0	15
				CFB	3	0	0.0	3.6	-4	-4.0	1.0	3.0	3
			2 h	Raw	3	0	13.0	2.0	11	11.0	13.0	15.0	15
				CFB	3	0	0.0	3.6	-4	-4.0	1.0	3.0	3
			4 h	Raw	3	0	11.7	1.5	10	10.0	12.0	13.0	13
				CFB	3	0	-1.3	1.2	-2	-2.0	-2.0	0.0	0
			8 h	Raw	3	0	11.7	1.2	11	11.0	11.0	13.0	13
				CFB	3	0	-1.3	0.6	-2	-2.0	-1.0	-1.0	-1
			12 h	Raw	3	0	14.0	1.0	13	13.0	14.0	15.0	15
				CFB	3	0	1.0	1.0	0	0.0	1.0	2.0	2
		FUP		Raw	3	0	13.0	1.0	12	12.0	13.0	14.0	14
				CFB	3	0	0.0	2.6	-3	-3.0	1.0	2.0	2
Cohort A3 Fast	Systolic blood pressure [mmHg]	SC		Raw	3	0	110.7	6.7	103	103.0	114.0	115.0	115
		BL		Raw	3	0	108.7	6.7	101	101.0	112.0	113.0	113
		D01	Pre-dose	Raw	3	0	113.0	10.4	107	107.0	107.0	125.0	125
				CFB	3	0	4.3	17.0	-6	-6.0	-5.0	24.0	24
			1 h	Raw	3	0	106.3	5.1	102	102.0	105.0	112.0	112
				CFB	3	0	-2.3	11.6	-10	-10.0	-8.0	11.0	11
			2 h	Raw	3	0	104.7	1.5	103	103.0	105.0	106.0	106
				CFB	3	0	-4.0	7.9	-10	-10.0	-7.0	5.0	5
			4 h	Raw	3	0	113.0	14.7	100	100.0	110.0	129.0	129
				CFB	3	0	4.3	11.0	-3	-3.0	-1.0	17.0	17
			8 h	Raw	3	0	100.7	4.6	98	98.0	98.0	106.0	106
				CFB	3	0	-8.0	5.6	-14	-14.0	-7.0	-3.0	-3

n: Number of non-missing observations; %: Percentage based on non-missing observations; Miss: Missing observations; SD: Standard deviation; TP: Timepoint of measurement; SC: Screening; BL: Baseline; FUP: Follow-up; D: Day; Raw: Raw values; CFB: Change from baseline; Cohort A1: 200mg oral dose niclosamide; Cohort A2: 600mg oral dose niclosamide; Cohort A3: 1600mg oral dose niclosamide; Fast/Fed: Treatment in cohort A3 was applied under fasting and fed conditions in the same subjects;

Output generated by program 'NIC002_T14_6_VitalSigns_V02_0_0'

Table 14.6: Vital signs

Part A

Cohort	Parameter	Visit	TP		n	Miss	Mean	SD	Minimum	Lower quartile	Median	Upper quartile	Maximum
Cohort A3 Fast	Systolic blood pressure [mmHg]	D01	12 h	Raw	3	0	117.0	16.6	105	105.0	110.0	136.0	136
				CFB	3	0	8.3	14.0	-3	-3.0	4.0	24.0	24
		FUP		Raw	3	0	112.3	8.4	107	107.0	108.0	122.0	122
				CFB	3	0	3.7	7.8	-5	-5.0	6.0	10.0	10
	Diastolic blood pressure [mmHg]	SC		Raw	3	0	60.0	5.6	55	55.0	59.0	66.0	66
		BL		Raw	3	0	59.3	3.2	57	57.0	58.0	63.0	63
		D01	Pre-dose	Raw	3	0	62.3	7.8	56	56.0	60.0	71.0	71
				CFB	3	0	3.0	9.5	-3	-3.0	-2.0	14.0	14
			1 h	Raw	3	0	62.7	1.5	61	61.0	63.0	64.0	64
				CFB	3	0	3.3	4.7	-2	-2.0	5.0	7.0	7
			2 h	Raw	3	0	57.3	0.6	57	57.0	57.0	58.0	58
				CFB	3	0	-2.0	3.5	-6	-6.0	0.0	0.0	0
			4 h	Raw	3	0	62.3	6.7	58	58.0	59.0	70.0	70
				CFB	3	0	3.0	3.6	0	0.0	2.0	7.0	7
			8 h	Raw	3	0	55.3	1.5	54	54.0	55.0	57.0	57
				CFB	3	0	-4.0	1.7	-6	-6.0	-3.0	-3.0	-3
			12 h	Raw	3	0	63.7	10.7	57	57.0	58.0	76.0	76
				CFB	3	0	4.3	7.6	-1	-1.0	1.0	13.0	13
		FUP		Raw	3	0	65.3	12.7	58	58.0	58.0	80.0	80
				CFB	3	0	6.0	9.5	0	0.0	1.0	17.0	17
	Heart rate [beats/min]	SC		Raw	3	0	64.7	6.4	60	60.0	62.0	72.0	72

n: Number of non-missing observations; %: Percentage based on non-missing observations; Miss: Missing observations; SD: Standard deviation; TP: Timepoint of measurement; SC: Screening; BL: Baseline; FUP: Follow-up; D: Day; Raw: Raw values; CFB: Change from baseline; Cohort A1: 200mg oral dose niclosamide; Cohort A2: 600mg oral dose niclosamide; Cohort A3: 1600mg oral dose niclosamide; Fast/Fed: Treatment in cohort A3 was applied under fasting and fed conditions in the same subjects;

Output generated by program 'NIC002_T14_6_VitalSigns_V02_0_0'

Table 14.6: Vital signs

Part A

Cohort	Parameter	Visit	TP		n	Miss	Mean	SD	Minimum	Lower quartile	Median	Upper quartile	Maximum
	Heart rate [beats/min]	BL		Raw	3	0	64.3	10.2	57	57.0	60.0	76.0	76
		D01	Pre-dose	Raw	3	0	58.3	6.7	51	51.0	60.0	64.0	64
				CFB	3	0	-6.0	10.0	-16	-16.0	-6.0	4.0	4
			1 h	Raw	3	0	67.3	13.9	52	52.0	71.0	79.0	79
				CFB	3	0	3.0	8.0	-5	-5.0	3.0	11.0	11
			2 h	Raw	3	0	65.7	11.4	53	53.0	69.0	75.0	75
				CFB	3	0	1.3	6.8	-4	-4.0	-1.0	9.0	9
			4 h	Raw	3	0	65.0	11.1	53	53.0	67.0	75.0	75
				CFB	3	0	0.7	5.7	-4	-4.0	-1.0	7.0	7
			8 h	Raw	3	0	65.3	4.2	62	62.0	64.0	70.0	70
				CFB	3	0	1.0	6.1	-6	-6.0	4.0	5.0	5
			12 h	Raw	3	0	67.3	4.6	62	62.0	70.0	70.0	70
				CFB	3	0	3.0	8.2	-6	-6.0	5.0	10.0	10
		FUP		Raw	3	0	69.0	13.9	53	53.0	77.0	77.0	77
				CFB	3	0	4.7	13.9	-7	-7.0	1.0	20.0	20
	Body temperature [°C]	SC		Raw	3	0	36.77	0.31	36.5	36.50	36.70	37.10	37.1
		BL		Raw	3	0	36.67	0.23	36.4	36.40	36.80	36.80	36.8
		D01	Pre-dose	Raw	3	0	36.83	0.29	36.5	36.50	37.00	37.00	37.0
				CFB	3	0	0.17	0.45	-0.3	-0.30	0.20	0.60	0.6
			3 h	Raw	3	0	37.00	0.66	36.3	36.30	37.10	37.60	37.6
				CFB	3	0	0.33	0.45	-0.1	-0.10	0.30	0.80	0.8
		FUP		Raw	3	0	36.63	0.06	36.6	36.60	36.60	36.70	36.7
				CFB	3	0	-0.03	0.21	-0.2	-0.20	-0.10	0.20	0.2

n: Number of non-missing observations; %: Percentage based on non-missing observations; Miss: Missing observations; SD: Standard deviation; TP: Timepoint of measurement; SC: Screening; BL: Baseline; FUP: Follow-up; D: Day; Raw: Raw values; CFB: Change from baseline; Cohort A1: 200mg oral dose niclosamide; Cohort A2: 600mg oral dose niclosamide; Cohort A3: 1600mg oral dose niclosamide; Fast/Fed: Treatment in cohort A3 was applied under fasting and fed conditions in the same subjects;

Output generated by program 'NIC002_T14_6_VitalSigns_V02_0_0'

Table 14.6: Vital signs

Part A

Cohort	Parameter	Visit	TP		n	Miss	Mean	SD	Minimum	Lower quartile	Median	Upper quartile	Maximum
	Respiration [breaths/min]	SC		Raw	3	0	13.0	1.7	12	12.0	12.0	15.0	15
		BL		Raw	3	0	15.7	0.6	15	15.0	16.0	16.0	16
		D01	Pre-dose	Raw	3	0	16.3	1.2	15	15.0	17.0	17.0	17
				CFB	3	0	0.7	1.5	-1	-1.0	1.0	2.0	2
			1 h	Raw	3	0	16.0	0.0	16	16.0	16.0	16.0	16
				CFB	3	0	0.3	0.6	0	0.0	0.0	1.0	1
			2 h	Raw	3	0	16.0	0.0	16	16.0	16.0	16.0	16
				CFB	3	0	0.3	0.6	0	0.0	0.0	1.0	1
			4 h	Raw	3	0	17.0	2.6	15	15.0	16.0	20.0	20
				CFB	3	0	1.3	3.2	-1	-1.0	0.0	5.0	5
			8 h	Raw	3	0	15.3	0.6	15	15.0	15.0	16.0	16
				CFB	3	0	-0.3	0.6	-1	-1.0	0.0	0.0	0
			12 h	Raw	3	0	14.7	0.6	14	14.0	15.0	15.0	15
				CFB	3	0	-1.0	0.0	-1	-1.0	-1.0	-1.0	-1
		FUP		Raw	3	0	15.0	4.4	12	12.0	13.0	20.0	20
				CFB	3	0	-0.7	4.2	-4	-4.0	-2.0	4.0	4
Cohort A3 Fed	Systolic blood pressure [mmHg]	BL		Raw	3	0	114.3	4.9	111	111.0	112.0	120.0	120
		D01	Pre-dose	Raw	3	0	112.7	3.5	109	109.0	113.0	116.0	116
				CFB	3	0	-1.7	3.2	-4	-4.0	-3.0	2.0	2
			1 h	Raw	3	0	105.0	5.3	99	99.0	107.0	109.0	109
				CFB	3	0	-9.3	4.7	-13	-13.0	-11.0	-4.0	-4
			2 h	Raw	3	0	107.0	7.2	101	101.0	105.0	115.0	115

n: Number of non-missing observations; %: Percentage based on non-missing observations; Miss: Missing observations; SD: Standard deviation; TP: Timepoint of measurement; SC: Screening; BL: Baseline; FUP: Follow-up; D: Day; Raw: Raw values; CFB: Change from baseline; Cohort A1: 200mg oral dose niclosamide; Cohort A2: 600mg oral dose niclosamide; Cohort A3: 1600mg oral dose niclosamide; Fast/Fed: Treatment in cohort A3 was applied under fasting and fed conditions in the same subjects;

Output generated by program 'NIC002_T14_6_VitalSigns_V02_0_0'

Table 14.6: Vital signs

Part A

Cohort	Parameter	Visit	TP		n	Miss	Mean	SD	Minimum	Lower quartile	Median	Upper quartile	Maximum
Cohort A3 Fed	Systolic blood pressure [mmHg]	D01	2 h	CFB	3	0	-7.3	10.0	-15	-15.0	-11.0	4.0	4
			4 h	Raw	3	0	109.7	4.0	105	105.0	112.0	112.0	112
				CFB	3	0	-4.7	9.0	-15	-15.0	0.0	1.0	1
			8 h	Raw	3	0	113.7	6.4	110	110.0	110.0	121.0	121
				CFB	3	0	-0.7	1.5	-2	-2.0	-1.0	1.0	1
			12 h	Raw	3	0	109.7	15.0	95	95.0	109.0	125.0	125
				CFB	3	0	-4.7	11.2	-17	-17.0	-2.0	5.0	5
		FUP		Raw	3	0	111.3	7.8	105	105.0	109.0	120.0	120
				CFB	3	0	-3.0	3.0	-6	-6.0	-3.0	0.0	0
	Diastolic blood pressure [mmHg]	BL		Raw	3	0	61.3	8.4	56	56.0	57.0	71.0	71
		D01	Pre-dose	Raw	3	0	60.7	7.4	55	55.0	58.0	69.0	69
				CFB	3	0	-0.7	1.5	-2	-2.0	-1.0	1.0	1
			1 h	Raw	3	0	59.7	7.5	52	52.0	60.0	67.0	67
				CFB	3	0	-1.7	4.9	-5	-5.0	-4.0	4.0	4
			2 h	Raw	3	0	59.3	4.5	55	55.0	59.0	64.0	64
				CFB	3	0	-2.0	4.6	-7	-7.0	-1.0	2.0	2
			4 h	Raw	3	0	58.0	2.0	56	56.0	58.0	60.0	60
				CFB	3	0	-3.3	6.8	-11	-11.0	-1.0	2.0	2
			8 h	Raw	3	0	60.0	4.4	55	55.0	62.0	63.0	63
				CFB	3	0	-1.3	7.5	-9	-9.0	-1.0	6.0	6
			12 h	Raw	3	0	60.7	11.6	53	53.0	55.0	74.0	74
				CFB	3	0	-0.7	3.2	-3	-3.0	-2.0	3.0	3
		FUP		Raw	3	0	61.0	8.7	56	56.0	56.0	71.0	71

n: Number of non-missing observations; %: Percentage based on non-missing observations; Miss: Missing observations; SD: Standard deviation; TP: Timepoint of measurement; SC: Screening; BL: Baseline; FUP: Follow-up; D: Day; Raw: Raw values; CFB: Change from baseline; Cohort A1: 200mg oral dose niclosamide; Cohort A2: 600mg oral dose niclosamide; Cohort A3: 1600mg oral dose niclosamide; Fast/Fed: Treatment in cohort A3 was applied under fasting and fed conditions in the same subjects;

Output generated by program 'NIC002_T14_6_VitalSigns_V02_0_0'

Table 14.6: Vital signs

Part A

Cohort	Parameter	Visit	TP		n	Miss	Mean	SD	Minimum	Lower quartile	Median	Upper quartile	Maximum
	Diastolic blood pressure [mmHg]	FUP		CFB	3	0	-0.3	0.6	-1	-1.0	0.0	0.0	0
	Heart rate [beats/min]	BL		Raw	3	0	63.3	1.5	62	62.0	63.0	65.0	65
		D01	Pre-dose	Raw	3	0	59.7	4.0	56	56.0	59.0	64.0	64
				CFB	3	0	-3.7	3.1	-7	-7.0	-3.0	-1.0	-1
			1 h	Raw	3	0	71.7	4.7	68	68.0	70.0	77.0	77
				CFB	3	0	8.3	3.2	6	6.0	7.0	12.0	12
			2 h	Raw	3	0	75.3	3.2	73	73.0	74.0	79.0	79
				CFB	3	0	12.0	2.0	10	10.0	12.0	14.0	14
			4 h	Raw	3	0	68.7	3.5	65	65.0	69.0	72.0	72
				CFB	3	0	5.3	4.2	2	2.0	4.0	10.0	10
			8 h	Raw	3	0	71.0	8.7	65	65.0	67.0	81.0	81
				CFB	3	0	7.7	7.2	3	3.0	4.0	16.0	16
			12 h	Raw	3	0	65.7	1.5	64	64.0	66.0	67.0	67
				CFB	3	0	2.3	0.6	2	2.0	2.0	3.0	3
		FUP		Raw	3	0	68.0	11.0	57	57.0	68.0	79.0	79
				CFB	3	0	4.7	10.6	-5	-5.0	3.0	16.0	16
	Body temperature [°C]	BL		Raw	3	0	36.23	0.61	35.7	35.70	36.10	36.90	36.9
		D01	Pre-dose	Raw	3	0	36.93	0.15	36.8	36.80	36.90	37.10	37.1
				CFB	3	0	0.70	0.50	0.2	0.20	0.70	1.20	1.2
			3 h	Raw	3	0	37.20	0.26	36.9	36.90	37.30	37.40	37.4
				CFB	3	0	0.97	0.57	0.5	0.50	0.80	1.60	1.6
		FUP		Raw	3	0	36.23	0.60	35.6	35.60	36.30	36.80	36.8

n: Number of non-missing observations; %: Percentage based on non-missing observations; Miss: Missing observations; SD: Standard deviation; TP: Timepoint of measurement; SC: Screening; BL: Baseline; FUP: Follow-up; D: Day; Raw: Raw values; CFB: Change from baseline; Cohort A1: 200mg oral dose niclosamide; Cohort A2: 600mg oral dose niclosamide; Cohort A3: 1600mg oral dose niclosamide; Fast/Fed: Treatment in cohort A3 was applied under fasting and fed conditions in the same subjects;

Output generated by program 'NIC002_T14_6_VitalSigns_V02_0_0'

Table 14.6: Vital signs

Part A

Cohort	Parameter	Visit	TP		n	Miss	Mean	SD	Minimum	Lower quartile	Median	Upper quartile	Maximum
	Body temperature [°C]	FUP		CFB	3	0	-0.00	0.56	-0.5	-0.50	-0.10	0.60	0.6
	Respiration [breaths/min]	BL		Raw	3	0	14.0	0.0	14	14.0	14.0	14.0	14
		D01	Pre-dose	Raw	3	0	16.7	1.5	15	15.0	17.0	18.0	18
				CFB	3	0	2.7	1.5	1	1.0	3.0	4.0	4
			1 h	Raw	3	0	15.0	1.7	13	13.0	16.0	16.0	16
				CFB	3	0	1.0	1.7	-1	-1.0	2.0	2.0	2
			2 h	Raw	3	0	15.3	0.6	15	15.0	15.0	16.0	16
				CFB	3	0	1.3	0.6	1	1.0	1.0	2.0	2
			4 h	Raw	3	0	14.7	1.2	14	14.0	14.0	16.0	16
				CFB	3	0	0.7	1.2	0	0.0	0.0	2.0	2
			8 h	Raw	3	0	12.7	0.6	12	12.0	13.0	13.0	13
				CFB	3	0	-1.3	0.6	-2	-2.0	-1.0	-1.0	-1
			12 h	Raw	3	0	12.3	0.6	12	12.0	12.0	13.0	13
				CFB	3	0	-1.7	0.6	-2	-2.0	-2.0	-1.0	-1
		FUP		Raw	3	0	13.3	1.2	12	12.0	14.0	14.0	14
				CFB	3	0	-0.7	1.2	-2	-2.0	0.0	0.0	0
Placebo	Systolic blood pressure [mmHg]	SC		Raw	3	0	109.0	5.6	103	103.0	110.0	114.0	114
		BL		Raw	3	0	121.3	9.5	112	112.0	121.0	131.0	131
		D01	Pre-dose	Raw	3	0	113.7	10.0	106	106.0	110.0	125.0	125
				CFB	3	0	-7.7	15.3	-25	-25.0	-2.0	4.0	4
			1 h	Raw	3	0	107.7	5.5	102	102.0	108.0	113.0	113

n: Number of non-missing observations; %: Percentage based on non-missing observations; Miss: Missing observations; SD: Standard deviation; TP: Timepoint of measurement; SC: Screening; BL: Baseline; FUP: Follow-up; D: Day; Raw: Raw values; CFB: Change from baseline; Cohort A1: 200mg oral dose niclosamide; Cohort A2: 600mg oral dose niclosamide; Cohort A3: 1600mg oral dose niclosamide; Fast/Fed: Treatment in cohort A3 was applied under fasting and fed conditions in the same subjects;

Output generated by program 'NIC002_T14_6_VitalSigns_V02_0_0'

Table 14.6: Vital signs

Part A

Cohort	Parameter	Visit	TP		n	Miss	Mean	SD	Minimum	Lower quartile	Median	Upper quartile	Maximum
Placebo	Systolic blood pressure [mmHg]	D01	1 h	CFB	3	0	-13.7	8.1	-23	-23.0	-10.0	-8.0	-8
			2 h	Raw	3	0	109.0	7.9	103	103.0	106.0	118.0	118
				CFB	3	0	-12.3	11.4	-25	-25.0	-9.0	-3.0	-3
			4 h	Raw	3	0	106.0	6.1	102	102.0	103.0	113.0	113
				CFB	3	0	-15.3	11.0	-28	-28.0	-10.0	-8.0	-8
			8 h	Raw	3	0	110.3	7.0	103	103.0	111.0	117.0	117
				CFB	3	0	-11.0	8.2	-20	-20.0	-9.0	-4.0	-4
			12 h	Raw	3	0	113.7	3.1	111	111.0	113.0	117.0	117
				CFB	3	0	-7.7	11.0	-20	-20.0	-4.0	1.0	1
		FUP		Raw	3	0	111.3	6.7	104	104.0	113.0	117.0	117
				CFB	3	0	-10.0	14.9	-27	-27.0	-4.0	1.0	1
	Diastolic blood pressure [mmHg]	SC		Raw	3	0	65.7	3.8	63	63.0	64.0	70.0	70
		BL		Raw	3	0	72.0	7.5	65	65.0	71.0	80.0	80
		D01	Pre-dose	Raw	3	0	68.0	8.7	62	62.0	64.0	78.0	78
				CFB	3	0	-4.0	11.5	-16	-16.0	-3.0	7.0	7
			1 h	Raw	3	0	65.0	6.2	58	58.0	67.0	70.0	70
				CFB	3	0	-7.0	3.0	-10	-10.0	-7.0	-4.0	-4
			2 h	Raw	3	0	64.3	2.1	62	62.0	65.0	66.0	66
				CFB	3	0	-7.7	5.7	-14	-14.0	-6.0	-3.0	-3
			4 h	Raw	3	0	64.3	6.7	57	57.0	66.0	70.0	70
				CFB	3	0	-7.7	2.5	-10	-10.0	-8.0	-5.0	-5
			8 h	Raw	3	0	66.3	6.0	60	60.0	67.0	72.0	72
				CFB	3	0	-5.7	7.0	-13	-13.0	-5.0	1.0	1

n: Number of non-missing observations; %: Percentage based on non-missing observations; Miss: Missing observations; SD: Standard deviation; TP: Timepoint of measurement; SC: Screening; BL: Baseline; FUP: Follow-up; D: Day; Raw: Raw values; CFB: Change from baseline; Cohort A1: 200mg oral dose niclosamide; Cohort A2: 600mg oral dose niclosamide; Cohort A3: 1600mg oral dose niclosamide; Fast/Fed: Treatment in cohort A3 was applied under fasting and fed conditions in the same subjects;

Output generated by program 'NIC002_T14_6_VitalSigns_V02_0_0'

Table 14.6: Vital signs

Part A

Cohort	Parameter	Visit	TP		n	Miss	Mean	SD	Minimum	Lower quartile	Median	Upper quartile	Maximum
	Diastolic blood pressure [mmHg]		12 h	Raw	3	0	67.7	2.3	65	65.0	69.0	69.0	69
				CFB	3	0	-4.3	5.9	-11	-11.0	-2.0	0.0	0
		FUP		Raw	3	0	65.3	3.1	62	62.0	66.0	68.0	68
				CFB	3	0	-6.7	4.7	-12	-12.0	-5.0	-3.0	-3
	Heart rate [beats/min]	SC		Raw	3	0	68.0	7.8	63	63.0	64.0	77.0	77
		BL		Raw	3	0	71.0	6.0	65	65.0	71.0	77.0	77
		D01	Pre-dose	Raw	3	0	64.0	7.5	56	56.0	65.0	71.0	71
				CFB	3	0	-7.0	6.2	-12	-12.0	-9.0	0.0	0
			1 h	Raw	3	0	60.3	6.8	55	55.0	58.0	68.0	68
				CFB	3	0	-10.7	8.0	-19	-19.0	-10.0	-3.0	-3
			2 h	Raw	3	0	57.7	5.9	51	51.0	60.0	62.0	62
				CFB	3	0	-13.3	4.0	-17	-17.0	-14.0	-9.0	-9
			4 h	Raw	3	0	58.0	8.7	52	52.0	54.0	68.0	68
				CFB	3	0	-13.0	10.0	-23	-23.0	-13.0	-3.0	-3
			8 h	Raw	3	0	63.7	6.7	58	58.0	62.0	71.0	71
				CFB	3	0	-7.3	7.5	-15	-15.0	-7.0	0.0	0
			12 h	Raw	3	0	67.0	8.7	57	57.0	72.0	72.0	72
				CFB	3	0	-4.0	4.6	-8	-8.0	-5.0	1.0	1
		FUP		Raw	3	0	70.0	3.0	67	67.0	70.0	73.0	73
				CFB	3	0	-1.0	5.2	-7	-7.0	2.0	2.0	2
	Body temperature [°C]	SC		Raw	3	0	36.67	0.12	36.6	36.60	36.60	36.80	36.8
		BL		Raw	3	0	36.50	0.46	36.0	36.00	36.60	36.90	36.9

n: Number of non-missing observations; %: Percentage based on non-missing observations; Miss: Missing observations; SD: Standard deviation; TP: Timepoint of measurement; SC: Screening; BL: Baseline; FUP: Follow-up; D: Day; Raw: Raw values; CFB: Change from baseline; Cohort A1: 200mg oral dose niclosamide; Cohort A2: 600mg oral dose niclosamide; Cohort A3: 1600mg oral dose niclosamide; Fast/Fed: Treatment in cohort A3 was applied under fasting and fed conditions in the same subjects;

Output generated by program 'NIC002_T14_6_VitalSigns_V02_0_0'

Table 14.6: Vital signs

Part A

Cohort	Parameter	Visit	TP		n	Miss	Mean	SD	Minimum	Lower quartile	Median	Upper quartile	Maximum
	Body temperature [°C]	D01	Pre-dose	Raw	3	0	36.77	0.25	36.5	36.50	36.80	37.00	37.0
				CFB	3	0	0.27	0.32	-0.1	-0.10	0.40	0.50	0.5
			3 h	Raw	3	0	36.90	0.00	36.9	36.90	36.90	36.90	36.9
				CFB	3	0	0.40	0.46	0.0	0.00	0.30	0.90	0.9
		FUP		Raw	3	0	35.70	0.61	35.3	35.30	35.40	36.40	36.4
				CFB	3	0	-0.80	1.04	-1.5	-1.50	-1.30	0.40	0.4
	Respiration [breaths/min]	SC		Raw	3	0	13.3	2.3	12	12.0	12.0	16.0	16
		BL		Raw	3	0	14.0	1.7	13	13.0	13.0	16.0	16
		D01	Pre-dose	Raw	3	0	14.0	1.7	13	13.0	13.0	16.0	16
				CFB	3	0	0.0	0.0	0	0.0	0.0	0.0	0
			1 h	Raw	3	0	15.0	1.0	14	14.0	15.0	16.0	16
				CFB	3	0	1.0	1.0	0	0.0	1.0	2.0	2
			2 h	Raw	3	0	14.3	1.5	13	13.0	14.0	16.0	16
				CFB	3	0	0.3	0.6	0	0.0	0.0	1.0	1
			4 h	Raw	3	0	13.7	1.2	13	13.0	13.0	15.0	15
				CFB	3	0	-0.3	0.6	-1	-1.0	0.0	0.0	0
			8 h	Raw	3	0	12.3	0.6	12	12.0	12.0	13.0	13
				CFB	3	0	-1.7	2.1	-4	-4.0	-1.0	0.0	0
			12 h	Raw	3	0	14.0	0.0	14	14.0	14.0	14.0	14
				CFB	3	0	0.0	1.7	-2	-2.0	1.0	1.0	1
		FUP		Raw	3	0	12.7	1.2	12	12.0	12.0	14.0	14
				CFB	3	0	-1.3	2.5	-4	-4.0	-1.0	1.0	1

n: Number of non-missing observations; %: Percentage based on non-missing observations; Miss: Missing observations; SD: Standard deviation; TP: Timepoint of measurement; SC: Screening; BL: Baseline; FUP: Follow-up; D: Day; Raw: Raw values; CFB: Change from baseline; Cohort A1: 200mg oral dose niclosamide; Cohort A2: 600mg oral dose niclosamide; Cohort A3: 1600mg oral dose niclosamide; Fast/Fed: Treatment in cohort A3 was applied under fasting and fed conditions in the same subjects;

Output generated by program 'NIC002_T14_6_VitalSigns_V02_0_0'

Table 14.6: Vital signs

Part A

Cohort	Parameter	Visit	TP		n	Miss	Mean	SD	Minimum	Lower quartile	Median	Upper quartile	Maximum
Placebo Fed	Systolic blood pressure [mmHg]	BL		Raw	1	0	120.0		120	120.0	120.0	120.0	120
		D01	Pre-dose	Raw	1	0	120.0		120	120.0	120.0	120.0	120
				CFB	1	0	0.0		0	0.0	0.0	0.0	0
			1 h	Raw	1	0	122.0		122	122.0	122.0	122.0	122
				CFB	1	0	2.0		2	2.0	2.0	2.0	2
			2 h	Raw	1	0	116.0		116	116.0	116.0	116.0	116
				CFB	1	0	-4.0		-4	-4.0	-4.0	-4.0	-4
			4 h	Raw	1	0	111.0		111	111.0	111.0	111.0	111
				CFB	1	0	-9.0		-9	-9.0	-9.0	-9.0	-9
			8 h	Raw	1	0	117.0		117	117.0	117.0	117.0	117
				CFB	1	0	-3.0		-3	-3.0	-3.0	-3.0	-3
			12 h	Raw	1	0	119.0		119	119.0	119.0	119.0	119
				CFB	1	0	-1.0		-1	-1.0	-1.0	-1.0	-1
		FUP		Raw	1	0	131.0		131	131.0	131.0	131.0	131
				CFB	1	0	11.0		11	11.0	11.0	11.0	11
	Diastolic blood pressure [mmHg]	BL		Raw	1	0	63.0		63	63.0	63.0	63.0	63
		D01	Pre-dose	Raw	1	0	74.0		74	74.0	74.0	74.0	74
				CFB	1	0	11.0		11	11.0	11.0	11.0	11
			1 h	Raw	1	0	68.0		68	68.0	68.0	68.0	68
				CFB	1	0	5.0		5	5.0	5.0	5.0	5

n: Number of non-missing observations; %: Percentage based on non-missing observations; Miss: Missing observations; SD: Standard deviation; TP: Timepoint of measurement; SC: Screening; BL: Baseline; FUP: Follow-up; D: Day; Raw: Raw values; CFB: Change from baseline; Cohort A1: 200mg oral dose niclosamide; Cohort A2: 600mg oral dose niclosamide; Cohort A3: 1600mg oral dose niclosamide; Fast/Fed: Treatment in cohort A3 was applied under fasting and fed conditions in the same subjects;

Output generated by program 'NIC002_T14_6_VitalSigns_V02_0_0'

Table 14.6: Vital signs

Part A

Cohort	Parameter	Visit	TP		n	Miss	Mean	SD	Minimum	Lower quartile	Median	Upper quartile	Maximum
	Diastolic blood pressure [mmHg]	D01	2 h	Raw	1	0	59.0		59	59.0	59.0	59.0	59
				CFB	1	0	-4.0		-4	-4.0	-4.0	-4.0	-4
			4 h	Raw	1	0	70.0		70	70.0	70.0	70.0	70
				CFB	1	0	7.0		7	7.0	7.0	7.0	7
			8 h	Raw	1	0	72.0		72	72.0	72.0	72.0	72
				CFB	1	0	9.0		9	9.0	9.0	9.0	9
			12 h	Raw	1	0	71.0		71	71.0	71.0	71.0	71
				CFB	1	0	8.0		8	8.0	8.0	8.0	8
		FUP		Raw	1	0	58.0		58	58.0	58.0	58.0	58
				CFB	1	0	-5.0		-5	-5.0	-5.0	-5.0	-5
	Heart rate [beats/min]	BL		Raw	1	0	67.0		67	67.0	67.0	67.0	67
		D01	Pre-dose	Raw	1	0	62.0		62	62.0	62.0	62.0	62
				CFB	1	0	-5.0		-5	-5.0	-5.0	-5.0	-5
			1 h	Raw	1	0	61.0		61	61.0	61.0	61.0	61
				CFB	1	0	-6.0		-6	-6.0	-6.0	-6.0	-6
			2 h	Raw	1	0	60.0		60	60.0	60.0	60.0	60
				CFB	1	0	-7.0		-7	-7.0	-7.0	-7.0	-7
			4 h	Raw	1	0	50.0		50	50.0	50.0	50.0	50
				CFB	1	0	-17.0		-17	-17.0	-17.0	-17.0	-17
			8 h	Raw	1	0	56.0		56	56.0	56.0	56.0	56
				CFB	1	0	-11.0		-11	-11.0	-11.0	-11.0	-11
			12 h	Raw	1	0	65.0		65	65.0	65.0	65.0	65
				CFB	1	0	-2.0		-2	-2.0	-2.0	-2.0	-2
		FUP		Raw	1	0	60.0		60	60.0	60.0	60.0	60
				CFB	1	0	-7.0		-7	-7.0	-7.0	-7.0	-7

n: Number of non-missing observations; %: Percentage based on non-missing observations; Miss: Missing observations; SD: Standard deviation; TP: Timepoint of measurement; SC: Screening; BL: Baseline; FUP: Follow-up; D: Day; Raw: Raw values; CFB: Change from baseline; Cohort A1: 200mg oral dose niclosamide; Cohort A2: 600mg oral dose niclosamide; Cohort A3: 1600mg oral dose niclosamide; Fast/Fed: Treatment in cohort A3 was applied under fasting and fed conditions in the same subjects;

Output generated by program 'NIC002_T14_6_VitalSigns_V02_0_0'

Table 14.6: Vital signs

Part A

Cohort	Parameter	Visit	TP		n	Miss	Mean	SD	Minimum	Lower quartile	Median	Upper quartile	Maximum
	Body temperature [°C]	BL		Raw	1	0	35.00		35.0	35.00	35.00	35.00	35.0
		D01	Pre-dose	Raw	1	0	36.60		36.6	36.60	36.60	36.60	36.6
				CFB	1	0	1.60		1.6	1.60	1.60	1.60	1.6
			3 h	Raw	1	0	36.30		36.3	36.30	36.30	36.30	36.3
				CFB	1	0	1.30		1.3	1.30	1.30	1.30	1.3
		FUP		Raw	1	0	36.50		36.5	36.50	36.50	36.50	36.5
				CFB	1	0	1.50		1.5	1.50	1.50	1.50	1.5
	Respiration [breaths/min]	BL		Raw	1	0	15.0		15	15.0	15.0	15.0	15
		D01	Pre-dose	Raw	1	0	16.0		16	16.0	16.0	16.0	16
				CFB	1	0	1.0		1	1.0	1.0	1.0	1
			1 h	Raw	1	0	15.0		15	15.0	15.0	15.0	15
				CFB	1	0	0.0		0	0.0	0.0	0.0	0
			2 h	Raw	1	0	16.0		16	16.0	16.0	16.0	16
				CFB	1	0	1.0		1	1.0	1.0	1.0	1
			4 h	Raw	1	0	13.0		13	13.0	13.0	13.0	13
				CFB	1	0	-2.0		-2	-2.0	-2.0	-2.0	-2
			8 h	Raw	1	0	14.0		14	14.0	14.0	14.0	14
				CFB	1	0	-1.0		-1	-1.0	-1.0	-1.0	-1
			12 h	Raw	1	0	15.0		15	15.0	15.0	15.0	15
				CFB	1	0	0.0		0	0.0	0.0	0.0	0
		FUP		Raw	1	0	13.0		13	13.0	13.0	13.0	13
				CFB	1	0	-2.0		-2	-2.0	-2.0	-2.0	-2

n: Number of non-missing observations; %: Percentage based on non-missing observations; Miss: Missing observations; SD: Standard deviation; TP: Timepoint of measurement; SC: Screening; BL: Baseline; FUP: Follow-up; D: Day; Raw: Raw values; CFB: Change from baseline; Cohort A1: 200mg oral dose niclosamide; Cohort A2: 600mg oral dose niclosamide; Cohort A3: 1600mg oral dose niclosamide; Fast/Fed: Treatment in cohort A3 was applied under fasting and fed conditions in the same subjects;

Output generated by program 'NIC002_T14_6_VitalSigns_V02_0_0'

Table 14.6: Vital signs

Part B

Parameter	Visit	Treatment	TP		n	Miss	Mean	SD	Minimum	Lower quartile	Median	Upper quartile	Maximum
Systolic blood pressure [mmHg]	SC			Raw	4	0	116.5	9.1	107	109.0	116.0	124.0	127
	BL			Raw	4	0	117.8	12.7	104	108.5	116.5	127.0	134
	D01	Chewing tablet 2000 mg	Pre-dose	Raw	4	0	114.0	8.5	107	108.0	111.5	120.0	126
				CFB	4	0	-3.8	5.9	-8	-7.0	-6.0	-0.5	5
			1 h	Raw	4	0	109.5	13.6	98	100.5	105.5	118.5	129
				CFB	4	0	-8.3	5.9	-17	-11.5	-5.5	-5.0	-5
			2 h	Raw	4	0	109.8	11.5	97	102.5	108.5	117.0	125
				CFB	4	0	-8.0	3.4	-12	-10.5	-8.0	-5.5	-4
			4 h	Raw	4	0	115.3	9.6	103	108.5	116.0	122.0	126
				CFB	4	0	-2.5	12.1	-20	-10.5	2.0	5.5	6
			8 h	Raw	4	0	107.8	12.0	96	98.0	106.5	117.5	122
				CFB	4	0	-10.0	8.3	-20	-16.0	-10.0	-4.0	0
			12 h	Raw	4	0	113.0	9.0	104	105.5	112.5	120.5	123
				CFB	4	0	-4.8	12.0	-16	-14.5	-6.5	5.0	10
		Solution 1600 mg	Pre-dose	Raw	4	0	108.8	5.2	104	104.5	108.0	113.0	115
				CFB	4	0	-9.0	7.8	-19	-14.0	-8.5	-4.0	0
			1 h	Raw	4	0	109.8	8.9	104	105.0	106.0	114.5	123
				CFB	4	0	-8.0	6.1	-14	-12.5	-9.0	-3.5	0
			2 h	Raw	4	0	107.0	10.8	92	99.5	109.5	114.5	117
				CFB	4	0	-10.8	10.7	-21	-19.0	-12.5	-2.5	3
			4 h	Raw	4	0	104.5	7.1	95	99.0	106.5	110.0	110
				CFB	4	0	-13.3	10.0	-24	-21.0	-14.0	-5.5	-1
			8 h	Raw	4	0	107.5	7.6	100	101.0	107.5	114.0	115
				CFB	4	0	-10.3	7.4	-19	-16.0	-10.0	-4.5	-2

n: Number of non-missing observations; %: Percentage based on non-missing observations; Miss: Missing observations; SD: Standard deviation; TP: Timepoint of measurement; SC: Screening; BL: Baseline; FUP: Follow-up; D: Day; Raw: Raw values; CFB: Change from baseline; Part B used a cross-over design;

Output generated by program 'NIC002_T14_6_VitalSigns_V02_0_0'

Table 14.6: Vital signs

Part B

Parameter	Visit	Treatment	TP		n	Miss	Mean	SD	Minimum	Lower quartile	Median	Upper quartile	Maximum
Systolic blood pressure [mmHg]	D01		12 h	Raw	4	0	111.5	12.0	96	102.0	114.0	121.0	122
				CFB	4	0	-6.3	6.7	-14	-11.0	-6.5	-1.5	2
	FUP			Raw	4	0	112.5	9.9	105	106.5	109.0	118.5	127
				CFB	4	0	-5.3	7.8	-12	-10.0	-7.5	-0.5	6
Diastolic blood pressure [mmHg]	SC			Raw	4	0	72.5	9.3	59	66.5	76.0	78.5	79
	BL			Raw	4	0	70.8	8.4	63	63.5	71.0	78.0	78
	D01	Chewing tablet 2000 mg	Pre-dose	Raw	4	0	67.5	10.7	56	59.5	66.5	75.5	81
				CFB	4	0	-3.3	5.2	-8	-7.5	-4.0	1.0	3
			1 h	Raw	4	0	62.0	6.7	58	58.0	59.0	66.0	72
				CFB	4	0	-8.8	6.2	-18	-12.0	-6.0	-5.5	-5
			2 h	Raw	4	0	58.5	6.0	52	53.5	58.5	63.5	65
				CFB	4	0	-12.3	3.3	-16	-14.5	-12.5	-10.0	-8
			4 h	Raw	4	0	68.3	6.7	61	63.5	67.5	73.0	77
				CFB	4	0	-2.5	4.7	-9	-5.5	-1.5	0.5	2
			8 h	Raw	4	0	66.8	5.9	59	62.5	67.5	71.0	73
				CFB	4	0	-4.0	5.0	-9	-7.0	-5.0	-1.0	3
			12 h	Raw	4	0	59.8	5.0	56	56.5	58.0	63.0	67
				CFB	4	0	-11.0	7.3	-21	-16.0	-9.5	-6.0	-4
		Solution 1600 mg	Pre-dose	Raw	4	0	62.3	9.7	52	54.0	62.5	70.5	72
				CFB	4	0	-8.5	2.1	-11	-10.0	-8.5	-7.0	-6
			1 h	Raw	4	0	66.8	9.1	56	60.5	66.5	73.0	78
				CFB	4	0	-4.0	5.4	-10	-8.5	-3.5	0.5	1
			2 h	Raw	4	0	63.3	6.7	54	59.0	64.5	67.5	70
				CFB	4	0	-7.5	6.2	-14	-11.5	-8.5	-3.5	1

n: Number of non-missing observations; %: Percentage based on non-missing observations; Miss: Missing observations; SD: Standard deviation; TP: Timepoint of measurement; SC: Screening; BL: Baseline; FUP: Follow-up; D: Day; Raw: Raw values; CFB: Change from baseline; Part B used a cross-over design;

Output generated by program 'NIC002_T14_6_VitalSigns_V02_0_0'

Table 14.6: Vital signs

Part B

Parameter	Visit	Treatment	TP		n	Miss	Mean	SD	Minimum	Lower quartile	Median	Upper quartile	Maximum
Diastolic blood pressure [mmHg]	D01	Solution 1600 mg	4 h	Raw	4	0	60.3	9.6	51	52.0	60.5	68.5	69
				CFB	4	0	-10.5	1.3	-12	-11.5	-10.5	-9.5	-9
			8 h	Raw	4	0	63.3	10.4	49	57.0	65.0	69.5	74
				CFB	4	0	-7.5	7.2	-14	-13.5	-8.5	-1.5	1
			12 h	Raw	4	0	63.5	11.7	52	53.5	63.5	73.5	75
				CFB	4	0	-7.3	3.8	-12	-10.0	-7.0	-4.5	-3
	FUP			Raw	4	0	66.0	6.8	57	61.0	67.0	71.0	73
				CFB	4	0	-4.8	4.2	-9	-7.5	-5.5	-2.0	1
Heart rate [beats/min]	SC			Raw	4	0	72.0	13.2	59	61.0	71.0	83.0	87
	BL			Raw	4	0	79.0	19.1	58	63.0	80.0	95.0	98
	D01	Chewing tablet 2000 mg	Pre-dose	Raw	4	0	66.0	12.2	51	56.0	68.5	76.0	76
				CFB	4	0	-13.0	7.3	-22	-19.0	-11.5	-7.0	-7
			1 h	Raw	4	0	69.3	10.5	57	60.5	70.5	78.0	79
				CFB	4	0	-9.8	8.6	-19	-17.0	-9.5	-2.5	-1
			2 h	Raw	4	0	66.8	11.3	53	58.0	67.5	75.5	79
				CFB	4	0	-12.3	9.9	-26	-19.5	-9.0	-5.0	-5
			4 h	Raw	4	0	67.8	14.5	50	57.5	68.0	78.0	85
				CFB	4	0	-11.3	7.7	-21	-17.0	-10.5	-5.5	-3
			8 h	Raw	4	0	70.5	18.1	50	59.0	69.0	82.0	94
				CFB	4	0	-8.5	11.1	-24	-16.0	-6.0	-1.0	2
			12 h	Raw	4	0	70.8	13.7	56	59.5	70.5	82.0	86
				CFB	4	0	-8.3	8.0	-20	-13.0	-5.5	-3.5	-2
		Solution 1600 mg	Pre-dose	Raw	4	0	69.5	12.9	51	62.0	73.0	77.0	81
				CFB	4	0	-9.5	11.0	-19	-18.0	-12.0	-1.0	5

n: Number of non-missing observations; %: Percentage based on non-missing observations; Miss: Missing observations; SD: Standard deviation; TP: Timepoint of measurement; SC: Screening; BL: Baseline; FUP: Follow-up; D: Day; Raw: Raw values; CFB: Change from baseline; Part B used a cross-over design;

Output generated by program 'NIC002_T14_6_VitalSigns_V02_0_0'

Table 14.6: Vital signs

Part B

Parameter	Visit	Treatment	TP		n	Miss	Mean	SD	Minimum	Lower quartile	Median	Upper quartile	Maximum
Heart rate [beats/min]	D01	Solution 1600 mg	1 h	Raw	4	0	72.8	15.3	55	60.0	74.5	85.5	87
				CFB	4	0	-6.3	3.9	-11	-9.5	-5.5	-3.0	-3
			2 h	Raw	4	0	69.8	14.4	54	58.0	69.5	81.5	86
				CFB	4	0	-9.3	5.1	-15	-13.5	-9.0	-5.0	-4
			4 h	Raw	4	0	65.0	13.5	48	54.5	66.5	75.5	79
				CFB	4	0	-14.0	6.5	-20	-19.5	-14.5	-8.5	-7
			8 h	Raw	4	0	68.5	11.2	55	59.5	69.5	77.5	80
				CFB	4	0	-10.5	8.1	-18	-17.5	-10.5	-3.5	-3
			12 h	Raw	4	0	69.3	12.4	58	59.0	67.5	79.5	84
				CFB	4	0	-9.8	8.3	-17	-15.5	-12.0	-4.0	2
	FUP			Raw	4	0	78.5	11.5	62	71.0	82.0	86.0	88
				CFB	4	0	-0.5	11.1	-14	-9.0	0.0	8.0	12
Body temperature [°C]	SC			Raw	4	0	36.73	0.21	36.5	36.55	36.75	36.90	36.9
	BL			Raw	4	0	36.30	0.22	36.0	36.15	36.35	36.45	36.5
	D01	Chewing tablet 2000 mg	Pre-dose	Raw	4	0	37.05	0.37	36.7	36.75	37.00	37.35	37.5
				CFB	4	0	0.75	0.25	0.4	0.60	0.80	0.90	1.0
			3 h	Raw	4	0	37.05	0.31	36.7	36.80	37.05	37.30	37.4
				CFB	4	0	0.75	0.24	0.4	0.60	0.85	0.90	0.9
		Solution 1600 mg	Pre-dose	Raw	4	0	37.08	0.39	36.7	36.75	37.05	37.40	37.5
				CFB	4	0	0.78	0.29	0.4	0.60	0.80	0.95	1.1
			3 h	Raw	4	0	37.20	0.29	36.8	37.00	37.25	37.40	37.5
				CFB	4	0	0.90	0.22	0.7	0.75	0.85	1.05	1.2
	FUP			Raw	4	0	35.95	0.13	35.8	35.85	35.95	36.05	36.1
				CFB	4	0	-0.35	0.31	-0.6	-0.55	-0.45	-0.15	0.1

n: Number of non-missing observations; %: Percentage based on non-missing observations; Miss: Missing observations; SD: Standard deviation; TP: Timepoint of measurement; SC: Screening; BL: Baseline; FUP: Follow-up; D: Day; Raw: Raw values; CFB: Change from baseline; Part B used a cross-over design;

Output generated by program 'NIC002_T14_6_VitalSigns_V02_0_0'

Table 14.6: Vital signs

Part B

Parameter	Visit	Treatment	TP		n	Miss	Mean	SD	Minimum	Lower quartile	Median	Upper quartile	Maximum
Respiration [breaths/min]	SC			Raw	4	0	14.0	0.8	13	13.5	14.0	14.5	15
	BL			Raw	4	0	14.0	2.8	12	12.0	13.0	16.0	18
	D01	Chewing tablet 2000 mg	Pre-dose	Raw	4	0	15.0	1.4	13	14.0	15.5	16.0	16
				CFB	4	0	1.0	2.9	-3	-1.0	1.5	3.0	4
			1 h	Raw	4	0	14.8	2.5	12	13.0	14.5	16.5	18
				CFB	4	0	0.8	4.6	-6	-2.0	2.5	3.5	4
			2 h	Raw	4	0	13.8	1.3	12	13.0	14.0	14.5	15
				CFB	4	0	-0.3	3.9	-6	-2.5	1.5	2.0	2
			4 h	Raw	4	0	13.8	0.5	13	13.5	14.0	14.0	14
				CFB	4	0	-0.3	2.9	-4	-2.5	0.5	2.0	2
			8 h	Raw	4	0	13.8	0.5	13	13.5	14.0	14.0	14
				CFB	4	0	-0.3	2.6	-4	-2.0	0.5	1.5	2
			12 h	Raw	4	0	14.0	0.8	13	13.5	14.0	14.5	15
				CFB	4	0	0.0	2.2	-3	-1.5	0.5	1.5	2
		Solution 1600 mg	Pre-dose	Raw	4	0	13.8	1.0	13	13.0	13.5	14.5	15
				CFB	4	0	-0.3	3.4	-5	-2.5	0.5	2.0	3
			1 h	Raw	4	0	13.8	1.0	13	13.0	13.5	14.5	15
				CFB	4	0	-0.3	2.2	-3	-2.0	0.0	1.5	2
			2 h	Raw	4	0	12.8	1.3	11	12.0	13.0	13.5	14
				CFB	4	0	-1.3	2.6	-5	-3.0	-0.5	0.5	1
			4 h	Raw	4	0	13.5	0.6	13	13.0	13.5	14.0	14
				CFB	4	0	-0.5	2.6	-4	-2.5	0.0	1.5	2
			8 h	Raw	4	0	13.3	0.5	13	13.0	13.0	13.5	14
				CFB	4	0	-0.8	2.9	-5	-2.5	0.5	1.0	1
			12 h	Raw	4	0	14.3	1.0	13	13.5	14.5	15.0	15

n: Number of non-missing observations; %: Percentage based on non-missing observations; Miss: Missing observations; SD: Standard deviation; TP: Timepoint of measurement; SC: Screening; BL: Baseline; FUP: Follow-up; D: Day; Raw: Raw values; CFB: Change from baseline; Part B used a cross-over design;

Output generated by program 'NIC002_T14_6_VitalSigns_V02_0_0'

Table 14.6: Vital signs

Part B

Parameter	Visit	Treatment	TP		n	Miss	Mean	SD	Minimum	Lower quartile	Median	Upper quartile	Maximum
Respiration [breaths/min]	D01	Solution 1600 mg	12 h	CFB	4	0	0.3	2.2	-3	-1.0	1.0	1.5	2
	FUP			Raw	4	0	13.0	2.2	11	11.5	12.5	14.5	16
				CFB	4	0	-1.0	4.1	-6	-3.5	-1.0	1.5	4

n: Number of non-missing observations; %: Percentage based on non-missing observations; Miss: Missing observations; SD: Standard deviation; TP: Timepoint of measurement; SC: Screening; BL: Baseline; FUP: Follow-up; D: Day; Raw: Raw values; CFB: Change from baseline; Part B used a cross-over design;

Output generated by program 'NIC002_T14_6_VitalSigns_V02_0_0'

Table 14.6: Vital signs

Part C

Group	Parameter	Visit	TP		n	Miss	Mean	SD	Minimum	Lower quartile	Median	Upper quartile	Maximum
Group 1	Systolic blood pressure [mmHg]	SC		Raw	4	0	115.3	6.4	106	111.0	117.5	119.5	120
		BL		Raw	4	0	123.8	11.6	110	114.5	124.5	133.0	136
		D01	Pre-dose	Raw	4	0	114.0	7.4	109	109.5	111.0	118.5	125
				CFB	4	0	-9.8	10.0	-24	-16.5	-7.0	-3.0	-1
			1 h	Raw	4	0	110.5	5.2	104	106.5	111.0	114.5	116
				CFB	4	0	-13.3	9.9	-27	-20.5	-10.0	-6.0	-6
			2 h	Raw	4	0	110.5	8.2	99	105.0	112.5	116.0	118
				CFB	4	0	-13.3	10.0	-25	-20.5	-13.5	-6.0	-1
			4 h	Raw	4	0	105.8	8.2	94	101.0	108.0	110.5	113
				CFB	4	0	-18.0	7.2	-28	-22.5	-16.5	-13.5	-11
			8 h	Raw	4	0	106.3	11.4	90	98.5	110.0	114.0	115
				CFB	4	0	-17.5	9.1	-23	-23.0	-21.5	-12.0	-4
			12 h	Raw	4	0	109.3	5.7	103	104.5	109.5	114.0	115
				CFB	4	0	-14.5	11.7	-30	-23.5	-12.0	-5.5	-4
		D03		Raw	4	0	108.5	7.0	100	104.0	108.5	113.0	117
				CFB	4	0	-15.3	7.9	-27	-20.0	-12.0	-10.5	-10
		D08		Raw	4	0	105.8	6.7	96	102.0	108.0	109.5	111
				CFB	4	0	-18.0	8.8	-28	-25.0	-18.0	-11.0	-8
		FUP		Raw	4	0	112.8	8.6	103	105.5	114.0	120.0	120
				CFB	4	0	-11.0	12.2	-28	-19.0	-8.5	-3.0	1
	Diastolic blood pressure [mmHg]	SC		Raw	4	0	67.3	4.3	62	64.0	67.5	70.5	72
		BL		Raw	4	0	67.3	11.5	57	57.5	66.0	77.0	80

n: Number of non-missing observations; %: Percentage based on non-missing observations; Miss: Missing observations; SD: Standard deviation; TP: Timepoint of measurement; SC: Screening; BL: Baseline; FUP: Follow-up; D: Day; CFB: Change from baseline; Group 1: 1200 mg niclosamide solution; Group 2: 1600 mg niclosamide solution; Group 3: Placebo;

Output generated by program 'NIC002_T14_6_VitalSigns_V02_0_0'

Table 14.6: Vital signs

Part C

Group	Parameter	Visit	TP		n	Miss	Mean	SD	Minimum	Lower quartile	Median	Upper quartile	Maximum
	Diastolic blood pressure [mmHg]	D01	Pre-dose	Raw	4	0	63.0	12.1	51	54.0	61.0	72.0	79
				CFB	4	0	-4.3	3.9	-9	-7.5	-3.5	-1.0	-1
			1 h	Raw	4	0	63.3	7.6	57	58.0	61.0	68.5	74
				CFB	4	0	-4.0	5.6	-11	-8.5	-3.0	0.5	1
			2 h	Raw	4	0	61.8	9.5	52	54.5	60.5	69.0	74
				CFB	4	0	-5.5	3.7	-10	-8.0	-5.5	-3.0	-1
			4 h	Raw	4	0	62.8	9.5	52	56.5	62.0	69.0	75
				CFB	4	0	-4.5	5.7	-11	-8.0	-5.0	-1.0	3
			8 h	Raw	4	0	62.3	6.0	54	58.0	63.5	66.5	68
				CFB	4	0	-5.0	7.9	-15	-10.5	-4.5	0.5	4
			12 h	Raw	4	0	66.3	7.6	56	60.5	68.5	72.0	72
				CFB	4	0	-1.0	6.2	-8	-5.0	-1.5	3.0	7
		D03		Raw	4	0	65.0	5.4	62	62.0	62.5	68.0	73
				CFB	4	0	-2.3	8.6	-12	-9.5	-1.0	5.0	5
		D08		Raw	4	0	61.0	5.4	54	57.5	61.5	64.5	67
				CFB	4	0	-6.3	7.6	-13	-12.5	-7.5	0.0	3
		FUP		Raw	4	0	67.0	10.2	54	59.5	68.0	74.5	78
				CFB	4	0	-0.3	15.2	-15	-9.5	-3.5	9.0	21
	Heart rate [beats/min]	SC		Raw	4	0	76.8	7.8	67	70.5	78.0	83.0	84
		BL		Raw	4	0	74.5	7.2	66	68.5	75.5	80.5	81
		D01	Pre-dose	Raw	4	0	71.5	10.7	58	63.5	72.5	79.5	83
				CFB	4	0	-3.0	7.3	-13	-8.5	-1.0	2.5	3
			1 h	Raw	4	0	77.8	5.3	70	74.5	80.0	81.0	81
				CFB	4	0	3.3	5.1	-2	-0.5	2.5	7.0	10
			2 h	Raw	4	0	77.0	11.8	61	69.0	79.0	85.0	89
				CFB	4	0	2.5	10.5	-5	-4.0	-1.5	9.0	18

n: Number of non-missing observations; %: Percentage based on non-missing observations; Miss: Missing observations; SD: Standard deviation; TP: Timepoint of measurement; SC: Screening; BL: Baseline; FUP: Follow-up; D: Day; CFB: Change from baseline; Group 1: 1200 mg niclosamide solution; Group 2: 1600 mg niclosamide solution; Group 3: Placebo;

Output generated by program 'NIC002_T14_6_VitalSigns_V02_0_0'

Table 14.6: Vital signs

Part C

Group	Parameter	Visit	TP		n	Miss	Mean	SD	Minimum	Lower quartile	Median	Upper quartile	Maximum
	Heart rate [beats/min]	D01	4 h	Raw	4	0	73.8	14.6	52	66.0	80.0	81.5	83
				CFB	4	0	-0.8	9.7	-14	-7.5	1.0	6.0	9
			8 h	Raw	4	0	71.5	7.2	62	66.0	73.0	77.0	78
				CFB	4	0	-3.0	1.4	-4	-4.0	-3.5	-2.0	-1
			12 h	Raw	4	0	73.3	7.6	67	68.0	71.0	78.5	84
				CFB	4	0	-1.3	5.1	-8	-5.0	-0.5	2.5	4
		D03		Raw	4	0	73.5	1.9	72	72.0	73.0	75.0	76
				CFB	4	0	-1.0	8.8	-9	-8.5	-1.5	6.5	8
		D08		Raw	4	0	69.0	8.4	61	62.5	67.5	75.5	80
				CFB	4	0	-5.5	8.0	-17	-11.0	-2.5	0.0	0
		FUP		Raw	4	0	71.5	10.8	60	62.5	71.5	80.5	83
				CFB	4	0	-3.0	10.2	-16	-11.0	-1.5	5.0	7
	Body temperature [°C]	SC		Raw	4	0	36.75	0.29	36.4	36.55	36.75	36.95	37.1
		BL		Raw	4	0	36.70	0.53	36.2	36.30	36.60	37.10	37.4
		D01	Pre-dose	Raw	4	0	36.73	0.40	36.4	36.45	36.60	37.00	37.3
				CFB	4	0	0.03	0.77	-0.7	-0.50	-0.15	0.55	1.1
			1 h	Raw	4	0	36.85	0.42	36.4	36.50	36.85	37.20	37.3
				CFB	4	0	0.15	0.65	-0.3	-0.25	-0.10	0.55	1.1
			2 h	Raw	4	0	37.20	0.35	36.7	37.00	37.30	37.40	37.5
				CFB	4	0	0.50	0.52	-0.1	0.10	0.50	0.90	1.1
			4 h	Raw	4	0	37.03	0.43	36.4	36.75	37.15	37.30	37.4
				CFB	4	0	0.33	0.62	-0.2	-0.10	0.15	0.75	1.2
			8 h	Raw	4	0	37.10	0.16	36.9	37.00	37.10	37.20	37.3
				CFB	4	0	0.40	0.50	-0.3	0.10	0.50	0.70	0.9
			12 h	Raw	4	0	37.15	0.30	36.9	36.90	37.10	37.40	37.5

n: Number of non-missing observations; %: Percentage based on non-missing observations; Miss: Missing observations; SD: Standard deviation; TP: Timepoint of measurement; SC: Screening; BL: Baseline; FUP: Follow-up; D: Day; CFB: Change from baseline; Group 1: 1200 mg niclosamide solution; Group 2: 1600 mg niclosamide solution; Group 3: Placebo;

Output generated by program 'NIC002_T14_6_VitalSigns_V02_0_0'

Table 14.6: Vital signs

Part C

Group	Parameter	Visit	TP		n	Miss	Mean	SD	Minimum	Lower quartile	Median	Upper quartile	Maximum
	Body temperature [°C]	D01	12 h	CFB	4	0	0.45	0.25	0.1	0.30	0.50	0.60	0.7
		D03		Raw	4	0	36.98	0.40	36.4	36.70	37.10	37.25	37.3
				CFB	4	0	0.28	0.64	-0.4	-0.20	0.20	0.75	1.1
		FUP		Raw	4	0	35.93	0.33	35.6	35.65	35.90	36.20	36.3
				CFB	4	0	-0.77	0.39	-1.1	-1.10	-0.85	-0.45	-0.3
	Respiration [breaths/min]	SC		Raw	4	0	13.8	1.7	12	12.5	13.5	15.0	16
		BL		Raw	4	0	12.8	1.5	11	11.5	13.0	14.0	14
		D01	Pre-dose	Raw	4	0	13.5	0.6	13	13.0	13.5	14.0	14
				CFB	4	0	0.8	1.7	-1	-0.5	0.5	2.0	3
			1 h	Raw	4	0	14.0	0.8	13	13.5	14.0	14.5	15
				CFB	4	0	1.3	1.0	0	0.5	1.5	2.0	2
			2 h	Raw	4	0	12.8	1.0	12	12.0	12.5	13.5	14
				CFB	4	0	0.0	0.8	-1	-0.5	0.0	0.5	1
			4 h	Raw	4	0	13.8	1.3	12	13.0	14.0	14.5	15
				CFB	4	0	1.0	2.6	-2	-1.0	1.0	3.0	4
			8 h	Raw	4	0	13.8	1.0	13	13.0	13.5	14.5	15
				CFB	4	0	1.0	1.4	-1	0.0	1.5	2.0	2
			12 h	Raw	4	0	13.8	1.5	12	12.5	14.0	15.0	15
				CFB	4	0	1.0	1.6	-1	0.0	1.0	2.0	3
		D03		Raw	4	0	14.3	0.5	14	14.0	14.0	14.5	15
				CFB	4	0	1.5	1.3	0	0.5	1.5	2.5	3
		D08		Raw	4	0	13.5	2.4	11	11.5	13.5	15.5	16
				CFB	4	0	0.8	1.3	-1	0.0	1.0	1.5	2
		FUP		Raw	4	0	14.8	1.7	13	13.5	14.5	16.0	17
				CFB	4	0	2.0	2.6	-1	0.0	2.0	4.0	5

n: Number of non-missing observations; %: Percentage based on non-missing observations; Miss: Missing observations; SD: Standard deviation; TP: Timepoint of measurement; SC: Screening; BL: Baseline; FUP: Follow-up; D: Day; CFB: Change from baseline; Group 1: 1200 mg niclosamide solution; Group 2: 1600 mg niclosamide solution; Group 3: Placebo;

Output generated by program 'NIC002_T14_6_VitalSigns_V02_0_0'

Table 14.6: Vital signs

Part C

Group	Parameter	Visit	TP		n	Miss	Mean	SD	Minimum	Lower quartile	Median	Upper quartile	Maximum
Group 2	Systolic blood pressure [mmHg]	SC		Raw	4	0	111.3	6.4	106	106.5	109.5	116.0	120
		BL		Raw	4	0	113.8	5.9	109	109.0	112.5	118.5	121
		D01	Pre-dose	Raw	4	0	108.3	6.1	103	104.5	106.5	112.0	117
				CFB	4	0	-5.5	10.3	-14	-13.5	-8.0	2.5	8
			1 h	Raw	4	0	113.5	6.7	104	109.0	115.5	118.0	119
				CFB	4	0	-0.3	4.3	-5	-3.5	-0.5	3.0	5
			2 h	Raw	4	0	110.5	8.6	100	103.5	112.0	117.5	118
				CFB	4	0	-3.3	8.0	-9	-9.0	-6.0	2.5	8
			4 h	Raw	4	0	112.8	3.5	109	110.0	112.5	115.5	117
				CFB	4	0	-1.0	4.1	-7	-3.5	0.5	1.5	2
			8 h	Raw	4	0	105.8	5.7	100	101.0	105.5	110.5	112
				CFB	4	0	-8.0	3.4	-12	-10.5	-8.0	-5.5	-4
			12 h	Raw	4	0	108.3	6.4	101	103.0	108.5	113.5	115
				CFB	4	0	-5.5	1.9	-8	-7.0	-5.0	-4.0	-4
		D03		Raw	4	0	111.5	4.4	108	109.0	110.0	114.0	118
				CFB	4	0	-2.3	7.2	-13	-6.0	1.0	1.5	2
		D08		Raw	4	0	112.0	5.5	104	108.5	114.0	115.5	116
				CFB	4	0	-1.8	4.6	-6	-5.5	-2.5	2.0	4
		FUP		Raw	4	0	114.5	5.8	110	111.0	112.5	118.0	123
				CFB	4	0	0.8	8.0	-11	-4.0	3.5	5.5	7
	Diastolic blood pressure [mmHg]	SC		Raw	4	0	70.3	4.6	65	67.0	70.0	73.5	76
		BL		Raw	4	0	70.5	7.5	63	64.0	71.0	77.0	77

n: Number of non-missing observations; %: Percentage based on non-missing observations; Miss: Missing observations; SD: Standard deviation; TP: Timepoint of measurement; SC: Screening; BL: Baseline; FUP: Follow-up; D: Day; CFB: Change from baseline; Group 1: 1200 mg niclosamide solution; Group 2: 1600 mg niclosamide solution; Group 3: Placebo;

Output generated by program 'NIC002_T14_6_VitalSigns_V02_0_0'

Table 14.6: Vital signs

Part C

Group	Parameter	Visit	TP		n	Miss	Mean	SD	Minimum	Lower quartile	Median	Upper quartile	Maximum
		D01	Pre-dose	Raw	4	0	66.0	6.1	60	61.5	65.0	70.5	74
				CFB	4	0	-4.5	10.1	-14	-12.0	-6.5	3.0	9
			1 h	Raw	4	0	67.3	1.5	66	66.0	67.0	68.5	69
				CFB	4	0	-3.3	8.1	-11	-10.0	-4.0	3.5	6
			2 h	Raw	4	0	64.0	3.6	61	61.5	63.0	66.5	69
				CFB	4	0	-6.5	6.8	-15	-11.5	-6.0	-1.5	1
			4 h	Raw	4	0	61.3	5.4	55	57.5	61.0	65.0	68
				CFB	4	0	-9.3	5.8	-17	-13.0	-8.5	-5.5	-3
			8 h	Raw	4	0	64.0	1.4	63	63.0	63.5	65.0	66
				CFB	4	0	-6.5	6.5	-13	-12.0	-6.5	-1.0	0
			12 h	Raw	4	0	67.3	5.2	61	63.0	68.0	71.5	72
				CFB	4	0	-3.3	2.8	-6	-5.5	-3.5	-1.0	0
		D03		Raw	4	0	66.3	6.3	60	62.0	65.0	70.5	75
				CFB	4	0	-4.3	8.6	-17	-9.5	-0.5	1.0	1
		D08		Raw	4	0	66.3	6.8	60	62.0	64.5	70.5	76
				CFB	4	0	-4.3	5.3	-12	-7.5	-2.0	-1.0	-1
		FUP		Raw	4	0	65.0	6.7	61	61.0	62.0	69.0	75
				CFB	4	0	-5.5	7.0	-16	-9.0	-2.0	-2.0	-2
	Heart rate [beats/min]	SC		Raw	4	0	68.0	11.7	54	58.5	69.0	77.5	80
		BL		Raw	4	0	64.5	11.0	54	57.5	62.0	71.5	80
		D01	Pre-dose	Raw	4	0	68.8	16.7	51	54.5	70.0	83.0	84
				CFB	4	0	4.3	13.5	-12	-4.0	4.0	12.5	21
			1 h	Raw	4	0	80.8	14.1	60	72.0	86.0	89.5	91
				CFB	4	0	16.3	9.5	6	8.5	16.0	24.0	27
			2 h	Raw	4	0	78.5	16.5	57	66.0	81.0	91.0	95
				CFB	4	0	14.0	8.6	3	8.5	14.5	19.5	24

n: Number of non-missing observations; %: Percentage based on non-missing observations; Miss: Missing observations; SD: Standard deviation; TP: Timepoint of measurement; SC: Screening; BL: Baseline; FUP: Follow-up; D: Day; CFB: Change from baseline; Group 1: 1200 mg niclosamide solution; Group 2: 1600 mg niclosamide solution; Group 3: Placebo;

Output generated by program 'NIC002_T14_6_VitalSigns_V02_0_0'

Table 14.6: Vital signs

Part C

Group	Parameter	Visit	TP		n	Miss	Mean	SD	Minimum	Lower quartile	Median	Upper quartile	Maximum
	Heart rate [beats/min]	D01	4 h	Raw	4	0	72.0	11.2	58	63.0	74.0	81.0	82
				CFB	4	0	7.5	9.3	0	2.0	4.5	13.0	21
			8 h	Raw	4	0	69.5	11.0	55	61.0	72.0	78.0	79
				CFB	4	0	5.0	9.1	-3	-1.0	2.5	11.0	18
			12 h	Raw	4	0	69.8	11.6	55	61.0	71.0	78.5	82
				CFB	4	0	5.3	6.0	1	1.5	3.0	9.0	14
		D03		Raw	4	0	71.3	8.7	60	65.5	72.0	77.0	81
				CFB	4	0	6.8	4.6	1	3.5	7.0	10.0	12
		D08		Raw	4	0	66.8	6.0	58	63.0	69.0	70.5	71
				CFB	4	0	2.3	8.6	-10	-3.0	4.5	7.5	10
		FUP		Raw	4	0	72.3	5.4	65	69.0	73.0	75.5	78
				CFB	4	0	7.8	10.3	-7	1.5	10.5	14.0	17
	Body temperature [°C]	SC		Raw	4	0	36.60	0.50	36.0	36.20	36.65	37.00	37.1
		BL		Raw	4	0	36.65	0.17	36.4	36.55	36.70	36.75	36.8
		D01	Pre-dose	Raw	4	0	36.88	0.36	36.4	36.60	36.95	37.15	37.2
				CFB	4	0	0.22	0.36	-0.3	0.00	0.35	0.45	0.5
			1 h	Raw	4	0	36.88	0.44	36.4	36.50	36.90	37.25	37.3
				CFB	4	0	0.22	0.38	-0.3	-0.05	0.35	0.50	0.5
			2 h	Raw	4	0	36.83	0.53	36.4	36.40	36.70	37.25	37.5
				CFB	4	0	0.17	0.46	-0.3	-0.15	0.10	0.50	0.8
			4 h	Raw	4	0	37.03	0.31	36.6	36.80	37.10	37.25	37.3
				CFB	4	0	0.38	0.32	-0.1	0.20	0.50	0.55	0.6
			8 h	Raw	4	0	37.08	0.25	36.8	36.90	37.05	37.25	37.4
				CFB	4	0	0.42	0.13	0.3	0.35	0.40	0.50	0.6
			12 h	Raw	4	0	37.05	0.24	36.8	36.85	37.05	37.25	37.3

n: Number of non-missing observations; %: Percentage based on non-missing observations; Miss: Missing observations; SD: Standard deviation; TP: Timepoint of measurement; SC: Screening; BL: Baseline; FUP: Follow-up; D: Day; CFB: Change from baseline; Group 1: 1200 mg niclosamide solution; Group 2: 1600 mg niclosamide solution; Group 3: Placebo;

Output generated by program 'NIC002_T14_6_VitalSigns_V02_0_0'

Table 14.6: Vital signs

Part C

Group	Parameter	Visit	TP		n	Miss	Mean	SD	Minimum	Lower quartile	Median	Upper quartile	Maximum
	Body temperature [°C]	D01	12 h	CFB	4	0	0.40	0.16	0.2	0.30	0.40	0.50	0.6
		D03		Raw	4	0	36.90	0.35	36.4	36.70	37.00	37.10	37.2
				CFB	4	0	0.25	0.17	0.0	0.15	0.30	0.35	0.4
		FUP		Raw	4	0	36.13	0.39	35.6	35.85	36.20	36.40	36.5
				CFB	4	0	-0.53	0.25	-0.8	-0.70	-0.55	-0.35	-0.2
	Respiration [breaths/min]	SC		Raw	4	0	12.8	1.0	12	12.0	12.5	13.5	14
		BL		Raw	4	0	13.3	1.0	12	12.5	13.5	14.0	14
		D01	Pre-dose	Raw	4	0	13.8	0.5	13	13.5	14.0	14.0	14
				CFB	4	0	0.5	1.3	-1	-0.5	0.5	1.5	2
			1 h	Raw	4	0	13.8	0.5	13	13.5	14.0	14.0	14
				CFB	4	0	0.5	0.6	0	0.0	0.5	1.0	1
			2 h	Raw	4	0	14.0	0.8	13	13.5	14.0	14.5	15
				CFB	4	0	0.8	1.0	0	0.0	0.5	1.5	2
			4 h	Raw	4	0	13.3	0.5	13	13.0	13.0	13.5	14
				CFB	4	0	0.0	1.2	-1	-1.0	0.0	1.0	1
			8 h	Raw	4	0	14.8	1.0	14	14.0	14.5	15.5	16
				CFB	4	0	1.5	1.3	0	0.5	1.5	2.5	3
			12 h	Raw	4	0	15.0	2.2	13	13.5	14.5	16.5	18
				CFB	4	0	1.8	2.5	-1	0.0	1.5	3.5	5
		D03		Raw	4	0	14.5	1.0	14	14.0	14.0	15.0	16
				CFB	4	0	1.3	1.0	0	0.5	1.5	2.0	2
		D08		Raw	4	0	16.5	1.0	16	16.0	16.0	17.0	18
				CFB	4	0	3.3	1.9	2	2.0	2.5	4.5	6
		FUP		Raw	4	0	16.8	1.9	14	15.5	17.5	18.0	18
				CFB	4	0	3.5	2.1	1	2.0	3.5	5.0	6

n: Number of non-missing observations; %: Percentage based on non-missing observations; Miss: Missing observations; SD: Standard deviation; TP: Timepoint of measurement; SC: Screening; BL: Baseline; FUP: Follow-up; D: Day; CFB: Change from baseline; Group 1: 1200 mg niclosamide solution; Group 2: 1600 mg niclosamide solution; Group 3: Placebo;

Output generated by program 'NIC002_T14_6_VitalSigns_V02_0_0'

Table 14.6: Vital signs

Part C

Group	Parameter	Visit	TP		n	Miss	Mean	SD	Minimum	Lower quartile	Median	Upper quartile	Maximum
Group 3	Systolic blood pressure [mmHg]	SC		Raw	4	0	105.5	3.4	101	103.0	106.0	108.0	109
		BL		Raw	4	0	107.0	4.2	101	104.5	108.0	109.5	111
		D01	Pre-dose	Raw	4	0	111.0	3.6	108	108.5	110.0	113.5	116
				CFB	4	0	4.0	3.4	0	1.5	4.0	6.5	8
			1 h	Raw	4	0	108.8	11.8	92	101.0	112.0	116.5	119
				CFB	4	0	1.8	11.9	-16	-5.0	7.0	8.5	9
			2 h	Raw	4	0	100.5	6.0	92	97.0	102.0	104.0	106
				CFB	4	0	-6.5	7.6	-16	-12.5	-5.5	-0.5	1
			4 h	Raw	4	0	109.5	7.9	102	103.0	108.5	116.0	119
				CFB	4	0	2.5	6.0	-6	-1.5	4.0	6.5	8
			8 h	Raw	4	0	103.5	7.9	93	97.5	105.5	109.5	110
				CFB	4	0	-3.5	7.7	-15	-8.0	0.0	1.0	1
			12 h	Raw	4	0	105.3	5.7	97	101.5	107.0	109.0	110
				CFB	4	0	-1.8	7.4	-11	-6.5	-1.5	3.0	7
		D03		Raw	4	0	102.3	3.5	98	99.5	102.5	105.0	106
				CFB	4	0	-4.8	4.1	-10	-7.5	-4.5	-2.0	0
		D08		Raw	4	0	105.0	5.5	101	101.5	103.0	108.5	113
				CFB	4	0	-2.0	9.6	-9	-8.0	-5.5	4.0	12
		FUP		Raw	4	0	108.8	3.3	105	106.5	108.5	111.0	113
				CFB	4	0	1.8	5.7	-6	-2.5	3.0	6.0	7
	Diastolic blood pressure [mmHg]	SC		Raw	4	0	63.5	2.9	61	61.0	63.5	66.0	66
		BL		Raw	4	0	64.3	1.0	63	63.5	64.5	65.0	65

n: Number of non-missing observations; %: Percentage based on non-missing observations; Miss: Missing observations; SD: Standard deviation; TP: Timepoint of measurement; SC: Screening; BL: Baseline; FUP: Follow-up; D: Day; CFB: Change from baseline; Group 1: 1200 mg niclosamide solution; Group 2: 1600 mg niclosamide solution; Group 3: Placebo;

Output generated by program 'NIC002_T14_6_VitalSigns_V02_0_0'

Table 14.6: Vital signs

Part C

Group	Parameter	Visit	TP		n	Miss	Mean	SD	Minimum	Lower quartile	Median	Upper quartile	Maximum
		D01	Pre-dose	Raw	4	0	64.5	4.0	59	61.5	65.5	67.5	68
				CFB	4	0	0.3	4.5	-6	-3.0	1.5	3.5	4
			1 h	Raw	4	0	60.3	5.6	54	56.0	60.0	64.5	67
				CFB	4	0	-4.0	6.1	-10	-8.5	-5.0	0.5	4
			2 h	Raw	4	0	56.3	1.0	55	55.5	56.5	57.0	57
				CFB	4	0	-8.0	1.4	-9	-9.0	-8.5	-7.0	-6
			4 h	Raw	4	0	66.8	6.9	62	62.5	64.0	71.0	77
				CFB	4	0	2.5	7.7	-2	-2.0	-1.0	7.0	14
			8 h	Raw	4	0	60.3	9.0	48	53.5	62.5	67.0	68
				CFB	4	0	-4.0	9.2	-16	-11.0	-2.5	3.0	5
			12 h	Raw	4	0	58.8	5.7	52	55.0	58.5	62.5	66
				CFB	4	0	-5.5	5.3	-12	-9.0	-5.5	-2.0	1
		D03		Raw	4	0	60.3	3.2	57	57.5	60.5	63.0	63
				CFB	4	0	-4.0	3.6	-7	-7.0	-4.5	-1.0	0
		D08		Raw	4	0	60.8	2.5	58	59.0	60.5	62.5	64
				CFB	4	0	-3.5	1.7	-5	-4.5	-4.0	-2.5	-1
		FUP		Raw	4	0	62.3	1.5	61	61.0	62.0	63.5	64
				CFB	4	0	-2.0	2.2	-4	-3.5	-2.5	-0.5	1
	Heart rate [beats/min]	SC		Raw	4	0	68.8	9.7	55	62.0	72.0	75.5	76
		BL		Raw	4	0	65.3	14.8	52	52.5	64.5	78.0	80
		D01	Pre-dose	Raw	4	0	63.8	7.7	55	57.5	64.0	70.0	72
				CFB	4	0	-1.5	8.5	-12	-8.0	-1.0	5.0	8
			1 h	Raw	4	0	71.3	7.6	61	65.5	73.0	77.0	78
				CFB	4	0	6.0	9.4	-4	-1.0	5.0	13.0	18
			2 h	Raw	4	0	67.8	6.0	60	63.0	69.0	72.5	73
				CFB	4	0	2.5	9.7	-7	-5.5	1.5	10.5	14

n: Number of non-missing observations; %: Percentage based on non-missing observations; Miss: Missing observations; SD: Standard deviation; TP: Timepoint of measurement; SC: Screening; BL: Baseline; FUP: Follow-up; D: Day; CFB: Change from baseline; Group 1: 1200 mg niclosamide solution; Group 2: 1600 mg niclosamide solution; Group 3: Placebo;

Output generated by program 'NIC002_T14_6_VitalSigns_V02_0_0'

Table 14.6: Vital signs

Part C

Group	Parameter	Visit	TP		n	Miss	Mean	SD	Minimum	Lower quartile	Median	Upper quartile	Maximum
	Heart rate [beats/min]	D01	4 h	Raw	4	0	67.0	5.7	59	63.0	69.0	71.0	71
				CFB	4	0	1.8	13.9	-13	-9.0	0.5	12.5	19
			8 h	Raw	4	0	64.8	5.9	60	60.5	63.0	69.0	73
				CFB	4	0	-0.5	11.0	-15	-9.0	2.5	8.0	8
			12 h	Raw	4	0	70.3	10.1	58	62.0	71.5	78.5	80
				CFB	4	0	5.0	7.0	-3	0.5	4.5	9.5	14
		D03		Raw	4	0	61.8	10.8	51	52.5	62.0	71.0	72
				CFB	4	0	-3.5	4.2	-8	-7.0	-3.5	0.0	1
		D08		Raw	4	0	67.5	7.0	58	63.0	68.5	72.0	75
				CFB	4	0	2.3	12.1	-12	-6.5	2.0	11.0	17
		FUP		Raw	4	0	69.8	12.1	53	62.0	72.0	77.5	82
				CFB	4	0	4.5	11.0	-7	-3.5	3.0	12.5	19
	Body temperature [°C]	SC		Raw	4	0	36.60	0.48	36.1	36.20	36.60	37.00	37.1
		BL		Raw	4	0	36.30	0.32	36.0	36.05	36.25	36.55	36.7
		D01	Pre-dose	Raw	4	0	36.85	0.31	36.6	36.65	36.75	37.05	37.3
				CFB	4	0	0.55	0.54	-0.1	0.15	0.55	0.95	1.2
			1 h	Raw	4	0	36.90	0.18	36.7	36.75	36.90	37.05	37.1
				CFB	4	0	0.60	0.32	0.3	0.35	0.55	0.85	1.0
			2 h	Raw	4	0	36.88	0.17	36.7	36.75	36.85	37.00	37.1
				CFB	4	0	0.57	0.39	0.2	0.25	0.55	0.90	1.0
			4 h	Raw	4	0	36.68	0.40	36.2	36.35	36.70	37.00	37.1
				CFB	4	0	0.38	0.63	-0.5	0.00	0.50	0.75	1.0
			8 h	Raw	4	0	36.98	0.33	36.6	36.75	36.95	37.20	37.4
				CFB	4	0	0.67	0.52	0.2	0.25	0.60	1.10	1.3
			12 h	Raw	4	0	36.63	0.51	36.0	36.25	36.65	37.00	37.2

n: Number of non-missing observations; %: Percentage based on non-missing observations; Miss: Missing observations; SD: Standard deviation; TP: Timepoint of measurement; SC: Screening; BL: Baseline; FUP: Follow-up; D: Day; CFB: Change from baseline; Group 1: 1200 mg niclosamide solution; Group 2: 1600 mg niclosamide solution; Group 3: Placebo;

Output generated by program 'NIC002_T14_6_VitalSigns_V02_0_0'

Table 14.6: Vital signs

Part C

Group	Parameter	Visit	TP		n	Miss	Mean	SD	Minimum	Lower quartile	Median	Upper quartile	Maximum
	Body temperature [°C]	D01	12 h	CFB	4	0	0.32	0.74	-0.4	-0.30	0.30	0.95	1.1
		D03		Raw	4	0	36.65	0.30	36.4	36.40	36.60	36.90	37.0
				CFB	4	0	0.35	0.49	-0.3	0.05	0.40	0.65	0.9
		FUP		Raw	4	0	35.63	0.60	35.2	35.25	35.40	36.00	36.5
				CFB	4	0	-0.68	0.41	-1.2	-0.95	-0.65	-0.40	-0.2
	Respiration [breaths/min]	SC		Raw	4	0	13.0	0.8	12	12.5	13.0	13.5	14
		BL		Raw	4	0	13.3	1.3	12	12.5	13.0	14.0	15
		D01	Pre-dose	Raw	4	0	12.0	0.8	11	11.5	12.0	12.5	13
				CFB	4	0	-1.3	0.5	-2	-1.5	-1.0	-1.0	-1
			1 h	Raw	4	0	12.5	1.7	10	11.5	13.0	13.5	14
				CFB	4	0	-0.8	1.0	-2	-1.5	-0.5	0.0	0
			2 h	Raw	4	0	12.5	1.3	11	11.5	12.5	13.5	14
				CFB	4	0	-0.8	1.3	-2	-1.5	-1.0	0.0	1
			4 h	Raw	4	0	14.0	1.4	12	13.0	14.5	15.0	15
				CFB	4	0	0.8	1.0	0	0.0	0.5	1.5	2
			8 h	Raw	4	0	13.5	1.3	12	12.5	13.5	14.5	15
				CFB	4	0	0.3	2.5	-3	-1.5	0.5	2.0	3
			12 h	Raw	4	0	14.5	2.6	12	12.5	14.0	16.5	18
				CFB	4	0	1.3	3.6	-2	-1.5	0.5	4.0	6
		D03		Raw	4	0	13.0	1.2	12	12.0	13.0	14.0	14
				CFB	4	0	-0.3	2.2	-3	-2.0	0.0	1.5	2
		D08		Raw	4	0	15.8	1.3	14	15.0	16.0	16.5	17
				CFB	4	0	2.5	2.4	-1	1.0	3.5	4.0	4
		FUP		Raw	4	0	14.3	1.7	12	13.0	14.5	15.5	16
				CFB	4	0	1.0	1.4	0	0.0	0.5	2.0	3

n: Number of non-missing observations; %: Percentage based on non-missing observations; Miss: Missing observations; SD: Standard deviation; TP: Timepoint of measurement; SC: Screening; BL: Baseline; FUP: Follow-up; D: Day; CFB: Change from baseline; Group 1: 1200 mg niclosamide solution; Group 2: 1600 mg niclosamide solution; Group 3: Placebo;

Output generated by program 'NIC002_T14_6_VitalSigns_V02_0_0'

Table 14.7: ECG Results

Part A

Visit	TP		Cohort A1		Cohort A2		Cohort A3		Placebo		Overall	
			n	(%)	n	(%)	n	(%)	n	(%)	n	(%)
SC		Normal	3	(100.0)	3	(100.0)	2	(66.7)	3	(100.0)	11	(91.7)
		Abnormal, ncs	0	(0.0)	0	(0.0)	1	(33.3)	0	(0.0)	1	(8.3)
		Abnormal, cs	0	(0.0)	0	(0.0)	0	(0.0)	0	(0.0)	0	(0.0)
		Missing	0	(0.0)	0	(0.0)	0	(0.0)	0	(0.0)	0	(0.0)
D01 Fast	Pre-dose	Normal	3	(100.0)	3	(100.0)	2	(66.7)	2	(66.7)	10	(83.3)
		Abnormal, ncs	0	(0.0)	0	(0.0)	1	(33.3)	1	(33.3)	2	(16.7)
		Abnormal, cs	0	(0.0)	0	(0.0)	0	(0.0)	0	(0.0)	0	(0.0)
		Missing	0	(0.0)	0	(0.0)	0	(0.0)	0	(0.0)	0	(0.0)
	3h post dose	Normal	3	(100.0)	3	(100.0)	2	(66.7)	2	(66.7)	10	(83.3)
		Abnormal, ncs	0	(0.0)	0	(0.0)	1	(33.3)	1	(33.3)	2	(16.7)
		Abnormal, cs	0	(0.0)	0	(0.0)	0	(0.0)	0	(0.0)	0	(0.0)
		Missing	0	(0.0)	0	(0.0)	0	(0.0)	0	(0.0)	0	(0.0)
FUP Fast		Normal					2	(66.7)	0	(0.0)	2	(50.0)
		Abnormal, ncs					1	(33.3)	1	(100.0)	2	(50.0)
		Abnormal, cs					0	(0.0)	0	(0.0)	0	(0.0)
		Missing					0	(0.0)	0	(0.0)	0	(0.0)
D01 Fed	Pre-dose	Normal					3	(100.0)	1	(100.0)	4	(100.0)
		Abnormal, ncs					0	(0.0)	0	(0.0)	0	(0.0)
		Abnormal, cs					0	(0.0)	0	(0.0)	0	(0.0)
		Missing					0	(0.0)	0	(0.0)	0	(0.0)
	3h post dose	Normal					3	(100.0)	1	(100.0)	4	(100.0)

n: Number of non-missing observations; %: Percentage based on non-missing observations; TP: Timepoint of ECG measurement; Fast/Fed: In Cohort A3 treatment was applied under fasting and fed conditions in the same subjects;

Output generated by program 'NIC002_T14_7_ECG_V02_0_0'

Table 14.7: ECG Results

Part A

Visit	TP		Cohort A1 n	Cohort A1 (%)	Cohort A2 n	Cohort A2 (%)	Cohort A3 n	Cohort A3 (%)	Placebo n	Placebo (%)	Overall n	Overall (%)
	3h post dose	Abnormal, ncs					0	(0.0)	0	(0.0)	0	(0.0)
		Abnormal, cs					0	(0.0)	0	(0.0)	0	(0.0)
		Missing					0	(0.0)	0	(0.0)	0	(0.0)
FUP		Normal	3	(100.0)	3	(100.0)	3	(100.0)	2	(66.7)	11	(91.7)
		Abnormal, ncs	0	(0.0)	0	(0.0)	0	(0.0)	1	(33.3)	1	(8.3)
		Abnormal, cs	0	(0.0)	0	(0.0)	0	(0.0)	0	(0.0)	0	(0.0)
		Missing	0	(0.0)	0	(0.0)	0	(0.0)	0	(0.0)	0	(0.0)

n: Number of non-missing observations; %: Percentage based on non-missing observations; TP: Timepoint of ECG measurement; Fast/Fed: In Cohort A3 treatment was applied under fasting and fed conditions in the same subjects;

Output generated by program 'NIC002_T14_7_ECG_V02_0_0'

Table 14.7: ECG Results

Part B

Visit	TP		Solution 1600mg		Chewing tablet 2000mg		Overall	
			n	(%)	n	(%)	n	(%)
SC		Normal					4	(100.0)
		Abnormal, ncs					0	(0.0)
		Abnormal, cs					0	(0.0)
		Missing					0	(0.0)
D01	Pre-dose	Normal	3	(75.0)	4	(100.0)		
		Abnormal, ncs	1	(25.0)	0	(0.0)		
		Abnormal, cs	0	(0.0)	0	(0.0)		
		Missing	0	(0.0)	0	(0.0)		
	3h post dose	Normal	4	(100.0)	4	(100.0)		
		Abnormal, ncs	0	(0.0)	0	(0.0)		
		Abnormal, cs	0	(0.0)	0	(0.0)		
		Missing	0	(0.0)	0	(0.0)		
FUP		Normal					4	(100.0)
		Abnormal, ncs					0	(0.0)
		Abnormal, cs					0	(0.0)
		Missing					0	(0.0)

n: Number of non-missing observations; %: Percentage based on non-missing observations; TP: Timepoint of ECG measurement; Part B used a cross-over design;

Output generated by program NIC002_T14_7_ECG_V02_0_0

Table 14.7: ECG Results

Part C

Visit		Group 1		Group 2		Group 3		Overall	
		n	(%)	n	(%)	n	(%)	n	(%)
SC	Normal	4	(100.0)	4	(100.0)	3	(75.0)	11	(91.7)
	Abnormal, ncs	0	(0.0)	0	(0.0)	1	(25.0)	1	(8.3)
	Abnormal, cs	0	(0.0)	0	(0.0)	0	(0.0)	0	(0.0)
	Missing	0	(0.0)	0	(0.0)	0	(0.0)	0	(0.0)
D03	Normal	2	(50.0)	4	(100.0)	3	(75.0)	9	(75.0)
	Abnormal, ncs	2	(50.0)	0	(0.0)	1	(25.0)	3	(25.0)
	Abnormal, cs	0	(0.0)	0	(0.0)	0	(0.0)	0	(0.0)
	Missing	0	(0.0)	0	(0.0)	0	(0.0)	0	(0.0)
D08	Normal	4	(100.0)	4	(100.0)	3	(75.0)	11	(91.7)
	Abnormal, ncs	0	(0.0)	0	(0.0)	1	(25.0)	1	(8.3)
	Abnormal, cs	0	(0.0)	0	(0.0)	0	(0.0)	0	(0.0)
	Missing	0	(0.0)	0	(0.0)	0	(0.0)	0	(0.0)
FUP	Normal	4	(100.0)	4	(100.0)	3	(75.0)	11	(91.7)
	Abnormal, ncs	0	(0.0)	0	(0.0)	1	(25.0)	1	(8.3)
	Abnormal, cs	0	(0.0)	0	(0.0)	0	(0.0)	0	(0.0)
	Missing	0	(0.0)	0	(0.0)	0	(0.0)	0	(0.0)

n: Number of non-missing observations; %: Percentage based on non-missing observations;

Output generated by program NIC002_T14_7_ECG_V02_0_0
